# Scientific Foundations for an IUCN Red List of Ecosystems

**DOI:** 10.1371/journal.pone.0062111

**Published:** 2013-05-08

**Authors:** David A. Keith, Jon Paul Rodríguez, Kathryn M. Rodríguez-Clark, Emily Nicholson, Kaisu Aapala, Alfonso Alonso, Marianne Asmussen, Steven Bachman, Alberto Basset, Edmund G. Barrow, John S. Benson, Melanie J. Bishop, Ronald Bonifacio, Thomas M. Brooks, Mark A. Burgman, Patrick Comer, Francisco A. Comín, Franz Essl, Don Faber-Langendoen, Peter G. Fairweather, Robert J. Holdaway, Michael Jennings, Richard T. Kingsford, Rebecca E. Lester, Ralph Mac Nally, Michael A. McCarthy, Justin Moat, María A. Oliveira-Miranda, Phil Pisanu, Brigitte Poulin, Tracey J. Regan, Uwe Riecken, Mark D. Spalding, Sergio Zambrano-Martínez

**Affiliations:** 1 Australian Wetlands Rivers and Landscapes Centre, University of New South Wales, Sydney, New South Wales, Australia; 2 New South Wales Office of Environment and Heritage, Hurstville, New South Wales, Australia; 3 Centro de Ecología, Instituto Venezolano de Investigaciones Científicas, Caracas, Venezuela; 4 Provita, Caracas, Venezuela; 5 EcoHealth Alliance, New York, New York, United States of America; 6 IUCN Commission on Ecosystem Management and IUCN Species Survival Commission, Gland, Switzerland; 7 Centre of Excellence for Environmental Decisions, University of Melbourne, Victoria, Australia; 8 Finnish Environment Institute, Helsinki, Finland; 9 Smithsonian Conservation Biology Institute, National Zoological Park, Washington, D.C., United States of America; 10 Royal Botanic Gardens, Kew, England; 11 Department of Biological and Environmental Science, Ecotekne Center, University of Salento, Lecce, Italy; 12 IUCN Global Ecosystem Management Programme, Nairobi, Kenya; 13 Royal Botanic Gardens Trust, Sydney, New South Wales, Australia; 14 Department of Biological Sciences, Macquarie University, New South Wales, Australia; 15 Science Resource Centre, Department of Environment and Natural Resources, Adelaide, South Australia, Australia; 16 NatureServe, Arlington, Virginia, United States of America; 17 Australian Centre of Excellence for Risk Assessment, University of Melbourne, Victoria, Australia; 18 NatureServe, Boulder, Colorado, United States of America; 19 Pyrenean Institute of Ecology, Zaragoza. Spain; 20 Environment Agency Austria, Vienna, Austria; 21 Department of Conservation Biology, Vegetation and Landscape Ecology, University of Vienna, Vienna, Austria; 22 School of Biological Sciences, Flinders University, Adelaide, South Australia, Australia; 23 Landcare Research, Lincoln, New Zealand; 24 Department of Geography, University of Idaho, Moscow, Idaho, United States of America; 25 School of Life and Environmental Sciences, Deakin University, Warnambool, Victoria, Australia; 26 Australian Centre for Biodiversity, School of Biological Sciences Monash University, Victoria, Australia; 27 Tour du Valat Research Center, Arles, France; 28 German Federal Agency for Nature Conservation, Bonn, Germany; 29 The Nature Conservancy and Conservation Science Group, Department of Zoology, University of Cambridge, Cambridge, England; University of Florida, United States of America

## Abstract

An understanding of risks to biodiversity is needed for planning action to slow current rates of decline and secure ecosystem services for future human use. Although the IUCN Red List criteria provide an effective assessment protocol for species, a standard global assessment of risks to higher levels of biodiversity is currently limited. In 2008, IUCN initiated development of risk assessment criteria to support a global Red List of ecosystems. We present a new conceptual model for ecosystem risk assessment founded on a synthesis of relevant ecological theories. To support the model, we review key elements of ecosystem definition and introduce the concept of ecosystem collapse, an analogue of species extinction. The model identifies four distributional and functional symptoms of ecosystem risk as a basis for assessment criteria: A) rates of decline in ecosystem distribution; B) restricted distributions with continuing declines or threats; C) rates of environmental (abiotic) degradation; and D) rates of disruption to biotic processes. A fifth criterion, E) quantitative estimates of the risk of ecosystem collapse, enables integrated assessment of multiple processes and provides a conceptual anchor for the other criteria. We present the theoretical rationale for the construction and interpretation of each criterion. The assessment protocol and threat categories mirror those of the IUCN Red List of species. A trial of the protocol on terrestrial, subterranean, freshwater and marine ecosystems from around the world shows that its concepts are workable and its outcomes are robust, that required data are available, and that results are consistent with assessments carried out by local experts and authorities. The new protocol provides a consistent, practical and theoretically grounded framework for establishing a systematic Red List of the world’s ecosystems. This will complement the Red List of species and strengthen global capacity to report on and monitor the status of biodiversity

## Introduction

The world’s biodiversity continues to diminish as human populations and activities expand [1], [2], [3], [4]. A sound understanding of risks to biodiversity is needed to plan actions to slow rates of decline, secure future ecosystem services for human use and foster investment in ecosystem management [5]. By identifying species most at risk of extinction, the IUCN Red List criteria [6] inform governments and society about the current status of biodiversity [7] and trends in extinction risks [8], and also provide data with which to formulate priorities and management strategies for conservation [9].

Despite the strengths and widespread acceptance of the IUCN Red List of Threatened Species [10], the need for biodiversity assessments that address higher levels of biological organisation has long been recognised [11], [12]. This need is reflected in the emergence of recent national and regional listings of ecosystems, communities and habitats [13], and recent resolutions by the World Conservation Congress to develop quantitative criteria for assessing ecosystems [14]. Opportunities to meet the need for ecosystem risk assessment are supported by emerging theories on ecosystem dynamics and function [15], [16], [17], methods for handling uncertainty [18], [19], ecosystem-specific measures of ecological change [20], [21], [22] and developing temporal data sets on ecosystem distribution and processes [23], [24].

The scientific challenges in building a unified risk assessment framework for ecosystems are likely greater than those faced during development of Red List criteria for species [25]. Foremost among these challenges is balancing the need for specificity (to support consistent, quantitative evaluation of risk) with the need for generality (to support application of common theoretical concepts across the wide variety of ecosystems). To achieve this trade-off, and to address other scientific challenges outlined below, we first construct a framework comprising generic concepts and models derived from relevant ecological theories, and second, propose requirements or ‘standards’ for translating the concepts into practical assessments, illustrated by examples. Our intent is to outline the concepts in enough detail that applications will be consistent in a very broad range of contexts. We also aim to avoid prescriptive or arbitrarily exact definitions that would exclude or misclassify many cases or prove to be unworkable in the variety of contexts in which ecosystem assessment is required. Although we recognise that this approach carries some risk of inconsistent application between ecosystems defined in different regions or environments, we believe this trade-off is necessary to achieve the generality and flexibility required of a globally applicable risk assessment protocol.

Early development of Red List criteria for ecosystems drew from analogies with species criteria and existing protocols designed for regional applications [12], [13]. Existing risk assessment protocols were primarily focussed on terrestrial plant communities and were national or regional in scope (e.g. [26], [27], [28]). Their assessment of declines in ecological function was mostly qualitative and they applied different treatments of common risk factors such as rates of decline and restricted distribution [13]. The reasons for differences between existing protocols were difficult to understand because their documentation provides limited theoretical rationale for their construction [13]. Our aim here is to develop a generic assessment method based on an explicit conceptual model for ecosystem risk. The intended scope of assessments spans terrestrial, subterranean, aquatic continental and marine realms, and transitional environments at their interfaces. The scope also includes semi-natural and cultural environments [29]. We first elucidate the goals and key concepts that underpin our approach to risk assessment. We then describe the conceptual model for assessing risks of ecosystem collapse, and justify the construction of risk assessment criteria with reference to relevant ecological theory. Finally, we trial the criteria on contrasting ecosystems from around the world to evaluate their applicability and performance relative to existing assessments, and to identify challenges for future research.

## Goals and Key Concepts of Risk Assessment

### Goals of a Red List of Ecosystems

Ideally, a Red List may be expected to identify ecosystems at risk of losing biodiversity, ecological functions and/or ecosystems services, since all three are inter-related and important objects for conservation [30]. However, an approach that simultaneously seeks to assess risks to all three is fraught with complexities in the relationships among them (we elaborate on these in the next section). Ecological changes that promote some ecosystem services may be detrimental to biodiversity or vice versa, leading to logical conflicts if a single assessment were to conflate biodiversity, functions and services. Therefore, to provide essential conceptual clarity for a simple and widely applicable risk assessment process, we have chosen to focus on risks to biodiversity as the primary goal for a Red List of Ecosystems, since this underpins many ecosystem functions [30], [31]. Under this approach, changes in functions and services may contribute to assessments of risk if they threaten the persistence of characteristic ecosystem biota, but not if they are unlikely to generate a biotic response.

#### Complex relationships among biodiversity, ecosystem functions, and services

There is growing empirical and theoretical evidence that ecosystem functions and services are linked with biodiversity [30], [32], [33], [34], [35], [36], [37]. However, several complexities in these relationships preclude presuming that one can serve as a proxy for the others or that they can be conflated into a single objective for risk analysis. Firstly, functional roles of many species are only detectable at particular spatial and temporal scales [16], [37]. Some ecosystem services may be initially insensitive to biotic loss because multiple species may perform similar functions in a replaceable manner (functional redundancy); some species may contribute little to overall function; or some functions may depend on abiotic components of ecosystems [34]. Conversely, small declines in species’ abundance can seriously disrupt or cease the supply of critical ecosystem services before any characteristic biota is actually lost [38]. The subset of biota that sustain functions and services is therefore uncertain, scale-dependent and temporally variable within any ecosystem. Consequently the relationship between biodiversity and many ecosystem services is poorly defined [30].

Secondly, the identification and valuation of ecosystem services depend on social, cultural and economic factors, and may vary locally [39]. Thus risks to ecosystem services may not always be concordant with risks to biodiversity; some processes that promote services may increase risks to biodiversity.

Thirdly, whether particular directional changes in ecosystem function or the abiotic environment are ‘good’ or ‘bad’ for conservation often involves local value judgements [16]. In contrast, the loss of characteristic biota is unambiguously negative for conservation goals [40], and therefore provides a clear and simple objective for risk assessment.

### Units of Assessment

Our purpose here is to develop a robust and generic risk assessment method that can be applied to any internally consistent classification of ecosystems. A generic risk assessment protocol requires clearly defined assessment units, yet it also requires flexibility to assess risks across contrasting ecosystems that vary greatly in biological and environmental characteristics, as well as scales of organisation, and for which varying levels of knowledge are available. Therefore we first propose an operational definition of ecosystems to guide delineation of assessment units that will be informative about the conservation status of higher levels of biodiversity. Second, we identify the potential sensitivities of risk assessment to scale of the assessment units and suggest a suitable level of ecosystem classification for global biodiversity assessment. Finally, we outline a number of requirements for ecosystem description that are necessary to translate the operational definition into a practical assessment unit.

#### Operational definition of ecosystems

In Appendix S1 we define terms used to describe ecosystems and other concepts required for risk assessment. We use the term ‘ecosystem types’ for units of assessment that represent complexes of organisms and their associated physical environment within an area (after [41]). Although many authors have proposed revised definitions of an ecosystem, most encapsulate four essential elements implicit in Tansley’s original concept [42]: i) a biotic complex or assemblage of species; ii) an associated abiotic environment or complex; iii) the interactions within and between those complexes; and iv) a physical space in which these operate. Thus, ecosystems are defined by a degree of uniqueness in composition and processes (involving the biota and the environment) and a spatial boundary. For our purposes, we regard other terms applied in conservation assessments, such as ‘ecological communities’, ‘habitats’, ‘biotopes’ and (largely in the terrestrial context) ‘vegetation types’, as operational synonyms of ‘ecosystem types’ [13].

#### The influence of scale

The unique features that define individual ecosystem types are scale-dependent. The four key elements of an ecosystem type may be organised on spatial, temporal and thematic scales [43]. Spatially, ecosystems vary in extent and grain size from water droplets to oceans [44], with boundaries delimited physically or functionally [45]. Temporally, ecosystems may develop, persist and change over time frames that vary from hours to millenia. They appear stable at some temporal scales, while undergoing trends or fluctuations at others [44]. Thematic scale refers to similarity of features within and between ecosystems, their degree of uniqueness in composition and processes, which may be depicted hierarchically [46].

The outcomes of ecosystem assessments are also likely to depend on spatial, temporal and thematic scales [13], [43]. Nonetheless, the applicability of the ecosystem concept across terrestrial, subterranean, freshwater and marine environments at any scale [47] offers important flexibility and generality for risk assessment. The diversity of conservation planning needs will likely require ecosystem risk assessments at multiple scales from global to local.

We do not consider ecological classifications in detail here, although we recognise that a global Red List will require a global classification of ecosystem types [12], [14]. To provide initial guidance, we suggest that a classification comprising a few hundred ecosystem types on each continent and in each ocean basin will be a practical thematic scale for global assessment. These globally recognisable ecosystem types should be finer units than ecoregions and biomes [48], [49], and should encompass variation that may be recognisable as distinct communities at regional and local scales. For example, a classification of approximately 500 assessment units has been adopted for an assessment of terrestrial ecosystems across the Americas [14]. These units correspond to the Macrogroup level of vegetation classification (see [50], [51]). Similar classifications may prove suitable for global assessments of freshwater and marine ecosystems. We anticipate that sub-global ecosystem assessments will be most useful when based on established national or regional classifications that are cross-referenced to global assessment units and justified as suitable proxies for ecological assemblages (see examples in Appendix S2).

### Describing Ecosystem Types

Since no universally accepted global taxonomy of ecosystems yet exists, lucid description of the assessment unit of interest is an important first step for a repeatable assessment process. Following from our operational definition of an ecosystem, we suggest that a description should address the four elements that define the *identity* of the ecosystem type (Table 1): the characteristic native biota; abiotic environment, key processes and interactions; and spatial distribution [41], [45]. For each of these elements, a description should: i) justify conformity of an ecosystem type with the operational definition; and ii) elucidate the scale of the assessment unit, its salient and unique features, and its distinctions and relationships with other units. Essential supporting information includes reference to the classification and more detailed descriptions from which the assessment unit was derived, as well as cross-referencing to the IUCN habitat classification to elucidate context and facilitate comparisons. A description should furthermore establish reference states and appropriate proxies of defining features that will be used to diagnose loss of biodiversity from the ecosystem (we address this in the section on Ecosystem Collapse). Detailed case studies (Appendix S2) illustrate the translation of our operational ecosystem definition into workable assessment units, using a variety of existing ecosystem classification schemes across a wide range of terrestrial, freshwater, marine and subterranean ecosystems.

**Table 1:** Description template for ecosystem types.

Elements of operational definition	Components of ecosystem description
1. Characteristic assemblage of biota	Identify defining biotic features
	a) List diagnostic native species and describe their relative dominance and uniqueness
	b) List functional component of characteristic biota and identify their roles
	c) Describe limits of variability in the ecosystem biota
	d) Exemplar photographs
2. Associated physical environment	Identify defining abiotic features (e.g. climate, terrain, water chemistry, depth, turbidity, ocean currents, substrate, etc.)
	a) Text descriptions and citations for characteristic states or values of abiotic variables
	b) Graphical descriptions of abiotic variables
	c) Exemplar photographs
3. Processes & interactions between components	Describe key ecosystem drivers and threatening processes
– among biota	a) Text descriptions and citations
– between biota & environment	b) Diagrammatic process models
	c) Exemplar photographs
4. Spatial extent	Describe distribution and extent
	a) Maps
	b) Estimates of area
	c) Time series, projections (past, present, future)
5. Classification context	Cross-references to relevant ecological classifications
	a) Source classification
	b) IUCN habitat classification
	c) Ecoregional classifications
6. Reference state(s)	Describe ecosystem-specific point of collapse
	a) Proxy variable
	b) Bounded threshold of collapse

See Appendix S2 for examples.

#### Characteristic native biota

The concept of ‘characteristic native biota’ (Appendix S1) is central to risk assessment in ecosystems and therefore to their description (Table 1): we define this as a subset of all native biota that either distinguishes an ecosystem from others (diagnostic components) or plays a non-trivial role in ecosystem function and persistence of other biota (functional components). Conversely, characteristic biota exclude uncommon or vagrant species that contribute little to function and may be more common in other ecosystems. The diagnostic components of an ecosystem exhibit a high abundance or frequency within it, relative to other ecosystems [52], and therefore demonstrate a level of compositional uniqueness within the domain of an assessment (i.e. global, regional, national).

The functional components of characteristic biota include species that drive ecosystem dynamics as ecosystem engineers, trophic or structural dominants, or functionally unique elements (see examples, Appendix S2). These essential components of ecosystem identity play key roles in ecosystem organisation by providing conditions or resources essential for species to complete their life cycles or by helping to maintain niche diversity or other mechanisms of coexistence. Typically they are common within the ecosystem [53], although sometimes they may be more common in other ecosystems. Examples include predators that structure animal communities in many ecosystems, tree species that create differential microclimates in their canopies or at ground level, reef-building corals and oysters that promote niche diversity for cohabiting fish and macro-invertebrates, nurse plants and those that provide sites for predator avoidance, flammable plants that promote recurring fires, etc.

Thus, characteristic native biota may be described using taxonomic or functional traits. To be useful for risk assessment, descriptions need not include exhaustive species inventories. However, they should demonstrate a level a compositional uniqueness and identify functionally important elements salient to the assessment of each ecosystem type (see Appendix S2 for examples).

#### Abiotic characteristics

Abiotic features are the second essential element of the ecosystem concept. Descriptions should similarly identify salient abiotic features that influence the distribution or function of an ecosystem type, define its natural range of variability and differentiate it from other systems (Table 1). For terrestrial ecosystems, salient abiotic features may include substrates, soils and landforms, as well as ranges of key climatic variables, while those of freshwater and marine ecosystems may include key aspects of water regimes, tides, currents, climatic factors and physical and chemical properties of the water column (see Appendix S2 for examples).

#### Characteristic processes and interactions

Characteristic ecological processes are a third element important to include in ecosystem description for risk assessment (Table 1). A qualitative understanding of the processes that govern ecosystem dynamics is essential for assessing risks related to functional declines. Again, to be practical this element of ecosystem description should not require extensive knowledge of interaction networks or fluxes of matter and energy: many ecosystems lack direct studies of ecological processes. However, generic mechanisms of ecosystem dynamics can often be inferred from related systems. For example, pelagic marine systems are invariably dominated by trophic interactions in which elements of the main trophic levels are known, even if most particular predator-prey relationships are not. Similarly, the tree/grass dynamic in savannas throughout the world is influenced by fire regimes, herbivores and rainfall, although their relative roles may vary between savanna types. In many cases, a broad understanding of ecosystem processes may be a sufficient basis for assigning an ecosystem to a risk category, especially if key threats to ecosystem persistence can be identified. The basic requirements for assessments based on ecological processes are to identify the major drivers of change, deduce reference states and infer measureable symptoms of ecosystem transformation (see next section).

Simple diagrammatic process models [54] are a useful means of summarising understanding of salient ecosystem processes for risk assessment (see examples in Appendix S2). These models may be structured to describe transitions among alternative states of an ecosystem (e.g. [55], [56]) or to show cause-effect dependencies between components and processes within the system (e.g. [57]). More complex models may identify variables and thresholds that define alternative states, pathways of transition between them and conditions or processes that drive the transitions (e.g. [58], [59]). Detailed simulation models can predict the relative dominance of alternative states, given estimates of environmental drivers, although these have been developed for relatively few ecosystems [60], [61].

#### Spatial distribution

Finally, a description of ecosystem properties requires their extent to be specified and bounded at a given observational resolution [62]. The spatial element of ecosystem definition is best described through maps or inventories of locations (Table 1). Mapping is available for many ecosystem types in terrestrial, freshwater aquatic and marine benthic environments, either derived from remote sensing, biophysical distribution models or a combination of both (see examples in Appendix S2). The spatial features of some types of ecosystem, such as pelagic fisheries, are inherently uncertain and dynamic over relatively short time scales, and hence spatial data are scarce and distributions can only be described at very coarse levels of resolution. Given the diversity of methods and maps available, an important aspect of this element of description is to justify why a particular map base is an adequate representation of the ecosystem distribution.

## Ecosystem Collapse and Risk Assessment

**Figure 1:**
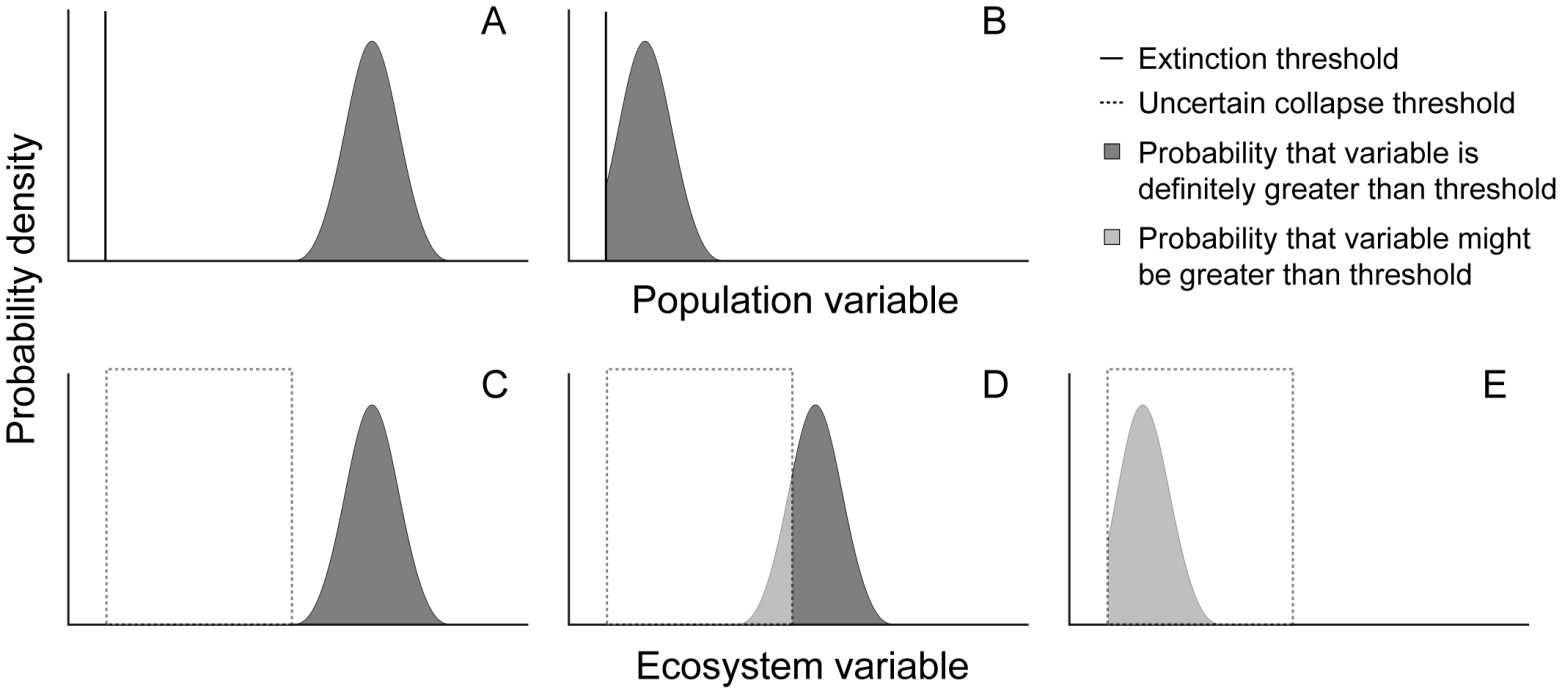
Probability density functions for the population and ecosystem variables that measure proximity to the thresholds that define species extinction (A, B) and ecosystem collapse (C, D). The probability density functions represent uncertainty in the measurement of the variables. For species, the population threshold that defines extinction is known with certainty (e.g. zero abundance of a species, defined by the vertical line in A and B). In A, the estimated population is definitely greater than the extinction threshold, so there is no doubt that the species is extant. Alternatively, the probability that the abundance is above the threshold (the area under the curve) might be less than one (B), in which case the species could be extinct or extant. The shaded area is the probability that the species remains extant. For ecosystems, the x-axis could represent spatial distribution, number of species, water quality, etc. In contrast to species, uncertainty about the definition of ecosystem collapse leads to a range of possible values for this threshold (dashed box in C and D). The ecosystem variable is above this upper bound in some cases (C), so there is no doubt that the ecosystem persists. Alternatively, probable values for the ecosystem variable might intersect the uncertain threshold (D), in which case the ecosystem may be collapsed or not. In this case, there is some probability that the ecosystem parameter is above the upper bound of the threshold (shaded dark grey), which places a lower bound on the probability that the ecosystem persists (i.e. that it has not collapsed). There is an additional probability (pale grey) that the ecosystem parameter is above the threshold that depends on the amount of uncertainty in the threshold (i.e. width of the box). The sum of these two probabilities places an upper bound on the probability ecosystem persists. With further deterioration (E), the lower bound on the probability of ecosystem persistence is zero (no dark shading) and the upper bound is the pale shaded area.

The protocol for Red Listing must synthesise the diverse evidence, causes, mechanisms and pathways of ecosystem decline within a generic risk assessment framework [63]. To estimate ‘risk’ – the probability of an adverse outcome over a specified time frame [64] – this framework must first define an endpoint to ecosystem decline (the adverse outcome). For species and populations, this endpoint is extinction, when the last individual dies [25]. Conceptually, species extinction appears to be a relatively discrete endpoint, although its measurement may be uncertain (Fig. 1a–b). Extinction may be uncertain because, for example, individuals may escape detection [65]. For ecosystems, an analogous endpoint may be identified in terms of distribution size – when the last occurrence of an ecosystem disappears. However, closer examination reveals that the concept of a discrete endpoint (both for species and ecosystems) is problematic for several reasons that we discuss in the next section.

### Uncertainties in the ‘Endpoints’ for Ecological Risk Assessment

The theory of risk assessment assumes a discrete endpoint or event (Fig. 1a–b) affecting the asset under evaluation [64]. Practical implementations of the theory, however, confront uncertainties in the definition of the asset itself, as well as endpoint threshold. For example, the boundaries of related species or ecosystem types are inherently vague [66]. Uncertainties include imperfect knowledge of character variation among individuals of species or occurrences of ecosystems, continuous rather than discrete patterns of natural variability between taxonomic units, and inconsistent taxon concepts that vary through time. These sources of uncertainty are likely greater for ecosystems than species, but they exist in both cases. Thus, the hazards addressed in a risk assessment are more accurately portrayed as bounded ranges than discrete endpoints (Fig. 1c–e).

The uncertainties become more conspicuous when considering endpoints in functional decline, than declines in distribution (Fig. 1) [12], [13]. For ecosystems, many characteristic features of an ecosystem may be gone long before the last characteristic species disappears from the last ecosystem occurrence (‘assemblage extinction’ of [53]). Some detrimental ecosystem changes may result from loss of individuals from the system, not loss of particular species [53]. In addition, ecosystems may not disappear, but rather transform into novel ecosystems with different characteristic biota and mechanisms of self-organisation [67]. Transition points from original to novel ecosystems, unlike theoretically discrete events, are inherently uncertain [66], though may still be estimated within plausible bounds (Fig. 1). An obvious analogue for this process in species is transformation by hybridisation [68], but more widespread vagueness in extinction becomes apparent when species concepts are viewed in the context of an artificial and continually developing taxonomy superimposed on dynamic constellations of genes of genotypes. Moreover, different ecosystems will have different points of transition to novel systems because they differ in resilience and natural variability [69], [70], [71], are threatened by different processes, and exhibit different symptoms of decline.

The definition of the endpoint to ecosystem decline needs to be sufficiently discrete to permit assessment of risk, but sufficiently general to encompass the broad range of contexts in which risk assessments are needed. To deal with this trade-off, we first propose a generic operational definition for an endpoint to ecosystem decline. Second, we provide guidance on how the operational definition of collapse may be translated for specific ecosystem types into an explicit threshold that recognises inherent uncertainties. Third, we propose a conceptual model of ecosystem risk as a basis for design of a protocol for assessing the risk of collapse.

### Ecosystem Collapse: an Operational Definition

To acknowledge the contrasts with species extinctions, we propose the concept of “ecosystem collapse” as transition beyond a bounded threshold in one or more variables that define the identity of the ecosystem. Collapse is thus a transformation of identity, loss of defining features, and replacement by a novel ecosystem. It occurs when all occurrences lose defining biotic or abiotic features, and characteristic native biota are no longer sustained. For example, collapse may occur when most of the diagnostic components of the characteristic biota are lost from the system, or when functional components (biota that perform key roles in ecosystem organisation) are greatly reduced in abundance and lose the ability to recruit. Chronic changes in nutrient cycling, disturbance regimes, connectivity or other ecological processes (biotic or abiotic) that sustain the characteristic biota may also signal ecosystem collapse. Novel ecosystems may retain some or many biotic and abiotic features of the pre-collapse systems from which they were derived, but their relative abundances will differ, they may be organised and interact in different ways and the composition, structure and/or function of the new system has moved outside the natural range of spatial and temporal variability of the old one. A collapsed ecosystem may have the capacity to recover given a long time scale, or with restoration, but in many systems recovery will not be possible.

In the next section, we illustrate how the operational definition of ecosystem collapse can be translated into practical applications. This is most easily done for ecosystems that have already collapsed and where time series data exist for relevant variables (Appendix S2.5). However, as shown in other case studies (Appendix S2), it will often be possible to infer characteristics of collapse from localised occurrences within the ecosystem distribution, even if the majority of the ecosystem remains extant and functional.

Transitions to collapse may be gradual, sudden, linear, non-linear, deterministic or highly stochastic [54], [72], [73], [74], [75]. These include regime shifts [72], but also other types of transitions that may not involve feedbacks. The dominant dynamic in an ecosystem will depend on abiotic or external influences (e.g. weather patterns or human disturbance), internal biotic processes (e.g. competition, predation, epidemics), historical legacies, and spatial context [76], [77]. An ecosystem may thus be driven to collapse by any of several different threatening processes and through multiple alternative pathways [54]. Symptoms that an ecosystem is at risk of collapse may differ, depending on the characteristics that define the ecosystem identity, the nature of threatening processes and the pathways of decline that these generate.

#### A modern example of ecosystem collapse

The Aral Sea (see Appendix 2.5), the world’s fourth largest continental water body, is fed by two major rivers, the Syr Dar’ya and Amu Dar’ya, in central Asia. Its characteristic native biota includes freshwater fish (20 species), a unique invertebrate fauna (>150 species) and shoreline reedbeds, which provide habitat for waterbirds including migratory species. Hydrologically, the sea was approximately stable during 1911–1960, with inflows balancing net evaporation [78]. Intensification of water extraction to support expansion of irrigated agriculture lead to shrinkage and salinisation of the sea. By 2005, only 28 aquatic species (including fish) were recorded, reed beds had dried and disappeared, the sea had contracted to a fraction of its former volume and surface area, and salinity had increased ten-fold. Consistent with our operational definition of ecosystem collapse, these changes suggest the Aral Sea had undergone a transformation of identity, lost many of its defining features (aquatic biota, reedbeds, waterbirds, hydrological balance and brackish hydrochemistry) and had been replaced by novel ecosystems (saline lakes and desert plains). Under this interpretation, collapse occurred before the volume and surface area of standing water declined to zero. Although the exact point of ecosystem collapse is uncertain, time series data for several variables are suitable for defining a functional reference state (prior to onset of change from 1960) and a bounded threshold of collapse (cf. Fig. 1c–e), assuming this occurred sometime during 1976–1989 when most of the biota disappeared (Table 2).

**Table 2:** Biotic and abiotic variables for assessing functional decline in the Aral Sea ecosystem, their reference values when the ecosystem was in a functional state (between 1911 and 1960) and bounded thresholds that define the collapsed state, assuming collapse occurred between 1976 and 1989.

	Functional reference state(1911–1960)	Bounded threshold of collapse (reference data1976, 1989)
Fish species richness and commercial catch (t)	20, 44,000	4–10, 0
Sea volume (km^3^)	1,089	364–763
Sea surface area (km^2^)	67,499	39,734–55,700
Average salinity (g.l^−1^)	10	14–30

Data from [78]. Further details in Appendix 2.5).

The choice of available variables for assessing the status of the ecosystem will depend on how closely they represent the ecosystem’s defining features, the quantity and quality of the data, and the sensitivity of alternative variables to ecological change. Of those listed above, fish species richness and abundance may be the most proximal biotic variable to the features that define the identity of the Aral Sea ecosystem. Sea volume may be a reasonable abiotic proxy, because volume is functionally linked with salinity, which in turn mediates persistence of the characteristic freshwater/brackish aquatic fauna. Sea surface area is less directly related to these features and processes, but can be readily estimated by remote sensing and may be useful for assessment when data are unavailable for other variables.

Collapse of the Aral Sea ecosystem may or may not be reversible. While it may be possible to restore the hydrological regime over a small part of the former sea [78], some components of the characteristic biota are apparently extinct (e.g. the Aral salmon, *Salmo trutta aralensis*), preventing reconstruction of the pre-collapse ecosystem.

### Risk Assessment Model

**Figure 2:**
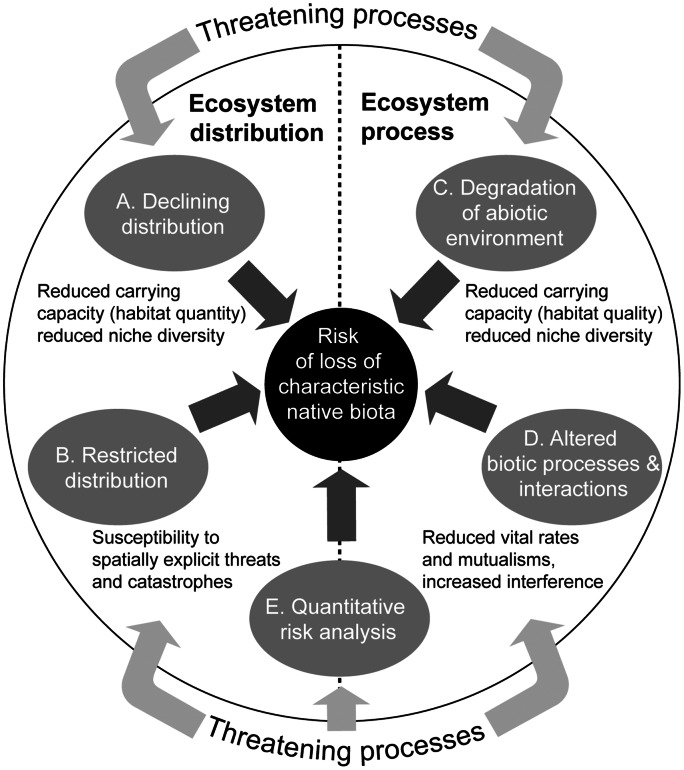
Mechanisms of ecosystem collapse, and symptoms of collapse risk.

Our risk assessment model (Fig. 2) groups symptoms of ecosystem collapse into four major types, and identifies the corresponding mechanisms that link the symptoms to the risk that an ecosystem will lose its defining features (characteristic native biota and/or ecological processes). Two of the four mechanisms produce distributional symptoms (Fig. 2): A) ongoing declines in distribution, which reduce carrying capacity for dependent biota; and B) restricted distribution, which predisposes the system to spatially explicit threats. Two other mechanisms produce functional symptoms (Fig. 2): C) degradation of the abiotic environment, reducing habitat quality or abiotic niche diversity for component biota; and D) disruption of biotic processes and interactions, resulting in the loss of mutualisms, biotic niche diversity, or exclusion of some component biota by others. Interactions between two or more of these four contrasting mechanisms may produce additional symptoms of transition towards ecosystem collapse. Multiple mechanisms and their interactions may be integrated into a simulation model of ecosystem dynamics to produce quantitative estimates of the risk of collapse (E). These five groups of symptoms form the basis of ecosystem Red List criteria (Table 3).

**Table 3:** IUCN Red List criteria for ecosystems, version 2.0.

			Critically Endangered	Endangered	Vulnerable
A	Reduction in geographic distribution over ANY of following periods:				
	1	Present (over the past 50 years)	≥80%	≥50%	≥30%
	2a	Future (over the next 50 years)	≥80%	≥50%	≥30%
	2b	Future (over any 50 year period including the present and future)	≥80%	≥50%	≥30%
	3	Historic (since 1750)	≥90%	≥70%	≥50%
B	Restricted geographic distribution indicated by EITHER:				
	1	Extent of a minimum convex polygon enclosing all occurrences (Extent ofOccurrence), OR	≤2,000 km^2^	≤20,000 km^2^	≤50,000 km^2^
	2	The number of 10×10 km grid cells occupied (Area of Occupancy)	≤2	≤20	≤50
		AND at least one of the following (a-c):			
		(a) An observed or inferred continuing decline in EITHER:			
		i. a measure of spatial extent appropriate to the ecosystem; OR			
		ii. a measure of environmental quality appropriate to characteristicbiota of the ecosystem; OR			
		iii. a measure of disruption to biotic interactions appropriate to thecharacteristic biota of the ecosystem			
		(b) Observed or inferred threatening processes that are likely to cause continuing declines in either geographic distribution, environmental quality or biotic interactions within the next 20 years			
		(c) Ecosystem exists at …	1 location	≤5 locations	≤10 locations
	3	A very small number of locations (generally fewer than 5) AND			
		prone to the effects of human activities or stochastic events within a very short time period in an uncertain future, and thus capable of collapse or becoming Critically Endangered within a very short time period			
C	1	Environmental degradation over the past 50 years based on change in an abiotic variable* affecting…	≥80% extent with ≥80% relative severity**	≥50% extent with ≥80% relative severity	≥50% extent with ≥50% relative severity
				≥80% extent with ≥50% relative severity	≥80% extent with ≥30% relative severity
					≥30% extent with ≥80% relative severity
	2	Environmental degradation over the next 50 years, or any 50-year periodincluding the present and future, based on change in an abiotic variable affecting…	≥80% extent with ≥80% relative severity	≥50% extent with ≥80% relative severity	≥50% extent with ≥50% relative severity
				≥80% extent with ≥50% relative severity	≥80% extent with ≥30% relative severity
					≥30% extent with ≥80% relative severity
	3	Environmental degradation since 1750 based on change in an abiotic variable affecting…	≥90% extent with ≥90% relative severity	≥70% extent with ≥90% relative severity	≥70% extent with ≥70% relative severity
				≥90% extent with ≥70% relative severity	≥90% extent with ≥50% relative severity
					≥50% extent with ≥90% relative severity
D	1	Disruption of biotic processes or interactions over the past 50 years based onchange in a biotic variable* affecting…	≥80% extent with ≥80% relative severity**	≥50% extent with ≥80% relative severity	≥50% extent with ≥50% relative severity
				≥80% extent with ≥50% relative severity	≥80% extent with ≥30% relative severity
					≥30% extent with ≥80% relative severity
	2	Disruption of biotic processes or interactions over the next 50 years, or any 50-year period including the present and future, based on change in a biotic variable affecting…	≥80% extent with ≥80% relative severity	≥50% extent with ≥80% relative severity	≥50% extent with ≥50% relative severity
				≥80% extent with ≥50% relative severity	≥80% extent with ≥30% relative severity
					≥30% extent with ≥80% relative severity
	3	Disruption of biotic processes or interactions since 1750 based on change in a biotic variable affecting…	≥90% extent with ≥90% relative severity	≥70% extent with ≥90% relative severity	≥70% extent with ≥70% relative severity
				≥90% extent with ≥70% relative severity	≥90% extent with ≥50% relative severity
					≥50% extent with ≥90% relative severity
E		Quantitative analysis that estimates the probability of ecosystem collapse to be…	≥50% within 50 years	≥20% within 50 years	≥10% within 100 years

These supercede an earlier set of four criteria [12]. Refer to Appendix S1 for definitions of terms.

* see text for guidance on selection of variable appropriate to the characteristic native biota of the ecosystem.

** see text and Fig. 6 for explanation of relative severity of decline.

#### Protocol structure

**Figure 3:**
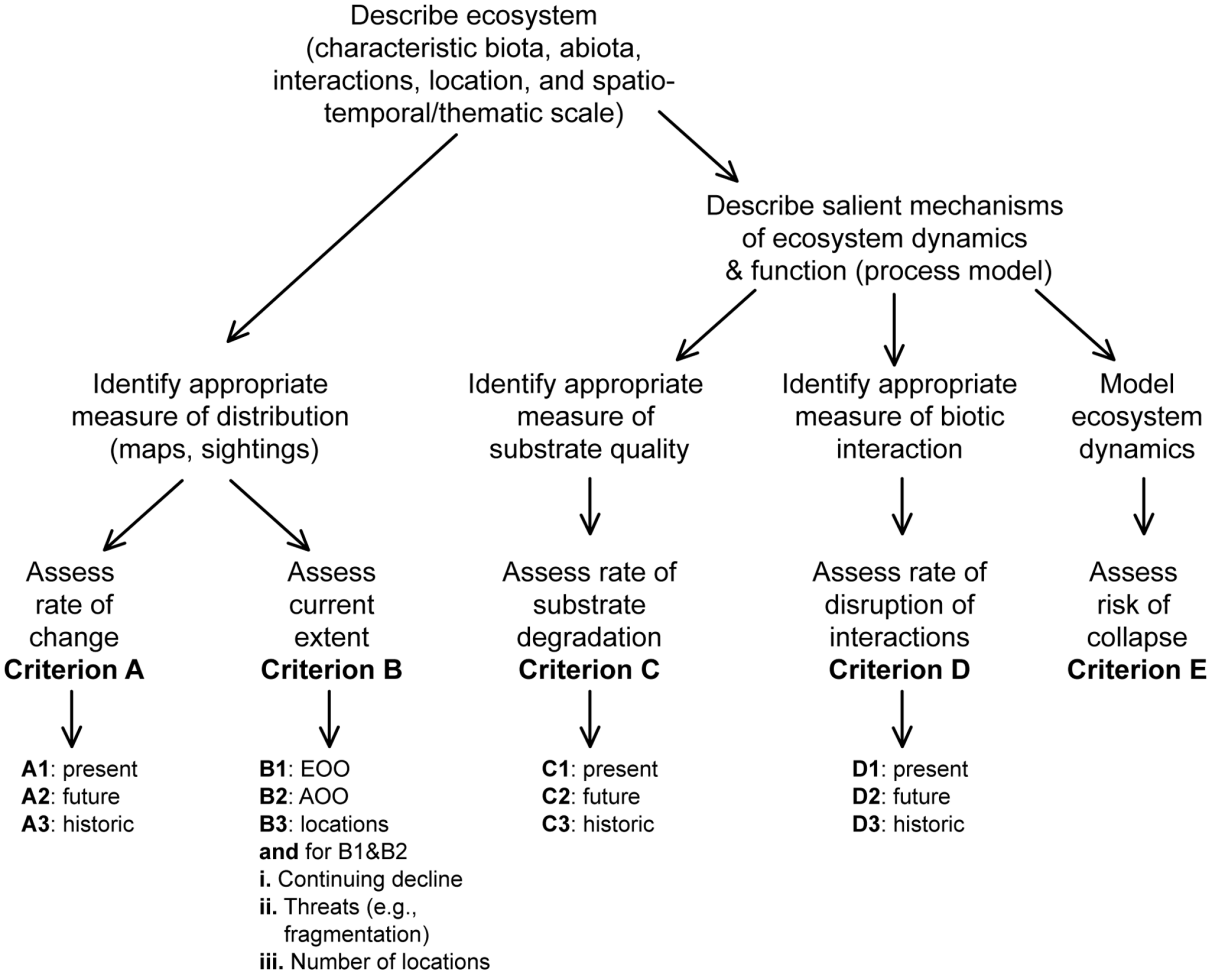
Protocol for assessing the risk of collapse of an ecosystem using proposed Red List criteria v2.0 (see **Table 3** **)** .

The risk assessment protocol comprises five rule-based criteria based on thresholds for distributional and functional symptoms represented in the risk model (Fig. 2, Table 3). Symptoms may be measured by one or more proxy variables. These may be generic or specific to particular ecosystems (see text on respective criteria for guidance on variable selection). The criteria and thresholds assign each ecosystem to one of three ordinal categories of risk (Table 3, Fig. 3), or else one of several qualitative categories.

An ecosystem under assessment should be evaluated using all criteria for which data are available. Overall threat status is the highest level of risk returned by any of the criteria (Fig. 3), since risk is determined by the most limiting factor [25]. The quantitative categories of risk [12] mirror those of the IUCN Red List of Threatened Species (IUCN 2001): Critically Endangered (CR); Endangered (EN); and Vulnerable (VU). These are complemented by several qualitative categories that accommodate 1) ecosystems that just fail to meet the quantitative criteria for the three threatened categories (NT, Near Threatened); 2) ecosystems that unambiguously meet none of the quantitative criteria (LC, Least Concern); 3) ecosystems for which too few data exist to apply any criterion (DD, Data Deficient); and 4) ecosystems that have not yet been assessed (NE, Not Evaluated). An additional category (CO, Collapsed) is assigned to ecosystems that have collapsed throughout their distribution, the analogue of the extinct (EX) category for species [6].

#### Time scales

**Figure 4:**
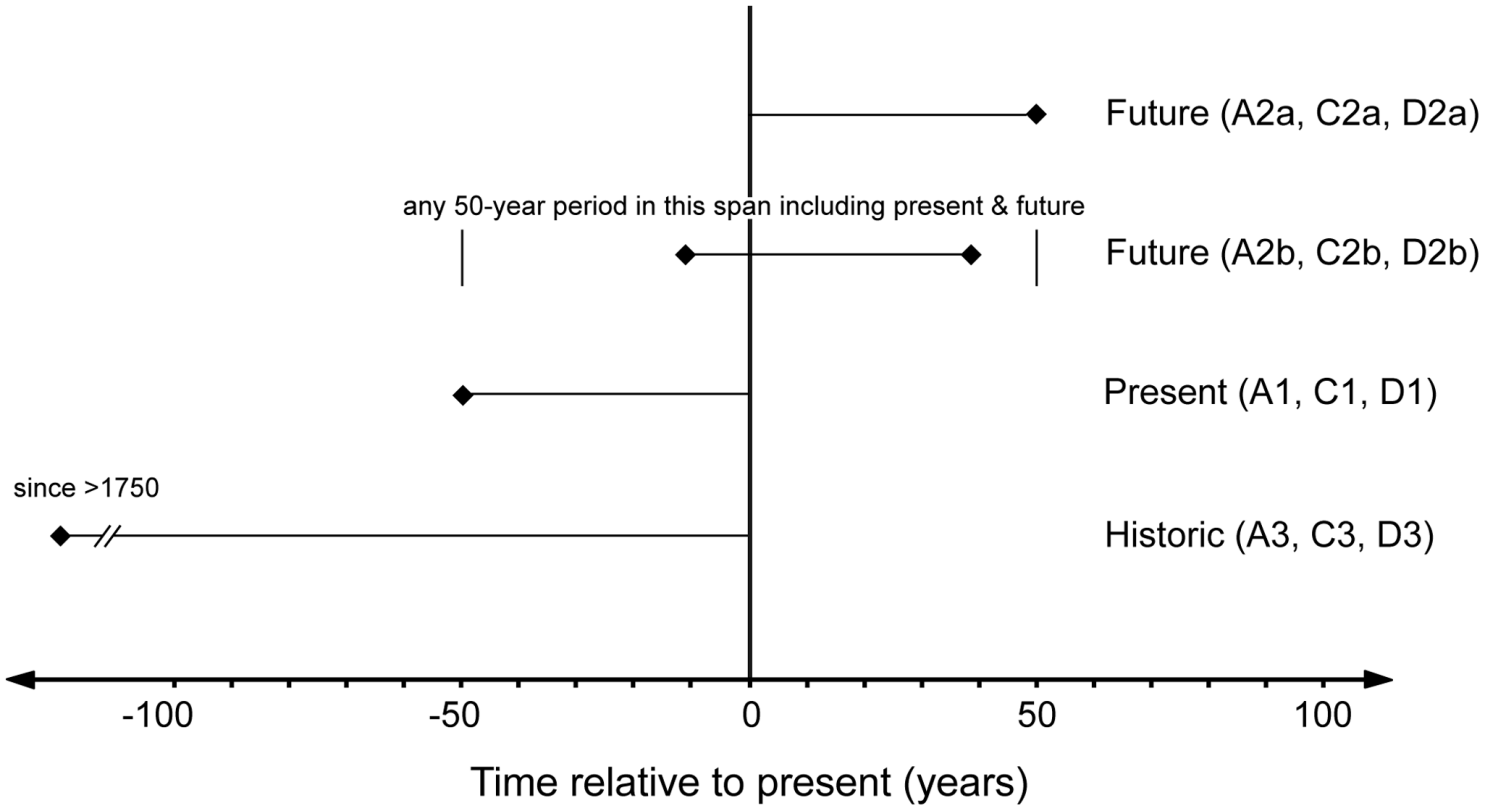
Time scales for assessment of change under criteria A, C and D.

The criteria assess declines over three time frames: current, future, and historic (Fig. 4). Current declines are assessed over the past 50 years: recent enough to capture current trends, but long enough to reliably diagnose directional change, distinguish it from natural fluctuations in most instances and to plan management responses. Causes of decline are often uncertain but, taking a precautionary approach, the protocol assumes that current declines indicate future risks irrespective of cause.

Assessment of future declines requires predictions about changes over the next 50 years or any 50-year period including the present and future (Fig. 4). Past declines may provide a basis for such predictions, but future declines may be predicted even when the ecosystem is currently stable. Such predictions require a defensible assumption about the pattern of future change (i.e. accelerating, constant, decelerating). Plausible alternative models of change should be explored [79], but a constant proportional rate of decline is often a reasonable default assumption for a range of ecosystems (e.g. [80]).

Assessments of historical declines are essential for ecosystems containing biota with long generation lengths and slow population turnover [25]. Even where future rates of decline abate, historical reductions in distribution or function may predispose an ecosystem to additional threats [81], [82], and reduce its ability to absorb adverse changes [68]. Historic declines are assessed relative to ecosystem status at a notional reference date of 1750 (Fig. 4), corresponding approximately with the earliest onset of industrial-scale exploitation of ecosystems, although the actual onset varies worldwide. Some anthropogenic changes occurred prior to 1750 [83], but knowledge of earlier distributions, trends and their causes is limited. Distribution models with environmental predictors may be used to estimate historic declines based on the difference between the current state of an ecosystem and its expected state in the absence of anthropogenic effects.

#### Decline thresholds

The ordinal categories of risk are delimited by different thresholds of decline. Our rationale for setting these thresholds is partly grounded in theory and partly pragmatic, recognizing that: i) theory provides a qualitative basis for ordered thresholds for decline, but offers limited guidance for setting their absolute values; and ii) our aim is to rank ecosystems into informative ordinal categories of risk, rather than estimate precise probabilities of collapse.

Species-area relationships [84] provide theoretical guidance for estimating loss of biota with declining area of available habitat. However, generic use of species-area relationships across many ecosystems and large scales is problematic for several reasons. Firstly, species loss cannot simply be calculated by reversing species accumulation curves [85]: the area in which the last individual of a species disappears (extinction) is always larger than the sample area needed to detect the first individual of a species. Secondly, the slope (z), of the species-area relationship varies empirically from 0.1 to 0.25, depending on the taxonomic groups assessed [84], habitat quality [86], habitat heterogeneity [87], mainland-island context [84] and time lags in reaching equilibrium [82], [88]. A third problem is that application of species-area relationships to landscapes and seascapes does not account for the patchiness of species occurrence within ecosystem types [89]. Moreover, some relationships exhibit context-dependent threshold behaviour that differs between taxonomic groups and landscape types [90], [91]. Fourthly, species-area relationships predict only species richness, not their abundance, which may affect ecosystem functions [53]. Species-area models are therefore unlikely to support universal threshold values of decline for assessing ecosystem status.

**Figure 5:**
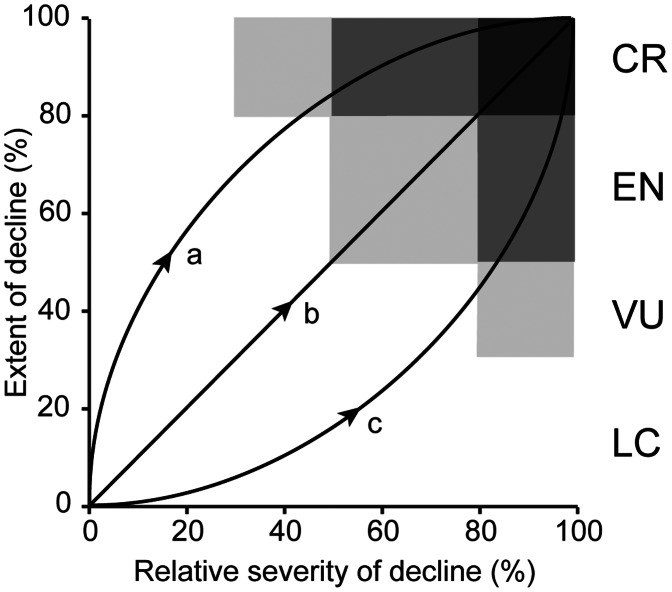
Contrasting pathways of environmental or biotic degradation and their corresponding risk classifications under criteria C and D. (a) initially widespread and benign degradation, later increasing in severity. (b) severity and extent of degradation increase at similar rates. (c) localised but severe degradation, later becoming more widespread. Ecosystems that just fail to meet the thresholds for Vulnerable status (e.g. extremely severe (>80%) decline in environmental quality over 20–30% of distribution, or severe (>30%) decline over 70–80% of distribution) may be assigned Near Threatened (NT) status.

It is noteworthy that the relationship between biodiversity and ecosystem function, when averaged over many cases, has a similar monotonic form to species-area relationships and also varies in slope [31]. Thus, in the absence of a clear theoretical foundation for setting particular thresholds for criteria involving declines in area or function (A, C, and D), we set threshold values at relatively even intervals for current and future declines (Vulnerable 30%, Endangered 50%, Critically Endangered 80%). The spread of thresholds between zero and 100% seeks to achieve an informative, rather than highly skewed ranking of ecosystems among the categories, while the lowest threshold of 30% recognises that an evidence of an appreciable decline in ecosystem distribution or function is necessary to support listing in a threatened category. These base thresholds are consistent with thresholds for population reduction in species Red List criteria (IUCN 2001). We set higher thresholds for historic declines (50%, 70%, 90%) because times frames are longer. Declines within 5–10% of VU thresholds may warrant listing as NT (Fig. 5), although we propose no quantitative thresholds for this category. Below, we explore the sensitivity of risk assessment outcomes to variation in these thresholds.

#### Collapse thresholds

Each of the five criteria implies a threshold of collapse (Fig. 1). For criteria based on spatial extent (A and B), ecosystems may be generally assumed to have collapsed if their distribution declines to zero (Fig. 1a–b) - when the ecosystem has undergone transformation throughout its entire range. However, use of the zero threshold will depend on the variables and maps used to represent the ecosystem distribution, and some ecosystems may collapse before their mapped distribution declines to zero (e.g. Table 2).

For criteria based on functional variables (C and D), a range of values will typically define collapse for a given variable (Fig. 1c–e). This range should be bounded between the minimum possible value, where there is no doubt that the ecosystem has collapsed, and a plausible maximum value based on observations of localised cases where the ecosystem appears to have moved beyond its natural range of variation (defined in the description of its characteristic native biota and processes), and as a result has lost characteristic native biota (see Appendix S2 for examples). A similar approach can be applied when simulation models are used to estimate the risk of collapse under criterion E. The collapsed state(s) should be identified among those represented in the model and bounded thresholds of relative abundance and/or persistence should be specified to identify the bounds of natural variation in the system.

## The Risk Assessment Criteria

The five risk assessment criteria are summarised in Table 3 and Appendix S1 contains a glossary of terms applied in the criteria and supporting concepts. Below we discuss the theoretical rationale that underpins each one and offer guidance for choosing and estimating the variables required to assess them.

### Criterion A. Decline in Distribution

#### Theory

Declining distribution is an almost universal element of existing ecosystem risk assessment protocols [13] and is analogous to Caughley’s declining population paradigm [92], as both represent diminishing abundance of biota. The diversity of species persisting within an ecosystem is positively related to the area or volume of substrate available [93]. Conversely, as ecosystem area declines, so do carrying capacities for component species, niche diversity and opportunities for spatial partitioning of resources and avoidance of competitors, predators and pathogens [87], [94], [95]. These area-related changes will increase extinction risks for component species and reduce an ecosystem’s ability to sustain its characteristic biota (Fig. 2). As ecosystem area declines, the resulting loss of biota depends on its spatial pattern in relation to threats and conservation measures [96], [97]. Although sampling effects preclude reversal of the quantitative species-area model [85], the qualitative relationship holds even for species that only lose unoccupied habitat, because such losses diminish opportunities for colonisation and rescue to compensate stochastic extirpations and declines [98].

#### Estimation

Rates of decline in ecosystem distribution will typically be estimated from time series of maps (e.g. [80]), field observations [65] or range maps constructed from point locations (e.g. [99]). Potential spatial proxies for ecosystem distributions include field observations of organism assemblages, climate, substrate, topography, bathymetry, ocean currents, flood regimes, aquifers or some synthesis of these that can be justified as valid representations of the distribution of ecosystem biota or its niche space. Vegetation mapping [100] and remote sensing [23] provide useful proxies for terrestrial, freshwater and benthic marine ecosystems [101]. The case studies (Appendix S2) provide a diversity of examples of such maps. For marine ecosystems, maps of physical factors such as sea floor characteristics, ocean currents, water temperatures and water chemistry may also be appropriate [49], [102], [103]. In some subterranean, freshwater and marine ecosystems, trends in the depth dimension may be appropriate proxies of declines in distribution (e.g. Table 2), so long as they reflect trends in carrying capacity and niche diversity for characteristic biota.

Current reductions in distribution may be calculated directly if data are available for 50 years ago and the present, or through an annual rate as a basis for cautious extrapolation. Spatial models [104] may be used for projecting expected distributions into the recent past (criterion A1, Table 3), future (criterion A2) or to estimate historic anthropogenic change (criterion A3) [105].

### Criterion B. Restricted Distribution

#### Theory

Many processes that threaten ecosystems are spatially autocorrelated (clustered). Examples include catastrophes or disturbance events [106], [107], localised invasions of alien species [108] and regional climate changes [74], [109], [110]. Risks posed by such processes are spread across multiple independent patches in widely distributed ecosystems, but not in ecosystems with geographically restricted distributions [13]. The primary role of criterion B is to identify ecosystems whose distribution is so restricted that they are at risk of collapse from the concurrence of threatening events or processes [13], [79]. It also serves as an assessment of occupied habitat for component biota which, through carrying capacity, is positively related to population viability irrespective of exposure to catastrophic events [64]. These concepts are analogous to Caughley’s (1994) small population paradigm [25], [92], and are incorporated into most existing risk assessment protocols [13].

#### Estimation

Two metrics, Extent of Occurrence (EOO) and Area of Occupancy (AOO), represent conceptually different aspects of species range size [111] and are also relevant to ecosystems (Table 3). EOO (criterion B1) measures the ability to spread risks over a contiguous area that encloses all occurrences using a minimum convex polygon, whereas AOO (criterion B2) measures the ability to spread risks among occupied patches with a count of occupied grid cells [53], [79], [112]. The same measurement protocols are appropriate to entities with depth dimensions or linear patterns of distribution [25]. In some cases, spatial data may be insufficient to estimate EOO or AOO, but there is evidence that a small number of plausible threatening events may cause an ecosystem to become Critically Endangered within the near future. Such ecosystems may be listed as Vulnerable under criterion B3 if they occupy few ‘locations’ relative to the extent of threatening events (Appendix S1).

Estimates of AOO are highly sensitive to both spatial and thematic grain [13], [79], [113]. Ecosystems may be classified so broadly or mapped so coarsely that they never meet thresholds for threatened categories or, conversely, so narrowly or finely that they always qualify for threatened status [13]. To reduce bias, all estimates of AOO for Red List assessment must be standardized to the same spatial grain. We recommend 10×10 km grid cells for estimating ecosystem AOOs (in contrast to the 2×2 km grids recommended for species assessments; [79]), first because ecosystem boundaries are inherently vague (*sensu* [66]), so it is easier to determine that an ecosystem occurrence falls within a larger grid cell than a smaller one. Second, larger cells may be required to diagnose the presence of ecosystems characterized by processes that operate over large spatial scales, or diagnostic features that are sparse, cryptic, clustered or mobile (e.g. pelagic or artesian systems). Last, larger cells allow AOO estimation even when high resolution data are limited. These considerations therefore suggest that a larger cell size is appropriate for ecosystems than recommended for species [79]. A potential limitation of AOO estimates based on large grain sizes is that they may be inflated for ecosystems with many small, dispersed patches (e.g. forest fragments, small wetland patches), yet such occurrences may not substantially offset risks. To reduce this effect, we recommend that cells are counted as occupied only if the ecosystem covers more than 1 km^2^ (1%) of cell area.

#### Thresholds and subcriteria

Critically Endangered, Endangered and Vulnerable ecosystems are delineated by AOO thresholds of two, 20 and 50 grid cells, respectively (Table 3). EOO thresholds were an order of magnitude larger (Table 3) because, like species, ecosystems generally extend across larger areas than they actually occupy [6]. We recognise that such thresholds are somewhat arbitrary and below, we explore the sensitivity of risk assessment outcomes to variation in the thresholds. However, the proposed thresholds are based on our collective experience on the extent of wildland fires, extreme weather events, chemical spills, disease epidemics, land conversion and other spatially explicit threats. Studies on the risks posed by spatial processes of varying extent are needed across a variety of ecosystems to inform the adequacy of these values.

To be eligible for listing in a threat category under criterion B, an ecosystem must also meet at least one of three subcriteria that address various forms of decline. These subcriteria distinguish restricted ecosystems at appreciable risk of collapse from those that persist over long time scales within small stable ranges [114], [115]. Only qualitative evidence of decline is required to invoke the subcriteria, but declines must i) reduce the ability of an ecosystem to sustain its characteristic native biota; ii) be non-trivial in magnitude; and iii) be likely to continue into the future (Appendix S1). These declines may be in ecosystem distribution or processes (abiotic or biotic). Evidence of past declines is not essential, but future declines may be inferred from serious and imminent threats or occurrence at few locations, indicating limited capacity to spread risks [79].

### Criterion C: Environmental Degradation

#### Theory

Environmental (abiotic) degradation may diminish the ability of an ecosystem to sustain its characteristic native biota by changing the variety and quality of environmental niche space available to individual species. This interpretation relies on measurement of abiotic variables and excludes biotic mechanisms of degradation. Most existing protocols conflate the assessment of biotic and abiotic declines in ecosystem function [13]. In contrast, our risk assessment model defines separate assessment pathways (criteria C and D, Fig. 2) because the threats, their causes, effects and mechanisms of functional decline differ fundamentally between biotic and abiotic degradation, and hence so do the variables needed to assess them.

**Figure 6:**
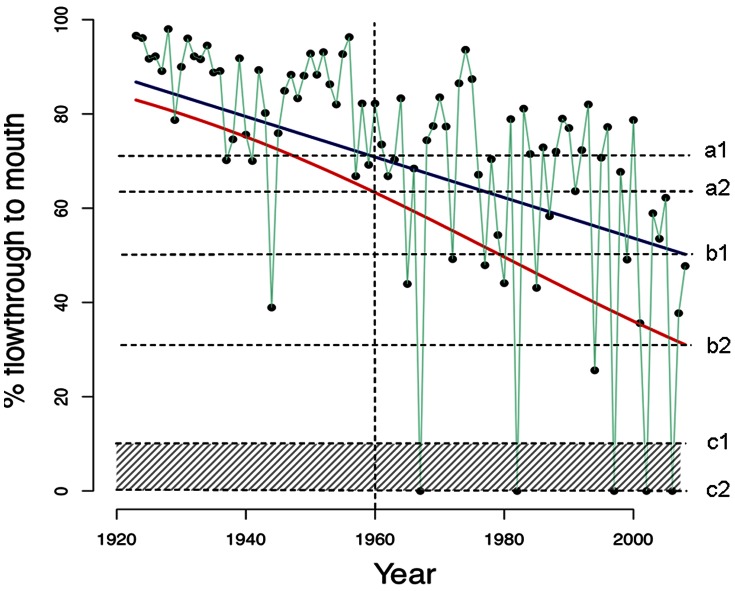
Estimation of relative severity of environmental degradation (criterion C) or disruption of biotic interactions (criterion D). Example using stream flowthrough data as percent of mean unregulated flows (aqua line joining filled circles) for the Murray River adapted from [57], see Appendix S2.8. There is uncertainty in both the rate of decline in flowthrough (two alternative regression lines) and the level of flowthrough at which the water-dependent ecosystem would collapse (shaded area). The threshold of collapse is the level of stream flowthrough that would result in widespread tree death and replacement of forest vegetation (most likely by shrubland). This was estimated to occur when mean flowthrough (as estimated by long-term regression) falls to 0–10% of unregulated flow levels (shown as a bounded estimate c1–c2, dashed lines), as widespread tree dieback began to occur when flowthrough was zero in several year of the past decade (see Appendix S2.8 for process model and justification). Based on a best-fit Gaussian regression model of the flowthrough data (dark blue line), the mean flowthrough fell from 71% in 1960 (dotted line a1) to 50% in 2009 (dotted line b1). A beta regression model (red line) gave an improved fit to the data and indicates a decline in mean flowthrough from 63% in 1960 (a2) to 31% in 2009 (b2). A standardised estimate of the relative severity of hydrological degradation over the past 50 years = 100×(b-a)/(c-a). The minimum plausible estimate = 100×(b1–a1)/(c1–a2) = 100×(71–50)/(71–0) = 30% and the maximum plausible estimate = 100×(b2–a2)/(c2–a1) = 100×(63–31)/(63–10) = 60%. Based on uncertainty in the flowthrough regression models and collapse threshold, a bounded estimate of hydrological degradation in this ecosystem is therefore 30–60% over the past 50 years.

A reformulation of the species-area relationship [86] provides a theoretical basis for degradation criteria by incorporating the influence of habitat quality on the number of species able to persist in a given area. This model predicts bird species richness by including a habitat complexity score relative to an optimal value. We generalise this to an index of ‘relative severity’ of degradation, representing the ratio of observed change in environmental suitability (for ecosystem biota) over a given time to the amount of change that would cause an ecosystem to collapse (Fig. 6). Theoretically, suitability is aggregated across all characteristic biota, but in practice may be estimated from key environmental variables that regulate ecosystem behaviour (e.g. river flows for riparian wetlands, see examples in Appendix S2).

Criterion C (Table 3) is structured to account for ecosystems undergoing environmental degradation with contrasting scenarios of severity and extent (Fig. 5). Thus, ecosystems are only eligible for listing as Critically Endangered if environmental change that threatens the persistence of their characteristic biota is both extremely severe (≥80% relative severity) and extremely extensive (≥80% of the distribution). In contrast, those undergoing extremely severe but localised degradation or less severe degradation over very extensive areas may be eligible for listing in lower threat categories (Fig. 5).

#### Estimation

We suggest four requirements to assess risks posed to ecosystems by environmental degradation. First, there must be plausible evidence of a causal relationship between a process of environmental change and loss of characteristic native biota (Fig. 2). For example, an assessment of wetland degradation based on change in water quality would require evidence that decline in water quality was associated with loss of wetland biota, at least in comparable ecosystem types. Development of simple diagrammatic process models can help to make explicit the diagnosis of salient processes that influence transitions between functional and degraded ecosystem states, as well as the characteristics that differentiate the states [54], [56]. Hence, these models serve the minimum requirements for inferring appropriate measures of environmental degradation for risk assessment (see examples in Appendix S2).

Second, assessing abiotic degradation requires suitable spatial and scalar variables for estimating the extent and severity of degradation. The characteristics of the ecosystem, environmental dependencies of biota and agents of degradation will determine which variables are relevant. The most suitable will be those with the most proximal cause-effect relationships and the greatest sensitivity to loss of biota. Approaches that apply generic indices across functionally contrasting ecosystems are unlikely to assess degradation accurately because salient processes may differ between ecosystems. Furthermore, aggregation of multiple variables could confound different mechanisms and directions of environmental change, making the index less sensitive to degradation than individual variables. Table 4 lists examples of potentially suitable abiotic variables for different ecosystems, while Appendix S2 provides more detailed justifications of variable selection for specific ecosystem types. For some ecosystems, it is noteworthy that measures of environmental heterogeneity may be more appropriate than absolute measures, because declines in the number of limiting resources (niche dimension) reduce species diversity in a range of terrestrial, freshwater and marine ecosystems [95].

**Table 4:** Examples of variables potentially suitable for assessing the severity of environmental degradation under criterion C.

Degradation process	Example variables	Sources
Desertification of rangelands	Proportional cover of bare ground, soil density, soil compaction indices, remote sensing landcover indices	[159], [160]
Eutrophication of soils, freshwaterstreams or lakes	Levels of dissolved or soil nitrogen, phosphorus, cations, oxygen, turbidity, bioassay	[15]
De-humidification of cloud forests	Cloud cover, cloud altitude	[161]
Deforestation by acid rain	Rain water chemistry	[62]
Homogenisation of microhabitats	Diversity of micro-terrain features, spatial variance in inundation depth and duration	[162]
Changed water regime or hydroperiod	Field-based monitoring of stream flow volume, or piezometric water table depth; remotesensing of spatial extent of surface water, frequency and depth of inundation	[57]
Salinisation of soils or wetlands	Field monitoring of salinity of soils or groundwater, remote sensing of ground surface albido	[163]
Sedimentation of streams, coral reefs	Sediment accumulation rates, sediment load of streams, discharge, turbidity of water column, frequency and intensity of sediment plume spectral signatures	[164]
Structural simplification of benthic marine ecosystems (e.g. by bottom trawling)	Microrelief, abundance of benthic debris, trawling frequency and spatial pattern	[165]
Sea level rise	Acoustic monitoring of sea level, extent of tidal inundation	[166]
Retreat of ice masses	Remote sensing of sea ice extent	[167]

Third, assessing environmental degradation requires calculation methods to compare observed or projected changes against the criteria. Assessors may either estimate the extent of degradation (as % of ecosystem distribution) that exceeds a threshold level of severity (Fig. 5) or estimate the average severity of degradation across the entire ecosystem distribution (100% of extent). ‘Relative severity’ measures the proportional progress of an ecosystem on a trajectory to collapse over the time frame of assessment, and is essential for comparing risks across ecosystems undergoing different types of degradation. It can be calculated by range-standardising the raw values of the degradation variable between its initial value and its collapse threshold (Fig. 6). This requires an assumption about the level of degradation that corresponds with collapse (Table 2), and a functional form for interpolation (e.g. linear). Comparisons with reference sites may justify these assumptions [116].

Finally, estimating, inferring or projecting the severity and extent of degradation over specific time frames may require extrapolation of trends from available time series. This requires assumptions about whether degradation is constant, accelerating, or decelerating (see criterion A), based on an understanding of the mechanism of decline and its historical and spatial context. Assessors also need to evaluate whether the available data are sufficiently representative of prevailing conditions to permit extrapolation, preferably with statistical inference (but subjective reasoning may play a greater role when sample sizes are too small). Where time series data are unavailable, it may be possible to infer changes in degradation using space-for-time substitution sampling with appropriate reference sites [117], [118].

### Criterion D: Disruption of Biotic Processes and Interactions

#### Theory

The persistence of biota within ecosystems depends on biotic processes and interactions (Fig. 2), including competitive, predatory, facilitatory, mutualistic, trophic and pathogenic processes, as well as interactions between organisms and their physical environment, habitat fragmentation, mobile links (e.g. seasonal migration), species invasions and direct exploitation by humans. There is a growing body of theory and empirical evidence that biodiversity loss reduces the capacity of ecosystems to capture resources, produce biomass, decompose organic matter and recycle carbon, water and nutrients, and also that biodiversity loss reduces the stability of these functions through time [30]. Both the identity and diversity of organisms within a system control its functioning, firstly because key taxa make disproportionate contributions to particular functions, and secondly because niche partitioning and positive species interactions promote complementary contributions to function from individual species [30].

Feedback interactions underpin self-organisation and are crucial to ecosystem resilience, the ability to absorb environmental change while maintaining structure, characteristic biota and processes [119]. Conversely, significant disruptions to biotic processes and interactions can cause collapse, regime shift and re-organisation into a new entity that is unable to sustain the biota of the original system [35], [74], [120], [121]. Diamond [122] identified trophic cascades caused by disruption to interactions as one of five major threats to biodiversity. Subsequent work has sought to identify factors that promote this mechanism of ecosystem collapse [123], [124], although non-trophic interactions also play important roles [125], [126].

Certain types of ecosystems may be especially sensitive to disruption of biotic processes and interactions. These include systems with strong top-down trophic regulation [58], [124], [127], [128], systems with many mutualistic or facilitation interactions [126], [129], systems that are strongly dependent on mobile links [130] and systems where disturbance regimes impose top-down regulation and positive feedbacks operate between the biota and the disturbance [131], [132].

#### Estimation

Assessment of criterion D must address the same four requirements as criterion C: i) plausible evidence of the causes or mechanisms of functional decline; ii) selection of appropriate biotic variables for assessing declines; iii) range standardisation to estimate relative severity; and iv) calculations and justifiable assumptions to estimate declines over relevant time frames. Process models again provide a useful framework for interpretation and explicit justification of analytical choices. A broad set of variables are potentially useful for assessing biotic processes and associated functional declines (Table 5). We briefly review some strengths and weaknesses of alternatives below and present detailed examples of assessment in Appendix S2.

**Table 5:** Examples of biotic variables potentially suitable for assessing the severity of disruption to biotic interactions under criterion D.

Variable	Role in ecosystem resilience and function	Example
Species richness (number ofspecies within a taxonomic groupper unit area)	Ecological processes decline at an accelerating rate withloss of species [168]. Species richness is relatedindirectly to ecosystem function and resiliencethrough its correlations with functionaldiversity, redundancy and complementarity(see below)	Response of graminoid diversity and relative abundance to varying levels of grazing in grassland [135].
Species composition and dominance	Shifts in dominance and community structureare symptoms of change in ecosystembehaviour and identity	Shift in diet of top predators (killer whales) due to overfishing effects on seals, caused decline of sea otters reduced predation of kelp-feeding urchins, causing their populations to explode with consequent collapse of giant kelp, structural dominants of the benthos [58]. See Appendix S2.
Abundance of key species (ecosystem engineers, keystone predators and herbivores, dominant competitors,structural dominants, transformerinvasive species)	Invasions of certain alien species may alter ecosystembehaviour and identity, and make habitat unsuitablefor persistence of some native biota. Transformeralien species are distinguished from benigninvasions that do not greatly influenceecosystem function and dynamics	Invasion of crazy ants simplifies forest structure, reduces faunal diversity and native ecosystem engineers [108]. Invasion of arid Australian shrublands and grasslands by Buffel Grass makes them more fire prone and less favourable for persistence of native plant species [169], [170].
Functional diversity (number and evenness of types)	High diversity of species functional types (e.g. resourceuse types, disturbance response types) promotesco-existence through resource partitioning, nichediversification and mutualisms [71]. Mechanismssimilar to functional complementarity(see below).	High diversity of plant-derived resources sustains composition, diversity and function of soil biota [171], Fire regimes promote coexistence of multiple plant functional types [134]. Appendix S2.
Functional redundancy (number oftaxa per type; within- and cross-scaleredundancy; see (Allen et al. 2005)	Functionally equivalent minor species may substitutefor loss or decline of dominants if many species performsimilar functional roles (functional redundancy).Low species richness may be associated with lowresilience and high risks to ecosystem function underenvironmental change [71], [135].	Response of bird communities to varying levels of land use intensity [138].
Functional complementarity (dissimilarity between types or species)	Functional complementarity between species (e.g. inresource use, body size, stature, trophic status,phenology) enhances coexistence through nichepartitioning and maintenance of ecosystemprocesses [172]	High functional complementarity within both plant and pollinator assemblages promotes recruitment of more diverse plant communities [125].
Interaction diversity (interaction frequencies and dominance, properties of network matrices)	Interactions shape the organisation of ecosystems,mediate evolution and persistence of participating speciesand influence ecosystem-level functions,e.g. productivity [173]	Overgrazing reduced diversity of pollination interactions [129].
Trophic diversity (number of trophic levels, interactions within levels, food web structure)	Compensatory effects of predation andresource competition maintain coexistence of inferior competitorsand prey. Loss or reduction of some interactions(e.g. by overexploitation of top predators) mayprecipitate trophic cascades via competitiveelimination or overabundance ofgeneralist predators	Diverse carnivore assemblages (i.e. varied behaviour traits and densities) promote coexistence of plant species [142], decline of primary prey precipitates diet shifts and phase shifts [174].
Spatial flux of organisms (rate, timing, frequency and duration of species movements between ecosystems)	Spatial exchanges among local systems in heterogeneous landscapes provide spatial insurance for ecosystem function [143]. Exchanges may involve resources, genes orinvolvement in processes [130]	Herbivorous fish and invertebrates migrate into reefs from seagrass beds and mangroves, reducing algal abundance on reefs and maintaining suitable substrates for larval establishment of corals after disturbance [175].
Structural complexity (e.g.complexityindices, number and cover of verticalstrata in forests, reefs, remotesensing indices)	Simplified architecture reduces niche diversity, providingsuitable habitats for fewer species, greater exposureto predators or greater competition for resources(due to reduced partitioning)	Structurally complex coral reefs support greater fish diversity [176], structurally complex woodlands support greater bird diversity [86].

Species loss reduces ecosystem function and resilience to ecosystem collapse and reduces the possible range of alternative ecological organizations [31], [120]. Species richness is the simplest and most generic measure of this process (Table 5), but its sensitivity may be limited if declines in some species are lagged or offset by increases in others that do not perform similar functions [16]. Also, the functional consequences of species loss may not be apparent. Ecosystem collapse often involves changes in species composition and dominance [74]. These variables avoid some pitfalls of species richness, although it may be difficult to discriminate functional decline from natural variability in composition and dominance.

Problems with generic measures may be mitigated by variables that are more proximal to biotic mechanisms that maintain ecosystem resilience and characteristic biota [133]. Partitioning component species into functional types or guilds [134] allows more direct analysis of declines in function and resilience through trends in functional diversity, redundancy and complementarity [33], [64], [135], [136], [137], [138]. The abundance, biomass or dominance of key native or alien species may be useful measures of functional decline (Table 5), so long as there is plausible evidence of their functional roles and their influence on the persistence of characteristic native biota. Declines in large herbivores and large predators, for example, may drastically affect the dynamics and functioning of ecosystems with top-down regulation [124], [128], [139]. Invasion of alien species may transform ecosystems through interactions as competitors, predators, pathogens or ecosystem engineers [108], [140].

Measures of interaction diversity, such as the structure and size of interaction networks, provide another perspective on functional decline (Table 5). Decoupling of interactions may reduce diversity by preventing some species from completing their life cycles [126], [129]. Trophic diversity (Table 5), a special case of interaction diversity where interactions are directional and hierarchical [141], can mediate co-existence, resilience and function in contrasting ecosystems [15], [58], [139], [142].

Spatial dynamics of biotic interactions influence ecosystem resilience and function through exchanges across heterogeneous landscapes and seascapes [130]. Movements of organisms involve transfer of nutrients and genes, and may initiate local reorganization through episodic predation and ecosystem engineering. These exchanges provide spatial insurance for sustaining ecosystem biota, both through spatial averaging and functional compensation [143], [144]. Measures of disruption to these processes include changes in identity and frequency of species movements, and measures of fragmentation (Table 5).

Finally, niche diversity in some ecosystems depends on structural complexity generated by components of the biota itself (Table 5). For example, vegetation structure is often used as a measure of habitat suitability for forest and woodland fauna [86], while reef rugosity is similarly used to evaluate habitat suitability for fish and some marine invertebrates [145]. As well as being salient representations of diversity in a range of ecosystems, data on structural complexity can relative inexpensive to obtain in the field, and some indices lend themselves to remote sensing.

### Criterion E. Quantitative Estimates of Risk of Ecosystem Collapse

#### Theory and estimation

A diverse range of simulation models of ecosystem dynamics allow the probability of ecosystem collapse to be estimated directly over the same 50-year future period as other criteria [59], [60], [136], [146], [147], [148], [149]. These models permit exploration of interactions and potential synergies between multiple mechanisms of collapse. This distinguishes direct risk estimation from the other criteria, each of which assess separate mechanisms through particular symptoms of risk (Fig. 2). Even where available data preclude construction of quantitative simulation models, criterion E provides a useful anchor for risk assessment and an overarching framework for other criteria, as its analogue does in Red List criteria for species [25]. Although development of simulation models was beyond the scope of this paper, we demonstrate criterion E with an existing model in a case study on the Coorong Lagoon in Appendix S2.

## Case Studies

### Sample Ecosystems

Twenty ecosystems were selected for assessment based on the authors’ areas of expertise, spanning five continents and three ocean basins (full details of assessments in Appendix S2). Although non-random, the selection encompassed terrestrial, subterranean, continental aquatic and marine aquatic environments in Europe, Africa, Asia, Australasia and the Americas and represented a wide range of thematic scales, threatening processes, data availability and levels of risk. Each ecosystem was assessed using the protocol in Fig. 3. The ecosystems assessed are summarised in Table 6.

**Table 6:** Summary of trial assessments for 17 ecosystems from freshwater (F), terrestrial (T), marine (M) and subterranean (S) environments.

	Localthreat status	IUCN status	# criteria assessed	# subcriteria assessed	# subcriteria supporting overall status	Spatial criteria assessed	Functional criteria assessed	Criteria determining overall status
1 Coastal sandstone upland swamps,Australia (F)	EN	EN-CR	4	9	2	+	+	A2,C2
2 Raised bogs, Germany	CR	CR	3	6	2	+	+	A3,C3
3 German tamarisk pioneer vegetation,Europe (F)	EN	EN	2	5	3	+		A1,A3, B2a,b
4 Swamps, marshes and lakes in theMurray-Darling Basin, Australia (F)	NE	EN-CR	4	10	2	+	+	D1,D3
5 Aral Sea, Uzebekistan and Kazakhstan (F)		CO	4	12	9	+	+	A1-3, C1-3, D1-3
6 Reedbeds, Europe (F)	LC	VU	4	8	3	+	+	A1,A3,D1
7 Gonakier forests of SenegalRiver floodplain (F)		CR	3	6	2	+	+	A1,A3
8 Floodplain Ecosystem of river red gumand black box, south-easternAustralia (F)	NE	VU	4	12	3	+	+	A2,C1,C2
9 Coolibah - Black Box woodland,Australia (F/T)	EN	EN	3	7	1	+	+	C1
10 Semi-evergreen vine thicket, Australia (T)	EN	EN	2	2	2	+		A3,B2a
11 Tepui shrubland, Venezuela (T)	LC	LC	3	8	8	+	+	A1-3,B1-3, D1-3
12 Granite gravel fields & sandplains,New Zealand (T)	LC	LC	4	11	11	+	+	A1-3,B1-3, C1-3,D1-3
13 Cape Sand Flats Fynbos, South Africa (T)	CR	CR	2	6	1	+		B1a,b
14 Tapia Forest, Madagascar (T)	NE	EN	2	4	1	+		A3
15 Great Lakes Alvar (T)		VU-EN	3	5	1	+	+	A3
16 Giant kelp forests, Alaska (M)	NE	EN-CR	2	4	2	+	+	D1,D3
17 Caribbean coral reefs (M)	NE	EN-CR	2	5	2	+	+	D1,D3
18 Seagrass meadows, South Australia (M)	NE	EN-CR	3	6	2	+	+	A1,C1
19 Coorong lagoons, Australia (F/M)	NE	CR	5	9	4	+	+	B1a,b,C2, D1,E
20 Karst rising springs, South Australia (C/F)	NE	CR	3	7	3	+	+	B1b,C1,C2

### Data Availability

**Figure 7:**
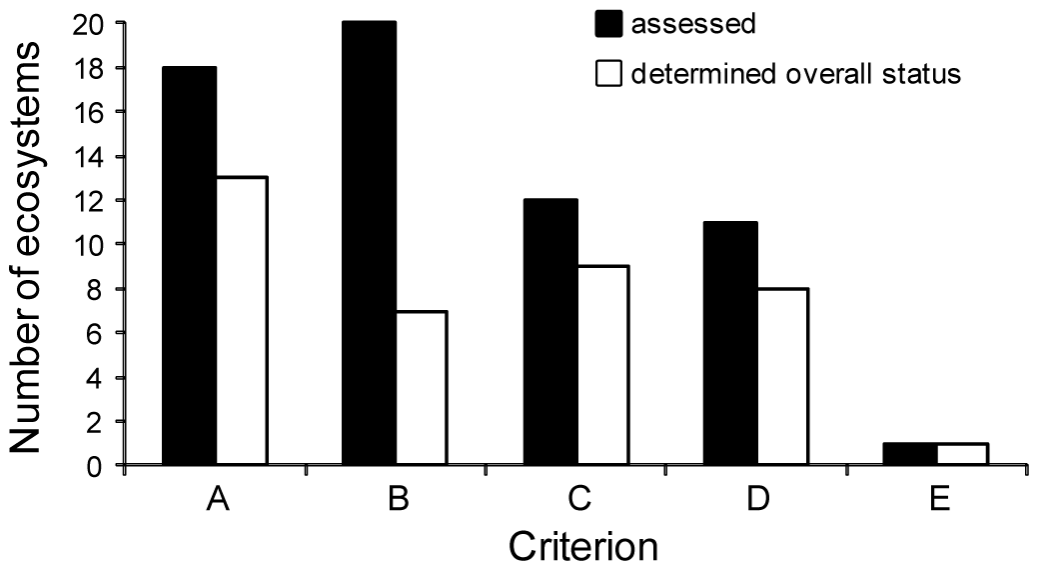
Number of ecosystems assessed for each criterion and number for which each criterion determined overall status.

Data were available to assess all five criteria in one ecosystem, four criteria in five ecosystems, three criteria in seven ecosystems and two criteria for the remainder (Table 6). Data were most commonly available to assess criterion B, followed by A, C and D, with only one ecosystem, the Coorong Lagoon, assessed for E (Fig. 7). The number of assessable subcriteria varied between ecosystems from two to 12, with at least seven of the 13 subcriteria assessed in half of the case studies (Table 6). All but four of the ecosystems (80%) had sufficient data to assess at least one distributional criterion (A or B) and one functional criterion (C or D).

The majority of terrestrial and freshwater case studies assessing criteria A and B used vegetation maps as spatial proxies to estimate ecosystem distributions, while some of the marine case studies used specialised map products derived from remote sensing. Estimates of current change in distribution were derived from time series of maps or imagery, almost all of which required reasoned assumptions to justify interpolation or extrapolation to the required 50-year time frame. Historical changes in distribution were most commonly inferred by comparing a contemporary map with a model of environmentally suitable areas which were assumed to be occupied by the ecosystem prior to human transformation of the landscape. This approach was less suitable for marine ecosystems, which were generally Data Deficient in criterion A3. In three ecosystems (Coastal upland swamps, River Red Gum forests, Cape fynbos), models of environmental suitability were used to project future changes in distribution, with outputs of alternative plausible models used to estimate uncertainty in the projections.

Eleven of the case studies used explicit process models to guide selection of functional variables for assessment of criteria C and D. Only one of these models was quantitative, permitting simulations to estimate risks of collapse under criterion E, although data appear sufficient to support construction of such models in at least two other case studies (1 and 8). A variety of abiotic proxy variables were used to assess environmental degradation, primarily in freshwater and marine ecosystems, including water flows and extraction rates, groundwater flows (subterranean/freshwater) nitrogen levels (both freshwater and marine ecosystems), climatic moisture, water volume, salinity, sea surface temperatures and ocean acidity. Proxy variables used to assess criterion D included the abundance of structurally important groups of species (resprouting shrubs, corals, kelp, seagrass), mobile links (birds), meso-predators (sea otters, fish), sensitive species (plankton), invasive species and threatened species. In a few cases, the available data were insufficient to make an assessment, but the identification of the proxy highlighted future needs.

### Assessment Outcomes

The outcomes of assessment varied from Least Concern to Collapsed (Table 6), with the overall status supported by multiple subcriteria for all but four of the ecosystems. In the four ecosystems for which overall status was supported by a single subcriterion, another subcriterion was assessed at the next lowest category of risk. Three ecosystems that were assessed as Least Concern or Collapsed were supported by 8–11 subcriteria. All of the criteria except E determined the overall status in multiple ecosystems, with criterion B yielding the highest threat in a lower proportion of ecosystems than A, C and D (Fig. 7). Nine of the ecosystem types selected for case studies had been assessed by government agencies or non-government organisations using local listing criteria. For eight of these nine case studies, the IUCN protocol produced the same threat status as those produced by local authorities. The status of the remaining ecosystem differed by only one category.

### Sensitivity Analysis of Thresholds

**Figure 8:**
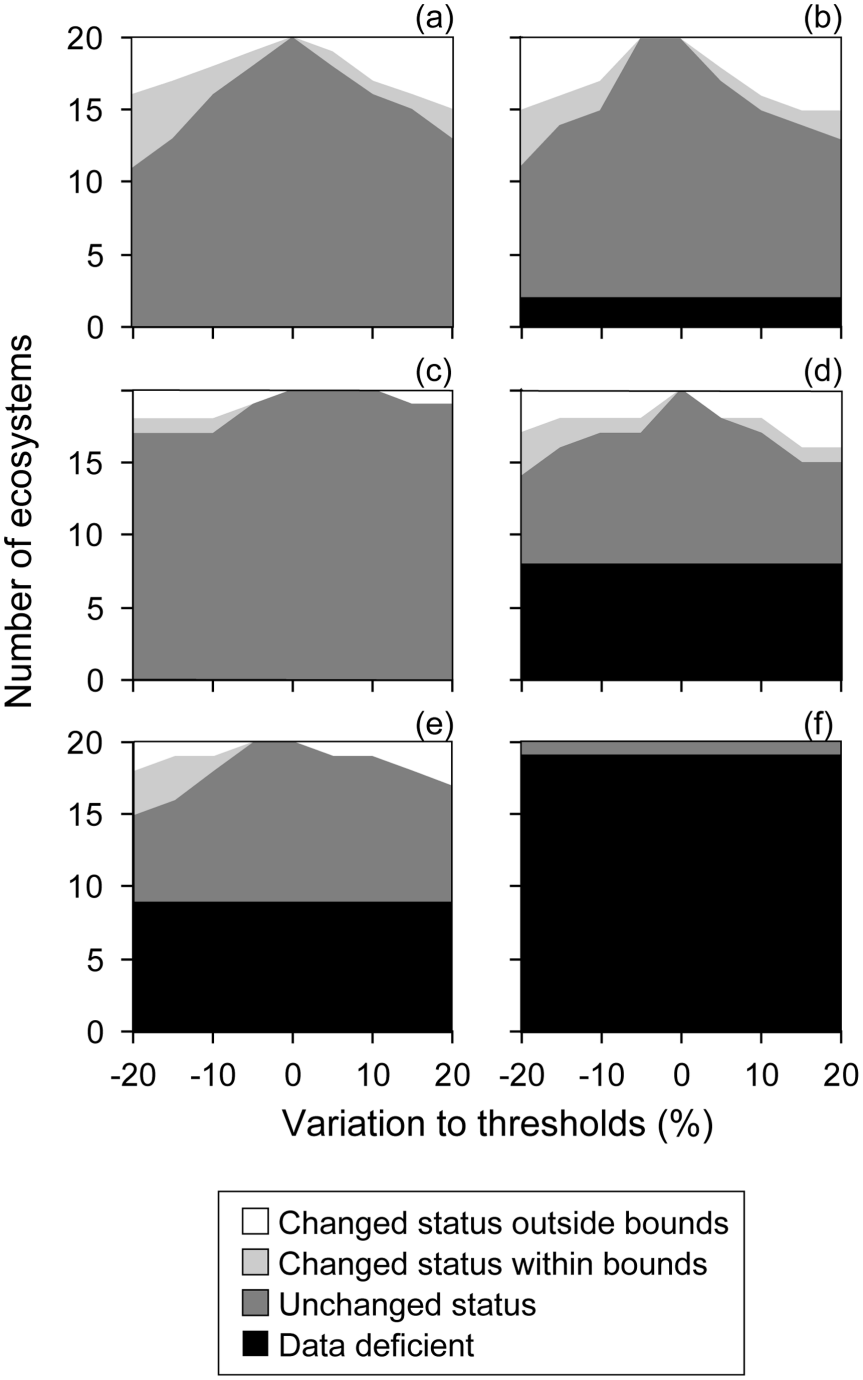
Sensitivity of risk assessment outcomes (relative to uncertainty bounds of the original assessment) to variation in threshold values for (a) all five criteria in combination; (b) criterion A only; (c) criterion B only; (d) criterion C only; (e) criterion D only; and (f) criterion E only.

A sensitivity analysis was carried out on the thresholds in all criteria using data from the 20 case studies. Thresholds were adjusted by ±5%, ±10%, ±15% and ±20% of current values i) for each individual subcriterion; ii) for all subcriteria in combination within each criterion; and iii) across all criteria in combination. This represents a plausible range of alternative thresholds, since larger adjustments would result in overlap between categories. Variation to thresholds by a given proportion across all criteria in combination resulted in a change in status for a slightly larger proportion of ecosystems (Figure 8a). For half of the ecosystems that changed status, however, the changes were within the bounds of uncertainty for the original assessment. The proportion of ecosystems that changed status outside the bounds of uncertainty were approximately commensurate with the proportional adjustment to thresholds. For example a 5% change in thresholds produced a change in status in approximately 5% of ecosystems, while a 20% change in thresholds produced a change in status for approximately 20–25% of ecosystems, depending on whether thresholds were increased or decreased. Although the sample size is limited, the results suggest moderate sensitivity of overall risk assessment outcomes to the thresholds, particularly as the case studies used for this analysis cover a wide variety of ecosystem types and data availability.

Individually, criteria A, C and D displayed similar levels of sensitivity to variation in their threshold values (allowing for different levels of data availability), and this was similar to the sensitivity of the overall risk status when all five criteria were combined (Figs. 8b, 8d, 8e cf. 8a). Criterion B was relatively insensitive, with only 5–10% of ecosystems changing status outside the bounds of uncertainty when thresholds were adjusted by ±20% (Fig. 8c). The only ecosystem assessable under criterion E (case study 19, Appendix S2) did not change status when criterion E thresholds were varied by up to 20% (Fig. 8f). The sensitivity of individual subcriteria (not shown) was similar to the criteria to which they belong.

### Performance of the Protocol

Several aspects of the case studies show that the IUCN Red List criteria for ecosystems are workable, robust and sufficiently general for application to wide range of ecosystems types and threatening processes. Firstly, the overall status was supported by assessments of multiple subcriteria in 90% of the case studies. This high level of concordance among criteria suggests that assessments are robust because outcomes are unlikely to be very sensitive to missing data.

Secondly, no one criterion had a consistently dominant or subordinate effect on overall status across the full set of case studies. This suggests strong complementarity among criteria. Collectively, they are able to detect symptoms that may signal the susceptibility of an ecosystem to any of several contrasting threatening processes.

Thirdly, close correspondence between Red List status and prior assessments carried out by local experts suggest that the IUCN criteria should not produce markedly different outcomes to most listing processes that currently operate in national and regional jurisdictions.

Fourthly, although poorly studied ecosystems were undersampled in our analysis, the case studies show that suitable data can be obtained from a range of sources and that defensible inferences may be drawn from appropriate use of proxies, various methods of estimation and scaling up.

Several aspects of protocol performance may be attributed to their rule-based structure. This structure promotes the ensemble properties of criteria, minimises the impact of missing data and avoids assumptions that different symptoms are additive or interchangeable in their effect on overall risk of ecosystem collapse [112]. A potential disadvantage of a rule-based structure is that it may underestimate risk if data on the most limiting criteria are lacking or if there are synergistic interactions between different mechanisms of threat [150]. Such interactions can be built into simulation models and used to assess risks of collapse under criterion E.

## Discussion

### Generality and Consistency

Our assessments of widely contrasting ecosystems from terrestrial, subterranean, freshwater and marine environments demonstrate the generality of the Red List criteria. A key feature of our risk assessment model (Figs. 1 and 2) is its generic framework for selecting and assessing ecosystem-specific biotic and abiotic variables to estimate the relative severity of declines in ecosystem function. Range standardisation of severity allows functional changes to be assessed in a wide range of ecosystems against a common set of thresholds. It also forces assessors to be explicit about their choice of functional variable and its threshold values that signal ecosystem collapse.

The common set of thresholds of decline and distribution size that delimit different categories of risk promotes consistency of risk assessments across contrasting terrestrial, subterranean, freshwater and marine ecosystems. Current theory provides limited guidance for setting the precise values of these thresholds. Our choice of thresholds was aimed at promoting informative risk categories based on relatively even intervals of decline, alignment with thresholds of decline in the species Red List protocol, consistency with the monotonic relationships for species - area and biodiversity - ecosystem function, and a broad understanding of the spatial extent of threatening processes. Although these pragmatic principles could also be met by slightly different threshold values, risk assessment outcomes were shown to be only moderately sensitive to variations in decline thresholds and relatively insensitive to variations in thresholds of distribution size. In the most extreme cases, the proportional change in risk classifications was only slightly greater than the proportional adjustment of the thresholds.

Although the flexibility to select appropriate variables for assessment underpins the generality of the protocol, this may have trade-offs if selections are poorly justified. These trade-offs may affect the consistency of assessments if, for example, different assessors select different proxy variables to assess the same or closely related ecosystem(s). An alternative risk assessment method could limit such inconsistency by prescribing one or a few mandatory generic variables to assess functional change (e.g. species richness, productivity, aggregated indices of condition, health or landscape geometry), but only by sacrificing alternative variables that are more proximal to causes and/or more sensitive to functional change. Moreover, a failure to apply ecosystem-specific mechanistic interpretations to trends in generic variables runs a risk of perverse assessment outcomes.

Some inconsistencies between assessments are an inevitable consequence of a risk model that seeks broad generality by incorporating flexibility to select ecosystem-specific measures of function. However, these inconsistencies can be partially offset, firstly by governance processes and standards that promote collaboration and critical evaluation of assessment outcomes (see below), and secondly by using methods to deal with uncertainties described above. Thirdly, the use of cause/effect process models to interpret salient processes and their proxies should mitigate inconsistency, especially if they are critically reviewed, either through peer-reviewed literature or though a structured elicitation process [19], [151]. These models provide a useful basis and context for distinguishing natural variability from functional decline, and help to translate general ecosystem concepts into usable tools [42].

The use of standardised measures of distribution in criterion B also contributes to generality of the protocol and mitigates some of its sensitivity to spatial scale [13]. The agreement between our assessments and those of local authorities for both broadly and narrowly defined ecosystems suggests some robustness to variation in thematic resolution. Nevertheless, risk assessments may be exposed to methodological artefacts if units are defined broadly or too finely. Data will often be more uncertain, fragmentary and more limited as the thematic resolution of assessment units increases and the available data are consequently subdivided among more units. Similarly, if the spatial domain of assessment is too small to consider relevant spatial processes, the outcomes of assessments may simply reflect patch dynamics. Further work is needed to define the limits of scale at which the criteria may be validly applied, and to develop methods to reduce scale-sensitive bias in the assessments as those limits are reached. This will support applications at fine thematic scales, which are sometimes needed for land use planning under national regulatory and legal frameworks (e.g. [43]).

### Uncertainty

Assessments of ecosystem risk will always carry some uncertainty due to incomplete knowledge. This includes measurement uncertainty related to data availability, boundary vagueness and system variability, as well as model uncertainty (including selection of functional variables, see below) due to imperfect understanding of processes. Risk assessments of ecosystems will generally be less certain than species assessments (Fig. 1), largely because of conceptual generalities required to accommodate assessments of a broad range of ecosystems (see below). Some components of measurement uncertainty, such as detectability, however, may be greater in magnitude for many species than ecosystems.

Uncertainties can be incorporated into risk assessment using bounded estimates (Fig. 6; Appendix S2), fuzzy arithmetic, structured elicitation or Bayesian approaches [19]. Model uncertainty may be accommodated by carrying out multiple assessments based on plausible alternative process models [66]. Very high levels of uncertainty may preclude meaningful assessments of any of the criteria, in which case an assessment will produce a ‘Data Deficient’ outcome. However, close collaboration between spatial scientists and process ecologists should ensure that both distributional and functional symptoms of risk are addressed as comprehensively as possible.

### Assessment Units

Unlike species, a widely accepted global classification of ecosystems is currently lacking. Development of a global taxonomy and classification of ecosystems would strengthen the consistency and comparability of assessments between regions and terrestrial/marine realms. It would also help resolve the limits of thematic scaling discussed above. The principal difficulties in delineating units of assessment stem from conceptual uncertainties in the nature of ecosystem properties, with conflicting discrete and continuum models both having strengths and limitations [43]. Abiotic elements of ecosystems are characteristically continuous, creating uncertain boundaries, although zones of transition may be identified where spatial turnover is high relative to adjacent areas, creating the appearance of discrete units at particular scales [152]. Further uncertainties stem from boundary dynamism or divergence between compositional, physical and functional boundaries [45], [62].

In comparison, the global taxonomy for species appears well established and plays an important role in defining units for risk assessment. In recent decades, however, development of cladistic methods and advent of molecular phylogenies are driving a major reconstruction of classifications at multiple levels to resolve polyphyletic taxa. Ongoing alpha taxonomic activity continually increases the number of described taxa, often resulting in new circumscriptions of existing taxa affected by splitting or lumping. Furthermore, the current operational taxonomic units are based on different morphological, biological or evolutionary species concepts, depending on the major taxonomic groups to which they belong, partly for pragmatic reasons and partly due to historical legacies. Successive Red Data Books and Red Lists have thus developed under substantial taxonomic dynamism and inconsistency. This suggests that Red Lists can be functional and reliable conservation tools despite uncertainties in the underlying classification, even though some changes in listings occur solely as a consequence of taxonomic changes [153].

We suggest that development of a global taxonomy for ecosystems can proceed contemporaneously with risk assessment. Indeed, the shortcomings of existing regional taxonomies underscore the need to describe characteristic biota, abiotic features, distribution and an ecological process model as integral components of ecosystem risk assessment. Ideally, the taxonomic framework should be hierarchical, elucidating relationships between assessment units defined at different scales and integrating elements of existing work at global, regional and national levels across terrestrial, subterranean, freshwater and marine environments biomes [48], [49], [51], [100], [154], [155], [156]. Such a framework would permit assessment at multiple thematic scales to suit different needs, including subglobal applications that provide essential support for local conservation planning [157].

### Governance

Developing a Red List of ecosystems will involve ongoing questions about ecosystem description, variable selection, data analysis and model development. This requires a governance structure that promotes technical support and rigorous peer review. Preparation of interpretive guidelines (cf. [79]) and regional training initiatives will build individual and institutional capacity to support a global network of assessors and scientific reviewers, similar to the species specialist groups and the Standards and Petitions Committee within IUCN’s Species Survival Commission (see http: //www.iucn.org/about/work/programmes/species/about_ssc/specialist_groups/directory_specialist_groups/).

## Conclusion

The Red List criteria for ecosystems will establish a consistent, robust, practical and theoretically grounded international standard for risk assessment of biodiversity, complementing the Red List criteria for species. A global Red List can raise awareness of conservation needs in governments, industries and communities worldwide. However, guidelines are also needed to support assessment at regional and national scales, where much conservation action is planned and implemented. A Red List of ecosystems will firstly strengthen global capacity to report on and monitor the status of biodiversity under internationally agreed Aichi targets [39]. Secondly, it will inform priorities and decisions in planning for land and water use, establishment and management of protected areas, economic development and investments under different governance regimes. The latter includes local community projects and international finance of major development projects that are evaluated against environmental risk standards (http: //www.equator-principles.com/). The separate task of setting priorities for these actions also requires inputs on irreplaceability of biodiversity features, cultural valuations, plasticity of demand for ecosystem services and the potential for investments to reduce risks of decline [40], [158]. Finally, an understanding of key services contributed by each ecosystem and the relationship between the symptoms of risk and delivery of services will help the Red List inform sustainable use of ecosystem services. Forging these links will help to avoid scenarios such as the collapse of the Aral Sea ecosystem, which has lead to collapse of a viable fishing industry and declines in human health associated with dust and chemical aerosols liberated from the dry sea bed [78].

Many of the mechanisms and symptoms of species vulnerability are relevant to ecosystems, because species are integral parts of ecosystems. Yet ecosystems embody processes and higher-order components of biodiversity that are difficult or impossible to account for in species-by-species assessment. Whereas species risk assessment rests on population theory, ecosystem risk assessment must draw from a wider array of inter-related theories that deal with continua, niches, fractal geometry, succession, resilience, ecological integrity, biodiversity-ecosystem function and insurance, as well as population theory. The success of ecosystem risk assessment therefore rests on a robust synthesis of conservation planning and process ecology to translate theoretical foundations into a practical assessment protocol that can be applied to a wide variety of ecosystems by specialist assessors with differing backgrounds and limited data.

## Supporting Information

Appendix S1 **Definitions of terms.**
(PDF)

Appendix S2 **Risk assessment case studies for example ecosystems.**
(PDF)
