# Supplementary material for: Scientific Foundations for an IUCN Red List of Ecosystems
Source: PLoS One. 2013 May 8;8(5):e62111. doi: 10.1371/journal.pone.0062111 (PMC3648534; doi:10.1371/journal.pone.0062111)
Supplement: Appendix S1 — Definitions of terms. (PDF) [file pone.0062111.s001.pdf]

Supplementary Material Appendix S1 for:

'Scientific foundations for an IUCN Red List of Ecosystems'

David A. Keith[1,2], Jon Paul Rodríguez[3,4,5,6], Kathryn M. Rodríguez-Clark[3], Emily Nicholson[7], Kaisu Aapala[8], Alfonso Alonso[9], Marianne Asmussen[3,5], Steven Bachman[10], Alberto Basset[11], Edmund G. Barrow[12], John S. Benson[13], Melanie J. Bishop[14], Ronald Bonifacio[15], Thomas M. Brooks[6,16], Mark A. Burgman[17], Patrick Comer[18], Francisco A. Comín[19], Franz Essl[20,21], Don Faber-Langendoen[16], Peter G. Fairweather[22], Robert J. Holdaway[23], Michael Jennings[243], Richard T. Kingsford[1], Rebecca E. Lester[25], Ralph Mac Nally[26], Michael A. McCarthy[7], Justin Moat[10], María A. Oliveira-Miranda[4], Phil Pisanu[15], Brigitte Poulin[27], Tracey J. Regan[7], Uwe Riecken[28], Mark D. Spalding[29], Sergio Zambrano-Martínez[3]

[1]Australian Wetlands Rivers and Landscapes Centre, University of New South Wales, Sydney NSW 2052, Australia. david.keith@unsw.edu.au
[2]NSW Office of Environment and Heritage, Australia.
[3]Centro de Ecología, Instituto Venezolano de Investigaciones Científicas, Venezuela.
[4] Provita, Apartado 47552, Caracas 1041-A, Venezuela.
[5] EcoHealth Alliance, New York, USA.
[6]IUCN Commission on Ecosystem Management and IUCN Species Survival Commission, Gland, Switzerland.
[7]Centre of Excellence for Environmental Decisions, University of Melbourne, Australia.
[8]Finnish Environment Institute, Helsinki, Finland.
[9]Smithsonian Conservation Biology Institute, National Zoological Park, Washington DC, USA.
[10]Royal Botanic Gardens, Kew, England.
[11]Department of Biological and Environmental Science, Ecotekne Center, University of Salento, Lecce, Italy.
[12]IUCN Global Ecosystem Management Programme, Nairobi, Kenya.
[13]Royal Botanic Gardens Trust, Sydney, Australia.
[14]Department of Biological Sciences, Macquarie University, Australia.
[15]Science Resource Centre, Department of Environment and Natural Resources, Australia.
[16]NatureServe, Arlington, USA.
[17]Australian Centre of Excellence for Risk Assessment, University of Melbourne, Australia.
[18]NatureServe, Boulder, Colorado USA.
[19]Pyrenean Institute of Ecology-CSIC. Zaragoza, Spain.
[20]Environment Agency Austria, Vienna, Austria.
[21]Department of Conservation Biology, Vegetation and Landscape Ecology, University of Vienna, Vienna, Austria
[22]School of Biological Sciences, Flinders University, Australia.
[23]Landcare Research, Lincoln, New Zealand.
[24]University of Idaho Department of Geography, Moscow, Idaho, USA.
[25]School of Life and Environmental Sciences, Deakin University, Warrnambool, Australia.

[26]Australian Centre for Biodiversity, Monash University, Australia.
[27]Tour du Valat Research Center, Le Sambuc, Arles, France.
[28]German Federal Agency for Nature Conservation, Bonn, Germany.
[29]The Nature Conservancy and Conservation Science Group, Department of Zoology University of Cambridge, Cambridge, England.

APPENDIX S1. DEFINITIONS OF TERMS

Area of Occupancy - Area of occupancy is defined as the area within its 'extent of occurrence' (see below), which is occupied by an ecosystem (cf. IUCN 2011). The measure reflects the fact that an ecosystem will not usually occur throughout its extent of occurrence, which may contain unsuitable or unoccupied areas. The size of the area of occupancy will be a function of the scale at which it is measured. For assessment using the criteria in Table 3 (see text), it must be estimated by counting the number of occupied 10 × 10 km grid cells (cf. IUCN 2011), excluding those in which the area of the ecosystem accounts for less than 1% (i.e. 1 km^2) of the cell area.

Characteristic native biota - genes, populations, species, assemblages of species and their key interactions that distinguish the ecosystem compositionally from others. It also includes biota that drive ecosystem dynamics, for example as ecosystem engineers, trophic or structural dominants, or functionally unique elements (see examples, Appendix 2), even though they may be common in other ecosystems. Characteristic biota may be defined in terms of functional traits (e.g. guild composition, dispersal spectra, key interacting components, etc.) as well as taxonomic traits. Characteristic biota excludes uncommon or vagrant species that contribute little to its function and may be more common in other ecosystems.

Collapse - A theoretical threshold, beyond which an ecosystem no longer sustains most of its characteristic native biota or no longer sustains the abundance of biota that have a key role in ecosystem organisation (e.g. trophic or structural dominants, unique functional groups, ecosystem engineers, etc.). Collapse has occurred when all occurrences of an ecosystem have moved outside the natural range of spatial and temporal variability in composition, structure and function. Some or many of the pre-collapse elements of the system may remain within a collapsed ecosystem, but their relative abundances may differ and they may be organised and interact in different ways with a new set of operating rules. Ecosystem collapse may be viewed as the analogue of functional extinction in species, which precedes or at least coincides with complete elimination of all characteristic biota.

Continuing decline - A in decline distribution, environmental degradation or disruption of biotic interactions that must i) reduce the ability of an ecosystem to sustain its characteristic native; ii) be non-trivial in magnitude; iii) be likely to continue into the future. Continuing declines may occur gradually or episodically through time. They exclude trivial trends that are unlikely to be associated with declines in characteristic native biota within the ecosystem.

Disruption of biotic interactions - A change in interactions among different groups of biota, or between the biota and the physical environment, that reduces the capacity of the ecosystem to sustain its characteristic native biota (i.e. biotic degradation cf. environmental degradation). Interactions may be between biota within an ecosystem, with biota of another ecosystem or between biota and environmental factors. Assessment of disruption to biotic interactions under criterion D involves the following steps: i) selection of a suitable biotic variable or variables, with justification of its relationship(s) to salient processes of ecosystem dynamics (e.g. with reference to a process model specific to the ecosystem under evaluation); ii) estimate the value of the variable across the distribution of the ecosystem at the end of the assessment period (present day for D1 & D3, 50 yrs in future for D2); iii) estimate how much the biotic variable changed since the beginning of the assessment period (50 yrs ago for D1, present day for D2, 1750 for D3). Generally, patches of the ecosystem that may have been destroyed (e.g. by land conversion) should be excluded from this estimate; iv) calculate the absolute % change in the biotic variable over the assessment period

(this may require temporal interpolation or extrapolation and justification of associated assumptions); v) range-standardise the estimated of absolute % change using a collapse threshold estimated specifically for the ecosystem to obtain an estimate of relative severity of degradation; vi) estimate the extent (as % of the ecosystem distribution) over which the change has occurred; and vii) compare the estimates of relative severity and extent to the assessment thresholds under criterion D (Table 3, main text). Example calculations are given in Fig. 6 and Appendix S2.

Distribution - The spatial occurrence of an ecosystem. For criterion A, changes in distribution should be estimated with the best available mapping of an appropriate surrogate for the ecosystem (e.g. remote sensing of terrestrial vegetation, marine reefs, etc.). For criterion B, distribution size must be estimated using the standard metrics - see definitions of Area of Occupancy (AOO), Extent of Occurrence (EOO), Locations.

Ecosystem - Complexes of organisms and their associated physical environment, within an area (after Tansley 1935). They have four essential elements: a biotic complex; an abiotic environment or complex; the interactions within and between them; and a physical space in which these operate (Pickett & Cadanesso 2002).

Environmental degradation - A change in the abiotic features of an ecosystem that reduce its capacity to sustain its characteristic native biota. Assessment of environmental degradation under criterion C involves the following steps: i) selection of a suitable environmental variable or variables, with justification of its relationship(s) to salient processes of ecosystem dynamics (e.g. with reference to a process model specific to the ecosystem under evaluation); ii) estimate the value of the variable across the distribution of the ecosystem at the end of the assessment period (present day for C1 & C3, 50 yrs in future for C2); iii) estimate how much the degradation variable changed since the beginning of the assessment period (50 yrs ago for C1, present day for C2, 1750 for C3). Generally, patches of the ecosystem that may have been destroyed (e.g. by land conversion) should be excluded from this estimate; iv) calculate the absolute % change in the degradation variable over the assessment period (this may require temporal interpolation or extrapolation and justification of associated assumptions); v) range-standardise the estimated of absolute % change using a collapse threshold estimated specifically for the ecosystem to obtain an estimate of relative severity of degradation; and vi) estimate the extent (as % of the ecosystem distribution) over which the degradation has occurred. Example calculations are given in Fig. 5 and Appendix 2.

Estimated - Information that is based on calculations that may include statistical assumptions about sampling, or biological assumptions about the relationship between an observed variable (e.g. an index of abundance of a key species) to the variable of interest (e.g. biotic interactions) (cf. 2011). These assumptions should be stated and justified in the documentation supporting and assessment. Estimation may also involve interpolation in time to calculate the variable of interest for a particular time step (e.g., a 50-year reduction in distribution based on observations or estimations of distribution 40 and 60 years ago).

Extent of Occurrence - Extent of occurrence is the area contained within the shortest continuous imaginary boundary which can be drawn to encompass all the known, inferred or projected sites of present occurrence of an ecosystem (cf. IUCN 2001). For assessment using the criteria in Table 3 (see text), it must be estimated using a minimum convex polygon (the smallest polygon in which no internal angle exceeds 180 degrees and which contains all the sites of occurrence).

Inferred - Information that is based on indirect evidence, on variables that are indirectly related to the variable of interest, but in the same general type of units (IUCN 2011). Inferred values rely on more assumptions than estimated values. For example, inferring disruption of biotic interactions from catch statistics not only requires statistical assumptions (e.g., random sampling) and biological assumptions (about the relationship of the harvested section of the population to the total population), but also assumptions about trends in effort, efficiency, and spatial and temporal distribution of the harvest in relation to the population. Inference may also involve extrapolating an observed or estimated quantity from known ecosystem occurrences to calculate the same quantity for other occurrences. Whether there are enough data to make such an inference will depend on how large the known occurrences are as a proportion of the whole distribution, and the applicability of the threats and trends observed in the known occurrences to the rest of the ecosystem. The method of extrapolating to unknown occurrences depends on the criteria and on the type of data available for the known occurrences.

Location - A geographically or ecologically distinct area in which a single threatening event can rapidly affect the ecosystem (cf. IUCN 2001). The size of the location depends on the area covered by the threatening event and may include part of one or many separate patches of the ecosystem. Where an ecosystem is affected by more than one threatening event, location should be defined by considering the most serious plausible threat. IUCN (2001) and Appendix 2 (below) contains further guidance and examples to support the interpretation of the 'location' concept.

Observed - information that is directly based on well-documented observations of all known occurrences of the ecosystem (cf. IUCN 2011).

Projected - **S**ame as “estimated”, but the variable of interest is extrapolated in time towards the future (IUCN 2011). Projected variables require a discussion of the method of extrapolation (e.g. justification of the statistical assumptions or the ecosystem model used) as well as the extrapolation of current or potential threats into the future, including their rates of change.

Relative severity - The magnitude of a decline in ecosystem function (criteria C and D) expressed as a percentage change in a relevant biotic or abiotic variable relative to a decline that would be large enough to exceed an ecosystem-specific threshold of collapse (see Fig. 6 in main text).

Threat - A tractable agent, mechanism or process that causes either a continuing decline in distribution, continuing environmental degradation or continuing disruption of biotic interactions or a future decline in those factors that is likely to occur in the near future (i.e. within 20 years).
