# Supplementary material for: Scientific Foundations for an IUCN Red List of Ecosystems
Source: PLoS One. 2013 May 8;8(5):e62111. doi: 10.1371/journal.pone.0062111 (PMC3648534; doi:10.1371/journal.pone.0062111)
Supplement: Appendix S2 — Risk assessment case studies for example ecosystems. (PDF) [file pone.0062111.s002.pdf]

Supplementary Material Appendix S2 for:

'Scientific foundations for an IUCN Red List of Ecosystems'

David A. Keith[1,2], Jon Paul Rodríguez[3,4,5,6], Kathryn M. Rodríguez-Clark[3], Emily Nicholson[7], Kaisu Aapala[8], Alfonso Alonso[9], Marianne Asmussen[3,5], Steven Bachman[10], Alberto Basset[11], Edmund G. Barrow[12], John S. Benson[13], Melanie J. Bishop[14], Ronald Bonifacio[15], Thomas M. Brooks[6,16], Mark A. Burgman[17], Patrick Comer[18], Francisco A. Comín[19], Franz Essl[20,21], Don Faber-Langendoen[16], Peter G. Fairweather[22], Robert J. Holdaway[23], Michael Jennings[243], Richard T. Kingsford[1], Rebecca E. Lester[25], Ralph Mac Nally[26], Michael A. McCarthy[7], Justin Moat[10], María A. Oliveira-Miranda[4], Phil Pisanu[15], Brigitte Poulin[27], Tracey J. Regan[7], Uwe Riecken[28], Mark D. Spalding[29], Sergio Zambrano-Martínez[3]

[1]Australian Wetlands Rivers and Landscapes Centre, University of New South Wales, Sydney NSW 2052, Australia. david.keith@unsw.edu.au
[2]NSW Office of Environment and Heritage, Australia.
[3]Centro de Ecología, Instituto Venezolano de Investigaciones Científicas, Venezuela.
[4] Provita, Apartado 47552, Caracas 1041-A, Venezuela.
[5] EcoHealth Alliance, New York, USA.
[6]IUCN Commission on Ecosystem Management and IUCN Species Survival Commission, Gland, Switzerland.
[7]Centre of Excellence for Environmental Decisions, University of Melbourne, Australia.
[8]Finnish Environment Institute, Helsinki, Finland.
[9]Smithsonian Conservation Biology Institute, National Zoological Park, Washington DC, USA.
[10]Royal Botanic Gardens, Kew, England.
[11]Department of Biological and Environmental Science, Ecotekne Center, University of Salento, Lecce, Italy.
[12]IUCN Global Ecosystem Management Programme, Nairobi, Kenya.
[13]Royal Botanic Gardens Trust, Sydney, Australia.
[14]Department of Biological Sciences, Macquarie University, Australia.
[15]Science Resource Centre, Department of Environment and Natural Resources, Australia.
[16]NatureServe, Arlington, USA.
[17]Australian Centre of Excellence for Risk Assessment, University of Melbourne, Australia.
[18]NatureServe, Boulder, Colorado USA.
[19]Pyrenean Institute of Ecology-CSIC. Zaragoza, Spain.
[20]Environment Agency Austria, Vienna, Austria.
[21]Department of Conservation Biology, Vegetation and Landscape Ecology, University of Vienna, Vienna, Austria
[22]School of Biological Sciences, Flinders University, Australia.
[23]Landcare Research, Lincoln, New Zealand.
[24]University of Idaho Department of Geography, Moscow, Idaho, USA.
[25]School of Life and Environmental Sciences, Deakin University, Warrnambool, Australia.
[26]Australian Centre for Biodiversity, Monash University, Australia.
[27]Tour du Valat Research Center, Le Sambuc, Arles, France.
[28]German Federal Agency for Nature Conservation, Bonn, Germany.
[29]The Nature Conservancy and Conservation Science Group, Department of Zoology University of Cambridge, Cambridge, England.

APPENDIX S2. RISK ASSESSMENT CASE STUDIES FOR EXAMPLE ECOSYSTEMS

This collection of case studies demonstrates interpretation of concepts, selection of variables and calculation of estimates required for application of the IUCN Red List criteria for Ecosystems version 2.0 presented in the accompanying paper (Table 3). The example ecosystems come from continental aquatic, terrestrial, marine and subterranean environments in different parts of the world, and are subject to a range of different threatening processes. They span a range of data quality and availability. The reference table below summarizes the criteria and concepts addressed in each case study (see contents table for quick reference).

Example of:	1	2	3	4	5	6	7	8	9	10	11	12	13	14	15	16	17	18	19	20
Criterion A1 current decline in distribution	x	x	x		x	x	x	x	x		x	x	x	x				x	x	x
Criterion A2 future decline in distribution	x	x			x						x	x	x	x				x	x	
Criterion A3 historic decline in distribution	x	x	x		x	x	x	x	x	x	x	x	x	x	x					x
Criterion B1 extent of occurrence (polygon)	x		x	x	x	x	x	x	x		x	x	x	x	x	x	x	x	x	x
Criterion B2 area of occupancy (grids)	x	x		x	x	x	x	x	x	x	x	x	x	x	x		x	x	x	x
Criterion B3 number of locations	x			x	x	x	x	x	x		x	x	x	x	x		x	x	x	x
Criterion C1-3 environmental degradation	x	x		x	x	x	x	x	x			x						x	x	x
Criterion D1-3 disruption of biotic processes	x			x	x	x		x			x	x			x	x	x		x	
Criterion E quantitative risk estimate																			x	
Classification system	x	x	x			x	x		x	x	x	x	x		x		x			
Calculating declines - interpolation, extrapolation, inference from subsamples	x		x	x			x	x	x					x		x	x	x		
Distribution models	x							x						x						
Estimating minimum convex polygon (EOO)	x			x			x		x		x		x	x	x			x		
Estimating occupied grid cells (AOO)	x						x		x		x		x	x				x		
Process models for variable selection	x			x	x		x	x								x	x	x	x	x
Functional types as biotic response variables	x			x		x										x			x	
Dealing with measurement uncertainty - bounded estimates	x			x	x		x	x	x						x	x	x	x		
Dealing with model uncertainty - alternative plausible models	x							x								x				
National/regional assessment		x								x								x		

Case studies:
1 Coastal sandstone upland swamps, Australia
2 Raised bogs, Germany (national assessment)
3 German tamarisk pioneer vegetation, Europe
4 Swamps, marshes and lakes in the Murray-Darling Basin, Australia
5 Aral Sea, Uzebekistan and Kazakhstan
6 Reedbeds, Europe
7 Gonakier forests for the Senegal River floodplain, Senegal - Mauritania
8 Floodplain Ecosystem of river red gum and black box, south-eastern Australia
9 Coolibah - Black Box woodland, Australia
10 Semi-evergreen vine thicket, Australia (state assessment)
11 Tepui shrubland, Venezuela
12 Granite gravel fields & sandplains, New Zealand
13 Cape Sand Flats Fynbos, South Africa
14 Tapia Forest, Madagascar
15 Great Lakes Alvar, North America
16 Giant kelp forests, Alaska
17 Caribbean coral reefs, Northwest Atlantic
18 Seagrass meadows of South Australia (state assessment)
19 Coorong Lagoon, Australia
20 Karst Rising Springs, South Australia

Contents

1 Coastal Sandstone Upland Swamps, south-eastern Australia 11
Classification 11
Ecosystem description 11
Characteristic native biota 11
Abiotic environment 12
Distribution 12
Key processes and interactions 12
Threatening processes 13
Ecosystem collapse 14
Assessment 14
Summary 14
Criterion A 14
Criterion B 16
Criterion C 18
Criterion D 18
Criterion E 19
References 19
2 Raised bogs of Germany (national assessment) 21
Background to national Red List of habitat types in Germany 21
Classification 21
Ecosystem description 21
Characteristic native biota 21
Abiotic environment and Distribution 21
Threatening processes 22
Ecosystem collapse 23
Assessment 23
Summary 23
Criterion A 23
Criterion B 24
Criterion C 25
Criterion D 26
Criterion E 26
Overall status 26
References 26
3 German tamarisk-pioneer vegetation (GTPV), Europe 28
Classification 28
Ecosystem Description 28
Characteristic native biota 28
Abiotic environment 28
Distribution 30
Threatening Processes 30
Ecosystem collapse 31
Assessment 31
Summary 31
Criterion A 31
Criterion B 33

Criterion C 33
Criterion D 34
Criterion E 34
References 34
4 Swamps, marshes and lakes in the Murray-Darling Basin, Australia 36
Classification 36
Ecosystem description 36
Characteristic native biota 36
Abiotic environment 38
Distribution 38
Key processes and interactions 39
Threatening processes 40
Ecosystem collapse 41
Assessment 41
Summary 41
Criterion A 41
Criterion B 42
Criterion C 42
Criterion D 44
Criterion E 47
References 47
5 Aral Sea, Uzebekistan and Kazakhstan 50
Classification 50
Ecosystem Description 50
Characteristic native biota 50
Abiotic environment 51
Distribution 52
Key processes and interactions 53
Threatening Processes 54
Ecosystem collapse 55
Assessment 57
Summary 57
Criterion A 57
Criterion B 59
Criterion C 59
Criterion D 61
Criterion E 62
References 63
6 European reedbeds 64
Classification 64
Ecosystem description 64
Characteristic native biota 64
Abiotic environment 65
Distribution 65
Key processes and interactions 66
Threatening processes 66
Ecosystem collapse 66
Assessment 66
Summary 66
Criterion A 67

Criterion B ... 68
Criterion C ... 68
Criterion D ... 68
Criterion E ... 69
References ... 69
7 Gonakier Forests of Senegal River Floodplain, Senegal and Mauritania ... 73
Classification ... 73
Ecosystem Description ... 73
Characteristic native biota ... 73
Abiotic environment ... 73
Distribution ... 74
Key processes and interactions ... 74
Threatening Processes ... 75
Ecosystem collapse ... 76
Assessment ... 77
Summary ... 77
Criterion A ... 77
Criterion B ... 78
Criterion C ... 79
Criterion D ... 80
Criterion E ... 80
References ... 80
8 The Floodplain Ecosystem of river red gum and black box, south-eastern Australia 82
Classification ... 82
Ecosystem description ... 82
Characteristic native biota ... 82
Abiotic environment ... 82
Distribution ... 83
Ecological dynamical model of the Floodplain Ecosystem and its threatening processes 83
Ecosystem collapse ... 86
Assessment ... 86
Summary ... 86
Criterion A: Reduction in Geographic Distribution ... 86
Criterion B ... 87
Criterion C ... 88
Criterion D ... 91
Criterion E ... 93
References ... 93
9 Coolibah - Black Box Woodlands, south-eastern Australia ... 95
Classification ... 95
Ecosystem description ... 95
Characteristic native biota ... 95
Abiotic environment ... 96
Distribution ... 96
Key processes and interactions ... 97
Threatening processes ... 97
Ecosystem collapse ... 97
Assessment ... 98
Summary ... 98
Criterion A ... 98

Criterion B ... 99
Criterion C ... 100
Criterion D ... 101
Criterion E ... 101
References ... 101
10 Mock Olive - Wilga - Peach Bush - Carissa dry sub-tropical semi-evergreen vine thicket in south eastern Australia. ... 104
Classification ... 104
Ecosystem Description ... 104
Characteristic Flora and Vegetation ... 104
Characteristic Fauna ... 105
Abiotic environment and Distribution ... 107
Threatening Processes ... 107
Ecosystem collapse ... 109
Assessment ... 109
Summary ... 109
Criterion A ... 109
Criterion B ... 109
Criterion C ... 109
Criterion D ... 109
Criterion E ... 110
References ... 110
11 Tepui shrublands, southern Venezuela ... 111
Classification ... 111
Ecosystem description ... 111
Characteristic native biota ... 111
Abiotic environment ... 113
Distribution ... 113
Key processes and interactions ... 114
Threatening processes ... 114
Ecosystem collapse ... 115
Assessment ... 115
Summary ... 115
Criterion A ... 115
Criterion B ... 116
Criterion C ... 116
Criterion D ... 117
Criterion E ... 117
References ... 118
12 Granite Gravel Fields & Sand Plains, New Zealand ... 120
Classification ... 120
Ecosystem description ... 120
Distribution ... 120
Abiotic environment ... 121
Characteristic native biota ... 121
Key processes and interactions ... 121
Threatening processes ... 123
Assessment ... 123
Summary ... 123
Criterion A ... 123

Criterion B 123
Criterion C 124
Criterion D 124
Criterion E 124
References 125
13 Cape Flats Sand Fynbos, South Africa 126
Classification 126
Ecosystem description 126
Characteristic native biota 126
Abiotic environment 127
Distribution 127
Key processes and interactions 128
Threatening processes 129
Ecosystem collapse 130
Assessment 130
Summary 130
Criterion A 130
Criterion B 131
Criterion C 131
Criterion D 131
Criterion E 131
References 132
14 Tapia Forest, Madagascar 133
Classification 133
Ecosystem description 133
Characteristic native biota 133
Abiotic environment 134
Distribution 134
Key processes and interactions 134
Threatening processes 135
Ecosystem collapse 135
Assessment 135
Summary 135
Criterion A 135
Criterion B 137
Criteria C and D 138
Criterion E 138
References 138
15 Great Lakes Alvar, North America 140
Classification 140
Ecosystem description 140
Characteristic native biota 140
Abiotic environment 140
Distribution 141
Key processes and interactions 142
Threatening processes 143
Ecosystem collapse 143
Assessment 144
Summary 144
Criterion A 144

Criterion B 145
Criterion C 145
Criterion D 146
Criterion E 146
References 146
16 Giant Kelp Forests, Alaska 148
Classification 148
Ecosystem description 148
Characteristic native biota 148
Abiotic environment 149
Distribution 149
Key processes and interactions 149
Threatening processes 151
Ecosystem collapse 152
Assessment 152
Summary 152
Criterion A 152
Criterion B 152
Criterion C 153
Criterion D 153
Criterion E 154
References 154
17 Caribbean coral reefs 157
Classification 157
Ecosystem description 157
Characteristic native biota 157
Abiotic environment 160
Distribution 160
Key processes and interactions 160
Threatening processes 162
Ecosystem collapse 163
Assessment 163
Summary 163
Criterion A 163
Criterion B 164
Criterion C 165
Criterion D 166
Criterion E 168
References 168
18 Seagrass Community of South Australia 172
Classification 172
Ecosystem description 172
Characteristic native biota 172
Abiotic environment 173
Distribution 173
Key processes and interactions 174
Threatening processes 174
Ecosytem collapse 175
Assessment 176
Summary 176

Criterion A 176
Criterion B 178
Criterion C 180
Criterion D 182
Criterion E 183
References 183
19 The Coorong lagoons and Murray Mouth inverse estuary, South Australia 185
Classification 185
Ecosystem description 185
Characteristic native biota 185
Abiotic characteristics 187
Key processes and interactions 188
Threatening processes 189
Ecosystem collapse 189
Assessment 190
Summary 190
Criterion A 190
Criterion B 190
Criterion C 191
Criterion D 193
Criterion E 193
Acknowledgments 196
References 196
20 Karst Rising-Spring (KRS) Wetland Community of the South East (SA) 198
Classification 198
Ecosystem description 198
Characteristic native biota 198
Abiotic environment 199
Distribution 200
Key processes and interactions 201
Threatening processes 202
Ecosystem collapse 204
Assessment 204
Summary 204
Criterion A 204
Criterion B 205
Criterion C 208
Criterion D 211
Criterion E 211
References 211

1 COASTAL SANDSTONE UPLAND SWAMPS, SOUTH-EASTERN AUSTRALIA

contributed by David Keith, Australian Wetlands and Rivers Centre, University of New South Wales and NSW Office of Environment & Heritage

CLASSIFICATION

International: Most Australian vegetation has not yet been classified in an international system. Regionally, this ecosystem is classified as an Endangered Ecological Community, 'Coastal upland swamps in the Sydney Basin bioregion' under New South Wales legislation (NSW Scientific Committee 2011), also 'Coastal Upland Swamp (map unit FrWp129)' according to Tozer et al. (2010). It does not occur outside New South Wales.
IUCN Habitats Classification Scheme (Version 3.0): 5. Wetlands (inland) / 5.4 Bogs, Marshes, Swamps, Fens, Peatlands.
Key references: Keith & Myerscough (1993), Tozer et al. (2010), NSW Scientific Committee (2011).

ECOSYSTEM DESCRIPTION

Characteristic native biota

Upland swamps are soligenous valley bogs, characteristised by a diverse assemblage of hydrophilous, mostly sclerophyllous shrubs, graminoids and forbs (Keith & Myerscough 1993). These bogs are essentially treeless and form relatively abrupt boundaries with surrounding eucalypt-dominated forests and woodlands that occupy more freely draining soils (Fig. 1). Many of the plant species that characterise the bogs are absent from or rare in the surrounding matrix, making them highly distinctive and easily recognised landscape features. Large numbers of characteristic plant taxa belong to families Cyperaceae, Restionaceae, Proteaceae, Myrtaceae, Fabaceae, Ericaceae, Haloragaceae and Droseraceae. Detailed descriptions of the vegetation are given by Keith & Myerscough (1993), Tozer et al. (2010) and NSW Scientific Committee (2011). Although their characteristic vertebrate fauna also occurs in surrounding forests and woodlands, the swamps provide essential resources including permanent water during drought and shelter from predators in dense vegetation cover. The macro-invertebrate fauna and micro-invertebrate stygofauna is highly distinctive, with species of burrowing crayfish, dragonflies, arachnids, hemipterans, dipterans and collembola dependent on the hydrological conditions or host plants that characterise the swamps (Bailey 2010; NSW Scientific Committee 2011).

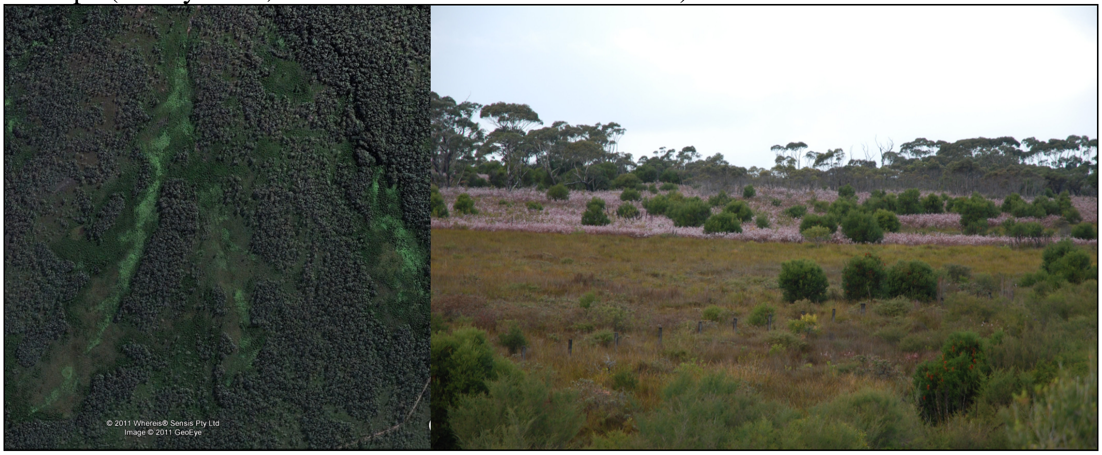

Figure S1. 1. A typical example of Coastal sandstone upland swamp embedded in a matrix of eucalypt forest and woodland from Dharawal Nature Reserve, 45 km south of Sydney, Australia. Note distinctive vegetation zonation within swamps.

Abiotic environment

Coastal sandstone upland swamps are strongly associated with high levels of climatic moisture (mean annual precipitation >1000 mm and exceeding evapotranspiration), low-relief, elevated terrain and poorly drained mineral-organic and peaty sediments on relatively impermeable sandstone substrates (Keith & Myerscough 1993). Several geomorphological and hydrological features of the swamps are typical of those found in uplands on all continents except Antarctica (Gore 1983).

Distribution

Coastal sandstone upland swamps are scattered on sandstone plateaux to the north and south of Sydney (Figure 2).

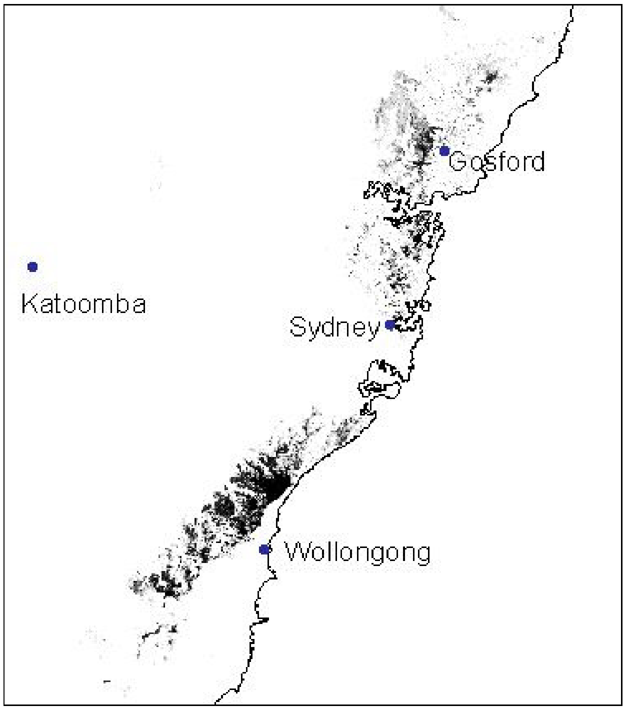

Figure S1. 2. Distribution of Coastal sandstone upland swamps.

Key processes and interactions

Interactions between hydrological processes and fire regimes are crucial to the development of upland swamps and maintenance of their diverse and characteristic biota (Figure 3). Development and persistence of the mire depends on an excess of precipitation over evapotranspiration combined with high surface run-on and low rates of low percolation and run-off. This promotes soil waterlogging, development of dense vegetation and sediment deposition, with positive feedbacks resulting in further impedance to surface and subsurface flow (Young 1982, 1986). Fires regulate plant regeneration niches and vegetation turnover, maintaining fine-scale diversity (Keith et al. 2007). Distinctive zonations of plant assemblages within the swamps (Figure 2 & 3) contribute to high beta diversity and are structured by hydrological gradients and recurring fires (Keith & Myerscough 1993). Variations in hydrological conditions combine with particular fire events (e.g. short fire intervals, intense crown fires, peat fires) to regulate transitions between these assemblages, resulting in a dynamic mosaic over decadal time scales (Keith et al. 2006; 2007; 2010a).

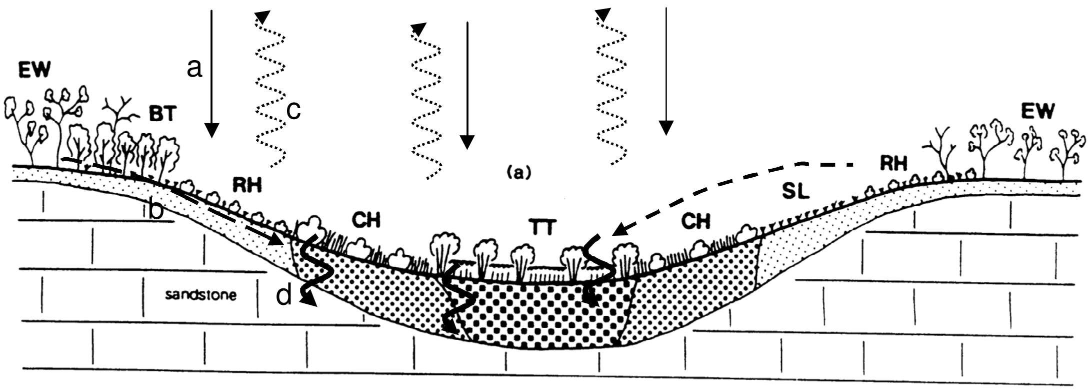

Figure S1. 3. Schematic cross section of upland swamp showing zonation of plant assemblages within upland swamps (TT- Ti-tre Thicket, CH- Cyperiod Heath, SL- Sedgeland, RH- Restioid Heath, BT- Banksia Thicket) and surrounding eucalypt woodland (EW) in relation to soil types with increasing organic matter content (represented by increasingly coarse stippling). Hydrological processes involve an excess of precipitation (a) and run-on/seepage (b) over evapotranspiration (c), percolation (d) and run-off/seepage discharge (not shown, perpendicular to page surface).

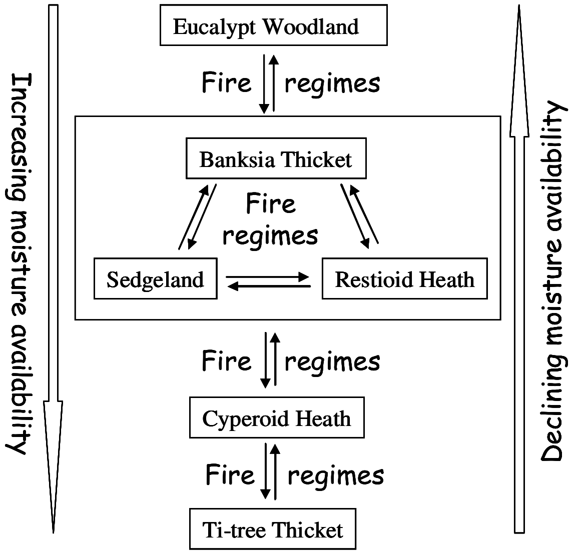

Figure S1. 4. A cause-effect process model summarising ecosystem dynamics in coastal sandstone upland swamps (adapted from Keith et al. 2006; 2007; 2010).

THREATENING PROCESSES

Three main processes threaten the persistence of this ecosystem. First, extraction of coal and/or gas from underground coal seams beneath the swamps affects their hydrological function by shattering bedrock strata, warping the land surface and disrupting perched aquifers (NSW Scientific Committee 2011). Impacts are highly variable, depending on patterns of bedrock jointing and subsidence, but may include increased percolation, decline of piezometric levels, water quality impacts from unconfinement of groundwater, creation of

erosion knick points, valley closure and redistribution of overland flows (Krogh 2007). Second, severe fires may initiate shifts in swamp/woodland boundaries, consumption of peat or erosion. These effects may be mediated or amplified by extremes in post-fire rainfall (Keith et al. 2010). Fire frequencies may also mediate diversity through differential selection of plant life history types (Keith et al. 2007). Third, swamps may be threatened by global climate change through its effects on regional hydrology and fire regimes (Keith et al. 2010a). IPCC projections suggestion declining precipitation, increased evapotranspiration and increased frequency of extreme fire weather days (Hennessy et al. 2004; Lucas et al. 2006).

Ecosystem collapse

For assessment of criteria A and B, collapse was assumed to occur when the mapped distribution of the ecosystem declines to zero, signalling the replacement of upland swamp either by developed areas (e.g. quarries, mines, rural-residential development and roading) or when by dryland ecosystems (e.g. woodland), due to changes in hydrology and fire regimes. Under future scenarios, a collapsed state was assumed when there were no areas remaining of even low modelled hydrological suitability based on the hydrological characteristics of the current distribution of the ecosystem. For criterion C, ecosystem collapse was defined using models of hydrological suitability based on precipitation, evapotranspiration, slope and substrate. Collapse was assumed to occur when summed hydrological suitability reaches zero. Under criterion D, the abundance of woody respouters was identified as the most appropriate biotic variable for assessment, as these species provide crucial habitat structure and sediment stability in the early post-fire environment and are the most sensitive functional group of swamp plants to adverse fire regimes. Collapse was assumed to occur when the abundance of woody resprouters declined to zero.

ASSESSMENT

Summary

Criterion	A	B	C	D	E	overall
subcriterion 1	LC	EN	LC	NT(NT-VU)	DD	EN(EN-CR)
subcriterion 2	EN(EN-CR)	EN	EN(EN-CR)	DD		
subcriterion 3	LC	LC	DD	DD		

Criterion A

Current decline: In a sample catchment located centrally within the distribution, Keith et al. (2010) compared the extent of upland swamps on aerial photographs flown in 1961 and 1998. They found a 10.2% net expansion of swamps into surrounding woodland (range -3 to 32% between subcatchments), coincident with moistening climatic conditions over that period and a major fire in the late 1960s. In addition, within this same area, it is estimated that approximately 5 to 7% of the area of swamps has been destroyed by quarries, mines, rural-residential development and roading in the past 50 years (Keith 1994 and unpubl. data). Combining these estimates produces an estimated -10 to +27% change in the distribution of the ecosystem over the past 50 years. Assuming the study area of Keith et al. (2010) is representative of trends across the whole distribution of Coastal sandstone upland swamps, the status of the ecosystem is Least Concern in under criterion A1. This assumption appears reasonable, given that the study area is centrally located within the core range of the ecosystem and includes both protected and unprotected land tenures.

Future decline: Global climate change may threaten the future persistence of Coastal sandstone upland swamps, given their hydrological dependencies (Figure 2). Keith et al. (2011) developed bioclimatic distribution models for the ecosystem to predict its distribution under future climate scenarios. They developed a range of plausible models based on climatic and terrain variables that were selected to represent the hydrological processes represented in Figure 2 and projected the distributions into the future using two different emission scenarios. Based on these models and scenarios, the distribution of the ecosystem was projected to decline by 58-90% (median 74%) over the next 50 years (Figures 5 & 6). Changes in land use may add to these estimates of decline, but the majority of the ecosystem is within conservation reserves and protected water catchments (Tozer et al. 2010). The status of the ecosystem is therefore Endangered (plausible range Endangered - Critically Endangered under criterion A2. This estimates does not include changes to hydrology that may be brought about by extraction of underground coal and gas.

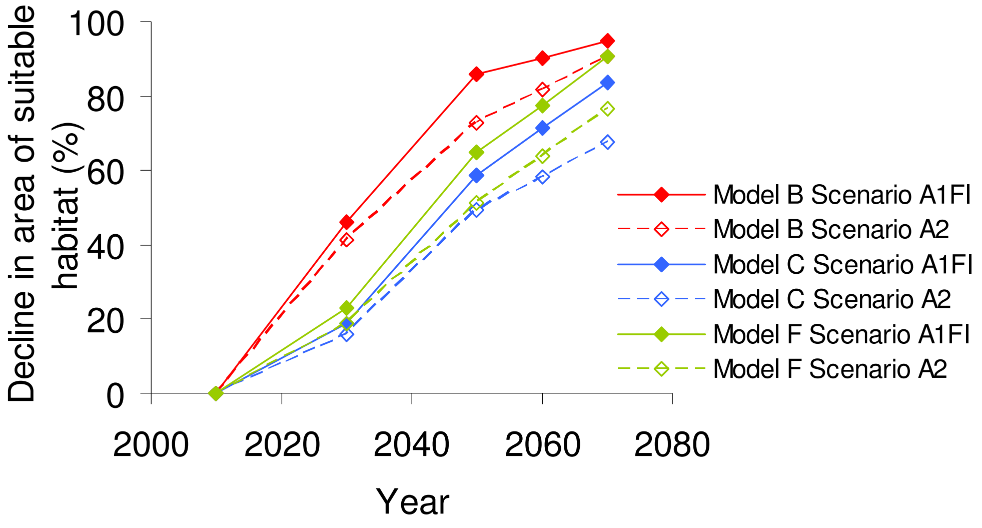

Figure S1. 5. Modelled trends in the distribution of Coastal sandstone upland swamps under climate change scenarios from AR4 (after Keith et al. 2011). See Figure 6 for example maps of projections based on model C and A1FI scenario.

Historic decline: There is insufficient data to estimate changes in swamp-woodland boundaries since 1750. However, 90% the current distribution of the ecosystem has been mapped from recent high resolution aerial photography (Tozer et al. 2010;) and, using historical imagery and mapping of headwater valleys in climatically suitable areas, this distribution has been interpolated into areas that have been transformed by human land use since 1750. Based on this mapping and interpolation, the decline in distribution since 1750 was estimated to be 6 to 8%. Based on its similar landscape and human activity, the remaining 10% of the distribution was assumed to have undergone a similar decline. The status of the ecosystem is therefore Least Concern under criterion A3.

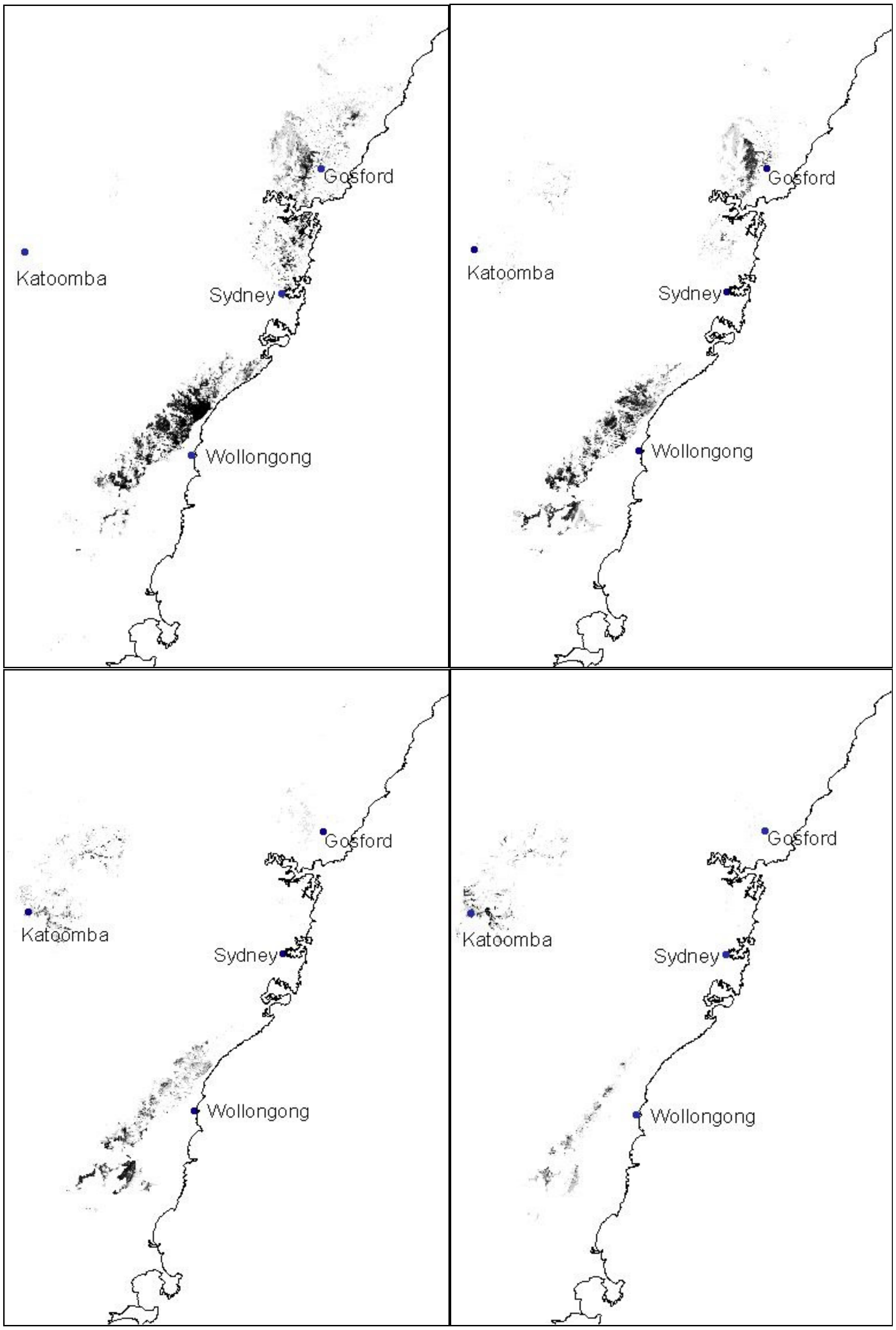

Figure S1. 6. Future distribution of Coastal sandstone upland swamps projected by a distribution model including wetness (incorporating precipitation and evapotranspiration), maximum and minim temperatures and topographic roughness under climate change (AR4 scenario A1FI).

Criterion B

Approximately 5360 ha of Coastal sandstone upland swamps have been mapped from recent high resolution aerial photography (NPWS 2000, DECCW 2009, Tozer *et al.* 2010). The

swamps occur naturally in small patches, with approximately 42% of mapped swamps covering less than 1 ha, and making up about 6% of the total mapped area.

Extent of occurrence: A minimum convex polygon enclosing all mapped occurrences of Coastal sandstone upland swamps (Figure 7) has an area of 4960 [4730-5200] km^2 (NSW Scientific Committee 2011). There is weak evidence of a current decline in distribution (subcriterion B1a), although the distribution could be currently stable (see criterion A1). Global climate change is projected to cause a future decline in distribution (subcriterion B1b), based on a substantial contraction in hydrological and climatic environment suitable for swamp persistence (see criterion A2). Underground coal and gas extraction and severe wildland fires are the most serious plausible threats to persistence of the swamps. Based on spatial extent and location of current mining leases and previous fire events, Coastal sandstone upland swamps are estimated to occupy five locations (subcriterion B1c). The status of the ecosystem is therefore Endangered under criteria B1b and B1c.

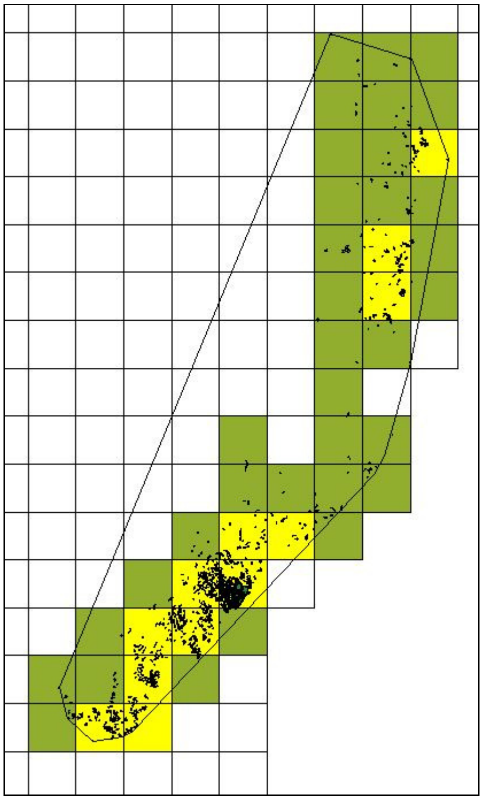

Figure S1. 7. Mapped distribution of Coastal sandstone upland swamps showing minimum convex polygon enclosing all occurrences (Extent of occurrence) and occupied 10 × 10 km grid cells (Area of occupancy). Green- all occupied cells. Yellow- cells with more than 1% of cell area occupied.

Area of occurrence: Superimposing a 10 km grid over the mapped polygons of Coastal sandstone upland swamps (Figure 7) indicates that they are present within 46 grid cells. Of these, 34 grid cells contains less than 1 km^2 of the ecosystem (i.e. <1% of the area of a grid cell). Excluding these small occurrences, the swamps are therefore estimated to occupy twelve 10 × 10 km grid cells. Assessments of B2 subcriteria are identical to those for criterion B1 (see Extent of occurrence, above). The status of the ecosystem is therefore Endangered under criteria B2b and B2c.

Number of locations: Based on underground coal and gas extraction and severe wildland fires as the most serious plausible threats, Coastal sandstone upland swamps are estimated to occupy five locations (see Extent of occurrence, above). Although serious, these threats seem unlikely to result in collapse or Critically Endangered status within a short time period due to ecological time lags in responses. The status of the ecosystem is therefore Least Concern under criteria B3.

Criterion C

The principal mechanism of environmental degradation is through declines in hydrological processes related to global climate change (Figures 2 & 3). Suitable hydrological variables for assessing criterion C include stream discharge volumes from swamp catchments, rates of precipitation and evapotransipration and organic carbon stocks and fluxes within the swamps. Data from stream flow gauges are available, but only for a few relatively large catchments. No data were available on stocks and fluxes of organic carbon.

Current decline: Trends in climatic data from regional weather stations suggest an increase in climatic moisture has occurred over recent decades, driven primarily by a steady decline in pan evaporation (Keith et al. 2010a). The status of the ecosystem is therefore Least Concern under criteria C1.

Future decline: Despite trends in recent decades, global circulation models project a decline in climatic moisture for the region in which this ecosystem occurs. The same distribution models used to assess future change in distribution (Figure 6) were also used to assess trends in climatic suitability. Predicted suitability values summed across the whole distribution were calculated for present day (2010) and fifty years into the future (2060). It was assumed that the proportional change in summed climatic suitability was assumed to represent the severity of environmental degradation and that ecosystem collapse occurs when summed suitability reaches zero. Based on the range of distribution models and emission scenarios described above, the proportional change in suitability (= severity of degradation) was estimated to be 62-93% (median 78%) over the next 50 years. As the ratio of summed suitability averages trends across the entire distribution, the estimate of severity applies across 100% of ecosystem extent. The status of the ecosystem is therefore Endangered (plausible range Endangered - Critically Endangered) under criterion C2.

Historic decline: There are currently insufficient spatial climatic data to assess changes in climatic suitability since 1750. The status of the ecosystem is therefore Data Deficient under criterion C3.

Criterion D

Current decline. Fire-mediated competition between overstorey shrubs and understorey plants is an important biotic interaction within upland swamps that affects persistence of the characteristic native biota. Fire regimes that promote development and persistence of dense overstorey strata result in declines in diversity of understorey flora, especially woody resprouters that are intolerant of shade and have low rates of growth, fecundity and recruitment (Keith & Bradstock 1994; Keith et al. 2007). The severity and extent of declines in woody resprouters was assessed using data from Keith et al. (2007) and Keith (unpubl. data), on the summed abundance of woody resprouters at 53 sites in 1983 and 2009. During this 26-year period, the summed abundance of woody resprouters declined by a mean of 37% at 72% of sampled sites. These are just below the severity and extent thresholds, respectively, for Vulnerable under criterion D1, assuming that zero abundance of resprouters marks the

point of ecosystem collapse. No data are available prior to 1983, but if current declines were initiated prior to that time, they may exceed the threshold for Vulnerable status. Hence, the status of the community is likely to be Near Threatened (plausible range Near Threatened - Vulnerable) under criterion D1.

Future and historic decline. Insufficient data were available to assess criteria D2 and D3.

Criterion E

No quantitative analysis has been carried out to assess the risk of ecosystem collapse for Coastal sandstone upland swamps. The status of the ecosystem is therefore Data Deficient under criterion E.

REFERENCES

Bailey J. 2010. Groundwater depth and biological interactions in an upland swamp on the Budderoo plateau, NSW. Honours thesis. Macquarie University.

DECCW. 2009. The Native Vegetation of the Sydney Metropolitan Catchment Management Authority Area. Department of Environment and Climate Change NSW, Hurstville.

Gore AJP. 1983. Mires: Swamp, Bog, Fen and Moor. Ecosystems of the World 4A. General Studies. Elsevier, Amsterdam.

Hennessy K, McInnes K, Abbs D, Jones R, Bathols J, Suppiah R, Ricketts J, Rafter T, Collins D, Jones D. 2004. Climate change in New South Wales. Part 2: Projected change in climate extremes. CSIRO, Melbourne.

Keith DA. 1994. Floristics, structure and diversity of natural vegetation in the O'Hares Creek catchment, south of Sydney. Cunninghamia 3: 543-594.

Keith DA Bradstock RA. 1994. Fire and competition in Australian heath: a conceptual model and field investigations. Journal of Vegetation Science 5: 347-354.

Keith DA, Myerscough PJ. 1993. Floristics and soil relations of upland swamp vegetation near Sydney. Australian Journal of Ecology 18: 325-344.

Keith DA, Elith J, Simpson CC. 2011. The response of coastal upland swamp habitat to climate change. Report to NSW Scientific Committee, Sydney.

Keith DA, Holman L, Rodoreda S, Lemmon J, Bedward M. 2007. Plant Functional Types can predict decade-scale changes in fire-prone vegetation. Journal of Ecology 95: 1324-1337.

Keith DA, Rodoreda S, Bedward M. 2010a. Decadal change in wetland-woodland boundaries during the late 20th century reflects climatic trends. Global Change Biology 16: 2300-2306.

Keith DA, Rodoreda S, Holman L, Lemmon J. 2006. Monitoring change in upland swamps in Sydney's water catchments: the roles of fire and rain. Department of Environment and Conservation, Sydney.

Krogh M. 2007. Management of longwall coal mining impacts in Sydney's southern drinking water catchments. Australasian Journal of Environmental Management 14: 155-165.

Lucas C, Hennessy K, Mills G, Bathols J. 2007. Bushfire weater in southeast Australia: recent trends and projected climate change impacts. CSIRO, Melbourne.

NPWS. 2000. Vegetation Survey, Classification and Mapping: Lower Hunter and Central Coast Region. Version 1.2. National Parks and Wildlife Service of NSW. Sydney.

NSW Scientific Committee. 2011. Final Determination to list Coastal upland swamp as an Endangered Ecological Community under the Threatened Species Conservation Act 1995. Government of New South Wales, Sydney.

Tozer MG, Turner K, Keith DA, Tindall D, Pennay C, Simpson C, MacKenzie B, Beukers P. 2010. Native vegetation of southeast NSW: a revised classification and map for the coast and eastern tablelands. Cunninghamia 11: 359-406.

Young, ARM. 1982. Upland Swamps. Dells. on the Woronora Plateau, NSW. PhD thesis, University of Wollongong, NSW.

Young, ARM. 1986. The geomorphic development of dells. upland swamps. on the Woronora plateau, N.S.W., Australia. Zeitschrift fur Geomorphologie 30: 317–327.

2 RAISED BOGS OF GERMANY (NATIONAL ASSESSMENT)

Contributed by Uwe Riecken, Peter Finck & Ulrike Raths, German Federal Agency for Nature Conservation, Department on Biotope Protection and Landscape Ecology, Konstantinstr. 110, 53179 Bonn, Germany.

BACKGROUND TO NATIONAL RED LIST OF HABITAT TYPES IN GERMANY

In 1994 the first edition of the German Red List on Threatened Ecosystems was published (Riecken et al. 1994) based on a first proposal for categories and criteria (Blab et al. 1995). Twelve years later a second edition was completed (Riecken et al. 2006). This Red List is based on a hierarchical classification system of habitat types in Germany which covers 100 % of the surface including freshwater and marine ecosystems. Altogether 690 different habitat types have been identified (764, respectively, if 'technical' habitat types like streets, buildings etc. are included). 590 types (72.5 %) are actually threatened in one of the categories CR to VU and two types have already become extinct. The evaluation is based on two criteria: 'historical decline of area' and 'decline of quality' looking back 150 - 200 years. Both criteria are qualitatively defined only. With regard to 'decline of area' there is nevertheless a good correlation to the quantitative criteria for historical decline presented in this paper. The degree of threat is also assessed on higher classification levels (Riecken et al. 2006, in German); for the later assessment an English version is available (Riecken et al. 2009). In this case study we have tried to apply the criteria from the IUCN Red list of Threatened Ecosystems as proposed in this paper to the national situation in Germany as far as the required data were available. From our list of habitat types we have chosen the type '36.01 raised bogs' which represents the second level of classification.

CLASSIFICATION

National and International: The German habitat type '36.01 raised bogs' corresponds to habitat type *7110 (active raised bogs) of Annex 1 of the European Habitats Directive.
IUCN Habitats Classification Scheme (Version 3.0): 5. Wetlands (inland) / 5.4 Bogs, Marshes, Swamps, Fens, Peatlands.

ECOSYSTEM DESCRIPTION

Characteristic native biota

Raised bogs (Fig. 1) are characterised by a very special vegetation dominated by peat mosses (e.g. *Sphagnum magellanicum, Sphagnum fuscum*) and insectivorous plants like sundew (*Drosera* sp.). Other typical species for raised bogs in Germany are the vascular plants Bog-rosemary (*Andromeda polifolia*) and Cranberry (*Vaccinium oxycoccos*), the butterfly species *Boloria aquilonaris* (Cranberry Fritillary), the moth *Carsia sororiata* (Manchester Treble-Bar) and the ground beetle *Agonum ericeti*. Raised bogs are also very poor in nutrients. The few nutrients naturally available are absorbed by the peat mosses in exchange for humic acids resulting in a very low pH-level. Some plants like sundew species (*Drosera* spp.) rely on catching insects to collect the nutrients they need to exist.

Abiotic environment and Distribution

Raised bogs used to have a wide distribution in the north western and the pre-alpine regions of Germany in the past. Raised bogs are the final ecosystem developing in a series of succession starting from lakes, with various intermediate states of silting up. Typically they have organic soils consisting of the decomposition products of peat mosses (genus *Sphagnum*) which have been conserved through the centuries due to very specific hydrological, anaerobic and acid conditions. Their hydrological regime is depending on rain water only with no

contact to the ground water. Therefore the distribution of raised bogs in Germany is limited to areas with at least 800 mm of rainfall per year.

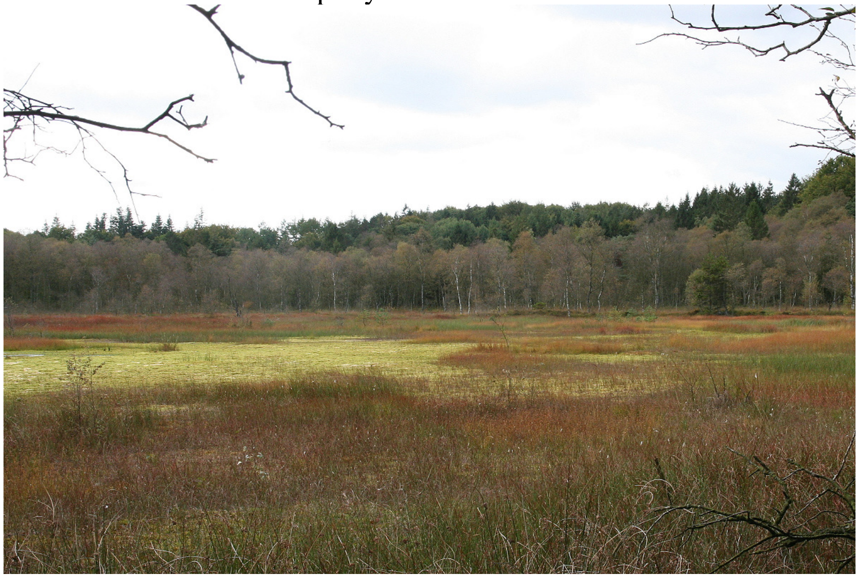

Figure S2. 1. Typical raised bog in northern Germany (Budschimoor, Schleswig-Holstein).

Threatening processes

Raised bogs have been very unattractive for people for centuries because in a natural state they are regularly very difficult to access and for a long time were not useful for any kind of agricultural utilization. The situation changed at the beginning of the industrialisation during the 18th century. Since most forests had already been destroyed at that time, peat became the most important energy source especially in the north western parts of Germany but also in other places in Europe. At that time some smaller peat extractions were already operating mostly at the edges of raised bogs which often covered several hundreds of square kilometres each. This situation changed dramatically: Organised by big companies or regional governments a special type of settlement was developed and installed at nearly all large bog ecosystems in Germany. In a first step channels were built to be used for transportation, especially of the peat. In a second step small houses were built on both sides of the channels and exploration started. The bogs were drained and all the peat was cut, dried and shipped. The peat was used as a surrogate for fire wood in houses but also in industrial production. Later mineral coal became more important but peat still remained a source for heating houses. In the 19th and 20th century a growing demand for peat as planting substrate for private and market gardens sustained this destructive use of raised bogs until today (Berg 2004).

When raised bogs are drained a lot of nutrients become available. As a result higher plant species like grasses, scrubs and trees, which are not able to survive in undisturbed bogs, are able to colonize these drained areas. Most of them are much taller than sphagnum mosses and therefore can successfully compete for light. Especially trees like birch (*Betula pubescens*) have a much higher rate of evapotranspiration than sphagnum mosses. This results in additional draining (Succow & Joosten 2001). After the exploitation of most of the peat, agricultural utilization (life stock farming and arable land) followed and finally spread into most bog areas that had not been disturbed by then. To enable this, further draining was necessary. In all drained peat soils the process of peat degradation starts with huge emission of CO_2 and nutrients (Freibauer et al. 2009). Agricultural utilisation of former bogs is still proceeding and has increased in intensity during the last decades.

Ecosystem collapse

For assessment of criteria A and B, Raised bog ecosystems were assumed to collapse when their mapped distribution declines to zero. Reductions in mapped distribution occur when bogs are drained and replaced by agriculture, or when their native vegetation is replaced by non-bog species such as grasses, scrubs and trees, which outcompete typical bog species for light and result in further drying. For assessment of criterion C, nutrient enrichment ia a key process of environmental degradation, and collapse was assumed to occur when the nutrient loads in bog soils reach 'critical levels' at which significant harmful effects on the raised bogs occur.

ASSESSMENT

This assessment applies to the distribution of raised bogs only within Germany. Their distribution extends over a much larger area in Europe (see below) and consequently their status may be different in the global context compared with the German national context assessed here.

Summary

Criterion	A	B	C	D	E	overall
subcriterion 1	EN	NE	VU	NE	DD	CR
subcriterion 2	LC	LC(LC-VU)	DD	NE	-	-
subcriterion 3	CR	NE	CR	NE		

Criterion A

For this case study we have decided to use the change in occupied area in absolute numbers (km^2), as we have done in the German Red List. The data are based on habitat mappings conducted on behalf of the nature conservation authorities of the German Federal States. The total area of raised bogs for 2007 has been reported to the European Commission in the context of the German national report on implementation measures (Article 17, Habitats Directive) (BfN 2007). The actual remaining distribution of the habitat type ‘raised bogs’ in Germany is shown in Fig. 2.

<u>Current decline</u>: Today the totals area of raised bogs in Germany in a natural or near natural state covers some 62.4 km^2 (BfN 2007). The respective area that still existed 50 years ago is difficult to estimate exactly, but we calculate it to be at least 150 km^2. According to mapping data from the nature conservation authority of the Federal State of Lower Saxony (one of the main areas of distribution of raised bogs in Germany) the area of natural raised bogs covered about 20 km^2 in Lower Saxony in 1984 (Drachenfels et al. 1984). This area had been reduced to roughly 10 km^2 by 2006 (BfN 2007). The resulting estimation of a decline in occupied area within the last 50 years of 58 % is substantially more than 50 % and thus leads to the category **Endangered** under criterion A1.

<u>Future decline</u>: Due to a lot of practical and legal measures to regenerate and protect raised bogs in the past years (e.g., Riecken 2002, Ssymank et al. 1998) we expect, that there will be no more reductions or even a smaller increase in the spatial extend of raised bogs. Hence,this ecosystem would not be listed as threatened under criterion A2 (i.e. **Least Concern**).

<u>Historic decline</u>: In 1750 most raised bogs in Germany were still mainly intact and human impact was restricted to relatively small areas along their edges. The total area covered by bogs at that time is estimated to have been around 3,360 km^2 (Succow & Joosten 2001).

Hence, since that time the occupied area of raised bogs has been reduced by more than 98 %. Raised bogs therefore are categorized in criterion A3 as **Critically Endangered**.

Criterion B

It is difficult to apply this criterion nationally because the thresholds given for B1 and B2 are developed for assessment of the global geographical distribution and area of occurrence of an ecosystem, respectively. In this case study we only looked at B2. Applying a grid with cell area of 128 km^2 (Fig. 2) to raised bogs in Germany (only 62.4 km^2 remaining) produces an estimate of just over 200 occupied grid cells. There are continuing declines and serious plausible threats to the ecosystem (subcriteria a and b, respectively). If less than 50 of the 200 cells are occupied by more than 1 km^2 of raised bog, the ecosystem may be categorized as **Vulnerable** under criteria B2a and B2b, or otherwise as Least Concern.
For comparison, the distribution of raised bogs across Europe is much larger than than the distribution within Germany (Fig. 3). Thus at the level of Europe the ecosystem would be categorized as **Least Concern** under criterion B

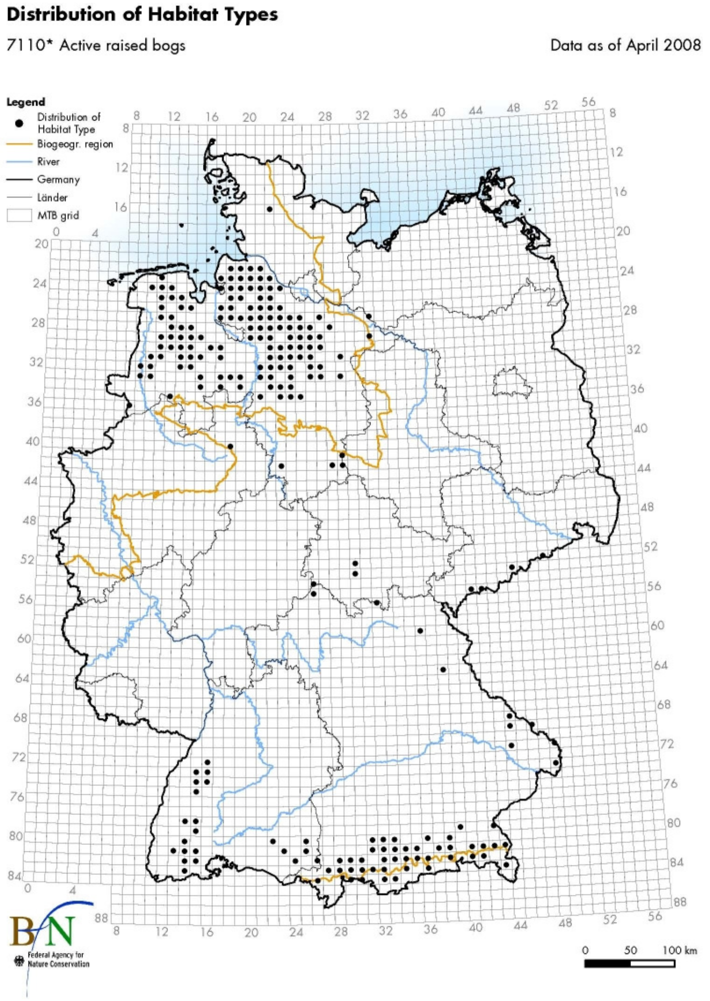

Figure S2. 2. Remaining distribution of ‘raised bogs’ in Germany based on a geographical grid (MTB grid) with an approximate area of 128 km^2 for each grid cell (1/10° of geographical longitude, 1/6° of geographical latitude). Cells occupied by raised bogs are marked.

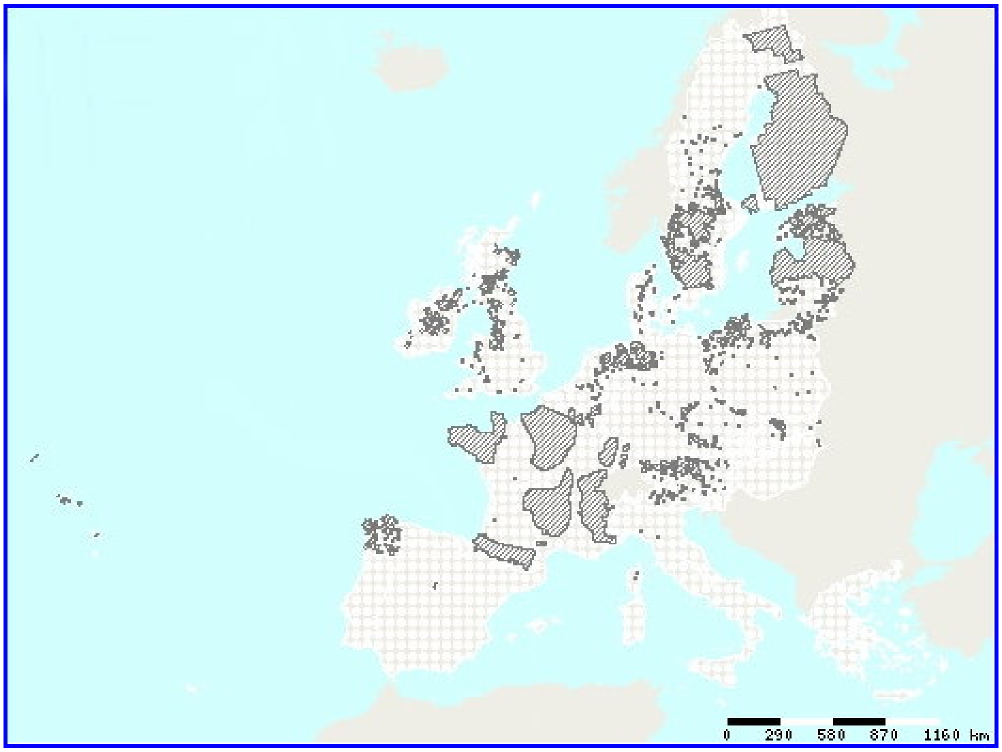

Figure S2. 3. Distribution of raised bogs in the EU based on geographical grids. Grid cells with an occurrence of raised bogs are marked (Eionet 2011). Grid sizes vary between the countries.

Criterion C

It is difficult to differentiate this criterion from the decline in occurrence. As we pointed out earlier the destruction of raised bogs always commenced with severe changes in ecological functions mainly caused by draining. Additionally all raised bogs in Germany are subject to nutrient leakage from intensive agricultural use in their vicinity as well as aerial deposition of nutrients. Raised bogs are naturally poor in nutrients and in more than 70 % of the area of sensible habitats in Germany the critical loads of nitrogen were still exceeded in 2007, coming down from almost 100 % of the corresponding area in 1990 (UBA 2011). This is especially true for the north-western part of the country, one of the main areas of the original distribution for raised bogs. If the nutrient input exceeds the ‘critical loads’, significant harmful effects on the raised bogs occur. The sphagnum mosses are no longer able to use all available nutrients. Especially if additional draining occurs, raised bogs become more and more suitable for higher plants like grasses, scrubs and trees which are better competitors for light and also add to the draining due to their high rate of evapotranspiration (Bobbink et al. 2001, 71ff, Succow & Joosten 2001: 462ff).

<u>Current decline</u>: It is clear from the above data that more than 80 % of the area of occupancy of raised bogs are facing a reduction in ecological quality during the last 50 years caused by nutrient input and by ongoing draining. To assess relative severity of environmental degradation, we assume that critical nitrogen loads represent a decline in environmental quality of at least 50 %, and that most bogs had loads well below critical levels 50 years ago. Summarized, declines of > 50 % relative severity across > 70 % of the extent would result in category **Vulnerable** under criterion C1.

Future decline: Nobody knows exactly what effects the ongoing impacts of nutrients will have on raised bogs in the next 50 years. We therefore assign the ecosystem to category **Data Deficient** under criterion C2.

Historic decline: As pointed out earlier human use and input on raised bogs started with severe impacts in ecological functions. Given that historic nitrogen levels were likely to be well below critical loads (pre-dating use of industrial-scale agricultural fertilizers), and that more than 70 % of bogs are currently above those levels, we assume that since 1750 more than 90 % of all raised bogs have had a decline of ecosystem quality which more than 90 % relative severity resulting in the category **Critically Endangered**.

Criterion D

We are sure that the changes in ecological functions caused by changes in water and nutrient regime are impacting the main biotic interactions to a similar extend as we have estimated under criterion C, however, we have not evaluated data on biotic interactions directly. This would result in category **Not Evaluated** for criterion D.

Criterion E

As we do not really know what will happen to raised bogs in the future, if the nutrient impacts continue or to what extend climate change will change the situation of rainfalls in Germany, we are not able to apply criterion E seriously, which means **Data Deficient** for this criterion.

Overall status

The overall status of raised bogs is estimated as being **Critically Endangered** (criteria A2, C3). This result corresponds exactly to the result of Riecken et al. (2006) who also listed raised bogs in the category **Critically Endangered**.

References

Berg E. 2004. Die Kultivierung der nordwestdeutschen Hochmoore. - Oldenburger Forschungen N.F.20; (Schriftenreihe des Landesmuseums); Isensee Verlag : 199 pp.

Blab J, Riecken U, Ssymank A. 1995. Proposal on a criteria system for a national Red Data Book of Biotopes. Landscape Ecology 10(1): 41-50.

BfN 2007. German national report on implementation measures (Article 17, Habitats Directive). -http://www.bfn.de/0316_bericht2007+M52087573ab0.html (accessed 2011-10-24)

Bobbink R, Ashmore M, Braun S, Flückinger W, Van den Wyngaert IJJ. 2002. Empirical nitrogen critical loads for natural and semi-natural ecosystems: 2002 update http://www.iap.ch/publikationen/nworkshop-background.pdf (accessed 2011-10-25)

Drachenfels OV, Mey H, Miotik P. 1984. Naturschutzatlas Niedersachsen. Erfassung der für den Naturschutz wertvollen Bereiche. Ergebnis der ersten landesweiten Kartierung, Stand. Naturschutz und Landschaftspflege in Niedersachsen 13, 263 pp.

Eionet 2011. ETC/BD biogeographical assessment. European Topic Centre on Biological Diversity - http://bd.eionet.europa.eu/article17/habitatsummary/?group=Ym9ncywgbWlyZXMgJiBmZW5z&habitat=7110&region= (accessed 2011-10-24)

Freibauer A, Droesler M, Gensior A, Schulze ED. 2009. Das Potenzial von Wäldern und Mooren für den Klimaschutz in Deutschland und auf globaler Ebene. Natur und Landschaft 84: 20-25.

Riecken U, Ries U, Ssymank A. 1994. Rote Liste der gefährdeten Biotoptypen der Bundesrepublik Deutschland. Kilda Verlag, Greven, 184 pp. (= Schr.R. f. Landschaftspfl. u. Natursch. 41).

Riecken U. 2002. Novellierung des Bundesnaturschutzgesetzes: Gesetzlich geschützte Biotope nach Paragraph 30. Natur und Landschaft 77: 397-406.

Riecken U, Finck P, Raths U, Schröder E,Ssymank A. 2006. Rote Liste der gefährdeten Biotoptypen Deutschlands. Zweite fortgeschriebene Fassung 2006. Natursch. Biol. Vielf. 34, 318 pp.

Riecken U, Finck P, Raths U, Schröder E, Ssymank A. 2009. German Red Data Book on endangered habitats (short version, July 2009). http://www.bfn.de/fileadmin/MDB/documents/themen/landschaftsundbiotopschutz/Red_Data_Book_Habiatats_krz.pdf (accessed 2011-10-28)

Ssymank, A., Hauke, U., Rückriem, C. & Schröder, E. unter Mitarbeit von Messer, D. (1998): Das europäische Schutzgebietssystem NATURA 2000 - BfN-Handbuch zur Umsetzung der Fauna-Flora-Habitat-Richtlinie (92/43/EWG) und der Vogelschutz-Richtlinie (79/409/EWG). - SchrR. f. Landschaftspfl. u. Natursch. 53, 560 pp.

Succow, M. & Joosten, H. (Hrsg.) (2001): Landschaftsökologische Moorkunde. - Schweizerbart'sche Verlagsbuchhandlung (Nägele & Obermiller), Stuttgart, 622 pp.

UBA (Umweltbundesamt) (2011): Indikator: Überschreitung der Critical Loads für Stickstoff (Eutrophierung), - http://www.umweltbundesamt-daten-zur-umwelt.de/umweltdaten/public/theme.do?nodeIdent=2870 (accessed 2011-10-17)

3 GERMAN TAMARISK-PIONEER VEGETATION (GTPV), EUROPE

Contributed by Franz Essl, Environment Agency Austria, University of Vienna.

CLASSIFICATION

International: Habitat Directive habitats of the Annex I**:** 3230 Alpine rivers and their ligneous vegetation with *Myricaria germanica* (Romao 1996)
Phytosociological units (according to Essl et al. 2002, Willner & Grabherr 2009): Salici-Myricarietum Moor 1958, Myricario-Chondrilletum Br.-Bl. In Volk 1933 pro parte, Epilobio-Myrcarietum Aichinger 1933
IUCN Habitats Classification Scheme: 5. **Wetlands** /5.11 Alpine Wetlands
Biogeographic Realm: Central, Western and Sub-mediterranean Europe
Key references: GTPV is defined as an ecosystem of European community interest in the EU Habitat Directive (Romao 1996). Detailed information on plant species composition, essential ecosystem processes, and ecology are e.g. provided in Müller & Bürger (1990), Romao (1996), Kudrnovsky (2005), Müllller (2005), and Willner & Grabherr (2009).

ECOSYSTEM DESCRIPTION

Characteristic native biota

GTPV is characterized by low to very low vegetation cover composed of herbs and shrubs, large fractions of bare gravel and sandy substrate, and high spatio-temporal heterogeneity between microsites regarding flooding regime, substrate grain size, and vegetation cover. The dominating vascular plant species is the low shrub German tamarisk (*Myricaria germanica*), whose European distribution is closely related to this ecosystem (Figs 1 - 3). *Myricaria* produces light small hairy seeds, which are spread by wind and water, and can readily colonize new sites, provided that sandy, wet microsites are available. Other characteristic plant species predominantly occurring in GTPV are the chamaephytes *Epilobium dodonaei* and *E. fleischeri*, and the grass *Calamagrostis pseudophragmites*. Albeit vegetation cover is low, plant species richness may be high, as many species of adjacent vegetation communities occur in low densities. This mostly includes species of screes, grasslands, ruderal habitats and riverine forests. Often young growth of willows (*Salix purpurea, S. alba, S. eleagnos, S. daphnoides*) is interspersed, which will, in absence of a disturbance event, within several years to few decades transform GTPV into the next successional stage, i.e. willow forest. Across its European range, accompanying species pools vary considerable. A full characteristic plant species list of the Central European range is provided in Ellmauer (2005) and Willner & Grabherr (2009).

Characteristic animal species include a range of taxa largely restricted to this ecosystem. These include beetles (e.g. Carabids: *Cicindela* spp., *Bembidion* spp.) and grasshoppers (e.g. *Bryodema tuberculata, Chorthippus pullus*) (Ellmauer 2005).

Abiotic environment

GTPV colonizes periodically flooded gravel and sand banks, predominantly along braided rivers which transport high sediment loads and show pronounced hydro-morphological dynamics (Billi et al. 1992). Suitable sites are subject to periodically heavy flooding, which relocate the substrate and reset the succession to vegetation free bare gravel and sand banks.

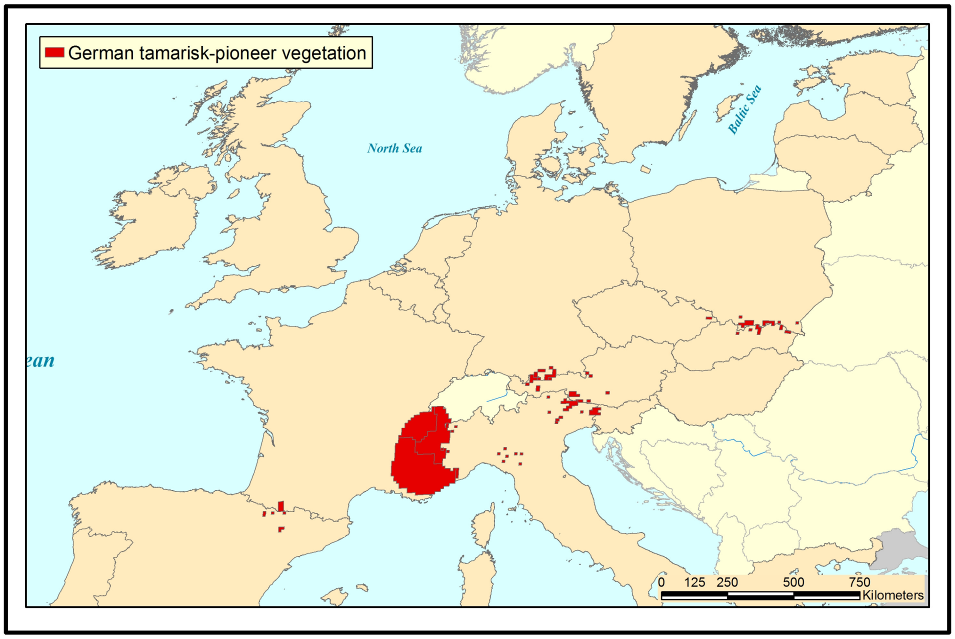

Figure S3. 1. Distribution of German tamarisk-pioneer vegetation (EEA 2009) within the conservation areas nominated for the Habitats Directive within the member states of the European Union. Note, that occurrences outside conservation areas nominated for the Habitats Directive are not shown. Outside the European Union, this ecosystem also occurrs in South-eastern Europe and Switzerland (distribution not shown).

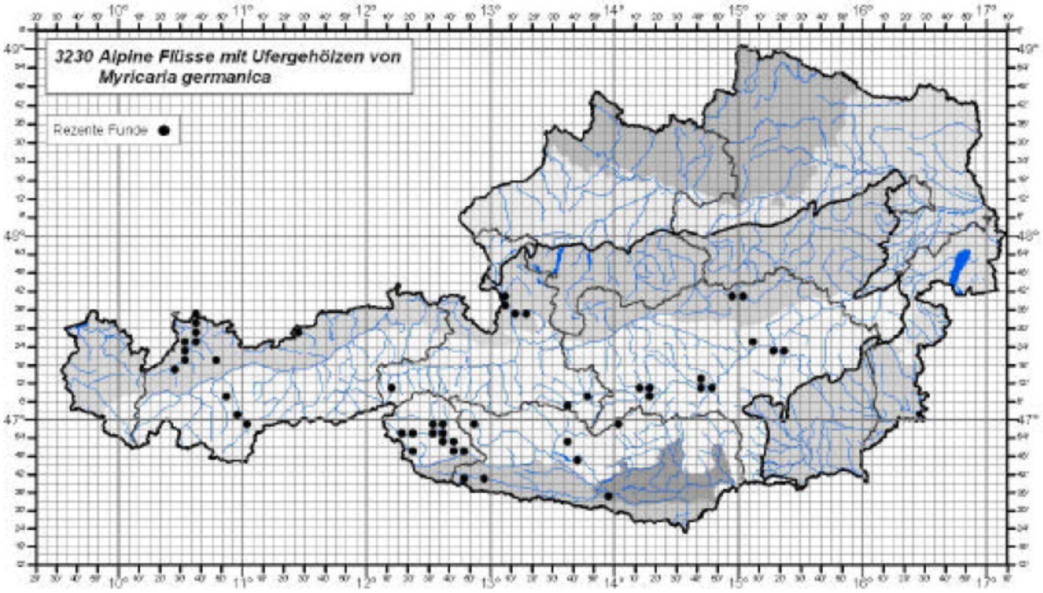

Figure S3. 2. Example of the current national distribution of the German tamarisk-pioneer vegetation within a country in the centre of its range (Austria, Central Europe). Distribution is presented as a grid-distribution map (3x5 geogr. minutes = ca. 35 km^2). Former occurrences, which have been lost before ca. 1990, are not shown in the map (Ellmauer 2005).

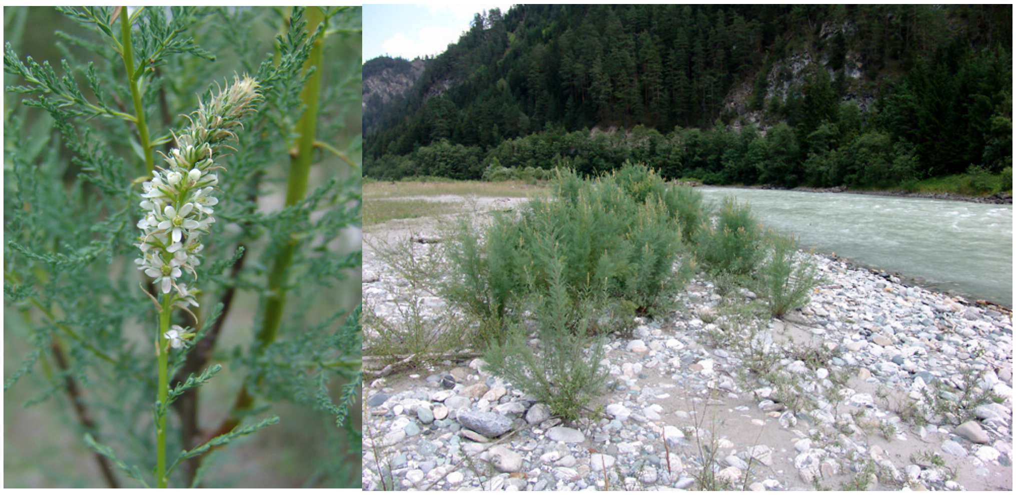

Figure S3. 3. An inflorescence of *Myricaria germanica* (left) and a patch of German tamarisk-pioneer vegetation (right) on a dynamic gravel bank along the Inn river in Tyrol (Austria). Grey-green *Myricaria germanica* shrubs dominate the vegetation. (c) Wikimedia commons.

Distribution

GTPV is restricted to mountain ranges and their foothills, i.e the Alps, Carpathians, Balkan Mountain ranges, and the Pyrenees (Fig. S3.1). It colonizes riverine habitats ranging from small streams to large rivers, with the largest stands being located in braided river systems, which can be > 1 km wide in places (e.g. rivers Piave and Tagliamento in northern Italy, Müller 2005). The total mapped area of GTPV within the European Union (excluding south-eastern Europe) is estimated to be ca. 60 km^2, with the largest occurrences in France and northern Italy (> 50 km^2). In all other countries the total mapped distribution never exceeds 1 km^2 (EEA 2009). For south-eastern Europe, no comprehensive quantitative data are available. However, there are significant occurrences in the Carpathians (Romania, western Ukraine), as well as on the Balkan Peninsula (e.g. Bulgaria, Albania). Based on available data, the combined mappable area of GTPV there is cautiously estimated to be 10-30 km^2 (Table 1). GTPV has lost parts of its former range, e.g. it is now absent from the Danube and most sections of the Rhine, where it still covered a significant fraction of riverine habitats in the mid-19th century.

Threatening Processes

Most of GTPV has been lost due to wide-spread alterations of hydro-morphological processes of running waters in Europe. From the mid-19t^{h} to the mid-20th centuries, losses were mainly driven by the effects of river channelization. In the last decades, the construction of hydro-electric power plants has emerged as a second main threatening factor. Both threats alter vital hydro-morphological processes, reduce river gravel dynamics and affect flood pulses, all being essential features for this ecosystem to develop and persist. Under altered, particularly reduced, floods and sediment loads, more competitive, but less disturbance-tolerant species of successional serial ecosystems (e.g. willow species) are able to invade and outcompete tamarisk and its allies. Recently, regional distribution has been stabilized or even moderately increased due to nature conservation activities.

GTPV is listed in the Annex I of the Habitats Directive (Romao 1996), hence EU member states are obliged to protect this ecosystem by establishing conservation areas and manage existing occurrences appropriately. Available data suggest that more than 50% of existing

stands are located in nature conservation areas. Since listing in the Habitats Directive, many European countries have increased the level of legal protection for this ecosystem.

Ecosystem collapse

For assessment of criteria A and B, we assumed GTPV to have collapsed when the mapped distribution declines to zero, either as a consequence of channelisation, or when the characteristic native biota including tamarisk is replaced by species such as willow.

ASSESSMENT

Summary

Criterion	A	B	C	D	E	overall
subcriterion 1	EN(VU-EN)	LC	NE	DD	DD	EN
subcriterion 2	DD	EN	NE	DD		
subcriterion 3	EN	LC	NE	DD		

Criterion A

Current decline. Historic maps, habitat mapping data and floristic records of *Myricaria germanica* indicate substantial losses in geographic extent during the last 50 years. In Austria, GTPV has been lost completely or nearly so along a number of rivers (e.g. Gurk, Mur, Salza, Inn, Salzach) during this time period (Petutschnig 1994, Essl et al. 2002, Ellmauer 2005). In Germany, GTPV completely disappeared during the last 50 years from the Lech River, where it formerly had its largest national occurrences (Müller & Bürger 1990). In Croatia and Slovenia, the significant occurrences along the Drava River (Trinajstic 1992) have been nearly totally lost. In northern Italy and southern France, losses were on average less severe (e.g. Müller 2005). Across its range, available data indicate that losses in geographic distribution during the last 50 years probably were over 50%, with the midpoint of estimated area-weighted loss being around 51%, with lower and upper boundaries of 37 to 65% (Table 2). Endangered (plausible range Vulnerable-Endangered)_under criterion A1.

Table S3. 1. Estimated share of global distribution of GTPV in European countries, estimated decline in GTPV distribution per country since 1750 and during the last 50 years.

Country	estimated % of global distribution 50 years ago	estimated decline in distribution over past 50 years	estimated % of global distribution in 1750	estimated decline in distribution since 1750	Source
Albania	3	20-40	2	30-50	Horvath et al. (1998), A. Mohl ined.
Austria	5	40-70	13	90-98	Essl et al. (2002), Ellmauer et al. (2005), Kudrnovsky (2005)
Bulgaria	2	40-70	1	70-90	Horvath et al. (1998), A. Mohl ined.
Croatia	2	60-80	2	90-98	Trinajstic (1992), A. Mohl ined.
France	28	40-70	20	70-90	Martinet & Dubost (1992), Pautou et al. (1997), EEA (2009)

Country	estimated % of global distribution 50 years ago	estimated decline in distribution over past 50 years	estimated % of global distribution in 1750	estimated decline in distribution since 1750	Source
Germany	3	40-70	12	90-98	Müller (1995), Riecken et al. (2006), Lang & Walentowski (2010)
Italy	30	40-70	23	60-80	Müller & Bürger (1990), EEA (2009)
Poland	2	40-70	2	?	EEA (2009)
Romania	12	20-40	10	50-70	A. Mohl (ined.)
Serbia & Montenegro	2	?	2	80-90	Horvath et al. (1998)
Slovakia	3	?	3	90-98	EEA (2009)
Slovenia	2	60-80	2	90-98	N. Jogan (pers. comm.)
Spain	1	?	1	?	EEA (2009)
Switzerland	2	40-70	5	90-98	Martinet & Dubost (1992), Gallandat et al. (1993), Lachat et al. (2010)
Ukraine	3	20-40	2	50-70	F. Essl (ined.), A. Mohl (ined.)

Table S3. 2. Estimates of minimum, average and maximum rates of decline of GTPV during the last 50 years and since 1750, shown for individual countries and as area-weighted total for the total range. Baseline figures have been calculated from Table 1.

	**national declines (last 50 yrs)**			**contribution to global decline**			**national declines (> 1750)**			**contribution to global decline**		
Country	lower bound	mid point	upper bound	lower bound	mid point	upper bound	lower bound	mid point	upper bound	lower bound	mid point	upper bound
Albania	20	30	40	0.6	1.0	1.3	30	40	50	0.6	0.8	1.0
Austria	40	55	70	2.1	2.9	3.7	90	94	98	12.1	12.6	13.1
Bulgaria	40	55	70	0.9	1.2	1.5	70	80	90	0.7	0.8	0.9
Croatia	60	70	80	1.3	1.5	1.7	90	94	98	1.9	1.9	2.0
France	40	55	70	11.9	16.4	20.9	70	80	90	14.4	16.5	18.6
Germany	40	55	70	1.3	1.8	2.2	90	94	98	11.1	11.6	12.1
Italy	40	55	70	12.8	17.6	22.3	60	70	80	14.2	16.6	19.0
Poland	40	55	70	0.9	1.2	1.5	?	?	?	?	?	?
Romania	20	30	40	2.6	3.8	5.1	50	60	70	5.2	6.2	7.2
Serbia & Montenegro	?	?	?	?	?	?	90	94	98	1.9	1.9	2.0
Slovakia	?	?	?	?	?	?	90	94	98	2.8	2.9	3.0
Slovenia	60	70	80	1.3	1.5	1.7	90	94	98	1.9	1.9	2.0
Spain	?	?	?	?	?	?	?	?	?	?	?	?
Switzerland	40	55	70	0.9	1.2	1.5	90	94	98	4.6	4.8	5.1
Ukraine	20	30	40	0.6	1.0	1.3	50	60	70	1.0	1.2	1.4
**total decline (%)**				**37.0**	**50.9**	**64.7**				**72.4**	**80.0**	**87.5**

Future decline: No projections of future distributions exist. Data Deficient under criterion A2.

Historic declines. Comparisons of current distribution with historic maps and floristic records of *Myricaria germanica* from the mid-18th century (before large-scale river channelization started in Europe) indicate average losses of over 90% within the last 250 years. Several river systems (e.g. Danube, large sections of Rhone, Rhine, Lech, Inn), where some of the largest occurrences had been, have totally lost GTPV occurrences, particularly since the mid-19th century (e.g. Müller & Bürger 1990, Essl et al. 2002). In several countries (e.g. Austria, Croatia, Germany, Slovenia, Switzerland), losses are highly probably > 95% of original AOO. In south-eastern Europe, the Balkan Peninsula, in Italy and France losses have been less severe and are estimated to be on average at about 70% (Table 1). Area-weighted estimates of total decline of GTPV since 1750 indicate that losses of AOO have been around 80%, with lower and upper boundaries of 72 to 88% (Table 2). Endangered under Criterion A3.

Criterion B

Extent of Occurrence. A minimum convex polygon enclosing all occurrences of GTPV exceeds 100,000 km^2, and hence the ecosystem does not qualify for a threat category under the criterion B1., i.e. Least Concern.

Area of Occupancy. Based on near-European wide data from the Habitats Directive (EEA 2009), GTPV is still present in more than 100 grid cells (10 x 10 km). However, in the majority of these cells, the mapped area of GTPV does not exceed 1 km^2 (1% of cell area). In Austria, for example, GTPV currently occupies approximately 31 10 x 10 km cells, yet it is unlikely that any of these cells contain more than 1 km^2 of the ecosystem. In Italy and France, it is estimated that 6 - 10 cells include more than 1 km^2 of GTPV, while Romania may have a further three. Therefore GTPV may occupy 6 - 13 10 x 10 km grid cells across the total distribution of the ecosystem, excluding those with less than 1% occupancy. Continuing declines of GTPV are occurring in many river systems (criterion B2a) due to the joint effects of river channelization and hydroelectric power plant construction on vital hydro-morphological processes, which may also affect occurrences downriver (criterion B2b). Elsewhere, declines have been recently stopped or even reversed due to river restoration projects (e.g. rivers Lech and Drau in Austria). Based on the number of occupied grid cells continuing declines and future threats, GTPV is Endangered under criterion B2.

Locations. GTPV occurs in several main river catchments across a relatively large geographic range. As threats are hydrologically based there are more than 10 locations and GTPV is Least Concern under criterion B3.

Criterion C

There is evidence of widespread and severe alterations in gravel sedimentation and erosion regimes, in flood pulses and discharge volumes (Lehner et al. 2011). Further, there are indications that water eutrophication and pollution may affect substrate quality. It is likely that these modifications affect more than 50% of current occurrences, but the scale and impact is insufficiently analysed for assessing this criterion, hence the Not Evaluated category is appropriate.

Criterion D

There is some evidence of recent increases in invasive alien woody plants (e.g. *Amorpha fruticosa, Buddleja davidii*, e.g. Müller 2005) in GTPV in occurrences located in warm-temperate to sub-mediterranean climates. This may negatively affect characteristic biota due to increased competition and changed vegetation structure. However, the scale and impact of this phenomenon is currently poorly understood, and TPV is assigned to Data Deficient under criterion D.

Criterion E

No modelling of risks has been carried out, hence GTPV is Data Deficient under criterion E.

REFERENCES

Billi P, Hey RD, Thorne CR & Tacconi P. 1992. Dynamics of gravel-bed rivers. Chichester: Wiley.

EEA. 2009. Habitats Directive Article 17 Reporting. http://eea.eionet.europa.eu/Public/irc/eionet-circle/habitats-art17report/library?l=/datasheets/habitats. accessed 2011-10-14. .

Egger G & Theiss M. 2000. Typisierung der Auen Österreichs. Literaturauswertung der auenspezifischen Pflanzengesellschaften österreichischer Fließgewässer. Unveröffentlichte Studie, Institut für Ökologie und Umweltplanung, Klagenfurt.

Ellmauer T. 2005. Entwicklung von Kriterien, Indikatoren und Schwellenwerten zur Beurteilung des Erhaltungszustandes der Natura 200-Schutzgüter. Band 3: Lebensraumtypen des Anhangs I der Fauna-Flora-Habitat-Richtlinie. http://www.umweltbundesamt.at/fileadmin/site/umweltthemen/naturschutz/Berichte_GEZ/Band_3_FFH-Lebensraumtypen.pdf. accessed 2011-10-14. .

Essl F, Egger G., Ellmauer T & Aigner S. 2002. : Rote Liste gefährdeter Biotoptypen Österreichs. Wälder, Forste, Vorwälder. Umweltbundesamt, Wien.

Gallandat JD, Gobat JM & Roulier C. 1993. Kartierung der Auengebiete von nationaler Bedeutung. Schriftenreihe Umwelt 199, Bundesamt für Umwelt, Wald und Landschaft, Bern.

Horvat I, Glavac V, Ellenberg H. 1998. Vegetation Südosteuropas. Urban & Fischer, München.

Kudrnovsky H. 2005. Die Deutsche Tamariske. *Myricaria germanica*. und ihre FFH-Ausweisung in Österreich. http://www.wasser-osttirol.at/media/studie_tamariske.pdf. (accessed 2011-10-14).

Lachat T, Pauli D, Gonseth Y, Klaus G, Scheidegger C, Vittoz P, Walter T. 2010. Wandel der Biodiversität in der Schweiz seit 1900. Haupt Verlag, Bern.

Lang A, Walentowski H. 2010. Handbuch der Lebensraumtypen nach Anhang I der Fauna-Flora-Habitat-Richtlinie in Bayern. Bayerisches Landesamt für Umwelt, Augsburg.

Lehner B, Liermann C, Revenga C, Vörösmarty C, Fekete B, Crouzet P, Döll P, Endejan M, Frenken K, Magome J, Nilsson C, Robertson JC, Rödel R, Sindorf N, Wisser D. 2011. High resolution mapping of the world's reservoirs and dams for sustainable river flow management. Frontiers in Ecology and the Environment. doi:10.1890/100125.

Martinet F, Dubost M. 1992. : Die letzten natumahen Alpenflusse. CIPRA Kleine Schriften 11/92: 6-60.

Müller N. 1995. Wandel von Flora und Vegetation nordalpiner Wildflusslandschaften unter dem Einfluss des Menschen. Berichte der ANL 19: 125-187.

Müller N. 2005. Die herausragende Stellung des Tagliamento. Friaul, Italien. im Europäischen Schutzgebietssystem NATURA 2000. Jahrbuch des Vereins zum Schutze der Bergwelt 70: 19-35.

Müller N, Bürger A. 1990. Flußbettmorphologie und Auenvegetation des oberen Lech im Bereich der Forchacher Wildflußlandschaft. Oberes Lechtal, Tirol. . Jahrbuch des Vereins zum Schutze der Bergwelt 55: 43-74.

Pautou G, Peiry JL, Girel J, Blanchard E, Hughes F, Richards K, Harris T, El-Hames A. 1997. Space-time units in floodplains: the example of the Drac River upstram of Grenoble. Global Ecology and Biogeography Letters 6: 311-319.

Petutschnig W. 1994. Die Deutsche Tamariske in Kärnten. Carinthia II 184/104: 19-30.

Riecken U, Finck P, Raths U, Schröder E, Ssymank A. 2006. Rote Liste der gefährdeten Biotoptypen Deutschlands – zweite fortgeschriebene Fassung 2006. Naturschutz und Biologische Vielfalt 34, 318 pp.

Romao C. 1996. Interpretation manual of European Union habitats. European Commission DG XI, Brussels.

Trinajstic I. 1992. Salici-Myricarietum Moor 1958. Salicion eleagni. in the vegetation of Croatia. Thaiszia 2: 67-74.

Willner W, Grabherr G. 2009. Die Gebüsche und Wälder Österreichs. Spektrum Verlag, Heidelberg.

4 SWAMPS, MARSHES AND LAKES IN THE MURRAY-DARLING BASIN

Contributed by Richard T. Kingsford, Australian Wetlands and Rivers Centre, University of NSW, NSW 2052

CLASSIFICATION

National/State: Combines two vegetation classes in New South Wales - Inland Floodplain Swamps and Inland Floodplain Shrublands (Keith 2004).
IUCN Habitats Classification Scheme: 5. **Wetlands** / 5.5 Permanent Freshwater Lakes / 5.6 Seasonal/Intermittent Freshwater Lakes
Biogeographic realm: Australasia

ECOSYSTEM DESCRIPTION

Characteristic native biota

Swamps, marshes and lakes in the Murray-Darling Basin comprise spatially complex and dynamic mosaics of flood dependent vegetation communities. These include aquatic macrophyte assemblages, open water lakes, swamps, herbfields, reedbeds and shrublands, whose distribution is structured by inundation regimes, which are in turn driven by river flow behaviour (Brock *et al.* 2006). These wetlands are interspersed with floodplain eucalypt forests and woodlands (see case studies on River Red Gum forests, Coolibah - Black Box Woodlands).

Characteristic plant species in the wetlands include aquatic macrophytes, reeds, grasses and shrubs that are all flood dependent. For example, one of the large marshes, the Macquarie Marshes, consists of a mosaic of different vegetation types, including aquatic macrophytes (e.g. *Ruppia* spp., *Lepilaena* spp., *Lamprothamnium* spp., *Vallisneria australis*, *Myriophylium* spp. *Marsilea villosa*), extensive swards of water couch *Paspalum distichum*, common reed *Phragmites australis*, and lignum *Muelenbeckia florulenta.*

Such wetlands provide habitat for a wide range of other biota, including algae (Bunn *et al.* 2006), invertebrates (Boulton *et al.* 2006) and vertebrates (Kingsford *et al.* 2006). Waterbirds are a functionally important component of the biota, that characteristically depend on the wetland habitats. Waterbird assemblages often comprises more than thirty species for an individual wetland at any one time and can be usefully organised into functional groups of species based on diet and habitat use, including ducks, herbivores, large wading birds, piscivores and small waders (Kingsford and Porter 1994).

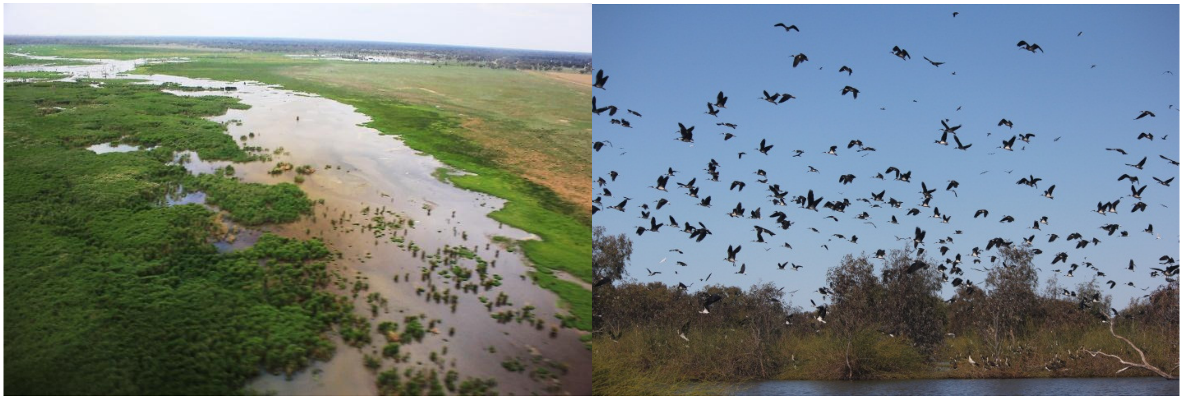

Figure S4. 1. Extensive marsh and swamp wetlands such as the Macquarie Marshes in the Murray-Darling Basin comprise complex and dynamic mosaics of vegetation (left) that support high concentrations of waterbirds such as large wading birds (e.g. straw-necked ibis *Threskiornis spinicollis*, right) which breed in colonies of tens of thousands of pairs, after widespread inundation.

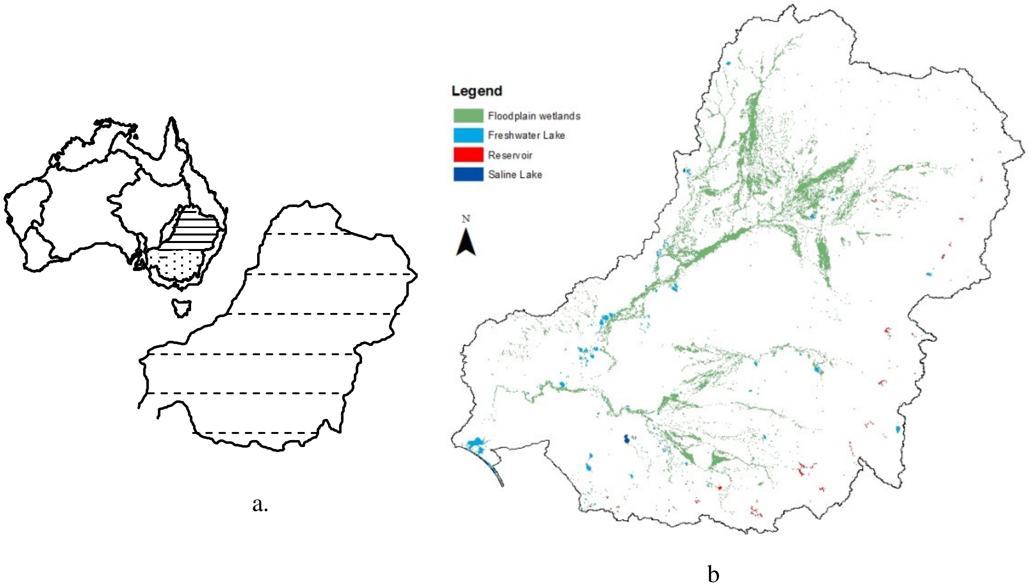

Figure S4. 2. a) Location of the Murray-Darling Basin, among 12 large river basins across Australia, showing the northern Darling basin (hatched) and the southern Murray basin (stippled) and six aerial survey bands (30 km wide) which form the basis of data collected for waterbirds. b) Distribution of four broad wetlands types in the Murray-Darling Basin, supplied by river flows from the 19 major river catchments.

Abiotic environment

The Murray-Darling Basin is dominated by semi-arid and arid climates (rainfall <500mm per year) with more mesic climates in the eastern regions (rainfall 800-1,000 mm per year). Of the 500,000 GL of rainfall which falls each year, only about 6% (31,800 GL) naturally flows into the rivers of the basin each year on average, although this is highly variable (range 6,740 GL (2006)-117,907 GL 1956); the rest evaporates or flows into groundwater ecosystems (MDBA 2010). Diversions from the Snowy and Glenelg Rivers have boosted inflow by 997 GL per year (MDBA 2010).

Within the Basin, there are 19 major rivers (MDBA 2010) which flow into large marshes, swamps and lakes (Kingsford *et al.* 2004). The rivers separate into those that mainly flow from east to west in the Murray catchment and those that flow east to west or north to south in the Darling catchment (Figure 2b). Most of the water flows down the rivers of the Murray catchment with many of the rivers of the Darling catchment flooding large wetland ecosystems. The two catchments also differ in flow seasonality. The southern Murray catchment primarily has a spring-dominated flow regime generated as rainfall and snow on the Great Dividing Range, which forms its eastern watershed. Snow falling in winter (June-August) melts in the subsequent spring months and begins to flow down the rivers. In contrast, the rivers of the Darling catchment in the northeast, are influenced by northern tropical weather patterns, with maximum flows typically during the summer months (December-February), coinciding with the wet season in northern Australia. The southern rivers in the Darling River catchment are also influenced by winter spring rainfall that dominates climatic patterns of southeastern Australia.

Generally, marshes, swamps and lakes in the Murray-Darling Basin can be distinguished by having reasonably frequent extensive flooding regimes, with inundation occurring in at least about one in every five years, and water retained for up to six months in swamps and years for open water lakes. This generally produces more complex and more extensive communities of flood dependent vegetation, with higher primary productivity, than those found elsewhere in Australia. Flows can take weeks to months to reach the dependent wetlands, which often lie at the ends of rivers, depending on the distance of the wetlands from the rainfall. Flows feeding the wetlands are characterised by high temporal and spatial variability (Maheshwari *et al.* 1995; Thoms and Sheldon 2000; Young and Kingsford 2006). Although the majority of the system is freshwater wetlands, some lakes are naturally saline and this influences the composition of the biotic community.

Distribution

The Murray-Darling Basin is one of 12 major river drainage basins in Australia (Figure 2a), covering about one seventh of the continent in the southeast (1.04 x 10^6 km^2). Within the Basin, there are about 28,000 wetlands mapped on the basis of flood extent (Figure 2b), covering about 5.68 million ha and most (>90%) of this area comprises floodplain wetlands, including swamps and marshes as well as forests and woodlands (Kingsford *et al.* 2004). Of the 28,000 mapped wetlands, relatively few (~20) are large wetland complexes associated with a single river and extend over hundreds of thousands of hectares for a single river (Kingsford 2004). Many of these large complexes lie at the ends of the 19 river systems within the Murray-Darling Basin, including 16 wetlands of international significance, listed under the Ramsar Convention (MDBA 2010, Pittock and Finlayson 2011), although a few of these belong to other ecosystem types.

Key processes and interactions

Ecological processes in floodplain wetlands are primarily driven by the boom and bust ecology of floodplain rivers (Boulton *et al.* 2006; Bunn *et al.* 2006; Brock *et al.* 2006; Kingsford *et al.* 2006; Young and Kingsford 2006). Natural river flow regimes are primarily responsive to rainfall in more mesic regions in the east. Natural flows drive hydrology, including the frequency and extent of inundation (Figure 3). They also control flow paths of small meandering channels in large wetlands, which shift over decadal and century periods, as a result of altered erosion and sedimentation processes (Yonge and Hesse 2010). Geomorphological and hydrological processes within the wetland are driven by upstream flows. These interact with local climate to produce different habitat types that drive primary and secondary productivity by creating the physico-chemical environment for different organisms and ecological processes (Figure 3). Floods trigger the release of nutrients, carbon and nitrogen cycling which stimulate key characteristic food web and life cycle processes including seed germination, egg hatching and movement between wetland areas. During boom flood periods, aquatic invertebrate food webs become highly productive as microcrustaceans either hatch out of sediments or are transported by floods and proliferate in wetland systems (Jenkins and Boulton 2007).

The frequency of flooding is critical for diversity and abundance of invertebrates (Boulton *et al.* 2006; Jenkins *et al.* 2009). Flow regimes govern dynamic zonation of flood dependent vegetation which responds to the frequency of flooding. For example, many aquatic macrophyte communities require frequent flooding regimes (Brock *et al.* 2006; Roberts and Marston 2011). The abundance of waterbirds in each functional group can correlate with food availability (Kingsford and Porter 1994). Waterbirds are also highly dependent on widespread flooding and rainfall to provide sufficient resources for breeding. Characteristically, many colonial waterbird species breed only when there is widespread flooding (Kingsford and Johnson 1998; Leslie 2001; Kingsford and Auld 2005; Brandis *et al.* 2011), with the extent of breeding often positively related to the number of nesting birds and even species richness (Kingsford and Johnson 1998). River flooding and floodplain inundation also stimulate responses from fish species, reptiles and frogs (Kingsford *et al.* 2006; Balcombe *et al.* 2007; King *et al.*2009; Wassens *et al.* 2010).

After flooding, large complex marshes and swamps generally remain inundated for up to about six months, but refugia (e.g. waterholes and lagoons) can retain water for much longer periods, along with lake systems, sometimes perennially (Kingsford *et al.* 2010). During dry periods organisms either leave the wetland (e.g. waterbirds), enter a resting phase (e.g. plants) or remain only as dormant eggs (e.g. invertebrates). Fires can occur during dry periods when floodplain vegetation is desiccated.

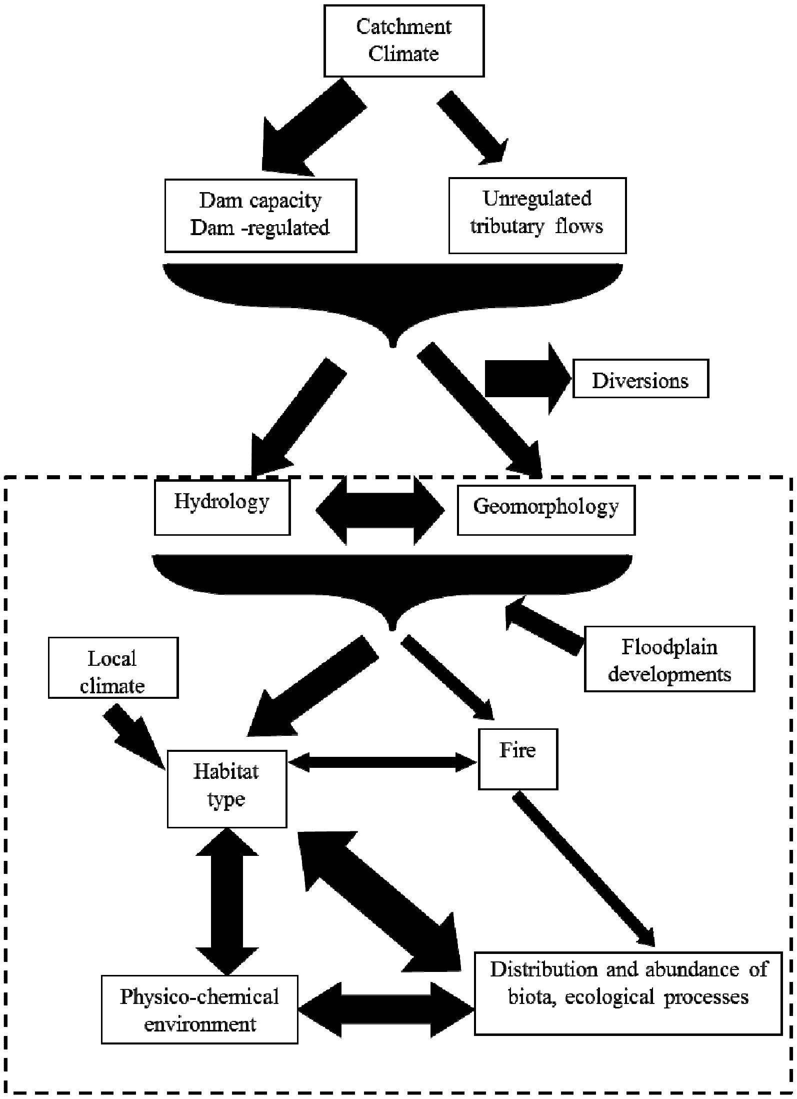

Figure S4. 3. Generalised cause and effect model summarising drivers of key characteristic biota and processes in floodplain wetlands (dashed rectangle) where the river system is regulated by dams in the upper catchment and floodplain developments within the floodplain wetland.

Threatening processes

The main threatening process is water resource development upstream which reduces frequency and extent of inundation of floodplain wetlands (Kingsford 2000a; Arthington and Pusey 2003). Flood regimes are significantly altered with the building of dams upstream of wetlands (Figures 2 and 3). These regulate flows which can then be controlled and diverted away from wetlands, mostly for irrigation (Figure 3; Kingsford *et al.* 2011). Hence, much of the diverted water no longer inundates marshes, swamps and lakes or replenishes groundwater systems. In addition to these major diversions upstream, floodplain developments including small dams, levees, channels and off-river storages (Steinfeld and Kingsford 2011), which often occur within the main wetland system, affect floodplain inundation, connectivity and health of flood dependent vegetation (Figure 3).

Changes in water quality, particularly increased salinity (Boon *et al.* 2006) and altered sedimentation are also key threats to floodplain ecosystems (Thoms *et al.* 2006). Other threats include introduced species (e.g. European carp *Cyprinus carpio*, water hyacinth *Eichhornia crassipes*), which may be exacerbated by river regulation (Gehrke *et al.* 1995). In addition, cereal crops are grown on some wetland systems, often after flood dependent vegetation is cleared (Jenkins and Briggs 1995). Finally, there is also increasing concern that reductions in rainfall and increases in temperatures will reduce duration of inundation of wetlands as a result of climate change (Kingsford 2011).

Ecosystem collapse

For assessment of criterion A and B, the ecosystem was assumed to collapse when its mapped distribution declines to zero, either as a consequence of conversion to agriculture or transition to dryland ecosystem types. Because the ecosystem is flood-dependent, the spatial extent of flooding was identified as an appropriate variable for assessing environmental degradation under criterion C. As collapse is likely to occur before flooding completely ceases, it was conservatively assumed that the ecosystem would collapse when flood extent is reduced to 90-100% of the mapped floodplain. Waterbird abundance was identified as an appropriate variable for assessing disruption of biotic processes and interactions under criterion D, as waterbirds plays a key role in flows of genes, matter and energy and also have important trophic roles within this ecosystem, and the disappearance of waterbirds would indicate a significant loss of key characterstic biota. Therefore we assumed that populations of all five functional groups of waterbirds must remain extant to avoid ecosystem collapse, with the threshold of collapse being the complete loss of any of the five functional groups.

ASSESSMENT

Summary

Criterion	A	B	C	D	E	overall
subcriterion 1	VU(VU-EN)	LC	VU(LC-EN)	EN(EN-CR)	DD	EN(EN-CR)
subcriterion 2	DD	LC	LC	DD		
subcriterion 3	LC(LC-VU)	LC	VU(LC-EN)	EN(VU-EN)		

Criterion A

Current decline: A decline in distribution of large swamps, marshes and lakes within the Murray-Darling Basin has occurred over the past 50 years in response to clearing and cropping as well as drying related to river regulation and accompanying water extraction. Some data are available on clearing and cropping, but only for recent years in part of the distribution. Based on total current area of wetlands (Kingsford et al. 2004), likely impacts on three major wetland areas (Keyte 1994; Kingsford and Thomas 1995; 2004) and linear effects of water resource development, at least 42% of wetlands have been lost in the Murray-Darling Basin since 1960. Declines in river flows are assessed further under criterion C. The status of the ecosystem is conservatively Vulnerable (plausible range Vulnerable - Endangered) under criterion A1.

Future decline: There are no projections on future trends in distribution available for large swamps, marshes and lakes, within the Murray-Darling Basin. The status of the ecosystem is Data Deficient under criterion A2.

Historic decline: Historic declines in ecosystem distribution to be estimated from available maps of expected native vegetation that were constructed from subjective models relating

remnant vegetation to landform, substrate and climate (Keith 2004). The estimates only account for declines attributable to land clearing and cropping, and do not take into account losses that may be attributable to water extraction or climate change. In the New South Wales portion of the Murray Darling Basin, the combined distribution of Inland Floodplain Wetlands and Inland Floodplain Shrublands was estimated to have declined by approximately 15% due to clearing and cropping since 1750 (based on updated map data from Keith 2004). While New South Wales accounts for the majority of the ecosystem distribution, declines in other states are likely to be similar or greater. The additional decline attributable to wetland drying is unknown but could be as much as 5-10%, based on draining of wetlands (Norman and Corrick 1988), prior to 1960 and a further 42%, after 1960. Based on these components, the overall historic decline is likely to be 47%-52% and so the status of the ecosystem is Least Concern (plausible range Least Concern - Vulnerable), under criterion A3.

Criterion B

About 5.68 million ha of wetland were mapped using recent satellite imagery (Kingsford *et al.* 2004). The current distribution of wetlands primarily reflects the elevation of different catchments within the Murray-Darling Basin, as well as the impacts of river regulation (Fig. 1b).

Extent of occurrence: A minimum convex polygon enclosing all mapped extant occurrences of open floodplain wetlands in the Murray Darling Basin includes at least 750,000 km^2. This is much larger than the threshold values of Extent of Occurrence for threatened categories and so the status of the ecosystem is Least Concern, under criterion B1.

Area of occupancy: Based on updated mapping of Inland floodplain swamps and Inland floodplain shrublands (updated v3.02 from Keith 2004), open floodplain wetlands in in the Murray Darling Basin occupy about 1500 10 × 10 km grid cells in New South Wales alone. Of these, the wetlands occupy more than 1% of the cell area in 997 cells. This greatly exceeds the threshold values of Area of occupancy for threatened categories and so the status of the ecosystem in Least Concern under criterion B2.

Locations: Wetlands occur within 19 different river systems within the Murray Darling Basin. As the most severe plausible threats are hydrologically based, these were assumed to represent 19 independent locations. Therefore, the status of the ecosystem is Least Concern under criterion B3.

Criterion C

Current decline: This process model (Fig. 3) and available research suggest that flood extent and river flow data are suitable for assessing rates of environmental degradation under criterion C. Time series of inundation maps are available for marshes and swamps in two of the major floodplain wetlands of the Murray-Darling Basin: the Lowbidgee floodplain on the Murrumbidgee River and the Macquarie Marshes on the Macquarie River (Kingsford and Thomas 1995; 2004). By hindcasting flow regimes and using historical maps, these latter studies showed 40 and 75% reductions in area of inundation for the Macquarie Marshes and Lowbidgee wetlands, respectively. These reductions occurred over approximately the last 50 years, a period when all irrigation areas were developed and water licences were fully activated for both the Macquarie Marshes and Lowbidgee wetlands. Some clearing of flood dependent vegetation also occurred during this time.

Although flood extent data are currently unavailable for other catchments within the Murray-Darling Basin, river flow data (from generic studies of hydrology across the Basin, CSIRO 2008) can be used to estimate changes in flood regimes in those catchments, relative to the

observed changes in the Macquarie and Lowbidgee. For example, the Macquarie-Castlereagh and Lachlan catchments are currently subject to similar levels of 20-30% water extraction (Table 1). As the Macquarie Marshes underwent a 40% reduction in flood extent in response to this level of water use (Table 1), a similar reduction in flood extent may be assumed for wetlands of the Lachlan. This assumed relationship between changes in river flows and changes in flood extent is justified because: i) most large swamps, marshes and lakes occur at the ends of other rivers in Murray-Darling Basin; ii) most catchments, with the exception of the Warrego and Paroo, had similar water resource development trajectories and similar declines in river flows (Kingsford 2000a; CSIRO 2008);and iii) end of system flows are correlated with loss of wetland area (Kingsford and Thomas 1995; 2004) and function (Kingsford *et al.* 2004). Thus by summing the initial wetland extent for the last four rows of Table 1, it was estimated that 71% of ecosystem extent underwent similar levels of reduction in flood extent to that observed for the Macquarie Marshes and Lowbidgee wetlands (i.e. 40-75% reduction). In addition, the major lake systems of the Murray-Darling Basin, the Coorong and Lower Lakes, may have had significant reductions in functionality, as only about 28% of median flows still reach the system (72% reduction), resulting in a likely change in ecological character (Kingsford *et al.* 2011).

A standardised estimate of relative severity of environmental degradation may be calculated by assuming that the ecosystem collapses when flood extent is reduced to a critical threshold. For example, at one extreme the cessation of all floods (100% reduction) would inevitably cause collapse of the ecosystem. As the ecosystem is flood-dependent (Fig. 3), collapse is likely to occur before complete cessation of flooding occurs, For the purpose of assessment, it was conservatively assumed that the ecosystem would collapse when flood extent is reduction by 90-100%. The relative severity of environmental degradation is therefore between 40/1.0 = 40% and 75/0.9 = 83% across 71% of the extent of the ecosystem, and is status is thus Vulnerable (plausible range Least Concern - Endangered) under criterion C1.

Table S4. 1. Relative level of water use, relative to natural, for 18 catchments in the Murray-Darling Basin (data from CSIRO 2008). Proportional extent is based on mapping of wetlands in 1984-1993 (Kingsford *et al.* 2004) and assessment of likely wetland extent in 1750, based on alteration of hydrology.

Current level of water use	Initial extent of wetlands(%)[a]	Catchments	Reductions in flood extent
Low (<10%)	11.7	Paroo, Ovens[b]	
Moderate (10-20%)	15.7	Barwon-Darling, Warrego	
Moderately high (20-30%)	21.1	Macquarie-Castlereagh, Lachlan	40% (Macquarie)
High (30-40%)	9.9	Border Rivers, Campaspe[c], Loddon-Avoca[c], Moonie, Murray[c], Namoi	
Very high (40-50%)	2.3	Goulburn-Broken, Gwydir	
Extremely high (>50%)	37.7	Condamine-Balonne, Murray-Darling Basin[d], Murrumbidgee, Wimmera	75% (Lowbidgee)

[a]Estimated % of ecosystem extent by assessing area of wetlands likely in 1960 on the basis of impacts of water resource developments in each catchment since then (Kingsford *et al.* 2004)
[b]Includes Upper Murray catchment (Victoria) (Kingsford *et al.* 2004)
[c]Includes Upper Murray catchment (NSW); Murray-Riverina, Benanee (Kingsford *et al.* 2004)
[d]Includes Lower Murray catchment (NSW, SA) (Kingsford *et al.* 2004)

Future decline: In addition to current decline, primarily as a result of water resource development, increased temperatures from climate change will reduce the duration of flooding and rainfall. This and the revegetation of catchments are likely to reduce flows to floodplain wetlands by an additional 15% by 2030 (Herron *et al.* 2002). Besides these sources of degradation, future government policy is aiming to return some water currently diverted for irrigation (2,750 GL yr^{-1}) to the environment, improving environmental flows to wetlands (MDBA 2011). If it occurs, the return of water to the rivers will improve the status of swamps and marshes but negative impacts of climate change will reduce effectiveness of increased flows to marshes, swamps and lakes. The net outcome of these compensatory changes is likely to be either restoration of some previously lost hydrological function or at least a slower future rate of environmental degradation with relative severity less than 30%. The status of the ecosystem would therefore be Least Concern under criterion C2.

Historic decline: There are insufficient data to estimate changes to flood extent prior to 1960, but the majority of water resource development occurred during the past 50 years. The historic reduction in flood extent is therefore likely to be similar to that estimated for the past 50 years, or perhaps 5-10% greater because there is evidence for widespread draining of swamps in the Murray-Darling Basin primarily for agriculture, but largely unrelated to, and preceding water resource development (Norman and Carrick 1988; Kingsford and Thomas 2004). This occurred within the same catchments that were later exposed to water resource development. The extent of historic environmental degradation due to reduced flooding was therefore estimated to cover 71% of the ecosystem distribution (same as current) with a relative severity of 45-93% (5-10% greater than current). The status of the ecosystem spans Least Concern, Vulnerable and Endangered, under criterion C3.

Criterion D

As waterbirds are central to several biotic interactions within the ecosystem and highly dependent on ecosystem function and water regimes (Fig. 3), the abundance of various waterbird functional groups is a suitable response variable for assessing criterion D. Long-term data sets on waterbirds are available for about 10% of wetlands in the Murray-Darling Basin which have been surveyed as part of an aerial survey each October between 1983-2011 (Figure 1). This long-term survey covers some of the major wetland systems in the Murray-Darling Basin, including Paroo overflow lakes, Menindee Lakes, Macquarie Marshes, Lowbidgee wetlands, Wallenjoe wetland system and the southern part of the Coorong wetlands. It also covers many small wetlands and rivers. Fixed 30 km wide survey bands, 200 km apart, extend across the Murray-Darling Basin (Kingsford and Porter 2009; Figure 2a). All waterbirds (>50 taxa) are identified and counted in each wetland within the survey bands to provide estimates of abundance and diversity.

These data were used to assess the severity and extent of changes to biotic interactions within the ecosystem. All waterbird species were assigned to one of five functional groups: ducks, herbivores, large wading birds, piscivores and small wading birds (Kingsford and Porter 1994). Long-term changes in abundance were modelled, estimating the effect of inundated area and time on total counts, using generalized additive models with a LOESS (locally weighted regression) smoother for Poisson distribution with log link function. Fitted data were compared to actual count data using the goodness of fit and a Mann-Kendall trend test (Mann 1945) was used to show the direction of trends in annual counts.

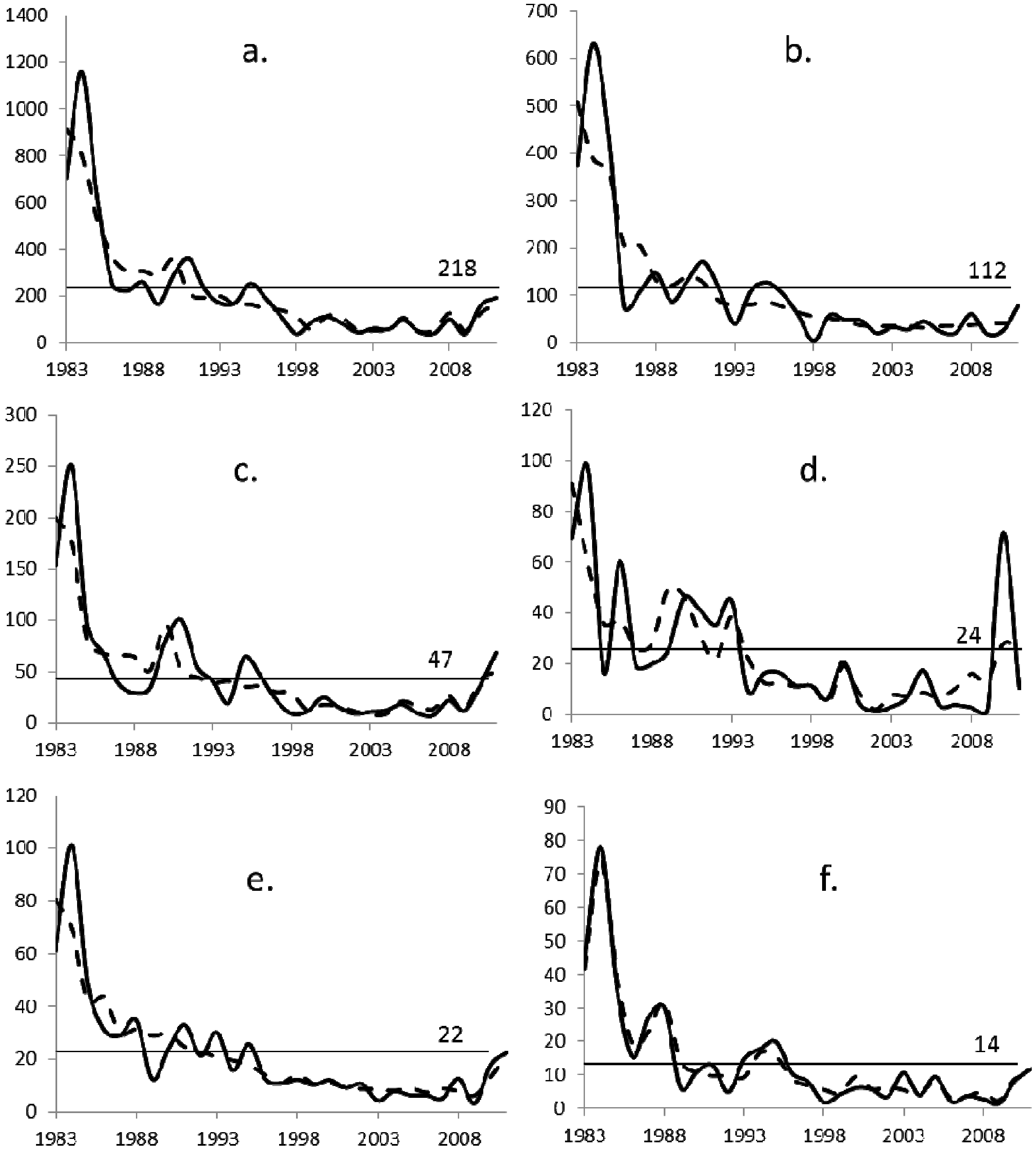

Figure S4. 4. Total numbers of all waterbirds (x 1000) (a), ducks (b), herbivorous waterbirds (c), large wading birds (d), piscivores (e) and small wading birds (f), showing long-term declines in the Murray-Darling Basin. Data were collected during annual aerial surveys of eastern Australia (1983-2011). Actual data linked by continuous line and dotted line shows modelled data, using year and wetland area. Long-term averages given as horizontal lines, with actual mean abundance for each category.

Current decline: There is strong evidence that most functional groups of waterbirds have experienced declines of >50% in abundance in the Murray-Darling Basin wetlands over a

period of 29 years, since the early 1980s (Figure 4). Only piscivores have exhibited some recovery above the long-term mean in more recent years (Figure 4d), despite significant flooding in 2010 that broke a long period of drought. Assuming that hydrological conditions were similar in the early 1980s to previously, it is plausible that the high numbers of the early 1980s (Figure 4), occurred in the previous 21 years. There may have been some decline in waterbird numbers prior to survey commencement, although construction of dams and irrigation infrastructure was significant in the 1950-1970 period, full diversion of water from rivers occurred later as irrigation areas were developed (Kingsford *et al.* 2011). Full development of all rivers probably occurred in the period of 1985-1995 when the last major river, the Condamine-Balonne was developed (Kingsford 200b). It was also assumed that populations of all five functional groups of waterbirds must remain extant to avoid ecosystem collapse. The severity of declines in waterbird numbers varied between 56 and 79% for five of the six functional groups, with the other group declingin by 85% (Table 2) over the past 50 years, assuming their populations were stable prior to start of the aerial survey in 1983. As the surveys sampled the full extent of the Murray-Darling Basin, these observed declines were assumed to have occurred over 100% of the extent of the ecosystem. Therefore, the status of the ecosystem is Endangered (plausible range Endangered - Critically Endangered), under Criterion D1.

It is noteworthy that these general population trends are reflected in data on waterbird breeding. Breeding of colonial waterbirds is also a key process that forms the basis of some flow management decision-making in floodplain wetlands. The frequency and size of breeding events is highly dependent on the size and frequency of floods with threshold effects (Kingsford and Johnson 1998; Arthur *et al.* in press). Large scale breeding of colonial waterbirds occurs with large floods in relatively few sites (~10), reflecting availability of resources. Declining flows have reduced the frequency and extent of breeding on wetland ecosystems (Kingsford and Johnson 1998; Leslie 2001). Given the relatively few areas where such significant breeding occurs, mostly on the regulated Murray-Darling Basin Rivers (Marchant and Higgins 1990) and the effects of the declining flows on inundation patterns (Thomas *et al.* 2011), breeding has declined.

Table S4. 2**.** Summary of statistical analyses of trends (all significant declines, $p<0.001$) in total waterbird numbers on wetlands of the Murray-Darling Basin, 1983-2011 and five functional groups of waterbirds.

Waterbird group	Decline[a] (%)	tau	Goodness of fit
All waterbirds	73	-0.586	83.7
Ducks	79	-0.581	77.4
Herbivores	56	-0.478	79.8
Large wading birds	85	-0.512	61
Piscivores	63	-0.635	80.9
Shorebirds	74	-0.542	96

[a]2011 estimate relative to 1983 estimate

<u>Future decline:</u> Future trends in waterbird numbers depend on climate change and government water policy and regulations, which are currently under review. The models required to make a robust projection of waterbird numbers into the future are yet to be developed. Consequently, the status of the ecosystem is Data Deficient under Criterion D2.

Historic decline: Few data exist for long-term changes beyond those in the last 50-100 years, but it is likely that water resource development prior to 1960 had a relatively minor effect on waterbird populations. The historical declines were therefore assumed to be of a similar severity and extent to those observed in the past 50 years. The status of the ecosystem is therefore Endangered (plausible range Vulnerable - Endangered- under Criterion D3.

Criterion E

There are no data to estimate risk of ecosystem collapse but the recent dry period was close to forcing collapse of the major wetland at the mouth of the River Murray – the Lower Lakes and the Coorong. Decreasing water levels, increased salinity and acidification of large areas of the major lake systems, considerably altered the ecology of the wetland (Kingsford *et al.* 2011). Under criterion E, the status of the ecosystem is Data Deficient.

REFERENCES

Arthington AH and Pusey BJ. 2003. Flow restoration and protection in Australian rivers. *River Research and Applications* **19**: 377-395.

Arthur AD, Reid JRW, Kingsford RT, McGinness HM, Ward KA and Harper MJ. in press. Breeding flow thresholds of colonial breeding waterbirds in the Murray-Darling Basin, Australia. *Wetlands*.

Bailey PCE, Boon PI, Blinn DW and Williams WD. 2006. Salinisation as an ecological perturbation to rivers, streams and wetlands of arid and semi-arid regions. In *Ecology of desert rivers*, RT Kingsford. Ed. Cambridge, Cambridge Academic Press: pp. 280-314.

Balcombe SR, Bunn SE, Arthingon AH, Fawcett JH, McKenzie-Smith FJ and Wright A. 2007. Fish larvae, growth and biomass relationships in an Australian arid zone river: links between floodplains and waterholes. *Freshwater Biology* **52**: 2385-2398.

Boulton AJ, Sheldon F and Jenkins KM. 2006. Natural disturbance and aquatic invertebrates in desert rivers. In *Ecology of desert rivers*. RT Kingsford. Ed. Cambridge, Cambridge University Press: pp. 133-153.

Brandis K, Kingsford R.T., Ren S and Ramp D. 2011. Crisis water management and ibis breeding at Narran Lakes in arid Australia. *Environmental Management* **48**: 489-498.

Brock M, Capon S and Porter J. 2006. Disturbance of plant communities dependent on desert rivers. In *Ecology of Desert Rivers*. RT Kingsford. Ed. Cambridge, Cambridge University Press: pp. 100-132.

Bunn SE, Balcombe SR, Davies PM, Fellows CS and McKenzie-Smith FJ. 2006 . Aquatic productivity and food webs of desert river ecosystems. In *Ecology of desert rivers*. RT Kingsford. Ed. Cambridge, Cambridge University Press: pp. 76-99.

CSIRO. 2008. Water availability in the Murray-Darling Basin. A report to the Australian Government from the CSIRO Murray-Darling Basin Sustainable Yields Project. CSIRO, Australia. 67pp.

Gehrke PC, Brown P, Schiller CB, Moffatt DB and Bruce AM. 1995. River regulation and fish communities in the Murray-Darling River system, Australia. *Regulated Rivers: Research and Management* **11**: 363-375.

Herron N, Davis R and Jones R. 2002. The effects of large-scale afforestation and climate change on water allocation in the Macquarie River catchment, NSW, Australia. *Journal of Environmental Management* **65**: 369-381.

Jenkins KM and Briggs SV. 1995. Ecological management of lakebed cropping on the Lakes of the Great Anabranch of the Darling River. *Final unpublished report, Australian Nature Conservation Agency*: 181pp.

Jenkins KM and Boulton AJ. 2007. Detecting impacts and setting restoration targets in arid-zone rivers: aquatic micro-invertebrate responses to reduced floodplain inundation. *Journal of Applied Ecology* **44**: 823-832.

Jenkins KM, Kingsford RT and Ryder D. 2009 Developing indicators for floodplain wetlands: managing water in agricultural landscapes. *Chiang Mai Journal of Science* **36**: 224-235.

Keith DA. 2004. Ocean shores to desert dunes: the native vegetation of New South Wales and the ACT. NSW Department of Environment and Conservation, Sydney.

Keyte P. 1994 *Lower Gwydir Wetland – Plan of Management 1994–97*. Report by New South Wales Department of Water Resources for the Lower Gwydir Wetland Steering Committee, Sydney.

King AJ, Tonkin Z and Mahoney J. 2009. Environmental flow enhances native fish spawning and recruitment in the Murray River, Australia. *River Research and Applications* **25**: 1205-1218.

Kingsford RT and Porter JL. 1994. Waterbirds on an adjacent freshwater and salt lake in arid Australia. *Biological Conservation* **69**: 219-228.

Kingsford RT and Thomas RF. 1995. The Macquarie Marshes and its waterbirds in arid Australia: a 50-year history of decline. *Environmental Management* **19**: 867-878.

Kingsford RT and Johnson W. 1998. Impact of water diversions on colonially nesting waterbirds in the Macquarie Marshes in arid Australia. *Colonial Waterbirds* **21**: 159-170.

Kingsford RT. 2000a. Ecological impacts of dams, water diversions and river management on floodplain wetlands in Australia. *Austral Ecology* **25**: 109-127.

Kingsford RT. 2000b. Protecting or pumping rivers in arid regions of the world? *Hydrobiologia* **427**: 1-11.

Kingsford RT, Thomas RF, Curtin AL. 2001. Challenges for the conservation of wetlands on the Paroo and Warrego Rivers. *Pacific Conservation Biology* **7**: 21-33.

Kingsford RT, Brandis K, Thomas RF, Knowles E, Crighton P and Gale E. 2004. Classifying landform at broad landscape scales: the distribution and conservation of wetlands in New South Wales, Australia. *Marine and Freshwater Research* **55**: 17-31.

Kingsford RT. 2004. Wetlands and waterbirds of the Darling River. In *The Darling*. R. Breckwoldt, Boden, R., Andrew, J. Eds. Canberra, Murray-Darling Basin Commission**:** 234-259 pp.

Kingsford RT and Thomas RF. 2004. Destruction of wetlands and waterbird populations by dams and irrigation on the Murrumbidgee River in arid Australia. *Environmental Management* **34**: 383-396.

Kingsford RT and Auld KM. 2005. Waterbird breeding and environmental flow management in the Macquarie Marshes, arid Australia. *Rivers Research and Applications* **21**: 187-200.

Kingsford RT, Georges A and Unmack PJ. 2006. Vertebrates of desert rivers-meeting the challenges of temporal and spatial unpredictability. In *Ecology of Desert Rivers.* RT Kingsford. Ed. Cambridge, Cambridge University Press: 154-200 pp.

Kingsford RT and Porter JL. 2009. Monitoring waterbird populations with aerial surveys - what have we learnt? *Wildlife Research* **36**: 29-40.

Kingsford RT, Roshier DA, Porter JL. 2010. Australian waterbirds - time and space travelers in dynamic desert landscapes. *Marine and Freshwater Research* **61**: 875-884.

Kingsford RT. 2011. Conservation management of rivers and wetlands under climate change - a synthesis. *Marine and Freshwater Research* **62**: 217-222.

Kingsford RT, Walker KF, Lester RE, Young WJ, Fairweather PG, Sammut J and Geddes MC. 2011. A Ramsar wetland in crisis – the Coorong, Lower Lakes and Murray Mouth, Australia. *Marine and Freshwater Research* **62**: 255-265.

Leslie DJ. 2001. Effect of river management on colonially-nesting waterbirds in the Barmah-Millewa forest, south-eastern Australia. *Regulated Rivers: Research and Management* **17**: 21-36.

Maheshwari BL, Walker KF and McMahon TA. 1995. Effects of regulation on the flow regime of the River Murray, Australia. *Regulated Rivers - Research and Management* **10**: 15-38.

Mann HB. 1945. Nonparametric Tests Against Trend. *Econometrica* **13**: 245-259.

Marchant S and Higgins PJ, Eds. 1990. Handbook of Australian, New Zealand and Antarctic birds. Volume 1 Ratites to ducks. Melbourne, Oxford University Press.

MDBA. 2010. Guide to the proposed Basin Plan. Technical background. Murray-Darling Basin Authority, Canberra, Australia.

MDBA. 2011. Proposed Basin Plan. Murray Darling Basin Authority, Canberra, Australia, Publication no: 192/11.

Norman FI and Carrick AH. 1988. Wetlands in Victoria: a brief review. *In The Conservation of Australian wetlands.* McComb, AJ. and Lake, PS. Eds. Surrey Beatty & Sons, Sydney. 17-34 pp.

Pittock J and Finlayson CM. 2011. Australia's Murray–Darling Basin: freshwater ecosystem conservation options in an era of climate change. *Marine and Freshwater Research* **62**: 232-243.

Ren S and Kingsford R. 2011. Statistically Integrated Flow and Flood Modelling Compared to Hydrologically Integrated Quantity and Quality Model for Annual Flows in the Regulated Macquarie River in Arid Australia. *Environmental Management* **48**: 177-188.

Roberts J and Marston F. 2011. Water regime for wetland and floodplain plants: a source book for the Murray–Darling Basin. National Water Commission, Canberra.

Steinfeld CMM and Kingsford RT. 2012. Disconnecting the floodplain: earthworks and their ecological effect on a dryland floodplain in the Murray–Darling Basin, Australia. *River Research and Applications*.

Thoms MC, Beyer P and Rogers KH. 2006. Variability, complexity and diversity - the geomorphology of river ecosystems in dryland regions. *In Ecology of Desert Rivers.* RT Kingsford. Ed., pp. 47-76. Cambridge University Press, Cambridge.

Thoms M and Sheldon F. 2000. Water resource development and hydrological change in a large dryland river: the Barwon-Darling River Australia. *Journal of Hydrology* **228**: 10-21.

Thomas RF, Kingsford RT, Lu Y and Hunter SJ. 2011. Landsat mapping of annual inundation. 1979–2006 of the Macquarie Marshes in semi-arid Australia. *International Journal of Remote Sensing* **32**: 4545-4569.

Yonge D and Hesse PP. 2009. Geomorphic environments, drainage breakdown, and channel and floodplain evolution on the lower Macquarie River, central-western New South Wales. *Australian Journal of Earth Sciences* **56**: S35-S53.

Young WJ and Kingsford RT. 2006. Flow variability in large unregulated dryland rivers. In *Ecology of Desert Rivers*. RT Kingsford. Ed. Cambridge: Cambridge University Press: 11-46 pp.

Wassens S, Hall A, Osborne W and Watts RJ. 2010. Habitat characteristics predict occupancy patterns of the endangered amphibian *Litoria raniformis* in flow-regulated flood plain wetlands. *Austral Ecology* **35**: 944-955.

5 Aral Sea, Uzebekistan and Kazakhstan

contributed by David Keith[1],
Australian Wetlands, Rivers and Landscapes Centre, University of NSW; and NSW Office of Environment and Conservation, Australia.

Classification

International: The Aral Sea is one of seven large continental water bodies in central Asia, the others being the Caspian Sea and lakes Balkhash, Issyk-Kul, Chany, Alakul and Tengiz (Aladin & Plotnikov 1993).
IUCN Habitats Classification Scheme (Version 3.0): 5. Wetlands (inland) / 5.14 Permanent Saline, Brackish or Alkaline Lakes

Ecosystem Description

Characteristic native biota

The characteristic native biota of the Aral Sea includes a unique combination of freshwater and saltwater assemblages of free-living and benthic aquatic biota as well as associated wetland vegetation and bird assemblages (Aladin & Potts 1992). For example, a biogeographic analysis of Aral invertebrates suggested that 17% were of Caspian origin, 78% were of freshwater or saline water continental origin and 5% were of Mediterranean or oceanic origin (Yablonskaya 1974). Aladin & Potts (1992) compiled lists from various surveys and estimated the sea's biota to include 20 species of fish, 195 species of free-living invertebrates and 71 species of parasites, 12 species of higher plants and 82 species of lower plants. Williams and Aladin (1991) remark that the Aral Sea biota is neither diverse or productive.

The Aral phytoplankton consisted mostly of diatoms, flagellates and blue-green algae, with biomass fluctuating from 0.5 to 2.6 g.m-^{3} (Aladin & Potts 1992). Species richness was greatest in the estuaries of the major rivers and lowest in the most saline sites. Only diatoms were common in the open sea, with *Actinocuclus ehrenbergii* var. *crassa* the dominant species present in densities of more than 1 million per cubic metre. Zooplanktonic biomass averaged 150 mg.m^{-3}, with higthest values in the shallow coastal waters. Common zooplankton in the open sea include: cladocerans *Podonevadne camptonyx, Evadne anonyx, Moina mongolica, Ceriodaphnia reticulata* and *Alona rectangula*; copepods *Arctodiaptomus salinus* and *Mesocyclops leuckarti*; rotifers *Synchaeta vorax, Keratella tropica* and *Brachionus plicatilis*; and mollusc larvae *Dreissena* and *Hypanis* spp. (Aladin & Potts 1992). Of these, *Arctodiaptomus salinus* was by far the most common, accounting for more than three-quarters of the zooplanktonic biomass. Several species of zooplankton were introduced to the sea during the 1960s (Aladin & Potts 1992).

The benthic flora of the Aral Sea consisted of angiosperms, green and red algae and charophytes, with species composition varying with sediment type and depth. The most common species include the angiosperm *Zostera nana*, the green algae *Polysiphonia violacea* and *Vaucheria dichotoma*, and the charophyte *Tolypella aralica* (Aladin & Potts 1992).

The benthic biomass of the Aral Sea averaged 23 g.m^{-2} and was dominated by molluscs (Aladin & Potts 1992). The benthic macro-invertebrates include bivalve molluscs *Dreissena polymorpha* var. *aralensis*, *D. polymorpha* var. *obtusecarinata, D. caspia* var. *pallasi,Hypanis*

minima var. *minima, H. minima* var. *sidorov*; oligochaete worms *Nais elengui* and *Paranais simplex*; ostracods *Cyprideus torosa*; amphipods *Dikerogammarus aralensis*; an dinsect larvae *Chironomus* and *Oecetis*. The Aral crustacean fauna includes some species of marine origin, such as *Evadne anonyx* and *Podonevadne camptonyx*, while others, such as *Cercopagispengoi aralensis*, derived from freshwater ancestors (Aladin 1995). Several species of crustacea and molluscs were introduced to the sea during the 1960s (Aladin & Potts 1992).

The Aral Sea fish fauna included bream (*Abramis brama* var. *orientalis*), carp (*Cyprinus carpio* var. *carpio*), Aral roach (*Rutilus rutilus* var. *aralensis*), Aral shemaya (*Chalcalburnus chalcoides*), zander (*Stizostedion lucioperca*), perch (*Perca jluviatilis*), ruffe (*Acerina cernua*), the Aral spiny sturgeon (*Acipenser nudiventris*), Aral sea trout (*Salmo trutta* var. *aralensis*), wels (*Silurus glanis*), pike (*Esox lucius*), and the Aral stickleback (*Pungitius platigaster* var. *aralensis*) (Aladin & Potts 1992). In addition to the twenty native species, fifteen additional species of fish were successfully introduced to the Aral Sea between 1920 and 196,1 including the Caspian sturgeon (Huso huso), the Caspian shad (*Alosa caspia*), the Baltic herring (*Clupea herengus membras*), grass carp (*Ctenopharyngodon idella*), round goby (*Pomatoschistis caucasicu*), monkey goby (*Neogobius melanostomus oficinus*), sand goby (*Neogobius juciatilis pallasi*), atherines (*Atherinus*), pipe-fish, and the Black Sea flounder (*Platichthys flesus* var. *lulscus*) (Aladain & Potts 1992). The native and introduced fish supported a fisheries industry with an average annual catch of 5 $kg.ha^{-1}$ reaching a maximum total annual catch for the whole sea of 44,000 tonnes, however the introductions did not significantly increase the annual fish catch (Aladin & Potts 1992).

Extensive reedbeds dominated by *Phragmites australis* lined hundreds of kilometres of the Aral Sea shoreline and were extensive in the river deltas, particularly in the south. The reedbeds, together with tugay woodlands in the river deltas and lower floodplains, provided breeding and foraging habitats for a diverse assemblage of birds. Other species forage in the sea itself, including the Dalmatian Pelican (*Pelicanus crispus*) and Great White Pelican (*P. onocrotalus*). These and other waterbirds are important dispersal vectors for planktonic biota and other aquatic taxa (Boomer et al. 1996). The total bird fauna of the sea and associated wetlands has been estimated at 319 species (Micklin & Aladin 2008).

Abiotic environment

The Aral Sea, the fourth largest continental water body in the world, occurs in a large topographic basin in central Asia (Fig. 1). It is fed by two major rivers, the Syr Dar'ya and Amu Dar'ya, which originate in the northern and western Himalayas and have a combined catchment area of more than 1.5 million km^2. In 1960, the sea covered 67,500 km^2, exceeding 20 m depth across about one-third of its area and 50 m at its deepest point (Boomer et al. 2000). Hydrologically, the sea level was approximately stable varying less than one metre during 1911-1960. During this time inflows balanced net evaporation, each approximately 56 $m^3.yr^{-1}$, with salinity approximately 10 $g.l^{-1}$ (Micklin 2006). Earlier observations suggest a longer period of sea level stability, with variations of less than 4.5 m since the mid-eighteenth century until the 1960s (Bortnik 1996). Over longer time scales, there is evidence of more substantial water-level fluctuations of up to 45 m since the late Pleistocene. Boomer et al. (2000) describe the geomorphological evolution of the sea from the early Pleistocene and map inferred fluctuations in its extent over the past 10,000 years. Since its initial filling, the Aral Sea has periodically been connected with the Caspian Sea via the Sarykamysh Depression and the Uzboi Channel to the southeast, and this has been important for colonisation of marine biota (Boomer et al. 1996, 2000).

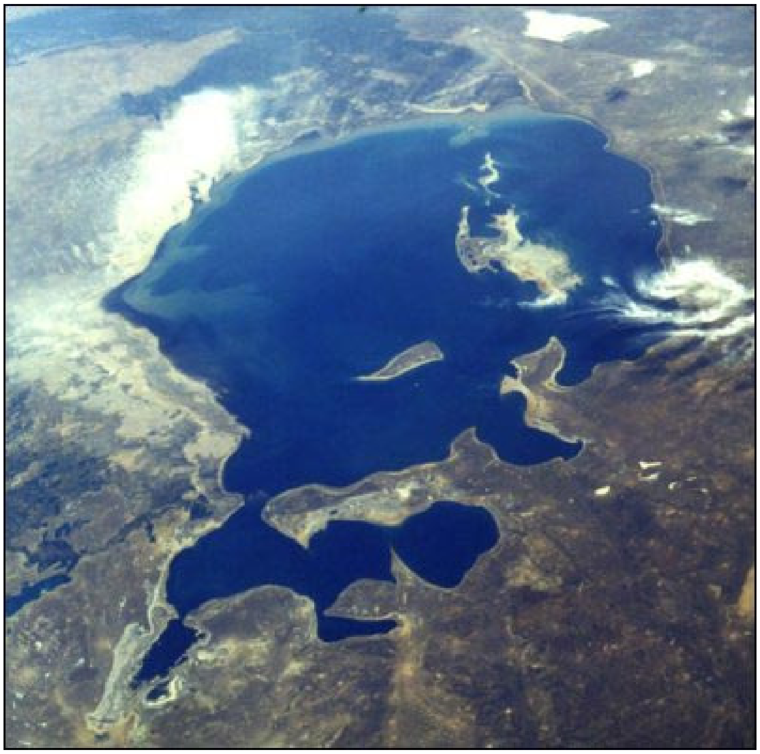

Figure S5. 1. Aral Sea oblique view taken from the north by NASA Space Shuttle mission STS-51-F on 6th August 1985. Source: http://en.wikipedia.org/wiki/Aral_Sea from http://eol.jsc.nasa.gov/sseop/EFS/photoinfo.pl?PHOTO=STS51F-36-59

Distribution

The Aral Sea is centred on 45°N and longitude 60°E, straddling the border between Uzebekistan and Kazakhstan in central Asia. In 1960 it was estimated to cover a contiguous area of 67,500 km^2. At the present time three lakes occupy the former sea bed with a combined area of 17,000 km^2 (Fig. 2).

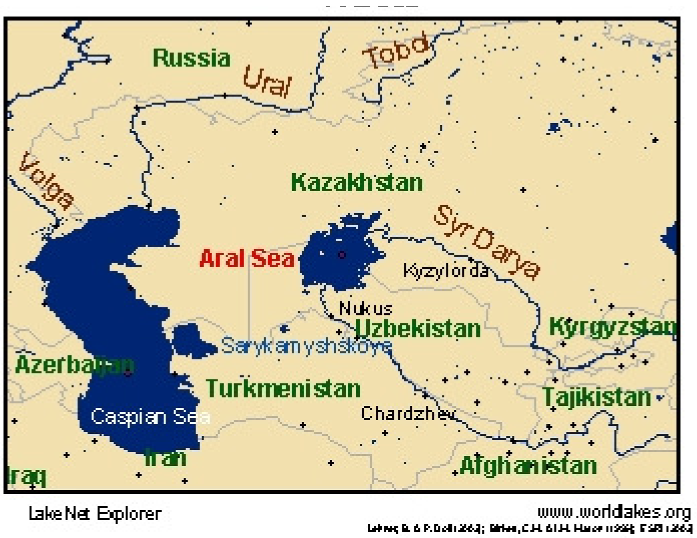

Figure S5. 2. Location of the Aral Sea on the border between Uzbekistan and Kazakhstan in central Asia. Source: World Lakes (http://www.worldlakes.org)

Key processes and interactions

Benthic vegetation and phytoplankton provide the primary productivity that sustains the characteristic assemblages of zooplankton, benthic macro-invertebrates and fish within the sea (Fig. 3). Productivity is promoted by nutrient inflow through the riparian and delta ecosystems connected to the sea (Aladin & Potts 1992, Micklin 2006). Hydrological connectivity also permits movement of freshwater organisms between these ecosystems (Fig. 3). Fringing reedbeds and riparian vegetation also contribute to primary productivity and provide breeding habitat for a variety of birds and foraging resources for mammals (Aladin & Potts 1992, Micklin 2006). Waterbirds, including migratory species, that consume fish and aquatic invertebrates from the sea play important roles in dispersing other organisms and transferring nutrients between the Aral Sea and other wetlands in the region (Boomer et al. 1996).

River inflow regulates the volume and water chemistry of the Aral Sea, and hence the relative abundance of freshwater and marine biota in the brackish ecosystem (Williams & Aladin 1991). The sea expands when inflows from rivers exceed net evaporative loss and contracts when inflows fail to compensate evaporative loss (Micklin 2006). When sea levels are high, salinity levels are low and freshwater biota predominate. As sea levels fall, the waters become more saline and saltwater biota predominate. Extreme drying results in transition of the brackish Aral Sea ecosystem into hypersaline lakes and desert (Fig. 3).

Climate and the human activity in the catchment of the Aral Sea influence its hydrological dynamics by regulating water inflow (Fig. 3). Regression of the sea corresponds with episodes of climatic drying and water extraction (Boomer et al. 2000, Micklin 2006). Drying of the sea may also have feedback effects on regional climate warming (Khan et al. 2004).

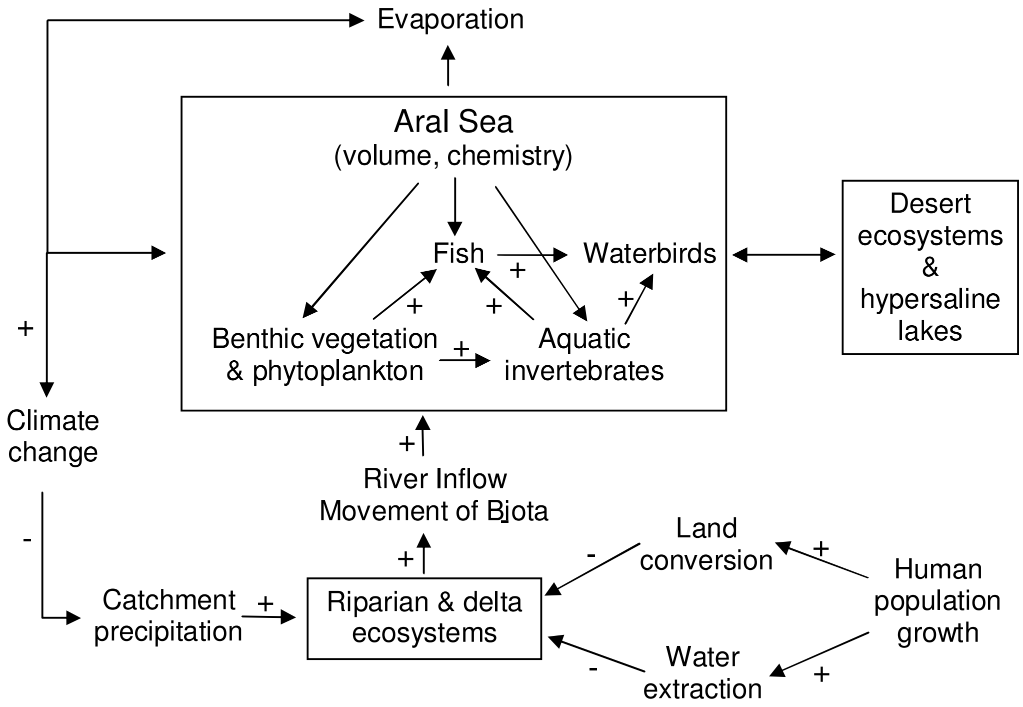

Figure S5. 3. Cause-effect model of ecosystem dynamics for the Aral Sea showing positive (+) and negative (-) environmental and anthropogenic influences on ecosystem processes and components, and interactions with neighbouring ecosystems. Feedbacks from the Aral Sea ecosystem to the regional human population are not shown.

Threatening Processes

The major threat to the Aral Sea ecosystem is desiccation and salinisation caused primarily by water extraction to support expansion of irrigated agriculture in the river valleys and deltas of the Syr Dar'ya and Amu Dar'ya and the adjacent desert (Micklin 1988, Micklin & Aladin 2008). Although irrigation has been practiced in the region for more than 5,000 years, the area under irrigation tripled between 1949 and 1986 from 2.14 to 6.41 million ha (Micklin 1988, Boomer et al. 2000), and by 2007 had expanded to 7.9 million ha (Micklin 2006). As irrigation expanded into the desert, more water was lost to evaporation than when irrigation was confined to the river deltas prior to 1950. This reduced inflow to the sea from 56 $m^3.yr^{-1}$ before 1960 to 5 -15 $m^3.yr^{-1}$ during 1970 - 2006, resulting in a substantial deficit to net evaporation. Consequently, the sea dried up throughout much of its area, fragmented into several separate lakes and the salinity of the remaining water increased by more than an order of magnitude (Micklin 2006). The hydrological changes precipitated a dramatic loss of invertebrate fauna, fish and plants from the sea. As well, the reedbeds of the sea shorelines have disappeared and the tugay woodlands and reedbeds of the river deltas have contracted to one-third of their area, resulting in a major diminution of the mammalian and avian fauna dependent upon the vegetation. Micklin and Aladin (2008) summarise the changes between 1960 and the mid 1990s as follows: marsh vegetation contracted from 100,000 ha to 15,000 ha; fish species declined from 32 to six; bird species declined from 319 to 160; and mammal species declined from 70 to 32. While some of these species remain in the reduced river systems or in other regions, others are presumed extinct (Aladin & Potts 1992, Micklin 2006). Drying of the sea also resulted in collapse of shipping and fisheries industries (Fig. 4), reducing dietary variety and increasing malnutrition. Wind-born dust and aerosols from the dry sea floor are also associated with major public health problems including respiratory illnesses, cancer, and digestive disorders, liver and kidney ailments, eye problems and elevated infant mortality (Micklin & Aladin 2008).

Historically, introduced species also posed a threat to the native biota, significantly altering the composition of fish, zooplankton and macro-invertebrate assemblages (Aladin & Potts 1992). However, these effects have largely been overriden by the impacts of desiccation and salisation, and many of the introduced species have been eliminated, along with the native biota.

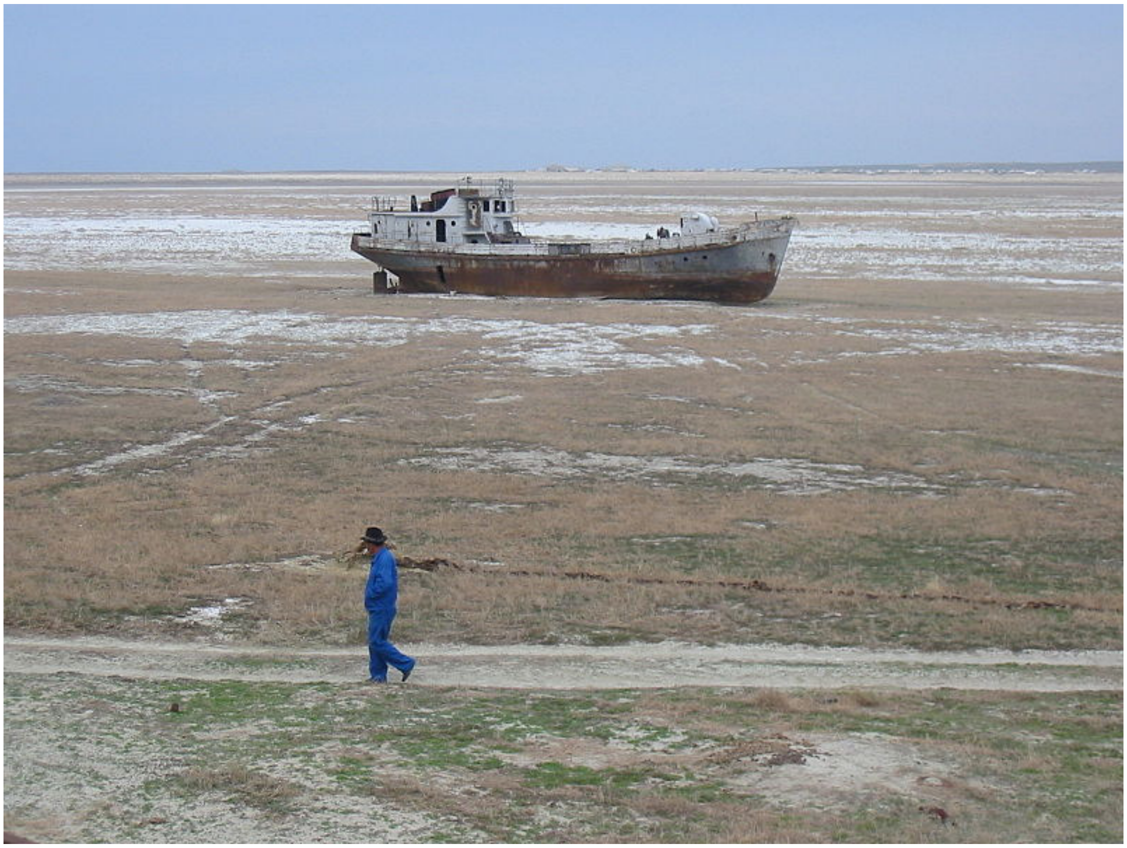

Figure S5. 4. Former bed of the Aral Sea transformed to a halophyte herbfield. Ecosystem collapse triggered economic ruin for shipping and fishing industries. Source: http://en.wikipedia.org/wiki/File:Aralship2.jpg

Ecosystem collapse

Much of the characteristic native biota recorded in the Aral Sea prior to 1960, including fish, zooplankton, maco-invertebrates and plants, has been eliminated from the ecosystem as their habitats became too dry or too saline to support persistence of viable populations (Micklin 1988, Micklin & Aladin 2008). The loss of biota, and hence collapse of the ecosystem, coincided with shrinkage of the water body and its fragmentation into several separate lakes (Fig. 5).

Thresholds of collapse were defined by examining the chronology of events. Aladin & Potts (1992) identified three periods of decline. The first occurred during 1960-68 when the volume of the sea declined by less than 10% and salinity increased marginally from 10 to 11 $g.l^{-1}$. Declines in the abundance of phytoplankton and native fish during this period were attributed primarily to introductions of plankton-eating copepods and fish in the 1960s, however, it appears that few if any native species were lost from the ecosystem (Aladin & Potts 1992).

The second period of decline occurred during 1970 -1975, when the sea volume declined by c. 20% and salinity increased from 11 to 14 $g.l^{-1}$ (Table 1). The decline in river inflows that caused these changes reduced influx of nutrients and hence productivity, causing a 3- to 5-fold reduction in phytoplankton biomass and a compositional shift from brackish and freshwater taxa to marine euryhaline taxa (Aladin & Potts 1992). At the beginning of this period the diversity of ostracods had declined from 11 to one species (Boomer et al. 1996),

and by the mid 1970s, benthic annelids declined from seven to one species, and the diversity of benthic arthropods declined from 27 to seven species (Williams & Aladin 1991). Salinisation and drying of spawning grounds in the shallow coastal waters initially affected juvenile fish, and by the mid 1970s had slowed growth rates, increased mortality and largely inhibited breeding in adults (Aladin & Potts 1992). Between 1976 and 1985, the Aral Sea biota was relatively stable (Aladin & Potts 1992), even though the sea volume declined by a further 45% and salinity rose from 14 to 22 14 $g.l^{-1}$ (Table 1) and commercial fishing had ceased by 1981.

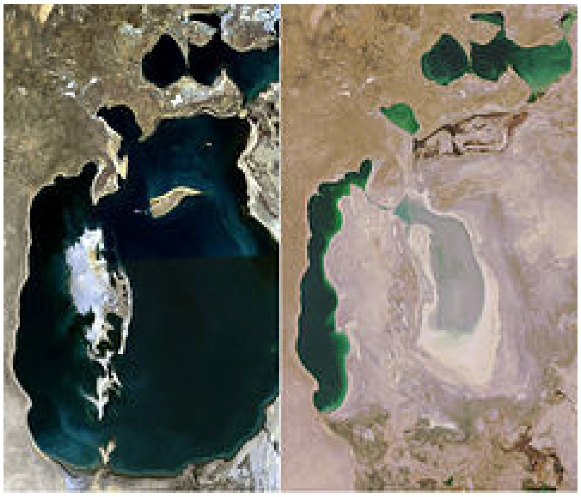

Figure S5. 5. Comparison of Aral Sea extent between 1985 (left) and 2011 (right) showing extensive drying and fragmentation of the water body. Source: http://en.wikipedia.org/wiki/File:Aral_Sea_1989-2008.jpg

The third period of decline occurred during 1987 - 1989, when the Aral Sea fragmented into two separate water bodies, a northern one fed by Syr Dar'ya and a southern one fed by Amu Dar'ya. By 1989, salinity had tripled to 30 $g.l^{-1}$, the total number fish species had declined from 32-35 (in 1960) to 10, with only four of the 20 native species remaining, and only 14 invertebrate fauna species were recorded (cf. 164 species in 1960).

Since 1990, the northern and southern water bodies have followed different trajectories (Micklin 2006). The southern water body, continued to decline in volume, with salinity reaching 100 $g.l^{-1}$ and all fish species disappearing by 2006 (Table 1). The northern waterbody received significant recharge due to high rainfall in the Syr Dar'ya catchment in 2002, and in 2005, a weir was constructed to regulate outflow, followed by another significant recharge event. This reduced salinity levels to 12 $g.l^{-1}$ by 2005 and 10 $g.l^{-1}$ by 2007 (Micklin & Aladin 2008). In response, numerous fish species recolonised the water body from refuges in the Syr Dar'ya and its delta wetlands, with the total number of species reaching 13 by 2005 and 16 by 2007, including fifteen of the twenty native species, albeit in different relative abundances.

From the above chronology, it was assumed that the Aral Sea ecosystem collapsed between 1976 and 1989 during either the second or third periods of decline described by Aladin & Potts (1992), as substantial losses of characteristic biota and changes in ecological character occurred during these times. The original brackish ecosystem was replaced by several novel ecosystems, including halophytic and xerophytic shrublands and herbfields on the dry sea bed, hypersaline lakes in the south and a new brackish ecosystem in the north comprising a

subset of the original native biota and some introduced biota. Collapse thresholds in distributional and functional variables were therefore set based on their values during 1976 - 1989. Thus, for assessing declines in distribution under criterion A, it was assumed that the ecosystem collapsed when sea surface area declined below 55,700 to 39,734 km^2.

For assessing environmental degradation under criterion C, it was assumed that the ecosystem collapsed when sea volume declined below 364 - 763 km^3 or when average salinity increased to 14 - 30 $g.l^{-1}$. Preliminary investigations based on analyses of ostracod assemblages and chemical analyses of molluscs and ostracod shells from two short cores and one section from the shores of the Aral Sea indicated that salinity levels of 30 $g.l^{-1}$ had not been attained during the period represented by the cores (Boomer et al. 1996), suggesting that these levels were outside the natural range of ecosystem variation.

For assessing disruption to biotic processes under criterion D, it was assumed that collapse occurred when the detectable number of originally native (i.e. 20 species in 1960, Aladin & Potts 1992) fish species fell below 4 – 10 species. The commercial catch of fish was assumed to be a proxy for total fish abundance, which had fallen to 10,000 tonnes by 1977 and zero by 1980. It was therefore assumed that ecosystem collapse was coincident with fisheries collapse. Although more data are available for fish than other taxa, Aladin (1995) studied the salinity tolerance of cladoceran crustaceans in the family Podonidae experimentally, showing that the upper limits of survival for the four Aral species varied from 26 to 30 $g.l^{-1}$. A collapse threshold was therefore set at 0-1 species in this group. Similarly, collapse thresholds could also be set for the number of detectable ostracods (1 species), benthic annelids (1 species) and benthic arthropods (4 – 7 species), as these groups also declined rapidly with salinisation of the sea.

ASSESSMENT

Summary

Criterion	A	B	C	D	E	overall
subcriterion 1	CO	VU	CO	CO	NE	CO
subcriterion 2	CO	LC	CO	CO		
subcriterion 3	CO	VU	CO	CO		

Criterion A

Current decline: Changes in the distribution of the Aral Sea were assessed using estimates of sea surface area from remote sensing (Fig. 6). Between 1960 and 2005, the surface area declined from 67,499 km^2 to 17, 382 km^2, passing the threshold of collapse (39,734 - 55,700 km^2) during 1976 - 1989. The status of the ecosystem under criterion A1 is therefore Collapsed.

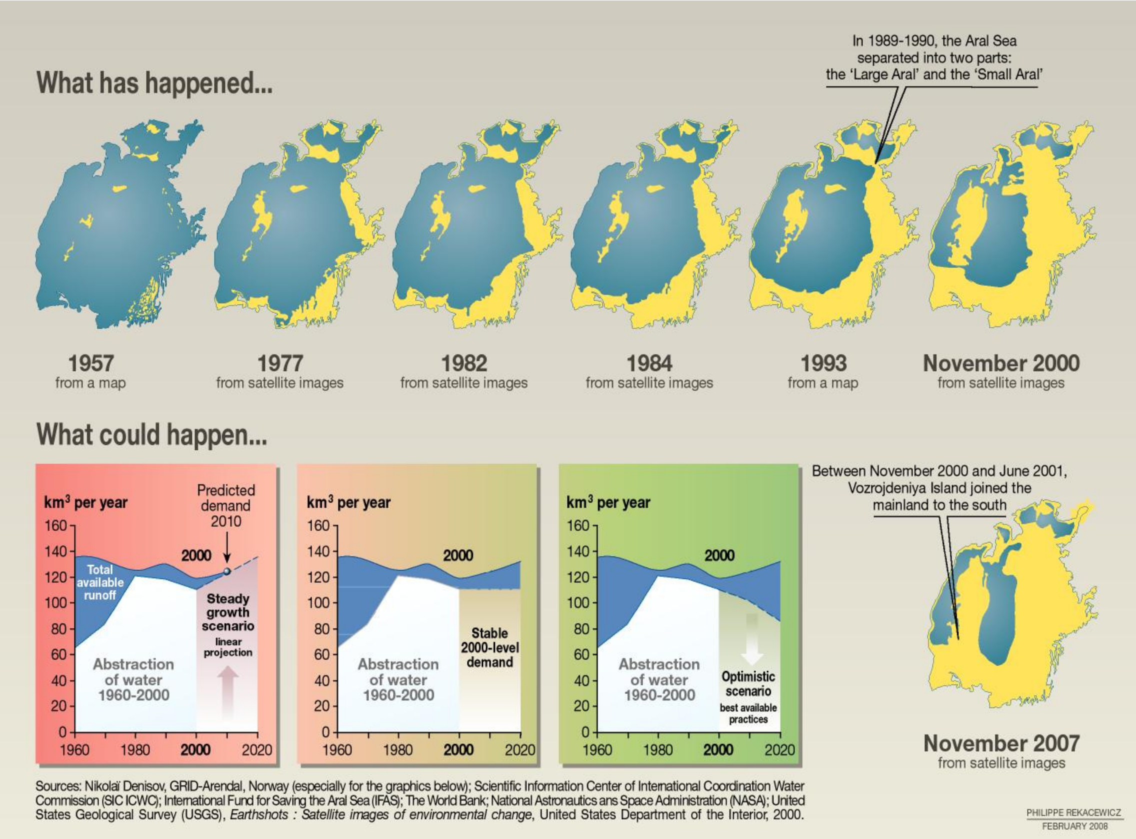

Figure S5. 6. Changes in surface area of the Aral Sea over 50 years 1957 to 2007. Source: UNEP (2008).

Future decline: Various projections exist for a range of hydrological restoration scenarios for the Aral Sea (Aladin et al. 2005). Hydrological restoration of the northern water body is considered feasible, assuming a modest inflow of 7 $km^3.yr^{-1}$ from the Syr Dar'ya and regulation of outflow by a dam constructed on Berg's Strait, which connects the northern and southern water bodies (Aladin et al. 2005). However, reversal of collapse by increasing water surface area above the collapse threshold is unlikely for two reasons. Firstly, the northern water body accounts for only a small fraction of the total area of the former sea, even when completely full it covers 5650 km^2 (Aladin et al. 2005), some 34,000 km^2 less than the lower bound of the collapse threshold. Hence, to exceed the collapse threshold, a large quantity of additional water would also need to be maintained in the southern water body. This is considered implausible, given the increased size of the regional population within the Amu Dar'ya catchment and the higher evaporation rates under a warming climate, compared to the pre-collapse era (Boomer et al. 2000; Aladin et al. 2005; Micklin 2006; Micklin & Aladin 2008). Secondly, even though water may be restored to the northern water body, the evidence supports its interpretation as a novel ecosystem, given that only a subset of the pre-collapse native biota are returning due to extinctions (Micklin 2006), that these are so present in different proportions and that the re-establishing biota includes species that were not present in the native biota of the original system (Aladin & Potts 1992; Micklin 2006). The status of the Aral Sea ecosystem under criterion A2 is Collapsed.

Historic decline: The Aral Sea was hydrologically stable since at least the mid eighteenth century (Bortnik 1996). Hence the decline in distribution over the historical time scale is the

same as the decline over the past 50 years, exceeding the threshold of ecosystem collapse. The status of the ecosystem under criterion A3 is therefore Collapsed.

Criterion B

Extent of occurrence: The current distribution of the Aral Sea based on the surface area of open water depends on the status of the southern water body. In recent years, the eastern arm of this water body has either been filled with a shallow depth or water or dry. The extent of occurrence of the Aral Sea was estimated as 25,000 - 40,000 km^2, based on two minimum convex polygons enclosing all occurrences, respectively assuming the eastern arm was filled with water (upper bound) and assuming it was dry (lower bound). There are continuing declines in distribution (see criterion A), plausible threats and the sea occupies 1 - 2 locations, depending on whether the northern and southern water bodies are interpreted as independent locations. The status of the ecosystem is therefore Vulnerable under criterion B1a,b,c.

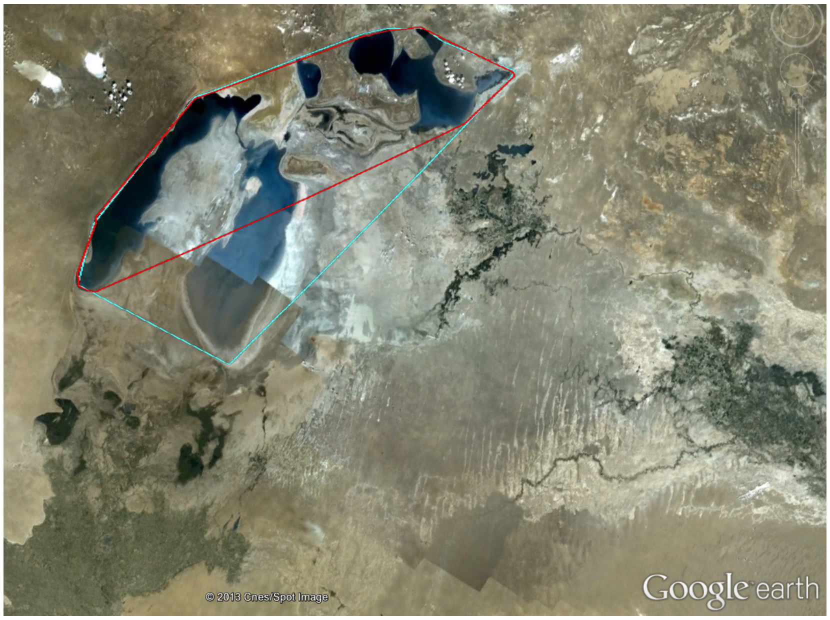

Figure S5. 7. Minimum convex polygons representing lower (red) and upper (blue) bounds of the current extent of occurrence of the Aral Sea surface waters.

Area of occupancy: Depending on whether the eastern arm of its southern water body is filled with water, the Aral Sea occupies 70 - 155 10 × 10 km grid cells. The status of the ecosystem is therefore Least Concern under criterion B2.

Number of Locations: The Aral Sea occupies 1 - 2 locations, depending on whether the northern and southern water bodies are interpreted as independent locations. The most severe plausible threat to the Aral Sea is continued drying and associated salinisation. The ecosystem is prone to severe threats, such as clearing and drought, such that is may become, or may already be Critically Endangered. It therefore qualifies for Vulnerable status under criterion B3.

Criterion C

Current decline: Sea volume and average salinity were identified as appropriate variables for assessing environmental degradation under criterion C. Total sea volume fell below the bounded collapse threshold between 1976 and 1989, and continued to decline in subsequent years (Fig. 8). Average salinity exceeded the collapse threshold during the same time. After

fragmentation of the sea, the salinity of the southern water body continued to increase, while the salinity of the northern water body declined, eventually below the collapse threshold (Fig. 9). However, at this stage, both the northern and southern water bodies were interpreted as novel ecosystems, and the data for the northern water body this were not interpreted as a reversal of collapse or a fluctuation in salinity for reasons discussed under criterion A. In addition, the salinity levels recorded in 1990 are higher than any historical values recorded in the history of sea sediments (Boomer et al. 2000), and therefore outside the natural range of variation in the ecosystem. The extent of degradation estimated by both sea volume and average salinity was 100% of the ecosystem distribution. The status of the ecosystem was therefore Collapsed under criterion C1.

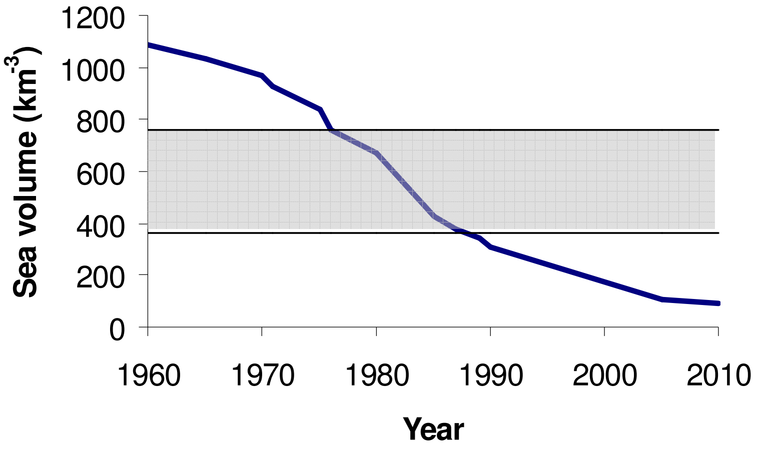

Figure S5. 8. Trends in total volume of the Aral Sea, relative to a bounded threshold of ecosystem collapse. Data compiled from Williams & Aladin (1991), Aladin (1995) and Micklin (2006).

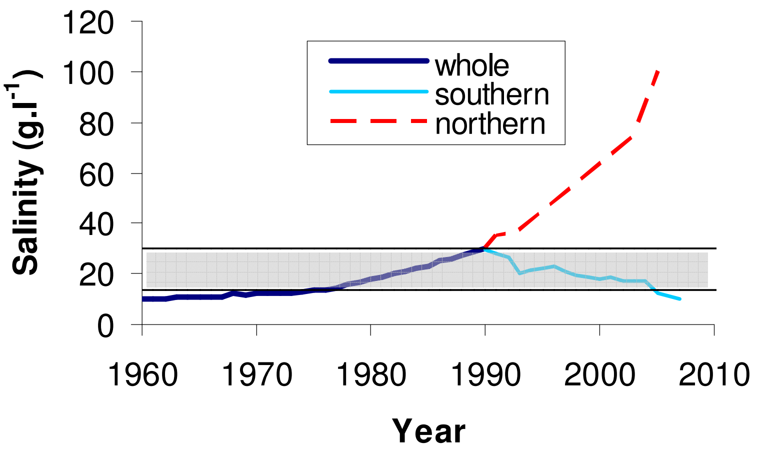

Figure S5. 9. Trends in average salinity of the Aral Sea, relative to a bounded threshold of ecosystem collapse. Data compiled from Aladin (1995), Aladin et al. (2005) and Micklin (2006).

<u>Future decline</u>: Various projections exist for a range of hydrological restoration scenarios for the Aral Sea (Aladin et al. 2005). For the southern water body, sea volume is projected to continue declining and salinity is projected to continue rising, possibly as high as 150-200 $g.l^{-1}$. For the northern water body, sea volume and salinity are projected to remain relatively stable, so long as inflows are maintains above 7 $km^3.yr^{-1}$ and outflows are regulated as described under

criterion A (Aladin et al. 2005). However, reversal of collapse by increasing sea volume or reducing salinity in the northern water body is unlikely for reasons discussed above under criterion A. The status of the ecosystem under criterion C2 is therefore Collapsed.

Historic decline: The Aral Sea was hydrologically stable since at least the mid eighteenth century (Bortnik 1996). Hence environmental degradation estimated from sea volume and salinity over the historical time scale is the same as the decline over the past 50 years, exceeding the thresholds of ecosystem collapse. The status of the ecosystem under criterion C3 is therefore Collapsed.

Criterion D

Current decline: Different biotic variables showed slightly different rates of decline. The commercial fish catch had ceased by 1981 (Fig. 10), although as many as 15 native species may have remained extant at that time (Fig. 11), albeit in low abundance and unable to reproduce. The number of native fish declined to four species by 1989 and only one remained in 1992. Although about 70% of the native fish fauna had recolonised the northern Aral water body from the delta of the Syr Dar'ya by 2007 (Fig. 11), the catch has recovered to only 5% of pre-decline levels and includes a substanital proportion of introduced species (Black Sea perch). The Southern water body remains without fish. The fisheries data therefore suggest that the ecosystem collapsed between 1981 and 1989.

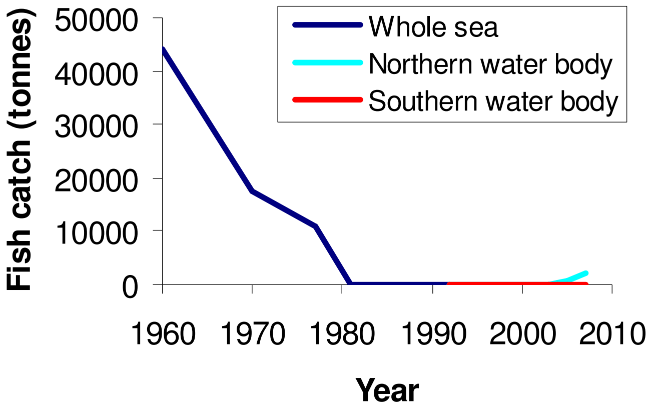

Figure S5. 10. Total commercial fish catch in the Aral Sea before and after its fragmentation into separate northern and southern water bodies. Threshold of ecosystem collpase assumed to be a catch of zero tonnes. Data compiled from Aladin & Potts (1992), Micklin (2006) and Micklin & Aladin (2008).

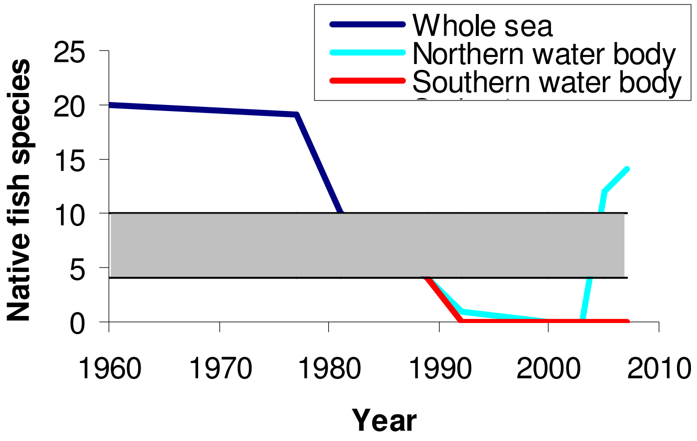

Figure S5. 11. Number of native fish in the Aral Sea before and after its fragmentation into separate northern and southern water bodies. Threshold of ecosystem collapse shown in grey. Data compiled from Aladin & Potts (1992), Micklin (2006) and Micklin & Aladin (2008).

The ostracod diversity had reached a collapse threshold of one species by 1970 (Boomer et al. 1996), while benthic annelids and arthropods had reached their thresholds of collapse (one and seven species, respectively) by 1976 (Williams & Aladin 1991). Podonod cladocerans had reached their threshold of collapse (one species) by 1989 (Aladin 1995). Collectively, the biotic data support the status of Collapsed under criterion D1 and suggest that collapse occurred during the period 1970 - 1989.

Future decline: There are no quantitative projections of future trends for any of the biotic variables. Although some further recolonisations and increases in fish catch are expected in the northern water body, some of the original biota is presumed extinct. The larger southern water body is expected to remain dry and hypersaline and hence unsuitable for the majority of the characteristic native biota of the former Aral Sea (Aladin et al. 2005, Micklin & Aladin 2008). Hence a reversal of ecosystem collapse is very unlikely during the next 50 years, even though biodiversity of the novel ecosystem in the northern water body may continue to increase if inflows are maintains above 7 $km^3.yr^{-1}$ and outflows are regulated as described under criterion A (Aladin et al. 2005). Hence, the status of the Aral Sea under criterion D2 is Collapsed.

Historic decline: The Aral Sea was relatively stable biologically prior to 1960 (Aladin & Potts 1992). Hence disruption to biotic processes and interactions estimated from the diversity of vertebrate and invertebrate taxa is the same over the historical time scale as the decline over the past 50 years, exceeding the thresholds of ecosystem collapse. The status of the ecosystem under criterion D3 is therefore Collapsed.

Criterion E

No quantitative analysis has been carried out to assess the risk of ecosystem collapse for Aral Sea. Hydrological models developed by Aladin et al. (2005) could potentially contribute to such an analysis. Until this work is done, the status of the ecosystem is therefore Data Deficient under criterion E.

REFERENCES

Aladin NV (1995) The conservation ecology of the Podonidae from the Caspian and Aral seas. Hydrobiologia 307: 85-97.

Aladin N, Cre´taux J-F, Plotnikov IS, Kouraev AV, Smurov AO, Cazenave A, Egorov AN, Papa F (2005) Modern hydro-biological state of the Small Aral sea. Environmetrics 16: 375–392.

Aladin NV, Plotnikov IS (1993) Large saline lakes of former USSR: a summary review. Hydvobiologia 267: 1-12.

Aladin NV, Potts WTW (1992) Changes in the Aral Sea ecosystems during the period 1960-1990. Hydrobiologia 237: 67-79.

Boomer I, Aladin N, Plotnikov I, Whatley R (2000) The palaeolimnology of the Aral Sea: a review. Quaternary Science Reviews 19: 1259-1278.

Boomer I, Whatley R, Aladin NV (1996) Aral Sea Ostracoda as environmental indicators. Lethaia 29: 77-85.

Bortnik, VN (1996) Changes in the Water-Level and Hydrological Balance of the Aral Sea. In: Micklin P, Williams WD, editors. The Aral Sea Basin (Proceedings of an Advanced Research Workshop, May 2–5, 1994, Tashkent, Uzbekistan). Heidelberg, Germany: Springer-Verlag, NATO ASI series, Vol. 12, pp 25–32.

Khan VM, Vilfanda RM, Zavialov PO (2004) Long-term variability of air temperature in the Aral sea region. Journal of Marine Systems 47: 25-33.

Micklin PP (2006) Desiccation of the Aral Sea: a water management disaster in the Soviet Union. Science 241: 1170-1176.

Micklin P (2006) The Aral Sea Crisis and Its Future: An Assessment in 2006. *Eurasian* Geography and Economics 47: 546-567.

Micklin P, Aladin NV (2008) Reclaiming the Aral sea. Scientific American : 64-71.

Williams WD, Aladin NV (1991) The Aral Sea: recent limnological changes and their conservation significance. Aquatic Conservation: Marine and Freshwater Ecosystems 1, 3-23.

6 EUROPEAN REEDBEDS

contributed by Brigitte Poulin, Tour du Valat Research Center, Le Sambuc, 13200 Arles, France.

CLASSIFICATION

International: In a strict sense, reedbeds refer to plant formations dominated by common reed *Phragmites australis*. Environmental perception of reedbeds varies according to continents and haplotypes (Marks et al. 1994; Güsewell & Klötzli 2000; Ludwig et al. 2003), as well as other biota associated with the dominant species. Hence, the reedbeds of Europe are regarded as a different ecosystem to those dominated by the same species on other continents.
National: Following the abundance and colonisation abilities of *Phragmites australis*, reedbeds have not been included in the European Habitat Directive. However, owing to their specific and vulnerable fauna, they are often listed as Wetlands of International Importance under the Ramsar convention or as Special Protection Areas under the European Union Bird Directive, being a priority habitat under the Biodiversity Action Plan in UK. In Europe, reedbeds are often referred to as a "priority species habitat."
IUCN Habitats Classification Scheme (Version 3.0): 13 Marine Coastal/Supratidal / 13.4 Coastal Brackish/Saline Lagoons/Marine Lakes

ECOSYSTEM DESCRIPTION

Characteristic native biota

Reedbeds are floristically impoverished ecosystems providing low niche diversity but high carrying capacity for wildlife. The dominant plant species, *Phragmites australis*, determines the structure of the system. It is often found in association with other species from the following plant genera: *Bolboschoenus, Carex, Glyceria, Juncus, Phalaris, Scirpus, Spartina* and *Typha*. These tall helophytes provide a sheltered and nutrient-rich habitat to various arthropods, birds and fish (Ward 1992; Hawke & Jose 1996; Okun & Mehner 2005; Self 2005; Valkamaa et al. 2008; White et al. 2006).

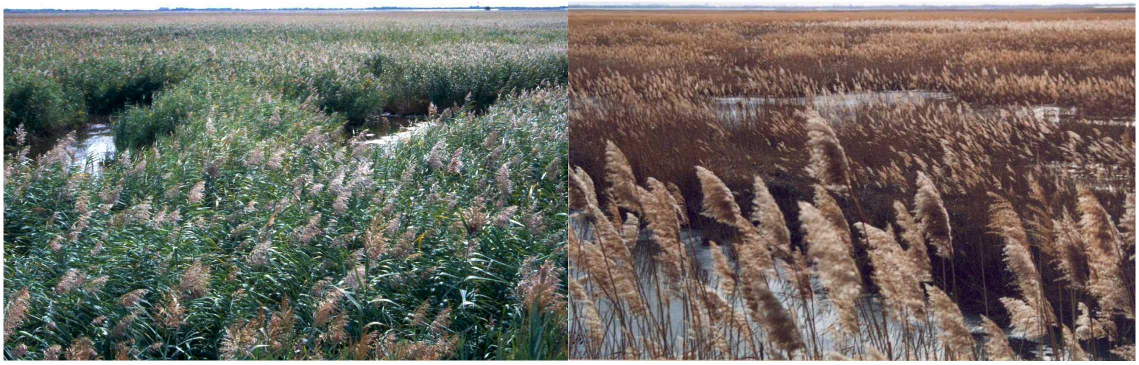

Figure S6. 1. Reedbeds in summer (left) and winter (right).

In Europe, reedbeds are the only or major breeding habitat of several vulnerable or endangered bird species at European level (eg., Eurasian bittern *Botauraus stellaris*, Purple heron *Ardea purpurea*, Little bittern *Ixobrychus minutus*, Moustached warbler *Acrocephalus melanopogon*), as well as the major migrating habitat of the globally vulnerable Aquatic warbler *Acrocephalus paliducola* (Provost et al. 2010). Several bird species further use predominantly reedbeds for nesting, feeding, resting and/or moulting during at least some part of the year (Bibby & Lunn 1982; Smiddy et al. 2007; Broyer & Calenge 2010).

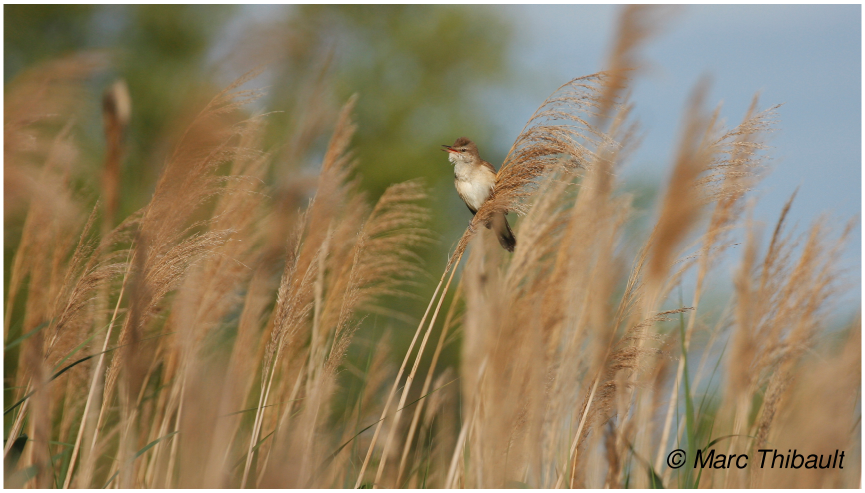

Figure S6. 2. A great reed warbler *Acrocephalus arundinaceus* in the Camargue.

Abiotic environment

Common reed can colonize a wide variety of permanent, semi-permanent and temporary wetlands: deltas, marshes, lake belts, edge of river and channels, roadsides and ditches. It is typically found in stagnant to slow-current fresh or brackish (0-22 ‰) shallow (0 – 1.5 m) waters. Factors limiting common reed occurrence and expansion are primarily water depth, current or waves, hypertrophic and hypersaline conditions (Engloner 2009). Under optimal conditions, common reed tends to form monospecific and productive stands.

Distribution

Present on all continents except Antarctica, common reed is probably the most widely distributed flowering plant on earth. The geographic distribution of Eurasian bitterns, a reed-specialist heron, provides a good approximation of reedbed distribution in Europe (Fig. 3).

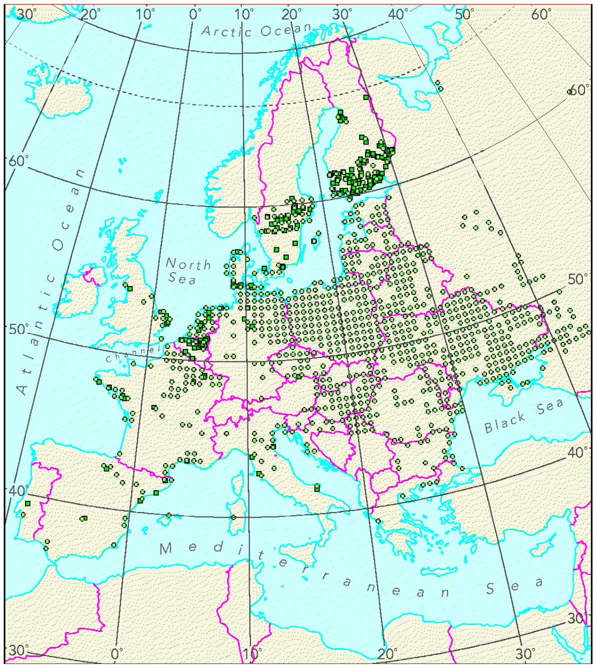

Figure S6. 3. Distribution of Eurasian bitterns (circles) and of Special Protection Areas (squares) in Europe. Source: http://www.eea.europa.eu/legal/copyright.

Key processes and interactions

Common reed may be established from seed, but spreads mainly by a rhizomatous root that extends both horizontally and vertically. The green growth of the plant provides the rhizome with food and oxygen during the summer. During the winter, standing dead stems bring oxygen to the rhizome. Reedbeds are characterized by a high primary productivity that permits rapid vegetative expansion and often lead to accumulation of organic matter and evolution towards woodlands. The presence of an autogenous soil made of rhizomatous roots and accumulated organic matter contributes to rising ground level or creates floating soils. The plastic morphology of reed stems and the capacity of rhizomes to accumulate reserves increase the plant's resistance to stress (Engloner 2009). Bacterial activity around the rhizomes, through aerobic and anaerobic processes, confers good properties to the plant for water purification (Chu & Zhang 2006; Stamati et al. 2010).

Threatening processes

Many reedbeds have been preserved or created because they provide services to humankind including fibre (roof thatch) and food (waterfowl hunting marshes, fishponds, pasture land), but also water purification, shoreline stabilization, water retention and flood control. The main cause of reedbed regression in the first part of the 19th century was land drainage and conversion for urban and agriculture development (Everett 1989; White et al. 2006). Main current threats are natural succession (terrestrialisation) processes that are not compensated by colonisation of new areas, stabilisation and rising of water levels translating into eutrophic and anoxic conditions, as well as increased salinity associated with sea level rise in coastal areas.

Main factors threatening the reedbed ecosystem in terms of geographic distribution, abiotic substrate, biotic interactions and reversibility are outlined in Table 1. Socioeconomic uses are not considered as threats, but their intensification can affect the ecosystem negatively and they were hence included in the table.

Ecosystem collapse

For assessment of criterion A and B, European reedbeds were assumed to have collapsed when their mapped distribution declines to zero, due to either replacement by development, such as agriculture, channelization or urban infrastructure, or by a terrestrial ecosystem through succession, rendering if unsuitable for the native characteristic biota. Flow regimes and levels of pollutants would be suitable variables for assessing abiotic degradation under criterion C if data were available. Abundance of reed-dependent birds and fish were identified as suitable variables for assessing disruption of biotic processes and under criterion D. Ecosystem collapse was conservatively assumed to occur if the abundance of these organisms declines to zero.

ASSESSMENT

Summary

Criterion	A	B	C	D	E	overall
subcriterion 1	VU	LC	DD	VU(VU-EN)	DD	VU(VU-EN)
subcriterion 2	DD	LC	DD	DD		
subcriterion 3	VU	LC	LC	LC		

Criterion A

Current decline. Declines of reedbeds have been reported in several European countries, such as UK (Bibby & Lunn 1982; Boar et al. 1989), Spain (Paracuellos 2008), Italy (Fogli et al. 2002), Germany (Sukopp & Markstein 1989; Kubín & Melzer 1997), Switzerland (Krumscheid et al. 1989), the Netherland (Graveland 1998), Hungary (van der Putten 1997), and the Czech Republic (Čížková et al. 1996; Šantrůčková et al. 2001) over the last few decades. Ostendorp (1995) reported reed dieback in 35 European lakes ranging from 18 to 94% (mean 53%). Although remotely-sensed techniques have recently been developed for mapping reedbed areas and ecosystem attributes (Davranche et al 2010; Poulin et al. 2010), information on reedbed loss is generally qualitative or restricted to local areas if quantitative. UK is one of the few countries providing an overall estimate of reed area (5000 ha at 900 sites) with 45% loss reported since 1945. In France, reed area has been estimated to 39 000 ha spread over 1048 sites (Le Barz et al. 2009), but data on reed regression is only indirect through the disappearance of Eurasian bitterns, a reed-specialist heron, from 29 out of 46 departments over the 1970-2008 period. A recent estimation of reedbed area by photo-interpretation in the Danube Delta, which encloses the largest European reedbed, reported 220 000 ha, which could represent a 22% decrease compared to previous reports of 284 000 ha. In Spain, a reported 56% loss in reedbeds from a small wetland in Almeria province due to agriculture (greenhouse) development is believed to be representative of the whole country, which has lost at least 60 % of its wetland surface, mainly coastal, over the last 200 years (Casado & Montes, 1995). In Germany, reedbeds are considered as vulnerable according to the National habitat red list (Riecken et al. 2006). In Austria, the largest reed area (lake Neusiedler See region) has increased from 1009 ha to 3016 ha between 1855 and 1993 following a stabilisation of the water table and cessation of extensive grazing (Kohler et al. 1994). However, this site is not considered as representative of the whole country which has lost 90% of its wetlands since the mid 19th century. Most of these estimates from different countries suggest reedbed losses of at least 30% over the past 50 years and a few exceed 50 % decline in distribution. Overall, the decline in distribution across Europe is therefore likely to be exceed 30%, but likely to be less than 50% over the past 50 years. Therefore the status of the ecosystem under criterion A1 is **Vulnerable.**

Future decline. Salt intrusion in coastal areas, litter build-up in continental areas, water pollution and eutrophication processes in large lakes and deltas are likely to continue in the next few decades, further contributing to reedbed regression in spite of increased conservation and protection measures. Water level stabilisation that prevents colonisation of new areas is also expected to continue, with reedbed restoration and creation not compensating for these losses owing to the small areas they involve. This could lead to a further decline of at least 30% over the next 50 years, however at present there are no quantitative estimates projecting future distribution and the status of the ecosystem is Data Deficient under criterion A2.

Historic decline. Land drainage and reclamation for agricultural and urban development has resulted in major wetland loss for most European countries since 1750. Although declines that occurred prior to the last 50 years are not well documented, reedbed destruction due to land drainage was the dominating threat before 1960, and estimates available for some countries (e.g. Austria) suggest substantial land drainage and reclamation since the mid 19th century, while in a number of countries (e.g. UK), the decline over the past 50 years alone is close to, or greater than 50%. Hence, the decline in distribution since 1750 is likely to be ≥ 50% and the status of the ecosystem under criterion A3 is **Vulnerable.**

Criterion B

Extent of occurrence. The ecosystem has an extent of occurrence encompassing Europe's total area (10,180,000 km^2), even though there is evidence of continuing decline and serious threats (Table 1). As the distribution of the ecosystem exceeds the thresholds for extent of occurrence, its status under criterion B1 is **Least Concern**.

Area of occupancy. Total reedbed area is estimated to be between 5000 and 10 000 km^2 in Europe. This area is dispersed widely across many wetlands. Area of occupancy (AOO) would therefore exceed 100 10 × 10 km grid cells, even excluding cells that contain small occurrences that cover less than 1% of cell area. The status of reedbeds under criterion B2 is thus **Least Concern.**

Locations. Reedbeds are naturally patchy ecosystems, colonising various habitat types and are found at many hydrologically independent locations. Hence their status under criterion B3 is Least Concern.

Criterion C

Current decline. Causes of degradation of the physical environment are diverse, involving reduced hydroperiod due to sediment/litter build up, decreased water quality resulting from industrial development, increased water eutrophication following agricultural development and stabilisation of water levels with reduced water flow. Embankment is a major large-scale problem threatening ecological functions of reedbeds. For instance, 59 dams were build along the first 1000 km of the River Danube for hydropower plants, with over 700 dams and weirs along its main tributaries. As a result, 100 000 ha of reedbeds were embanked for flood protection. In the Danube Delta the reduced flows and embankment prevent the refreshment of water in the limans (small estuary lagoons) by rising and falling water levels, reduces the filtering of silt, nutrients and pollutants in the Danube waters as well as the fertilization of the floodplain by floods. It also contributes to the salinisation of the nearby agricultural polders, and in combination with river dredging, halt key geomorphological processes (sedimentation) increasing risk of delta drowning by the sea. However, quantitative estimates of these processes are currently unavailable and the status of the ecosystem under criterion C2 is **Data Deficient**.

Future decline. Increased management and protection of remaining reedbeds, as well as improved water quality will hopefully refrain degradation of the physical environment in the future. Some 15% of the Danube Delta has been restored by partial dyke removal. However, increased salinisation caused by sea level rise is expected to threaten many coastal reedbeds in the next 50 years and more. Because quantitative projections of these processes are currently unavailable, the status of the ecosystem under criterion C2 is **Data Deficient**.

Historic decline. Reedbed destruction (land drainage) was the dominating threat before 1960. Factors affecting the physical environment of remaining reedbeds (embankment, intensification of uses, water pollution) had a lower extent and severity historically than over the past 50 years. Hence it is unlikely that the extent and severity of environmental degradation exceeds 70% since 1750 and the status of the ecosystem under criterion C3 is **Least Concern**.

Criterion D

Common reed is relatively tolerant to reduced flooding periods, embankment and water pollution. However, reduced quality and quantity of waters directly affect the aquatic flora

and fauna, which are crucial component of the food web. Shorter flooding period from June to December has been shown to reduce abundance of invertebrates and passerines the next spring (Poulin et al. 2002). Nesting birds are particularly sensitive to water level and a water shortage in early/late spring will translate into the desertion of breeding sites/lower breeding success of heron and duck species (Barbraud et al. 2002; Poulin et al. 2005). For example, Eurasian bitterns, a reed-specialist heron, disappeared from 29 out of 46 departments in southern France over the 1970-2008 period (Poulin et al. 2005). In this area, the complete disappearance of this key reedbed species represents a disruption to biotic interactions of 100% severity across 63% of the sampled extent. Embankment results in loss of habitat connectivity, reducing spawning areas for fish (Self 2005; Kallasvuo & Urho 2011). In the Danube Delta, a major centre of reedbed distribution, drainage, water regulation and pollution all had drastic effects on the waterbirds and fish, resulting in population decreases in 20 bird species (Schneider 1990), and the collapse of migratory anadromous sturgeons (*Huso huso, Acipenser güldenstaedti* and *Acipenser stellatus*) fisheries from 1000 tons/year at the beginning of the 20th century to 10 tons/year in 1990. Sedimentation and anoxia can further increase the impact of grazing by exogenous invasive mammals such as nutria *Myocastor coypus* (Boorman & Fuller 1981). Overall, the large declines in waterbird populations at two major centres of reedbed distribution, suggest a decline in biotic interactions with a relative severity of at least 50% over at least 50% of the extent of the ecosystem, with some biota declining by up to 90% in parts of the ecosystem distribution. The status of the ecosystem under criterion D1 is therefore **Vulnerable** (plausible range Vulnerable - Endangered).

Future decline. Disruption or decoupling of biotic interactions is not expected to decrease in the following 50 years. Salinisation of coastal reedbed will lead to a decrease in reed biomass and reduction in number of aquatic species. Intensification of reed exploitation is a likely scenario considering the increased demands for energy crop and renewable eco-material for green buildings. Overall decrease in bird and fish abundances could further lead to cascading effects (Tscharntke 1992; Mancinelli 2002; Xiong et al. 2010). However, quantitative projections of these processes are not currently available, and the status of the ecosystem under criterion D2 is **Data Deficient**.

Historic decline. Factors affecting biotic interactions, besides habitat destruction, had a lower extent and severity historically than over the past 50 years. Hence it is unlikely that the extent and severity of disruption to biotic interactions exceeds 70% since 1750 and the status of the ecosystem under criterion D3 is **Least Concern**.

Criterion E

No quantitative analysis has been carried out to assess the risk of ecosystem collapse for European reedbeds. The status of the ecosystem is therefore Data Deficient under criterion E.

REFERENCES

Barbraud C, Lepley M, Mathevet R, Mauchamp A. 2002. Reedbed selection and colony size of breeding purple herons *Ardea purpurea* in southern France. Ibis 144: 227-235.

Bibby CJ, Lunn J. 1982. Conservation of reed beds and their avifauna in England and Wales. Biological Conservation 23: 167-186.

Boar RR, Crook CE, Moss B. 1989. Regression of *Phragmites australis* reed-swamps and recent changes of water chemistry in the Norfolk Broadland, England. Aquatic Botany 35: 41-55.

Boorman LA, Fuller RM. 1981. The changing status of reedswamp in the Norfolk Broads. Journal of Applied Ecology 18: 241-269.

Broyer J, Calenge C. 2010. Influence of fish-farming management on duck breeding in French fish pond systems. Hydrobiologia 637: 173-185.

Casado S, Montes C, 1995. Guía de los lagos y humedales de España. J. M. Reyero, Madrid.

Chu WK; Wong MH; Zhang J. 2006. Accumulation, distribution and transformation of DDT and PCBs by *Phragmites australis* and *Oryza sativa* L.: I. Whole plant study. Environmental Geochemistry and Health 28: 159-168.

Čížková H, Strand JA, Lukavská J. 1996. Factors associated with reed decline in a eutrophic fishpond, Rožmberk (South Bohemia, Czech Republic). Folia Geobotanica & Phytotaxonomica 31: 73-84.

Davranche A, Lefebvre G, Poulin B. 2010. Wetland monitoring using classification trees and SPOT-5 seasonal time series. Remote Sensing of Environment 114: 552-562.

Engloner AI, 2009. Structure, growth dynamics and biomass of reed *(Phragmites australis)* – A review. Flora 204: 331-346.

Essl, F; Egger, G; Ellmauer Th. & Aigner, S. 2002. Rote Liste der gefährdeten Biotoptypen sterreichs: Konzept. UBA-Monographien, Umweltbundesamt, Wien.

Everett MJ. 1989. Reedbeds: a scarce habitat. RSPB Conservation Review 3: 14-19.

Fogli S, Marchesini R, Gerdol R. 2002. Reed *(Phragmites australis)* decline in a brackish wetland in Italy. Marine Environmental Research 53: 465-479.

Graveland J. 1998. Reed die-back, water level management and the decline of the Great Reed Warbler *Acrocephalus arundinaceus* in the Netherlands. Ardea 86: 187-201.

Güsewell S, Klötzli F. 2000. Assessment of aquatic and terrestrial reed *(Phragmites australis)* stands. Wetlands Ecology and Management 8: 367-373.

Hawke CJ, Jose PV. 1996. Reedbed management for commercial and wildlife interests, The Royal Society for the Protection of Birds, Sandy, UK.

Kallasvuo M, Lappalainen A, Urho L, 2011. Coastal reed belts as fish reproduction habitats. Boreal Environment Research 16: 1-14.

Kohler B, Rauer G, Wendeiln B. 1994. Landschaftswandel. In: Dick G, Dvorak M, Grüll A, Kohler B, Rauer G (eds.): Vogelparadies mit Zukunft? Ramsa-Gebiet Neusiedler See – Seewinkel. Wien: Umweltbundesamt: 21–34.

Krumscheid P, Stark H, Peintinger M. 1989. Decline of reed at Lake Constance (Obersee) since 1967 based on interpretations of aerial photographs. Aquatic Botany 35: 57-62.

Kubín P, Melzer A. 1997. Chronological relationship between eutrophication and reed decline in three lakes of Southern Germany. Folia Geobotanica & Phytotaxonomica 32: 15-23.

Le Barz C, Michas M, Fouque C. 2009. Les roselières en France métropolitaine : premier inventaire (1998-2008). Faune Sauvage 283 : 14-26.

Ludwig DE, Iannuzzi TJ, Esposito AN. 2003. Phragmites and environmental management: A question of values. Estuaries 26(2B): 624-630.

Mancinelli G, Costantini ML, Rossi L. 2002. Cascading effects of predatory fish exclusion on the detritus-based food web of a lake littoral zone (Lake Vico, central Italy). Oecologia 133: 402-411.

Marks M, Lapin B, Randall J. 1994. *Phragmites australis (p communis):* threats, management, and monitoring. Natural Areas Journal 14: 285-294.

Navodaru, I. 1998. Transition in the Danube Delta fisheries management. The World Bank/WBI's CBNRM Workshop. http://srdis.ciesin.org/cases/romania-002.html

Okun N, Mehner T. 2005. Distribution and feeding of juvenile fish on invertebrates in littoral reed *(Phragmites)* stands. Ecology of Freshwater Fish 14: 139-149.

Ostendorp W. 1989. Die-back of reeds in Europe - a critical review of literature. Aquatic Botany 35: 5-26.

Paracuellos M, 2008. Effects of long-term habitat fragmentation on a wetland bird community. Revue d'Écologie - Terre Vie 63: 227-238.

Poulin B, Lefebvre G, Mauchamp A. 2002. Habitat requirements of passerines and reedbed management in southern France. Biological Conservation 107: 315-325.

Poulin B, Lefebvre G, Mathevet R. 2005. Habitat selection by booming bitterns *Botaurus stellaris* in French Mediterranean reed-beds. Oryx 39: 265-274.

Poulin B, Davranche A, Lefebvre G. 2010. Ecological assessment of *Phragmites australis* wetlands using multi-season SPOT-5 scenes. Remote Sensing of Environment 114: 1602-1609.

Provost P, Kerbiriou C, Jiguet F. 2010. Foraging range and habitat use by Aquatic Warblers *Acrocephalus paludicola* during a fall migration stopover. Acta Ornithologica 45: 173-180.

Riecken U, Finck P, Raths U, Schröder E,, Ssymank A. 2006. Rote Liste der gefährdeten Biotoptypen Deutschlands. Zweite fortgeschriebene Fassung 2006. Naturshutz und Biologische Vielfalt Heft 34. Bonn: Bundesamt für Naturschutz.

Šantrůčková H, Picek T, Šimek M, Bauer V, Kopecký J, Pechar L, Lukavská J, Čížková H. 2001. Decomposition processes in soil of a healthy and a declining *Phragmites australis* stand. Aquatic Botany. 69: 217-234.

Schneider E. 1990. The wet grasslands in the catchment area of the lower Danube. International Wet-Pasture Symposium, Rastatt, 1988.

Self M, 2005. A review of management for fish and bitterns, *Botaurus stellaris*, in wetland reserves. Fisheries Management and Ecology 12: 387-394.

Smiddy P, Cullen C, O'Halloran J, 2007. Time of roosting of Barn Swallows *Hirundo rustica* at an Irish reedbed during autumn migration. Ringing and Migration 23: 228-230.

Stamati FE, Chalkias N, Moraetis D, Nikolaidis NP, 2010. Natural attenuation of nutrients in a mediterranean drainage canal. Journal of Environmental Monitoring 12: 164-171.

Sukopp H, Markstein B, 1989. Changes of the reed beds along the Berlin Havel 1962-1987. Aquatic Botany 35: 27-39.

Tscharntke T, 1992. Cascade effects among four trophic levels: bird predation on galls affects density-dependent parasitism. Ecology 73: 1689-1698.

Valkamaa E, Lyytinena S, Koricheva J, 2008. The impact of reed management on wildlife: a meta-analytical review of European studies. Biological Conservation 141: 364-374.

van der Putten WH, 1997. Die-back of *Phragmites australis* in European wetlands: an overview of the European research program on reed die-back and progression. Aquatic Botany 59: 263-275.

Ward D, 1992. Reedbeds for wildlife. Oxford: Royal Society for the Protection of birds, University of Bristol.

White G, Purps J, Alsbury S, 2006. The bittern in Europe: a guide to species and habitat management. Sandy: The RSPB.

Xiong L, Wu X, Lu JJ, 2010. Bird predation on concealed insects in a reed-dominated estuarine tidal marsh. Wetlands 30: 1203-1211.

Table S6. 1. Identification of major threats affecting the reedbed ecosystem with their relation to the Red List Criteria assessment

							Intensification of socioeconomic uses			
**Ecosystem threats**	**Drainage**	**Natural succession process**	**Xenobiotic water pollution**	**Increased water level or nutrient inputs**	**Sea level rise**	**Embankment**	**Fish farming**	**Waterfowl hunting**	**Grazing & summer mowing**	**Reed harvesting**
**Reduction in geographic distribution**	Reed stands replaced by agricultural land and urban fabric	Evolution of reedbeds towards woodland	Little effect on reed area owing to reed resistance to pollution	Reed regression leading to disappereance within one or two decades under severe conditions	Evolution of coastal marshes into lagoons with reedbed disapperance above 20 g/L surface water	Major perturbation increasing risks of agricultural conversion	Reduction of reed belt in fishpond	Increased open water areas at the expense of reed	Unsustainable practices reducing reed area and dominance	No reduction in reed area, reed tolerant to winter cutting, beneficial for refraining litter accumulation
**Degradation of abiotic substrate**	Complete loss of ecosystem substrate	Reduction in flooding period through litter/sediment build up	Poor water quality	Water eutrophication and anoxia, thick mud layer with undecomposed vegetal matter	Salinisation of surface/ground waters	Stabilisation of water levels, reduced water flow and oxygenation, reduced water quality (purification)	Reduced drawdown frequency, h increased eutrophication, absence of gradual slopes	Permanent flooding with increased water eutrophication	Compacted, less oxygenated ground for grazing and mechanical cutting	Soil compaction and risk of root damage if cutting engines not adapted to ground softness
**Disruption of biotic interactions**	Complete disruption of reedbed biotic interactions	Loss of aquatic organisms and of the reed-specific fauna	Reduced richness and abundance of aquatic organims and their predators	Reduced foraging and nesting opportunites for birds, impoverishment of the aquatic flora and fauna	Reduced reed biomass starting at 5 g /L, impoverishment of the invertebrate aquatic fauna	Decrease in reed density and vigor, reduction of spawning areas for fish.	Degradation of hydrophyte beds, reduced fish species & trophic guild richness with consequences on birds	Degradation of hydrophyte beds, reduction of foraging and nesting opportunities for nongame birds	Reed replaced by less palatable or summer-cutting tolerant species with loss of typical reed fauna	Loss of dry reed as refuge to the wintering fauna, reduced opportunities for breeding birds in spring
**Proxys**	Modification of land use	High ratio of dry to green stems, litter thickness, scrub encroachment.	Water quality	Presence of green algae, dark/ smelly water, reeds in tussocks with floating, unattached rhizomes	Surface and underground water salinity	Dyke	Vertical slopes of ponds, reed belt limited or absent	Artificially created open water areas, low richness of submerged macrophytes	Loss in reed height and multiplication of side-shoots before reed disappearance	Large areas with only green (growing) reeds in spring-summer
**Reversibility**	Irreversible, but partially compensated by reedbed creation in small areas	Irreversible without soil scraping, formally compensated by colonisation of new areas	Regulations expected to improve water quality	Reversible with summer drawdown	Locally reversible with improved management (freshwater input)	Restauration possible by dyke (partial) removal	Promotion of sustainable practices, bank reprofiling	Promotion of sustainable practices with periodical short drawdown and maintenance of a 50% reed area	Promotion of extensive grazing (0.5 cattle/ha) not followed by flooding, summer cutting in mosaics	Promotion of sustainable practices with maintenance of a mosaïc of harvested/non harvested patches

7 GONAKIER FORESTS OF SENEGAL RIVER FLOODPLAIN, SENEGAL AND MAURITANIA

contributed by David Keith[1], Taibou Ba[2] and Gray Tappan[3]
[1] Australian Wetlands, Rivers and Landscapes Centre, University of NSW; and NSW Office of Environment and Conservation, Australia.
[2] Centre de Suive Ecologique, Dakar, Senegal.
[3] United States Geological Survey, South Dakota, USA.

CLASSIFICATION

National: *Acacia nilotica* (Gonakier) forest was recognised as a distinctive vegetation type (Unit F4) by Stancioff et al. (1985) and is recognised in subsequent biodiversity surveys (CSE 2005). It is an important component within the Senegal River valley (Vallee du Fleuve Senegal) ecoregion (Tappan et al. 2004), which also includes shrub steppes and wetlands.
IUCN Habitats Classification Scheme (Version 3.0): 1. Forests / 1.5 Subtropical/Tropical Dry Forest.

ECOSYSTEM DESCRIPTION

Characteristic native biota

This unique forest ecosystem is dominated by *Acacia nilotica* (Gonakier) with *A. raddiana* and *A. seyal* (Fig. A.1). Other woody species are uncommon but include *Acacia albida, Adenololobus rufescens* (syn. *Piliostigma rufescens*), *Balanites aegyptiaca, Mitragyna inermis and Ziziphus mauritiana.* The understorey is generally sparse and grassy, with *Chloris plieurii, Dactyloctenium aegyptium* and forbs *Alysicarpus ovalifolius* and *Zornia glochidiata* (Stancioff et al. 1985). Gaps, created naturally or anthropogenically, are colonised by *Panicum anabatistum, Borreia verticellata* and *Vetiveria nigritana* (Stancioff et al. 1985). Due to regular inundation, the forest provides critical habitat for fish and migrating birds (Tappan et al. 2004). The diversity of woody species is comparatively low and the groundlayer flora is sparse and poorly documented (Fig. A.1). However, the structure, fluvial function and composition of the ecosystem is unique in the region and contrasts markedly with surrounding steppe and bare-earth ecosystems within the Senegal River valley ecoregion. Although *A. nilotica* does occur in other ecosystems, it is a less common member of more floristically diverse savannas and shrub steppes, and does not form a forest canopy.

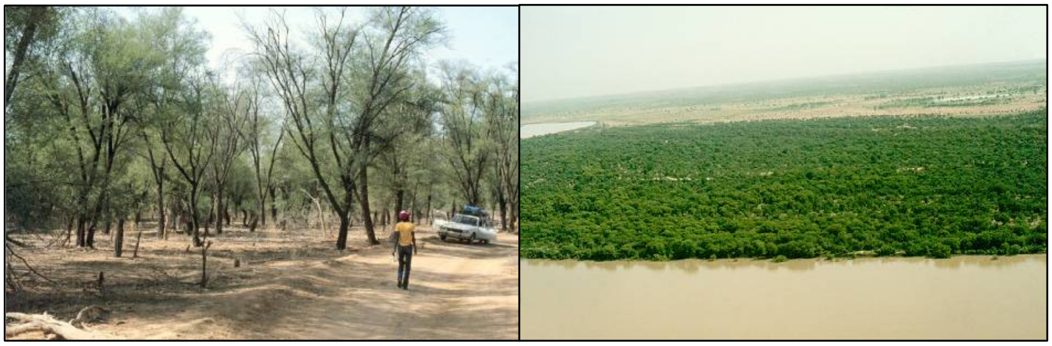

Figure A7.1. Gonakier forests of the Senegal River floodplain showing dominance of *Acacia nilotica* with sparse understorey (left) and aerial view of forest structure with Senegal River in foreground (right). Photos: Gray Tappan.

Abiotic environment

The ecosystem is restricted to a broad alluvial valley along the Senegal River comprising levees, flats and depressions subject to annual flooding (Tappan et al. 2004). The valley includes a mosaic of forest, savanna and steppe that is determined largely by the hydrological and pedological conditions of the floodplain. The soils supporting Gonakier forest are hydromorphic and vertic sandy clay loams and

clays and are inundated annually between July and November (Fig. A8.1). The Sahelian tropical climate has a seasonal rainfall pattern, with wet summers in June-August and dry winters. Mean annual rainfall in the 1990s varied from 150 to 600 mm along the riverine corridor (Tappan et al. 2004).

Distribution

Gonakier forests of the Senegal River floodplain are restricted to the riparian zone of the Senegal River from Dagana to Bakel, This area includes the border regions of northern Senegal, southern Mauritania and southeastern Mali (Fig. A8.4, A8.5).

Key processes and interactions

Gonakier forests of the Senegal River floodplain are typically inundated annually by wet season floods between July and November. Periodic floods are vital for sustaining forest structure and water-dependent biota in the arid and semi-arid landscapes through which the river flows (Fig. A8.2). The trees are able to tolerate periods of inundation of up to several months. Stancioff et al. (1985) describe the forest as a pseudo-climax community because it occurs at the end of a post-disturbance succession, although a change in flood regime will likely result in a new formation. Overbank flows also redistribute resources and disperse propagules of plants and aquatic biota. The precise mechanisms and dependencies of the ecosystem on flood regimes are uncertain, however Stancioff et al. (1985) noted drought induced declines in tree density during the 1970s and 1980s, when an absence of flooding in some stands lead to the highest tree mortality rates observed within the wooded vegetation formations of Senegal (Fig A8.2). In some cases, the forest has been replaced by steppe.

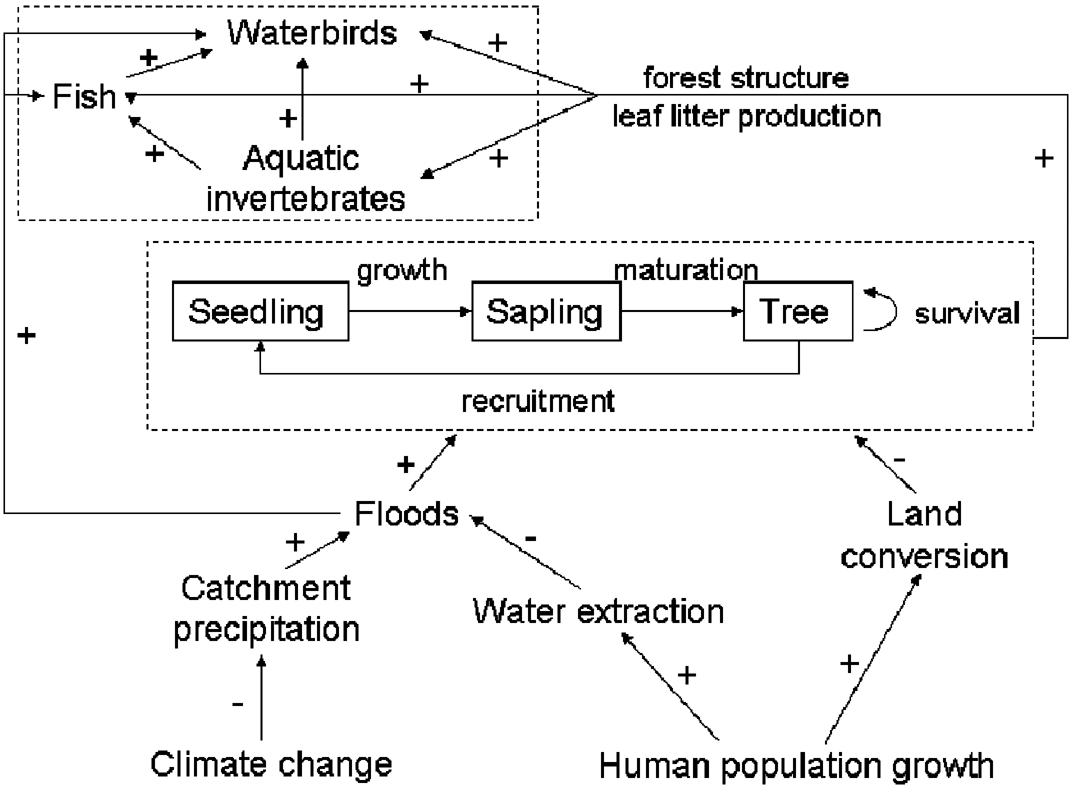

Figure A7.2. Cause-effect model of ecosystem dynamics for Gonakier Forests of Senegal River floodplain showing positive (+) and negative (-) environmental and anthropogenic infleunces on ecosystem processes and components.

The sparse structure of groundlayer suggests that grazing is not an important process in the ecosystem. The role of fire is poorly known, but the intact forests are unlikely to carry fire due to sparse ground fuels and annual inundation.

Threatening Processes

Change in land use (conversion of forest to agriculture) and changes to river flow regimes are the major current threats to Gonakier forests of the Senegal River floodplain (Fig. A8.2). Land clearing was initially associated with traditional subsistence agriculture, which increased gradually over several centuries (Fig. A8.3). Historically, clearing of the forest was carried out to make it more suitable for grazing by promoting development of the groundlayer with incursion of shrubs and grasses from the surrounding steppe (Stancioff et al. 1985). More recently, the land occupied by Gonakier forests has become an important resource for flood recession agriculture. In recent decades, extensive hydro-agricultural projects, designed to increase domestic food production and export production of rice and sugarcane, have resulted in more rapid transformation (Tappan et al. 2004). Cutting of wood for charcoal production, a major source of energy for local communities, exacerbates the trends driven primarily by expansion of irrigated agriculture (Tappan et al. 2004). These processes have resulted in a contraction of the distribution of the ecosystem, which may be considered to have collapsed when its area falls to zero. Twenty-eight patches of forest were designated as forest reserves by 1974 (Giffard, 1974), however, declines in distribution occurred despite these protected areas.

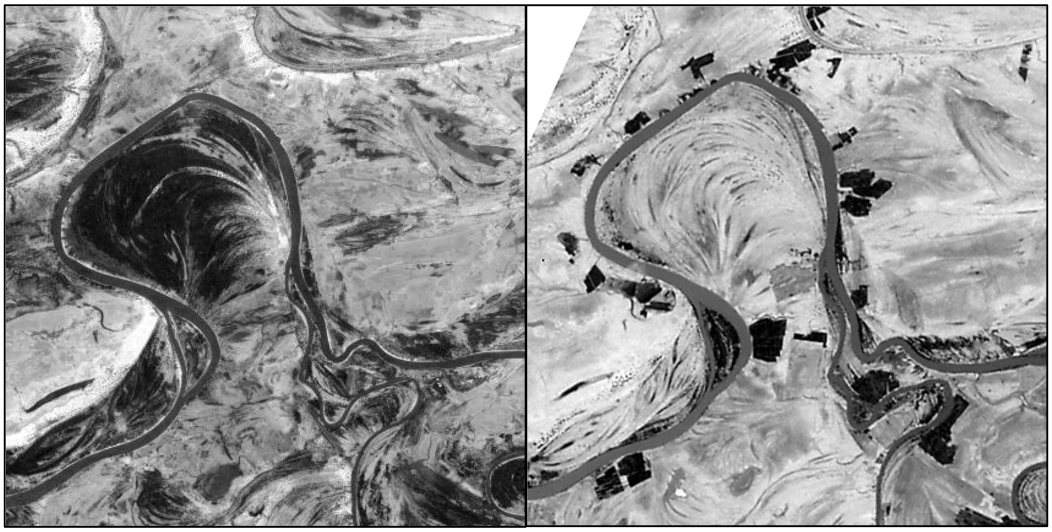

Figure A7.3**.** Conversion of Gonakier Forests of Senegal River floodplain to agricultural land use over the period from 1965 (left) to 1992 (right). Source: Tappan et al. (2004).

Changes in the flow regime of the Senegal River have occurred, both as a consequence of regional climate change and the construction of river regulation infrastructure (Fig. A8.2). The West African Sahel has experienced the most substantial and sustained decline in rainfall recorded in the world since rain gauge measurements began in the late 1800s (Nicholson 2000). This region includes the catchment of the Senegal River, which originates in Guinea and southern Mal (Fig. A.4)i, where mean annual precipitation exceeds 1400 mm (Hollis 1990), and flows through drier parts of Mali, Senegal and Mauritania, where precipitation is less than 250 mm.

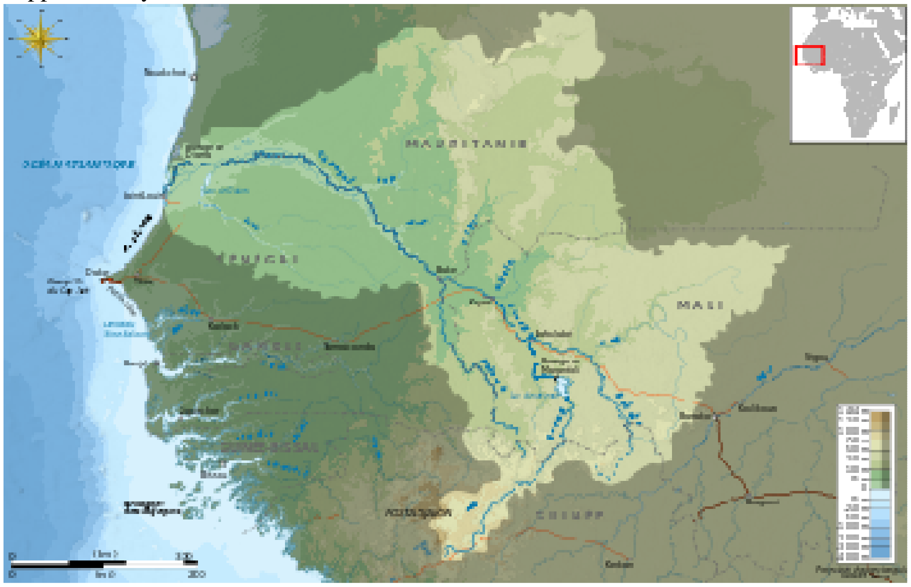

Figure A7.4. Catchment of the Senegal River. The major source of river water are forested areas in the south of the basin (Guinea and southern Mali), and the river flows through semi-arid and arid environments in the northwest (Senegal and Mauritania).

The entire catchment experienced declines in precipitation in the order of 10-50% during the twentieth century, with the greatest declines occurring in Senegal (Gonzalez et al. 2010; 2012). Inter-annual variation in Senegal's precipitation is closely (inversely) related to Sea Surface Temperatures in the Southern Atlantic Ocean (Fall et al. 2006a). Large and long cyclical variations in precipitation make trends difficult to detect over decadal time scales (Fall et al. 2006b). Although four extended regional droughts during the twentieth century were within extremes inferred from lake sediments spanning the past 2500 years (Shanahan et al. 2009), directional changes over the past 100 years in both precipitation and temperatures have been attributed to anthropogenic climate change (Held et al. 2005; Zhang et al. 2007; Gonzalez et al. 2012). In addition to climate change, Senegal's population has grown tenfold since 1900, increasing pressure on its limited arable land and water resources (Tappan et al. 2004; Fig. A8.2). Construction of the Manantali dam upstream in Mali in 1988 accelerated diversion of water for agriculture and reduction of flood volumes. Hollis (1990) estimated that 14.37 $km^3.year^{-1}$ of water would need to be extracted from the Senegal River and its tributaries for the region to achieve full irrigation potential, an area of 420,000 ha.

The relationship between flood occurrence and maximum river flows allows the conditions for ecosystem collapse to be estimated, given the dependence of Gonakier forest on flooding. It may be assumed that collapse was underway when extensive forest dieback was observed by Stancioff et al. (1985), and that this can be measured by the concurrent maximum river flows. For example, at Bakel the river height was continually below 800 cm between 1976 and 1986 when extreme rates of tree mortality were observed, whereas river height had fallen below that level once in the preceding 70 years (Fig. A8.6).

Ecosystem collapse

For assessment of criteria A and B, collapse was assumed to occur when the mapped distribution or projected suitable habitat declines to zero. Because this is a floodplain ecosystem threatened by water interception and climate change, we identified maximum river height as a proxy for overbank inundation to assess the relative severity of environmental degradation under criterion C. Conservatively, we assumed that the ecosystem will collapse when maximum river height falls to 0-10% of unregulated levels.

ASSESSMENT

Summary

Criterion	A	B	C	D	E	overall
subcriterion 1	CR	VU	VU	DD	DD	CR
subcriterion 2	DD	LC	DD	DD		
subcriterion 3	CR(EN-CR)	VU	DD	DD		

Criterion A

Current decline: Gonakier forests of Senegal River floodplain have a distinctive remote sensing signature (Fig. A8.3) that makes them readily distinguishable from the surrounding shrub steppe and other land cover types. Three independent temporal analyses of the distribution of Gonakier forests have been carried out for time periods during the past 50 years (Table A8.1). The first was a comparison of Corona and Landsat images captured in 1965 and 1992, respectively (Fig. A8.5), showing a 77% decline in mapped area (Tappan et al. 2004). The second was a comparison of two Landsat images captured in 1975 and 2000 showed a 72% decline in area (Tappan, unpubl. analysis). The annualised rates of decline from these studies are remarkably similar (Table A8.1), suggesting that rates of decline were roughly constant at 5% per year over a 35-year period 1965-2000 and that area estimates were insensitive to mapping methods and source of imagery. Assuming that rates of decline remained roughly constant for a 50-year period, extrapolation produces an estimate of 92-93% decline in distribution over the past 50 years (Table A8.1).

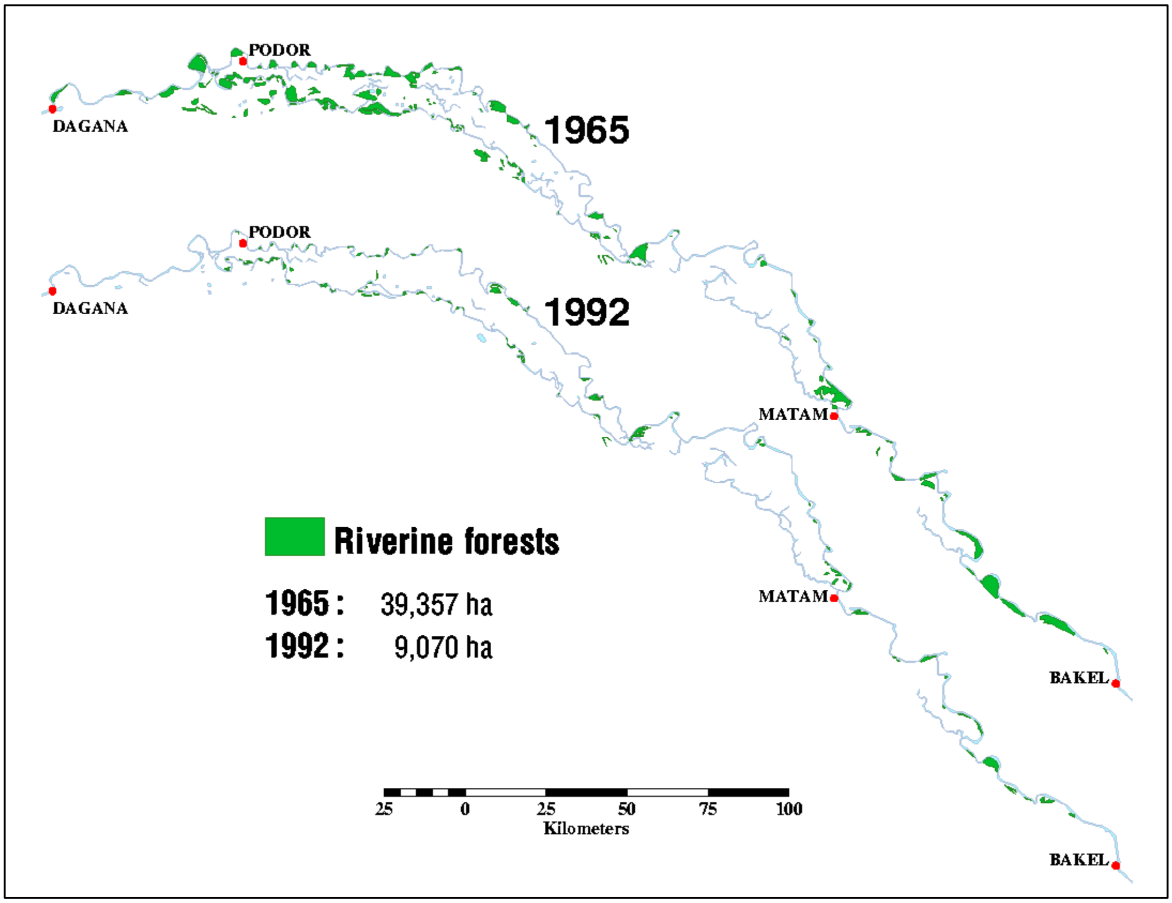

Figure A7.5. Change in distribution of Gonakier forest mapped from Corona imagery captured in 1965 and Lasndsat imagery captured in 1992 (adapted from Tappan et al. 2004).

Table A7.1. Estimated declines in distribution in distribution of Gonakier forests of Senegal River floodplain.

Year	Area (km^2)	% decline	Time frame (yrs)	Annual decay rate	Annual decline	Extrapolated decline over 50 years	Domain	Source
1965<br>1992	393.57<br>90.7	77.0%	27	-0.0544	5.29%	93%	National (Senegal only)	Tappan et al. (2004)
1975<br>2000	116<br>32	72.4%	25	-0.0515	5.02%	92%	National (Senegal only)	Tappan (unpubl. data)
1984<br>2003	1071.73<br>526.02	50.9%	19	-0.0375	3.68%	85%	Global (Senegal & Mauritania)	CSE (2005)

For both of these analysis, the spatial data were limited to the Senegal side of the border. A third analysis has been carried out on the global distribution of Gonakier forests encompassing both Senegal and Mauritania (CSE 2005). This comparison of Landsat images captured in 1984 and 2003 shows a decline in Gonakier forest area of 51% (Table 1). Although the time frame of comparison was slightly shorter, the annualised rate for decline was estimated to be 3.7%, suggesting slightly slower rates of loss on the Mauritania side of the river than in Senegal. Assuming the rate of decline was roughly constant in Mauritania, as it appears to be in Senegal (Tappan et al. 2004 cf. Tappan unpubl. analysis), extrapolation produces an estimated decline of 85% over the past 50 years. Based on a bounded estimate of 85-93% decline in distribution over the past 50 years, the status of Gonakier forests of Senegal River floodplain under criterion A1 is Critically Endangered.

Future decline: No projections are available for the future distribution of Gonakier forests of Senegal River floodplain. If declines continue at the current rate, the distribution may be expected to contract by a further 85 - 93%, however, this assumption may not hold true if protected areas prevent conversion of forests to agriculture. The status of the ecosystem is therefore Data Deficient under criterion A2.

Historic decline: The historic declines in Gonakier forests are likely to be greater than those estimated for the past 50 years. There is anecdotal evidence that agricultural expansion took place in Senegal at an increasing rate during the twentieth century. The national population increased tenfold during that period (Tappan et el. 2004) and continues to increase at 1 - 3 % per year (Gonzalez et al. 2012). Tappan et al. (2000) noted that eastern Senegal had been inhabited by people practicing subsistence agriculture for centuries and that most of the present day villages had been established by the 1930s when colonial agriculture was expanding at an increasing rate. In northwestern Senegal Gonzalez (2001) documented declines in forest species richness and tree density from 1945 to 1993. However, little of the historical information is specific to Gonakier forests of Senegal River floodplain, so it is difficult to estimate how much of the decline in the distribution of these forests took place prior to 1965, the earliest date of imagery used in published mapping studies (Tappan et al. 2004). Although further work is needed to map the historic distribution of Gonakier forests of Senegal River floodplain, the available evidence (Table S.1) suggests that the magnitude of in area decline must be at least 85-93%. The status of the ecosystem under criterion A3 is therefore at least Endangered and likely to be Critically Endangered.

Criterion B

Extent of occurrence: A minimum convex polygon enclosing all occurrences mapped by CSE (2005) from imagery captured in 2003 has an area of 22,307 km^2. There are continuing declines in distribution (see criterion A) and in a measure of environmental quality (see criterion C) of the ecosystem due to

reduced flows of the Senegal River, and there may be as few as two locations (see below). The status of the ecosystem is therefore Vulnerable under criterion B1.

Area of occupancy: Gonakier forests mapped from 2003 imagery occupied 151 10 x 10 km grid cells. Of these, 93 cells contained more than 1 km^2 Gonakier forest (i.e. more than 1% of their area). The status of the ecosystem is therefore Least Concern under criterion B2.

Number of Locations: Gonakier forests of Senegal River floodplain do not have a highly restricted area of occurrence (see above). The most severe plausible threat to Gonakier forest is currently land conversion. Senegal and Mauritania represent different socio-economic communities and apparently exhibit different rates of forest clearing (see criterion A). If rates of clearing are similar between village communities, there may be as few as two locations of the ecosystem. The ecosystem is prone to severe threats, such as clearing and drought, such that is may become, or may already be Critically Endangered. It therefore meets criterion B3 for Vulnerable status.

Criterion C

Current decline: Flooding is a key ecological process that sustains this ecosystem (Fig. A6.2). As floods occur only during the wet season months, the maximum annual river heights were assumed to be indicative of the river's capacity to flood each year. River height data were available for 100 years from 1904 to 2003 from four gauging stations within the distribution of Gonakier forests of Senegal River floodplain: Bakel; Matam; Podor; and Dagana (Fig. A6.5 for locations). To assess criterion C, we assumed that the mean annual maximum river height across these four gauging stations was a suitable proxy for environmental degradation. River flows declined sharply, reaching a minimum during the late 970s and 1980s. Hollis (1990) estimated that floods of 2500 m3/s needed for floodplain inundation would be very unlikely to occur based on river flows observed during 1986 -1989. Stancioff et al. (1985) observed extreme rates of tree mortality between the mid 1970s and the mid 1980s, corresponding to the lowest maximum river heights (473±-27 cm) observed during the 100 years of records (Fig. A6.6).

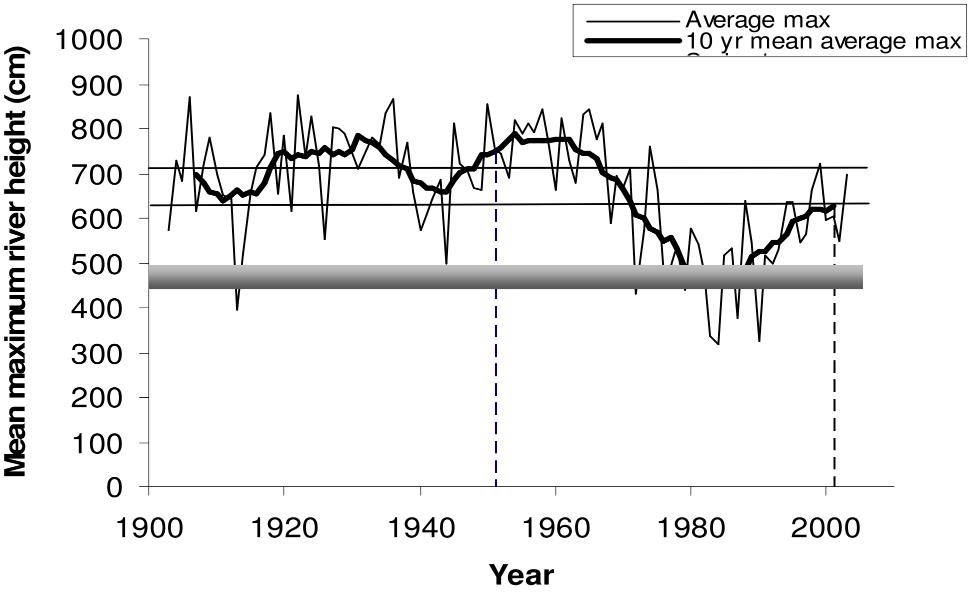

Figure A7.6. Trends in annual maximum river height averaged across four river gauge stations at Bakel, Matam. Podor and Dagana. Vertical broken lines show the past 50 years. Upper horizontal line shows mean maximum river height for 1904 - 1953, lower horizontal line shows mean maximum river height for 1954 - 2003, and horizontal grey bar shows assumed 50-year mean maximum flow that

would cause ecosystem collapse based on observed flows during 1976 - 1985. Data from Centre de Suive Ecologique, Dakar.

We therefore assumed that the ecosystem would collapse (due to extensive tree mortality) when the mean maximum river height for a 50 year period fell below 450 - 500 cm. To calculate the relative severity of hydrological decline, we first calculated the difference between mean maximum river height between the periods 1904 - 1953 and 1954 - 2003. We then divided this by the difference between mean maximum river height during 1904 - 1953 and the assumed mean height assumed at collapse. Relative severity of hydrological decline over the past 50 years was therefore between 100×(712-619)/(712-450) = 35% and 100×(712-619)/(712-500) = 44%. The status of the ecosystem was therefore Vulnerable under criterion C1.

Future decline: The are currently no future projections of flows of the Senegal River. The status of the ecosystem under criterion C2 is therefore Data Deficient.

Historic decline: Available historic data suggest regional declines in precipitation have been ongoing since records began in the late 1800s (Nicholson 2000). For example mean annual precipitation at St Louis at the mouth of Senegal River declined from 450 mm in 1855 to 240 mm in 2009, while twentieth century declines in precipitation were estimated to vary between 10 and 50% across the Senegal River catchment (Gonzalez et al. 2012). The impact of these changes on flows in the Senegal River are uncertain. For example, maximum river height showed no obvious trend between 1904 and the mid 1950s (Fig. A8.6) when precipitation was declining. As data on river flows are lacking over a longer historic time frame, the status of the ecosystem is Data Deficient under criterion C3.

Criterion D

Tree dieback and mortality is one of the biological outcomes of the degradation processes described above and in Fig. A.2. Changes in tree density of cover would therefore be suitable proxy variables for biotic degradation. Gonzalez e al. (2012) found that tree density declined significantly from 1954 to 2002 in the western Sahel at Njóobéen Mbataar and Fété Olé, in northern Senegal and that tree species richness declined significantly from 1960 to 2000 across the Sahel in a sample of 14 village areas in Mauritania, Mali, Burkina Faso, Niger, and Chad. They attributed tree decline to two forms of climate variability: increasing temperature and decreasing precipitation, which were jointly attributable to global climate change (Gonzalez et al. 2012). There is also independent evidence of declines in tree density and species richness across the northern and central Senegal savannas. Unfortunately, there are insufficient data available specifically for Gonakier forests of Senegal River floodplain to draw reliable inferences about changes in tree density and species richness within that ecosystem. The status of the ecosystem under criterion D is therefore Data Deficient.

Criterion E

No quantitative analysis has been carried out to assess the risk of ecosystem collapse for Gonakier forests of Senegal River floodplain. The status of the ecosystem is therefore Data Deficient under criterion E.

REFERENCES

CSE (2005) Projet Biodiversité Mauritanie-Sénagal: caractérisation de la zone et des sites du projet, 230p. Projet-RAF/98/G31. Centre de Suive Ecologique, Dakar.

Fall S, Semazzi FHM, Niyogi DDS, Anyah RO, Bowden J. (2006a) The spatiotemporal climate variability over Senegal and its relationship to global climate. *International Journal of Climatology* **26**: 2057-2076

Fall S, Niyogi DDS, Semazzi FHM (2006b) Analysis of Mean Climate Conditions in Senegal (1971–98). *Earth Interactions* **10**: 1-40. (doi:http://dx.doi.org/10.1175/EI158.1)].

Giffard P. (1974) L'Arbre dans le paysage senegalais, Dakar.

Gonzalez P. (2001) Desertification and a shift of forest species in the West African Sahel. *Climate Research* **17**: 217-228.

Gonzalez P, Neilson RP, Lenihan JM, Drapek RJ. (2010) Global patterns in the vulnerability of ecosystems to vegetation shifts due to climate change. *Global Ecology and Biogeography* **19**: 755-768.

Gonzalez P, Tucker CJ, Sy H. (2012) Tree density and species decline in the African Sahel attributable to climate. *Journal of Arid Environments* **78**: 55-64.

Held IM, Delworth TL, Lu J, Findell KL, Knutson TR. (2005) Simulation of Sahel drought in the 20th and 21st centuries. *Proceedings of the National Academy of Sciences of the United States of America* **102**, 17891-17896.

Hollis GE. (1990) Senegal River Basin Monitoring Activity. Hydrological Issues: Part II. Working Paper No. 57. Institute for Development Anthropology. Binghamton, USA.

Nicholson S. (2000) Land surface processes and Sahel climate. *Reviews of Geophysics* **38**: 117–139.

Shanahan TM, Overpeck JT, Anchukaitis KJ, Beck JW, Cole JE, Dettman DL, Peck JA, Scholz CA, King, JW. (2009) Atlantic forcing of persistent drought in West Africa. *Science* **324**: 377-380.

Stancioff, A. S, Staljanssens M, Tappan G. (1985) Mapping and remote sensing of the resources of the Republic of Senegal: A study of the geology, hydrology, soils, vegetation and land use potential. South Dakota State University. Brookings, USA.

Tappan GG, Hadj A, Wood EC, Lietzow RW. (2004) Use of Argon, Corona, and Landsat Imagery to Assess 30 Years of Land Resource Changes in West-Central Senegal. *Photogrammetric Engineering & Remote Sensing* **66**: 727-735.

Tappan GG, Sall M, Wood EC, Cushing M. (2004) Ecoregions and land cover trends in Senegal. *Journal of Arid Environments* **59**: 427–462.

Zhang X, Zwiers FW, Hegerl GC, Lambert FH, Gillett NP, Solomon S, Stott PA, Nozawa T. (2007) Detection of human influence on twentieth-century precipitation trends. *Nature* **448**: 461-465.

8 THE FLOODPLAIN ECOSYSTEM OF RIVER RED GUM AND BLACK BOX, SOUTH-EASTERN AUSTRALIA

Contributed by [1]Ralph Mac Nally, [1]Shaun C Cunningham & [2]Graeme Newell

[1]Australian Centre for Biodiversity, Monash University
Arthur Rylah Research Institute, Victorian Department of Sustainability & [2]Environment

CLASSIFICATION

National/State: Vegetation classes in New South Wales - Inland Riverine Forests (Keith 2004).
IUCN Habitats Classification Scheme: 5. Wetlands / 5.1 Permanent Rivers, Streams, Creeks
Biogeographic realm: Australasia

ECOSYSTEM DESCRIPTION

Characteristic native biota

The river red gum (*Eucalyptus camaldulensis* Dehnh.) and black box (*Eucalyptus largiflorens* F.Muell.) are the dominant tree species on the floodplains of the Murray-Darling Basin, which at 1.1 million km^2 is Australia's largest river system. These species occur on the same floodplains but dominate areas with different soil types and elevations and, therefore, differing water availabilities. The biomass of these forests often is similar to forests in high rainfall areas (ca 1200 mm yr^{-1}) (Bren 1990), which, given the aridity, especially to the west, suggests a strong dependence on overbank flows from upstream and on ground water (Cunningham et al. 2011b). Forest structure ranges from closed to open forests in the east (10-30 m tall, 30-45% projective foliage cover) to woodlands further west (10-30 m tall, 20-25% projective foliage cover), with an increasing proportion of woodlands in the semi-arid west (Specht 1981). There are shrubby, sedgy and grassy understoreys, with Asteraceae (daisies), Poaceae (grasses) and Chenopodiaceae (saltbushes) being common plant families (Margules et al. 1990). The fauna shares many species with other vegetation types, but the westerly extension of the floodplain forests into increasingly arid conditions means that species that are characteristic of mesic and even humid areas appear much further west than would be expected otherwise (Tzaros 2001). The yellow rosella is a subspecies of the crimson rosella (*Platycercus elegans flaveolus*) that appears only in these floodplain forests. The superb parrot (*Polytelis swainsonii*) is a Vulnerable species that depends on hollows of large floodplain trees for breeding. The yellow-footed antechinus (*Antechinus flavipes*) is a numerically important native dasyurid marsupial, being much more common in floodplain forests than in nearby plains and upland vegetation types (Lada et al. 2008).

Abiotic environment

The Murray River floodplain covers a wide range of annual precipitation from medium rainfall at Albury in the east (715 mm yr^{-1}) to semi-arid at Renmark further west (250 mm yr^{-1}). Historically, river red gum forests were flooded more regularly (45 up to 90 yr century^{-1}) than black box woodlands (10 to 50 yr century^{-1}) (Roberts 2004). Average summer temperatures range from 14 to 31°C and average winter temperatures range from 3 to 13°C (VEAC 2008). We focus on the Murray River corridor and its proximate distributaries, and on other major tributaries (e.g. Murrumbidgee, Lachlan, Ovens & Campaspe rivers) supporting similar systems.

Distribution

The Floodplain Ecosystem occupies principally the inundation zones of Order 4 to Order 7 rivers of the southern Murray-Darling Basin (Figure 1).

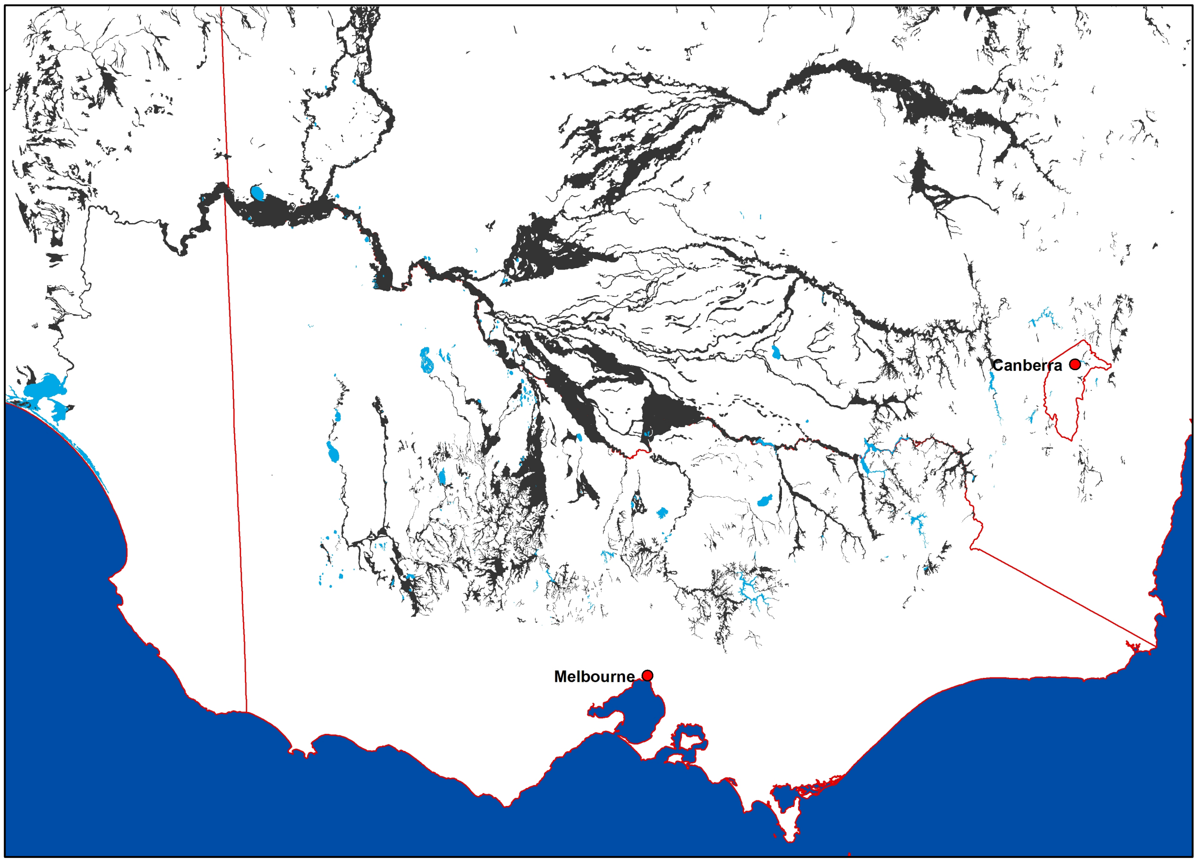

Figure S8. 1. Floodplains that encompass the probable original distribution of floodplain ecosystem in the southern Murray-Darling Basin (black). Blue denotes lakes and waterways.

Ecological dynamical model of the Floodplain Ecosystem and its threatening processes

We present a model of the factors affecting the ecological condition of the Floodplain Ecosystem (Fig. 2), which is a distillation of work done over the past 25 years (Mac Nally et al. 2011). The Murray River is Australia's longest river (2530 km), its water is used for extensive irrigation farming, domestic consumption, power generation and native ecosystems. Increasing regulation since the 1920s, through dams, weirs, levees and diversions, has greatly altered the flow regime. Between 1930 and 1991, mean annual water extraction from the Murray River increased by 73%, reducing the natural mean annual flow by 50% (Thomson 1992). Average peak monthly flows have been reduced by more than 50% along the Murray River (Maheshwari et al. 1995). The seasonal distribution of flows has shifted from winter-spring to summer-autumn since the construction of Hume Dam in the 1930s (Dexter et al. 1986).

In the Middle Murray, regulation has reduced extensive flooding in the Barmah Forest from 46% of years to 25% of years (Bren et al. 1987). This reduction has been more pronounced in the Mallee (a semi-arid region in the west dominated by multi-stemmed eucalypts), with the frequency of extensive floods on Wallpolla Island and Lindsay Island having been reduced from 44% of years to 16% of years (SKM and Roberts 2003). The Murray mouth stopped flowing in 2002, with barrages erected to arrest saline inflows from the ocean; it only began flowing again in 2010. Over the last two decades, the

Murray River floodplain has experienced two extended periods of below-average rainfall (1991-1995, 2001-2010) with record low inflows especially in 2006 (Cai and Cowan 2008b).

Consistent with the decrease in flooding frequency, surveys of forests on the Murray River floodplain have shown a substantial deterioration in tree condition over the past 20 or more years. By the late 1980s, degradation of tree canopies had increased dramatically below the Wakool Junction in the Mallee (Margules et al. 1990). A survey between Wentworth and Renmark found 52% of trees to be stressed or dead in 2002 (Resource_and_Environmental_Management 2003), and when these sites were resurveyed in 2004, the proportion of trees that were stressed had increased to 78% (MDBC 2005). In 2006, the condition of river red gum stands was mapped across the Victorian Murray River floodplain and 70% of these forests were in a stressed condition (Cunningham et al. 2009). These recent rapid declines in tree condition are likely to be the consequence of both protracted low rainfall, and extensive river regulation and water extraction (Figure 2).

Much of the biota is adapted to flooding pulses, with population dynamics highly dependent on the extent, duration and timing of inundation. The most significant human intervention is the modification of river flows, and the reduction in the frequency, duration and timing of flooding (Kingsford 2000), which are compounded by climate change (higher temperatures and reduced rainfall) and deteriorating groundwater conditions (depth and salinity) (Cunningham et al. 2011b). The lack of extensive flooding has led to widespread tree dieback across the Murray River floodplain (currently 79% by area) (Cunningham et al. 2009).

Since European settlement (1788), the lowland areas of the Murray-Darling Basin have been extensively cleared for agriculture and remnant forests remain heavily harvested for timber (Walker et al. 1993). A consequence is extensive secondary salinization both across the plains landscapes that surround the floodplains and also of waters flowing into the major rivers (Bennetts et al. 2006). The Murray-Darling Basin has highly variable rainfall and high evaporation, which leads to saline groundwater (Gee and Hillel 1988). Impoundments maintain elevated river levels and elevate groundwater levels upstream of them (Jolly 1996). Groundwater extraction increases groundwater depth. Reductions in flooding have resulted in a higher dependence of trees on groundwater, which has lowered further the groundwater; this has caused the salinization of soils in areas underlain by saline groundwater (Jolly 1996). Riverine systems have received vast amounts of salt from upland areas and the plains, which are deposited in high-order rivers such as the Murray River. River regulation has prevented the flushing flows that would leach salts from floodplain soils (Overton et al. 2006). This is particularly important in areas of drying climates, where reduced rainfall is insufficient to leach salts from root zones.

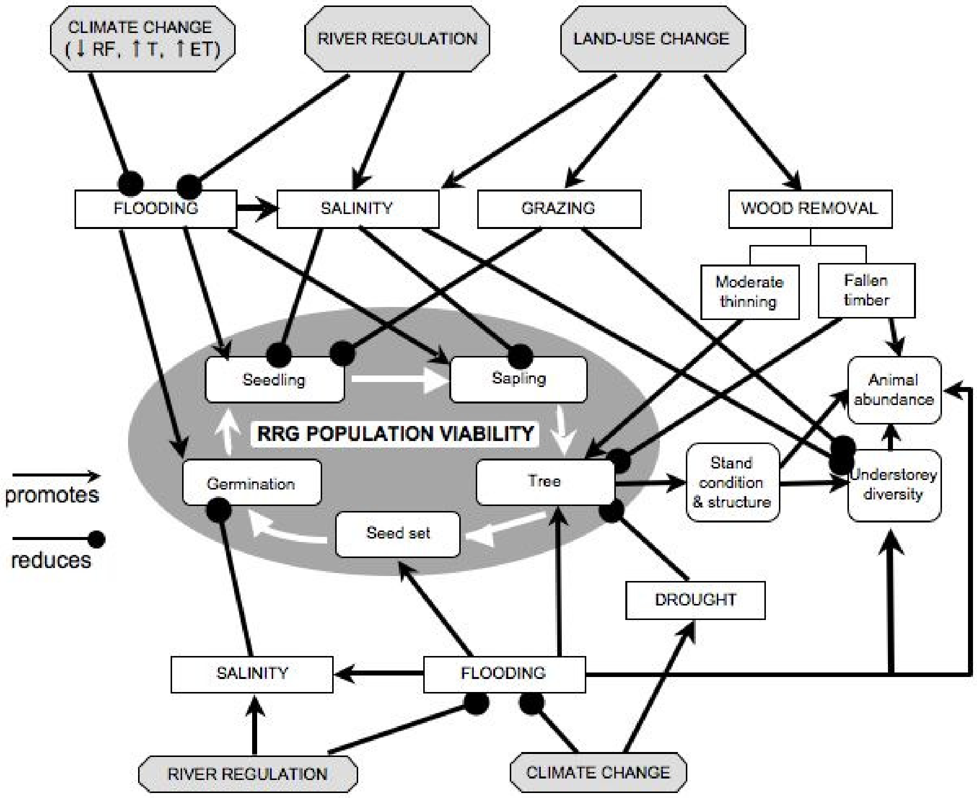

Figure S8. 2**.** Process model linking human actions (octagons) to ecological stressors (white rectangles) to consequences on river red gum (RRG) population viability and stand condition. Arrows indicate relationships that promote the quantity or process to which the arrow is directed; solid circles have reducing or inhibiting effects. RF = rainfall, ET = evapotranspiration and T = temperature, with up (increases) and down (decreases) arrows indicating directions of change [source: (Mac Nally et al. 2011)].

Timber management has left floodplain forests in a much-altered condition. Timber harvesting has resulted in predominantly even-aged stands of straight ‘poles’ with few large trees, especially in the easterly remnant floodplains (Horner et al. 2009, Horner et al. 2010). Forest structure prior to European settlement is thought to have consisted of relatively few large (diameter at breast height ≥ 1 m), widely spreading trees interspersed with a mosaic of mixed-aged stands of differing sizes (Jacobs 1955). River red gum forests have been extensively exploited for firewood for more than a century. In the public forests of the Murray-Darling Basin, ca 1.15×10^5 t yr^{-1} of firewood and ca 1.22×10^5 t yr^{-1} of timber (including wood chips) are removed (Crabb 1997). This intense harvesting pressure has much reduced the loads of fallen timber, possibly by 80% (Mac Nally et al. 2002). Reductions in numbers of birds and other vertebrates followed the decline in habitat quality (hollow-bearing trees, fallen timber) (Mac Nally et al. 2001).

Grazing by cattle and sheep on Murray-Darling Basin floodplains began in the 1830s (VEAC 2008). Feral rabbits became abundant in the 1890s (Bacon et al. 1994). Grazing licenses for livestock continue on some of the floodplains (NRC 2009a). The grazing of floodplain forests by livestock, sheep and cattle has demonstrably adverse effects on understorey vegetation, including the saplings of river red gum (Robertson and Rowling 2000). Floodplain condition (predominantly understorey vegetation indicators) is adversely affected by grazing intensity (Jansen and Robertson 2001). Rabbit numbers have recovered from the impacts of rabbit haemorrhagic disease virus (Lawrence 2009), while some

native herbivores, such as kangaroos, have been largely restricted to remnant native vegetation, including the floodplain forests, by landscape change for agriculture, (Morgan and Pegler 2010).

Ecosystem collapse

For assessment of criteria A and B, collpase was assumed to occur when the mapped distribution or projected suitable habitat declines to zero. Because this is a floodplain ecosystem threatened by water diversion and climate change, we identified river flow as a suitable variable for assessing the relative severity of environmental degradation under criterion C and we identified the extent of mappable tree dieback as a suitable variable for estimating the extent of degradation. Conservatively, we assumed that the ecosystem will collapse when mean flowthrough to mouth falls to 0-10% of unregulated levels with tree dieback occurring throughout 100% of the distribution. We identified several measures of forest-dependent birds as a suitable variables for assessing disruption to biotic processes and interactions under criterion D, as the Red Gum ecosystem is the only forest ecosystem throughout most of its distribution. We assumed the ecosystem would collapse if bird abundance or breeding activity declined to zero.

ASSESSMENT

Summary

Criterion	A	B	C	D	E	Overall
Subcriterion 1	LC	LC	VU(LC-VU)	LC	DD	VU
Subcriterion 2	VU	LC	VU	LC		
Subcriterion 3	LC	LC	LC(LC-VU)	LC		

Criterion A: Reduction in Geographic Distribution

Current decline: There is little evidence of a major reduction in the extent of floodplain forests in the southern Murray-Darling Basin in the past 50 years. The status of the ecosystem is **Least Concern** under criterion A1.

Future decline: Predictions of change for the Floodplain Ecosystem have been conducted for the Victorian floodplains that are based on climate change associated with three IPCC emission scenarios (Newell et al. 2009). These are a low-emission scenario (B1: increase in average temperature of 2 °C by 2100), a medium-emission scenario (A1B: increase in average temperature of 3 °C by 2100) and a high-emission scenario (A1F: increase in average temperature of 4.5 °C by 2100) (www.ipcc.ch). Models were built with *maxent* (www.cs.princeton.edu/~shapire/Maxent) and used a range of environmental predictors in conjunction with known occurrences of the river red gum and black box (Newell et al. 2009). Only the predictor 'flood extent' ultimately was deemed useful for future projections. Distributions were computed for the three emission scenarios for 2030, 2050, and 2080.

The results suggested that suitability will be little reduced for the Floodplain Ecosystem in the low-emission scenario (Figure 3), although this would not be the case by 2080 (not shown, beyond the 50-year horizon). In the medium- and high-emission scenarios, there would be a major loss of area of suitability for the Floodplain Ecosystem by 2050. The modellers were cautious about the predictions because both the river red gum and the black box are widely distributed across inland Australia in conditions that are hotter and more arid than the southern Murray-Darling Basin (Newell et al. 2009). Nevertheless, a decline in area of suitable floodplain of at least 30% is likely, and the status of the ecosystem is **Vulnerable** under criterion A2.

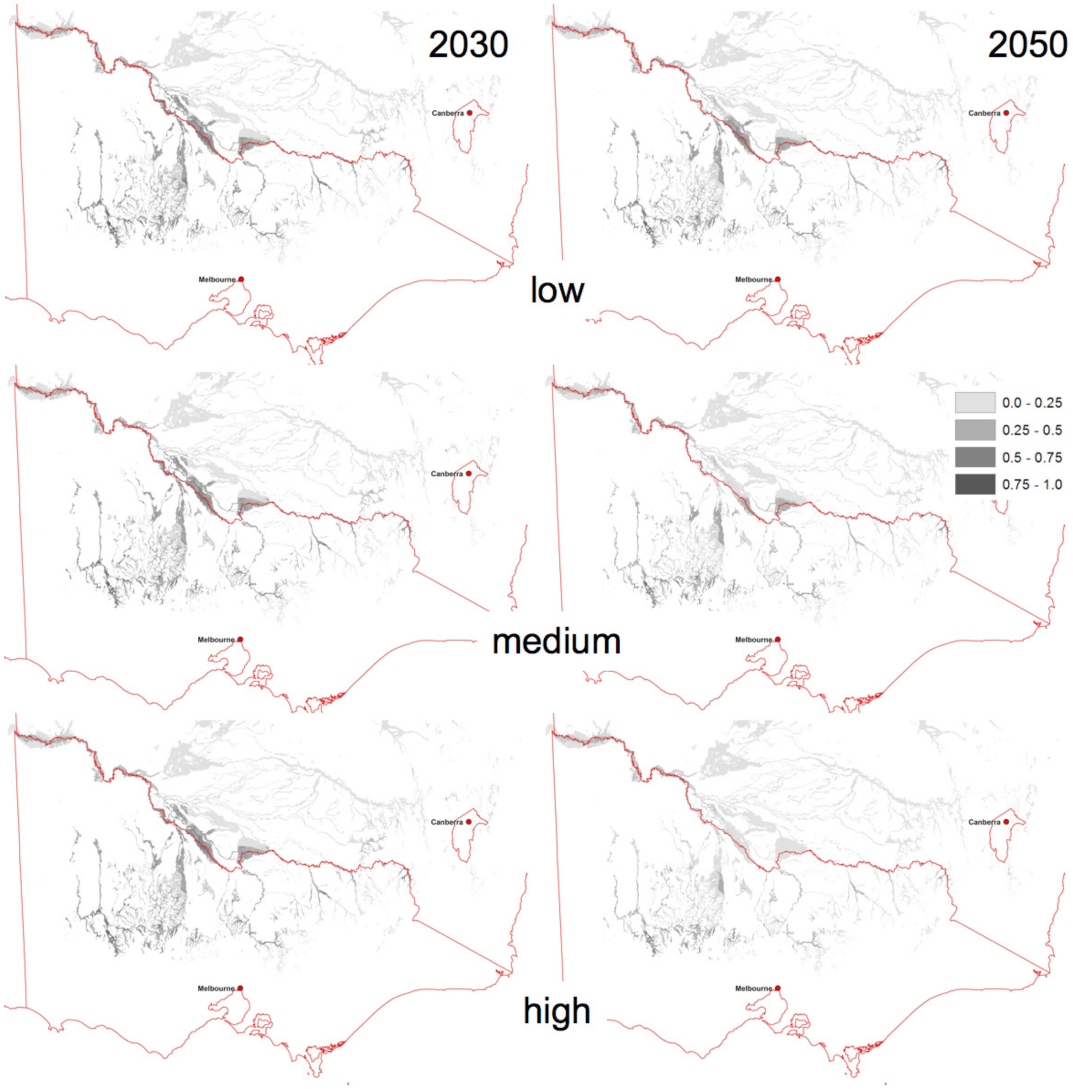

Figure S8. 3. Projected distributions of the Floodplain Ecosystem in Victoria in 2030 and 2050 under three IPCC emission scenarios (Newell et al. 2009).

Historic decline: The floodplain forest systems have been spared the degree of total tree clearance seen in the Murray-Darling Basin as a whole (Walker et al. 1993). For example, 90,000 ha (60% of historical) of the floodplain forest in northern Victoria (VEAC 2008) and 236,000 ha (68% of historical) of the floodplain forest in the Riverina of southern New South Wales remains (NRC 2009b). This relatively high areal retention is due to the management of river red gum forests for timber extraction (poles, posts, house frames, railway sleepers and firewood), which has been a major activity for almost 200 years (Crabb 1997). As the historic decline in distribution is estimated to be less than 50%, the status of the ecosystem is **Least Concern** under criterion A3.

Criterion B

Extent of occurrence: A minimum convex polygon enclosing occurrences of the Floodplain Ecosystem along the Murray River and its distributaries has an area of about 69,350 km^2 (Figure 4). Extension of this polygon to include occurrrences along the the Murrumbidgee and Lachlan Rivers (major northern tributaries) encompasses an area in excess of 221 700 km^2. As both estimates exceed an extent of occurrence of 50,000 km^2, the status of the ecosystem is **Least Concern** under criterion B1.

Area of occupancy: The Floodplain Ecosystem occupies 243 10 × 10 km grid cells along the Murray River and its distributaries alone, excluding cells with less than 1% of cell area occupied (Fig. 4). A further 183 cells are similarly occupied along the Murrumbidgee and Lachlan Rivers. These estimates greatly exceed the thresholds for threatened status, the ecosystem is **Least Concern** under Subcriterion B2.

Number of locations: As changes in river flows are the most serious plausible threat, and the Floodplain Ecosystem is fed by multiple river systems, there are more than five locations each exceeding 1000 ha each across the southern Murray-Darling Basin. The status of the ecosystem is **Least Concern** under Subcriterion B3.

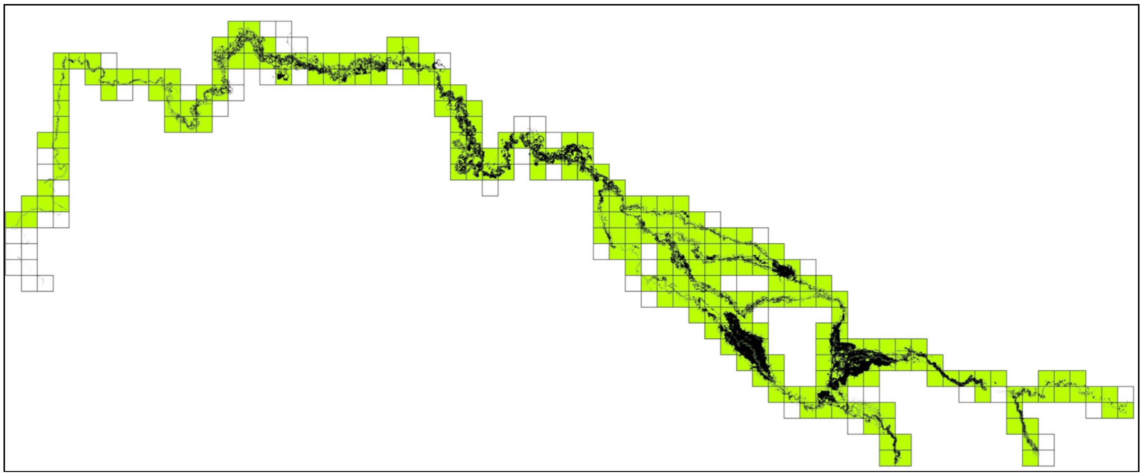

Figure S8. 4**.** Mapped distribution of Floodplain Ecosystem along the Murray River and its major tributaries in the southern MDB showing occupied 10 × 10 km gird cells (Area of occupancy). Green indicates cells with more than 1% of cell area occupied.

Criterion C

Climate change and river regulation are two major anthropogenic drivers of degradation of the physical environment for the Floodplain Ecosystem (Figure 2). The primary stressors that emanate from these changes are: (1) decreases in flows and flooding frequency, extent and duration; and (2) increases in salinity of the river, ground water and soil.

Current degradation: The region in which the Floodplain Ecosystem occurs has been heating without remission since the late 1990s. The mean temperature anomaly (south-eastern Australia) relative to the 1961-1990 baseline of the Bureau of Meteorology is about 0.6 °C since 1997 (http://www.bom.gov.au/cgi-bin/climate/change/, accessed December 14, 2011). After an historically unprecedented run of very dry years between 1997 and 2010 (Kiem and Verdon-Kidd 2010), rainfall was much above the baseline from late-2010 and into 2011. However, autumnal and winter rainfall remained low, and rainfall in these seasons has a major effect on catchment wetting.

Interception of flows by impoundments – major dams, weirs and off-river storages including hundreds of thousands of farm dams – has been a major contributor to ecological decline. River regulation in the Murray-Darling Basin has reduced peak flows, and the frequency (35–62% of historical) and duration (40–80% of historical) of extensive floods that connect the Murray River to its anabranches (Maheshwari et al. 1995). That variation declined markedly from the late-1990s, mainly due to much-reduced inflows (Cai and Cowan 2008b). Diversions (for irrigation, stock and domestic) grew steadily from the 1930s, especially following the commissioning of the Hume Dam in the upper reaches of the

Murray River in the early 1930s (Kingsford 2000). By the early 1990s, the state and federal governments imposed a cap on further increases in extractions. However, temperature rises and reduced

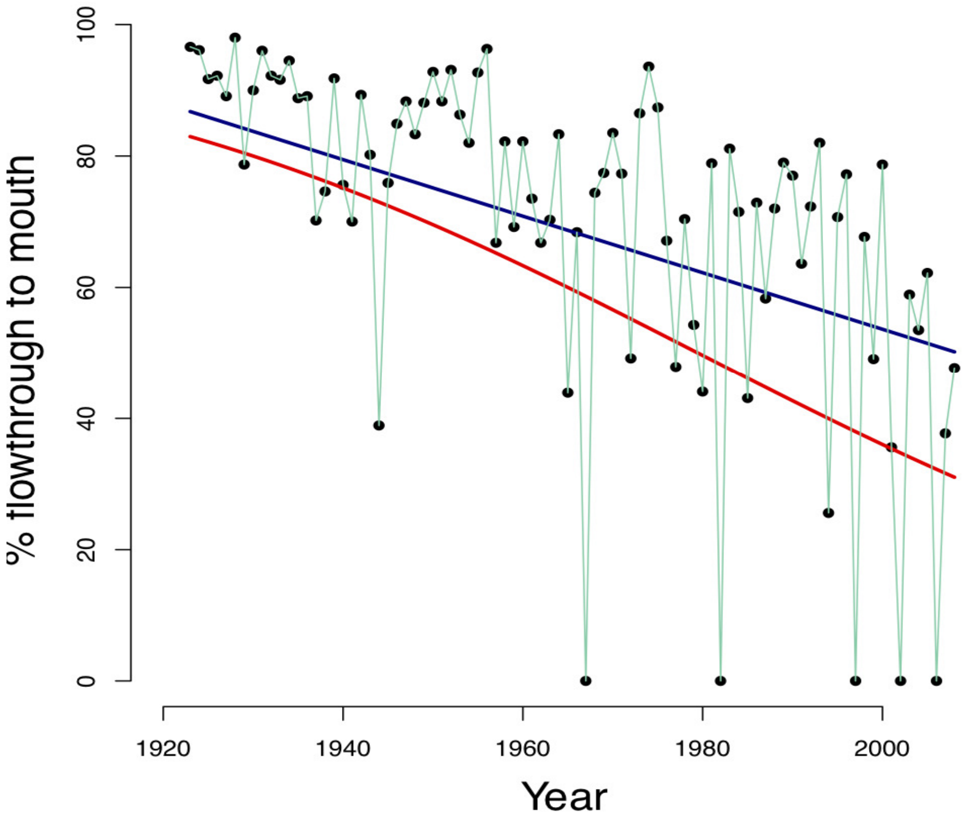

rainfall since the late 1990s, especially in autumn (Cai and Cowan 2008a), led to a marked decline in water availability (Cai and Cowan 2008b).

Figure S8. 5. The percentage of water flowing through to the Murray mouth (points and aqua line) is the difference between the modeled flows given no development and the actual water extractions. A linear fit with time (navy line) and a beta-regression fit (red line) are shown.

Modelling from the Murray-Darling Basin Authority for its Basin Plan provided estimates of outflows to the Murray mouth in the absence of development (Murray Darling Basin Authority 2012). Data for actual extractions also are available from which the percentage of flows reaching the mouth can be calculated (aqua line Figure 5). We fitted both a conventional Gaussian linear fit to the data (navy line, Figure 5) and a beta regression (red line, Figure 5); the latter is more appropriate for values bounded between 0 and 1 (percentages converted to proportions). The fitted percentages in 1960 were 71% (Gaussian) and 63% (beta), while the latest fitted values were 50% (Gaussian) and 31% (beta). Note that the zero values are truncated so that in those years, there was a net loss of flow to the mouth of the Murray River; water was "mined" from storages in those years. Including those values (-800% in 2006) would favour the beta-regression estimates more strongly than the Gaussian estimates. Assuming that the ecosystem will not collapse until mean flowthrough falls to zero, these values suggest a decline in water flowthrough to mouth (severity of degradation) of 100 × (71-50)/(71-0) = 30% (Gaussian model) to 100 × (63-31)/(63-0) = 51% (beta model) over the past 50 years. If the ecosystem collapses when mean flowthrough falls below 10% of unregulated levels, the severity of degradation is estimated to be 100 × (71-50)/(71-10) = 34% (Gaussian model) to 100 × (63-31)/(63-10) = 60% (beta model) over the

past 50 years. Hence the estimates of relative severity of environmental degradation vary from 30-60% based on decline in flowthrough.

The extent of the floodplain ecosystems affected by reduced flows has risen from 44.7% in 1990 (the earliest quantitative assessment) to 79% in 2010 (Table 1, Figure 6). Our extensive examinations of the literature did not find any references to extensive die-back until the 1980s, which suggests that one can assume an *extent* of 0% affected in 1960.

The 79% extent and 51(30 - 60)% relative severity of environmental degradation based on hydrological changes since 1960 suggests a status of **Vulnerable** (plausible range Least Concern - Vulnerable) under Subcriterion C1, and marginally below Endangered (Fig. 7).

Table S8. 1**.** Proportions of forested areas (%) in good condition and in die-back on the Murray River floodplain (Cunningham et al. 2011a, Mac Nally et al. 2011).

Condition class	Proportion of forest area (%)				
	1990	2003	2006	2009	2010 (1st half)
Good	55.3	47.1	29.6	30.5	21.0
Die-back	44.7	52.9	70.4	69.5	79.0

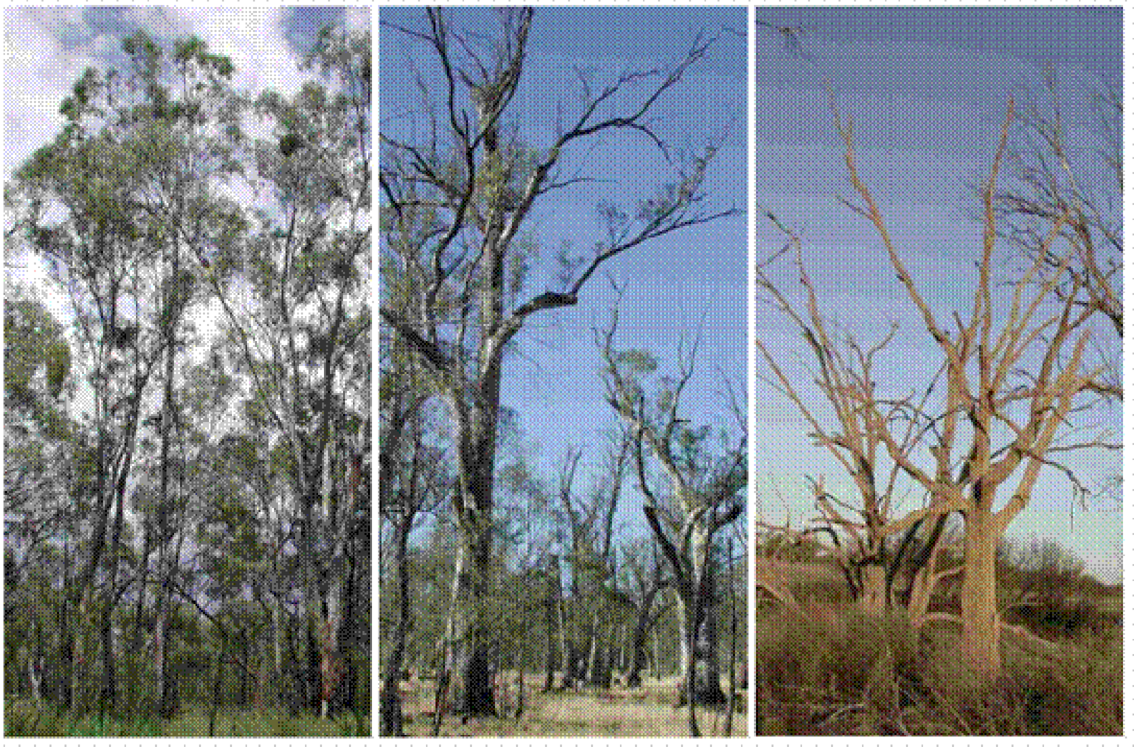

Figure S8. 6. Example stands showing a gradient from good condition river red gum stands (left) to poor (centre) to severely degraded (right). Photographic credit: Shaun C Cunningham.

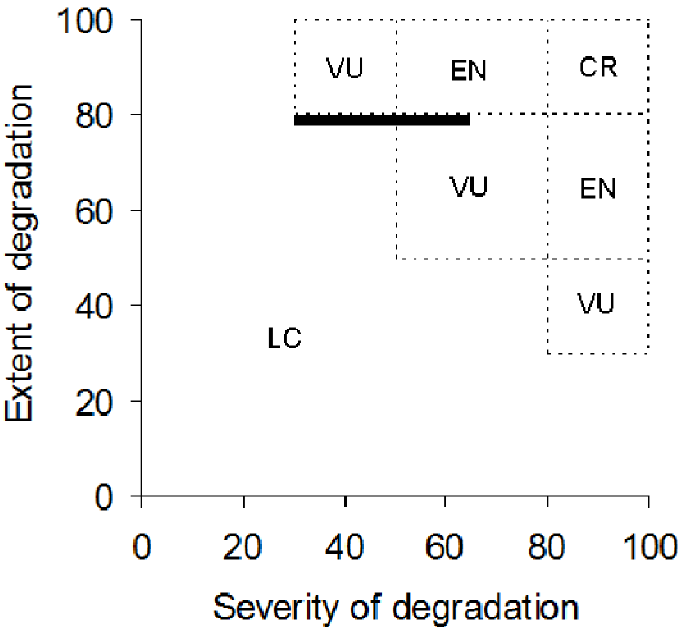

Figure S8. 7**.** Status of the floodplain ecosystem under criterion C1 (black bar). A small increase in extent of hydrological degradation to 80% will change the status from LC-VU to VU-EN.

Future degradation: Warming and drying are expected to increase to an even greater extent than occurred in the 'Big Dry' from 1997-2010 (Garnaut 2011). Australia's Federal Government has implemented a water-rights buy-back scheme with the intention to deliver environmental water for river and wetland health, including floodplains. Depending on the relative increase in the severity of the climate effects and the efficacy of the management water strategy, the status of the ecosystem is **Vulnerable** under Subcriterion C2.

Historic degradation: The severity of change since 1750 is 50-79% (depending on the fitted model) and the extent of area affected is 79%. This leads to a status of **Least Concern** (plausible range Least Concern - Vulnerable) under Subcriterion C3.

Criterion D

The effects of reduced stand condition on some of the characteristic fauna, birds and small mammals, provide some indications of disrupted biotic interactions, although more data are required for a broader range of taxonomic groups (e.g. understorey plants and invertebrates). The total number of bird species recorded, the total records of all species, the number of species showing any breeding activities and total breeding activity (summed over all species) (Mac Nally 2007) all were positively related to stand condition (Mac Nally et al. 2011). Retrospective (to 1990) estimates of distributions of stand condition have been made using remotely sensed information, while the most recent measures are for 2010. We assumed that pre-European settlement (1750) stand condition was uniformly "good." Results of regression models for bird use and breeding in 2009 as a function of stand condition were combined with estimated proportions of stand condition to produce estimates of total change in four measures of bird use and breeding over the floodplain domain (Figure 8).

Current decline: Data exist from 1990–2010 and the four avian measures have declined by about 10 - 25% over that 20-year period. The decline in avian communities may have continued over a longer period than that for which data are available. If similar rates of decline had commenced in 1980, the declines would be 15-35%. If these declines occurred through 79% of the extent of the ecosystem (Table 1), the status of the ecosystem is **Least Concern** under Criterion D1.

Future decline: There was a strong rainfall and flooding event in southeastern Australia between the middle of 2010 and much of 2011. Stand condition needs to be re-evaluated to assess the degree to which this possibly rare spike in rainfall reverses the declines observed until early 2010 (Table 1). At this stage, an assessment would remain be similar to D1 and hence **Least Concern** under Subcriterion D2.

Historical decline: Assuming that the floodplain ecosystem in 1750 was uniformly “good”, the four measures for the birds would have declined by between 25-40%. Assuming these declines occurred through 79% of the extent of the distribution (Table 1), the status of the ecosystem is **Least Concern** under Criterion D3.

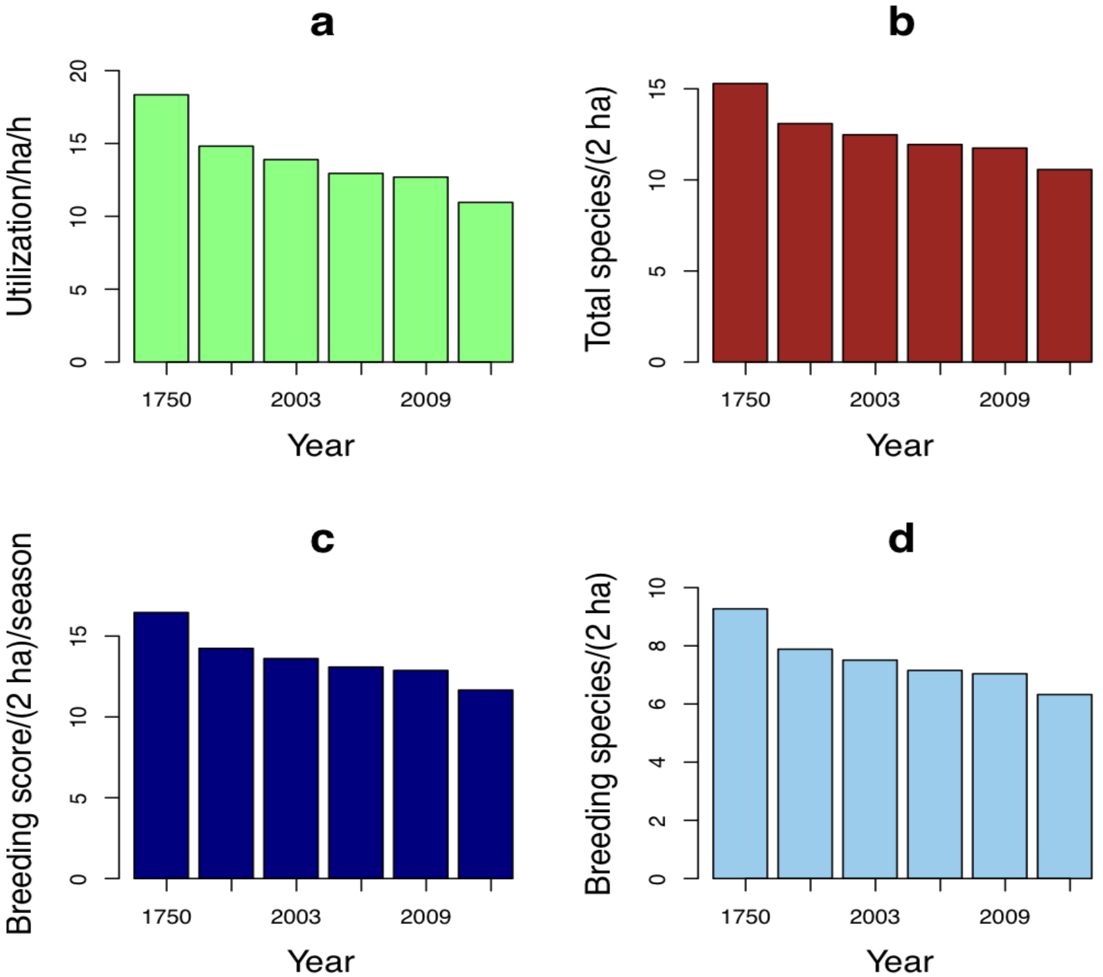

Figure S8. 8**.** Four measures of the effects of deterioration in stand condition on floodplain woodland birds: (a) utilization (effectively numbers of birds seen per ha per h); (b) total species seen per 2 ha; (c) a measure of breeding activity (Mac Nally 2007); and (d) numbers of breeding species. Years are: 1750, 1990, 2003, 2006, 2009 and 2010.

Criterion E

No quantitative analysis has been carried out to assess the risk of ecosystem collapse for the Floodplain Ecosystem. Therefore, the status of the ecosystem is **Data Deficient** under Criterion E.

REFERENCES

Bacon, P. E., K. Ward, P. Craven, M. Harper, and M. Bone. 1994. Floodplain and land-use issues in the Murray-Darling basin. Pages 76-87 in T. Sharley and C. Higgins, editors. Murray-Darling basin floodplain wetlands management. Murray-Darling Basic Commission, Canberra.

Bennetts, D. A., J. A. Webb, D. J. M. Stone, and D. M. Hill. 2006. Understanding the salinisation process for groundwater in an area of south-eastern Australia, using hydrochemical and isotopic evidence. Journal of Hydrology 323:178-192.

Bren, L. J. 1990. Red gum forests. Pages 231-242 in N. Mackay and D. Eastburn, editors. The Murray. Murray Darling Basin Commission, Canberra.

Bren, L. J., I. C. O'Niell, and N. L. Gibbs. 1987. Flooding in the Barmah Forest and its relationship to flow in the Murray-Edward River system. Australian Forest Research 17:127-144.

Cai, W., and T. Cowan. 2008a. Dynamics of late autumn rainfall reduction over southeastern Australia. Geophysical Research Letters 35:L09708.

Cai, W., and T. Cowan. 2008b. Evidence of impacts from rising temperature on inflows to the Murray-Darling Basin. Geophysical Research Letters 35:L07701.

Crabb, P. 1997. Murray-Darling Basin Resources. Murray-Darling Basin Commission, Canberra.

Cunningham, S., P. Griffioen, M. White, and R. Mac Nally. 2011a. Mapping the condition of river red gum (*Eucalyptus camaldulensis* Dehnh.) and black box (*Eucalyptus largiflorens* F.Muell.) stands in the Living Murray icon sites. Australian Centre for Biodiversity, Monash University, Melbourne.

Cunningham, S., R. Mac Nally, J. Read, P. Baker, M. White, J. R. Thomson, and P. Griffioen. 2009. A robust technique for mapping vegetation across a major river system. Ecosystems 12:207-219.

Cunningham, S., J. Thomson, R. Mac Nally, J. Read, and P. Baker. 2011b. Groundwater change forecasts widespread forest dieback across an extensive floodplain system. Freshwater Biology 56:1494-1508.

Dexter, B. D., J. J. Rose, and N. Davies. 1986. River regulation and associated forest management problems in the River Murray Red Gum forests. Australian Forestry 49:16-27.

Garnaut, R. 2011. The Garnaut Review 2011: Australia in the Global Response to Climate Change. Cambridge University Press Cambridge.

Gee, G. W., and D. Hillel. 1988. Groundwater recharge in arid regions - review and critique of estimation methods. Hydrological Processes 2:255-266.

Horner, G., P. J. Baker, R. Mac Nally, S. Cunningham, J. Thomson, and F. Hamilton. 2009. Regeneration and mortality of floodplain forests subjected to a drying climate and water extraction. Global Change Biology 15:2176-2186.

Horner, G., P. J. Baker, R. Mac Nally, S. C. Cunningham, J. R. Thomson, and F. Hamilton. 2010. Carbon and habitat benefits from thinning floodplain forests: managing early stand structure makes a difference. Forest Ecology and Management 259:286-293.

Jacobs, M. R. 1955. Growth habits of the eucalypts. Australian Government, Canberra.

Jansen, A., and A. I. Robertson. 2001. Relationships between livestock management and the ecological condition of riparian habitats along an Australian floodplain river. Journal of Applied Ecology 38:63-75.

Jolly, I. D. 1996. The effects of river management on the hydrology and hydroecology of arid and semi-arid floodplains. Pages 577-609 in M. Anderson, D. Walling, and P. Bates, editors. Floodplain Processes. John Wiley & sons, Chichester.

Keith, D. A. 2004. Ocean shores to desert dunes: the native vegetation of New South Wales and the ACT. NSW Department of Environment and Conservation, Sydney.

Kiem, A. S., and D. C. Verdon-Kidd. 2010. Towards understanding hydroclimatic change in Victoria, Australia - preliminary insights into the 'Big Dry'. Hydrology and Earth System Sciences 14:433-445.

Kingsford, R. T. 2000. Ecological impacts of dams, water diversions and river management on floodplain wetlands in Australia. Austral Ecology 25:109-127.

Lada, H., R. Mac Nally, and A. C. Taylor. 2008. Responses of a carnivorous marsupial (Antechinus flavipes) to local habitat factors in two forest types. Journal of Mammalogy 89:398-407.

Lada, H., J. R. Thomson, R. Mac Nally, G. Horrocks, and A. C. Taylor. 2007. Evaluating simultaneous effects of three anthropogenic effects on a floodplain-dwelling marsupial Antechinus flavipes. Biological Conservation 134:527-536.

Lawrence, L. 2009. Australian rabbits are doing what comes naturally - again. Outlooks on Pest Management 21:19-21.

Mac Nally, R. 2007. Consensus weightings of evidence for inferring breeding success in broad-scale bird studies. Austral Ecology 32:479-485.

Mac Nally, R., P. J. Baker, S. C. Cunningham, G. J. Horner, and J. R. Thomson. 2011. Dynamics of Murray-Darling floodplain forests under multiple water stressors - The past, present and future of an Australian icon. Water Resources Research 47:W00G05.

Mac Nally, R., A. Parkinson, G. Horrocks, L. Conole, and C. Tzaros. 2001. Relationships between terrestrial vertebrate diversity, abundance and availability of coarse woody debris on south-eastern Australian floodplains. Biological Conservation 99:191-205.

Mac Nally, R., A. Parkinson, G. Horrocks, and M. Young. 2002. Current loads of coarse woody debris on south-eastern Australian floodplains: evaluation of change and implications for restoration. Restoration Ecology 10:627-635.

Maheshwari, B. L., K. F. Walker, and T. A. McMahon. 1995. Effects of regulation on the flow regime of the River Murray, Australia. Regulated Rivers: Research & Management 10:15-38.

Margules, P., J. Smith, and P. Smith. 1990. Riparian vegetation of the River Murray. Murray Darling Basin Commission, Canberra.

MDBC. 2005. Survey of River Red Gum and Black Box Health Along the River Murray in New South Wales, Victoria and South Australia - 2004. Murray-Darling Basin Commission, Canberra.

Morgan, D. G., and P. Pegler. 2010. Managing a kangaroo population by culling to simulate predation: the Wyperfeld trial. Pages 349-360 in D. Coulson and M. Eldridge, editors. The biology of kangaroos, wallabies and rat-kangaroos. CSIRO, Melbourne.

Murray Darling Basin Authority. 2012. Basin Plan Knowledge and Information Directory. Murray Darling Basin Authority, Canberra.

Newell, G., M. White, and P. Griffioen. 2009. Potential impacts of a changing climate on selected ecosystems in northern Victoria. Arthur Rylah Institute for Environmental Research, Heidelberg, Victoria.

NRC. 2009a. Final Assessment Report. Riverina Bioregion. Regional Forest Assessment. River red gum and woodland forests. Natural Resources Commission, Sydney.

NRC. 2009b. Recommendations Report. Riverina Bioregion. Regional Forest Assessment. River red gum and woodland forests. Natural Resources Commission, Sydney.

Overton, I. C., I. D. Jolly, P. G. Slavich, M. M. Lewis, and G. R. Walker. 2006. Modelling vegetation health from the interaction of saline groundwater and flooding on the Chowilla floodplain, South Australia. Australian Journal of Botany 54:207-220.

Resource_and_Environmental_Management. 2003. Hydrogeological Benchmark Assessment for the River Murray Between Wentworth and Renmark. Final Report - Work Initiatives. Resource and Management Pty. Ltd., Kent Town, South Australia.

Roberts, J. 2004. Floodplain forests in the southern Murray-Darling Basin. Australian Conservation Foundation, Canberra.

Robertson, A. I., and R. W. Rowling. 2000. Effects of livestock on riparian zone vegetation in an Australian dryland river. Regulated Rivers-Research & Management 16:527-541.

SKM, and J. Roberts. 2003. Assessment of water management options for Lindsay and Wallpolla Islands. Sinclair Knight and Merz, Melbourne.

Specht, R. L. 1981. Major vegetation formations in Australia. Pages 163-297 in A. Keast, editor. Ecological biogeography of Australia. Dr. W. Junk, The Hague.

Thomson, C. 1992. The impact of river regulation on the natural flows of th Murray-Darling Basin. Technical Report 92/5.1. Murray-Darlin Basin Commission, Canberra.

Tzaros, C. L. 2001. Importance of riparian vegetation to terrestrial avifauna along the Murray River, south-eastern Australia. MSc. Deakin, Melbourne.

VEAC. 2008. River red gum forests investigation: Final Report. Victorian Environmental Assessment Council, Melbourne.

Walker, J., F. Bullen, and B. G. Williams. 1993. Ecohydrological changes in the Murray-Darling Basin. 1. The number of trees cleared over two centuries. Journal of Applied Ecology 30:265-273.

9 COOLIBAH - BLACK BOX WOODLANDS, SOUTH-EASTERN AUSTRALIA

contributed by David Keith, Australian Wetlands and Rivers Centre, University of New South Wales and NSW Office of Environment & Heritage

CLASSIFICATION

International: Currently not classified.
National: Nationally and within New South Wales, Coolibah - Black Box Woodlands are classified as an Endangered Ecological Community (TSSC 2011; NSW Scientific Committee 2004; 2008). In Queensland, the ecosystem includes: RE 11.3.3 *Eucalyptus coolabah* woodland on alluvial plains; RE 11.3.15 *Eucalyptus coolabah, Acacia stenophylla, Muehlenbeckia florulenta* fringing woodland on alluvial plains; RE 11.3.16 *Eucalyptus largiflorens,* ±*Acacia cambagei* ±*A. harpophylla* woodland to low open woodland on alluvial plains; RE 11.3.28 *Eucalyptus coolabah* ± *Casuarina cristata* open woodland on alluvial plains; and RE 11.3.37 *Eucalyptus coolabah* fringing woodland on alluvial plains. In New South Wales, the ecosystem belongs to the North-west Floodplain Woodlands vegetation class (Keith 2004) and includes: ID 37 – Black Box woodland on floodplains of the NSW central and northern wheatbelt including the Darling Riverine Plains Bioregion; ID 39 – Coolibah – River Coobah – Lignum woodland of frequently flooded channels mainly of the Darling Riverine Plains Bioregion; and ID 40 – Coolibah open woodland with chenopod/grassy ground cover on grey and brown clay floodplains (Benson et al. 2006). These units all fall within the Northwest Floodplain Woodlands vegetation class (Keith 2004).
IUCN Habitats Classification Scheme (Version 3.0): 2. Savanna / 2.1 Dry savanna.
Key references: TSSC 2011; NSW Scientific Committee 2004; 2008; Benson et al. 2006.

ECOSYSTEM DESCRIPTION

Characteristic native biota

In its mature state, Coolibah – Black Box Woodland has an open structure with widely scattered trees, a variable cover of shrubs and grassy groundlayer. In its regeneration phase, it may include dense stands of saplings with limited understorey and ground layer development. *Eucalyptus coolabah* is most frequently occurring tree species, with other species including *Eucalyptus largiflorens, Eucalyptus camaldulensis*, *Eucalyptus populnea* subsp. *bimbil, Acacia stenophylla, A. salicina*, *Casuarina cristata* and *Eremophila bignoniiflora*. Common shrubs species include *Muehlenbeckia florulenta* and *Rhagodia spinescens,* while the ground layer comprises a diverse suite of grasses including species of *Astrebla, Chloris, Dichanthium, Enteropogon, Panicum, Paspalidium* and *Sporobolus*.

The characteristic vertebrate fauna includes diverse assemblages of woodland and wetland bird species, many of which depend on tree hollows, other features of large trees or standing water for breeding and or foraging (see NSW Scientific Committee, 2004; 2008 for a full description). Regionally, the ecosystem is distinguished compositionally from other woodlands, which lack *E. coolibah* and the diverse grassy ground layer, and structurally from grasslands and shrublands, which lack trees and many of the characteristic woody species. Further west, floodplains are more limited in extent and experience less inundation, with ephemeral plants replacing many of the perennial plant species.

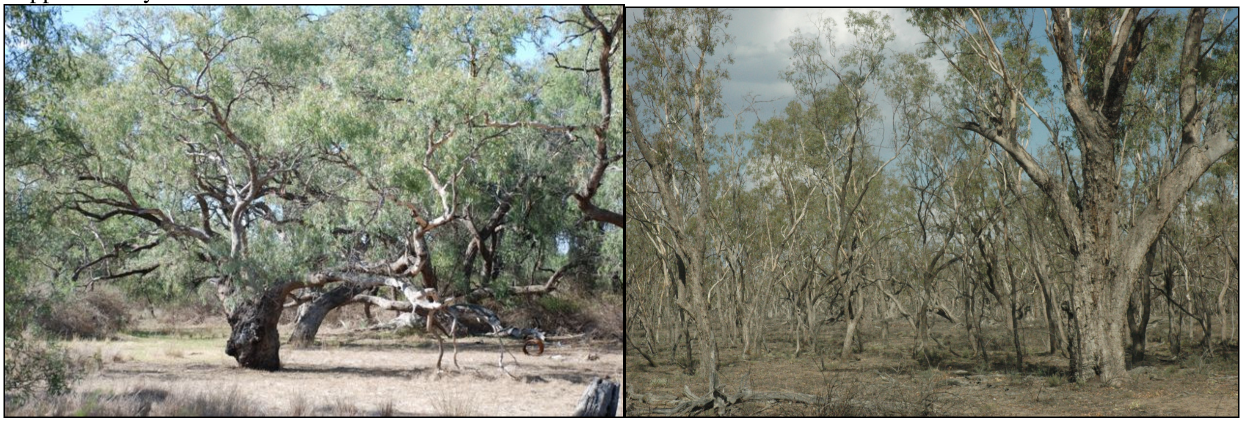

Figure S9. 1. Examples of Coolobah - Black Box Woodland in mature state (left, near Moree, NSW, Australia) and regeneration phase (right, Culgoa National Park, NSW, Australia). Both sites show effects of grazing by feral herbivores.

Abiotic environment

Coolibah - Black Box Woodland occurs on level floodplains dissected by meandering river channels. The plain has deep alluvial clay and silt soils, which are periodically inundated by overbank flows and may remain waterlogged for extended periods. The climate is subtropical and semi-arid, with summer-dominant rainfall, declining from an average of about 600 mm per annum in the east to less than 350 mm per annum in the western part of the distribution.

Distribution

Coolabah - Black Box Woodland occurs on the floodplain of the upper Darling River and its tributaries, within latitudes 28 - 31° S and 146 - 150° E, about 400 km southwest of Brisbane in southeastern Australia. (Figure 2).

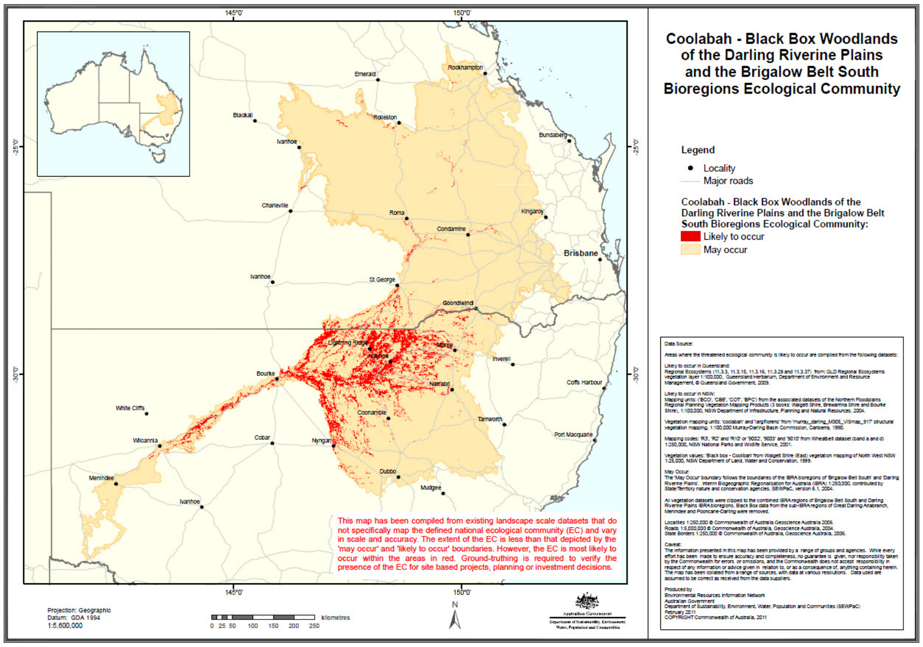

Figure S9. 2. Distribution of Coolibah - Black Box Woodland. Source: Australian Government Department of Sustainability, Environment, Water, Population and Community (http://www.environment.gov.au/biodiversity/threatened/communities/maps/pubs/66-map.pdf) compiled from data supplied by the NSW Office of Environment and Heritage and Queensland Department of Environment and Resource Management.

Key processes and interactions

Water regimes are a key driver of ecosystem dynamics in Coolibah - Black Box Woodland. Major floods may trigger periodic tree recruitment from which dense stands of saplings may develop and self-thin over time, eventually resulting in a sparser cover of large trees (Roberts 1993; Good et al. 2011). Extended dry periods are associated with episodes of tree mortality, which accelerate the thinning process. Different plant species apparently have different recruitment responses to floods of varying magnitude and duration and also different tolerances to droughts of varying severity and duration (Roberts and Marston 2000; Capon 2003; Capon et al. 2009). The composition and structure of overstorey and understorey therefore varies spatially and temporally, depending on soil moisture and local flood regimes (Reid et al. 2011). For example, *Eucalyptus camaldulensis* and *E. populnea* subsp. *bimbil* occur, respectively, on lower and upper parts of the floodplain characterised by contrasting water regimes. *Muehlenbeckia florulenta, Cyperus, Marsilea* and *Sporobolis mitchellii* tend to dominate the woodland understorey at sites where soil moisture is sustained, while grasses and chenopod shrubs dominate in less frequently moist locations (Reid et al. 2011). The water regime also profoundly influences the dynamics of fauna assemblages, with breeding cycles of waterbirds, amphibians and many invertebrates cued to major floods associated with high resource levels (Lee and Mercer 1967; Boulton and Lloyd 1992; Kingsford and Auld 2005). Floods also mediate the movement of nutrients, organic matter, water and biota by periodically connecting rivers, wetlands and floodplains, which are otherwise isolated under dry conditions (Humphries *et al.* 1999; Thoms 2003).

The composition of ground layer vegetation depends on past and present grazing pressure as well as the water regime (Capon 2003, 2005; Reid *et al.* 2011). Feral herbivores and domestic livestock are the most abundant herbivores in the system, and their effects probably overshadow those of native macropods whose abundance depends on inter-annual rainfall variation.

Threatening processes

Four main processes threaten the persistence of this ecosystem (NSW Scientific Committee 2004; 2008). First, expansion and intensification of agricultural land use has replaced large areas of woodland with crops and pastures in recent decades (Keith et al. 2009). Second, extraction of water from rivers for irrigation has altered flood regimes and their spatial extent (Thoms & Sheldon 2000; Thoms 2003), reducing opportunities for reproduction and dispersal of characteristic flora and fauna (Kingsford and Thomas 1995; Kingsford and Johnson 1998; Sims 2004; Kingsford and Auld 2005). Future climate change may also affect the spatial and temporal availability of water in the system (Hennessy et al. 2004). Third, invasive plants have spread with agricultural intensification and are reducing the diversity and abundance of native biota. Invasion of the mat-forming forb, *Phyla canescens,* reduces the diversity of native ground layer plants by (Taylor and Ganf 2005; Price et al. 2010; 2011a,b,c). This species has spread rapidly, in response to altered water regimes and persistent heavy livestock grazing (McCosker 1999; Earl 2003). Finally, overgrazing by feral goats and rabbits and domestic livestock has altered the composition and structure of the woodland vegetation, through slective consumption of palatable native ground layer plants and seedlings of trees and shrubs, with effects most marked beneath trees where livestock concentrate their grazoing activities (Robertson & Rowling 2000; Reid et al. 2011).

Ecosystem collapse

For assessment of criteria A and B, this ecosystem assumed to have collapsed when its mapped distribution has declined to zero as a consequence of clearing for agriculture. Because water regimes are a key driver of ecosystem dynamics and water diversion for irrigation is a major threat in Coolibah - Black Box Woodland, median daily river flow was identified as a suitable variable for assessing environmental degradation under criterion C. Conservatively, it was assumed that the ecosystem would collapse if median flow declined to 0 - 10% of unregulated levels.

ASSESSMENT

Summary

Criterion	A	B	C	D	E	overall
subcriterion 1	VU(VU-EN)	LC	EN	DD	DD	EN
subcriterion 2	DD	LC	DD	DD		
subcriterion 3	VU(VU-EN)	LC	VU	DD		

Criterion A

Current decline: Annual rates of decline in the distribution of Coolibah - Black Box Woodland were estimated from a time series of maps compiled from base maps described by Cox et al. (2000), Metcalfe et al. (2003) and NFRPC (2004a,b,c) and interpretation of subsequent Landsat imagery (Keith et al. 2009). These maps cover almost 90% of the distribution in New South Wales (TSSC 2011). It was assumed that rates of clearing were similar in the remaining 12% of the distribution occurring in Queensland, which seems reasonable given similar economic development and conservation regulations. Keith et al. (2009) estimated that the area of the ecosystem declined on average by 0.79% per year between c. 1984 and 2004. Rates of decline varied within this period, for example, averaging 1.72±0.30% between 1998 and 2004 (Keith et al. 2009). Rates of clearing have not been assessed for the periods before c. 1984 and after 2004. There is evidence that clearing activity commenced after 1900, and accelerated between 1940 and 1969 due to increasing deployment of heavy farm machinery and development of river regulation infrastructure, which made more water available for irrigation (Bedward et al. 2007). Cropping data suggest that rates of clearing continued at similar rates after 2004, at least up until 2007 (Keith et al. 2009), although may have slowed subsequently due to prolonged drought and compliance actions under clearing legislation. For assessment, it was assumed that the rate of decline in distribution during 50 years 1960-2010 was at least 0.8% and at most 1.7% per year, with an intermediate scenario of 0.8% per year for the past 25 years and 1.7% per year for the preceding 25 years. Under these scenarios, the distribution of the community was estimated to have declined by $100\times(1-0.008)^{50} = 33\%$, $100\times(1-0.008)^{25} \times (1-0.017)^{25} = 47\%$, $100\times(1-0.008)^{50} = 58\%$, respectively. A bounded estimate of decline in distribution for the past 50 years is therefore 47% (plausible bounds 33-58%). The status of the ecosystem is therefore Endangered (plausible range Vulnerable-Endangered) under A1.

Future decline: Projections of future declines in distribution can be made by assuming similar rates of land conversion continue into the future (Keith et al. 2009). There is little impediment to continued clearing imposed by protected area land tenure (the ecosystem occurs mostly on freehold or leasehold land used for agriculture) and declaration of dominant trees under 'invasive native scrub' regulations promotes clearing of the ecosystem when in the juvenile thicket phase (Fensham 2008). However, clearing of native vegetation and availability of water for irrigation are regulated by permit under legislation. These opposing influences, together with uncertainties in future trends of water extraction and impacts of climate change on the water regime, create complex future scenarios that are yet to be modelled. The status of the ecosystem under criterion A2 is therefore Data Deficient.

Historic decline: Keith et al. (2009), using maps constructed from simple habitat suitability models and field reconnaissance, estimated that the historic distribution of Coolibah - Black Box Woodland in NSW had declined by 61% (plausible bounds 50-67%). Similar mapping in Queensland produced an estimated historic decline of 82% (Queensland Herbarium 2009). The combined data suggest an overall historic decline of 65% (TSSC 2011), with a plausible lower bound above 50% and upper bound likely to be marginally above 70%. The status of the ecosystem is therefore Vulnerable under criterion A3.

Criterion B

Approximately 1.3 million ha of Coolibah - Black Box Woodland have been mapped from recent aerial photography and Landsat imagery (Fig. 2, TSSC 2011). The swamps occur naturally in small patches, with approximately 42% of mapped swamps covering less than 1 ha, and making up about 6% of the total mapped area.

Extent of occurrence: A minimum convex polygon enclosing all mapped occurrences of Coolibah - Black Box Wooland has an area of 266,400 (130,200 - 437,300) km^2 (best estimate based on Fig. 3, lower bound excludes putative occurrences north of 28°S and west of 145°30'E, upper bound based on full extent in Fig. 2). Although there is evidence of continuing decline in distribution and continuing environmental degradation, the status of the ecosystem under criterion B1 is Least Concern because the estimated extent of occurrence exceeds the thresholds for all threatened categories.

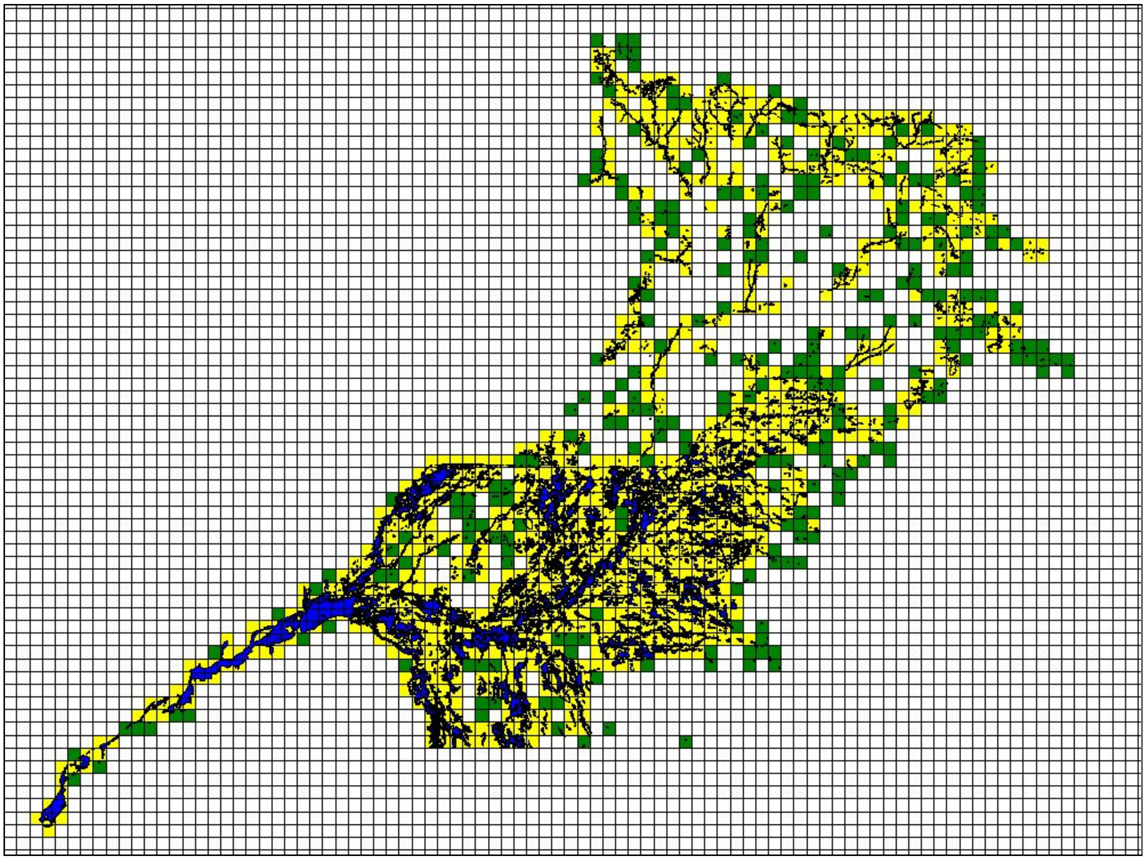

Figure S9. 3. Mapped distribution of Coolibah - Black Box Woodland (blue) showing occupied 10 × 10 km grid cells (Area of occupancy) in New South Wales (data from Keith et al. 2009, Queensland Environment Protection Authority 2009). Yellow- cells with more than 1% of cell area occupied. Green- additional occupied cells with less than 1% of cell area occupied.

Area of occurrence: Superimposing a 10 km grid over the mapped polygons of Coolibah - Black Box Woodland (Figure 3) indicates that they are present within 1193 grid cells (752 within New South Wales). Of these, 283 grid cells (92 in NSW) contain less than 1 km^2 of the ecosystem (i.e. <1% of the area of a grid cell). Excluding these small occurrences, the swamps are therefore estimated to occupy 910 10 × 10 km grid cells (shaded yellow in Fig. 3), exceeding the threshold for Vulnerable by a substantial margin. Assessments of B2 subcriteria are identical to those for criterion B1 (see Extent of occurrence, above). The status of the ecosystem is therefore Least Concern under criteria B2b and B2c.

Number of locations: The most serious plausible threats to Coolibah - Black Box Woodland are land clearing and changes to water regimes. Spatial patterns of land clearing show a high degree of contagion, with the best predictor of future clearing being the proximity of a patch to land parcels already cleared of native vegetation (Bedward et al. 2007). A broad interpretation of 'locations' under criterion B3 would be three jurisdictional zones with different regulatory controls on land clearing: the leasehold western Division of NSW; the freehold Central Division of New South Wales; and Queensland. A more narrow interpretation of locations based on neighbourhoods of contagion would produce an estimate of more than five. Small protected areas are excluded from these locations, as they are not threatened by land clearing. These areas were assessed by considering the next most serious plausible threat, changes to water regimes. As protected areas are located in at least two different subcatchments with different water management infrastructure, there are at least two further locations. Hence the most precautionary interpretation produces an estimate of five locations, although it is likely that there are more. Based on current rates of depletion due to land clearing (see criterion A1) and current rates of environmental degradation due to changes in water regime (see criterion C1), the ecosystem is unlikely to collapse or become Critically Endangered within the near future (c. 20 years). The status of the ecosystem is therefore Least Concern under criteria B3.

Criterion C

The principal mechanism of environmental degradation is through declines in hydrological processes related to extraction of water from rivers than flow through the floodplains that support the woodland ecosystem. The changes to water regimes and their effects are complex. There is evidence from gauging stations and modelled scenarios that median and mean river flows have reduced, that flows during small floods (average return interval <2 years) have been reduced more than flows during larger flood, that flooding is sustained for shorter durations (i.e. more rapid recession of flood waters), and that flows are less temporally variable as peak extraction in summer months coincides with timing of peak flows (Thoms & Sheldon 2000). Changes in the extent and duration of floods are likely to be the most direct measure of te extent and severity of hydrological degradation, given the importance of overbank flows for ecosystem function (see above). A temporal analysis of flood extent and duration is not available, however stream flow data provide a summary of average hydrological changes in catchments upstream, and hence a suitable proxy for assessing the severity of degradation under criterion C. Data from a flow gauge on the Darling River at Bourke was selected for analysis because of its position at the bottom of a catchment that contains almost the entire distribution of Coolibah - Back Box Woodland. Median flows provide an overall summary of general water availability in the system.

Current decline: Thoms & Sheldon (2000) used a hydrological model to simulate current stream discharge (with water extraction) and 'natural' discharge (without extraction). The model was evaluated by comparing modelled current flows with observed flows at the Bourke stream gauge and close correspondence was confirmed. Black et al. (1997) present a full description and evaluation of the model). For assessing criterion C1, it can be assumed that the ratio of current to natural flow represents change in flow over the past 50 years. This is a reasonable assumption because the first major river regulation infrastructure in the catchment was constructed in 1961 (Keepit Dam) and water extraction is likely to have been negligible prior to that year.

The flow volume at which the ecosystem would collapse is uncertain, but collapse is likely to occur before median daily flow declines to zero at Bourke (i.e. zero flow on 50% of days). Conservatively, it was assumed that the ecosystem would collapse if median flow declined to 0 - 10% of 'natural' (unregulated) levels. Thoms & Sheldon (2000) estimated that median flow at Bourke declined from 2917 ML/day (natural) to 1342 ML/day (current). Applying range standardisation (see Fig. 6 in main paper) gives a relative severity between 100 × (2917 - 1342)/(2917 - 0) = 54% and 100 × (2917 - 1342)/(2917 - 291.7) = 60%. As the flow gauge at the bottom of the catchment is indicative of range-

wide change in water regime, the extent of the decline is taken as 100%. The status of the ecosystem under criterion C1b is therefore Endangered.

Future decline: Future projections for flooding in the upper Darling catchment would need to take into account plausible scenarios of irrigation, environmental flows and climate change. No such projections are currently available. The status of the ecosystem is therefore Data Deficient under criterion C2.

Historic decline: As water extraction was assumed to be negligible prior to 1961, historic declines in the water regime are the same as current declines; with relative severity of 54 - 60 % over 100% extent. The status of the ecosystem is therefore Vulnerable under criterion C3.

Criterion D

Suitable variables for assessing declines in biotic interactions include vegetation responses to grazing, changes in structure due to tree thinning and ringbarking and the abundance of transformer invasive plants, particularly *Phyla canescens*. There are currently insufficient data available on these processes to assess the relative severity and extent of declines in biotic interactions under criterion D. The status of the community is Data Deficient under criteria D1, D2 and D3.

Criterion E

No quantitative analysis has been carried out to assess the risk of ecosystem collapse for Coolibah - Black Box Woodland. The status of the ecosystem is therefore Data Deficient under criterion E.

REFERENCES

Bedward M, Simpson CC, Ellis MV, Metcalfe LM. 2007. Patterns and determinants of historical woodland clearing in central-western New South Wales, Australia. Geographical Research 45: 348-357.

Benson JS, Allen, CB, Togher C, Lemmon J 2006. New South Wales vegetation classification and assessment. Part 1 Plant communities of the NSW western plains. Cunninghamia 9: 383-450.

Black D, Sharma P, Podger G. 1997. Simulation modelling for the Barwon–Darling river system for management planning. In Researching the Barwon Darling (eds. Thoms MC, Gordon A, Tatnell W). Canberra: CRC for Freshwater Ecology, p 34–43.

Boulton AJ, Lloyd LN 1992. Flooding frequency and invertebrate emergence from dry floodplain sediments of the river Murray, Australia. Regulated Rivers: Research and Management 7: 137-151.

Capon SJ 2003. Plant community responses to wetting and drying in a large arid floodplain. River Research and Applications 19: 509-520.

Capon SJ 2005 Flood variability and spatial variation in plant community composition and structure on a large arid floodplain. Journal of Arid Environments 60: 283-302.

Capon SJ, James C, Williams L, Quinn G. 2009. Responses to flooding and drying in seedlings of a common Australian desert floodplain shrub: Muehlenbeckia florulenta Meisn. (Tangled Lignum). Environmental and Experimental Botany 66: 178–185.

Earl J. 2003. The distribution and impacts of Lippia (Phyla canescens) in the Murray Darling System. Final report to the Lippia working group. Australian Cotton Cooperative Research Centre.

Fensham RJ 2008. A protocol for assessing applications to selectively clear vegetation in Australia. Land Use Policy 25: 249-258.

Good M, Price J Clarke P, Reid N. 2011. Densely regenerating coolibah Eucalyptus coolabah. woodlands are more species-rich than surrounding derived grasslands in floodplains of eastern Australian. Australian Journal of Botany 59: 468 - 479.

Humphries P, King AJ, Koehn JD. 1999. Fish, flows and floodplains: links between freshwater fishes and their environment in the Murray – Darling River system, Australia. Environmental Biology of Fishes 56: 151-163.

Lee AK, Mercer AH. 1967. Cocoon surrounded desert-adapted frogs. Science 157: 87-88.

Keith DA 2004. Ocean shores to desert dunes: the native vegetation of New South Wales and the ACT. NSW Department of Environment and Conservation, Sydney.

Keith DA, Orscheg C, Simpson CC, Clarke PJ, Hughes L, Kennelly SJ, Major RE, Soderquist TR, Wilson AL, Bedward M. 2009. A new approach and case study for estimating extent and rates of habitat loss for ecological communities. Biological Conservation 142: 1469-1479.

Kingsford RT, Auld KM. 2005. Waterbird breeding and environmental flow management in the Macquarie Marshes, arid Australia. River Research and Applications 21: 187-200.

Kingsford RT, Johnson W. 1998. Impact of water diversions on colonially-nesting waterbirds in the Macquarie Marshes of arid Australia. Colonial Waterbirds 21: 159-170.

Kingsford RT, Thomas RF. 1995. The Macquarie Marshes in arid Australia and their water birds: a 50 year history of decline. Environmental Management 19: 867-878.

McCosker RO. 1999. 'Gwydir Wetlands: Ecological Response to Flooding.' Landmax Pty Ltd, Kangaroo Point, Queensland.

NSW Scientific Committee. 2004. Final Determination. Coolibah - Black Box Woodland of the northern riverine plains in the Darling Riverine Plains and Brigalow Belt South bioregions as an Endangered Ecological Community. NSW Scientific Committee, Sydney. [http://www.environment.nsw.gov.au/determinations/CoolibahBlackBoxWoodlandEndSpListing.htm]

NSW Scientific Committee. 2009. Final Determination to reject a proposal to delist Coolibah - Black Box Woodland of the northern riverine plains in the Darling Riverine Plains and Brigalow Belt South bioregions as an Endangered Ecological Community. NSW Scientific Committee, Sydney. [http://www.environment.nsw.gov.au/determinations/coolibahblackboxrejectdelistfd.htm]

Price J, Berney P, Ryder D, Whalley W, Gross C. 2011. Disturbance governs dominance of an invasive forb in a temporary wetland. Oecologia: in press.

Price J, Macdonald M, Gross CL, Whalley W, Simpson I. 2011. Vegetative reproduction facilitates early expansion of Phyla canescens in a semi-arid floodplain. Biological Invasions 13: 285 - 289.

Price J, Whalley W, van Klinken R, Duggin J, Gross C. 2011. Periodic rest from grazing provided no control of an invasive perennial forb. The Rangeland Journal 33: 287 - 298.

Price J, Gross CL, Whalley W. 2010. Prolonged summer flooding switched dominance from the invasive wetland weed Lippia Phyla canescens. to native species in one small, ephemeral wetland. Ecological Management and Restoration 11: 61 - 63.

Queensland Environmental Protection Agency. 2009. Queensland wetland mapping and classification, Version 2.0, Queensland Government, Brisbane. [http://wetlandinfo.derm.qld.gov.au/wetlands/MappingFandD/WetlandMandDBackground.html]

Queensland Herbarium 2009. Regional Ecosystem Description Database REDD. Version 6.0b. Updated November 2009. Department of Environment and Resource Management, Brisbane.

Reid MA, Ogden R, Thoms MC. 2011. The influence of flood frequency, geomorphic setting and grazing on plant communities and plant biomass on a large dryland floodplain. Journal of Arid Environments 75: 815-826 .

Roberts J. 1993. Regeneration and growth of Coolibah, Eucalyptus coolabah subsp. arida, a riparian tree, in the Cooper Creek region of South Australia. Australian Journal of Ecology 18: 345-350.

Roberts J, Marston F. 2000. Water regime of wetland and floodplain plants in the Murray – Darling Basin. Technical report 30-00. CSIRO Land and Water, Canberra.

Robertson AI, Rowling RW. 2000. Effects of livestock on riparian zone vegetation in an arid Australian dryland river. Regulated Rivers: Research and Management 16: 527-541.

Sims NC. 2004. The landscape-scale structure and functioning of floodplains. PhD thesis, University of Canberra, Australia.

Taylor B, Ganf GG. 2005. Comparative ecology of two co-occurring floodplain plants: the native *Sporobolus mitchellii* and the exotic *Phyla canescens*. *Marine and Freshwater Research* **56**, 431-440.

Thoms MC. 2003. Floodplain – river ecosystems: lateral connections and the implications of human interference. Geomorphology 56: 335-349.

Thoms MC, Sheldon F 2000. Water resource development and hydrological change in a large dryland river: the Barwon-Darling River Australia. Journal of Hydrology 228: 10-21.

TSSC. 2011. Approved Conservation Advice for Coolibah – Black Box Woodlands of the Darling Riverine Plains and the Brigalow Belt South Bioregions ecological community. Australian Government: Canberra.

10 MOCK OLIVE - WILGA - PEACH BUSH - CARISSA DRY SUB-TROPICAL SEMI-EVERGREEN VINE THICKET IN SOUTH EASTERN AUSTRALIA.

Contributed by J.S. Benson, Royal Botanic Gardens and Domain Trust, Sydney Australia, January 2012.

CLASSIFICATION

International Terrestrial Vegetation Classification (adapted from Faber-Langendoen *et al.* 2012): Terrestrial Natural Vegetation: Formation Class: Forest – Woodland / Formation Subclass: Tropical Forest / Formation Tropical Seasonally Dry Forest / Division: Sub-tropical Dry Rainforest / Alliance: Semi-evergreen Vine Thicket / Association (Colloquial Name)**:** Mock Olive - Wilga - Peach Bush - Carissa dry sub-tropical semi-evergreen vine thicket in south eastern Australia. Dominant plant species**:** *Notelaea microcarpa var. microcarpa - Geijera parviflora - Ehretia membranifolia - Elaeodendron australe var. integrifolium / Carissa ovata - Beyeria viscosa - Spartothamnella juncea - Solanum parvifolium / Austrostipa verticillata - Panicum queenslandicum var. queenslandicum - Austrodanthonia bipartita - Dichondra sp. A.*
Level of Classification: Association.
IUCN Habitats Classification (version 3.0): 1. Forest / 1.5 Subtropical – Tropical Dry
Biogeographic Realm: Australasian, New South Wales (NSW)
Key references: Defined as plant community ID147 in the NSW vegetation classification and assessment database of Benson *et al*. 2010. Detailed analysis of plant species composition and regeneration ecology is documented in Curran (2006). Assessment of avifauna for part of the distribution is provided in Holmes (1979). Fauna is described for a key site in (NSW NPWS 2004).

ECOSYSTEM DESCRIPTION

Characteristic Flora and Vegetation

Mid-high to low closed or open forest known as semi-evergreen vine thicket dominated by rich diversity of low trees and shrubs to about 6 m high (Figs. 1 - 3). A full characteristic plant species list is provided in NSW plant community ID147 in Benson *et al.* (2010) and biological survey information is held on the NSW National Parks and Wildlife Service administrative file 1992/M/3793 (NSW Government archives). Trees include Mock Olive (*Notelaea microcarpa var. microcarpa*), Wilga (*Geijera parviflora*), Peach Bush (*Ehretia membranifolia*) along with *Elaeodendron australe* var. *integrifolium, Ventilago viminalis, Psydrax oleiofolia, Alectryon subdentatus* and *Alstonia constricta.* Some tree species are facultatively deciduous. Emergent trees to 15 m high are often present including White Box (*Eucalyptus albens*), Silver-leaved Ironbark (*Eucalyptus melanophloia*), White Cypress Pine (*Callitris glaucophylla*) and Belah (*Casuarina cristata*). The shrubs layer may be mid-dense or dense and includes *Carissa ovata, Beyeria viscosa, Spartothamnella juncea, Solanum parvifolium, Rhagodia parabolica, Olearia elliptica, Senna coronilloides, Indigofera adesmiifolia, Indigofera brevidens, Breynia cernua, Solanum semiarmatum, Cassinia laevis, Myoporum montanum, Capparis lasiantha, Pimelea neo-anglica* and *Phyllanthus subcrenulatus.* Vines are common and include Wonga Vine (*Pandorea pandorana*), *Parsonsia eucalyptophylla, Clematis microphylla var. microphylla, Cayratia clematidea* and *Jasminum lineare*. Mistletoes include *Lysiana exocarpi, Lysiana subfalcata* and *Amyema miraculosum*. The ground cover is mid-dense in open areas or sparse under dense tree or shrub canopies. Common grass species include *Austrostipa verticillata, Leptochloa asthenes, Poa sieberiana* var. *hirtilli, Elymus scaber, Panicum queenslandicum var. queenslandicum, Chloris ventriculosa, Austrodanthonia bipartita, Paspalidium gracile* and *Cymbopogon refractus*. The sub-shrub *Desmodium brachypodum* is often abundant. Forbs include *Boerhavia dominii* and *Dichondra* sp. *A*. Sedges such as *Carex inversa* may be present along with the rock fern *Cheilanthes sieberi* subsp. *sieberi*. Planchonella Hill Nature Reserve contains a population of the NSW restricted small tree *Pouteria cotinifolia* var. *pubescens* (Fig. 2). This ecosystem grades into *Eucalyptus albens* (White Box)

or *Casuarina cristata* (Belah) woodlands. A more complex SEVT community occurs in more tropical climes to the north in Queensland.

Characteristic Fauna

Fauna surveys have been undertaken the most complex remaining patch in Planchonella Nature Reserve (NSW NPWS 2004). This area contains one of the few known southern Australian records of the mammal Black-striped Wallaby (*Macropus dorsalis)*, an endangered species under the *NSW Threatened Species Conservation Act, 1995.* Other endangered fauna include the birds Bush Stone-curlew (*Burhinus grallarius*) and the Squatter Pigeon (*Geophaps scripta*). Vulnerable species include Glossy Black-Cockatoo (*Calyptorhynchus lathami*), Turquoise Parrot (*Neophema pulchella*), Brown Treecreeper (*Climacteris picumnus*), Speckled Warbler (*Pyrrholaemus sagittatus*), Black-chinned Honeyeater (*Melithreptus gularis gularis*) and the Yellow-bellied Sheathtail Bat (*Saccolaimus flaviventrus*). Some species reach their inland limit including the White-browed Scrubwren (*Sericornis frontalis*), the Rainbow Lorikeet (*Trichoglossus haematodus*) and the Chocolate Wattled Bat (*Chalinolobus morio*). Others reach their eastern Australian limit including the White-winged Fairy Wren (*Malurus leucopterus*), Singing Honeyeater (*Lichenostomus virescens*), Spotted Bowerbird (*Chlamydera maculata*) and the Black-faced Woodswallow (*Artamus cinereus*). Other common bird species include Wedge-tailed Eagle (*Aquila audax*), Nankeen Kestrel (*Falco cenchroides*), Bar-shouldered Dove (*Geopelia humeralis*), Redwinged Parrot (*Aprosmictus erythropterus*), Fan-tailed Cuckoo (*Cacomantis flabelliformis*), Southern Boobook (*Ninox boobook*), Sacred Kingfisher (*Todiramphus sanctus*), White-throated Treecreeper (*Cormobates leucophaeus*), and the Spinycheeked Honeyeater (*Acanthagenys rufogularis*). Reptiles and amphibians species such as the Soft-tailed Gecko (*Diplodactylus williamsi),* Bynoe's Gecko (*Heteronotia binoei*), Green Tree Frog (*Litoria caerulea*), Lace Monitor (*Varanus varius*), Eastern Brown Snake (*Pseudonaja textilis*), Shingleback Lizard (*Trachydosaurus rugosus*) and skinks including *Ctenotus robustus*, *Anomalopus leuckartii*, *Carlia vivax*, *Lygisaurus foliorum*, *Cryptoblepharus virgatus*, and *Morethia boulengeri*.

The dense vegetation of the vine thicket provides a refuge for smaller macropods such as the Red-necked Wallaby (*Macropus rufogriseus*) and the Swamp Wallaby (*Wallabia bicolor*) (Henderson, 1997). Other mammals occupying the Reserve include the Common Brushtail Possum (*Trichosurus vulpecula*), Eastern Grey Kangaroo (*Macropus giganteus*), Gould.s Long-eared Bat (*Nyctophilus gouldi*) and Lesser Long-eared Bat (*Nyctophilus geoffroyi).*

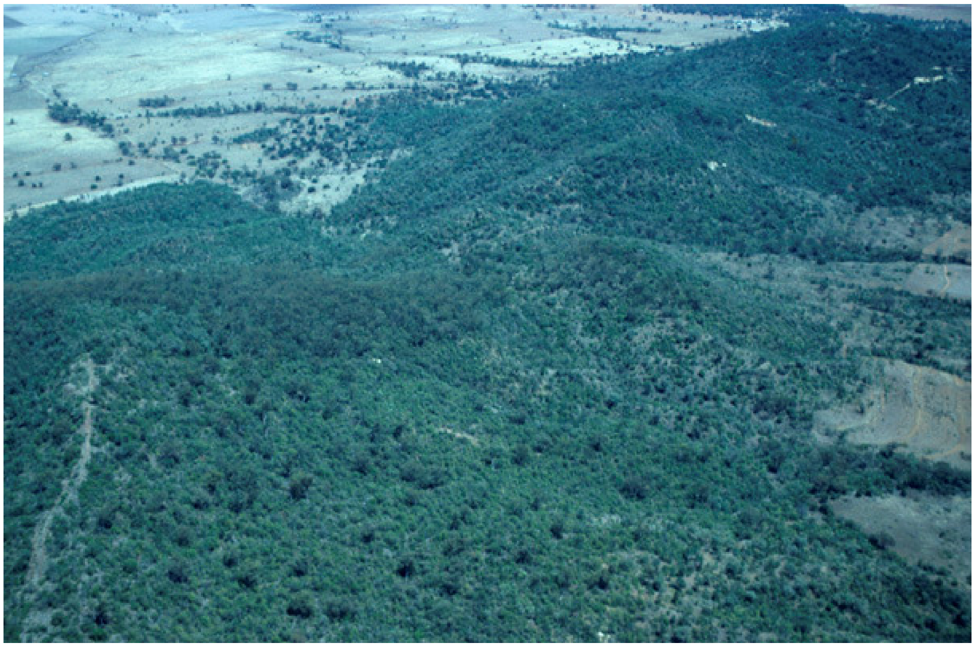

Figure S10. 1. Aerial view of the largest known stand of Mock Olive - Wilga - Peach Bush - Carissa semi-evergreen vine thicket in New South Wales on Derra Derra Ridge near Bingara, North Western Slopes of NSW, south-eastern Australia, 1994, J.S. Benson.

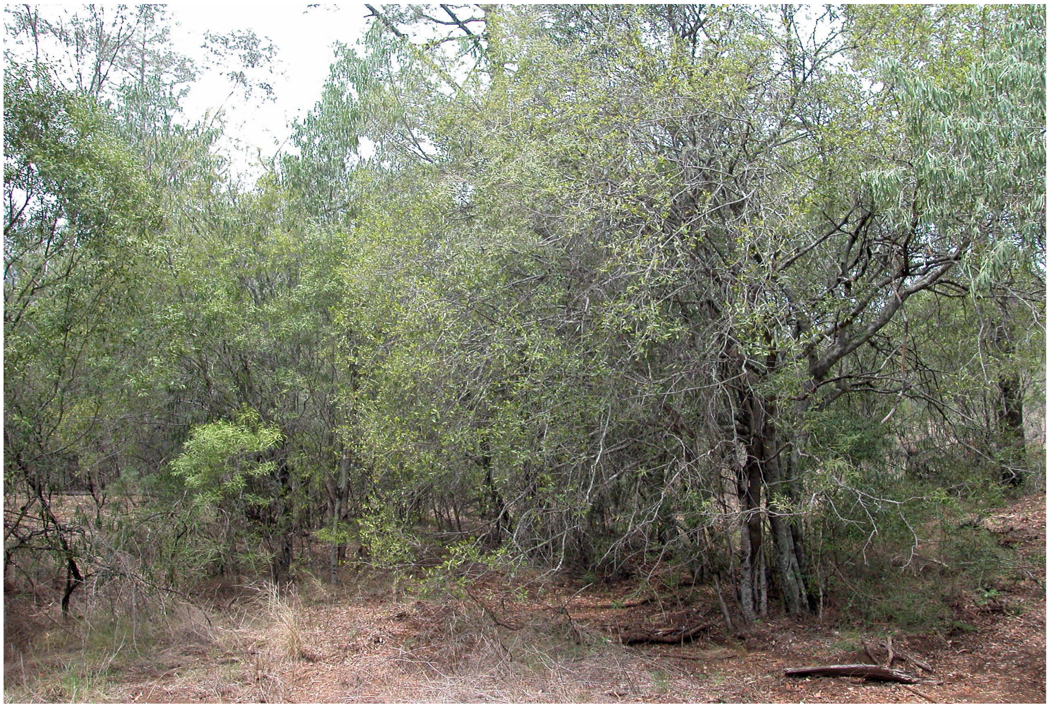

Figure S10. 2. Semi-evergreen vine thicket dominated by the restricted small tree *Pouteria cotinifolia* var. *pubescens* in Planchonella Hill Nature Reserve, near Yetman, north-west NSW, 2007, J. Benson.

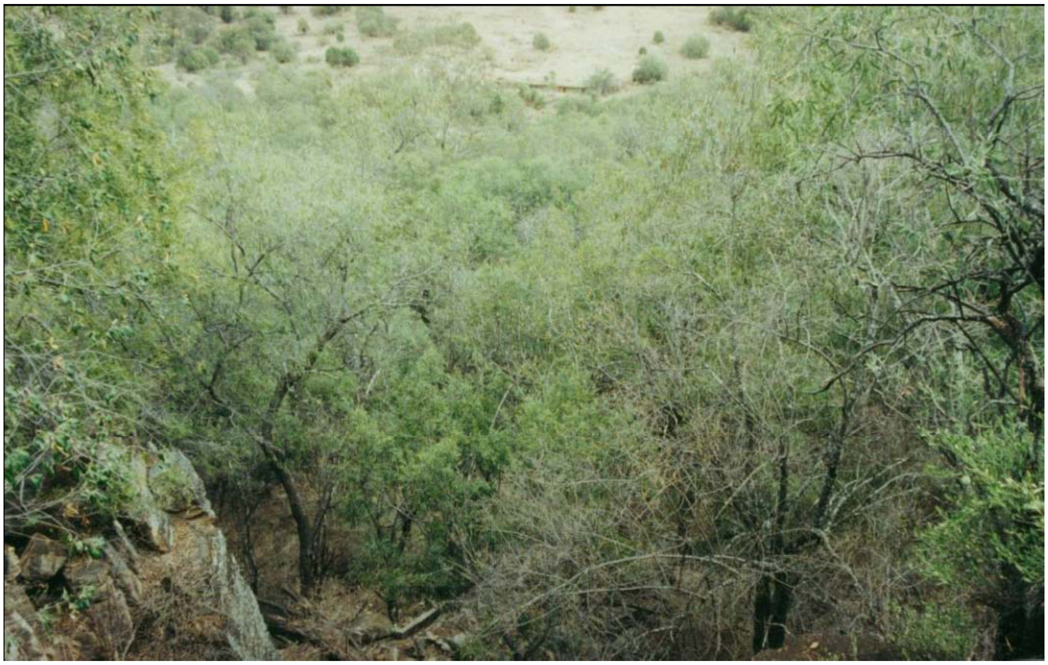

Figure S10. 3. Canopy of *Notelaea microcarpa–Geijera parviflora–Ehretia membranifolia* semi-evergreen vine thicket on Porcupine Hill, near Gunnedah, New South Wales, 2004, T. Curran.

Abiotic environment and Distribution

Dry subtropical SEVT occurs on high nutrient, chocolate brown or black loam soils often derived from basalt on flats, rises, or low hill landforms. Altitude of occurrences ranges from 450-700m a.s.l. The climate is warm to hot with low to moderate rainfall averaging 570-750mm p.a across the distribution. Mean maximum monthly temperatures range from 32-35°C with mean minimum monthly temperatures 0.2-4.6° C (Curran 2006). Heatwaves of over 40°C occasionally occur. Therefore, this "dry" relictual rainforest has adapted to limited precipitation and summer heat of inland mountain range slopes in south-eastern Australia. The limited extent of this ecosystem may be explained by contractions of extent during the Pleistocene ice ages including the last severe ice age that reached its maximum c 20,000 B.P. Fire history may also explain its rarity and location of occurrences. Frequent and intense fire would egrade or eliminate this rainforest leading to replacement by fire-tolerant *Eucalyptus*-dominated woodlands. Today, Eucalyptus woodland surrounds most patches of this dry rainforest. Little is known about the impacts on this ecosystem of Aboriginal burning practices prior to European settlement in south-eastern Australia in 1788.

This southern type of sub-tropical vine thicket occurs on the western slopes of the Great Dividing Range in south-eastern Australia in the State of New South Wales in the Brigalow Belt South and Nandewar Australian Bioregions (Floyd 1990, Curran 2006, Benson *et al*. 1996). Its distribution is bounded by the towns of Gunnedah in the south, Yetman in the north and Narrabri and Moree in the west. SEVT stands in the Yalloroi - Warialda region were surveyed by Holmes (1979). By that time it was considered that most of the original extent had been cleared based on field observations of its current extent in relation to suitable substrates and landforms (Holmes 1979). Locations south of the study area in Holmes (1979) were identified in Benson et al. (1996) using 1994 aerial photographs and light aircraft aerial survey. The 1994 aerial survey took place one year before land clearing control laws were introduced in New South Wales.

The estimated pre-industrial (pre-1750) and pre-European settlement (1788) extent of the ecosystem over its full distribution is estimated to have been in the order of 12000 hectares (Benson et al. 2010). This is based on areas of cleared land that contain basalt substrate with topographical features similar to current remnants. It is estimated that less than 20% of the pre-1750 extent remains. The current extent is estimated as being approximately 2000 hectares based a combination of surveys and mapping covering the distribution (summarised in Benson et al. 2010). Holmes (1979) documents patches in the north – less than 500 ha; Benson et al. (2006) document between 500-1000 ha in the mid-south area; Peasley (2001) maps 97 hectares east of Moree; Cannon et al. (2002) map some areas near Narrabri; small areas exist near Gunnedah at its southern-most distribution based on survey data in Curran (2006). Most occurrences other than the largest stands at Derra Derra Ridge and Planchonella Hill (Fig. 4) are one to a few hectares in size. These small patches are vulnerable to clearing, grazing by domestic stock and feral goats.

Overall, there are less than 25 occurrences of remaining. 12 are in the north to central parts of the distribution (Fig. 4), a few occur in the south near Gunnedah and a few to the west near Narrabri and Moree.

Threatening Processes

Most of this community has been cleared because it occurs on high nutrient soils on undulating terrain suitable for grazing and cropping, particularly for growing wheat. Domestic stock and feral animal (goats) graze the understorey of remnants outside conservation reserves and this effects the regeneration of some tree and shrub species. Extended drought and increased temperatures due to climate change could negatively impact on he regeneration and survival of some charcteristic plant species. Invasive weeds include the Prickly Pear species *Opuntia stricta* and *Opuntia tomentosa*. Rare stochastic disturbance events such as wild fire would kill rainforest species and change the functioning of the ecosystem.

Protected area status: Two conservation reserves contain this ecosystem. 176 ha are mapped in Planchonella Hill Nature Reserve (Hunter 2006) and 320 ha are mapped in Bingara State Conservation Area (Derra Derra Ridge) (Hunter 2009). In total 496 ha are protected representing 4% of original extent and 25% of the current extent (Benson et al. 2010).

Conservation ecosystem listings

Listed by the NSW Scientific Committee (1999) as an ENDANGERED Ecological Community under the NSW Threatened Species Conservation Act 1995: *Semi-evergreen Vine Thicket in the Brigalow Belt South and Nandewar Bioregions.*

Listed as ENDANGERED as part of a Threatened Ecological Community under the Australian Government Environmental Protection and Biodiversity Conservation Act 1999: *Semi-evergreen vine thickets of the Brigalow Belt (North and South) and Nandewar Bioregions.*

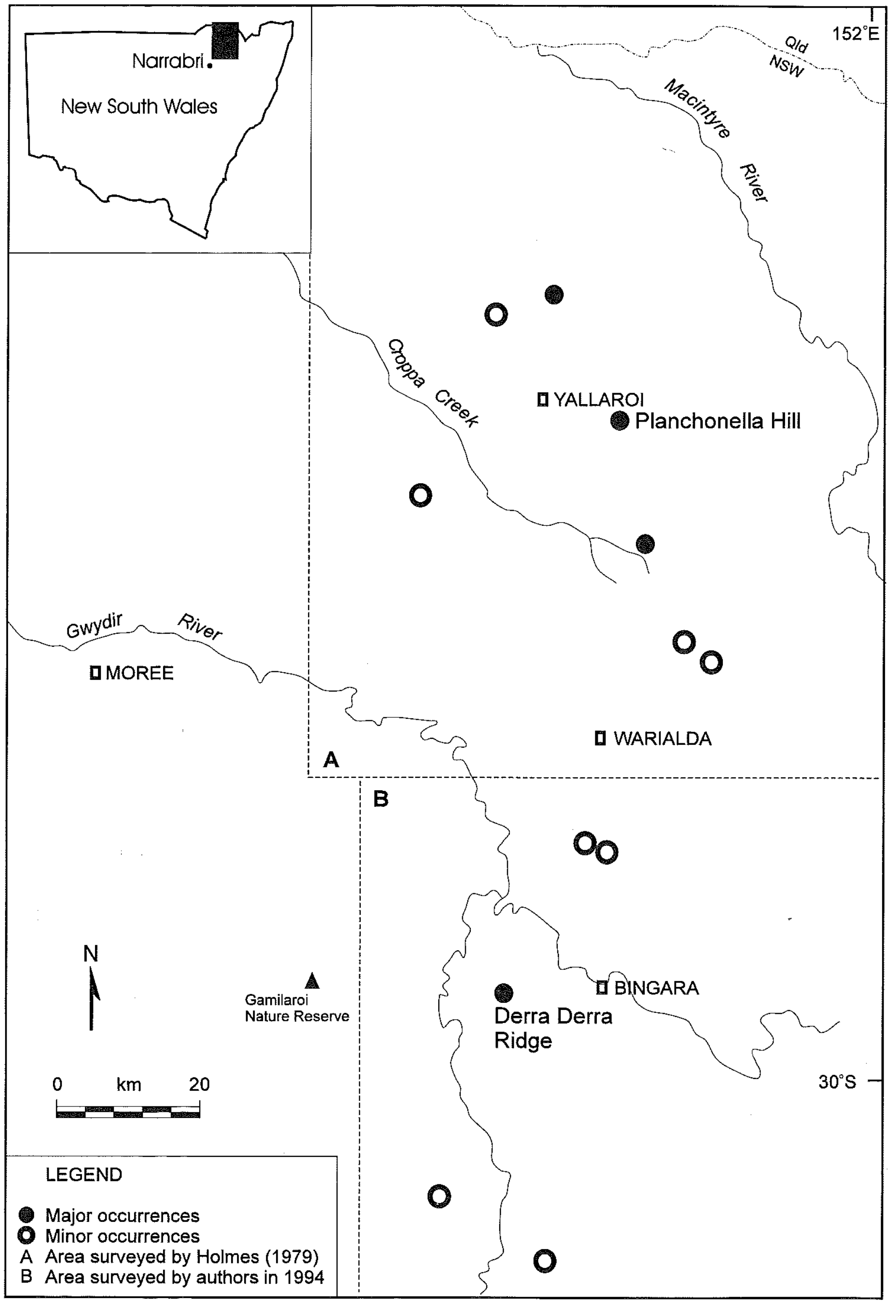

Figure S10. 4. Northern and central occurrences of *Mock Olive - Wilga - Peach Bush - Carissa dry sub-tropical semi-evergreen vine thicket* mapped by Holmes (1979) and Benson et al (1996) in New South Wales, Australia. Derra Derra Ridge and Planchonella Hill contain the largest areas.

Ecosystem collapse

For assessment of criteria A and B, the vine thicket ecosystem was assumed to collapse when its mapped distribution declines to zero due to conversion to agriculture.

ASSESSMENT

Summary

Criterion	A	B	C	D	E	overall
subcriterion 1	NE	NE	NE	NE	DD	EN
subcriterion 2	NE	EN	NE	NE		
subcriterion 3	EN	NE	NE	NE		

Criterion A

Current and future decline. The rate of extent change over 50 years is difficult to accurately establish from existing, disparate mapping. A range of vegetation maps and field observations from the late 1970s to 2002 indicate continued loss of extent due to land clearing, however, the rate of decline has not yet ben quantified. Land clearing may have no slowed down due to legislative changes in NSW. The status under Criteria A1 and A2 is Not Evaluated.

Historic decline. Vegetation surveys mapping locations (Holmes 1979, Benson et al. 1996, Peasley 2001) infer loss of >70% of the pre-1750 extent, supported by the level of clearing of mapped landforms of similar soils and topography within the extent of occupancy of the ecosystem. The status under criterion A3 is Endangered.

Criterion B

Extent of Occurrence. Not Evaluated.

Area of Occupancy. There are less than 25 total occurrences in 10 x 10 km cells, of which less than 10 contain more than 1% vine thicket. There is observed/suspected continuing degradation of spatial extent and biotic interactions (Criteria B2a. i & iii) caused by small-scale land clearing and grazing of the understorey / ground cover that is inhibiting regeneration of some characteristic plant species and affecting vegetation structure. The small area of occupancy, combined with continuing degradation supported Endangered status under criterion B2.a. i & iii.

Number of locations. Not Evaluated.

Criterion C

Not Evaluated, although there is observational evidence of soil erosion at some sites.

Criterion D

Not Evaluated, although there is observational evidence of negative impacts of domestic stock and feral animal grazing on the regeneration of character plant species.

Criterion E

Data Deficient, no model available for quantitative analysis of ecosystem collapse.

REFERENCES

Benson JS, Dick R, Zubovic A. 1996. Semi-evergreen vine thicket vegetation at Derra Derra Ridge, Bingara, New South Wales. Cunninghamia 43: 433-530.

Benson JS, Richards P, Waller S, Allen C. 2010. New South Wales Vegetation classification and Assessment: Part 3 Plant communities of the NSW Brigalow Belt South, Nandewar and west New England Bioregions and update of NSW Western Plains and South-western Slopes plant communities, Version 3 of the NSWVCA database. *Cunninghamia* 114: 457-579.

Cannon G, Cannon M, Harding W, McCosker R, Spunner B, Steenbeeke G, Watson G. 2002. Native vegetation map report No 3: Bellata, Gravesend, Horton and Boggabri 1:100 000 map sheets NSW Department of Land and Water Conservation.

Curran TJ. 2006. Rainforest, drought and soil type: Phytogeography and functional and evolutionary ecology of dry rainforest on the western slopes of New South Wales. Thesis submitted for the degree of Doctor of Philosophy of The University of New England.

Curran TJ, Clarke PJ, Bruhl JJ. 2008. A broad typology of dry rainforests on the western slopes of New South Wales. Cunninghamia 10: 381-407.

Faber-Langendoen D, Keeler-Wolf T, Meidinger D, Tart D, Josse C, Navarro G, Hoagland B, Ponomarenko S, Saucier J-P, Weakley A, Comer P. 2012. Guidelines for a vegetation – ecologic approach to vegetation description and classification. Hierarchy Revisions Working Group, Federal Geographic Data Committee. FGDC Secretariat, U.S. Geological Survey, Reston, VA, and NatureServe, Arlington, VA.

Floyd AG. 1990. Australian Rainforest in New South Wales, volumes 1 & 2. Surrey Beattie & Sons Pty Limited: Sydney.

Holmes G. 1979. Preliminary survey of vegetation remnants and associated avifauna on basaltic soils on the North Western Slopes of New South Wales. Unpublished. NSW National Parks and Wildlife Service: Hurstville.

Hunter JT. 2006. Vegetation and floristics of Planchonella Nature Reserve. Report to NSW National Parks and Wildlife Service.

Hunter JT. 2009. Vegetation and florisitics of Derra Derra section of the Bingara State Conservation Area. Report to NSW National Parks and Wildlife Service.

NSW National Parks and Wildlife Service 2004. Planchonella Hill Nature Reserve Plan of Management NSW NPWS: Hurstville, Australia.

NSW Scientific Committee 1999. Final determination. Listing of Semi-evergreen Vine Thicket as an endangered ecological community under the Threatened Species Conservation Act. NSW Scientific Community: Hurstville.

Peasley B. 2001. Vegetation map of Moree Plains Shire. Department of Land and Water Conservation: Inverell.

11 TEPUI SHRUBLANDS, SOUTHERN VENEZUELA

contributed by María A. Oliveira-Miranda[1], Jon Paul Rodríguez[1,2] and Sergio Zambrano-Martínez[2].
[1]Provita, Caracas, Venezuela; [2]Centro de Ecología, Instituto Venezolano de Investigaciones Científicas, Caracas, Venezuela.

CLASSIFICATION

Tepui shrublands are included in the Pantepui, a phytogeographical province totaling 6,000 km^2 formed by grouping all table mountain (tepui) summits above 1500 m. Pantepui is part of the Guayana Shield, one of the oldest continental areas in the western hemisphere (Zinck & Huber 2011). They were not recognized as a distinct primary vegetation community until the 1980s, when they were first included as discrete units in vegetation maps (Huber & Alarcon 1988, Huber 1995a, 1995b, Riina & Huber 2003).
IUCN Habitats Classification Scheme (Version 3.0): 3. Shrubland / 3.6 Subtropical / Tropical Moist
Key references: Huber 1995a, 1995b, Oliveira-Miranda et al. 2010.

ECOSYSTEM DESCRIPTION

Characteristic native biota

Tepui shrublands (Fig. 1) are plant communities that occur on tepui summits, characterized by high physiognomic diversity, low species diversity and high degree of endemism. "Tepui" is a native term referred to the table mountains located primarily on the Venezuelan portion of the Guayana Shield (Huber 1995a), mostly above 1500 m. Phytogeographically, Tepui shrublands are one of the main habitats of the Pantepui floristic province (Huber 1987), and can also be called Pantepui scrub (Huber 1995a).

The composition, diversity and distribution of Tepui shrublands (Fig. 1a) are conditioned by edaphic constraints (Huber 1989), with many plant species restricted to particular substrate types such as rock outcrops, sandy soils or peat. Their physiognomy may vary between tepui summits, ranging from 0.5-1 m tall in Guanay-tepui and Auyan-tepui, to 8 m tall on top of Jaua Sarisariñama (Riina & Huber 2003).

Broadly speaking, the shrubs found in Tepui shrublands produce branches that originate at their base. They have coriaceous or sclerophyllous leaves, that are usually densely aggregated at the end of their branches. Typically, they also have conspicuous flowers or inflorescences (Huber 1995a). These shrublands may build up to large formations, such as those found on Huachamacari and Duida (Fig. 1b, c), or appear in small depressions, such as those on top of the tepuis (Riina & Huber 2003). Common plant genera found on tepui shrublands include *Bonnetia* (Theaceae), *Maguireothamnus* and *Pagameopsis* (Rubiaceae), *Tepuianthus* (Tepuianthaceae) and others from Ochnaceae, Ericaceae, Malpighiaceae and Melastomataceae families (Riina & Huber 2003). More than 70 plant taxa are endemic to the Tepui summits, including several endemic genera (Rull & Vegas-Vilarrubia 2006).

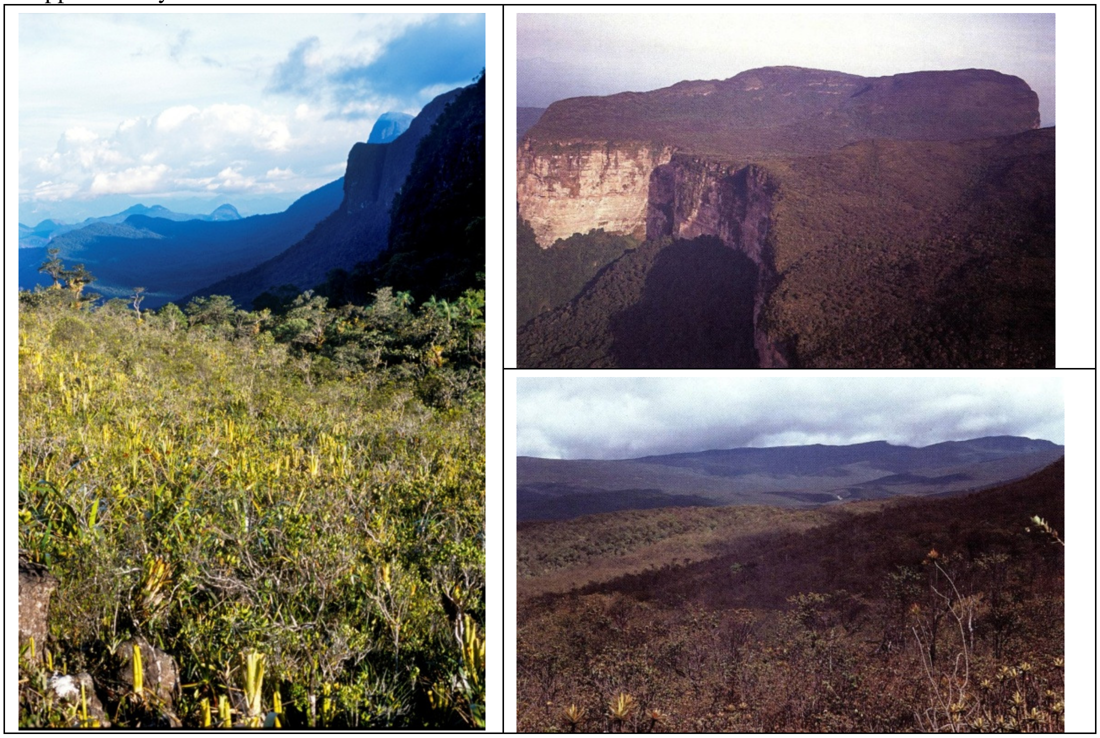

Figure S11. **1.** From left to right: (a) Tepui shrubland on Cerro Sipapo, Amazonas, Venezuela. Photo: Gustavo Romero. (b) (top) Tepui shrubland on Cerro Huaramacachi, Amazonas, Venezuela. Photo: Otto Huber. Photo 3. (c). Tepui shrubland on Cerro Duída, Amazonas, Venezuela. Photo: Otto Huber.

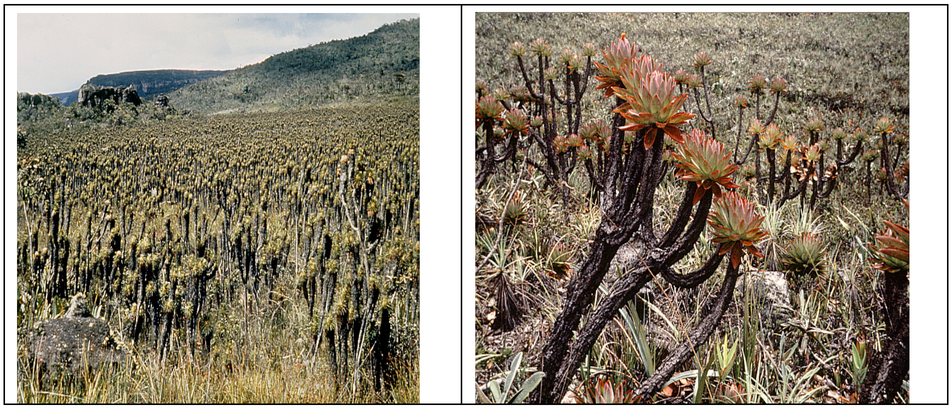

Figure S11. 2. (a) Left. Tepui shrubland on Chimanta massif, showing a paramoid shrubland (*Chimantaeamirabilis*), photo: Otto Huber. (b) Right. Caulirosulate shrubland on Serranía La Neblina (*Bonnetia maguireorum*), photo: Susanne Renner.

Several peculiar types of shrubs grow in the Venezuelan tepui shrublands, such as the paramoid and caulirosulate (which hold the shape of an artichoke). The paramoid (Fig. 2a) and caulirosulate scrublands are found on the Chimantá massif. The paramoid receive their name from their physiognomic and floristic similarity with Andean shrublands (known as “páramos”) (Huber 1992). The shrubs with caulirosulate growth form (Fig. 2b) are also found on the summit of Serranía La Neblina, at the southernmost tip of Venezuela. Both shrubland types develop dense communities formed by thousands of individuals of the *Chimantaea* genus, on the Chimantá massif, and by the species *Bonnetia maguireorum* on Serranía La Neblina (Huber 1992, 1995a, Steyermark *pers. comm.*).

The vertebrate fauna is also highly distinctive, with numerous amphibians, reptiles, birds and mammals exclusive or almost restricted to the tepuis (Señaris et al. 2009). For example, the sira poison frog (*Allobates rufulus*; Amphibia: Aromobatidae), *Stefania ginesi* (Amphibia: Hemiphractidae) and *Thamnodynastes chimanta* (Reptilia: Colubridae), have only been found on the summit of the Chimantá massif (Gorzula 1992). Likewise, over 30 bird species are endemic to Pantepui, primarily from the order Passeriformes, but also Apodiformes, Psittaciformes, Caprimulgiformes, and Tinamiformes (Molina & Salcedo 2009).

Tepui shrublands, as well as other tepui communities, were considered biologically isolated units for a long time (Maguire 1970; Brewer-Carías 1978; George 1988). However, in many tepuis, especially on the larger massifs of Venezuelan Guayana, the summits are not completely isolated from the surrounding lower vegetation belts by vertical walls (Huber 1995a). Total physical isolation of tepui summits occurs only in a few tower-like mountains, such as Roraima-tepui, Ilú-tepui, and Los Testigos massif, where impoverished plant communities prevail.

Abiotic environment

Tepui shrublands are mostly associated with the slopes and summits of the tepuis, on granite and pink sandstone formations, dating back to the Precambrian period (Huber 1995 veg). Although the Tepui shrubland ecosystem appears sporadically at altitudes of 800-1,500 m, with a mesothermic climate (12-24ºC), the best developed and densest communities are found on higher grounds (1,500-3,000 m), with submicrothermic climate (6-12ºC). At all elevations, Tepui shrublands are exposed to high air humidity and precipitation, greater than 2000 mm (Huber 1989; Riina & Huber 2003). With the exception of soils with high organic content, these communities develop on rocky soils (sandstone or granite), where in spite of high precipitation, moisture conditions are limited due to the low water-retention capacity of the substrate and excessive draining (Riina & Huber 2003), as a consequence of strong winds and high solar radiation periods (Huber 1995a, Zinck & Huber 2011).

Distribution

Typical montane tepui shrublands are only found on higher elevations of the Guayana Shield, mainly in the States of Bolivar and Amazonas in southern Venezuela (Figure 3), on two tepui summits in adjacent Guyana and, to a very small extent, on the Brazilian section of Cerro de la Neblina (Huber 2012, *pers. comm.*). At lower elevations (800-1500 m), other peculiar scrub types are growing with different floristic composition and growth forms, such as in the Gran Sabana, Cerro Guaiquinima, or Cerro Sarisariñama,where they could be considered as "subtepuian" vegetation (Huber 1995a), but they are not yet sufficiently well explored.

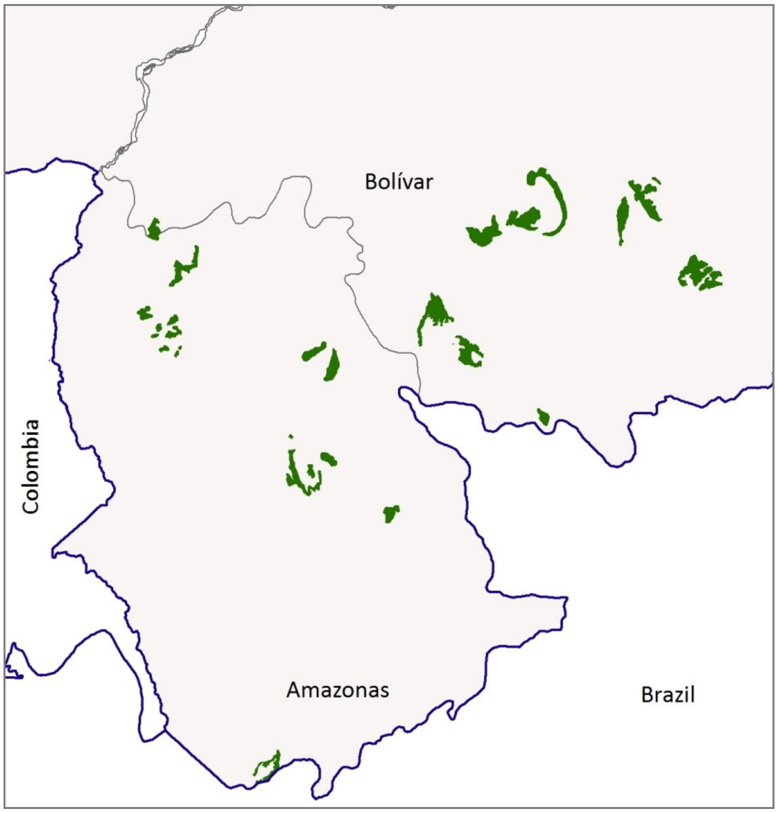

Figure S11. 3. Geographic distribution of Venezuelan tepui shrublands. They are restricted to the States of Amazonas and Bolívar.

Key processes and interactions

Venezuelan Tepui shrublands (> 1,500 m) correspond to the montane life zone, which is present in all tropical and extra-tropical mountains above the tree line, such as the páramos in the Andes and the alpine meadows of the Alps and the Rocky Mountains (Huber 1995a). Furthermore, as mentioned above, tepuis are not actually isolated from lowland landscapes (Huber 1989; 1995a). Extremely adverse environmental conditions such as lack of soil, nutrient deficiency, strong winds, and high radiation appear as the main cause of these relatively depauperate communities (Huber 1995a) and play key roles in the evolution and ecology of the ecosystem. For example, sclerophyllous foliage and diversity of carnivorous plants are likely evolutionary responses to nutrient deficient soils that characterize the old stable landscapes in which these shrublands occur (Hopper 2009). Like other sclerophyllous shrublands, the tepui are prone to recurring fires (e.g. Givinsh et al. 1986), although these appear to be infrequent and their role in ecosystem dynamics is poorly understood.

Threatening processes

The Tepui shrublands have few known processes that threaten their persistence. Human effects are limited to localized impacts in the tepuis more visited by tourists, or to damage caused by the arrival of groups (tourists or scientists) in helicopters or small planes. Among the most common adverse effects are the disposal of garbage, fuel cans and other items left by visitors in their temporary camps (Huber 1995c). Nevertheless, in many tepuis there are clear signs of deterioration of the vegetation due to trampling, helicopter landing, rock-climbing and rappelling, paragliding, introduction of exotic species and accumulation of rubbish. This damage, although limited to relatively small areas, is almost irreversible, as it has been shown that the affected vegetation recovers very slowly (Gorzula & Huber 1992; MARN 2000; Riina & Huber 2003).

The second process currently considered a threat to the persistence tepui shrubland is global climate change. A small range of tolerance of the component species to changes in humidity and temperature could be a clear weakness of the system (Huber 1995c; Rull et al. 2005; Rull et al. 2009), but additional research is necessary to support this assumption. On the other hand, it is estimated that an increase of 2-4°C in average temperatures could cause the extinction of 10% to 30% of the plants endemic to the tepuis before the end of the century (Rull & Vegas-Vilarrubia 2006). For eight species of *Chimantaea*, it is predicted that two will completely lose their habitat and six would be very close to the critical limit. Although their habitat would not disappear completely, it would be reduced to a few tiny isolated areas in the highest summits (Rull et al. 2005). However, there are no data available either on the resistance nor the resilience capacity of any tepui plant or animal species to climatic change, so such assumptions must be considered carefully (Huber 2012, *pers. comm.*).

Ecosystem collapse

For assessment of criteria A and B, Tepui shrublands were assumed to collapse if their mapped distributiuon declines to zero. Species composition and richness of Tepui shrubland vegetation were selected as suitable variables for assessing biotic processes and interactions under criterion D. As no trends were evident, collapse thresholds were not specified.

ASSESSMENT

Summary

Criterion	A	B	C	D	E	Overall
subcriterion 1	LC	LC	NE	LC	DD	LC
subcriterion 2	LC	LC	NE	DD		
subcriterion 3	LC	LC	NE	LC		

Criterion A

Current and future decline: There is no evidence of measurable reductions in the distribution of Tepui shrublands over the last 50 years, considering botanical, faunal and geological expeditions carried out since the 1950s. Studies performed comparing recent and historical maps based on Landsat and radar images (currents and from the early eighties) did not detect any appreciable reduction of spatial occurrence of the Venezuelan tepui shrublands in the past ~25 years (Huber & Oliveira-Miranda 2010; Oliveira-Miranda et al. 2010). The current knowledge of the spatial distribution of this ecosystem in Venezuela, however, is better than previous estimates which were calculated very roughly (Huber & Alarcon 1988; Huber 1995), so some degree of uncertainty regarding their exact past extent still remains. Despite this uncertainty, the available data strongly support a classification of Least Concern under A1.

We do not expect any major change in the next 25 years, as all Tepui shrublands are included in National Parks and Natural Monuments, and they are fairly remote, located relatively far away from major human settlements. Changes in distribution due to landuse or exploitation are therefore unlikely in the short to medium term. Within a 50-year period that represents 25 years into the past and 25 years into the future, the distribution of tepui scrublands is expected to remain stable and well below the threshold for Vulnerable under criterion A2b, and therefore they are considered Least Concern.

Global climate change, is a possible threat for the future persistence of Venezuelan Tepui shrublands, given their temperature and humidity constraints (Huber 1995; Rull et al. 2005; Rull & Vegas-Vilarrubia 2006; Rull et al. 2009). Some individual species are projected to decline under future climates, but these estimates are for the end of the century, a period longer than 50 years into the future (Rull et al. 2005). However, there are no models or projections of change in extent at the community level. In terms of critierion A2a, therefore, these data are not adequate for performing an assessment, leading to a listing as Data Deficient.

Despite the lack of information for assessing criterion A2a, sufficient data on recent and future decline of this ecosystem exist to support a classification of Least Concern under criteria A1 and A2b.

Historic decline: To the best of our knowledge, the distributions of the ecosystems associated with summits of tepuis have not significantly declined since 1750. There is no evidence that tepui shrublands once occupied significant areas of land that are now dominated by human land use (Huber 1995c). Although indigenous peoples have lived in the area for centuries, tepuis have been considered sacred and therefore object of minimal intervention. The status of the ecosystem is therefore Least Concern under criterion A3.

Criterion B

The current mapped area of Venezuelan Tepui shrublands, estimated from Landsat satellite images, is approximately 5,170 km^2 (Figure 3). They naturally occur in ~35 locations (patch size range: 4-456 km^2) associated with table-mountains and massifs in the Venezuelan Guayana Shield.

Extent of occurrence: A minimum convex polygon enclosing all mapped occurrences of Venezuelan Tepui shrublands (Figure 4a) has an area of 196,148 km^2, well above the threshold for Vulnerable under criterion B1. The ecosystem is therefore listed under this criterion as Least Concern.

Area of occurrence: Superimposing a 10 km grid over the mapped polygons of Venezuelan Tepui shrublands (Figure 4b) indicates that 210 grid cells contain more than 1 km^2 of the ecosystem. Since this is well above the threshold for Vulnerable, they are listed as Least Concern under criterion B2.

Number of locations: There are ~35 locations of Venezuelan tepui shrublands, where human pressures are minimal. The status of the ecosystem is therefore Least Concern under criterion B3.

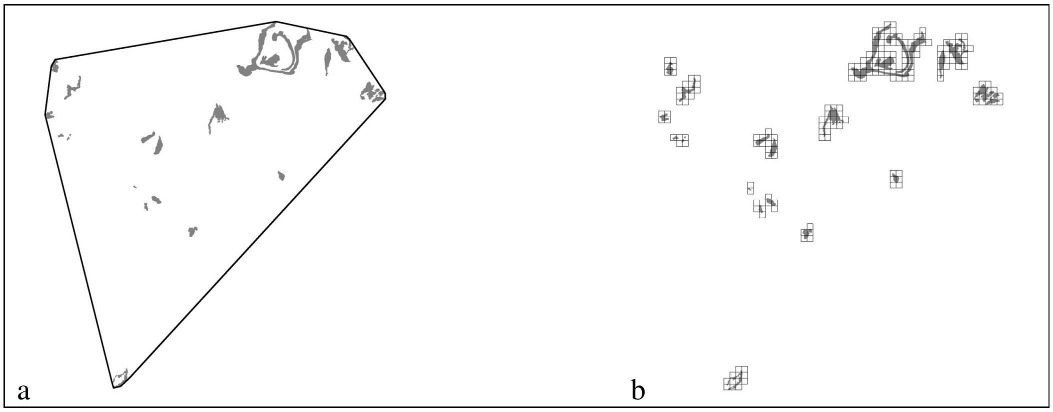

Figure S11. 4. Estimates of the geographic distribution of Tepui shrublands in Venezuela: a) extent of occurrence (EOO) considering one minimum convex polygon encompassing all known patches of the ecosystem, b) area of occupancy estimated by the number of occupied 10 x 10 km cells that include at least 1 km^2 of the ecosystem.

Criterion C

We have no evidence of major degradation or biotic disruption in ecological function. Global climate change could be the main process to threaten the ecosystem in the future, but the time scale of this process is uncertain and could be longer than the 50 year period considered by the criteria. Vega et al. (2012) project that the area of the climatic envelope currently occupied by the Tepui summits will

contract by 57-85% based on projected rises in mean annual temperature of 2-4C to year 2100, and that the number of Tepuis within this envelope will decline by 21-37%. However, it is unclear how this will affect the persistence of the characteristic native biota. The status of the ecosystem is therefore Not Evaluated under criterion C.

Criterion D

Climate change is the most salient threat to Tepui shrublands, as warming potentially makes the plateau summits unsuitable for persistence of the characteristic native biota. Changes in plant species composition and richness are potentially suitable for assessing disruption to biotic processes and interactions in the ecosystem, although the availability of data is limited.

Current decline: There are no records of any extinctions in the Tepui shrublands over the last 50 years. Although there are insufficient data to support more quantitative assessment of changes in plant species composition over this period, the lack of evidence of species loss or disturbances that might result in losses tentatively suggests a status of Least Concern under criterion D1.

Future decline: Several recent studies have projected the persistence of climatically suitable habitat for plant species of the Tepui shrublands under future climate change (Rull & Vegas-Vilarrubia 2006, Nogue et al. 2009a, Vega et al. 2012). An initial Altitudinal Range Displacement analysis of endemic flora suggests that temperatures will move outside the current range by the end of the twenty-first century for 9-27 of the 76 species assessed (9-35%), potentially threatening them with extinction unless they can persist under the new temperature regime (Rull & Vegas-Vilarrubia 2006). Further analysis combining species-area relationships with projected available areas derived from present-day temperatures at low altitude limits, suggests that 28-90% of the total Tepui flora, depending on the model used, will be outside its present-day temperature envelope by year 2100 (Nogue et al. 2009a). For individual Tepuis, the projected loss of climatically suitable potential habitat varies from 50 to 100%, while the potential loss of endemic species varies from 2 to 100% (Vega et al. 2012). These projections are based on limited survey data and spatial climatic data, as well as indirect modelling methods that exclude consideration of species life histories, potential lagged responses and micro-refugia, and will be revised as improved data become available to support use of more advanced methods (Nogue et al. 2009a).

The broad bounds of the estimates and limited modelling methods suggest substantial uncertainty in future projections. In addition, the projections are based on a time frame extending to year 2100, beyond that required for Red List assessment. Consequently, further work is needed to assess the functional significance of these projections and to assess changes over the relevant time frames, and the Tepui shrublands are currently assigned to Data Deficient status under criterion D2.

Historic decline: Limited historical data are available on the composition of the Tepui flora. However, Rull (2005) and Nogue et al. (2009b) carried out paleo-ecological studies of peat sediments from the summits of several Tepuis, and found that the vegetation had been stable over the past 4,300 - 6,000 years, with representation of pollen types consistent with contemporary vegetation and minor temporal variations in pollen abundance apparently reflecting local dynamics of meadow-forest ecotones. The long-term stability of Tepui vegetation composition suggests a status of Least Concern under criterion D3.

Criterion E

No quantitative assessment is available for this ecosystem, so under criterion E its status is Data Deficient.

References

Brewer-Carías C. 1978. La Vegetación del Mundo Perdido. Caracas: Fundación Eugenio Mendoza.

George U. 1988. Inseln in der Zeit. Venezuela-Expeditionen zu den letzten weissen Flecken der Erde. Hamburg: GEO.

Givnish TJ, McDiarmid RW, Buck WR. 1986. Fire adaptation in *Neblinaria celiae* (Theaceae), a high-elevation rosette shrub endemic to a wet equatorial tepui. Oecologia 70: 481–485.

Gorzula S. 1992. La herpetofauna del macizo del Chimantá. Chapter 15. Pp. 267-280. In: O. Huber (ed.) Chimantá, Escudo de Guayana, Venezuela. Oscar Todtmann Editores, Caracas.

Gorzula S. & Huber O. 1992. Consideraciones finales. Chapter 18. Pp. 325-330. In: O. Huber (ed.) Chimantá, Escudo de Guayana, Venezuela. Oscar Todtmann Editores, Caracas.

Hopper SD. 2009. OCBIL theory: towards an integrated understanding of the evolution, ecology and conservation of biodiversity on old, climatically buffered, infertile landscapes. Plant Soil 322: 49–86.

Huber O. 1987. Consideraciones sobre el concepto de Pantepui. Pantepui 2: 2-10.

Huber O. 1989. Shrublands of the Venezuelan Guayana. Pp. 271-285. In: LB Holm-Nielsen, IC Nielsen, H Balslev (eds.). Tropical forest: Botanical dynamics, speciation and diversity. Academic Press: London & N.Y.

Huber O. 1992. El macizo de Chimantá, Escudo de Guayana, Venezuela. Un ensayo ecológico tepuyano. Oscar Todtmann Editores: Caracas. 343 pp.

Huber O. 1995a. Vegetation. Pp. 97-160. In: PE Berry, BK Holst, K Yatskievych (eds.). Flora of the Venezuelan Guayana. Vol. 1: Introduction. Missouri Botanical Garden: St. Louis, Missouri & Timber Press: Portland, Oregon.

Huber O. 1995b. Mapa de vegetación de la Guayana Venezolana. Escala 1:2.000.000. CVG EDELCA, Missouri Botanical Garden. Ediciones Tamandúa: Caracas.

Huber O. 1995c. Conservation of the Venezuelan Guayana. Pp. 193-218. En: PE Berry, BK Holst, K Yatskievych (eds.). Flora of the Venezuelan Guayana. Vol. 1: Introduction. Missouri Botanical Garden: St. Louis, Missouri & Timber Press: Portland, Oregon.

Huber O, Alarcón C. 1988. Mapa de vegetación de Venezuela 1:2.000.000. The Nature Conservancy, MARNR. Oscar Todtmann Editores: Caracas.

Huber O, Oliveira-Miranda MA. 2010. Ambientes terrestres. Pp: 29-89. In: JP Rodríguez, F Rojas-Suárez, D Giraldo Hernández (eds.). Libro Rojo de los Ecosistemas Terrestres de Venezuela. Provita, Shell Venezuela, Lenovo (Venezuela). Caracas: Venezuela.

Maguire B. 1970. On the flora of the Guayana Highland. Biotropica 2: 85-100.

MARN. 2000. Primer informe de Venezuela sobre Diversidad Biológica. Ministerio del Ambiente y de los Recursos Naturales: Caracas.

Molina C, Salcedo M. 2009. Aves del Parque Nacional Canaima. Pages 133-149 In: J. C. Señaris, D. Lew and C. Lasso, editors. *Biodiversidad del Parque Nacional Canaima: bases técnicas para la conservación de la Guayana venezolana*. Fundación La Salle de Ciencias Naturales and The Nature Conservancy, Caracas.

Nogué S, Rull V, Vegas-Vilarrúbia, T. 2009a. Modeling biodiversity loss by global warming on Pantepui, northern South America: projected upward migration and potential habitat loss. Climate Change 96: 77–85.

Nogué S, Rull V, Montoya E, Huber O, Vegas-Vilarrúbia T. 2009b. Paleoecology of the Guayana Highlands (northern South America): Holocene pollen record from the Eruoda-tepui, in the Chimantá massif. Palaeogeography, Palaeoclimatology, Palaeoecology 281: 165–173.

Oliveira-Miranda MA, Huber O, Rodríguez JP, Rojas-Suárez F, De Oliveira-Miranda R, Hernández-Montilla M, Zambrano-Martínez S, Giraldo-Hernández D. 2010. Riesgo de eliminación de los ecosistemas terrestres de Venezuela. Pp: 106-236. In: JP Rodríguez, F Rojas-Suárez, D Giraldo-Hernández (eds.). Libro Rojo de los Ecosistemas Terrestres de Venezuela. Provita, Shell Venezuela, Lenovo (Venezuela). Caracas: Venezuela.

Riina R, Huber O. 2003. Ecosistemas exclusivos de la Guayana. Pp. 828-861. In: M Aguilera, A Azócar, E González Jiménez (eds.). Biodiversidad de Venezuela. Tomo II. Fundación Polar,

Ministerio de Ciencia y Tecnología, Fondo Nacional para la Ciencia, Tecnología e Innovación (FONACIT). Editorial ExLibris: Caracas.

Rull V. 2005. Vegetation and environmental constancy in the Neotropical Guayana Highlands during the last 6000 years? Review of palaeobotany and palynology 135: 205 -222.

Rull V, Vegas-Vilarrúbia T. 2006. Unexpected biodiversity loss under global warming in the neotropical Guayana Highlands: a preliminary appraisal. Global Change Biology 12: 1-9.

Rull V, Vegas-Vilarrúbia T, Nogué S. 2005. Cambio climático y diversidad de la flora vascular en las montañas tabulares de Guayana. Orsis 20: 61-71.

Rull V, Vegas-Vilarrubia T, Nogué S, Huber O. 2009. Conservation of the unique Neotropical vascular flora of the Guayana Highlands in the face of global warming. Conservation Biology 23(5): 1323-1327.

Señaris J C, Lew D, Lasso C, editors. 2009 Biodiversidad del Parque Nacional Canaima: bases técnicas para la conservación de la Guayana venezolana. Fundación La Salle de Ciencias Naturales and The Nature Conservancy, Caracas. 256 pp.

Vegas-Vilarrúbia T, Sandra Nogué S, Rull V. 2012. Global warming, habitat shifts and potential refugia for biodiversity conservation in the neotropical Guayana Highlands. Biological Conservation 152: 159–168.

Zinck JA, Huber O, editors. 2011. Peatlands of the western Guayana Highlands, Venezuela: properties and paleogeographic siginficance of peats. Springer-Verlag, Berlin. 295 pp.

12 GRANITE GRAVEL FIELDS & SAND PLAINS, NEW ZEALAND

Contributed by Robert Holdaway, Susan Wiser, Sarah Richardson & Elise Arnst, Landcare Research, P.O. Box 40, Lincoln 7640, New Zealand

CLASSIFICATION

National: Granite gravel fields and granite sand plains are indistinguishable from each other; both are classified as Naturally Uncommon Ecosystems in New Zealand (Williams et al. 2007; Richardson et al. 2012).
International: No international example of this ecosystem type is yet known
IUCN Habitats Classification Scheme (Version 3.0): 4. Grassland / 4.4 Temperate

ECOSYSTEM DESCRIPTION

Distribution

Granite is widespread bedrock in western South Island, New Zealand, forming the dominant bedrock in two alpine regions; Fiordland and North-West Nelson (Rattenbury et a. 1998, Turnbull et al. 2010). These two regions, once contiguous, share a common origin as part of the Fiordland Terrane, formed during the Cretaceous, 120 million years ago (Coates 2002, Reay 2003). Lateral movement of the Pacific and Australian plates along New Zealand's Alpine Fault during the last 25 million years has separated granites within this Terrane. Typical granite landforms throughout this belt are tors, screes and boulders. Nationally, only a few examples are known of extensive dunes or plains of fine sands and small-grained gravels in high alpine situations, with the most significant areas being located on the Lookout Range in NW Nelson, and Mt Titiroa in eastern Fiordland (Figure 1). Worldwide, there are no other known examples of this ecosystem type, suggesting that it may be an ecosystem that is endemic to New Zealand.

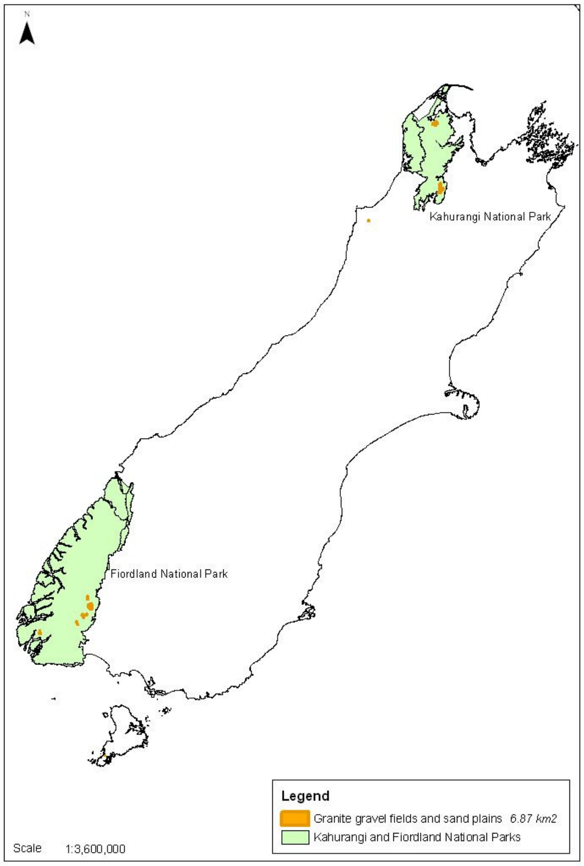

Figure S12. 1. Map of the South Island of New Zealand showing the current known occurrences of the granite gravel field and sand plain ecosystem.

Abiotic environment

Granite gravel fields and sand plains are characterized by extensive dunes or plains of fine sands and small-grained gravels (Figure 2) (Richardson et al. 2012). They occur on very exposed sites above treeline (1100-1600 m, mean annual temperate 3.4°C-4.7°C), where erosion-prone granite bedrock is close to the surface. Sites typically comprise approximately 80% bare ground and bedrock (Richardson et al. 2012, Figure 2). Due to their chemistry and structure, these granites crumble and rot to form angular gravels and ultimately sands. Relative to other alpine granite ranges in New Zealand, rainfall is relatively low (2100-2400 mm) and the terrain is flatter (mean slope of 12°, range 1°-32°), and this may encourage the production of low dunes. While many features of these ecosystems are shared with other alpine granite surfaces, such as low soil nutrients high light and extreme weathering, others such as the mobile substrate and excessive drainage, are unique.

The NW Nelson granites were formed at a shallower depth and have experienced substantially less physical and chemical weathering relative to the granites in Fiordland (Reay 2003). Soils are very poorly developed, well-drained and excessively nutrient-poor, with low nutrient concentrations. For example, mean total phosphorus (P) was 69.5 mg kg^{-1} and 55.7 mg kg^{-1}, and total nitrogen (N) was 0.04%and 0.03%, at the Lookout Range and Mt Titiroa, respectively. The proportion of total P as inorganic P was higher on Mt Titiroa (53%) than on the Lookout Range (33%; Table S1), reflecting steeper topography, erosion and input of mineral P.

Characteristic native biota

Vegetation is sparse and species poor. For example, a sample of 90 plots (each 100 m^2) from the Nelson and Fiordland locations identified a total of only 86 vascular plant species (Richardson et al. 2012). The native biota is characterised by low shrubs (e.g., *Dracophyllum pronum*), grasses (e.g., *Poa colensoi*), cushion plants (e.g., *Chionohebe pulvinaris*) and small herbs (e.g., *Anisotome imbricata* var. *prostrata* and *Notothlaspi australe*) (Richardson et al. 2012). Vegetation height rarely exceeds 30 cm, with many plants being prostrate and woven among the granite particles, forming small mounds or islands (Figure 2). Few native invertebrates or animals are known to be strongly associated with this characteristic vegetation. However, the wider mosaic of granite gravel fields and sand plains, screefield, alpine tussock grassland and herbfield support a range of invertebrate species including the rare carabid beetle, *Mecodema integratum*, the range-restricted giant scree weta *Deinacrida connectens*, and the Mt Titiroa sand and gravel fields support a recently described moth *Hierodoris extensilis*, known only from there and nearby Mt Burns (Hoare et al. 2012).

Key processes and interactions

The weathering and erosion of the granite bedrock is the key abiotic process governing the production of mobile, highly drained and nutrient poor substrate. It is thought that the relatively gentle topography, relatively low rainfall and high wind exposure promotes the formation of extensive dune systems. The vegetation is adapted to cope with low soil nutrients and highly mobile substrate. Facilitation may be important for plant establishment, with the establishment of pioneer vegetation (e.g. *Dracophyllum pronum* or *Chionohebe pulvinaris*) creating locally stabilised "islands" in which other species may become established. Coupled with on-going erosion processes, these processes create the open mosaic of mounded vegetation clumps that is characteristic of this ecosystem (Figure 2). The loose gravels may provide burrowing sites for insects, but this has not been explicitly studied.

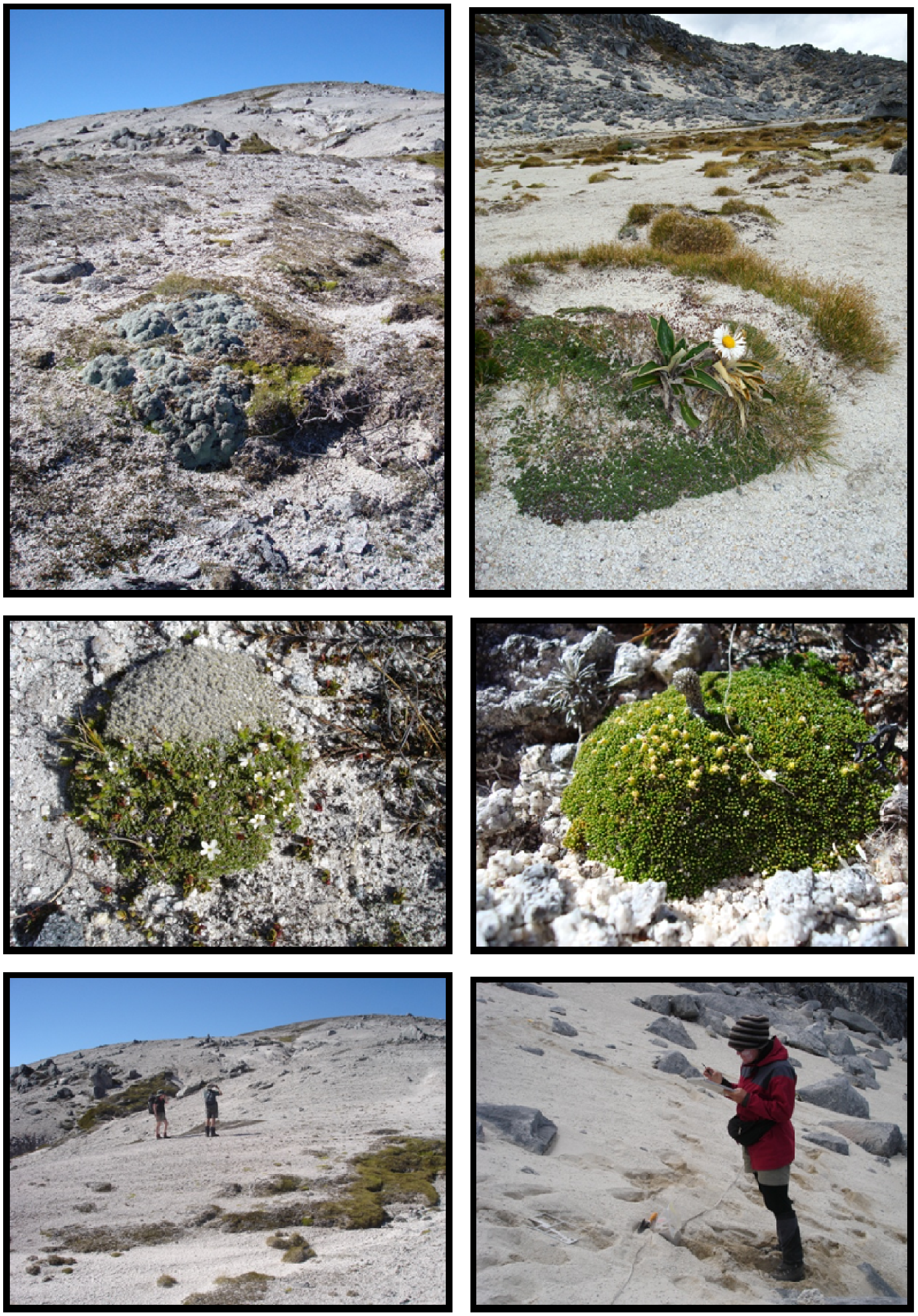

Figure S12. 2. Examples of the granite gravel fields and sand plains ecosystem from the Lookout Range, NW Nelson (left) and Mt Titiroa, Fiordland (right) showing landforms and characteristic plant associations (photos provided by Sarah Richardson & Susan Wiser).

Threatening processes

We know of very few, if any, processes that significantly threaten the characteristic biota of the granite gravel fields and sand plains ecosystem. This ecosystem is located entirely within conservation estates (99.8% is contained within Kahurangi and Fiordland National Parks), and due to its geographic isolation it receives little anthropogenic pressure. Invasive species are uncommon (e.g. only one of the 86 plant species found in 180 plots was exotic). Exotic mammals are likely to be transient and live predominantly in the surrounding mosaic of grassland, shrubland and forest. Mammalian predation may threaten the invertebrate fauna and climate change may pose an increasing threat in the future, but these are not thought to be significant threats to the maintenance of this ecosystems characteristic biota within the next 20 years.

Because of the lack of threatening processes and the apparent stability of the biotic and abiotic components of this ecosystem over the assessment time frames, we do not define thresholds of collapse.

ASSESSMENT

Summary

Criterion	A	B	C	D	E	overall
subcriterion 1	LC	LC	LC	LC	DD	LC
subcriterion 2	LC	LC	LC	DD		
subcriterion 3	LC	LC	LC	LC		

Criterion A

<u>Current decline</u>: Current spatial extent of granite gravel fields and sand plains is estimated as 687 ha (Figure 1). All examples of this ecosystem type are located in legally protected conservation land, with 99.8% located within two National Parks; Fiordland, gazetted in 1952 and Kahurangi, gazetted it 1996 (Figure 1). There is no evidence to suggest declines in the extent of granite gravel fields and sand plains over the last 50 years. The status under criterion A1 is therefore Least Concern.

<u>Future decline</u>: All areas of this ecosystem are located within legally protected conservation land. Although mining is permitted on conservation land in some locations, there are no known minerals of economic value beneath this ecosystem. There is no reason to expect future declines in extent over the next 50 years. The status under criterion A2 is therefore Least Concern.

<u>Historical decline</u>: The isolated alpine environment of granite gravel fields and sand plains has insulated them from anthropogenic land clearance and burning. There is no evidence of decline in extent since 1750, so it is therefore listed as Least Concern under criterion A3

Criterion B

<u>Extent of occurrence</u>: Extent of occurrence is 61,727 km^2, making this ecosystem Least Concern under criterion B1.

<u>Area of occupancy</u>: The number of 10 km × 10km grid squares occupied by granite gravel fields and sand plains is 15, with 7 containing >1% of the ecosystem type. This initially places the ecosystem within the threshold to be Endangered. However, there are no observed or inferred continuing declines in spatial extent, abiotic environmental or biotic interactions, no observed or inferred threatening processes, and the ecosystem exists at more than five locations. Granite gravel fields and sand plains are therefore listed as Least Concern under criterion B2.

Locations: Granite gravel fields and sand plains occur at nine distinct locations (Figure 1). There are no major threatening processes operating at these locations that are capable of causing collapse or making the ecosystem critically endangered within a short time period. This ecosystem is therefore listed as Least Concern under criterion B3.

Criterion C

Current decline: A recent survey of the granite gravel fields and sand plains characterised the abiotic environment in which these ecosystems occur (Richardson et al. 2012). There is no evidence to suggest that this environment has changed over the past 50 years and the surrounding mosaic includes extensive areas of virgin old-growth forest. Granite gravel fields and sand plains are therefore Least Concern under criterion C1.

Future decline: Although internationally the temperature increase due to climate change is expected to be stronger in the alpine zone, geology, fertility, exposure and substrate mobility, rather than temperature, are the main abiotic factors affecting the characteristic biota in this ecosystem. These features are expected to remain stable over into the future. Granite gravel fields and sand plains are therefore listed as Least Concern under criterion C2.

Historical decline: New Zealand's alpine ecosystems have a high degree of natural climate variability, and the long term trend has been relatively stable since 1750 (Duncan et al. 2010). As there is no evidence to suggest that there have been any major long-term changes to the ecosystems abiotic environment and its ability to sustain its characteristic biota, it is listed as Least Concern under criterion C3.

Criterion D

Current decline: Invasive plants are largely absent from these ecosystems, due primarily to their geographic isolation and harsh environment (Richardson et al. 2012). Invasive mammals are likely to be affecting insect and bird abundance across the entire extent, however, these species are not key components of the ecosystem identity, appear not to play critical roles in ecosystem function, and changes in their abundance are unlikely to be symptoms of future ecosystem collapse. Granite gravel fields and sand plains are therefore listed as Least Concern under criterion D1.

Future decline: Although there are no reasons to expect future decline in the biotic interactions that sustain the native biota, other than apparently minor disruptions caused by invasive mammals, the basis for projection is highly uncertain. Granite gravel fields and sand plains are therefore listed as Data Deficient under criterion D2.

Historical decline: There is no evidence to suggest historical decline in biotic processes and interactions that sustain the characteristic biota of this ecosystem. Although there are likely to have been a number of now-extinct bird species present in these ecosystems (Worthy and Holdaway 2002), they are unlikely to have played critical roles in the maintenance of this ecosystem. Granite gravel fields and sand plains are listed as Least Concern under criterion D2.

Criterion E

No quantitative modelling of risk of collapse has been undertaken. Granite gravel fields and sand plains is therefore Data Deficient under criterion E.

REFERENCES

Coates G (2002) The rise and fall of the Southern Alps. Christchurch: Canterbury University Press.

Duncan R, Fenwick P, Palmer J, McGlone M, Turney C (2010) Non-uniform interhemispheric temperature trends over the past 550 years. Climate Dynamics 35: 1429-1438.

Rattenbury MS, Cooper RA, Johnston MR (1998) Geology of the Nelson area: scale 1:250,000. Lower Hutt: Institute of Geological & Nuclear Sciences Limited. Institute of Geological & Nuclear Sciences 1:250,000 geological map 9. 67 p. + 1 folded map

Reay T. 2003. Geology. In The natural history of southern New Zealand. Eds. Darby J, Fordyce RE, Mark A, Probert K, Townsend C. pp. 1–16. Otago University Press, Dunedin, New Zealand.

Richardson SJ, Williams PA, Mason NWH, Buxton RP, Courtney SP, et al. (2012) Rare species drive local trait diversity in two geographically disjunct examples of a naturally rare alpine ecosystem in New Zealand. Journal of Vegetation Science: n/a-n/a. DOI: 10.1111/j.1654-1103.2012.01396.x

Turnbull IM, Allibone AH, Jongens R (2010) Geology of the Fiordland area: scale 1:250,000. Lower Hutt: GNS Science. Institute of Geological & Nuclear Sciences 1:250,000 geological map 17. 97 p. + 1 folded map

Williams PA, Wiser S, Clarkson B, Stanley MC (2007) New Zealand's historically rare terrestrial ecosystems set in a physical and physiognomic framework. New Zealand Journal Of Ecology 31: 119-128.

Worthy TH, Holdaway RN (2002) The Lost World of the Moa. Prehistoric Life of New Zealand. Bloomington: Indiana University Press.

13 CAPE FLATS SAND FYNBOS, SOUTH AFRICA

contributed by David Keith, Australian Wetlands, River and Landscapes Centre, University of New South Wales, and NSW Office of Environment and Heritage.

CLASSIFICATION

National: Cape Flats Sand Fybos (FFd 5) is one of eleven vegetation units within the Sand Fynbos vegetation group (Rebelo et al. 2006). Eight other fynbos vegetation groups are recognised on the basis of substrate. Fynbos is one of three major vegetation complexes, with renosterveld and strandveld, that make up the shrublands of the Fynbos Biome (Rebelo et al. 2006).
IUCN Habitats Classification Scheme (Version 3.0): 3. Shrubland / 3.4 Temperate
Key references: Rebelo et al. 2006.

ECOSYSTEM DESCRIPTION

Characteristic native biota

Cape Flats Sand Fynbos is a species-rich, dense, moderately tall shrubland with scattered emergent shrubs (Rebelo et al. 2006). This ecosystem is species rich in low shrubs compared with other fynbos units on sand flats. The dominant plant families are Ericaceae, Proteaceae, Asteraceae and Restionaceae (Fig. 1). Tall shrubs include *Metalasia densa, Morella crdifolia, M. serrata, Passerina cormbosa, Protea burchellii, P. repens, Psoralea pinnata, Pterocelatrus tricuspidatus, Rhus lucida* and *Wiborgia obcordata*. Low shrubs include *Diastella proteoides, Diosma hisuta, Erica lasciva, E. muscosa, Phylica cephalantha, Senecio halimifolius, Serruria glomerata, Stoebe plumosa* and a large number of less common species. Herbs include *Berkheya rigida, Conyza pinnatifida, Edmondia sesamoides, Helichrysum tinctum, Indigofera procumbens, Knowtonia vesicatoria* the succulent *Carpobrotus acinaciformis* and the climber *Dipogon lignosus*. Geophytes include *Watsonia meriana* with *Aristea dichotomoa, Geoissorhiza tenella, Othonna heterohylla, Pelargongium longifolium, Wachendorfia paniculata* and *Zantdeschia aethiopica*. Graminoids include *Cynodon dactylon, Ehrharta villosa* var. *villosa, Elgia tectorum, Restio quinquefarius, Sporobolis virginicus, Thamnochortus erectus, Willdenowia incurvata* and numerous less common species (Rebelo et al. 2006).

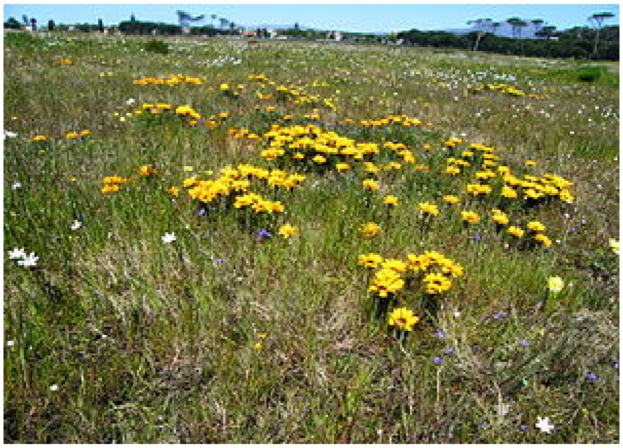

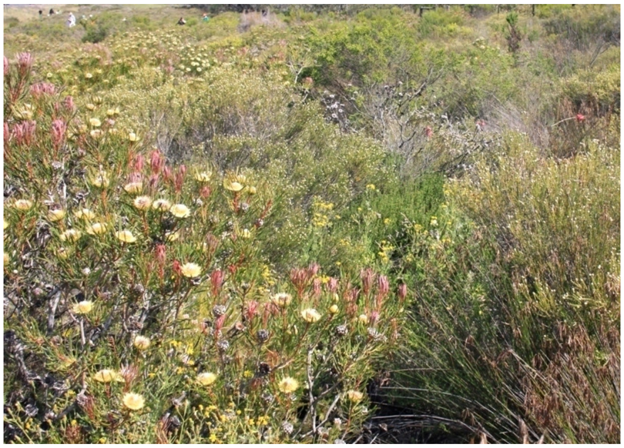

Figure S13. 1. Remnant of Cape Flats Sand Fynbos with *Gazania pectinata* at Rondebosch Common (left, photo: A. Massyn) and with *Leucodendron, Erica* and *Resto* species (right, photo: S. Molteno).

Plant taxa essentially restricted to Cape Flats Sand Fynbos include low shrubs *Erica margaritacea, Aspalathus variegata, Athanasia capitata, Cliffortia ericifolia, Erica pyramidalis, E. turgida, E. verticillata* (Fig. 2), *Leucadendron levisanus, Liparia graminifolia, Serruria aemula, S. foeniculacea,*

and *S. furcellata;* the succulent *Lampranthus stenus;* the geophytic *Ixia versicolor;* and graminoids *Tetraria variabilis* and *Trianoptiles solitaria* (Rebelo et al. 2006).

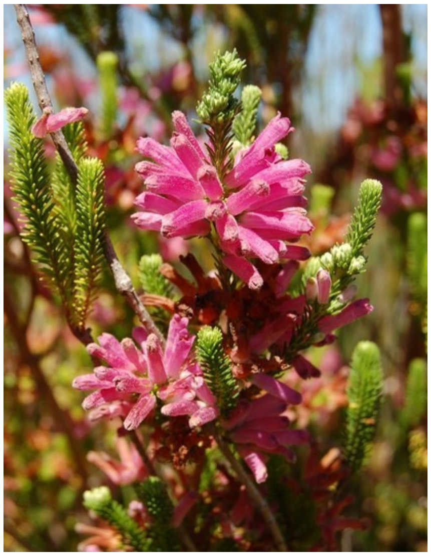

Figure S13. 2. *Erica verticellata*, endemic to Cape Flats Sand Fynbos and now listed as extinct in the wild. Photo: A. Shawka.

Dures & Cumming (2010) recorded 79 bird taxa within Cape Flats Sand Fynbos, the most common of which were Cape White Eye (*Zosterops virens*), Karoo Prinia (*Prinia maculosa*), Cape Turtle Dove (*Streptopelia capicola*) and Pied Crow (*Corvus albus*).

Abiotic environment

Cape Flats Sand Fynbos is an edaphically determined species assemblage restricted to Tertiary acid, deep grey regic sands at low elevations (20 - 200 m) on flat to undulating terrain (Rebelo et al. 2006). The sands are heavily leached and nutrient deficient podzolic soils of the Lamotte form. The climate is dry temperate, receiving a mean annual preciptation of 580 - 980 mm, with strong winter dominance, including precipitation as mists (Rebeo et al. 2006). On average, less than 10 mm of rain falls per month between November and March. Mean annual temperatures are 16.2°C, with mean summer daily maximum of 27°C in February and mean winter daily minimum of 7°C in July (Rebelo et al. 2006). Frosts occur on about three days per year.

Distribution

Cape Flats Sand Fynbos is restricted to the Western Cape Province of South Africa (latitude 33° 55' S, longitude 18° 22' E), almost entirely within the limits of the City of Cape Town (Fig. 3, Rebelo et al. 2006). Its distribution extends from Blouberg and Koeberg Hills west of the Tygerberg Hills to Lakeside and Pelican Park in the south near False Bay, from Bellville and Durbanville to Klapmuts and Joostenberg Hill in the east, and to the southwest of the Bottelary Hills to Macassar and Firgrove in the south.

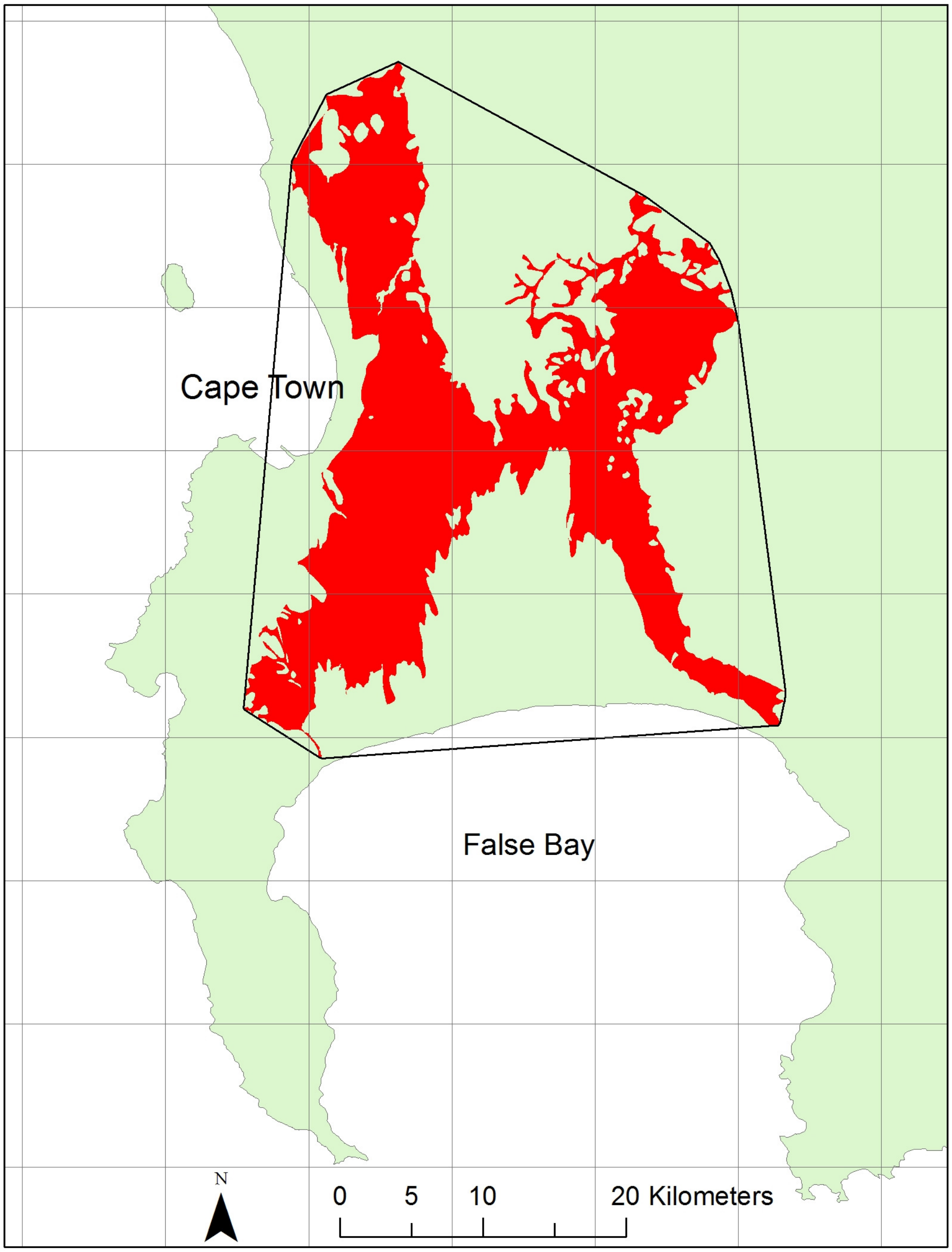

Figure S13. 3. Geographic distribution of Cape Flats Sand Fynbos near the Cape of Good Hope, South Africa. Map shows the historical distribution, projected from occurrences remnants and distribution of soil types (source: Mucina & Rutherford 2006), within minimum convex polygon and 10 km grid.

Key processes and interactions

Soil nutrient deficiency determines much of the functional character of this and other fynbos ecosystems (Stock & Allsopp 1992). Key characteristics of fynbos vegetation that are linked to nutritional poverty include sclerophyllous foliage, low nutrient concentrations in foliage and reproductive tissues, high concentrations of fibre and phenolic compounds in foliage, specialised mechanisms for nutrient uptake such as cluster roots and mycorrhiza, high root:shoot mass ratios, high rates of nitrogen and phosphorus withdrawal from senescent leaf tissues, slow rates of leaf turnover litter production and decomposition, low rates of herbivory and high rates of nectivory among birds and

mammals (Low 1983; Rebelo et al. 1984; Mitchell et al. 1986; Stock & Allsopp 1992; Lambers et al. 2003).

The high fibre and phenolic concentration, together with a climate characterised by seasonal drought predisposes fynbos vegetation to recurring fires, generally every 5 - 50 years. Fires have a profound effect on the evolution of plant life histories and on ecosystem dynamics (Bond & van Wilgen 1996). Plant life history traits that are well respresented in the fynbos flora include resprouting and obligate seeding, serotinous seedbanks, myrmecochory, heat- and smoke-stimulated seeed germination, pyrogenic flower production and post-fire seedling recruitment (Le Maitre & Midgley 1992). Variations in fire frequency, intensity and season drive changes in community composition and structure of fynbos through successive fire intervals, as different plant species are favoured by contrasting fire regimes (Bond et al. 1984; Bond & van Wilgen 1996).

Threatening processes

Habitat loss, through conversion of native vegetation to urban land use associated with the expansion of Cape Town, now with a population of almost 4 million people, is a major threat to Cape Flats Sand Fynbos (Wood et al. 1994). Eight locally endemic plant species are presumed extinct as a consequence of extensive vegetation clearing throughout the distribution of the ecosystem. Other taxa including birds, reptiles, amphibians and butterflies show similar patterns of threat (Rebelo 1992). Legislative protection of Cape Flats Sand Fynbos as a Critically Endangered ecological community (Department of Environmental Affairs 2011) is likely to have slowed clearing rates, however much of the remnant vegetation will undergo continuing loss of its biodiversity due to lagged effects of fragmentation, changed fire regimes, invasion of exotics, stochastic processes and Allee effects (e.g. Dures & Cumming 2010). Extraction of ground water for urban consumption is a related threat that is likely exacerbate fragmentation impacts on urban remnants of the ecosystem (Rebelo et al. 2006).

Invasion of alien plants is the most severe threat to the remaining area of Cape Flats Sand Fynbos (Rebelo et al. 2006). These include a range of annual herbs and grasses (Vlok 1988), but the most problematic species are woody plants, including *Pinus halepensis, P. pinaster, P. radiata, Acacia saligna, A. cyclops, A. longifolia, A. mearnsii, Hakea drupacea, H. gibbosa* and *H. sericea*. These species reduce native plant diversity through competition (Richardson & Cowling 1992) and also reduce diversity of avifauna through habitat modification (Dures & Cumming 2010). Some of the displacement of native vegetation by weeds is due to nutrient enrichment associated with runoff from urban development, as well as prior agricultural land use. Additional nutirents give some exotic plants a competitive advantage over native species, and may accelerate mortality in the latter due to toxicity effects (Stock & Allsopp 1992).

Alterations to fire regimes pose a potenital threat to persistence of biota within Cape Flats Sand Fynbos. Serotinous obligate seedling plant species are especially susceptible to elimination by high fire frequencies that may result from arson and accidental ignitions in urban landscapes (Bond & van Wilgen 1996). Conversely, the same species may also be eliminated by long intervals between fires, as standing plants sensesce after 50 years of age and depend on fires for seedling regeneration. Some remnant patches of the ecosysyem may be expected to experience reduced fire frequency due to the lack of vegetation connectivity, which inhibits fire spread. Seedling regeneration is also highly sensitive to fire season due to the seasonal variability in available climatic moisture, which is vital to seedling emergence and establishment (Le Maitre & Midgley 1992).

Climate change is a third major threatening process potentially affecting Cape Flats Sand Fynbos, with mean annual temperatures projected to increase by roughly 1.8°C by 2050. Modelled projections of species distributions suggest an eastward shift in bioclimatically suitable habitat for many fynbos plant taxa, with that much the Cape Flats will no longer be suitable for fynbos by 2050 (Midgley et al. 2003; 2006). Overall biome-level distribution models projected that bioclimatically suitable habitat for fynbos would contract by 51-65%, with 61% of fynbos habitat below 300 m elevation projected to be lost by

2050, including much of the Cape Flats (Midgley et al. 2003). Likely secondary effects of warming include changes to precipitation and fire regimes.

Ecosystem collapse

For assessment of criteria A and B, collapse was assumed to occur when the mapped distribution of Cape Flats fynbos declines to zero, signalling the replacement of upland swamp by developed areas. The relative abundance of exotic plant species would be suitable for assessing disruption of biotic processes and interactions under criterion D if sufficient data were available.

ASSESSMENT

Summary

Criterion	A	B	C	D	E	Overall
subcriterion 1	VU	CR	DD	DD	DD	CR
subcriterion 2	EN	EN	DD	DD		
subcriterion 3	EN	VU	DD	DD		

Criterion A

Current decline: Limited data are available for estimating the reduction in distribution of Cape Flats Sand Fynbos over the past 50 years. However, Rebelo et al. (2011) estimate the trajectories of change in extent of native vegetation within the City of Cape Town, in which Cape Flats Sand Fynbos was the most widespread vegetation type (22% of the area). The extent of proclaimed reserves and unproclaimed vegetation, respectively, was approximately 132,000 ha and 3,000 ha in 1955, and 57,000 ha and 32,000 ha in 2005 (Rebelo et al. 2011, Fig. 3). The extent of native vegetation within the City area therefore declined from 135,000 ha to 89,000 ha. These estimates include three major protected areas, Table Mountain the Cape Peninsula and the Hottentots Holland mountains (part), each of which contains rocky, mostly steep landscapes and a negligible area of Cape Flats Sand Fynbos, as mapped by Mucina and Rutherford (2006). Subtracting the combined area of these reserves (37,000 ha), gives a change in the remaining vegetated area from 98,000 ha to 52,000 ha, a 47% decline over the past 50 years. Assuming that the decline in distribution of Cape Flats Sand Fynbos was proportionate to that in other vegetation types outside protected areas, suggests Vulnerable status under criterion A1.

Future decline: Midgley et al. (2003) modelled bioclimatically suitable habitat for the Fynbos Biome using five bioclimatic variables: mean minimum temperature of the coldest month; annual sum of daily temperatures exceeding 18 °C; annual potential evaporation (PE); the number of winter days on which soil moisture is above a critical level for plant growth in winter; and in siummer. They then projected the model using a future climate change scenario, assuming atmospheric CO2 would reach 550 ppm by 2050, generated by the general circulation models CSM and HadCM2 (including two sulphate amelioration scenarios), downscaled and interpolated for South Africa (Hewitson, 1999; Schulze & Perks, 1999). They found an overall contraction to the south, with total bioclimatically suitable area of the Fynbos Biome declining by 51-65% between the present and 2050. Spatially explicit projections show the eastern portion (50-60%) of the distribution of Cape Flats Sand Fynbos becoming climatically unsuitable by 2050 (Midgley et al. 2003, Fig. 1). In addition, the area climatically suitable for fynbos was estimated to decline by approximately 60% within the altitudinal band occupied by this ecosystem (0 - 300 m ASL). These esimates suggest a future decline in distribution of 50-65%, and hence Endangered status under criterion A2.

Historic decline: The principle sources of spatial data on the impact of clearing include a map showing the reconstructed historic distributions of vegatation types (Mucina & Rutherford 2006) and a map of the extant native vegetation based on 2005 aerial photography ground truthed in 2007-2008 (Fig. 1 in Rebelo et al. 2011). These suggest that the extent of Cape Flats Sand Fynbos has declined from 54,448

ha to 8,467 ha, a decline of 84.4% (Stipinovich & Holmes 2009). It is assumed that almost all of this decline occurred since 1750, although it had been occupied by Europeans a centuryt earlier (Rebelo et al. 2011). The status of the ecosystem is therefore Endangered under criterion A3.

Criterion B

Extent of occurrence: A minimum convex polygon enclosing all mapped occurrences of Cape Flats Sand Fynbos has an area of 1,427 km^2 (Fig. 3). There is a continuing decline in distribution of the ecosystem, with loss of unproclaimed vegetation accelerating from the 1990s (Rebelo et al. 2011). The remaining distribution is highly fragmented, with many of the remnants ungoing invasions from exotic plants and associated declines in diversity of avifauna. This, together with thee large number of Red Listed plant taxa (92), including 4 extinct species (Raimondo et al. 2009; Rebelo et al. 2011), suggest continuing declines in ecosystem diversity and degradation of biotic processes, as well as future threats. The status of the ecosystem is therefore Critically Endangered under criterion B1a,b.

Area of occurrence: Superimposing a 10 km grid over the mapped polygons of Cape Flats Sand Fynbos indicates that no more than 12 grid cells contain more than 1 km^2 of the ecosystem (Fig. 3). This, together with evidence of declines in distribution and function described under 'Extent of occurrence' supports Endangered status under criterion B2.

Number of locations: The most severe threat to the ecosystem is habitat destruction associated with urban development. Occurrences of ecosystem that are currently within proclaimed reserves are protected from this threat. However, these stands are threatened by invasion of exotic plants. As the entire distribution of the ecosystem is within the City of Cape Town, the unproclaimed remnant vegetation is subject to the same development pressures, regulatory regimes and planning authoriity. Given that all remaining vegetation occurs within an urban landscape, all is exposed to similar pressures from invasive exotics. The distribution is therefore interpreted as two semi-independent locations; one outside proteced areas (threatened by habitat destruction and invasive plants) and one within protected areas (threatened by invasive plants, but not habitat destruction). Given the severe and immediate nature of the threats (Rebelo et al. 2011), the ecosystem is prone to the effects of human activity or stochastic events such that it is capable of collapse or becoming Critically Endangered within a very short time period. The status of the ecosystem is therefore Vulnerable under criterion B3.

Criterion C

There are currently no time series data available enabling an assessment of the rate of degradation in the abiotic environment of Cape Flats Sand Fynbos. The status of the ecosystem is therefore Data Deficient under criterion C.

Criterion D

There is evidence of ongoing decline in ecosystem function through disruption of biotic processes and interactions. Remnant patches of the ecosystem are fragmented and undergoing loss of plant species diversity, as indicated by the large number (92) of species listed as threatened on the South African Red List (Raimondo et al. 2009; Rebelo et al. 2011). In addition, remnant patches are becoming increasng degraded by invasion of exotic plants and there is an associated loss of diversity in avifauna (Dures & Cumming 2010). At present, however, there are insufficient data to quanitfy these trends. The status of Cape Flats Sand Fynbos is therefore Data Deficient under criterion D.

Criterion E

No quantitative assessment is available for this ecosystem, so under criterion E its status is Data Deficient.

REFERENCES

Bond WJ, van Wilgen BW (1996) Fire and plants. Chapman and Hall, London.

Bond WJ, Vlok J,& Vivers M (1984) Variation in seedling recruitment of Cape Proteaceae after fire. Journal of Ecology 72, 209-221.

Department of Environmental Affairs (2011) National Environmental Management: Biodiversity Act (1 0/2004): National list or ecosystems that are threatened and in need ot protection. National Gazette No 34809 of 9 December 2011, Volume 558. Government of South Africa, Pretoria.

Dures SG, Cumming GS (2010). The confounding influence of homogenising invasive species in a globally endangered and largely urban biome: Does habitat quality dominate avian biodiversity? Biological Conservation 143, 768–777.

Lambers H, Cramer HD, Shane MW, Wouterlood M, Poot P, Veneklass EJ (2003) Structure and functioning of cluster roots and plant responses to phosphate deficiency. Plant Soil 248, ix-xix.

Le Maitre DC, Midgley JJ (1992). Plant reproductive ecology. Pp 135-174 in R. M. Cowling (ed.) *The ecology of fynbos: nutrients, fire and diversity*. Oxford University Press, Cape Town.

Low AB (1983) Phytomass and major nutrient pools in an 11-year old post-fire coastal fynbos community. South African Journal of Botany 2, 98-104.

Midgley GF, Hannah L, Millar D, Thuiller W, Booth D (2003) Developing regional and species-level assessments of climate change impacts on biodiversity in the Cape Floristic Region. Biological Conservation 112, 87–97.

Midgley GF, Hughes GO, Thuiller W, Rebelo AG (2006) Migration rate limitations on climate change-induced range shifts in Cape Proteaceae. Diversity and Distributions 12, 555–562.

Michell DT, Coley PGF, Webb S, Allsopp N (1986) Litterfall and decomposition processes in the coastal fynbos vegetation, South-western Cape, South Africa. Journal of Ecology 74, 977-993.

Mucina L, Rutherford MC (eds.) *The vegetation of South Africa, Lesotho and Swaziland*. Strelitzia 19. South African National Biodiversity Institue, Pretoria.

Raimondo D,Von Staden L, Foden W,Victor JE, Helme NA, Turner RC, Kamundi DA, Manyama PA (2009) Red List of South African Plants. Strelitzia 25.

Rebelo AG (1987) A preliminary synthesis of pollination biology in the Cape flora. South African National Science Progress Report 141, 1-254.

Rebelo AG (1992) Preservation of biotic diversity. Pp 309-344 in RM Cowling (ed.) *The ecology of fynbos: nutrients, fire and diversity*. Oxford University Press, Cape Town.

Rebelo AG, Boucher C, Helme N, Mucina, Rutherford MC (2006) Fynbos biome. Pp 53-219 in Mucina L, Rutherford MC (eds.) *The vegetation of South Africa, Lesotho and Swaziland*. Strelitzia 19. South African National Biodiversity Institue, Pretoria.

Rebelo AG, Homes PM, Dorse C, Wood J (2011) Impacts of urbanization in a biodiversity hotspot: conservation challenges in metropolitan Cape Town. South African Journal of Botany 77, 20-35.

Rebelo AG, Siegfried WR, Crowe AA (1984) Avian pollinators and the pollination syndromes of selected Mountain Fynbos plants. South African Journal of Botany 3, 285-296.

Richardson DM, Cowling RM (1992) Why is mountain fynbos invasible to and which species invade? In: va Wilgen BW, Richardson DM, Kruger FJ, van Hensbergen HJ (eds.), Fire in South African mountian fynbos, pp161-181. Springer, Berlin.

Stock WD, Allsopp N (1992) Functional perspective of ecosystems. Pp 241-259 in RM Cowling (ed.) *The ecology of fynbos: nutrients, fire and diversity.* Oxford University Press, Cape Town.

Vlok JHJ (1988) Alpha diversity of lowland fynbos herbs at various leve;ls of infestation by alien annuals. South African Journal of Botany 54, 623-627,

Wood JC, Low AB, Donaldson JS, Rebelo AG (1994) Threats to plant species diversity through urbanizaation and habitat fragmentation in the Cape Metropolitan area, South Africa. In: Huntley BJ (ed.) otanical diversity in southern Africa, pp259-274. National Botanical Institute, Pretoria.

14 TAPIA FOREST, MADAGASCAR

contributed by Justin Moat & Steven Bachman, Royal Botanic Gardens, Kew

CLASSIFICATION

National: Tapia Forest is recognised as a major vegetation type in the Atlas of vegetation of Madagascar (Rabehevitra and Rakotoarisoa 2007).
IUCN Habitats Classification Scheme (Version 3.0): 1. Forest / 1.5 Subtropical/Tropical Dry Forest

ECOSYSTEM DESCRIPTION

Characteristic native biota

A forest comprising an evergreen canopy of 10–12 m, with an understorey of ericoid shrubs. Lianas are frequent, but epiphytes are few. The herbaceous layer is dominated by grasses (Rabehevitra & Rakotoarisoa 2007).

The primary form of Tapia forest is a stratified, evergreen forest (Fig. S12.1), with a low (8 - 12 m) semi-continuous tree canopy, an understorey of ericoid shrubs, frequent lianas but few epiphytes, and a moderate to rich herbaceous component dominated by grasses (Rabehevitra & Rakotoarisoa 2007). Tapia forest is floristically diverse compared to its surrounding vegetation of denuded grassland and wooded grasslands of the high plateaux. The plants are mostly sclerophyllous, hairy or heliophilous. The tree bark in this type is frequently thick, and resistant to fire. The most characteristic species are *Uapaca bojeri*, *Sarcolaena oblongifolia*, *Pentachlaena latifolia*, *Schizolaena microphylla*, *Asteropeia labatii*, *Weinmannia* spp. and *Agarista* spp. Common canopy families are Cunoniaceae, Anacardiaceae, Rubiaceae, Ericaceae, Sarcolaenaceae and Asteraceae, while two endemic plant families (Sarcolaenaceae and Asteropeiaceae), also characterise the Tapia forest ecosystem. Poaceae and Asteraceae dominate the herbaceous stratum, with *Pachypodium rossulatum* occurring frequently on rock outcrops.

Three mammals occurring within Tapia forests are known to be hunted: *Echinops telfairi*, *Setifer setuosus* and *Tenrec ecaudatus* (Kull 2003a). Insects occurring within this ecosystem include *landibe* (*Borocera cajani*) or wild silk moth (Razafimanantsoa *et al.* 2012).

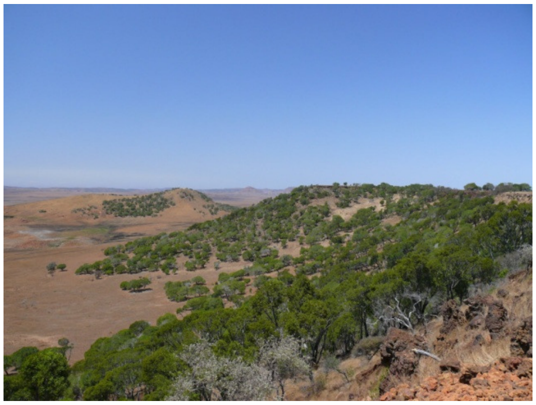

Figure 1a.

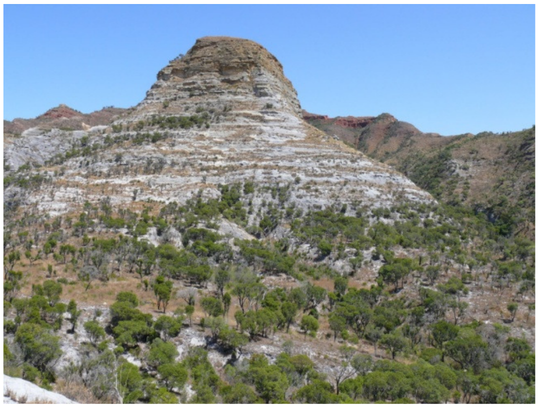

Figure 1b.

Figure S14. 1. (a) Tapia forest on Iron stone hill sides. The dominate canopy tree seen here is *Uapaca bojeri*. Near Isalo National Park Madagascar (Justin Moat). (b) Tapia forest on Sandstone. The

dominate canopy tree seen here is *Uapaca bojeri*. South of Isalo National Park Madagascar (Justin Moat).

Abiotic environment

Tapia forest typically occurs at 500–1,800 metres elevation above sea level on dissected terrain. Edaphic factors are likely to determine Tapia distribution (Kull 2003a, 2003b, 2004). Soils are derived primarily from basement gneiss, quartzite, schist, with frequent intrusions of granite. In the Isalo region (southern extent of the habitat) the forest is on eroded ruiniform sandstone, soils tend to be acidic.

Tapia forest occurs within sub-humid to sub-arid climates (Cornet 1974), although the average annual rainfall ranges from 900 mm up to 1,400 mm with 80–90 rainy days (Gade 1985). Tapia forest tends to occupy rain shadow microclimates, where rainfall is lower and temperatures are higher than the surrounding areas. The dry season ranges from non-existent to eight months or more, though typically occurs from May to September. Following the Köppen–Geiger scheme Tapia forest occurs typically in the tropical savanna to humid subtropical climate.

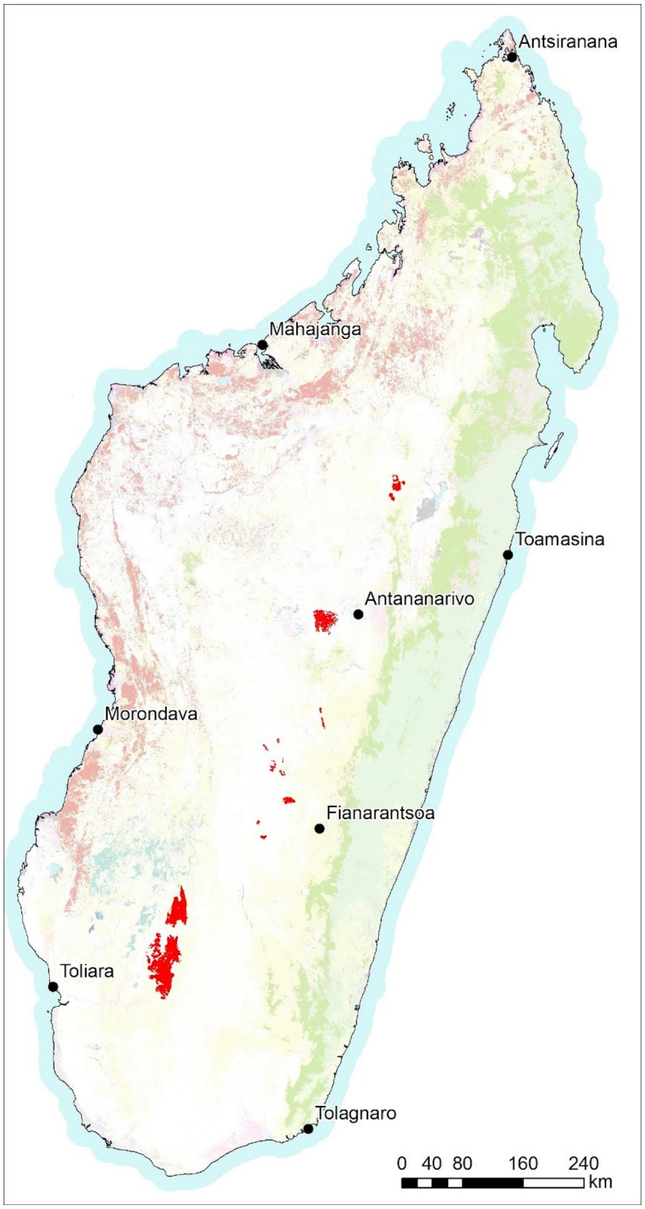

Figure S14. 2. Distribution of Tapia forest (in red, background is vegetation of Madagascar). Extent of vegetation as of 2000 (Moat and Smith 2007)

Distribution

Tapia occurs on the western and central parts of the main plateaux of Madagascar, approximately 17 - 24°S and 44.5 - 48°E (Rabehevitra & Rakotoarisoa 2007, Fig. S13.2). Substantial stands of Tapia forest occur in the south and in Isalo National Park. Smaller patches occur around Ambatofinandrahana and surrounding areas (central Madagascar west of Fianarantsoa) and Morarano (north-east of Antananarivo).

Key processes and interactions

Fire is highly influential in the distribution and diversity of Tapia forest, influencing both survival and regeneration of its component species (Rabehevitra & Rakotoarisoa 2007). this has both positive (see threatening processes) and negative aspects. Other key factors that determine the distribution of Tapia are not clear, although edaphic (soil) factors are likley to have an influence, as Tapia forest is often found on nutrient-poor rocky soils with granites and gneisses (Kull 2003a).

Interactions involving humans also influence the dynamics of Tapia forest, with burning practices likely to have involved significant modification of the system over the 1500 years of human occupation. 'Tapia' itself (*Uapaca bojeri*) is an endemic

species valued for its edible fruit which is high in Vitamin C, also the bark is used in Malagasy folk medicine to relieve diarrhoea (Gade 1985). These uses may influence the dispersal of seeds. In addition, this species is highly prized because it hosts silk worms and thereby supports village-based silk industries, which often afford it some local protection. *Uapaca* usually resprouts from roots and stumps, but can also regenerate through rhizomes and via seed dispersal (Kull 2003a).

Threatening processes

Tapia forest occurs throughout Madagascar's plateau areas, which has ensured its exposure to multiple human pressures including: charcoal manufacture, firewood and timber collecting, grazing and change in fire regime (Rabehevitra & Rakotoarisoa 2007). These threats result in transformation of Tapia forest to grassland. Individual Tapia trees have the ability to regenerate vegetatively when burnt. However, if fires are too frequent, they limit regeneration and species diversity of the herb layer can become very low. Tapia is not ideal for charcoal production due to its slow growth and low energy output, but the human pressure and lack of any alternatives, have dramatically increased the charcoal production from Tapia since the 1970's.

Kull (2003b, 2004) argues that Tapia forest owes its existence to fire, and he rebuffs the assertion that the woodlands are reducing with repeated burning. Small studies in Itermo (central Madagascar around Ambatofinandrahana) and Afotsara region (central Madagascar, between Ambostra and Ansirabe) have shown the forest to be generally stable with a few areas of increase. On the other hand, recent fieldwork south of Isalo has highlighted the increased use of Tapia for charcoal production to fuel the population influxes in the main due to localised mineral mining in the region.

An additional threat comes from non-native species (*Pinus* in the main, but also *Eucalyptus* sp.) that can encroach on Tapia forest and may affect local conditions e.g. soil characteristics, flammability and shade, although further work is needed to understand the extent of this disturbance (Kull 2003a).

Ecosystem collapse

For assessment of criteria A and B, it was assumed that Tapia forest will collapse when its mapped distribution declines to zero.

ASSESSMENT

Summary

Criterion	A	B	C	D	E	overall
subcriterion 1	DD	VU	DD	DD	DD	EN
subcriterion 2	DD	LC	DD	DD		
subcriterion 3	EN	LC	DD	DD		

Criterion A

<u>Current decline</u>: Limited data exist to quantify the current rate of decline (i.e. over the past 50 years) in the distribution of Tapia forest, as this ecosystem is not well respresented in some country-wide mapping of the region.

Moat and Smith (2007) use Harper *et al.* (2007) to show the general trend for the main vegetation types of Madagascar, they quote a 43% reduction in Tapia forest for a 25 year period from 1975 to 2000. However, vegetation types that occur characteristically in small patches (of which Tapia is included) are problematic to classify (Moat and Smith 2007), and the same areas were often classified very differently by Harper *et al.* (2007) and Moat and Smith (2007) due to differences in classification and

mapping methods. Inspection of the data of Harper *et al.* (2007) shows that very little of the Tapia was mapped in 1975, probably because this vegetation can be sparser and occurs in smaller patches compared to the dry and humid forest vegetation types, which were the main vegetation types in that study. The estimates of decline in distribution based on a comparison of mapping from these different sources are thus too uncertain to use for assessment of criterion A1. Further work is needed to clarify the recent changes in Tapia forest extent. As a result, the ecosystem is assessed as Data Deficient (DD) under criterion A1.

Future decline: As of 2006, 20% of the area of Tapia forest was protected in national parks (mainly in Isalo National Park). Proposed increases in number and area of protected areas (SAPM 2011) may eventually limit the ongoing decline in distribution of Tapia forest, if management of these reserves is able to eliminate exploitative activities such as wood harvesting and charcoal production. As present rates of decline are poorly known, however, there is little to drive any analysis of projected declines. Modelling responses to climate change could provide appropriate data for this criterion, but until that is carried out the rating under criterion A2 should be classified as Data Deficient (DD).

Historic decline: The current distribution of Tapia forest is most likely a remnant of a larger and more diverse forest (Kull 2003b, 2004) transformed since the arrival of humans some 1500 years ago. Although the estimates of decline (Table 1) span the entire period of human occupation, it was assumed that most (though not all) of the decline occurred since 1750 due to the increase in human population and activity during this latter period. O'Connor (1996) after Jarozs (1993) states that deforestation started "with the movement of population into the forests after 1896 and [the] French annexation [of Madagascar]".

The magnitude of historical decline was estimated by comparing environmental niche models with maps of present-day distribution. The niche models were developed by applying MaxEnt v3.2 with default settings (Phillips & Dubik 2008) to Bioclim variables (http://www.worldclim.org/bioclim) with sample points selected randomly from mapped Tapia forest over multiple iterations (J. Moat, unpubl.). Similar models were developed for other vegetation types, and Tapia forest was mapped only in pixels where it had the highest suitability value across all vegetation types. Models showing maximum and minimum extent were selection for analysis. The availability of alternative models and maps made it possible to account for uncertainty in estimates of distribution at each point in time. Niche models calculated for Tapia in comparison to the other primary vegetation in the region (unpublished) give a minimum area of 13,900 km^2 and a maximum area of 26,200 km^2. Estimates of present day extent of Tapia forest vary from 1,319 km^2 (Moat and Smith 2007) to 2,600 km^2 (DEF 1996), this variation will be largely due to the methods and scale of mapping. Total historical decline was therefore estimated to be between 81.3% and 95.0% by combining area estimates obtained from different pairs of maps (Table S12.1).

Allowing for a small amount of over-prediction and deforestation prior to 1750, and given qualitative evidence of ongoing declines from the 1990s to present, the decline in distribution since 1750 was therefore assumed to be within the bounds of 70% to 90%. Tapia forest was thus assessed as **Endangered** under criterion A3.

Table S14.1. Estimated historical decline in Tapia based on alternative models of historical distribution and alternative maps of present-day distribution.

	Maximum modelled area **26,200 km^2**	Minimum modelled area **13,900 km^2**
DEF - coverage early 1990's **2,600 km^2**	90.0% decline	81.3% decline

Moat and Smith - coverage in year 2000: **1,319 km²**	95.0% decline	90.5% decline

Criterion B

Approximately 1,319 km2 of Tapia has been mapped from landsat satellite imagery (Moat and Smith 2007) for the year 2000. Tapia forest is often highly fragmented and sparse within the landscape.

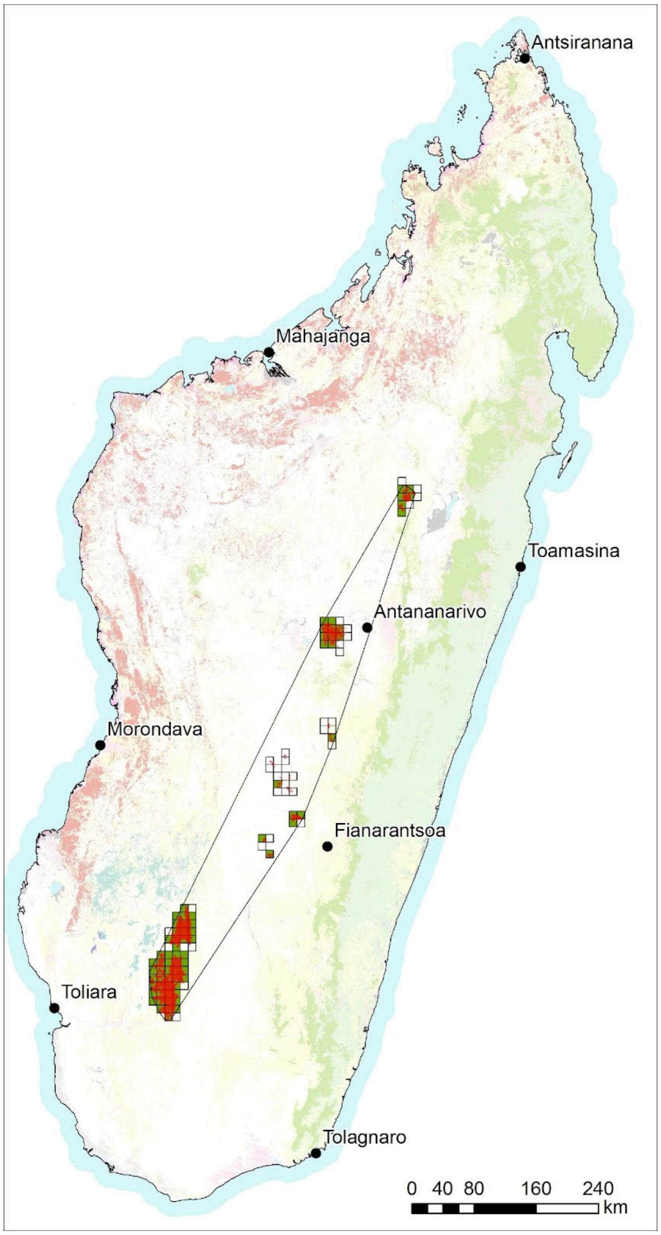

Figure S14. 3. Distribution of Tapia after Moat and Smith 2007, showing minimium convex polygon (extent of occurrence). Cells 10 x10 km (Area of occupancy) green cells are occupied with more than 1% occupancy, white cells less than 1%.

Extent of occurrence: The minimum convex polygon enclosing all occurrences (Figure S12.3) as mapped in 2000 (Moat and Smith 2007), has an area of 47,485 km2. There is good evidence for a decline in distribution (see criterion A1). Little work has been done on the future decline and quality of the ecosystem, but ongoing human pressures and plausible climate change scenarios suggest that the ecosystem is undergoing a continuing decline in distribution. Also we have observed that the ecosystem exhibits less biodiversity (and therefore decline in quality) in areas of frequent fires, especially those areas not formally protected or not safeguarded by local human populations. Insufficient information about the spatial pattern of threats precluded an estimate of the number of locations. Therefore, the status of the ecosystem under B1a(ii)b is Vulnerable.

Area of occurrence: Spatial analysis revealed that 108 10 x 10 km cells were occupied with Tapia Forest, of which 73 had over 1% level of occupancy (Figure S12.3). This estimate of Area of Occupancy is beyond the 50 grid cell threshold for Vulnerable, and hence Tapia forest was assignd a status of Least Concern under Criterion B2.

Number of locations: As noted above, too little is known about the spatial patterns of threatening processes to estimate the number of locations. Although threats are ongoing, they are not likely to rapidly affect the ecosystem. Hence the second requirement of criterion B3 is therefore unlikely to be met, even though the number of locations is unknown. The status of the ecosystem under Criterion B3 is therefore Least Concern.

Criteria C and D

Tapia forest is too poorly studied to estimate the extent and magnitude of degradation in abiotic components or biotic processes and interactions within the ecosystem. Some of the Tapia forest will have low diversity due to fire and mans influcence, but a present it would be very difficult to disentangle this from the abiotic influences (ie soils, microclimate etc). The status of the ecosystem is therefore Data Deficient under criterion C and D.

Criterion E

No quantitative analysis has been carried out to assess the risk of ecosystem collapse for Tapia forest. The status of the ecosystem is therefore Data Deficient under criterion E.

REFERENCES

Cornet A. (1974) Essai de cartographie bioclimatique à Madagascar. NoticeExplicative. ORSTOM No. 55

DEF (Direction des Eaux et Forêts, Deutsche Forstservice GmbH, Enterprise d'Etudes de Développement Rural 'Mamokatra' and Foiben-Taosarintanin'I Madagasikara) (1996) Inventaire ecologique forestier national: situation de départ, résultats, analyses et recommandations Ministère de l'Environnement, Plan d'Actions Environnementales, Programme Environnemental – Phase 1 Direction des Eaux et Forêts, République de Madagascar.

Gade DW (1985) Savanna Woodland, Fire, Protein and Silk in Highland Madagascar. *Journal of Ethnobiology* **5**, 109-122.

Harper GJ, Steininger, MK, Tucker CJ, Juhn D. and Hawkins F. (2007). Fifty years of deforestation and forest fragmentation in Madagascar. *Environmental Conservation* **34**, 325-333. 34: 1. doi:10.1017/S0376892907004262

Jarosz L (1993) Defining and Explaining Tropical Deforestation: Shifting Cultivation and Population Growth in Colonial Madagascar (1896-1940). *Economic Geography* 69, 366-379. [Environment and Development, Part 2]

Moat, J, Paul S. (2007). *Atlas of the Vegetation of Madagascar.* Kew Publishing, Kew.

Kull CA, Goodman SM., Benstead JP. (2000). Isle of fire: The political ecology of grassland and woodland burning in highland Madagascar. In S. M. Goodman & J. P. Benstead (Eds.), *The Natural History of Madagascar* (pp. 1-424). The University of Chicago Press.

Kull,CA. (2002). The "degraded" tapia woodlands of highland Madagascar: Rural economy, fire ecology, and forest conservation. *Journal of Cultural Geography*, *19*, 95-128.

Kull CA. (2003a) *Uapaca* Woodlands. In SM Goodman & JP Benstead (Eds.), *The Natural History of Madagascar* (pp. 393-398). The University of Chicago Press, Chicago.

Kull CA. (2003b) Fire and the Management of Highland Vegetation. In S. M. Goodman & J. P. Benstead (Eds.), *The Natural History of Madagascar* (pp. 153-157). The University of Chicago Press, Chicago.

Kull CA. (2004) *Isle of fire. The political ecology of landscape Burning in Madagascar.* (1st ed., p. 325). University of Chicago Press, Chicago. Retrieved from: http://books.google.co.uk/books?id=yd07nTGT9PwC&lpg=PA120&ots=TyiphAtLS2&dq=sclerophyllous tapia&pg=PA116#v=onepage&q=sclerophyllous tapia&f=false

O'Connor A (1996) What are the historical causes of deforestation in Madagascar and what is the situation today?. from Tietenberg T Sustainable Development Case Studies: http://www.colby.edu/personal/t/thtieten/defor-mad.html

Phillips SJ, Dudik M (2008) Modeling of species distributions with Maxent: new extensions and a comprehensive evaluation. Ecography 31, 161-175.

Rabevehitra D, Lowry II PP. (2009). Endemic families of Madagascar. XI. A new critically endangered species of Schizolaena (Sarcolaenaceae) from Tapia woodlands in south-central Madagascar. *Young*, *31*(1), 149-155.

Rabehevitra D, Rakotoarisoa S. (2007) Tapia forest in Moat, J., & Paul, S. Atlas of the Vegetation of Madagascar. Kew Publishing. p38-39

Razafimanantsoa TM, Rajoelison G, Ramamonjisoa B, Raminosoa N, Poncelet M, Bogaert J, Haubruge E, Verheggen FJ. (2012) Silk moths in Madagascar: A review of the biology, uses, and challenges related to Borocera cajani (Vinson, 1863) (Lepidoptera: Lasiocampidae). *Biotechnology, Agronomy, Society and Environment (BASE).* 16, 269-276

SAPM (2011) *Atlas numerique du SAPM.* Rebioma. http://atlas.rebioma.net/index.php?option=com_wrapper&Itemid=39

15 GREAT LAKES ALVAR , NORTH AMERICA

contributed by Don Faber-Langendoen (NatureServe, Conservation Science Division, Arlington, VA). and Wasyl Bakowsky (Natural Heritage Information Centre, Ontario Ministry of Natural Resources, Peterborough, ON).

CLASSIFICATION

International Vegetation Classification :

Eastern North American Grassland, Meadow & Shrubland Division (D024 2.B.2.Nc)
Northern Alkaline Scrub - Herb & Outcrop Vegetation MacroGroup (M507)
Great Lakes Alvar Group (G061).

IUCN Habitats Classification Scheme (Version 3.0): 4. Grassland / 4.4 Temperate Grassland

Key References: Reschke et al. (1999), Catling and Brownell (1999).

ECOSYSTEM DESCRIPTION

Characteristic native biota

This type is characterized by a variable physiognomy, from open perennial (rarely annual) grassland or shrubland and nonvascular pavement (5-25% herb and or shrub cover) to dense grassland or shrubland (> 25%) with scattered evergreen needleleaf (more rarely broadleaf deciduous) trees < 10%, but variable) (Figure 1). Species composition contains a mix of tallgrass prairie graminoids and forbs and sub-boreal to boreal shrubs and trees. Sites are on thin-soil, limestone pavement, with a xero-hydric moisture regime. Key dominants and differentials include the perennials *Schizachyrium scoparium, Sporobolus heterolepis*, *Danthonia spicata* and *Deschampsia caespitosa*; less commonly with *Sporobolus neglectus, Sporobolus vaginiflorus*, and *Panicum philadelphicum*. Key shrubs, when present, are *Juniperus communis ,J. horizontalis, Dasiphora fruticosa* ssp. *floribunda and Rhus aromatica*. Trees, when present, include *Thuja occidentalis, Picea glauca, Pinus banksiana*, and *Abies balsamea* (in more northern sites) and *Juniperus virginiana, Quercus macrocarpa* or *Quercus muehlenbergii* (more southern sites).

Abiotic environment

Climate: Alvars are found in humid and subhumid climates.
Soil/substrate/hydrology: Alvars are centered on areas of glaciated horizontal limestone/dolomite (dolostone) bedrock pavement with a discontinuous thin soil mantle. Most hydrologic studies of alvars in the Great Lakes region have concentrated on Chaumont Barrens in New York State (Feeney 1996, 1997, Reschke et al. 1999). Reschke (1995) found strong correlations between soil moisture conditions and vegetation types, with "alvar grasslands" (equivalent to tufted hairgrass wet alvar grassland) located in the wettest, seasonally flooded areas, and "calcareous pavement barrens" (equivalent to juniper alvar shrubland) in the drier, never-flooded areas. At the Limerick Cedars alvar, also in New York State, Gilman (1995) observed that alvar community structure was influenced by rapidly changing environmental conditions and differential tolerances of plants, especially to periodic drought.

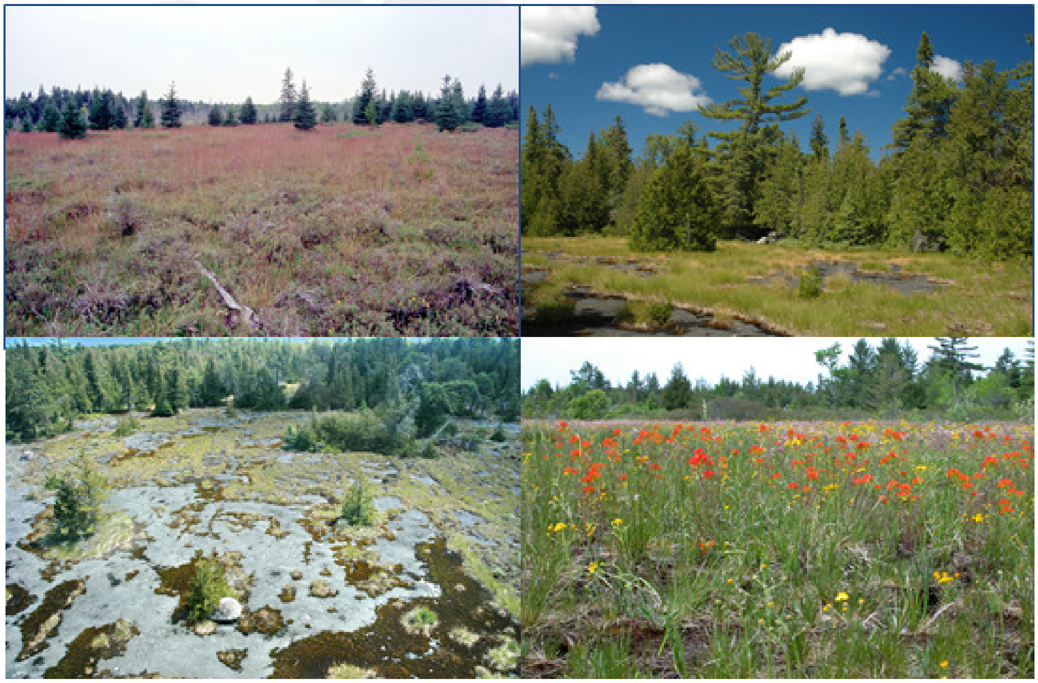

Figure S15. 1. Typical examples of alvar in Ontario, Canada, showing clockwise from top right: little bluestem – shrubby cinquefoil (*Schizachyrium scoparium, Dasiphora fruticosa* ssp. *floribunda*) dwarf-shrub grassland, in Carden Township; prairie dropseed (*Sporobolus heterolepis*) grassland, with white cedar (*Thuja occidentalis*) limestone woodland in background, on Manitoulin Island; alvar grassland with *Deschampsia cespitosa* in Carden Township; alvar nonvascular pavement on Manitoulin Island. All photos by Wasyl Bakowsky.

Distribution

Almost all of North America's alvars occur within the Great Lakes basin, primarily in an arc along the Niagaran Escarpment from northern Lake Michigan across northern Lake Huron and eastern Ontario and northern New York State (Figure 2).

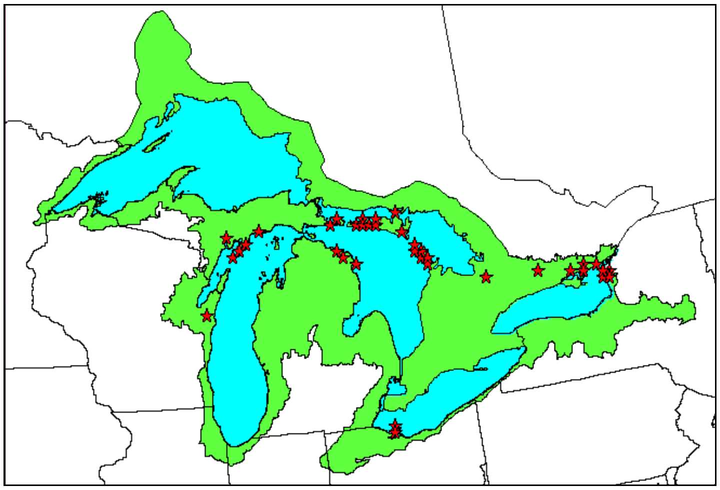

Figure S15. 2. Distribution of Great Lakes Alvar sites within the Great Lakes basin (green area). A few Great Lakes alvar sites occur near, but outside, the basin.

Key processes and interactions

Hydrology: The extreme range in hydrologic conditions on a relatively flat, fractured limestone with thin soils habitat appears to be a principal factor in limiting the establishment of woody tree species and maintaining grassland and other open alvar communities (Figure 3; Stephenson and Herendeen 1986, Reschke 1995). The shallow soils limit rooting depth, making them prone to summer drought. Hard summer droughts can result in a die-back of woody plants that do not have their roots in moist bedrock cracks, along with an increased diversity of annual alvar plants the following year (Stephenson and Herendeen 1986).

Fires: Natural fires appear always to have been at least an incidental part of alvar history, and probably instrumental in maintaining some alvar types, such as juniper alvar shrubland. It is estimated that fire return intervals range from 200-500 years in the Great Lakes basin (Jones and Reschke 2005). From the alvars with old trees and no burn evidence, it is clear that not all alvars require fire to remain in an open state, although it may be beneficial for some sites such as Stone Road Alvar on Pelee Island (Reschke et al. 1999). A post-fire successional alvar shrubland that replaced semi-open white cedar woodland adjacent to open alvar near Ottawa had twice as many species present, including many that were regionally rare (Catling 2009). Persistence of invertebrate fauna may depend on local patchiness of burnt areas.

Grazing: Alvars in the Great Lakes basin and elsewhere have long been influenced by grazing livestock. While this influence has been little studied in North America, the effects of grazing have been documented on alvar habitats of the Swedish island of Oland, where grazing by domestic animals has occurred since the first centuries A.D. (Titlyanova et al. 1988). The intensity of grazing appears to be a critical factor in maintaining open conditions. A comparison of ungrazed, moderately grazed, and overgrazed sites showed decreased biomass and floristic changes in the overgrazed area, with perennial and annual ruderal (quick-germinating, disturbed soil specialists) species replacing the dominant alvar grassland species (Brownell (1998). The Alvar Working Group suggested that cattle grazing is generally detrimental to alvar communities, but some light grazing may help to keep alvar areas open. Also, as the intensity of grazing increases, diversity of native species decreases and the number of exotics increases (Reschke et al. 1999).

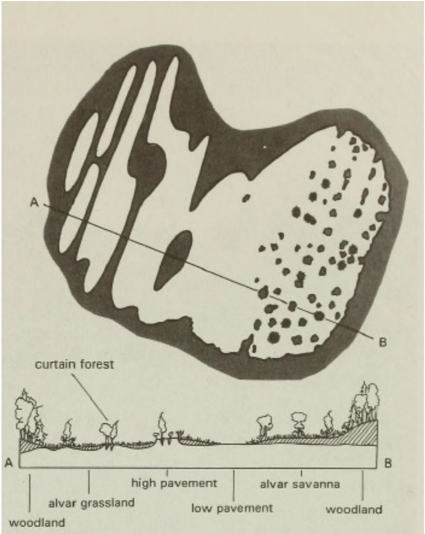

Figure S15. 3. Key hydrological and substrate processes shape the alvar found on the relatively flat, fractured limestone with thin soils, as shown from the diagrammatic cross-section of an alvar complex. Types include alvar grassland, alvar pavements, and alvar savanna, and adjacent limestone woodland (from Catling and Brownell 1995). Used with permission from Canadian Field Naturalist and the authors.

Threatening processes

Across their Great Lakes range, alvar habitats face a daunting series of threats to their future survival and quality. For example, The NatureServe protection urgency rankings for multiple-value alvar sites places 56% of the sites with high or very high securement urgency, and 53% with high or very high management urgency (Reschke et al. 1999, Table 10). Although the nature and extent of these threats tend to be site-specific and constantly changing, a number of common factors emerge:

Quarrying: The loss of alvar habitats to quarries (for easily accessible limestone with no overburden) has taken place across the Great Lakes basin and continues to be a primary threat in many places.

Residential and related development: The construction of rural residences, cottages and second homes, trailer parks, and other forms of low-density rural development is an ongoing threat to many alvar habitats, especially shoreline alvars. A diverse mix of other rural developments can be located on alvar habitats.

All-terrain vehicle and off-road vehicle use: Recreational users of all-terrain vehicles, trail bikes, and off-road trucks are attracted to some alvar areas because of their flat open terrain and remoteness. The rutting caused by these vehicles disrupts local hydrological patterns, creates conditions suitable for the invasion by exotic species and visually scars the alvar surface. Snowmobiles are also used on many alvar sites, but their impact appears to be substantially less, or at least less documented.

Grazing and browsing: Many grassland alvars have been grazed by cattle for decades. The degree of threat posed by grazing to alvar quality is the subject of ongoing research. While intensive grazing appears to be associated with the loss of some alvar species and an increased presence of exotic species, light grazing helps to maintain the open character of some alvars. For some alvar-related fauna such as loggerhead shrike (*Lanius ludovicianus migrans*), grazing to maintain short grass conditions appears to be an essential habitat requirement for nesting. Deer browsing is also an important factor in most Great Lakes alvar sites. High deer densities, possibly due to loss of top predators, may be preventing successful regeneration of some alvar species, but the longer-term effects are uncertain at this stage.

Exotic species: Virtually all Great Lakes alvars include a diverse mix of exotic species in their flora and fauna, but the extent and trends of non-native species populations vary widely. Aggressive species which are problematic include buckthorn (*Rhamnus cathartica*), common St. John's wort (*Hypericum perforatum*), rough-fruited cinquefoil (*Potentilla recta*), Dog-strangling Vine (*Cynanchum rossicum*) and many others. Canada blue grass (*Poa compressa*), which is considered by most experts to be an introduced species, is also well established on many alvar sites. These exotic species compete for space and nutrients with native species and, in some cases, become dominant, significantly reducing the ecological value of alvar communities.

Plant collecting: The extent of plant collecting on alvars by hobbyists is unknown, but the removal of stunted old-growth cedars and other trees by bonsai collectors is a serious management problem on the Bruce Peninsula. Other showy wildflowers of alvars, such as dwarf lake iris (*Iris lacustris*) and several orchid species, are also at risk from collectors or from careless photographers who trample surrounding vegetation.

Logging and forestry: Logging of mature trees from alvar savannas and adjacent woodlands can disrupt the landscape integrity of alvar sites. In some instances, the flat open areas provided by alvars have been used as log assembly areas or skidways, resulting in serious damage to shallow soils and vegetation communities from rutting and accumulation of bark and other debris. A related issue is the inappropriate planting of alvar sites with trees designed to provide a future commercial crop.

Ecosystem collapse

For assessment of criteria A and B, collapse was assumed to occur when the mapped distribution of the ecosystem declines to zero, signalling the replacement of alvar by developed areas (e.g. quarries, rural-residential development and roads). For criterion C, ecosystem collapse was defined based on degree of mechanical disturbance to the soil substrate. Collapse was assumed to occur between 50 and 90% of

the alvar surface was severely degraded, as basic abiotic states and processes are sufficiently damaged to make them unrestorable. Severely degraded processes include trampling, creation of berms, or removal of rocks and/or soil (Reschke et al. 1999). Under criterion D, the abundance of invasive species is the most appropriate biotic variable for assessment, as these species alter the native biotic composition. Collapse was assumed to occur when the abundance of exotic species exceeds between 50 and 90%, a sufficiently high level of exotics such that restoration of natural processes could not successfully recover a typical set of alvar species and interactions, and when > 90% of the surrounding landscape was intensive agriculture, commercial or residential development (Reschke et al. 1999).

ASSESSMENT

Summary

Criterion	A	B	C	D	E	overall
subcriterion 1	DD	LC	DD	VU(NT- VU)	DD	EN(VU-EN)
subcriterion 2	DD	VU(NT-VU)	DD	DD		
subcriterion 3	EN(VU-EN)	LC	DD	DD		

Criterion A

Current decline: The Great Lakes Alvar has experienced a wide range of threats (see Threatening Processes above). Based on the combination of these threats, the distribution of the ecosystem is likely to have declined by > 30% over the past 50 years. But the data are insufficient to document this trend, so the status of the ecosystem is therefore Data Deficient under criterion A1.

Future decline: The future persistence of Great Lakes Alvar is affected by a wide range of threats (see Threatening Processes above). Based on the combination of these threats, the distribution of the ecosystem is likely to decline by > 30% over the next 50 years. But the data are insufficient to document this trend, so the status of the ecosystem is therefore Data Deficient under criterion A2

Historic decline: In 1999, alvars were estimated to cover approximately 11,200 ha (112 km^2) of reasonable quality (NatureServe integrity ranks A–C) across the entire Great Lakes Basin (Reschke et al. 1999), and, combined with more recent survey work in Ontario, that total is now closer to 130 km^2 of reasonable quality alvar, with Ontario containing almost 100 km^2, and 30 km^2 is found in Michigan, Ohio and New York. Considerable areas of alvar that have been substantially degraded by agricultural and other uses are excluded from these estimates.

There is little data to estimate changes in alvar since 1750. We have two sources. First, Catling and Brownell (1995) estimate that the extent of alvar in southern Ontario prior to settlement was 1100 to 1500 km^2, including both open alvar and limestone woodland, but they provided no clear distributional boundaries. A substantial portion of the original extent has been lost. In 1999, alvars were estimated to cover approximately 11,200 ha (112 km^2) of reasonable quality (NatureServe integrity ranks A –C) across the entire Great Lakes Basin (Reschke et al. 1999). We can develop approximate estimates for the upper bound of historic loss across Ontario as follows: we assume that the remaining areas of degraded habitat are roughly comparable to that of reasonable quality (i.e. 100 km^2 from above), giving a total of 200 km^2, and we choose the lower estimate of historic extent (1100 km^2), which gives a decline in distribution since 1750 of 82%. Second, we also estimated loss within one township (Carden) within the Carden Plains region, based on reconstruction of presettlement maps from early surveys and current extent (Bakowsky unpublished). We determined that the current extent of alvar is 1335 ha = 13.35 km^2 and the presettlement estimates total 35 km^2, giving values of 38% still remaining, or 62% loss since settlement. Assuming that changes to alvar distribution in Carden and Ontario are indicative of a range-wide decline of 62 - 82%, and given that Ontario accounts for the

majority of the distribution, the status of the ecosystem is Endangered (plausible range Vulnerable to Endangered) under criterion A3.

Criterion B

In 1999, alvars were documented to cover approximately 11,200 ha (112 km^2) of reasonable quality (integrity ranks A – C) across the entire Great Lakes Basin (Reschke et al. 1999). More recent surveys suggest the total is closer to 13,000 ha (130 km^2), see "Criterion A: Historic decline" above.

Extent of occurrence: A minimum convex polygon enclosing all mapped occurrences of Great Lakes alvar (Figure 4) has an area of 153,000 km^2 based on moderately conservative criteria for its distribution and over 295,000 km^2 if outliners are included. The status of the ecosystem is therefore Least Concern under criteria B1.

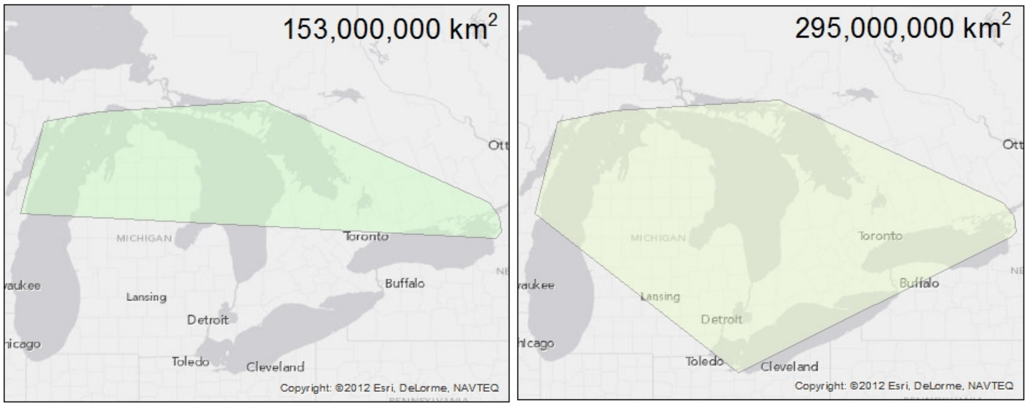

Figure S15. 4. Mapped distribution of Great Lakes Open Alvar showing minimum convex polygon enclosing all occurrences (Extent of occurrence). Compare with Figure 2 above.

Area of occurrence: Confirmed occurrences of Great Lakes Alvar (Fig. S15. 2) occupy at least 37 10 × 10 km grid cells, but there is some uncertainty in this estimate and there could be as many as 60 occupied cells. The Great Lakes Alvar ecosystem meets subcriterion B2b because of continued disruption by exotic plants and continued degradation from quarrying, low density rural development, such as cottages and second homes, and off-road vehicles that disrupt the natural water flow patterns and create ideal conditions for invasions by exotic species (See Threatening Processes above). With respect to criterion B2c, there are well over 10 locations. The status of the ecosystem is therefore Vulnerable (plausible range Near Threatened to Vulnerable) under criteria B2b.

Number of locations: Based on the widespread distribution of Great Lakes Alvar throughout the Great Lakes Basin, there are far more than 5 locations present, and likely at least 37 because of the localised nature of threats. The status of the ecosystem is therefore Least Concern under criteria B3.

Criterion C

The principal mechanism of environmental degradation is through continued degradation from quarrying, low density rural development, such as cottages and second homes, and off-road vehicles that disrupt the natural water flow patterns and create ideal conditions for invasions by exotic species. Above, we assumed that collapse would occur between 50 and 90% of the alvar surface was severely degraded by these processes. But current methods of evaluation typically give emphasis to the biotic response of alvars to these threats and there are insufficient data on the status of alvar soils across their range. Thus we have insufficient data to independently evaluate this criterion. The status of the ecosystem is Data Deficient under criteria C1, C2 and C3.

Criterion D

Ongoing disruption of ecosystem function is caused by continued degradation from quarrying, low density rural development, such as cottages and second homes, and off-road vehicles that disrupt the natural water flow patterns and create ideal conditions for invasions by exotic plant species. Our strongest data are for exotics, which have been obtained in surveys focussed on the high quality Alvar remnants (NatureServe integrity ranks A and B), which account for a minority of Alvar occurrences. Estimates from Reschke et al. (1999) show that the median cover of exotics by alvar type, in the higher quality stands, varied from 0.1 to 20%, and the maximum cover value varied from 5% to 75%. Based on historical development of the region, we assumed that the increase in cover of exotic plants occurred primarily during the past 50 years. Given a collapse threshold of 50-90% exotic cover, the relative severity of decline in some of the higher quality stands could be as low as 0.1% (0.1/90) or as high as 100% (75/75). The median exotic cover values for more degraded remnants of the higher quality Alvar types suggest a functional decline with relative severity of up to 40% (20/50) during the 50-year period.

As noted previously, data are unavailable for lower quality stands, which currently precludes a formal calculation of the relative severity of functional decline across the entire ecosystem. Nonetheless, we inferred that Alvars underwent a functional decline over the past 50 years with a relative severity of at least 30% over at least 80% of the ecosystem extent based on the following:

i) cover of exotics in an appreciable portion of higher quality stands had reached 20% by 1998, corresponding to functional decline with a relative severity of up to 40%;
ii) higher quality stands (ranked A or B) account for a minority of Alvar distribution;
iii) lower quality stands (ranked C or lower), which make up the majority of Alvar distribution, are likely to have suffered higher levels of invasion than high-quality stands (ranked A or B); and
iv) cover of exotic plants has continued to increase since 1998, and hence estimates based on the survey data are likely to further under-estimate the severity of functional decline.

Given the uncertainty about the severity of invasion in low-quality stands, estimates of severity and extent just below 30% and 80%, respectively, are plausible. The status of the ecosystem was therefore assessed as Vulnerable (plausible range Near Threatened - Vulnerable) under criteria D1b.

Criterion E

No quantitative analysis has been carried out to assess the risk of ecosystem collapse for Great Lakes Alvar. The status of the ecosystem is therefore Data Deficient under criterion E.

REFERENCES

Brownell, Vivian R. 1998. Significant alvar natural heritage areas in the Ontario Great Lakes region: a preliminary discussion paper. Prepared for Federation of Ontario Naturalists, Toronto. 54 pp.

Catling, P.M. 2009. Vascular plant diversity in burned and unburned alvar woodland: More evidence of the importance of disturbance to biodiversity and conservation. Canadian Field Naturalist 123: 240-245.

Catling, P. M. and V. R. Brownell. 1995. A review of the alvars of the Great Lakes region: distribution, floristic composition, biogeography and protection. Canadian Field-Naturalist 109(2): 143-171.

Catling, P M. and V. R. Brownell. 1999. Alvars of the Great Lakes region. Pp 375-391 *In* R.C. Anderson, J.S. Fralish, and J.M Baskin. Savannas, Barrens, and Rock Outcrop Plant Communities of North America. Cambridge University Press. New York.

Feeney, T.P. 1996. The Source of Seasonal Flood Waters in Alvar Grasslands: Chaumont Barrens, New York State. Unpublished report to The Nature Conservancy.

Feeney, T.P. 1997. The Geomorphic Evolution of Limestone Pavements and Alvar Grasslands in Northwestern New York State, USA. Unpublished Ph.D. dissertation, University of Georgia, Athens GA. 311 p.

Gilman, Bruce A. 1995. Vegetation of Limerick Cedars: Pattern and Process in Alvar Communities. Unpublished dissertation, SUNY College of Environmental Science and Forestry, Syracuse, NY. 322 pp.

Jones, J. and C. Reschke. 2005. The role of fire in Great Lakes alvar landscapes. Michigan Botanist 44: 13-27.

Reschke, Carol. 1995. Biological and hydrological monitoring at the Chaumont Barrens Preserve. Unpublished report for The Nature Conservancy's Rodney Johnson Grants Program, Grant #R93NY01. 65 p., plus 4 appendices. Copies available from The Nature Conservancy, Arlington, VA.

Reschke, C., R. Reid, J. Jones, T. Feeney, and H. Potter, on behalf of the Alvar Working Group. 1998. Conserving Great Lakes Alvars. Final Technical Report of the International Alvar Conservation Initiative. The Nature Conservancy, Great Lakes Program, Chicago, IL. 119 pp. plus 4 appendices.

Stephenson, S. N., and P. S. Herendeen. 1986. Short-term drought effects on the alvar communities of Drummond Island, Michigan. Michigan Botanist 25:16-27.

Titlyanova, A., G. Rusch, and E. van der Maarel. 1988. Biomass structure of limestone grasslands on Oland in relation to grazing intensity. Acta Phytogeographica Suecica 76:125-134.

16 GIANT KELP FORESTS, ALASKA

contributed by David Keith, Australian Wetlands and Rivers Centre, University of New South Wales and NSW Office of Environment & Heritage

CLASSIFICATION

International: Recognised by Steneck et al. (2002) as one of 19 subtidal kelp ecosystems of the world.
IUCN Habitats Classification Scheme (Version 3.0): 9. Marine Neritic / 9.7 Macroalgal/Kelp

ECOSYSTEM DESCRIPTION

Characteristic native biota

Alaskan kelp forests are structurally and functionally diverse assemblages (Figure 1). They are characterised by species of brown algae in the Order Laminariales including *Nereocystis luetkeana, Laminaria groenlandica, Alaria fistulosa, Agarum fimbriatum* and *Thalassiophyllum* sp. (Steneck et al. 2002). These create a complex and dynamic layered forest architecture up to 15 m tall, that provides substrate, shelter and foraging resource for a diverse fauna assemblage of epibenthic invertebrate herbivores (Steneck & Watling 1982. and pelagic vertebrate predators (Estes et al. 2009). Characteristic invertebrates include urchins, *Strongylocentrotus franciscanus*, *S. purpuratus* and *S. droebachiensis,* limpets, and starfish, *Solaster* spp. Fish, including the Pacific cod (*Gadus macrocephalus*) and rock greenling (*Hexagrammos lagocephalus*., are important predators that depend directly or indirectly on the ecosystem (Reisewitz et al. 2005). Characteristic mesopredators include sea otters, (*Enhydra lutris*., harbour seals (*Phoca vitulina*). Steller sea lions (*Eumetopias jubatus*) and northern fur seals (*Callhorinus ursinus*). Steller's sea cow (*Hydrodamalis gigas*), now extinct, was a functionally unique herbivorous member of the vertebrate assemblage (Domning 1972). Large pelagic predators are also important components of the ecosystem, including killer whales (*Orcinus orca*. and over 15 species of great whales including sperm (*Physeter macrocephalus*) and fin whales (*Balaenoptera physalus*).

The ecosystem is distinguished compositionally from other kelp forests, which are generally separated geographically by continental land masses or deep sea. The Alaskan kelp forests are continuous with those off California, which differ in their more diverse assemblage of macroalgae, including *Macrocystis pyrifera*.

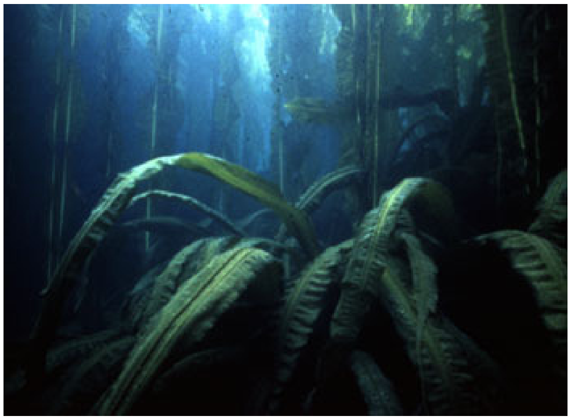

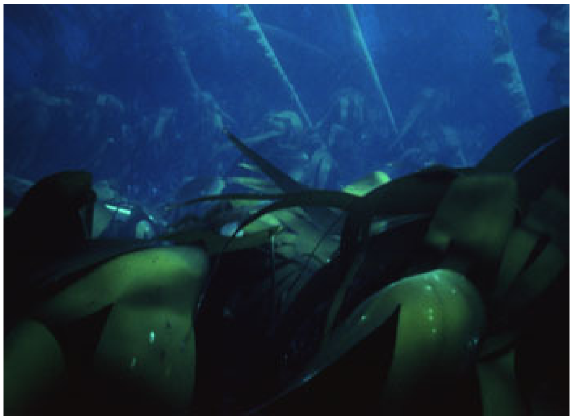

Figure S16. 1. Alaskan kelp forest near Aleutian Islands (left. dominated by *Cymathera triplicata* in foreground with *Alaria fistulosa* in rear and (right. dominated by *Laminaria groenlandica.* Photos by David Duggins, University of Washington (Source: Stony Brook University, http://life.bio.sunysb.edu/marinebio/kelpforest.html).

Abiotic environment

Kelp forests are confined to cold, nutrient-rich, open, rocky coastal waters, A dependence upon light for photosynthesis restricts them to clear shallow water, rarely deeper than 15-40m.

Distribution

Alaskan kelp forests occur in nearshore marine environments of the western Gulf of Alaska, Aleutian Islands and eastern Bering Sea between latitudes of about 45–60° N (Figure 2).

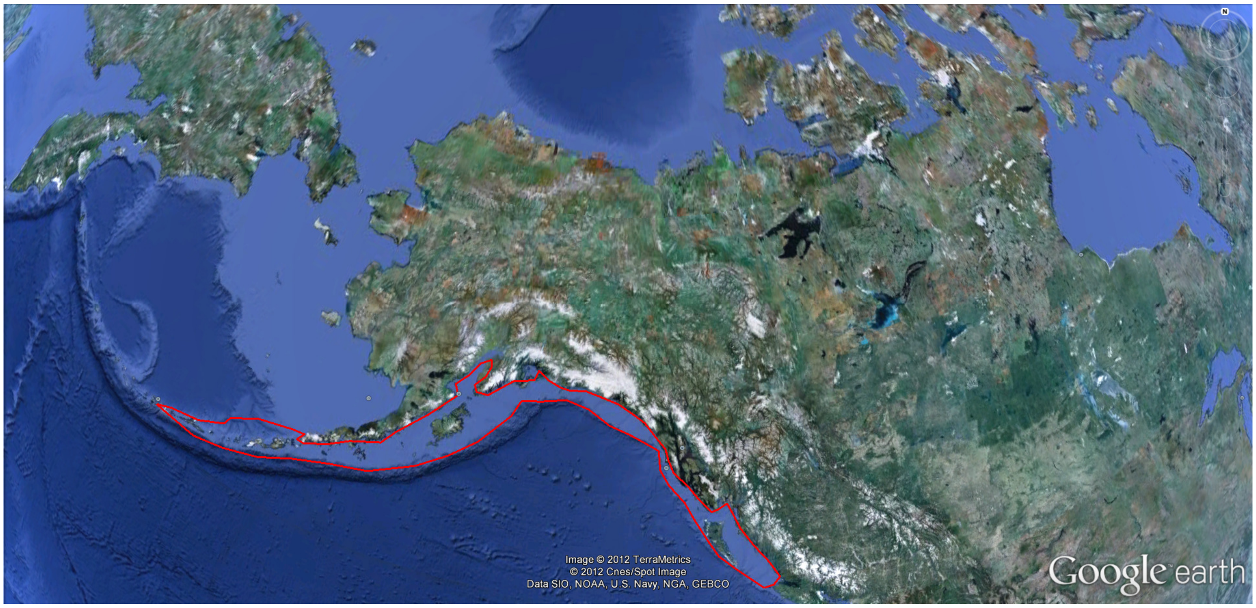

Figure S16. 2. Approximate distribution of Alaskan kelp forests off the coast of North America, north from about 40°N. Adapted from Raffaelli & Hawkins (1996) and, Steneck et al. (2002).

Key processes and interactions

Kelp canopies influence the near-shore marine environment and its biota by dampening waves, reducing light and providing a three-dimensional architecture within the water column. Through its influence on wave action and water movement, kelp affects coastal erosion, sedimentation, upwelling, benthic productivity and recruitment of benthic invertebrates (Duggins *et al.* 1990). Reduced light intensity under kelp canopies creates benthic conditions favourable for particular species (Santelices & Ojeda 1984) and affects competitive relationships among algal species (Dayton 1985). Kelp forest architecture provides shelter, breeding sites and food for pelagic and benthic organisms (Bernstein & Jung 1980; Bologna & Steneck 1993; Levin 1994; Anderson *et al.* 1997), while the kelp tissue itself provides substrates for epiphytic algae and sessile animals Duggins 1980; Reed & Foster 1984; Dunton & Schell 1987), as well as food for herbivores and detritivores (Linley *et al*. 1981; Duggins *et al*. 1989; Mann 2000), including some trophic specialists (Steneck & Watling 1982; Estes & Steinberg 1988). The kelp forests are part of an extensive trophic network that extends to the open ocean and terrestrial habitats (Estes et al. 2009; Figure 3).

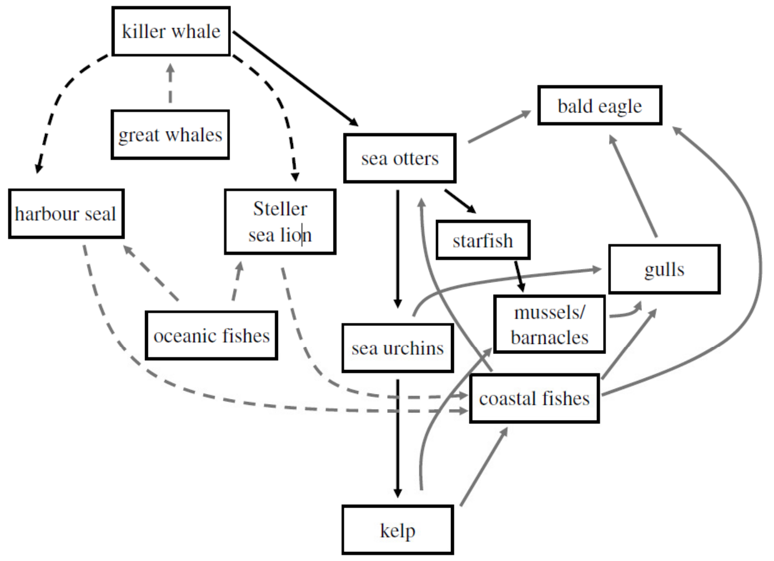

Figure 2. Food-web relationships among selected species in the North Pacific Ocean and southern Bering Sea. The arrows represent linkages for which there are known (solid lines) or suspected (dashed lines) dynamic interactions. Black arrows represent top-down forcing and the grey arrows represent bottom-up forcing. Strong dynamic responses are known for food-web pathways that connect at least eight species (e.g. great whales → killer whales → sea otters → sea urchins → kelp → coastal fishes → gulls → bald eagles). See text for explanations.

Figure S16. 3. Trophic interactions intrinsic and extrinsic to Giant Alaskan Kelp Forests (from Estes et al. 2009).

Disturbance regimes, competition, and predation are key processes that influence kelp forest ecosystem dynamics. Competition strongly regulates recruitment and establishment, which occurs largely within canopy gaps created by population thinning or disturbance (Reed & Foster 1984; Chapman 1986). structure Outcomes of interspecific competition depend on differential growth rates, which are regulated by water temperatures and availability of light and nutrients, and vary seasonally and between years (Steneck et al. 2002). Canopy gaps of varying size may be created by disturbances such as thermal events, salinity anomalies and storms, although gaps created by these physical disturbances tend to be relatively small and short-lived (Tegner *et al*. 1997; Steneck et al. 2002).

More extensive deforestation may result from biological interactions such as outbreaks of disease or herbivore populations. Kelps are more susceptible to lethal diseases when under physiological stress initiated by low nutrient availability, high salinity or high temperatures (Steneck et al. 2002). Herbivory by the extinct Steller's sea cow may have influenced the availability of gaps, but its overall influence on kelp forest dynamics is poorly known (Tegner & Dayton 2000). Overgrazing by sea urchins causes the most widespread and long-lasting denudation of Alaskan kelp forests (Duggins 1983; Estes & Duggins 1995). While dense stands of kelp deter urchin attack, formation of gaps can initiate rapid conversion of kelp forests to urchin barrens (Estes et al. 2009). Once established on open substrate, the urchins eliminate kelp recruitment and maintain open conditions by switching their behaviour from consumers of detrital fallout within forest to mobile herbivores when kelp is rare (Konar & Estes 2003). The system thus tends to exhibit non-linear dynamics between well developed kelp forest and urchin barrens, with unstable transitory intermediate states, depending on otter abundance (Estes & Duggins 1995; Steneck et al. 2002; Estes et al. 2010; Figures 4 & 5). Since kelp canopies provide habitat for predatory fish and mammals, large reductions in structural complexity of the forest can increase survival of resident prey and their larvae (Gaines & Roughgarden 1987). The abundance of urchins is regulated by several trophic levels of predators and the availability of alternative prey (Figure 3), resulting in highly complex ecosystem dynamics influenced by multiple processes and interactions that are intrinsic and extrinsic to the kelp forests (Springer 2003; Springer et al. 2008; Estes et al. 2009).

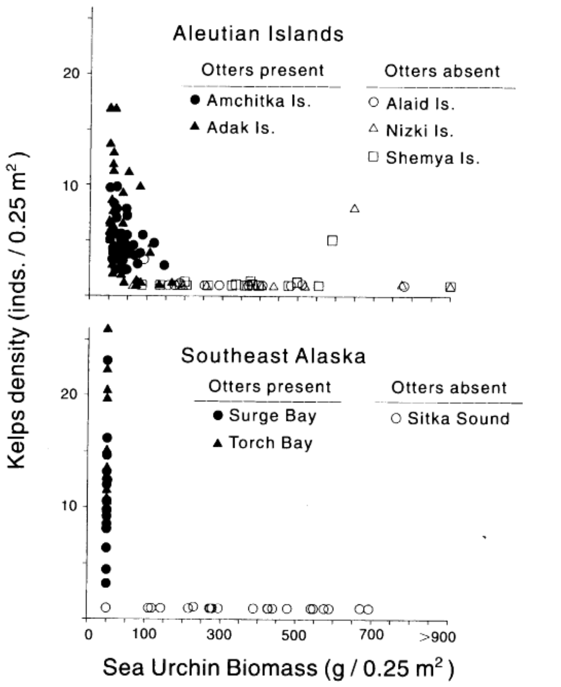

FIG. 7. Epibenthic kelp density (inds./0.25 m²) plotted against estimated sea urchin biomass (g/0.25 m²) for the Aleutian Islands and southeast Alaska. Points represent averages for sites within locations. Sea urchin biomass was estimated from samples of population density, size–frequency distribution, and the functional relation between test diameter and wet mass.

Figure S16. 4. Non-linear inverse relationship between kelp density and sea urchin abundance, dependent upon presence of sea otters (from Estes & Duggins 1995, see also Estes et al. 2010).

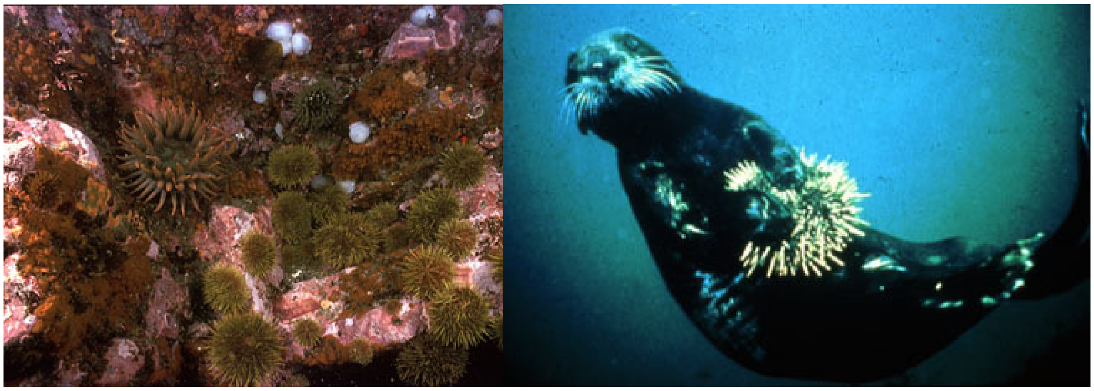

Figure S16. 5. Urchin barren (left, photo: David Duggins. cf. kelp forest in Fig. 1, and sea otter predation on urchins (right, photo: James A. Mattison III. Source: Stony Brook University, http://life.bio.sunysb.edu/marinebio/kelpforest.html).

Threatening processes

Widespread deforestation is the major threat to Alaskan kelp forests, as this eliminates or greatly simplifies the structural complexity that provides essential habitat for characteristic native biota of the ecosystem (see previous section). Although transient kelp-free patches are likely to be expressions of natural ecosystem turnover, there is evidence that over-exploitation of marine mammals and fish can initiate a trophic cascade that results in widespread conversion of kelp forest to urchin barrens (Estes et al. 2009). As sea otters are major predators of urchins, decline in otter populations can lead to explosion of urchin populations and subsequent elimination of kelp. Otter populations are in turn regulated by predators, notably killer whales, which are thought to have switched their diet in response

to depletion of great whale and piniped populations during the second half of the twentieth century, substantially increasing predator pressure on otter populations (Springer 2003; Springer et al. 2008).

Other threats include changes to ocean currents and temperatures, as concentrations of nutrients are inversely related to water temperature, and kelp forests are dependent for growth on cold waters with high nutrient availability. For example, nitrate concentrations decline by more than an order of magnitude as temperatures increase from 6 to14°C (Dayton et al. 1999). Thus, the kelp forests are likely to be susceptible to climate change that weakens cold water currents or increases frequency of thermal events. Oil spills also pose threats of long-lasting impacts over localised parts of the ecosystem distribution (Peterson et al. 2003).

Ecosystem collapse

For assessment of criteria A and B, the kelp forests are assumed to collapse when their distribution declines to zero and they are entirely replaced by urchin barrens. are found, with kelp density of zero, throughout the former range. Two variables were identified as potentially suitable for assessing disruption to biotic processes and interactions under criterion D: the density of kelp; and the abundance of sea otters, both having important trophic roles within the ecosystem. It was assumed that ecosystem collapse occurs when either of these measures declines to zero.

ASSESSMENT

Summary

Criterion	A	B	C	D	E	overall
subcriterion 1	DD	LC	NE	EN(EN-CR)	DD	EN(EN-CR)
subcriterion 2	DD	DD	NE	DD		
subcriterion 3	DD	LC	NE	EN(EN-CR)		

Criterion A

Although there is qualitative information to suggest that a substantial area of kelp forest to urchin barrens was converted to urchin barrens within the past 50 years, spatially explicit data for the recent and historic past were unavailable for this assessment. Similarly, no future projections were available for assessment. Consequently, the status of the ecosystem is Data Deficient under criteria A1, A2 and A3.

Criterion B

Extent of occurrence: A minimum convex polygon enclosing rocky reefs suitable for development of kelp forest along the Aleutian Islands and south Alaskan coast has an area of at least 1.5 million km^2. Given that the distribution may extend further east and south of this core area, the extent of occurrence of the ecosystem is likely to be larger than this estimate. Although there is evidence of continuing disruption to biotic interactions (Estes et al. 2009., the status of the ecosystem under criterion B1 is Least Concern because the estimated extent of occurrence exceeds the thresholds for all threatened categories.

Area of occurrence: Available distribution data are of insufficient resolution to estimate the number of occupied 10 km grid cells. The status of the ecosystem is therefore Data Deficient under criteria B2.

Number of locations: The most serious plausible threat to Giant Alaskan Kelp Forests is deforestation by urchins. The number of locations where urchin outbreaks occur independently is uncertain. Even though depletion of otter populations is widespread throughout the distribution of the kelp forests, this may not lead to urchin outbreaks in some cases (Tegner & Dayton 2000; Estes et al. 2010). Remnant otter colonies plausibly persist at locations in between the areas where they have been extirpated.

Furthermore, different conditions and ecological responses of urchins to otter extirpation occur in different parts of the range, possibly due to differing regional patterns in urchin recruitment (Estes & Duggins 1995; Estes et al. 2010). Consequently there are likely to be more than five locations and the status of the ecosystem is likely to be Least Concern under criteria B3.

Criterion C

Environmental degradation may occur through changes to ocean currents and temperatures (affecting nutrient availability), as well as oil spills. At this time suitable data sets to support an assessment of criterion C had not been located and hence the ecosystem is assigned to Not Evaluated.

Criterion D

The most serious disruption to biotic interactions occurs through trophic cascades involving sea otters, their predators and their prey, urchins that graze on kelp. Since densities of kelp are inversely related to densities of urchins and that phase shifts between forests and urchin barrens are related to a threshold abundance of otters (Estes et al. 2010), any of these variables is potentially suitable for assessing criterion D. Other potential response variables include the abundance of great whales and pinipeds, since these species are alternative prey for killer whales, which putatively switched their diet to sea otters when the larger mammals sequentially became scarce (Figure 3, Springer et al. 2003). Although good data are available on population changes in great whales and pinipeds, they were not used to assess the status of kelp forests because i. data on more proximal response variables are available and ii. the causal relationship linking great whales and pinipeds with otter abundance via killer whale predation is less certain than the link between otters, urchins and kelp (Springer et al. 2008). The assessment is therefore based on accurate, but localised surveys of kelp density and more extensive, but less precise surveys of sea otter populations.

Current decline: Repeat survey data of kelp density (Estes et al. 2010) and aerial surveys of otter populations were used to assess the severity and extent of current declines in biotic processes of the kelp forests. It was assumed that populations of otters, urchins and kelp were essentially stable prior to about 1986 based on evidence in Doroff et al. (2003).

Survey data for kelp stipe densities were available for 2-4 years between 1987 and 2000 from seven islands, based on 7-30 sites per island (Table 1 in Estes et al. 2010). It was assumed that the seven surveyed islands, scattered across the Aleutian chain, were representative of the full distribution of the ecosystem. Rates of change in kelp density were calculated for each island assuming an exponential model and varied from 29.5% decline per year to 15.4% increase per year. A weighted average across all sites, based on initial abundance indicated that kelp densities declined on average by 49.2% over 13 years between 1987 and 2000. Subsequent trends in kelp densities are uncertain, but declines are suspected to have continued beyond 2000, with later reversal at some sites. It was assumed that ecosystem collapse occurs when kelp density is close to zero across all sites, consistent with kelp replacement by urchin barrens throughout the distribution. Based on kelp densities, the relative severity of decline was therefore estimated to be approximately 50% averaged across the full extent (100%. of the distribution for the 13 years 1987-2000. Allowing for some decline prior to 1987 or after 2000 suggests that the declein in kelp density over the past 50 years was at least 50%.

Aerial survey data for sea otters were available for 55 islands in six island groups along the entire Aleutian chain (Table 1 in Doroff et al. 2003). All islands were surveyed in 1959, 1965, 1992 and 2000. It was assumed that the surveyed islands were representative of the full distribution of the ecosystem. Total otter counts were comparable in 1959 and 1965 (9507 and 9700, respectively). By 1992, there had been a net decline to 8048 sightings, although populations had increased on some islands and declined on others (Doroff et a. 2003). By 2000, otter populations had declined to low levels on all surveyed islands, with 2442 sightings recorded in total. Over the 40 year period 1959 - 2000, the otter

population declined by an estimated 74.3%. Doroff et al. (2003) suggest this may be a conservative estimate of decline since more animals may have eluded detection in the earlier surveys due to fast flight speeds. Skiff surveys at a subset of six islands produced very similar estimates of decline between 1992 and 2000 (Doroff et al. 2003). The fate of otter populations since 2000 is uncertain, although they are believed to have recovered at some sites (Estes et al. 2009). Assuming that ecosystem collapse occurs when otter populations are close to zero across all sites, produces an estimated relative severity of decline slightly larger than 75% across the full extent (100%. of the distribution. An alternative estimate of decline can be obtained from estimates of the total population reported by Doroff et al. (2003). In the mid 1980s (prior to recent decline. the total population was estimated to be 55,000 - 74,000 animals. By 2000, Doroff et al. (2003. estimated there were a total of 3,924 - 13,580 animals based on extrapolation from survey. The lower and upper bounds of otter population decline are therefore 100 × (55000-13580./55000 = 75.3% and 100 × (74000-3924./74000 = 94.7%, respectively, somewhat larger than the estimate obtained directly from the raw survey data. The upper bound may be extreme, as Doroff et al. (2003. caution that their 2000 estimate

Evidence from trends in kelp density and sea otter sightings suggest a decline in biotic function of 50-95% relative severity across 100% of the extent of the ecosystem. The upper bound of this range may overestimate the severity of decline because i) Doroff et al. (2003) caution that their 2000 survey may have underestimated the population due to detectability issues (although it is not known whether the estimates from the 1980s suffer similar bias) and ii) the calculations assume that otter and kelp populations have not recovered since 2000 when there is qualitative evidence of some recovery. The most likely status of the ecosystem under criterion D1 is therefore Endangered, although a status of Critically Endangered is possible.

Future decline: No projections are currently available for any of the biotic response variables. The status of the ecosystem is therefore Data Deficient under criterion D2.

Historic decline: Although historical data on kelp densities are lacking, some estimates of otter populations are available over the past century. During the nineteenth century extensive overexploitation by hunters eliminated sea otters from most of their historic range within the region, leaving only a few depleted remnant populations in the central Aleutians (Doroff et al. 2003). Harvesting ceased in 1911 when the species was protected by international treaty. This lead to recovery of the central Aleutian populations apparently to pre-harvest levels by about 1950 and by the 1980s, the species had recolonised many other islands and the total population was estimated to be 55,000 - 74,000 animals. A second decline followed, as described under 'Current decline'. Although, in the 1980s, some otter populations were thought to have reached carrying capacity, the overall population was still expected to be recovering to pre-harvest levels due to expected increases on more recently colonised islands (Doroff et al. 2003). It may therefore be reasonable to assume that the population in 1750 was comparable or slightly larger than its peak in the mid 1980s. Based on this assumption, the decline in otter populations throughout the distribution of the kelp forest was 75-95% since 1750 the status of the ecosystem is therefore Endangered (plausible range Endangered - Critically Endangered under criterion D3.

Criterion E

No quantitative analysis has been carried out to assess the risk of ecosystem collapse for Coastal sandstone upland swamps. The status of the ecosystem is therefore Data Deficient under criterion E.

REFERENCES

Anderson RJ, Carrick P, Levitt GJ, Share A. 1997. Holdfasts of adult kelp Ecklonia maxima provide refuges from grazing for recruitment of juvenile kelps. Marine Ecology Progress Series 159: 265–273.

Bernstein BB, Jung, N. 1980. Selective pressures and coevolution in a kelp canopy community in Southern California. Ecological Monographs 49: 335–355.

Bologna PAX, Steneck RS. 1993. Kelp beds as habitat for American lobster Homarus americanus. Marine Ecology Progress Series 100:127–134.

Chapman ARO. 1986. Age versus stage: an analysis of age- and size-specific mortality and reproduction in a population of Laminaria longicruris Pyl. Journal of Experimental Marine Biology and Ecology 97: 113–122.

Dayton PK. 1985. Ecology of kelp communities. Annual Review Ecology Systems 16: 215–245.

Dayton PK, Tegner MJ, Edwards PB, Riser KL. 1999. Temporal and spatial scales of kelp demography: the role of oceanography climate. Ecological Monographs 69: 219–250.

Domning DP. 1972. Steller's sea cow and the origin of North Pacific aboriginal whaling. Syesis 5: 187–189.

Doroff, AM, Estes JA, Tinker MT, Burn DM, Evans TJ. 2003. Sea otter population declines in the Aleutian archipelago. Journal of Mammalogy 84: 55–64.

Duggins DO. 1980. Kelp beds and sea otters: an experimental approach. Ecology 61: 447–453.

Duggins DO. 1983. Starfish predation and the creation of mosaic patterns in a kelp dominated community. Ecology 64: 1610– 1619.

Duggins DO, Eckman JE, Sewell AT. 1990. Ecology of understory kelp environments. II. Effects of kelps on recruitment of benthic invertebrates. Journal of Experimental Marine Biology and Ecology 143: 27–45.

Duggins DO, Simenstad CA, Estes JA. 1989. Magnification of secondary production by kelp detritus in coastal marine ecosystems. Science 245: 101–232.

Dunton KH, Schell DM. 1987. Dependence of consumers on macroalgal (Laminaria solidungula. carbon in an Arctic kelp community: delta-carbon-13 evidence. Marine Biology 93: 615–626.

Estes JA, Duggins DO. 1995. Sea otters and kelp forests in Alaska: generality and variation in a community ecological paradigm. Ecological Monographs 65, 75–100.

Estes JA, Steinberg PD. 1988. Predation, herbivory, and kelp evolution. Paleobiology 14: 19–36.

Estes JA, Doak DF, Springer AM, Williams TM. 2009. Causes and consequences of marine mammal population declines in southwest Alaska: a food-web perspective. Philosophical Transactions of the Royal Society B 364: 1647–1658.

Estes JA, Tinker MT, Bodkin JL. 2010. Using Ecological Function to Develop Recovery Criteria for Depleted Species: Sea Otters and Kelp Forests in the Aleutian Archipelago. Conservation Biology 24: 852–860.

Gaines SD, Roughgarden J. 1987. Fish in offshore kelp forests affect recruitment to intertidal barnacle populations. Science 235: 479–481.

Harrold C, Reed DC. 1985 Food availability, sea urchin grazing, and kelp forest community structure. Ecology 66, 1160–1169.

Konar B, Estes JA. 2003 The stability of boundary regions between kelp beds and deforested areas. Ecology 84, 174–185.

Levin PS. 1994. Fine-scale temporal variation in recruitment of a temperate demersal fish: the importance of settlement versus post-settlement loss. Oecologia 97: 124–133.

Linley EAS, Newell RC, Bosma SA. 1981. Hetertrophic utilization of mucilage released during fragmentation of kelp (Ecklonia maxima and Laminaria pallida. development of microbial communities associated with the degradation of kelp mucilage. Marine Ecology Progress Series 4: 31–41.

Mann KH. 2000. Ecology of Coastal Waters, with Implications for Management. Volume 2. Blackwell, Oxford.

Peterson CH, Rice SD, Short JW, Esler D, Bodkin JL, Ballachey BE, Irons DB. 2003. Long-Term Ecosystem Response to the Exxon Valdez Oil Spill. Science 302: 2082-2086.

Raffaelli D, Hawkins S. 1996. Intertidal Ecology. London, UK: Chapman & Hall.

Reed DC, Foster MS. 1984. The effects of canopy shading on algal recruitment and growth in a giant kelp forest. Ecology 65: 937–948.

Reisewitz SE, Estes JA, Simenstad CA. 2005 Indirect food web interactions: sea otters and kelp forest fishes in the Aleutian archipelago. Oecologia 146, 623–631.

Santelices B, Ojeda FP. 1984. Population dynamics of coastal forests of Macrocystis pyrifera in Puerto Toro, Isla Navarino, Southern Chile. Marine Ecology Progress Series 14: 175–183.

Springer AM. 2003 Sequential megafaunal collapse in the North Pacific Ocean: an ongoing legacy of industrial whaling? Proceedings of the National Academy of Science USA 100, 12 223–12 228.

Springer AM, Estes JA, van Vliet GB, Williams TM, Doak DF, Danner EM, Pfister B. 2008 Mammal-eating killer whales, industrial whaling, and the sequential megafaunal collapse in the North Pacific Ocean: a reply to the critics of Springer et al. 2003. Marine Mammal Science 24: 414–442.

Steneck RS, Watling LE. 1982. Feeding capabilities and limitations of herbivorous molluscs: a functional group approach. Marine Biology 68: 299–319.

Steneck RS, Graham MH, Bourque BJ, Corbett D, Erlandson JM, Estes JA, Tegner MJ. 2002 Kelp forest ecosystem: biodiversity, stability, resilience and their future. Environ. Conserv. 29, 436–459.

Tegner MJ, Dayton PK, Edwards PB, Riser KL. 1997. Large-scale, low-frequency oceanographic effects on kelp forest succession: a tale of two cohorts. Marine Ecology Progress Series 146: 117–134.

Tegner MJ, Dayton PK. 2000. Ecosystem effects of fishing in kelp forest communities. ICES Journal of Marine Science 57: 576–589.

17 CARIBBEAN CORAL REEFS

contributed by David Keith[1] and Mark Spalding[2]
[1]Australian Wetlands and Rivers Centre, University of New South Wales and NSW Office of Environment & Heritage.
[2]The Nature Conservancy and Conservation Science Group, Department of Zoology University of Cambridge, Cambridge, England.

CLASSIFICATION

International: Coral reefs mainly fall within two major biogeographic regions: the tropical Indo-Pacific realms and the tropical Atlantic. Almost all of the latter are found within the Tropical Northwestern Atlantic ecological province of Spalding et al. (2007), sometimes referred to as the Wider Caribbean, and occur within all nine of its ecoregions (Burke and Maidens 2004). The only other coral reefs in the Tropical Atlantic realm are found off the coast of Brazil, and although these have considerable biogeographic affinities to the wider Caribbean region they are also marked by lower diversity, distinct faunal communities and somewhat different reef morphology (Leâo et al. 2003).
IUCN Habitats Classification Scheme (Version 3.0): 9. Marine Neritic / 9.8 Coral Reef

ECOSYSTEM DESCRIPTION

Characteristic native biota

Coral reefs are physical structures that have been built up, and continue to grow over decadal time-scales, as a result of the accumulation of calcium carbonate laid down by hermatypic corals and other organisms (Spalding 2001). The Caribbean coral reefs are primarily fringing reefs and bank barrier reefs (Fiure. 1) separated from island and mainland shorelines by reef flats, shallow waters or slightly deeper lagoons (Alevizon 2010). There are only a very small number of oceanic reefs and atolls surrounded by very deep water. Most of these reefs lack an algal ridge on the seaward crest, unlike many reefs of the Indo-Pacific region. Instead windward crests are, or were, dominated by *Acropora palmata*, a large branching coral (Bruckner & Bruckner 2006; Alevizon 2010), or in its absence, octocorals, sponges and algae (Dudgeon et al. 2010).

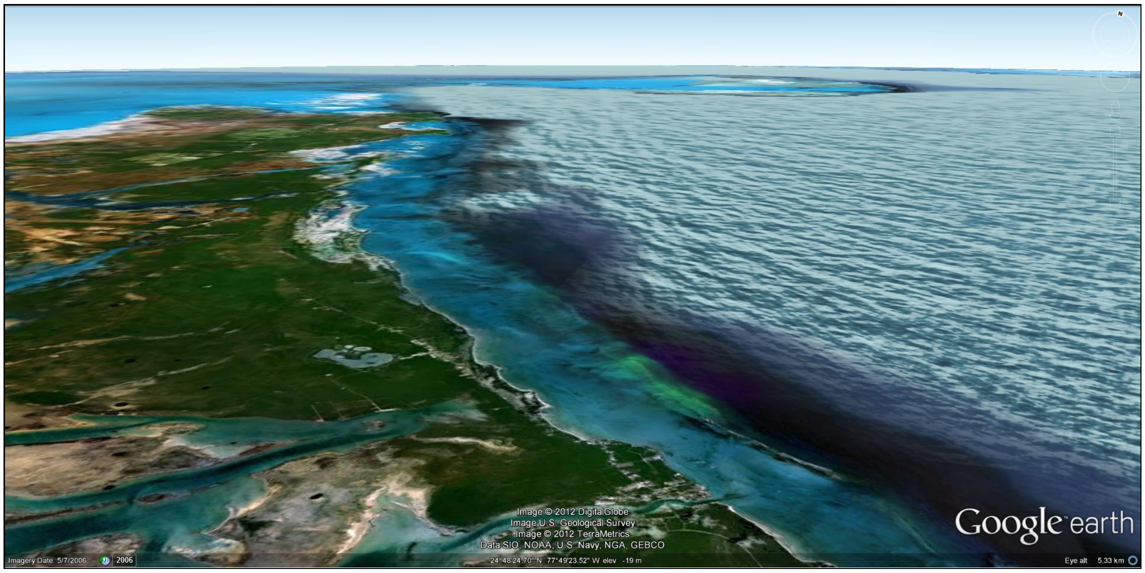

Figure S17. 1. Fringing coral reef off the east coast of The Bahamas (Google Earth).

Caribbean reefs include about 65 - 75 species of hermatypic (reef-building) coral (mostly Scleractinia). Many of these are endemic to the region due to the long isolation of the west Atlantic from the east

Pacific Ocean since the formation of the Panamanian isthmus. Widespread hermatypic genera include *Acropora*, *Montastrea Porites Agaricia Diploria, Colpophylia, Meandrina, Mycetophyllia, Dendrogyra* as well as the non-Scleractinian fire corals *Millepora* spp. Many of these corals have distinct growth forms – notably branching, massive, sheet-like and leafy forms. Others show remarkable variation even within the same species (Goreau 1959; Spalding 2004; Alevizon 2010; CARMABI 2012; CARMABI Foundation 2012).

Different coral species are associated with different water conditions (Goreau & Goreau 1973). For example, *Acropora palmata* is primarily on the reef crest and fore reef at 0-5 m depth, while *A. cervicornis* occurs at depths of 5-15 m on exposed reefs, as well as shallower areas at 1-10 m depth on more protected reefs , where it may co-occur with *Montastraea annularis* (Bruckner & Bruckner 2006). Caribbean coral reefs are also distinguished by an abundance of octocorals (e.g. *Eunicea, Gorgonia, Plexaura, Muricea* spp.) and sponges that grow from the hard coral base and contribute to the diversity and structural complexity of the reef. Other prominent invertebrate groups include crustacea, mollusca and holothuria, including *Diadema antillarum,* a key algal herbivore.

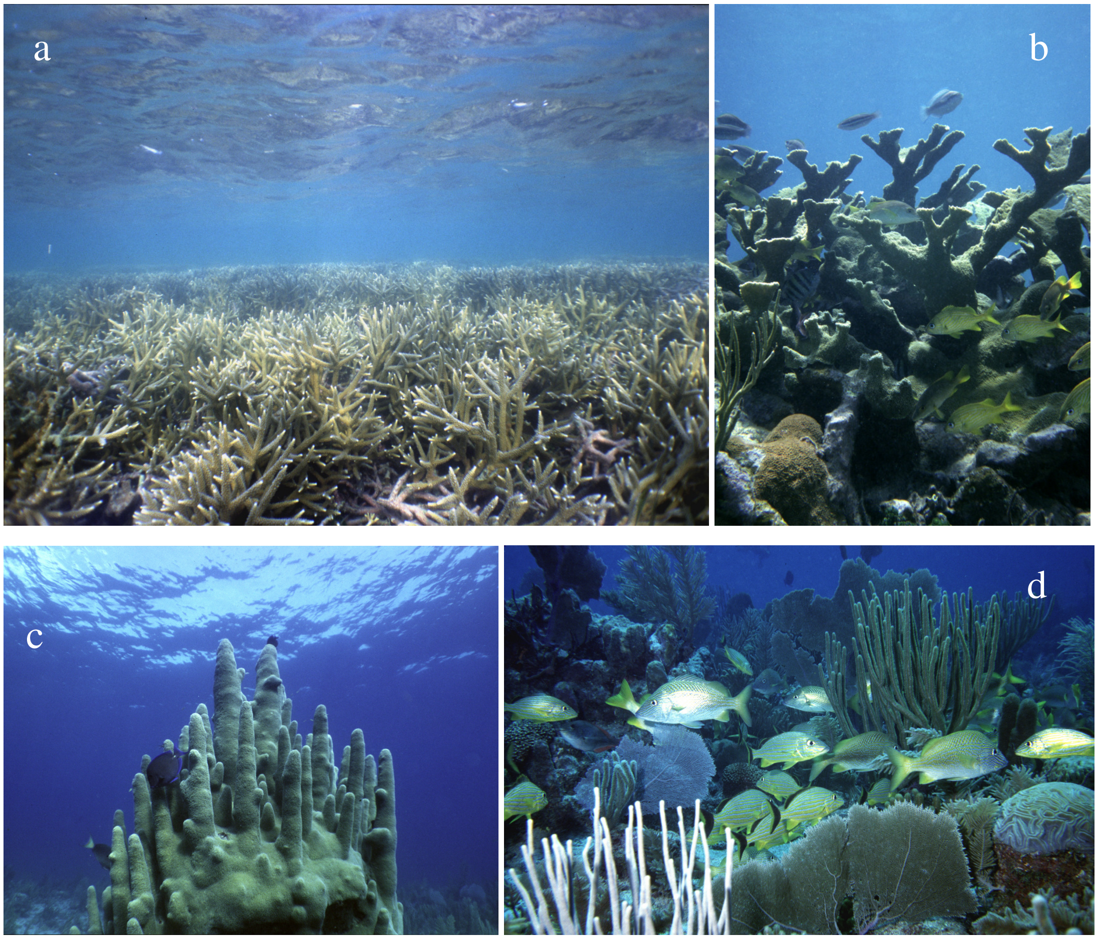

Figure S17. 2. Caribbean coral reef with (a) staghorn coral, *Diploria strigosa,* (b) Elkhorn coral *Acropora palmata,* (c) Pillar coral *Dendrogyra cylindrus*, and (d) soft corals (Photos: Mark Spalding).

Approximately 500 - 700 species of fish are associated with Caribbean coral reefs (Figure 2). These include grunts (Haemulidae), snappers (Lutjanidae) groupers (Serranidae), angelfish (Pomacanthidae), butterflyfish (Chaetodontidae), damselfish (Pomacentridae), jawfish (*Opistognanthus* spp.), parrotfish (Scaridae), wrasses (Labridae) and surgeonfish (Acanthuridae). About 80% and coral and fish species occur within the Bahamas portion of the ecosystem distribution. The Caribbean Monk Seal (*Monachus*

tropicalis) was an apex predator of fish in Caribbean reef systems until its extinction in the mid-late twentieth century due to overexploitation (Le Boef et al. 1986; Jackson et al. 2001). Marine turtles, notably green and hawksbill are still found, but have probably lost their role as keystone species due to overexploitation (Jackson et al, 1997; McClenachan, 2006).

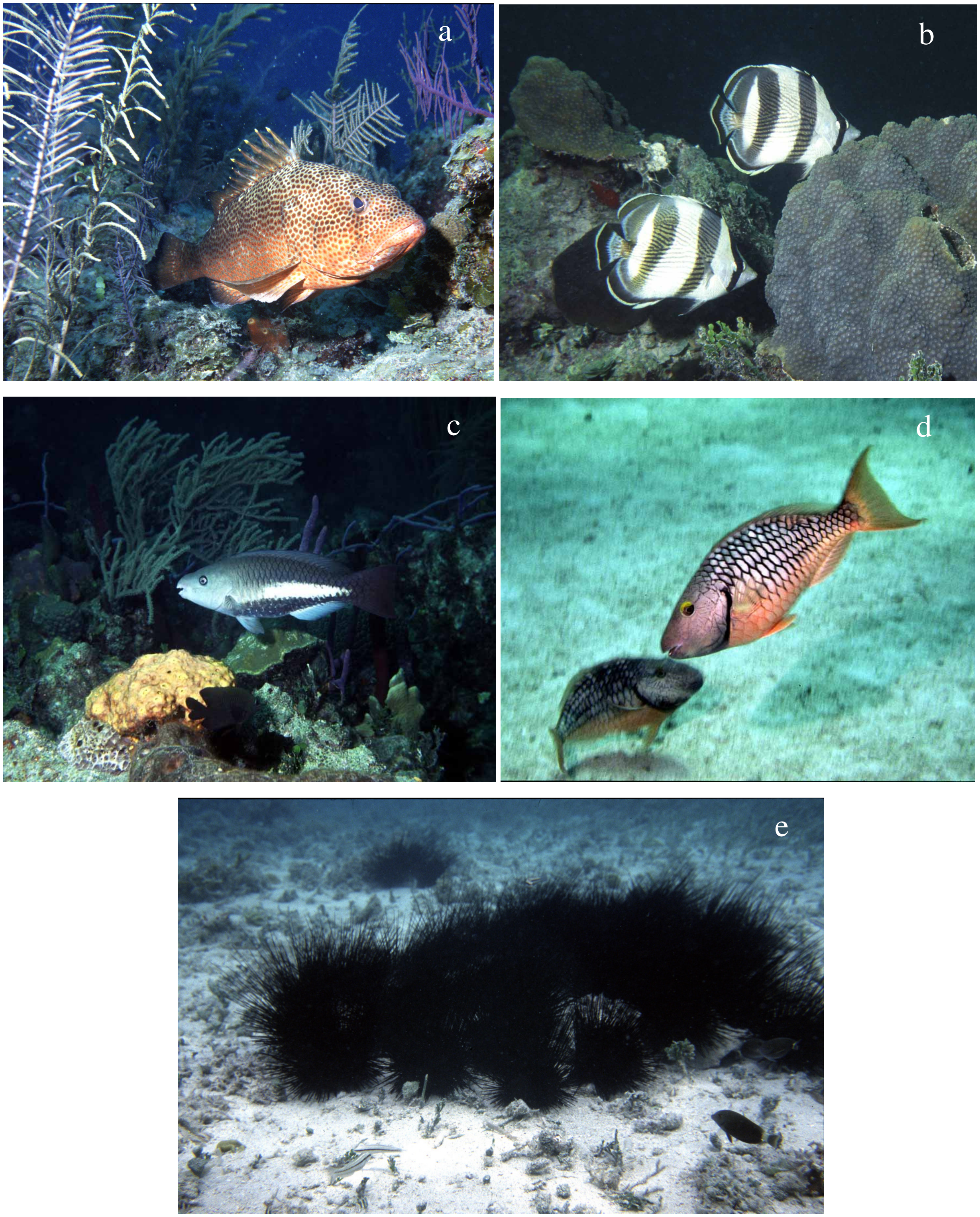

Figure S17. 3. Characteristic fish of Caribbean coral reefs: (a) Red Hind, (b) Banded Butterflyfish, (c) Queen Parrotfish, and (d) Stoplight parrotfish; and (e) sea urhin *Diadema* sp. (Photos: Mark Spalding).

Abiotic environment

Caribbean coral reefs occur in warm, shallow (rarely >60 m depth), clear, relatively nutrient-poor, open coastal waters, where sea temperatures vary between 17-34°C and salinity is 30-38 ppt. By contrast turbid coastal waters, estuaries, deltas and deep oceans are not suitable for coral reef development, and there are no reefs, for example, in the vicinity of the Mississippi delta.

Distribution

The Tropical Northwestern Atlantic province (Spalding et al. 2007) stretches between latitudes of about 10–30° N and longitudes of about 60–95° W, including the Caribbean Sea, Gulf of Mexico, Florida Keys, The Bahamas, Cuba, Antilles and Venezuelan coast, with an outlier at Bermuda (Figure 4). The latest global reef map estimates some 26,000 km^2 of reefs in the Caribbean, or about 10% of the global total (Burke et al 2011).

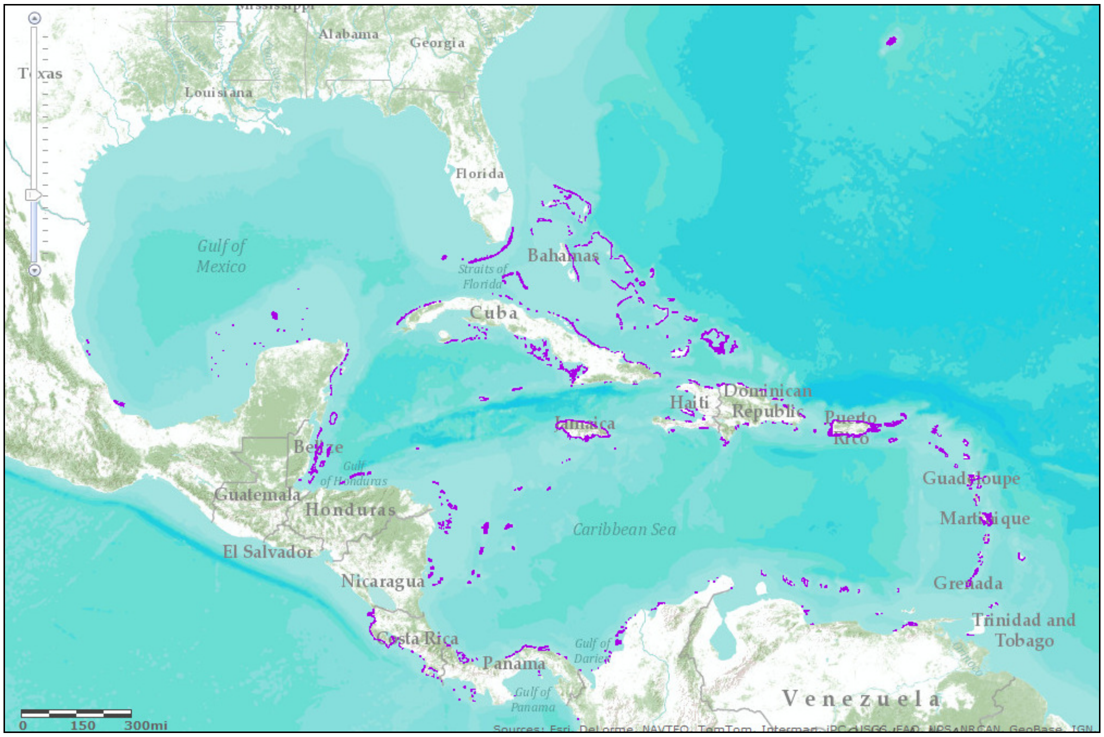

Figure S17. 4. Distribution of Caribbean coral reefs (excluding reefs off the southeast coast of Costa Rica and Panama). Source: Global distribution of coral reefs, United Nations Environment Program - World Conservation Monitoring Centre (Andréfouët et al. 2005) [http://www.arcgis.com/home/webmap/viewer.html?useExisting=1].

Key processes and interactions

Most reef corals are colonial organisms, with individual coral polyps forming a communal skeleton. Most species form endosymbiotic relationships with dinoflagellates (*Symbiodinium* spp.), which assimilate solar energy and nutrients, providing more than 95% of the metabolic requirements of the coral host. Scleractinian corals develop their skeletons by extracting dissolved carbonate ions from seawater and depositing it as aragonite crystals. The obligate endosymbiosis and calcification processes that underpin the survival, growth and reproduction of individual corals, are highly dependent on environmental conditions including ambient temperature, tubidity, pH and carbonate concentration of seawater (Hoegh-Guldberg 1999; Hoegh-Guldberg et al. 2007).

Individual coral polyps reproduce asexually to enable colony growth. While asexual reproduction is important for growth and regeneration of individual colonies, establishment of new colonies relies on sexual reproduction, which occurs by simultaneous spawning (release of gametes) over one to several

nights per year around a full moon. The fertilised zygotes settle on a range of hard substrates including red (coralline) algae and other corals, and once established, undergo asexual reproduction. Colonies of different species co-occurring within a reef community have different characteristic growth forms, creating a complex spatial architecture (rugosity) that provides shelter and breeding sites for a high diversity of fish and a wide range of marine invertebrates including sponges, crustacea, polychaete worms, holothurians, etc. (Alvarez-Filip et al. 2009).

Many Caribbean coral reefs are impacted by irregular disturbance from storms and outbreaks of disease or predators, which interrupt more prolonged periods of reef building (e.g. Woodley et al. 1981; Aronson & Precht 2001). The recovery of reefs after these events often involves a transitory phase of algal dominance which, under other conditions is kept in check by herbivores, notably parrotfish and the urchin *Diadema antillarum*. The algal phase may be prolonged or established more permanently by local and regional factors that increase growth of algae relative to that of coral. These include eutrophication due to increased runoff from developed coastal catchments and reduced herbivory due to overfishing or herbivore diseases (Fabricius 2005, Hughes, 1994; Hughes et al 2007). Diseases have also had a tremendous impact on certain Caribbean corals, including two of the major reef-building corals (*Acropora palmata* and *A. cervicornis*) and while susceptibility to disease does appear to be heightened by other stressors, the widespread prevalence of disease, even in areas where other perturbations are minimal suggests that this is an independent impact which is also affecting whole ecosystem persistence in the region (Sutherland et al. 2011; Weil and Rogers 2011).

In addition to the above factors, reef-scale dynamics are influenced by interactions with global climate through three main processes (Figure 5). Firstly, rising temperatures, or more specifically increased duration and intensity of high-temperature anomalies, increase the frequency of coral dieback events. These 'bleaching' events are caused by disintegration of obligatory endosymbiosis between corals and dinoflagellates (Hoegh-Guldberg 1999; van Oppen and Lough 2008) and a bleaching response is typically triggered when temperatures exceed summer maxima by 1-2°C for 3-4 weeks. Consequently, for analysis of bleaching events, thermal anomalies are defined by a 1°C elevated temperature threshold. Under mild or short periods of thermal stress, corals may survive and recover their symbiont but typically show reduced calcification, growth and fecundity for an extended period. They may also be more susceptible to disease (Bruno et al. 2007). Bleaching and mortality become progressively more severe as thermal anomalies intensify and lengthen (Hoegh-Guldberg 1999), although there is some evidence that past exposure to thermal anomalies may confer partial resilience to subsequent events (Donner & Potere 2007; Ateweberhan & McClanahan 2010; Guest et al. 2012). Secondly, as atmospheric concentrations of CO_2 increase, approximately 25% is taken up by the ocean and reacts with water to reduce pH. As ocean acidification progresses, the seawater concentration of carbonate ions is reduced as they react with free hydrogen ions, and this in turn reduces aragonite formation, calcification and growth rates of coral and coralline algae (Hoegh-Guldberg et al. 2007). A third potential mechanism affecting the dynamics of Caribbean coral reefs is through changes in frequencies of storms (Woodley et al. 1981; /Robbins et al. 2011), as this influences the balance between rates of reef depletion and building, as well as turbidity of coastal waters.

Overall, some of the above processes affect the frequency and severity of coral mortality events on reefs, while others affect rates of growth and recruitment, and hence rates of reef regeneration and recolonisation. Several of these processes may interact at a range of scales to mediate reef persistence and rates of expansion or decline (Wilson et al. 2006). For example, there is evidence that warm sea temperature anomalies can drive disease outbreaks where coral cover is high (Bruno et al. 2007). A cause-effect process model proposed by Hoegh-Guldberg et al. (2007) shows the mechanisms of reef dynamics in relation to warming, acidification and some of the regional and local-scale processes discussed above (Figure 5).

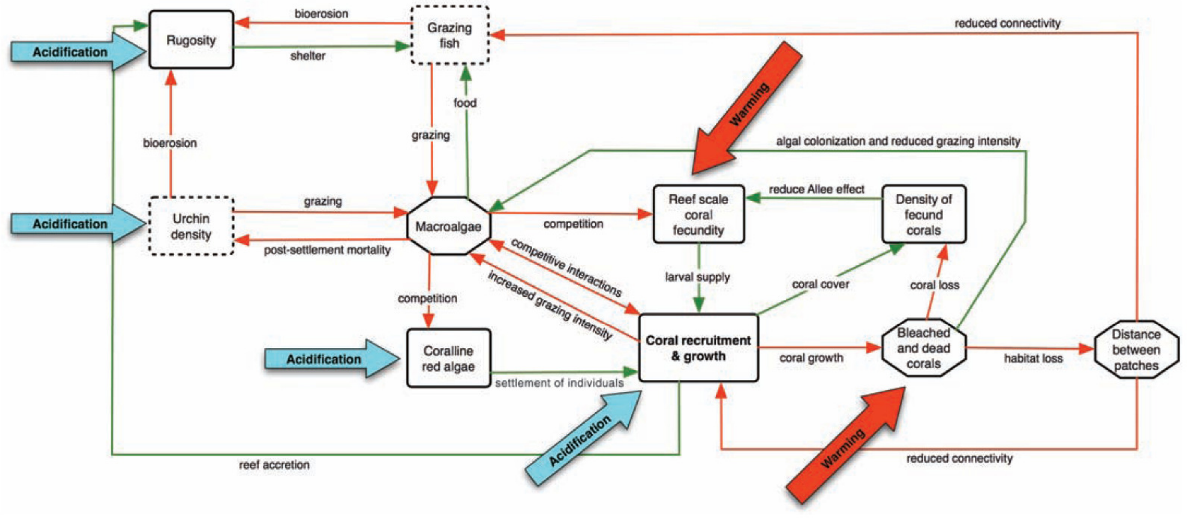

Fig. 3. Ecological feedback processes on a coral reef showing pathways of disturbance caused by climate change. Impact points associated with ocean acidification (e.g., reduced reef rugosity, coralline algae) are indicated by the blue arrows, and impact points from global warming (e.g., bleached and dead corals) by the red arrows. Boxes joined by red arrows denote that the first factor has a negative (decreasing) influence on the box indicated. Green arrows denote positive (increasing) relationships. Over time, the levels of factors in hexagonal boxes will increase, whereas those in rectangular boxes will decline. Boxes with dashed lines are amenable to local management intervention.

Figure S17. 5. Cause-effect process model for coral reefs with a focus on warming and acidification (from Hoegh-Guldberg et al. 2007). Other important processes include sedimentation, eutrophication and overfishing (see text)

Threatening processes

The principal threats to Caribbean coral reefs include diseases, pollution, bleaching, ocean acidification, increased storm frequencies and overfishing. These factors have compounding effects on reef dynamics, for example; as pollution reduces the ability of reefs to recover from disease epidemics or storm damage; or as severe storms increase sedimentation and turbidity from runoff.

Caribbean coral reefs are vulnerable to sedimentation and eutrophication of coastal waters from onshore agricultural, urban and industrial land uses (Rogers 1990; Boyer & Jones 2002; Fabricius 2005). Many are fringing reefs and hence exposed to runoff from coastlines, of which some have very high human population densities (e.g. Florida, Haiti). By 2010 43 million lived within 30 km of a coral reef (Burke et al. 2011). Consequently, 25% of Caribbean coral reefs are currently threatened by coastal development, including sewage discharge, urban runoff, construction and tourist development. Overuse associated with heavy tourist activity within the Caribbean region also poses threats from physical damage incidental to recreational boating, fishing, diving and snorkeling, in addition to degradation of water quality.

Diseases of coral and algal herbivores have had a major impact on Caribbean coral reefs in recent decades, with an outbreak of White Band Disease associated with a precipitous decline in abundance of *Acropora palmata* in the 1980s, and few reefs showing evidence of subsequent recovery (Aronson & Precht 2001). A subsequent outbreak of Yellow Band Disease substantially reduced the abundance of *Montastraea* spp. from the mid 1990s, especially across the southern Caribbean (Gil-Agudelo et al. 2004; Bruckner & Bruckner 2006). Diseases may also threaten coral reefs indirectly. In 1983 and 1984, for example, there was Caribbean-wide die-off of the sea urchin *Diadema antillarum*. Prior to this date, overharvesting of many herbivorous fish had left this one urchin species as the only remaining significant algal grazer on many reefs (Lessios 1988). A substantial increase in algal abundance followed. While the algal response was not sustained in all areas, the urchin populations have not recovered (Schutte et al. 2010).

Extensive bleaching events occurred in Caribbean coral reefs following sea temperature anomalies in 1998 (Aronson et al. 2000) and in late 2005 (Donner et al. 2007; Wilkinson & Souter 2008), especially in the southern portion of the ecosystem distribution. Other large bleaching events were recorded in 1987, 1990 and 1995 (McWilliams et al. 2005). The frequency and severity of bleaching events is projected to increase under climate change (Hoegh-Guldberg et al. 2007).

Periodic hurricanes cause substantial physical damage to Caribbean coral reefs (e.g. Stoddart 1963; Woodley et al. 1981). There is some evidence that the frequency of hurricanes may have increased since 1995 (Robbins et al. 2011). Although this is consistent with climate change projections for rising sea surface temperatures, the trend was primarily due to an increase in the number of weak, short-duration storms (Landsea et al. 2010) and it is difficult to disentangle climate-related trends from multi-decade variability (Knutson et al. 2008; Bender et al. 2010). Although trends in hurricane frequency and intensity are uncertain, reductions in inter-storm recovery time, if they occur, may compound the impacts of other threats (Hoegh-Guldberg et al. 2007).

Ocean acidification has been shown empirically to reduce calcification rates of corals and coralline algae (Langdon et al. 2003), although the precise geochemical mechanism is uncertain (Kleypas & Yates 2009). The effects may be expressed as reduced rates of coral growth (linear extension) or reduced density of coral skeletons (Cooper et al. 2008). In the Caribbean region, aragonite saturation, which is related to calcification rates, is projected to decline from 4.0 - 4.2 under pre-industrial CO_2 levels (280ppm) to 3.0 - 3.2 under elevated CO_2 levels of 550 ppm (Hoegh-Guldberg et al. 2007).

Trophic cascades initiated by overfishing are likely responsible for some of the observed changes in Caribbean reef fish assemblages (Dustan 1999; Paddack et al. 2009). Overexploitation of herbivorous fish also increases the competitive advantage of algae over coral. Historic declines in large herbivorous fish in the Caribbean lead to dependence on urchins for control of algal dominance, and may have been precursors of changes in coral and algal communities when disease caused collapse of urchin populations (Jackson et al. 2001). Burke et al (2011) estimated that almost 70% of Caribbean coral reefs are currently threatened by overfishing. Extinction of the Caribbean Monk seal and loss of its trophic function is also thought to have had a dramatic effect on reef fish assemblages (McClenachan and Cooper 2008).

Ecosystem collapse

For criteria A and B, ecosystem collapse was assumed to occur when the mapped distribution of Caribbean coral reefs declined to zero. As coral is the main structural element of the reef, we identified coral cover as a suitable variable for assessing disruption to biotic processes and interactions under criterion D. We assumed that collapse will occur when live coral cover declines to 0-5% throughout the ecosystem.

ASSESSMENT

Summary

Criterion	A	B	C	D	E	overall
subcriterion 1	DD	LC	NE	EN(VU-CR)	DD	EN(EN-CR)
subcriterion 2	DD	LC	NE	DD		
subcriterion 3	DD	LC	NE	EN		

Criterion A

Current decline:

One widely cited statistic suggests that some 20% of coral reefs have been lost to human impacts in recent years (Wilkinson 2004), however there are no robust statistics to justify this estimate. One of the greatest challenges to measuring declines in terms of spatial extent arises from the definition of the

ecosystem – most authors agree that reefs are large physical structures with a living veneer including an important component of live coral cover, but that reefs are long-lived, slow-growing and subject to natural fluctuations. By definition, then, the loss of a coral reef is not clearly measurable, and this is exacerbated by most mapping efforts (e.g. Andréfouët et al. 2006) which tend to focus on the physical structures which are built by corals, but which remain robust following coral death.

Improvements in remote sensing technology are increasing the possibility of measuring fine resolution changes in reef habitat, and Palandro et al (2008) provide a useful, field-tested, example of change in the Florida Keys. In a number of test locations they showed that coral dominated substrate declined from 19% in 1984 to 7.6% in 2002, a reduction of 61% (3.4%/year) over 18 years. Such changes are in many ways more relevant to Criterion A than the more commonly measured coral cover (see below), but most definitions would include the other habitat classes they measured (bare sand, seagrass and non-Scleractinian hardbottom) as part of the reef ecosystem and hence that these data reflect a shift of dominance rather than loss of reef. The authors also caution against generalising their results to other regions, which so far lack similar analyses (Palandro et al. 2008).

The status of the ecosystem is therefore **Data Deficient** under criterion A1. While these data are unsuitable to support overall estimates of change in distribution of the ecosystem, changes in coral cover are relevant to biotic interactions within reefs and are assessed under criterion C below.

Future decline: No projections are currently available for future reef distribution. Consequently, the status of the ecosystem is **Data Deficient** under criterion A3.

Historical decline: Estimates of changes in reef distribution exist only for localised areas (e.g. Duerdin 1901), and given their limited temporal resolution it is difficult to distinguish declines from natural fluctuations in reef distribution. The status of the ecosystem is therefore **Data Deficient** under criterion A3.

Criterion B

Spatial data from the Millenium coral reef mapping project (Andréfouët et al. 2006) were used to assess the distribution of Caribbean coral reefs under criterion B. The total mapped area of coral in the Caribbean is estimated to be 21,000 to 26,000 km^2 (Andréfouët et al. 2006; Burke et al. 2011).

Extent of occurrence: A minimum convex polygon enclosing mapped coral reefs of the Caribbean region has an area of at least 7.37 million km^2. Even with the outlying reefs of Bermuda excluded, the estimated extent of occurrence greatly exceeds the thresholds for threatened status (c. 6 million km^2). The status of the ecosystem under criterion B1 is therefore **Least Concern**.

Area of occurrence: Caribbean coral reefs occupy approximately 10,000 10 × 10 km grid cells, including more than 2,000 that contain more than 1 km^2 of reef. Once again, then, the status of the ecosystem is **Least Concern** under criteria B2 because the estimated area of occurrence greatly exceeds the thresholds for threatened status.

Number of locations: There are likely to be many thousands of individual reefs and locally interacting reef systems across the Caribbean. Based on recent past declines, these reefs are threatened by a suite of threats operating over a range of scales from local to global. Local impacts such as overfishing and pollution occur independently on individual or small-scale groups of reefs. By contrast disease outbreaks and bleaching events are widely occurring, and it is difficult to define or determine how independent such events may be across the region. Although these processes have generated strong region-wide declines in coral cover, there is substantial variation between different reefs with some maintaining stable coral cover or undergoing only minor declines over the same period that other undergo large declines (Schutte et al. 2010) and this is almost certainly linked to the interaction of local modifiers. It is thus impossible to determine any exact number of independent locations at which reefs

may be considered to be impacted from these combined stressors, although it is clearly much greater than five.

Future global change, both from warming and from ocean acidification (Kleypas and Yates 2009) is likely to increase the relative importance of regional threats to a level where they drive ecosystem collapse independently of more localised threats. At this point it may be relevant to consider these processes alone as the 'most serious plausible threats', and because of their broad scale of impact it may become relevant to consider the much broader spatial scale of their operation as defining much larger locations of threat. Future scenarios for ocean acidification, for example, suggest that there will be a strong cline of change in pH across the wider Caribbean, with more dramatic impacts along the coast of Central America, and much lower rates of change in the Greater Antilles (Cao and Caldeira 2008; Burke and others 2011). Under this interpretation, the ecosystem possibly occupies one to three locations, with reefs in the central portion of the distribution likely to maintain higher aragonite saturation for longer than the southern Caribbean, Gulf and Florida-Bahamas areas. Although aragonite saturation will decline markedly as atmospheric CO_2 increases, this process is projected to occur over the next 50-100 years, and the resulting declines in coral reefs may involve further lags of uncertain duration. As a consequence of this process, it is therefore doubtful that the ecosystem is capable of collapse or becoming Critically Endangered within a very short time period (e.g. the next 20 years). The status of the ecosystem is therefore likely to be **Least Concern** under criteria B3.

Criterion C

Environmental degradation of Caribbean coral reefs may occur through sedimentation and pollution (e.g. eutrophication), changes in the frequency and severity of ocean thermal anomalies associated with bleaching events and changes in ocean aragonite saturation due to acidification (Figure 5). All three processes were examined in the application of criterion C.

Sedimentation and pollution are generally localised and have only been quantified at a few locations (e.g. Rogers 1990; Fabricius 2005). However, an extensive spatial analysis of threats posed by coastal development, catchment-based sedimentation and pollution, marine-based pollution and damage, and overfishing has been carried out under the Reefs At Risk project (Burke and Maidens 2004; Burke et al. 2011). For each mapped grid cell (500 × 500 m) across the Caribbean reef ecosystem, each of these threats was ranked high, medium or low based on a number of spatial metrics. The ranks were integrated into an overall local threat index, "integrated local threat", by summing ordinal scores assigned to the threat levels (high, medium, low) across all four local threat types within each cell (Burke et al. 2011). These were further integrated with a thermal stress threat index to produce "integrated local threat plus thermal stress index". The analysis suggests that 92% of Caribbean coral reef extent is under at least a medium level of threat, 55% is under at least a high level of threat, while about 25% is under a very high level of threat (Burke et al. 2011). Changes in the extent and intensity of these threats were also assessed over a 10-year period 2001 - 2011 (Burke et al. 2011) , however the assessment is limited by the aggregated construction of the index and this limited period of assessment.

Increasing summer sea surface temperature anomalies are a major degradation process influencing coral reef dynamics (Figure 5). Remote monitoring of sea surface temperatures has recently been established in the Caribbean (Hayes & Goreau 2008; Cerdeira-Estrada & López-Saldaña 2011). Mean (±se) sea surface temperatures (SST) at reef locations across the Caribbean increased by 0.66±0.06°C between 1982 and 2003, with the western Caribbean warming more rapidly than the east (based on mean of regressions in Hayes & Goreau 2008). Summer anomalies in SST, which are more closely related to bleaching events than mean SSTs, also increased in the region during 1983 to 2000 relative to a 1961-1990 base period (McWilliams et al. 2005). Burke et al. (2011) reported that 55% of Caribbean coral reefs were located in areas where water temperatures were warm enough to cause bleaching on at least one occasion since 1998. A further analysis of thermal stress anomalies related to bleaching in the Caribbean between 1985 and 2005 showed that maximum annual numbers of anomalies occurred in 2005 in the eastern Caribbean and in 1998 in the west (Selig et al. 2010). Based on sea surface

temperature data and bleaching records averaged across all sampled Caribbean sites, McWilliams et al. (2005) showed that an increase of 0.1°C in summer SST anomalies was associated with 35% and 42% increases in the geographic extent and intensity of coral bleaching, respectively. By extrapolation of their regression models, they predicted maximum bleaching extent (i.e., 100% of coral-bearing cells) and maximum bleaching intensity (100% of coral colonies) when regional SST anomalies reach 0.97–0.98°C and 0.80–0.85°C, respectively (McWilliams et al. 2005). These changes in SST anomalies are slightly below the most conservative forecasted temperature increases projected to occur between 1990 and 2100 (McClean & Tysban 2001). There is some limited evidence of partial adaptation by at least some corals to rising temperatures (Maynard et al. 2008; Jones et al. 2008; LaJeunesse et al. 2009; Ateweberhan & McClanahan 2010; Donner 2011), and thus although there is a consensus that this will not be sufficient for reefs to withstand the projected future change it makes it even more challenging to estimate what levels may become critical to continued coral survival.

Ocean acidification is a salient process influencing ecosystem dynamics (Figure 5), and effects are related to aragonite saturation. Aragonite saturation has been monitored at four stations in the Greater Caribbean region since 1988 and show a constant rate of decline reflecting acidification over that period (Friedrich et al. 2012). In addition, a coupled carbon cycle - climate model enables projection of aragonite saturation back to pre-industrial times and forward to year 2100 (Friedrich et al. 2012). Simulations suggest that anthropogenic effects on ocean acidification became detectable around 1850-1875 and after initial acceleration, became constant by about the mid 1960s. However, the noise around acidification impact is likely to be large at species, community and regional levels due to compensatory responses and non-linear relationships (Ries et al. 2010; Anthony et al. 2011).

The data from all three proxies and their associated assumptions for environmental degradation are currently under review. At the time of writing, the status of the ecosystem under criteria C1, C2 and C3 was assigned to **Not Evaluated**.

Criterion D

Based on salient processes within the ecosystem, coral recruitment and growth are central to ecosystem dynamics (Figure 5). Consequently, coral cover is widely regarded as a critical measure of habitat loss and degradation, given the key role that corals play in facilitating the entire reef ecosystem (Bruno & Selig 2007). More broadly, disruption of biotic interactions within Caribbean coral reefs are expressed as changes in coral cover, reef architecture and loss of fish diversity. To compare alternative choices of biotic variables, all three processes were assessed below using criterion D.

<u>Current decline</u>: Two meta-analyses have been carried out to estimate changes in coral cover (% of seafloor covered by live scleratinian corals) in the Caribbean region during recent decades (Gardner et al. 2003; Schutte et al. 2010).

Gardner et al. (2003) analysed data from 263 sites from 65 separate studies across the Caribbean and found that coral cover declined from an estimated 54±10% cover in 1977 to 9±1% cover in 2001, a decline of 83% (range 77-88%) over 24 years. However, few surveys were obtained for early years and may not have accurately estimated coral cover. Scutte & Bruno (2009) were able to obtain data from 3777 sites on 1962 reefs recorded between 1971 and 2006. These included a majority of sites that were surveyed only once and a smaller number (376) repeat-surveyed in two or more years. Based on this larger data set, Schutte et al. (2010) estimated that coral cover declined from 32±10.5% in 1971 to 13±1.5% in 2006, representing a reduction of 59% (range 33-75%) over 35 years (Schutte et al. 2010). Based on the repeat monitoring sites only, the estimate was in the upper part of this range (74% decline over 34 years). Both studies recorded the most precipitous declines in coral cover during the 1980s, coincident with major coral disease epidemics and bleaching events, from which reefs have generally failed to recover. Both studies also show a broad consistency across subregions, although there is some variability in declines between time periods and reefs. Neither study covered the entire 50-year period required for assessing criterion D. Although trends are uncertain prior to the 1970s, declines appear to

have continued for more than a century and possibly much longer (Pandolfi et al. 2003). The level of coral cover at which reefs are unable to support their characteristic native biota is uncertain, but likely to be greater than zero. For this assessment, it was assumed that collapse will occur at 0-5% coral cover. Using Schutte's et al. best estimates of coral cover decline, the relative severity of disruption to biotic interactions was estimated to be 100 × (32-13)/(32-5) = 70%, or 59% if collapse is assumed to occur at 0% coral cover. Using uncertainty bounds around Schutte's et al. best estimates of decline in coral cover produces estimates of relative severity as low as 100 × (21.5-14.5)/(21.5-0) = 33% and as high as 100 × (42.5-14.5)/(42.5-5) = 85%. As these estimates of relative severity are based on samples throughout the distribution of the ecosystem, the disruption to biotic interactions was assumed to have occurred over 100% of the extent of the ecosystem. The status of the ecosystem under criterion D1 is therefore **Endangered** (plausible range Vulnerable - Critically Endangered).

While the overall cover of coral is declining, the architecture of the reef structures is becoming more simplified. Alverez-Filip et al. (2009) carried out a meta-analysis of studies that quantified the surface rugosity at 464 sites on 200 reefs between 1969 and 2008. Rugosity was assessed using an index that scales to one for a flat surface and takes values around three for the most architecturally complex reefs. They found a decline in rugosity index from 2.4 in 1969 to 1.2 in 2008, a 50% decline over 39 years. The temporal pattern of decline included a period between 1985 and 1997 when rugosity remained stable. Declines prior to 1985 were apparently driven by outbreaks of coral disease, while those after 1997 were apparently driven by a series of thermal anomalies, associated bleaching and storms (Alvarez-Filip et al. 2009).

Reductions in reef fish community diversity and biomass, linked to unsustainable fishing practices can be traced back over extended periods of centuries in many areas of the Caribbean (Hughes 1994; Jackson 1997; Wing & Wing 2001; Hughes et al. 2003). Further evidence of the altered state of fish populations region-wide comes from the dramatic changes resulting from local fisheries closures. More recently, reductions in coral cover and reef rugosity have apparently led to further declines in diversity of reef fish (Bohnsack 2000; Halpern 2003; Mahon & Mascia 2003; Mumby & Harborne 2010). Paddack et al. (2009) analysed a time series of reef fish density from 318 sites across the distribution of Caribbean coral reefs during the period 1955-2007. For all species combined, densities were essentially stable from 1955 until a period of increase in 1981-1985, followed by continuing declines over 1996-2000 and 2001-2007, particularly in three trophic groups of fish: herbivores, invertivores and generalist carnivores. Overall, however, the recent declines compensated the earlier increase so that the mean rate of change over the 52 year period was not statistically different from zero. The increase in the 1980s coincided with mass mortality of the algal herbivore urchin *Diadema antillarum*, which may have delayed the onset of decline (Paddack et al. 2009). Paddack et al. (2009) suggest a degradation debt, in which trends in fish populations lag considerably behind changes in coral cover and rugosity, with declines in herbivorous fish potentially contributing to positive feedbacks by increasing algal dominance over coral.

The historical impacts of overfishing in the Caribbean appear to have taken place much greater than 50 years ago. Meanwhile, apparent lags in fish population densities make this a less suitable response variable for assessing disruptions to biotic interactions than coral cover and reef rugosity.

The estimates of relative severity and extent of declines in reef rugosity are within the range of values estimated for decline in coral cover: relative severity 33-85% over 100% of the extent of the ecosystem. Based on these estimates, the status of the ecosystem is **Endangered** (plausible range **Vulnerable - Critically Endangered** under criterion D1.

Future declines: No projections are available for disruption to biotic interactions in Caribbean coral reefs over the next 50 years. The status of the ecosystem is therefore **Data Deficient** under criterion D2.

Historic declines: Historic levels of coral cover in the Caribbean are uncertain. Bruno & Selig (2007) suggest that historic (100-1000 years ago) average coral cover in the Indo-Pacific was probably 50%. If a similar cover of coral occurred historically in the Caribbean, then a present-day mean estimate of 13±1% coral cover (Schutte et al. 2010) suggests a decline with relative severity of 100 × (50-11.5)/(50-0) = 71% to 100 × (50-14.5)/(50-5) = 88%. An alternative analysis presented by Pandolfi et al. (2003) based on a semi-quantitative index of reef degradation integrated across seven groups of biota, including corals and fish. Caribbean coral reefs span index values of 52 to 78, where 0 represents pristine condition and 100 represents ecological extinction. Standardising these changes to a 1750 baseline (equating to the 'colonial occupation' period of Pandolfi et al. 2003), produces an estimated relative severity of biotic decline approximately 65%. Both estimates are applicable across 100% of the extent of the ecosystem.

The two estimates of relative severity are remarkably concordant. The estimate derived from Pandolfi's degradation index is possibly lower due to averaging across some biotic groups that are less sensitive to threats than corals. Bruo & Selig (2007) argue that coral cover is a critical measure of habitat loss and degradation, given the key role that corals play in facilitating the entire reef ecosystem, hence the relative severity of decline iwas assumed to be 71-88%. Based on this approach, the status of the ecosystem is **Endangered** under criterion D3.

Criterion E

No quantitative analysis has been carried out to assess the risk of ecosystem collapse for Coastal sandstone upland swamps. The status of the ecosystem is therefore **Data Deficient** under criterion E.

REFERENCES

Alevizon WS. 2010. Introduction to Caribbean coral reefs including Florida and The Bahamas: structure, marine life, ecology. WS Alevizon, Gainesville.

Alvarez-Filip L, Dulvy NK, Gill JA, Côté IM, Watkinson AR. 2009. Flattening of Caribbean coral reefs: region-wide declines in architectural complexity. Proceedings of the Royal Society B 276 : 3019 -3025.

Anthony KRN, Kleypas JA, Gattuso JP. 2011. Coral reefs modify their seawater carbon chemistry – implications for impacts of ocean acidification: Global Change Biology 17: 3655-3666.

Andréfouët S, Muller-Karger FE, Robinson JA, Kranenburg CJ, Torres-Pulliza D, Spraggins SA, Murch B. 2006. Global assessment of modern coral reef extent and diversity for regional science and management applications: a view from space. In Proceedings of the Tenth International Coral Reef Symposium, Okinawa, Japan, p 14.

Aronson RB, Precht WF. 2001. White-band disease and the changing face of Caribbean coral reefs. Hydrobiologia 460: 25–38.

Aronson RB, Precht WF, Macintyre IG, Murdoch TJT. 2000. Ecosystems—coral bleach-out in Belize. Nature 405: 36.

Ateweberhan M, McClanahan TR. 2010. Relationship between historical sea-surface temperature variability and climate change-induced coral mortality in the western Indian Ocean: Marine Pollution Bulletin 60: 964-970.

Bohnsack JA. 2000. A comparison of the short-term impacts of no-take marine reserves and minimum size limits. Bulletin of Marine Science 66: 635-650.

Bender MA, Knutson TR, et al. 2010. Modeled Impact of Anthropogenic Warming on the Frequency of Intense Atlantic Hurricanes. Science 327: 454-458.

Boyer JN, Jones RD. 2002. A view from the bridge: External and internal forces affecting the ambient water quality of the Florida Keys National Marine Sanctuary (FKNMS). In The Everglades, Florida Bay, and coral reefs of the Florida Keys: An ecosystem source book (JW Porter, KG Porter, eds.). Boca Raton: CRC Press, pp. 609–628.

Bruckner AW, Bruckner RJ. 2006. The recent decline of Montastraea annularis (complex) coral populations in western Curaçao: a cause for concern? Revista de Biologia Tropical 54 (Suppl. 3): 45-58.

Bruno JF, Bertness MD. 2001. Habitat modification and facilitation in benthic marine communities. In Marine community ecology (eds. Bertness MD, Gaines SD, Hay ME). Sunderland: Sinauer, pp. 201–218.

Bruno JF, Selig ER, Casey KS, Page CA, Willis BL, et al. 2007. Thermal stress and coral cover as drivers of coral disease outbreaks. PLoS Biology 5: e124. [doi:10.1371/journal.pbio.0050124]

Burke L, Maidens J. 2004, Reefs at Risk in the Caribbean. Washington DC: World Resources Institute.

Burke L, Reytar K, Spalding M, Perry AL. 2011, Reefs at Risk Revisited, Washington DC: World Resources Institute, The Nature Conservancy, WorldFish Center, International Coral Reef Action Network, UNEP World Conservation Monitoring Centre and Global Coral Reef Monitoring Network.

CARMABI. 2012. Caribbean coral species identification tool. Caribbean Marine Biological Institute, Curacao. [http://www.researchstationcarmabi.org/news/latest-news/88-updated-caribbean-coral-species-identification-tool, searched 24/2/2012].

CARMABI Foundation. 2012. Dutch Caribbean biodiversity explorer. Caribbean Research and Management of Biodiversity Foundation, Curacao. [http://www.dcbiodata.net/explorer/home, searched 24/2/2012].

Cerdeira-Estrada S, López-Saldaña G. 2011. A novel Satellite-based Ocean Monitoring System for Mexico. Ciencias Marinas 37: 237–247.

Cooper TF, De'ath G, Fabricius KE, Lough JM. 2008. Declining coral calcification in massive Porites in two nearshore regions of the northern Great Barrier Reef. Global Change Biology 14: 529–538.

Donner, S. D., 2011, An evaluation of the effect of recent temperature variability on the prediction of coral bleaching events: Ecological Applications, v. 21, no. 5, p. 1718-1730.

Donner SD, Potere D. 2007. The inequity of the global threat to coral reefs: BioScience 57: 214-215.

Donner SD, Knutson TR, Oppenheimer M. 2007. Model based assessment of the role of human-induced climate change in the 2005 Caribbean coral bleaching event. Proceedings of the National Academy of Science USA 104: 5483–5488.

Dudgeon SR, Aronson RB, Bruno JF, Precht WF. 2010. Phase shifts and stable states on coral reefs. Marine Ecology Progress Series 413: 201–216.

Duerden JE. 1901. The marine resources of the British West Indies. West Indies Bulletin 2:121-163.

Dustan P. 1999. Coral reefs under stress: Sources of mortality in the Florida Keys. Natural Resource Forum 23: 147–155.

Fabricius KE. 2005. Effects of terrestrial runoff on the ecology of corals and coral reefs: review and synthesis. Marine Pollution Bulletin 50: 125–146.

Friedrich T, Timmermann A, Abe-Ouchi A, Bates NR, Chikamoto MO, Church MJ, Dore JE, Gledhill DK, González-Dávila M, Heinemann M, Ilyina T, Jungclaus JH, McLeod E, Mouchet A, Santana-Casiano JM. 2012. Detecting regional anthropogenic trends in ocean acidification against natural variability. Nature climate Change 2: 167–171.

Gil-Agudelo DL, Smith GW, Garzón-Ferreira J, Weil E, Peterson D. 2004. Dark spots disease and yellow band disease, two poorly known coral diseases with high incidence in Caribbean reefs. In Coral Health and Disease (eds. Rosenberg E, Loya Y). Berlin: Springer, pp. 337-350.

Goreau TF. 1959. The ecology of Jamaican coral reefs. I. Species composition and zonation. Ecology 40: 67-90.

Goreau TF, Goreau NI. 1973. The ecology of Jamaican coral reefs. II Geomorphology, zonation, and sedimentary phases. Bulletin of Marine Science 23: 399-464.

Guest JR, Baird AH, Maynard JA, Muttaqin E, Edwards AJ, Campbell SJ, Yewdall K, Affendi YA, Chou LM. 2012. Contrasting patterns of coral bleaching susceptibility in 2010 suggest an adaptive response to thermal stress: PLoS ONE 7(3): e33353.

Halpern B. 2003. The impact of marine reserves: do reserves work and does reserve size matter? Ecological Applications 13: S117–S137.

Hayes RL, Goreau TF. 2008. Satellite-derived sea surface temperature from Caribbean and Atlantic coral reef sites, 1984-2003. Revista de Biologia Tropical 56 (Suppl. 1): 97-118.

Hoegh-Guldberg O. 1999. Climate change, coral bleaching and the future of the world's coral reefs. Marine and Freshwater Research 50: 839–866.

Hoegh-Guldberg O, Mumby PJ, Hooten AJ, Steneck RS and others. 2007. Coral reefs under rapid climate change and ocean acidification. Science 318: 1737–1742.

Hughes TP. 1994. Catastrophes, phase-shifts, and large-scale degredation of a Caribbean coral reef. Science 265: 1547-1551.

Hughes TP, Baird AH, Bellwood DR, Card M, Connolly SR, Folke C, Grosberg R, Hoegh-Guldberg O, Jackson JBC, Kleypas J, Lough JM, Marshall P, Nystrom M, Palumbi SR, Pandolfi JM, Rosen B, Roughgarden J. 2003. Climate change, human impacts, and the resilience of coral reefs. Science 301: 929-933.

Hughes TP, Rodrigues MJ, Bellwood DR, Ceccarelli D, Hoegh-Guldberg O, McCook L, Moltschaniwskyj N, Pratchett MS, Steneck RS, Willis B. 2007. Phase Shifts, Herbivory, and the Resilience of Coral Reefs to Climate Change. Current Biology 17: 360-365.

Jackson JBC. 1997. Reefs since Columbus: Coral Reefs 16: S23-S32.

Jackson JBC, Kirby MX, Berger WH, Bjorndal KA and others. 2001. Historical overfishing and the recent collapse of coastal ecosystems. Science 293: 629–637.

Jones AM, Berkelmans R, van Oppen MJH, Mieog JC, Sinclair W. 2008. A community change in the algal endosymbionts of a scleractinian coral following a natural bleaching event: field evidence of acclimatization: Proceedings of the Royal Society B: Biological Sciences 275: 1359-1365.

Kleypas JA, Yates. 2009. Coral Reefs and Ocean Acidification. Oceanography 22: 108-117.

Knutson TR, Sirutis JJ, Garner ST, Vecchi GA, Held IM. 2008, Simulated reduction in Atlantic hurricane frequency under twenty-first-century warming conditions. Nature Geoscience 1: 359-364.

McClean RF, Tysban A. 2001. Coastal zones and marine ecosystems. In Climate change 2001: impacts, adaptation, and vulnerability (McCarthy JJ, Canziani OF, Leary NA, Dokken DJ, White KS, eds.) Cambridge: Cambridge University Press, pp 343–379.

La Jeunesse TC, Smith RT, Finney J, Oxenford H. 2009. Outbreak and persistence of opportunistic symbiotic dinoflagellates during the 2005 Caribbean mass coral 'bleaching' event. Proceedings of the Royal Society B. 276: 4139-4148.

Landsea CW, Vecchi GA, Bengtsson L, Knutson TR. 2010. Impact of duration thresholds on Atlantic tropical cyclone counts. Journal of Climate 23: 2508–2519.

Le Boef BJ, Kenyon KW, Villa-Ramirez B. 1986. The Caribbean monk seal is extinct. Marine Mammal Science 2: 70-72.

Leâo ZMAN, Kikuchi RKP, Viviane T. 2003. Corals and coral reefs of Brazil. In Latin American Coral Reefs (Cortés J, ed.) Amsterdam: Elsevier, pp. 9-53.

Mahon R, Mascia MB. 2003. The Barbados (alias Folkestone) Marine Reserve, Barbados: a late bloomer? Gulf and Caribbean Research 14: 171-180.

Maynard J, Anthony K, Marshall P, Masiri I. 2008. Major bleaching events can lead to increased thermal tolerance in corals. Marine Biology 155: 173-182.

McClenachan L, Cooper. 2008, Extinction rate, historical population structure and ecological role of the Caribbean monk seal. Proceedings of the Royal Society of London, B, v. online publication 10.1098/rspb.2007.1757.

McClenachan L, Jackson JB, Newman MJ. 2006. Conservation implications of historic sea turtle nesting beach loss: Frontiers in Ecology and the Environment 4: 290-296.

McWilliams JP, Côté IM, Gill JA, Sutherland WJ, Watkinson AR. 2005. Accelerating impacts of temperature-induced coral bleaching in the Caribbean. Ecology 86: 2055–2060.

Mumby PJ, Harborne AR. 2010. Marine reserves enhance the recovery of corals on Caribbean reefs. PLoS ONE 5(1): e8657.

Paddack M, Reynolds J, Aguilar C, Appledoorn R and others. 2009. Recent region-wide declines in Caribbean reef fish abundance. Current Biology 19: 590–595.

Pandolfi JM, Bradbury RH, Sala E, Hughes TP and others. 2003. Global trajectories of the long-term decline of coral reef ecosystems. Science 301: 955–958.

Palandro DA, Andréfouët S, Hu C, Hallock P, Müller-Karger FE, Dustan P, Callahan MK, Kranenburg C, Beaver CR. 2008. Quantification of two decades of shallow-water coral reef habitat decline in the Florida Keys National Marine Sanctuary using Landsat data (1984–2002). Remote Sensing of Environment 112: 3388–3399.

Ries J, Cohen A, McCorkle D. 2010. A nonlinear calcification response to CO_2-induced ocean acidification by the coral Oculina arbuscula. Coral Reefs 29: 661-674.

Robbins MW, Lund RB, Gallagher CM, Lu Q. 2011. Changepoints in the North Atlantic tropical cyclone record. Journal of the American Statistical Association 106: 89 - 99.

Rogers CS. 1990. Responses of coral reefs and reef organisms to sedimentation. Marine Progress Series 62: 185-202.

Schutte VGW, Selig ER, Bruno JR. 2010. Regional spatio-temporal trends in Caribbean coral reef benthic communities. Marine Ecology Progress Series 402: 115–122.

Selig ER, Casey KF, Bruno JF. 2010. New insights into global patterns of ocean temperature anomalies: implications for coral reef health and management. Global Ecology and Biogeography 19: 397–411.

Spalding MD. 2004. A Guide to the Coral Reefs of the Caribbean. Berkeley: University of California Press.

Spalding MD, Fox HE, Allen GR, Davidson N, Ferdaña ZA, Finlayson M, Halpern BS, and others 2007. Marine ecoregions of the world: a bioregionalization of coastal and shelf areas. Bioscience 57: 573–583.

Stoddart DR. 1963. Catastrophic storm effects on the British Honduras reefs and cays. Nature 196: 512-515.

Sutherland KP, Shaban S, Joyner JL, Porter JW, Lipp EK. 2011. Human pathogen shown to cause disease in the threatened Eklhorn Coral Acropora palmata. PLoS ONE 6(8): e23468.

van Oppen M, Lough JM. 2008. Coral bleaching: patterns, processes, causes and consequences. Berlin: Springer Verlag.

Weil E, Rogers CS. 2011. Coral Reef Diseases in the Atlantic-Caribbean. In Coral reefs: an ecosystem in transition (Dubinsky Z, Stambler N, eds.). Netherlands: Springer, pp 465-491.

Wilkinson CR, Souter D. 2008. Status of Caribbean coral reefs after bleaching and hurricanes in 2005. Global Coral Reef Monitoring Network and Reef and Rainforest Research Centre, Townsville.

Wilson SK, Graham NAJ, Pratchett MS, Jones GP, Polunin NVC. 2006. Multiple disturbances and the global degradation of coral reefs: are reef fishes at risk or resilient? Global Change Biology 12: 1–15.

Wing SR, Wing ES. 2001. Prehistoric fisheries in the Caribbean. Coral Reefs 20: 1-8.

Woodley JD, Chornesky EA, Clifford PA, Jackson JBC and others. 1981. Hurricane Allen's impact on Jamaican coral reefs. Science 214: 749–755.

18 SEAGRASS COMMUNITY OF SOUTH AUSTRALIA

contributed by Ronald Bonifacio and Phil Pisanu, Science Resource Centre, Department of Environment and Natural Resources, South Australia May 21, 2012

CLASSIFICATION

International: Den Hartog (2003) proposed a global classification of seagrass communities, of which this assemblage is a local expression of an alliance dominated by Posidonia.
IUCN Habitats Classification Scheme (Version 3.0): 9. Marine Neritic / 9.9 Seagrass (Submerged)

ECOSYSTEM DESCRIPTION

Characteristic native biota

In Australia, 30 different species of seagrass from 12 genera are known to exist (Kirkman, 1997). Thirteen species are found in South Australia (SA) belonging to seven genera (Wear et al. 2006; Westphalen et al. 2004) with an estimated area of 9,620 km^2 (Edyvane 1999). The main seagrass species occurring in SA are *Posidonia angustifolia, Posidonia australis, Posidonia coriaceae, Posidonia sinuosa, Amphibolis antarctica, Amphibolis griffithii, Heterozostera tasmanica, Halophila australis and Zostera muelleri* (Larkum & den Hartog 1989).

The majority of seagrasses inhabit the semi-enclosed bays of Gulf St. Vincent and Spencer Gulf, which form the most extensive seagrass meadows (5,000 km^2) in South Australia (Shepherd and Robertson 1989), but they are also found in embayments in the western part of the State, Kangaroo Island (Kinloch et al. 2007) and the South East. The seagrass communities in South Australia are considered as amongst the largest and most diverse in the world (Shepherd et al. 1989). The ecological community assessed here (Figure 1) is the meadow forming seagrass species, which is usually dominated by ribbon weed or tape weed (*Posidonia* spp) and wire weed (*Amphibolis* spp) (Bryars et al. 2011; EPA 2008; EPA 2009; Miles and Peters 2011).

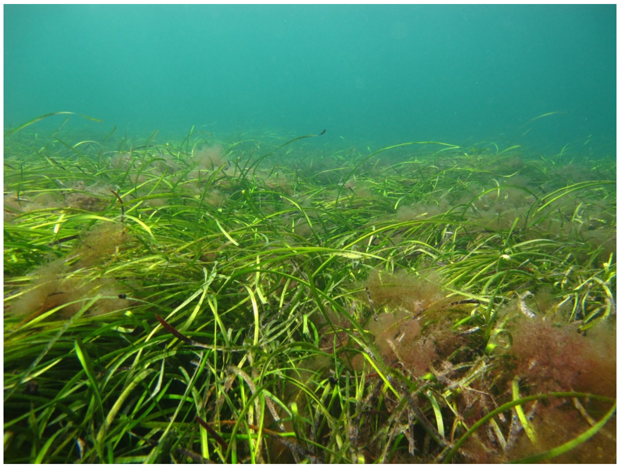

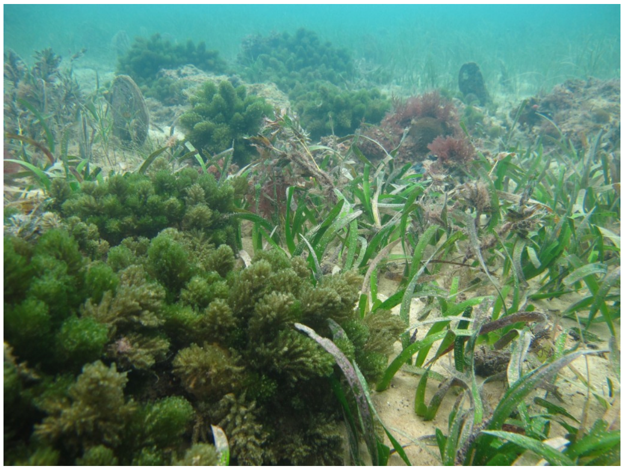

Figure S18. 1. Seagrass community of South Australia dominated by: a) *Posidonia sinuosa;,* and b) a mixture of *Posidonia australis* and *Amphibolis antarctica* with macro-alga *Caulerpa obscura*. Photos: Kym Lasmar.

Tubeworms, ascidians, seastars, sea urchins, crabs and razorfish (*Pinna* spp.) were found to be components of seagrass meadows in Gulf St Vincent (Shepherd and Sprigg 1976). At Kangaroo Island, seagrass meadows are dominated by “small shrimps (decapods), slaters (amphipods), sea lice (isopods)

and snails, crabs and syngnathid fish (pipefish and seahorses), weedy whitings, scorpionfish and clingfish, plus the odd seastar, polychaete worm and sea cucumber" (Kinloch et al. 2007, p 21).

Abiotic environment

Seagrasses are generally found on sandy or muddy areas in estuaries, coastal lagoons, gulfs and sheltered bays (EPA 2009). Seagrasses, particularly those found in South Australia, are confined to naturally low nutrient environments and as such they are sensitive to any increases in nutrient levels. They are also sensitive to increases in water turbidity as they require sunlight for growth.

Distribution

The majority of seagrass (82.7%) is found in the waters of Gulf St. Vincent (Adelaide metropolitan area) and Spencer Gulf (Eyre Peninsula) (Figure 2). They are also found in Streaky Bay and Smoky Bay (west of Spencer Gulf) with Fowlers Bay as the western most site containing extensive seagrass meadows (Kirkman 1997). In the South East, the seagrass community is found in shallow sheltered bays of Lacepede Bay, Guichen Bay, Nora Creina, Stinky Bay, Rivoli Bay, Bucks Bay, Bungaloo Bay and in waters adjacent to the township of Port MacDonnell. The seagrass habitat in South Australia is estimated to be over 9,620 km^2 (Edyvane 1999). However, the most current analysis using 1:100,000 benthic habitat mapping covering SA's coastal waters (to 20 m depth) indicates that the area of seagrass is 8,532 km^2. It is not certain whether this discrepancy is due to difference in methodology or actual loss since the earlier estimate or whether seagrass species occurring in deeper waters were not captured by the latest analysis.

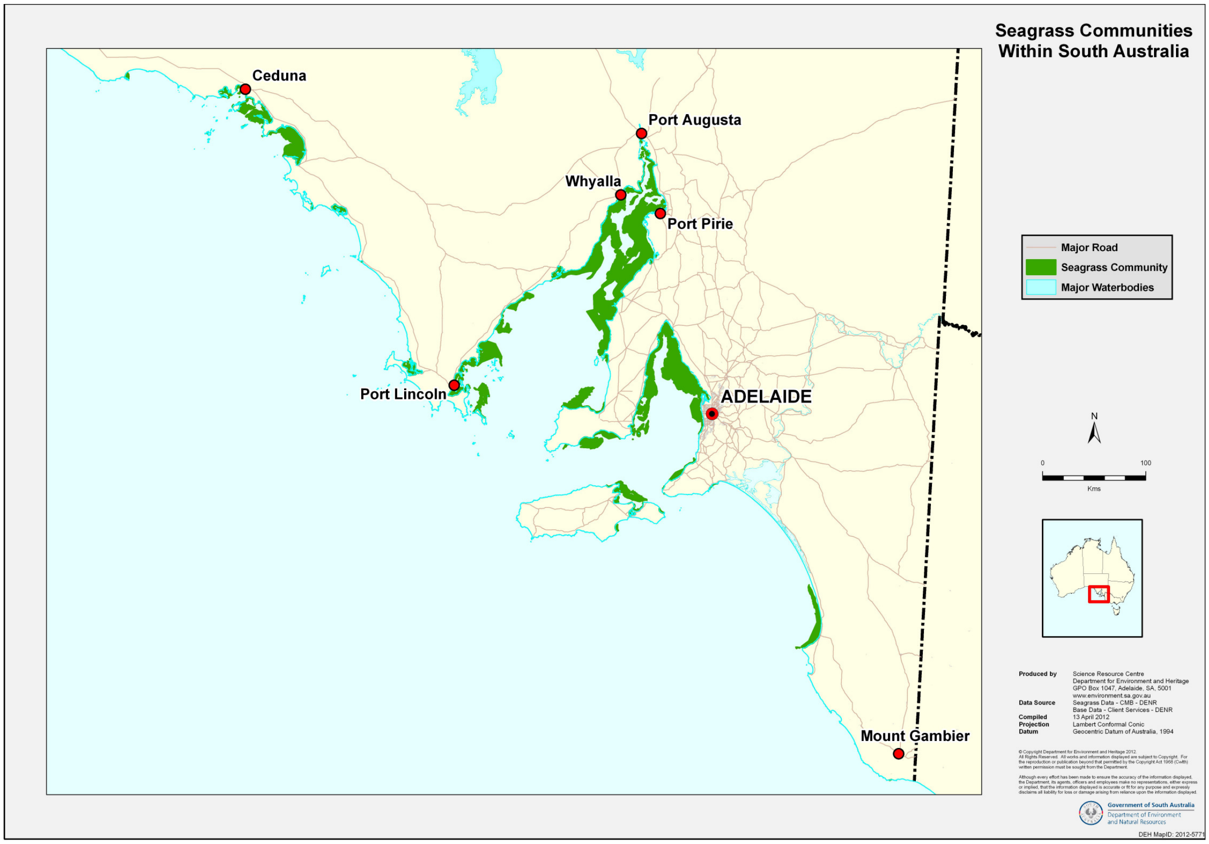

Figure S18. 2. The distribution of the Seagrass Community in South Australia

Key processes and interactions

Generally, seagrasses grow on sandy or muddy substrates and are dependent upon their rhizomes or underground stems for anchorage (Kirkman 1997). Seagrass meadows are not capable of withstanding great energy from swell and waves and thus are usually found in sheltered bays. The critical factors for seagrass growth and survival are light, temperature, dissolved carbon dioxide, nutrients and a suitable substrate for anchoring (Figure 3). The extensive root systems of seagrass meadows stabilise the underlying sediments (Fox et al. 2007). In South Australia, the largest seagrass meadows are found in Spencer Gulf and Gulf St Vincent. A conceptual model regarding the effect of light and nutrient to seagrasses is shown in Figure 3.

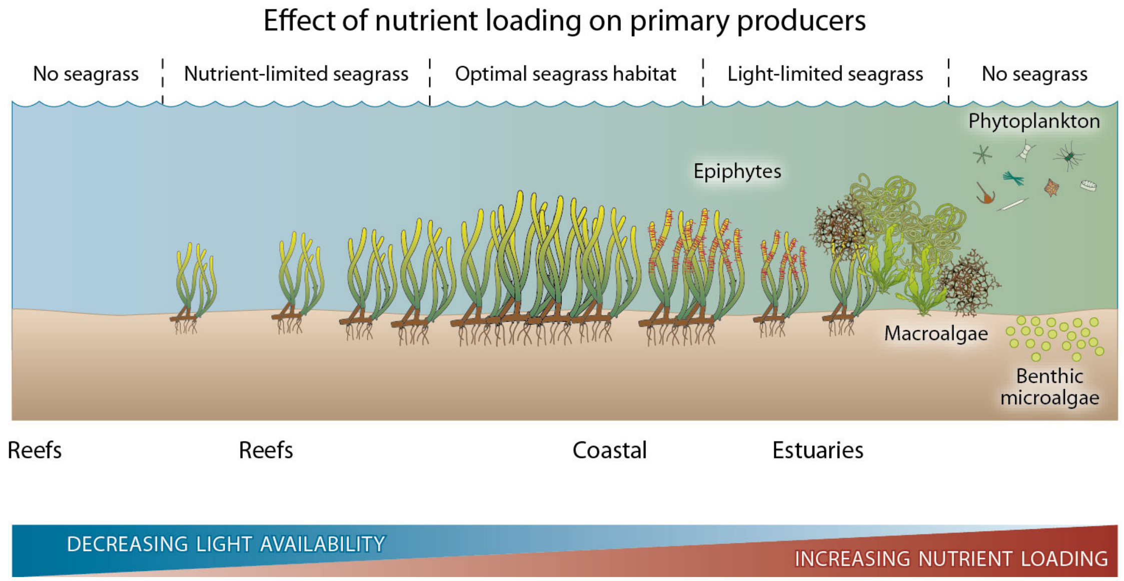

Figure S18. 3**.** Effect of substrate, light and nutrients on environmetnal suitability for seagrasses.

Seagrass ecosystems have been reported to contribute up to 15% of global ocean annual net carbon production (Duarte and Chiscano 1999). Seagrass ecosystems have been ranked closely with coral reefs and mangrove habitats in terms of productivity (Short and Wyllie-Echeverria, 1996). This highly productive ecosystem provides habitats and nursery areas to a variety of marine invertebrates and vertebrates (see above).

The Science Resource Centre of the Department of Environment and Natural Resources conducted an expert panel workshop (March 2012) to create a conceptual model of the seagrass community in South Australia (Figure 4). The conceptual model provides an illustration of the drivers of change operating within this marine ecological community.

Threatening processes

Urbanisation of coastal areas and near shore development has resulted in decline in water quality affecting seagrasses (Shepherd et al. 1989; Seddon, 2000). Changes in water properties such as increases in temperature, pollutant levels and turbidity, as well as nutrient enrichment and altered salinity may negatively affect seagrasses. The secondary effect of increased epiphytic load as a result of high levels of nutrients in water is also detrimental to seagrasses (Bryars et al. 2011). Sources of nutrients and pollutants in South Australia's marine environment are urban and rural runoff, sewage treatment plants, and some industrial sources.

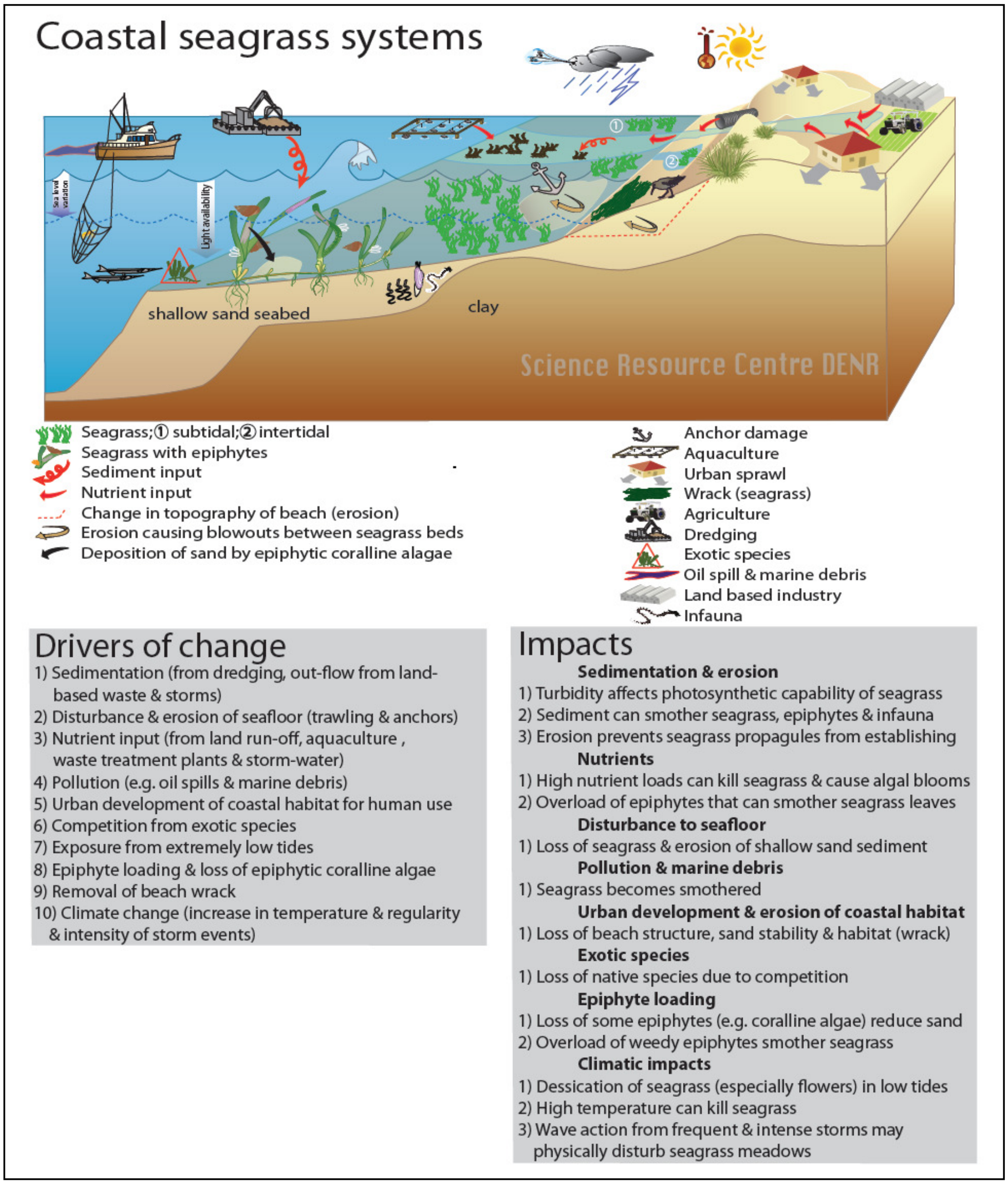

Figure S18. 4. Conceptual model of the seagrass community in South Australia (Science Resource Centre, 2012)

Ecosytem collapse

For assessment of criteria A and B, the seagrass community was assumed to collapse if its mapped distribution declined to zero. For assessing environmental degradation under criterion C, experimental work by Bryars et al. (2011) was used to set thresholds of collapse for seagrass due to high levels of ammoniacal and oxidized nitrogen concentration in the water column that result in high or total mortality of seagrass. It was assumed that the ecosystem would collapse when either inorganic form of nitrogen is maintained above the threshold levels 100% of the time throughout the distribution of the ecosystem (i.e. at all sample sites).

ASSESSMENT

Summary

Criterion	A	B	C	D	E	Overall
sub-criterion 1	EN(VU-CR)	LC	EN(VU-EN)	DD	DD	EN(VU-CR)
sub-criterion 2	DD	LC	DD	DD		
sub-criterion 3	VU(NT-CR)	LC	DD	DD		

Criterion A

Current decline. An estimate of decline for the entire distribution of seagrass meadows in South Australia has never been done. However, separate site-specific studies have been conducted in the past. For example, the loss of seagrass meadows in the coastal waters of metropolitan Adelaide (Largs Bay to Aldinga) was estimated to be 40.86 km^2 (32.8%) from 1949 to 1996 (Hart 1997). This change was inferred from the estimated increase of sand substrate from 26.26 to 67.12 km^2 during that time period. Extrapolated (slightly) to a full 50-year period, the estimated decline in distribution in this area was 33.3% (Table 1). Causes of decline were attributed by the Environmental Protection Agency to increased development within Metropolitan Adelaide, which in turn resulted in increased discharges in the Gulf St. Vincent (Figure 5).

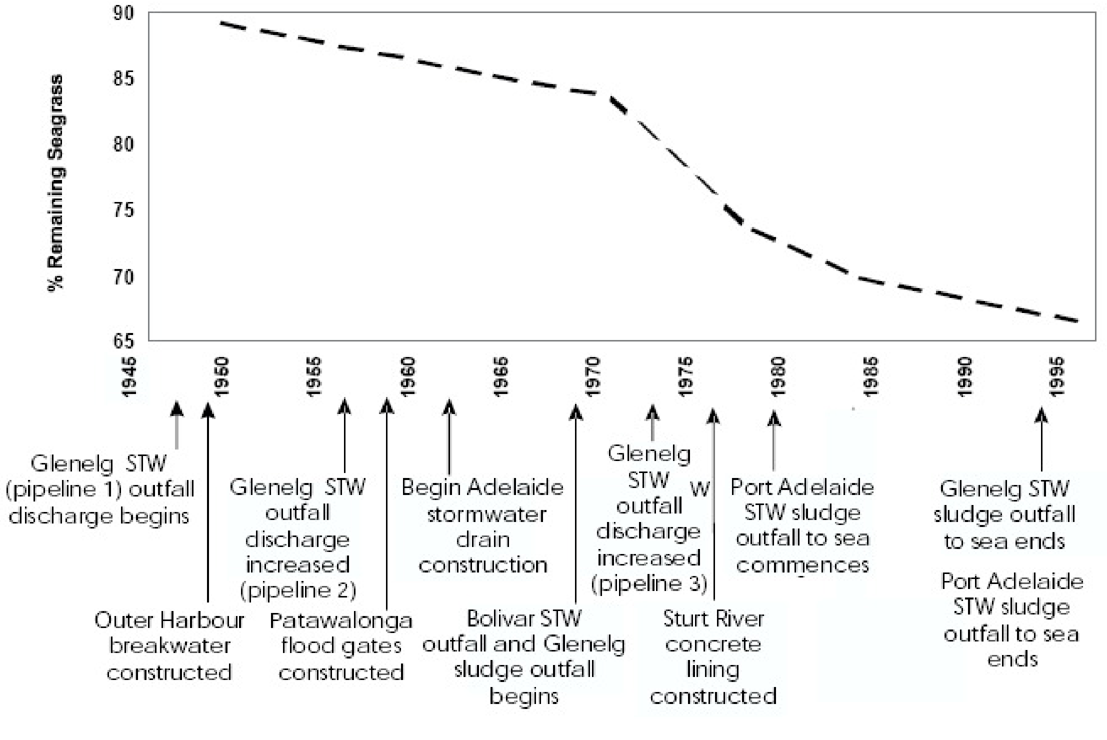

Figure S18. 5. Rate of seagrass loss 1949-1995 between Largs Bay and Glenelg (source: EPA 2008).

In a separate analysis, a particular site within this area (between Glenelg North and West Beach) was reported to have lost 50% of the seagrass meadow during the period 1949 to 1995 (Fox et al. 2007). Outside of Gulf St. Vincent in Rivoli Bay, South East, seagrass extent in 1951 was estimated to be 0.364 km^2 (36.4 ha), but declined to just 0.077 km^2 (7.7 ha) by 1997 (Seddon et al. 2003). This is a total loss of 0.287 km^2 (28.7 ha) or 78.85% of seagrass area at northern Rivoli Bay in 46 years. Extrapolating to 50 years gives an estimated decline of 81.5% (Table 1). The seagrass meadow in Rivoli Bay constitutes a small proportion of the total area of seagrasses in SA but is of major importance since this is the only location in the South East of SA where a seagrass meadow is found.

These studies are the only analyses conducted on changes of seagrass cover in South Australia that cover a span of more than 45 years.

There are other studies on other sites, albeit more recent ones. For example, Seddon (2000) documented the loss of approximately 60% (from 159.3 to 64 km^2) of near-shore seagrasses from a 95 km stretch of coast in Spencer Gulf from 1987 to 1994 due to heat stress. This short-term study may have failed to capture increased environmental degradation and significant losses in seagrass meadows that occurred before and during the 1970's, about 8 years after the maximum rate of population growth in the metropolitan region (EPA 2008; Fox et al. 2007). The estimated decline over 50 years depends on whether a similar rate of decline was maintained over the past 50 years (99.9%) or whether a decline only took place between 1987 and 1994 (68.0%) (Table 1).

Table S17. 1. Estimated percentage declines in distribution of the Seagrass community over the past 50 years assuming constant proportional rates of decline over respective survey periods.

Assumption	Proportional annual rate of decline (r)	% decline over past 50 yrs	Calculation (r)	Calculation (% decline)
1 Range-wide proportional rate of decline over past 50 yrs is same as annual rate at Largs Bay - Aldinga during 1949 - 1998 (Hart 1997)	-0.008	33.3	ln(83.7/124.6)/(1998-1949)	$100x(1-e^{-0.008})^{50}$
2 Range-wide proportional rate of decline over past 50 yrs is same as annual rate at Rivoli Bay during 1951 - 1997 (Seddon et al. 2003)	-0.034	81.5	ln(0.077/0.364)/(1997-1951)	$100x(1-e^{-0.034})^{50}$
3 Range-wide proportional rate of decline over past 50 yrs is same as annual rate in 95km stretch of Spencer Gulf during 1987-1994 (Seddon 2000)	-0.13	99.9	ln(64.0/159.3)/(1994-1987)	$100x(1-e^{-0.13})^{50}$
4 Range-wide proportional rate of decline over past 50 yrs is same as area lost in 95km stretch of Spencer Gulf during 1987-1994, averaged over a 50 yr period (Seddon 2000)	-0.023	68	ln(64.0/159.3)/(1994-1954)	$100x(1-e^{-0.023})^{50}$
5 Area-weighted average based on 1, 2 & 3	-0.077	**97.8**	(-0.008x124.6-0.034x0.364-0.13x159.3)/(124.6+0.034+159.3)	$100x(1-e^{-0.077})^{50}$
6 Area-weighted average based on 1, 2 & 4	-0.016	**55.9**	(-0.008x124.6-0.034x0.364-0.023x159.3)/(124.6+0.034+159.3)	$100x(1-e^{-0.016})^{50}$

The status of the community can be based on the analyses of more than 45 years in two sites (Largs Bay to Aldinga and Rivoli Bay), assuming that these sites are representative of the overall pressures and decline on seagrass community in SA. This produces an estimated decline of 33 - 82% (Table 1). Alternatively, weighted average declines across all three sites produce an estimate of 56 - 98% decline, depending on whether a lower or upper bound is used for the third site (Table 1). The threat status therefore of this ecosystem is Endangered (plausible range Vulnerable - Critically Endangered (VU-CR) under criterion A1.

Future decline. It is well recognised that factors critical for seagrass growth (light, temperature, CO_2, nutrients and suitable substrate) are affected by climate change (Conolly, 2009). The loss of 127.17 km^2 of near-shore seagrasses in Spencer Gulf from 1987 to 1994 (Seddon et al. 2000) may represent the potential impact of climate change to seagrasses due to increased ambient temperature. However,

there are no studies nor modelling conducted for possible decline in seagrass meadows in South Australia using factors associated with climate change as independent variables. Also, there is no predictive modelling of seagrass extent based on current rate of decline. The status of the community is therefore Data Deficient (DD) under criterion A2.

Historic decline. There is anecdotal evidence of historical loss in the area of the seagrass community in the southern part of Rivoli Bay in the South East. The following account was cited by Wear et al (2006): "Firstly, an early nautical survey of Rivoli Bay in 1871 reported at Southend *weeds or grass showing in places during fine weather similar to those observed in the northern end of the bay* (Howard 1871). Secondly, a 1956 Marine and Harbours Board survey line shows what appears to be a remnant seagrass bank. This feature was no longer evident when resurveyed by the Coast Protection Board in 1987 and 2005"
If the above historical account is true, the loss in seagrass in the southern part of Rivoli Bay is 100% since 1871. There was also a record of seagrass dredging conducted near Port Broughton (Spencer Gulf) to harvest *Posidonia* fibre before the First World War (Winterbottom 1917). However, estimation of the extent of loss is not possible (Kirkman 1997).

Given the paucity of data in historical decline of seagrass meadows in South Australia, the estimated 29% decline in global seagrass meadow area between 1879 and 2006 (Waycott et al. 2009) is recommended to be used as surrogate measure representing the lower bound of estimated historic decline, with the upper bound the same as the upper boud of current decline (98%, Table 1). The use of this estimate is warranted since it is an average global estimate and there is evidence of continuing decline in South Australia's seagrass community (EPA 2008). The status of the ecosystem under criterion A3 therefore is likely to be at least Vulnerable (plausible range Near Threatened - Critically Endangered).

Criterion B

The Extent of Occurrence (EOO) and Area of occupancy (AOO) were determined using 1:100,000 benthic habitat mapping covering SA's coastal waters (to 20 m depth). Benthic habitat mapping was undertaken by the CSIRO and SARDI in 2001 as part of the National CSIRO Marine Habitat mapping program. Using this polygon information, seagrass communities were extracted and combined with the generated grid to determine the AOO and further differentiate the unique cells where seagrass areas are both below and above the 1% level of cell area occupied. The Extent of Occurrence (EOO) was determined by generating a minimum convex polygon that bound and encompassed the entire seagrass community polygon on the distribution map (Figure 6). The Area of Occupancy (AOO) of the seagrass community was estimated by overlaying the entire seagrass community polygon with a 10 km x 10 km and counting the number of occupied grids.

Extent of occurrence. The extent of occurrence of the seagrass meadow of South Australia is estimated to be 167,913 km^2 (see Figure 4). The decline in the extent of seagrass community is continuous (EPA 2008) and threats such as nutrient enrichment and other types of pollution still exist. The status of the EC therefore is Least Concern (LC) under criterion B1.

Area of occupancy. Superimposing a 10 x 10 km grid over the mapped polygons of seagrass meadow (Figure 6) indicates that they are present within 326 grid cells. Of these, 48 grid cells contains less than 1 km^2 of the community (i.e. <1% of the area of a grid cell). Excluding these small occurrences, the ecosystem is therefore estimated to occupy 278 grid cells. The assessment of B2 sub-criterion is similar to those for the subcriterion B1, which place the community under Least Concern (LC) for criterion B2.

Number of locations. Based on the distribution map and the community's Area of Occurrence (AOO), the seagrass meadow of South Australia occurs in 278 10 × 10 km grid cells, which could be interpreted as independent locations. If larger clusters of contiguous cells were interpreted as independent locations, there would be no fewer than ten. The status of the community is therefore Least Concern (LC) under criterion B3.

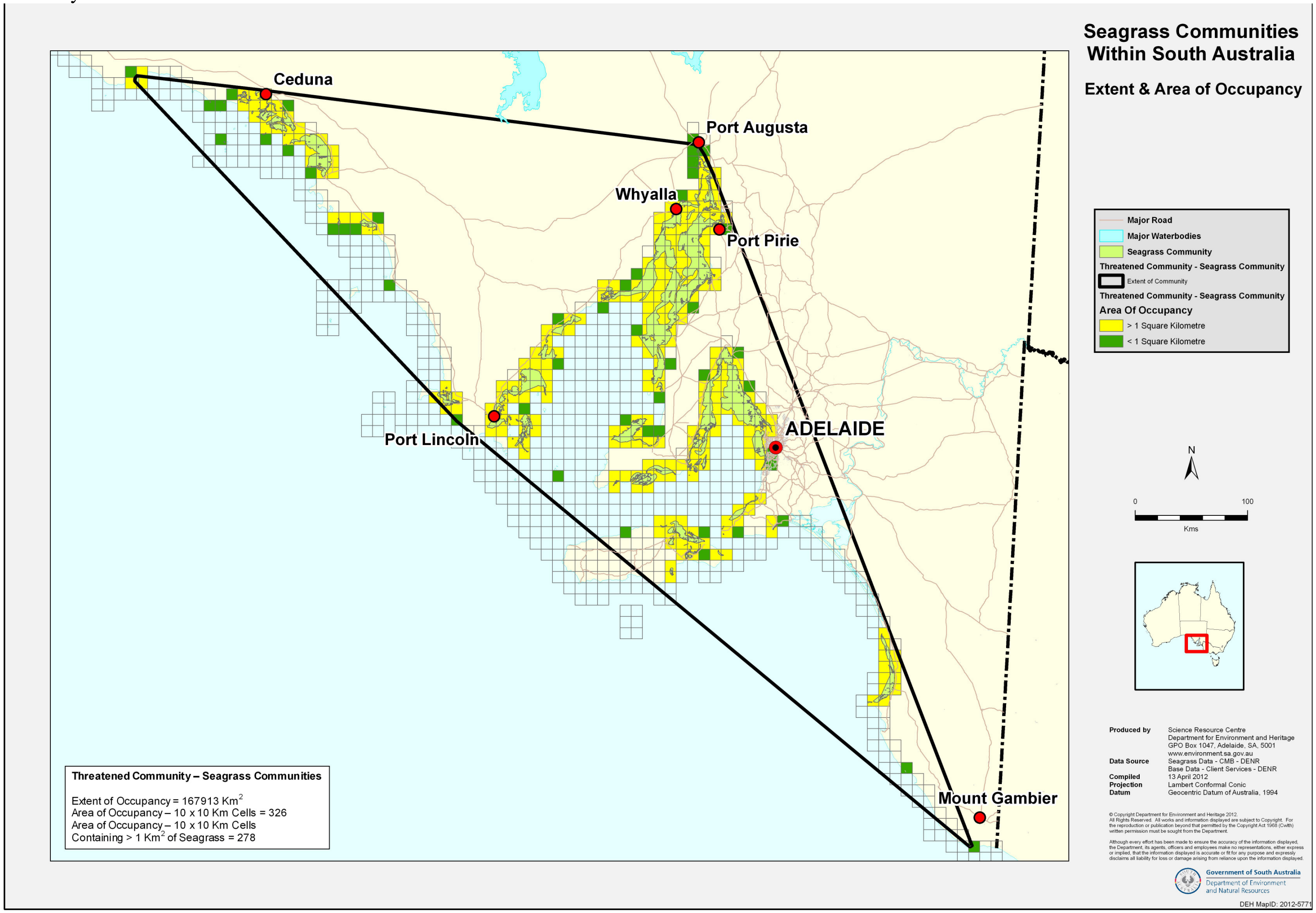

Figure S18. 6**.** Map polygon of the Seagrass Community of South Australia (SCSA) showing EOO and AOO. Green - all occupied cells. Yellow – with more than 1% of cells area occupied.

Criterion C

The principal mechanism of environmental degradation is through decline in water quality caused by agricultural runoff, land-based pollution and changes in the optimal conditions for seagrasses due to natural events. Data are available for nutrient levels, physico-chemical properties of water and other pollutants but are limited to specific sites and short periods of time (less than 15 years). In the assessment under Criterion C, the experimental work of Bryars et al. (2011) was used to set the threshold of collapse for seagrass community due to increased levels of ammoniacal and oxidised nitrogen concentration in the water column. In that study, an annual mean concentration of 0.026 mg/L of ammoniacal nitrogen and 0.025 mg/L of oxidised nitrogen were shown to decrease seagrass biomass and density by 70-80% and 50-55%, respectively. The inorganic forms of nitrogen (such as ammoniacal nitrogen and oxidised nitrogen) were used as the proxy measure for environmental degradation since they are biologically available (Fox et al. 2007). It was assumed that the ecosystem would collapse when either inorganic form of nitrogen is maintained above the threshold levels 100% of the time throughout the distribution of the ecosystem (i.e. at all sample sites).

Current decline. Ten to eleven years of water quality data from the Environmental Protection Authority (EPA) were used to determine the extent and relative severity of environmental degradation that negatively affects seagrasses in South Australia. These data were collected from specific sites ($n = 9$) in the coastal waters of the State, covering the Spencer Gulf, Gulf St. Vincent and Kangaroo Island. However, the water quality data are highly fluctuating and exhibit a lack of trend through time (Figure 7).

It can be surmised that the concentration of nutrients in the coastal waters of the South Australia fluctuate as opposed to being present at a continuous chronic level. This can be attributed to high rainfall events that flush nutrients and other pollutants from land base sources. In addition, data from some sites showed decreasing trends through time when the annual mean was computed, plotted against time and a polynomial trendline fitted (Fig. 8). The decrease in the level of nutrients may have been the result of efforts by the government regulating agencies to reduce the amount of pollutants entering the coastal waters through more efficient treatment facilities.

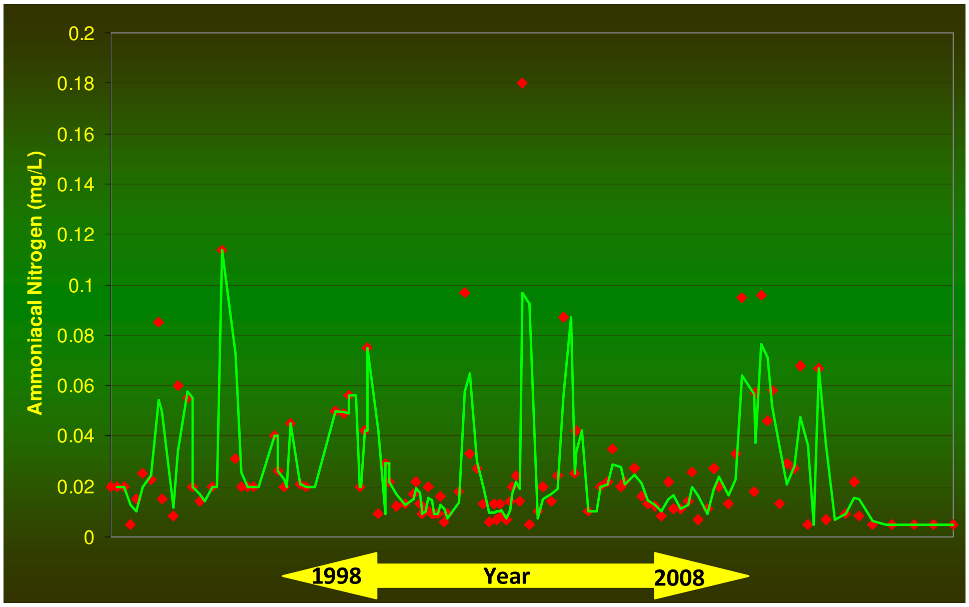

Figure S18. 7. Ammoniacal nitrogen concentration near Brighton Jetty, Gulf St. Vincent, SA showing highly fluctuating levels from 1998 to 2008 (data points fitted with moving average trendline).

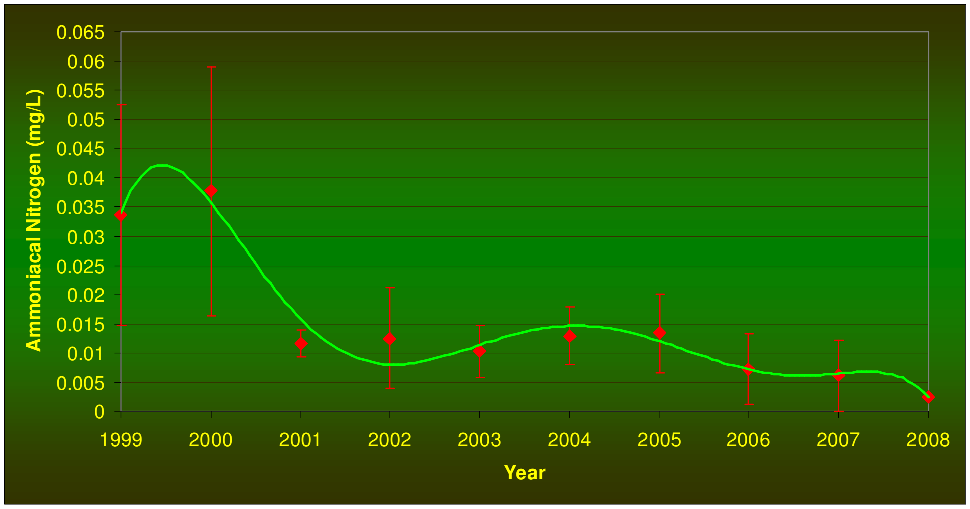

Figure S18. 8. Ammoniacal nitrogen concentration offshore of Nepean Bay, Kangaroo Island, SA showing relative decrease in levels from 1999 to 2008 (data points fitted with a 6th order polynomial, $\boldsymbol{R}^2 = 0.9613$).

Given the above scenario, the severity of environmental degradation was computed for each component of inorganic nitrogen using the following formula:

$$\textbf{Severity of abiotic degradation} = \left(\frac{b}{n}\right)\times 100$$

Where: $\boldsymbol{b}$ = number of observed values (annual mean) that is greater than $\boldsymbol{a}$
$\boldsymbol{a}$ = the value inferred as the threshold of collapse
$\boldsymbol{n}$ = the total number of observation

The inferred thresholds of collapse (a) were derived from the work of Bryars et al. (2011) (see above). The estimates of relative severity were averaged across all sites, with the resulting mean taken as representing the severity of declines across 100% of the ecosystem extent.

This approach to assessing environmental degradation under criterion C means that the ecosystem will be assessed at increasing levels of risk as nitrogen levels are more frequently maintained above threshold levels during the past 10 years at more of the sites (for purposes of assessment, we assume the past 10 years represent the past 50 years). If nitrogen levels become less frequently above the thresholds in future, then the risk status of the ecosystem will improve.

The use of frequency data in relation to a collapse threshold for assessing relative severity was justified due to the following reasons: i) the method is less sensitive to fluctuations, which may obscure environmental degradation which may be undetectable as trends nutrient enrichment in the water column with 10 to 11 years of the available data; ii) the data are highly fluctuating suggesting that increased nutrient levels in the water column comes in fluxes; and iii) many of the observed nutrient level values were above the inferred threshold of collapse values. These values were assumed to have a negative effect on seagrasses even without a clear linear or polynomial trend particularly when yearly averaged was used since the threshold of collapse values were also annual means.

Applying the above formula, the relative severity of degradation (using nutrients as the surrogate measure) are shown for different sites in Table 2. Sample computations are given in Appendix A.

Table S17. 2. The relative severity and extent of environmental degradation that negatively affects seagrass community in South Australia (values are derived from annual means and expressed as percentage above threshold collapse values.

General location	Sites	Relative severity based on Ammoniacal N (mg/L)	Relative severity based on Oxidised N (mg/L)
Gulf St. Vincent	Brighton Jetty	45.45	18.18
	Glenelg Jetty	81.82	27.27
	Grange Jetty	72.73	45.45
	Henley Beach	72.73	36.36
	Semaphore Jetty	45.45	9.09
	Largs Bay Jetty	45.45	9.09
Spencer Gulf	Port Hughes Jetty	69.23	23.08
Kangaroo Island	Nepean Bay	20.00	0.00
	Port Morrison	20.00	0.00
Mean (se) over 100% extent		53 (8)	19 (5)

Using values for ammoniacal nitrogen, the average extent of environmental degradation across 100% of the ecosystem extent is 53±8% (plausible bounds 45-61%). The status of the community is therefore Endangered (plausible range Vulnerable - Endangered) under criterion C1. However, this could be a conservative estimate since data were available extend back to 1998 only and environmental conditions would have been worse in earlier years (Fox et al. 2007; EPA 2008).

Future decline. There are no available data to estimate the parameter under this criterion. The status of the community is therefore Data Deficient (DD) under criterion C2.

Historic decline. There are no available data to estimate the parameter under this criterion. The status of the community is therefore Data Deficient (DD) under criterion C3.

Criterion D

Changes in the biotic component of seagrass communities have been observed. For example, seagrass communities of the Adelaide Coastal Waters are generally dominated by a mix of *Posidonia* and *Amphibolis* but a noticeable trend away from this distinctive composition has been observed by Fox et al. (2007). In another example, the loss of seagrass cover and eventual removal of sediment (due to wave action) exposes clay or calcrete base material that increases the risk of colonisation by exotic pests (Fox et al. 2007). These two examples provide qualitative evidence that disruption of biotic processes and interactions may have been occurring among the seagrass community of South Australia due to anthropogenic and natural perturbations. However, such changes in the biotic component of this ecological community have never been quantified.

Current decline. In the same study of Bryars et al. (2011), elevated levels of ammoniacal and oxidised nitrogen (see above Criterion C) resulted in the increase in epiphytic load and changes in epiphytic composition. The increase in epiphytic load and shift in composition may have been factors contributing to the decline of seagrass biomass and density due to decreased light availability. As these have not been quantified, the status of the community is Data Deficient (DD) under criterion D1.

Future decline. There are no available data to estimate the parameter under this criterion. The status of the community is therefore Data Deficient (DD) under criterion D2

Historic decline. There are no available data to estimate the parameter under this criterion. The status of the community is therefore Data Deficient (DD) under criterion D3

Criterion E

No quantitative analysis has been carried out to assess the risk of EC collapse for Seagrass Community of South Australia. The status of the ecosystem is therefore Data Deficient (DD) under criterion E.

REFERENCES

Bryars, S., Collings, G. and Miller, D. 2011. Nutrient exposure causes epiphytic changes and coincident declines in two temperate Australian seagrasses. *Marine Ecological Progress Series*, 441: 89-103.

Cameron, J. and Hart, D. 2002. Near shore seagrass mapping, Cygnet River, Kangaroo Island, South Australia. Department for Environment and Heritage, Adelaide.

Connolly, R.M. 2009. Seagrass. In A Marine Climate Change Impacts and Adaptation Report Card for Australia 2009 (Eds. E.S. Poloczanska, A.J. Hobday and A.J. Richardson), NCCARF Publication 05/09, ISBN 978-1-921609-03-9.

den Hartog, C. 2003. Phytosociological classification of seagrass communities. *Phytocoenologia* 33: 203 -229

Duarte, C.M., Chiscano, C.L., 1999. Seagrass biomass and production: a reassessment. *Aquatic Botany* 65: 159–174

Edyvane, K. S. 1999. Conserving marine biodiversity in South Australia – Part 2 – Identification of areas of high conservation value in South Australia. South Australian Research and Development Institute (Aquatic Sciences), Adelaide. 281 p.

EPA (Environmental Protection Agency) 2008. Changes in seagrass coverage and links to water quality off the Adelaide Metropolitan coastline. Government of South Australia, Adelaide.

EPA (Environmental Protection Agency) 2009. Adelaide Coastal Waters Information Sheet No. 1: Importance of Seagrasses. Government of South Australia, Adelaide.

Fox, D.R., Batley, G.E., Blackburn, D., Bone, Y., Bryars, S., Cheshire, A., Collings, G., Ellis, D., Fairweather, P., Fallowfield, H., Harris, G., Henderson, B., Kämpf, J., Nayar, S., Pattiaratchi, C., Petrusevics, P., Townsend, M., Westphalen, G., Wilkinson, J. 2007. The Adelaide coastal waters study, Final report, Volume 1: Summary of Study Findings. CSIRO, Adelaide.

Hart, D. 1997. Near shore seagrass change between 1949 and 1996 mapped using digital aerial orthophotography metropolitan Adelaide area Largs Bay – Aldinga South Australia. Report to Environmental Protection Authority, Department of Environment and Natural Resources (DENR) and SA Water, South Australia. DENR, Adelaide 45 pp.

Howard, F. (1871) Nautical description of Rivoli Bay. No. 23.

Kinloch M. A., Brock D. J., Kirkman H. and Laperousaz T. 2007. *Seagrass Biodiversity on Kangaroo Island*. KI NRM Board Coast and Marine Program Report No. CMP07/004.

Kirkman, H. 1997. *Seagrasses of Australia*, Australia: State of the Environment Technical Paper Series (Estuaries and the Sea), Department of the Environment, Canberra.

Larkum A.W.D. & den Hartog, C. 1989. Evolution and Biogeography of Seagrasses. In: Larkum, McComb & Shepherd (Eds), Biology of Seagrasses, Elsevier, Amsterdam.

Miles, M.W and Peters, D. 2011. *Coastal Carbon Sinks in South Australia*, South Australian Department for Environment and Natural Resources, Adelaide

Seddon, S., 2000. Causes and ecological consequences of the Spencer Gulf seagrass dieback. PhD Thesis, Department of Environmental Biology, University of Adelaide, Adelaide, pp. 171.

Seddon, S. Conolly, R. M. and Edyvane, K. S. 2000. Large-scale seagrass dieback in northern Spencer Gulf, South Australia. *Aquatic Botany*, 66:297–310.

Seddon, S., Miller, D., Fotheringham, D., Burgess, S., McKechnie, J. 2003. Beachport seagrass loss and links with Drain M in the Wattle Range Catchment. Prepared for the Coast Protection Board, Department for Environment and Heritage and the Environment Protection Authority. South Australian Research and Development Institute (Aquatic Sciences), Adelaide. Publication no. RD03/0190.

Shepherd, S. A. McComb, A. J., Bulthuis, D. A., Neverauskas, V., Steffensen, D. A. and West, R. 1989. Decline of seagrasses. In: Larkum, A. W. D., Mc Comb, A. J. and Shepherd, S. A. (Eds.)

Biology of Seagrasses. A Treatise of the Biology of Seagrasses with Special Reference to the Australian Region. Elsevier, Netherlands, pp. 346-393.
Shepherd S.A. and Robertson E.L. 1989. Regional studies - seagrasses of South Australia, Victoria and Bass Strait. *In:* Larkum A.W.D., McComb A.J. and Shepherd S.A. Eds. *Biology of seagrasses.* Elsevier, Amsterdam, pp. 211-229.
Shepherd S.A. and Sprigg R.C. 1976. Substrate, sediments and subtidal ecology of Gulf St Vincent and Investigator Strait. *In* Twidale, C.R., Tyler, M.J. and Webb, B.A. Eds. *Natural History of the Adelaide Region.* Royal Society of South Australia Inc., p. 189.
Short, F.T., Wyllie-Echeverria, S., 1996. Natural and human-induced disturbance of seagrasses. Environment and Conservation 23, 17–27.
Waycott, M., Duarte, C. M., Carruthers, T. J. B., Orth, R. J., Dennison, W. C., OlyarniK, S., Calladine, A., Fourgurean, J. W., Heck, Jr. K. L., Hughes, A. R., Kendrick, G. A., Kenworthy, W. J., Short, F. T. and Willaims, S. L. 2009. Accelerating loss of seagrasses across the globe threatens coastal ecosystems. *Proceedings of the National Academy of Sciences of the United States of America*. 106: 12377-12381.
Wear, R. J., Eaton, A., Tanner, J. E., Murray-Jones, S. (2006) The impact of drain discharges on seagrass beds in the South East of South Australia. Final Report Prepared for the South East Natural Resource Consultative Committee and the South East Catchment Water Management Board. South Australian Research and Development Institute (Aquatic Sciences) and the Department of Environment and Heritage, Coast Protection Branch, Adelaide. RD04/0229-3.
Westphalen, G., Collings, G., Wear, R., Fernandes, M., Bryars, S. and Cheshire, A. 2004. *A review of seagrass loss on the Adelaide metropolitan coastline.* ACWS Technical Report No. 2 prepared for the Adelaide Coastal Waters Study Steering Committee. South Australian Research and Development Institute (Aquatic Sciences), Publication No. RD04/0073, Adelaide.
Winterbottom, D.C. 1917. *Marine fibre*, Bulletin No. 4, Department of Chemistry, South Australia.

Appendix A. Sample computation for the extent and relative severity of environmental degradation for seagrass community.

Threshold of collapse (*a*): 0.026 mg/L of ammoniacal nitrogen (from Bryars et al. 2001)

Relative severity of degradation = $\left(\frac{b}{n}\right)\times 100$

Where: $\boldsymbol{b}$ = number of observed values (annual mean) that is greater than $\boldsymbol{a}$
$\boldsymbol{a}$ = the value inferred as the threshold of collapse
$\boldsymbol{n}$ = the total number of observation

Dataset for Brighton Bay, Gulf St. Vincent, South Australia:

Year	Ammoniacal Nitrogen (mg/L)*
1998	0.02925
1999	0.032375
2000	0.033875
2001	0.023933333
2002	0.018411765
2003	0.034714286
2004	0.018636364
2005	0.02415
2006	0.044
2007	0.009333333
2008	0.005

*Annual mean
Number of observed values greater than *a* (0.026 mg/L) = 5, total number of observations = 11
Relative severity of environmental degradation at Brighton Bay = 45.45% (5/11 x 100)

19 THE COORONG LAGOONS AND MURRAY MOUTH INVERSE ESTUARY, SOUTH AUSTRALIA

contributed by Rebecca E. Lester (Deakin University) and Peter G. Fairweather (Flinders University)

CLASSIFICATION

International and National: The coastal wetland complex represented by the Coorong supports numerous bird species that are listed under the Japan-Australia Migratory Bird Agreement, the China-Australia Migratory Bird Agreement and the Republic of Korea-Australia Migratory Bird Agreement (Phillips and Muller 2006). The Coorong, Lower Lakes and Murray Mouth region itself is a Wetland of International Importance under the Ramsar Convention (Phillips and Muller 2006), one of six identified icon sites under The Living Murray initiative, and a hydraulic indicator site under the draft Murray-Darling Basin Plan (MDBA 2010).

IUCN Habitats Classification Scheme (Version 3.0): 13 Marine Coastal/Supratidal / 13.4 Coastal Brackish/Saline Lagoons/Marine Lakes

ECOSYSTEM DESCRIPTION

Coastal wetlands can be relatively simple systems (e.g. riverine estuaries) or can include a complex of estuarine, marine and hypersaline habitats and biota that are often quite dynamic in space and time. This dynamic diversity of habitat types results in a diverse and unique characteristic biota and therefore high ecological values. The Coorong, which is the inverse estuary for the Murray-Darling Basin, Australia's largest river, is the sole example of such a large, diverse and complex ecosystem (Phillips & Muller 2006; Brookes *et al.* 2009; Kingsford *et al.* 2011), and so warrants assessment as a potentially-threatened ecosystem. The Coorong is part of a complex of freshwater, estuarine and hypersaline wetlands known as the Coorong, Lower Lakes and Murray Mouth (Figure 1). This case study focuses on the coastal lagoons that comprise the Coorong, and the Murray Mouth estuary, where the River Murray meets the sea. The upstream Lower Lakes are largely freshwater, being separated from the Coorong by a series of artificial barrages that control the flow of water between the two. The Lower Lakes therefore comprise a different characteristic biota to the Coorong and Murray Mouth, so are not included in this assessment, but may warrant separate consideration.

Characteristic native biota

The characteristic biota for such coastal wetland complexes includes a range of typically estuarine, marine and hypersaline taxa, and it is the combination of these that is one of the unique features of this ecosystem.

Characteristic habitat types include submergent macrophytes (*Ruppia megacarpa* and *R. tuberosa*, in particular but also including a range of seagrass species; Gehrig and Nicol 2010), littoral samphire shrublands, intertidal marshes, mudflats and emergent freshwater reeds (*Typha* spp. and *Phragmites australis*) (Figure 2; Phillips and Muller 2006). *Ruppia* spp., in particular, provide large areas of physical habitat and are a food source for many of the region's birds, fish and macroinvertebrates (Phillips and Muller 2006).

This ecosystem is particularly noted for the wide range of bird life that it supports (Brookes *et al.* 2009). Characteristic taxa include piscivores such as Australian pelican (*Pelecanus conspicillatus*; Figure 2) and cormorants (*Phalacrocarax* spp.), ducks (*Anas* spp.), waders (e.g. *Calidris ruficollis*, *Calidris acuminata*, *Charadrius ruficapillus*) and terns (e.g. *Sternula nereis*) (Phillips and Muller 2006).

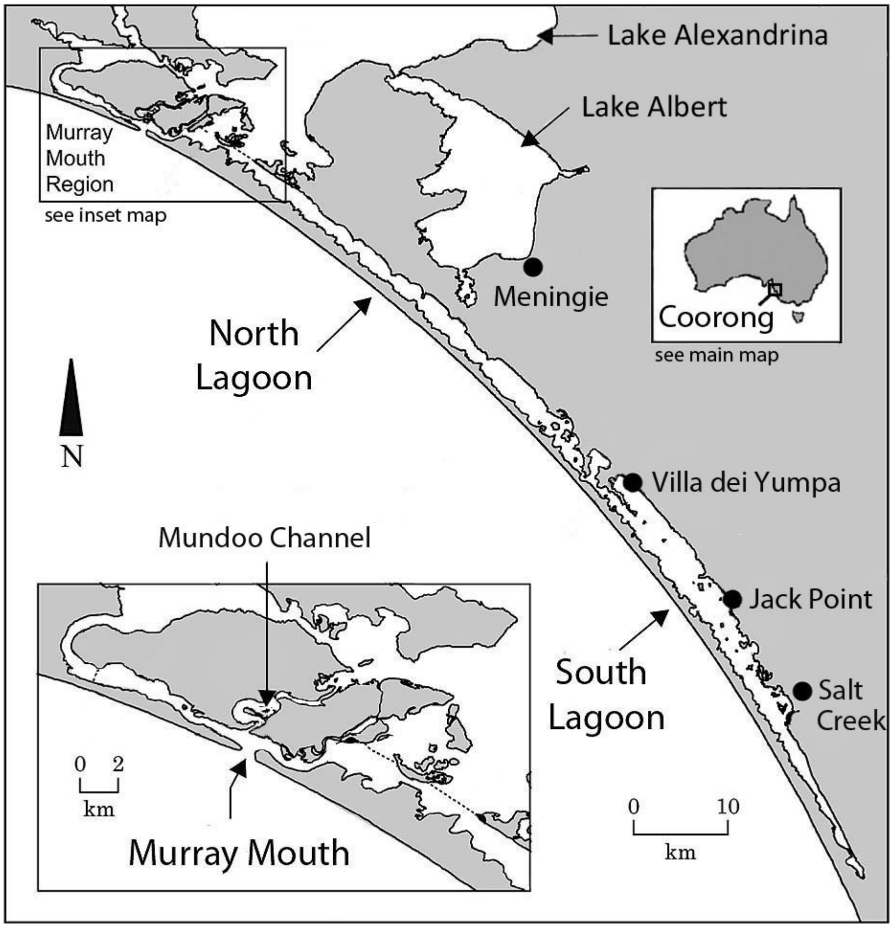

Figure S19. 1. Map of the Coorong and Murray Mouth region, including the North Lagoon, South Lagoon and the Murray Mouth (see inset map). Dotted lines on the inset map show positions of the barrages. Also shown is the location of the study region on a map of Australia. Locations mentioned within the text are shown, except for Victor Harbour, which is 25 km west of the Murray Mouth region shown here. Adapted from a map created by Craig Noell (SARDI Aquatic Sciences, South Australia).

Forty-nine species of fish have been described within this coastal wetland complex ecosystem, including diadromous species such as the culturally-significant congolli (*Pseudaphritis urvillii*), commercial and recreationally-fished yellow-eyed mullet (*Aldrichetta forsteri)* and black bream (*Acanthopagrus butcheri*), and small-mouthed hardyhead (*Atherinosoma microstoma*), which is an important prey item for many of the birds in the region (Phillips and Muller 2006).

Macroinvertebrate taxa occurring in the intertidal marshes and mudflats include Amphipoda, Oligochaeta, polychaete worms (e.g. *Nephtys australiensis*, *Simplesetia aequisetis*, *Capitella* spp.) and bivalves such as *Arthritica helmsi*. A calcareous tubeworm (*Ficopomatus enigmaticus*) and brine shrimp (*Paratemia zieziana*) are also characteristic.

In order to simplify the definition of characteristic biota for the Coorong, we use the suites of co-occurring biota that are described by the ecosystem state model for the region (Figure 3; Lester and Fairweather 2011). This is a data-derived state-and-transition model that identified eight distinct ecosystem states for the Coorong, including two 'basins of attraction' (i.e. a marine basin and a hypersaline basin) and a trajectory of ecosystem health in each (i.e. ranging from 'healthy' states to 'degraded' states in each basin) (Lester and Fairweather 2011).

The healthy state in the marine basin ('Estuarine/marine') is characterised by a large number of marine and estuarine fish (e.g. yellow-eyed mullet, mulloway (*Argyrosomus japonicas*), black bream and Australian salmon (*Arripis truttacea*). Characteristic birds in this state included cormorants, waterfowl, migratory waders and the Australian white ibis (*Threskiornis molucca*). Amphipoda and polychaete

species were characteristic of the infaunal assemblages, while the macrophyte *Ruppia tuberosa* was present (Lester and Fairweather 2011).

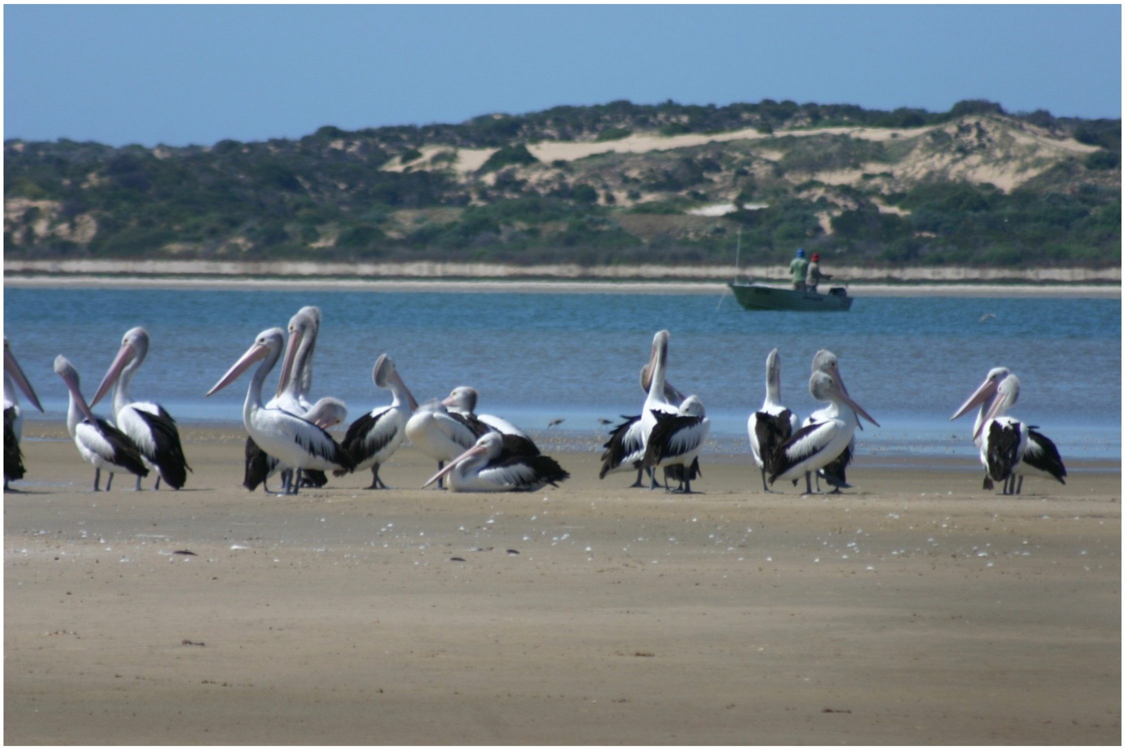

Figure S19. 2. Characteristic biota of the Coorong include samphire shrublands (in the distance) and Australian pelicans (in the foreground)

In the hypersaline basin, two healthy states were identified. The Healthy Hypersaline state is thought to be associated with times of high flow, and is characterised by waders (e.g. red-necked avocet *Recurvirostra novaehollandiae*), and waterfowl (e.g. teal, black swan *Cygnus atratus*), but with smaller numbers of waders and piscivores than were found in the marine basin (e.g. red-capped plover, red-necked stint and whiskered tern). This state supported few estuarine and marine fish, but infauna were characterised by polychaete worms and bivalves. The second healthy state in the hypersaline basin ('Average Hypersaline') had few macroinvertebrates other than chironomid (midge) larvae and Amphipoda. Few estuarine fish were again found, and Australian pelican was the only piscivorous species characteristic of this state. Waders and waterfowl again characterised the bird assemblage, with *Ruppia tuberosa* more common than in other hypersaline basin states.

Abiotic characteristics

A coastal wetland complex of the type described here consists of a long, narrow and shallow series of lagoons. The Coorong is 121 km long, with an average width of 1.9 km. It is divided in to two main lagoons with average depths of 1.2 and 1.4 m for the North and South Lagoons, respectively (Figure 1; S. Benger, Flinders University, pers. comm.; Webster 2010). The Murray Mouth is located at the northern end of the lagoon complex, adjacent to the major source of freshwater flows in the region, the barrages, which allow River Murray water to enter, making it an inverse estuary (*sensu* Wolanski 1987).

Salinities thus range from estuarine to hypersaline along the length of the system, and this spatial variability is one of the primary abiotic characteristics of the ecosystem (Lester *et al.* 2011a). Barrage flows (i.e. the flow of freshwater across the barrages), nearby sea levels in Encounter Bay, wind and a seasonal hydrologic disconnection between the North and South Lagoon are the main drivers of

complex seasonal and inter-annual patterns in salinity and water level throughout the region (Webster 2010). There is also a smaller input of fresh water in the South Lagoon, via Salt Creek.

The connection to the Southern Ocean, via the Murray Mouth, is highly dynamic, with the effective transmissivity of water (i.e. the amount of water that passes through the mouth as influenced by a combination of depth and width) varying seasonally and inter-annually, largely as a function of barrage flows (Webster 2010).

The ecosystem states model for the Coorong identified the key abiotic characteristics associated with each of the eight ecosystem states. These included the average daily tidal range (i.e. which was responsible for dividing the eight states into two basins), the number of no-flow days over the barrages (i.e. dividing the states into relatively healthy and unhealthy states), the annual average water level, annual average depth from two years previous (i.e. a lag in response time was detected, suggesting this was a leading indicator; Fairweather and Lester 2010) and the annual average salinity. This illustrates the interactions between water levels, flow and salinity that occur within the region thus giving it its unique abiotic characteristics.

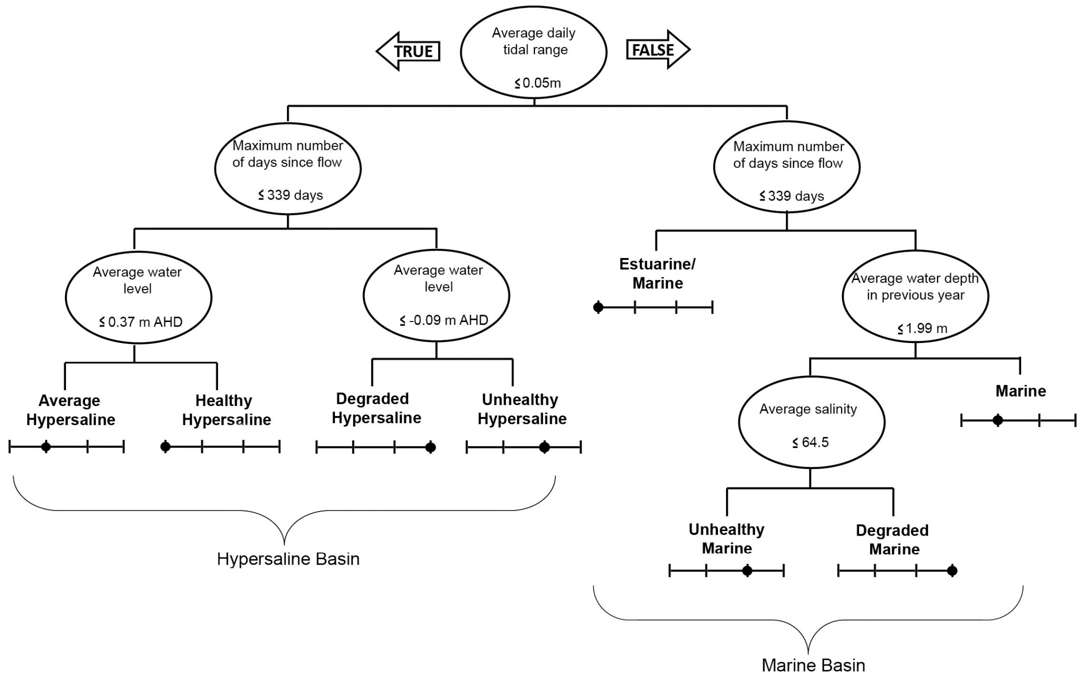

Figure S19. 3. Ecosystem states model for the Coorong (reproduced from Lester and Fairweather 2011) The states are presented here as a logic tree, where each oval should be read as a logic statement. For a given site-year, if the condition in the oval is true, then the tree should be followed to the left-hand side. If the condition is false, then the tree proceeds to the right, until a terminal node is reached. This terminal node determines which state the Coorong is in at any given location and time, based on its environmental characteristics. Under each state is a scale of degradation (from healthiest on the left to most degraded on the right), where a dot represents where each state fell along that trajectory (see Lester & Fairweather 2011).

Key processes and interactions

The key process occurring within this ecosystem is the flow of fresh water entering the system, which in the Coorong occurs from the River Murray over the barrages. This then interacts with mouth openness, water levels, local sea levels, wind, weather systems, flows via Salt Creek and evaporation

rates to drive the hydrodynamics of the Coorong lagoons (Webster 2010), which in turn, drive the ecological characteristics of the system (Figure 3; Lester and Fairweather 2011).

An environmental water regime for the region has been identified based on minimum freshwater flow volumes from the River Murray to support salinity targets in the upstream Lake Alexandrina (Heneker 2010). These have also been shown to support characteristic biota and abiotic conditions in the Coorong (Lester *et al.* 2011b). Heneker (2010) described three regimes of 3-year minimum barrage flow regimes, with different recommendations for the proportion of years where they apply (i.e. a long-term average of 700 µS cm^{-1}, a maximum of 1000 µS cm^{-1} in 95% of years, and an absolute maximum of 1500 µS cm^{-1} in 100% of years). The most relevant to this assessment is the water regime designed to support a salinity of 1000 µS cm^{-1} in Lake Alexandrina in 95% of years. To meet that target, minimum barrage flows in any one year should be the maximum of: i) 650 GL; ii) 4000 GL minus the flow volume of the previous year; and iii) 6000 GL minus the flow volumes of the previous two years (with a cap on the effective volume of the first year in that sequence) (Heneker 2010).

Furthermore, the rate and timing of delivery can have a large influence on the effect of freshwater flows, with prolonged low flows identified as more effective than short, larger flows to avoid high salinities and ecological degradation, for comparable volumes (Lester *et al.* 2011c).

Threatening processes

The primary threatening process to the Coorong ecosystem is thus a reduction of freshwater inflows from the River Murray. Under unregulated conditions, approximately 52% of flows (or a barrage flow of 10 764 GL $year^{-1}$) in the Murray-Darling Basin reach the sea, and thus pass through the Coorong (Kingsford *et al.* 2011). Under current water sharing arrangements, the average end-of-system flow is 3075 GL $year^{-1}$, which represents a 71% reduction. This loss of flow is disproportionately high during drought years, so that flows are now zero for 40% of days, compared with <1% under unregulated conditions (Kingsford *et al.* 2011). Thus, there is an interaction between extractions in the Murray-Darling Basin and drought, and there is likely to be an interaction between extractions and future climate change, as that develops within the region.

The second major threatening process is change to the source and timing of water delivery to the region. That is, while the overall volume may be sufficient, water may not be delivered from a suitable source, or with the timing required by the Coorong ecosystem. As described above, the timing of barrage flows has a large impact on conditions within the Coorong, making suboptimal delivery a potential threat.

Ecosystem collapse

Ecosystem collapse for this ecosystem type is defined conceptually as the loss of the range in estuarine, marine and hypersaline environments that have traditionally coexisted. Thus, it is effectively a loss of diversity of habitat types, rather than a complete loss of species. This is most likely to occur as a result of a loss of freshwater flows to the region that would increase salinity and decrease water levels and marine connectivity.

For the purposes of this case study, we have largely defined thresholds of ecosystem collapse for the Coorong, with respect to the ecosystem states model for the region. We define ecological collapse as occurring when half of the modelled years occur either in degraded ecosystem states or are in a period of recovery following the occurrence of degraded ecosystem states. Periods of recovery are defined as twice the duration of occurrence of degraded ecosystem states, as recovery from drought is likely to take significantly longer than recovery from flooding in freshwater systems (Lake 2000), which is also likely in estuarine systems, and the effect of high flow events is known to last for up to two years after the event (Heneker 2010).

This threshold is intended to capture both periods in which ecological degradation is likely (i.e. due to the occurrence of degraded ecosystem states) and the time required to recover characteristic biota following drought. The rationale for this is that long-term persistence of the characteristic biota for the region is highly unlikely if ecological degradation occurs frequently enough (here, defined at 50% of the time, as a starting point) without sufficient periods of recovery, and then ecological collapse is highly likely.

There are also parts of this assessment where the ecosystem states model is not applied, in an attempt to use multiple lines of evidence in the assessment. Therefore, alternative thresholds of ecological collapse were also required. In Criterion C, we assessed ecological condition relative to two alternative thresholds for salinity (i.e. an annual average of 117 g L^{-1} and an annual maximum of 100 g L^{-1}) in the South Lagoon that have been shown to be correlated with various forms of ecological degradation (Fairweather and Lester 2010; MDBA 2010), and against defined environmental water requirements for the Coorong (Lester *et al.* 2011e). Therefore, in Criterion C, we defined ecological collapse as occurring when the entire South Lagoon exceeded either threshold in all years within the model simulations used, or when environmental water requirements were not met in all model years.

In Criterion D, we again used an assessment independent of the ecosystem states model. We assessed the decline in the iconic and key macrophytes *Ruppia megacarpa* and *R. tuberosa*. There, we assumed that assemblages dependent on *Ruppia* spp. had collapsed when the percent coverage of *Ruppia* spp. declined to zero (see Criterion D).

ASSESSMENT

Summary

Criterion	A	B	C	D	E	Overall
Sub-criterion 1	LC	CR	VU	CR	CR(EN-CR)	CR
Sub-criterion 2	LC	EN	CR	DD		
Sub-criterion 3	DD	VU	DD	DD		

Criterion A

The spatial extent of the Coorong is largely geographically defined, and so changes little. Thus, criterion A is unlikely to elucidate changes in the risk of collapse for this case study.

Current decline: There have been no declines in the extent of the Coorong in the past 50 years. Declines in river flow and changes to salinity distributions are assessed further under criterion C. The status of the ecosystem is Least Concern under criterion A1.

Future decline: The extent of the Coorong is very unlikely to decline over the next 50 years or for any period of 50 years including the present to the future. This is because very low barrage flows would be compensated by more seawater flowing in through the Murray Mouth. Thus the status of the ecosystem is Least Concern under criterion A2.

Past decline: No estimate of a long-term reduction in spatial extent exists for the Coorong in the period since 1750. The status of the ecosystem is Data Deficient under criterion A3.

Criterion B

The Coorong is 121 km long, with an average width of 1.9 km, measured from the Goolwa Barrage to the bottom of the South Lagoon (S. Benger, Flinders University, pers. comm.). Areas of the North Lagoon (110.7 km^2) and South Lagoon (94.4 km^2) together are used to approximate the size of the Coorong (Phillips and Muller 2006).

Extent of occurrence: The extent of occurrence is less than 2000 km^2 (i.e. at 205 km^2), and there is evidence of increasing salinity in the Coorong since 1996, with extremely high salinities observed since 2006 (i.e. a decline in environmental quality; see Criterion C1). Further environmental declines (i.e. additional increases in salinity) are also forecast under median and dry future climate projections (see Criterion C2). In addition, barrage flows are predicted to fall under climate change simulations (Lester *et al.* 2011d), indicating that the processes threatening the Coorong ecosystem are likely to continue to cause declines in environmental condition in the next 20 years. Furthermore, the ecosystem occurs at only one location. Under these circumstances, the status of the ecosystem is Critically Endangered under Criterion B1a,b.

Area of occupancy: The number of 10 x 10 km grid cells occupied by the Coorong ecosystem has been calculated as 17 (S. Benger, Flinders University, pers. comm.). Together with the continuing and future declines in environmental quality described under Extent of occurrence, as well as its restricted occurrence at a single location, this indicates an Endangered status of the ecosystem under Criterion B2a,b.

Number of locations: The Coorong occupies a single location and is prone to the effects of both human activities (i.e. water extraction and changes to flow regimes upstream) and stochastic events (e.g. drought) simultaneously. The recent severe drought between 2006 and 2010 in the region illustrated the impact that the combination of human activity and drought could have on Coorong ecosystems (e.g. Brookes *et al.* 2009; Kingsford *et al.* 2011), demonstrating that the ecosystem is capable of becoming critically endangered within a short period of time (here, five years). Therefore, the status of the ecosystem is Vulnerable under criterion B3.

Criterion C

Two main components in the abiotic environment have been identified as reducing habitat quality for characteristic biota of the Coorong ecosystem: small volumes of fresh water delivered to the Coorong via the River Murray, and the extreme salinity of the South Lagoon.

The Environmental Water Requirements for the Coorong specify minimum rolling average volumes of annual barrage flows. Of the three target salinities for Lake Alexandrina, here we assess the likelihood of failing to deliver sufficient water to achieve an average of 1000 $\mu S\ cm^{-1}$, as this is the threshold that should not be exceeded in 95% of years. Failing to meet the rolling average volumes that are sufficient to meet that target would reduce habitat quality for characteristic biota (Lester *et al.* 2011e). As described above, collapse was defined as failing to meet environmental water requirements in all years included in the model simulation.

We also used two measures of salinity in the South Lagoon to assess the abiotic environment: average annual salinity and maximum annual salinity. Average annual salinity in the South Lagoon has been demonstrated to be a predictor of likely future ecological degradation in the Coorong based on the presence of degraded ecosystem states (Fairweather and Lester 2010). A threshold of 117 $g\ L^{-1}$ has been shown to predate ecological degradation by three years (Fairweather and Lester 2010). A similar threshold (100 $g\ L^{-1}$) has been suggested as the upper limit of suitable habitat for the iconic macrophyte *Ruppia tuberosa* (MDBA 2010). Thus, crossing either threshold, particularly for any length of time, represents a component of the abiotic environment that is likely to reduce habitat quality for characteristic biota. Here, ecological collapse was considered to occur when each threshold was exceeded for all years simulated.

Current decline: When barrage flows decline, the whole Coorong is affected, so the extent of impact where the thresholds are not met is always 100%. The relative severity was calculated as the proportion of years within the available modelling (see Criterion E for scenario definitions) under Current Conditions for which each site exceeds the threshold.

Under current extraction levels, existing water resources infrastructure and historical climatic conditions, hindcast between 1895 to 2008, minimum rolling-average barrage flow volume targets were

not met in 36% of years (Lester *et al.* 2011e). While this assessment extends for longer than the 50 years specified by Criterion C1, the hindcast cannot be thought of as an accurate simulation of change in the system since 1895, as current extraction levels and current water resources infrastructure are included for the entirety of the model run. Thus, the 114 years of the model run should be thought of as an assessment of possible but realistic variability due to climate, rather than a progression of deterioration (or otherwise) through time. As a result, we have included the whole model sequence in this analysis to indicate the likelihood that environmental water requirements would be met under an historical climate, given current management of the Basin. Thus, as flows were below threshold in 36% of years and this affects 100% of ecosystem extent, the status of the ecosystem Vulnerable under Criterion C1.

Both thresholds (a maximum salinity of 100 g L^{-1} and an annual average of 117 g L^{-1} in the South Lagoon) are calculated based on the whole of the South Lagoon; however, the three sites at which salinity is modelled in the South Lagoon (i.e. Villa dei Yumpa, Jack Point and Salt Creek) were assessed separately to estimate the extent of deterioration. The relative severity was calculated in the same manner as that for annual barrage flow volumes.

Under the same scenario as that used for barrage flow volumes, more than 30% (i.e. 1/3) sites in the South Lagoon exceeded an annual average salinity of 117 g L^{-1} for 18% of years (Table 1). However, all sites (i.e. >80%) exceeded the maximum salinity of 100 g L^{-1} for 41% of years (Table 1). This assessment also finds that the status of the ecosystem is Vulnerable under Criterion C1.

Table S18. 1. Average and maximum annual salinity for the South Lagoon and extent and severity of exceedance of thresholds for each under three alternative climate change projections (refer to Criterion E for additional information).

**Scenario**	**South Lagoon salinity (g L^{-1})**	**>80% of sites exceed threshold (% model years)**	**>50% of sites exceed threshold (% model years)**	**>30% of sites exceed threshold (% model years)**
Average annual salinity of 117 g L^{-1}				
Current Conditions	79	3%	17%	18%
Median Future	104	16%	39%	40%
Dry Future	201	81%	94%	94%
Maximum annual salinity of 100 g L^{-1}				
Current Conditions	102	41%	47%	50%
Median Future	140	71%	82%	84%
Dry Future	274	98%	99%	100%

Future decline: Under a median future climate projection for 2030 (Median Future; see Criterion E for a description of the scenarios used), with current extraction levels and existing water resources infrastructure, across 114 years of possible climate variability, minimum rolling-average barrage volume targets were not met in 45% of years (Lester *et al.* 2011e). Under a dry future climate projection for 2030 (Dry Future), again including current water management conditions and 114 years of possible climate variability resulted in failure to meet those minimum targets for barrage flow volumes in 86% of years. Under a median future climate, the status of the ecosystem would be considered Vulnerable, while under a dry future climate, it would be Critically Endangered.

Using the same median future climate scenario, no combination of extent and severity exceeded the values specified by Criterion C2 for an average annual salinity of more than 117 g L^{-1} (Table 1). However, all sites (i.e. >80%) exceeded the maximum salinity of 100 g L^{-1} for 82% of years (Table 1). This makes the status of the ecosystem Critically Endangered under Criterion C2.

A dry future climate projection, consistent with that used above, resulted in all sites exceeding the annual average salinity threshold of 117 g L^{-1} in 81% of years, and the maximum salinity of 100 g L^{-1} in 98% of years (Table 1), again making the status of the ecosystem Critically Endangered under Criterion C2, regardless of the degree of climate change experienced.

These assessments of the likelihood of future decline show some variability, depending on the future climate scenario used and the environmental variable assessed, but three of the four assessments result in a risk level of Critically Endangered for Criterion C2. Also important to note is that future climatic conditions in these assessments extend only until 2030, not for an additional 30 years, so the likelihood of degradation would probably be higher after the 50 year duration. Therefore the higher estimate of risk is likely to better represent risks over the next 50 years.

Past decline: No estimate of a long-term change in South Lagoon salinities the period since 1750 exists. The status of the ecosystem is Data Deficient under criterion C3.

Criterion D

Ruppia spp. are thought to be a critical component in the structure and functioning of the Coorong and Murray Mouth. Historically, there have been two species present (*R. megacarpa* and *R. tuberosa*). *Ruppia* spp. allow for characteristic biotic interactions through the provision of food and habitat resources for birds (e.g. black swan, *Calidris* spp.), fish (e.g. small-mouthed hardyhead) and macroinvertebrates (e.g. chironomids) (Rogers and Paton 2009). *Ruppia* spp. also modify physical and biogeochemical processes in the Coorong (Rogers and Paton 2009). Therefore, the spatial coverage of *Ruppia* spp. is likely to act as a surrogate measure for changes in some characteristic biotic interactions in the system. As described above, for the purpose of this analysis, it was assumed that the ecosystem reaches a state of collapse when the abundance of *Ruppia* spp. declines to zero.

Current decline: *Ruppia megacarpa* once dominated the submerged macrophyte assemblage of the Murray Mouth and North Lagoon (Gehrig and Nicol 2010). However, its range has declined due to the near-closure of the Murray Mouth and ongoing low barrage flows, which have increased salinities, and so the plant has not been observed in the Coorong since the mid-1990s (Gehrig and Nicol 2010). *Ruppia tuberosa* has traditionally dominated in the South Lagoon. In 1999, 33 to 91% of cores taken from four sites in the South Lagoon contained *R. tuberosa* shoots. In 2005, *R. tuberosa* was no longer present at the two southernmost sites, and by 2008, no South Lagoon sites sampled were found to contain *R. tuberosa*, although it had begun to colonise the North Lagoon by 2005 where it had not previously been recorded (Rogers and Paton 2009). Similar changes were observed for the occurrence of seeds and turions (Rogers and Paton 2009). Despite this colonisation, it is not likely that *R. tuberosa* was functionally replacing *R. megacarpa*, as the two species are morphologically different and are thought to support different assemblages. While some colonisation of the North Lagoon was observed for *R. tuberosa*, the extent and severity of the decline in both species exceeded 80% with *R. megacarpa* now locally extinct, making the Coorong ecosystem Critically Endangered under Criterion D1.

Future decline: No simulations for future declines in *R. tuberosa* exist, while *R. megacarpa* is already extinct from the system, and no evidence of recolonisation has been observed. Thus, the state of the Coorong ecosystem under this assessment would be Data Deficient.

Past decline: No estimate of long-term changes biotic interactions exists. The status of the ecosystem is Data Deficient under Criterion D3.

Criterion E

The ecosystem states model for the Coorong has been used to simulate hundreds of scenarios of possible future climate, water extraction and management options (e.g. Lester *et al.* 2009, 2011d). Among these scenarios are a number that are directly relevant for the assessment of the likelihood of ecosystem collapse in the next 50 or 100 years.

Scenarios that are likely to be of relevance include those that investigate the likely effect of future climate change (i.e. to 2030) and the effect of water extractions at the current level of take. The potential impact of the current draft Murray-Darling Basin Plan (MDBA 2010) is also relevant, but at this time, the modelled scenarios for water delivery under that Plan are not publically available for analysis.

Hydrologic, hydrodynamic & ecosystem states modelling
The quantitative assessment of the likelihood of ecosystem collapse in the Coorong was undertaken using a chain-of-models approach summarised in Lester *et al.* (2011d). Here, down-scaled simulations from multiple global climate models were applied to hydrologic models for the Murray-Darling Basin to estimate a time series of flows for each of the scenarios investigated (see below) (CSIRO 2008).

The output from the hydrologic modelling, along with sea levels at Victor Harbour and Meningie, flows via Salt Creek, precipitation and evaporation at Mundoo Channel and wind at Meningie were used as input for a hydrodynamic model of the Coorong (Webster 2010). This model simulated water levels and salinities along the length of the Coorong (Webster 2010). Refer to Webster (2010) for details of the model calibration and validation.

The time series of barrage flows and the outputs from the hydrodynamic model were then used as input to the ecosystem state model (Lester and Fairweather 2011). The average daily tidal range, number of days with no barrage flows, annual average water level, annual average water depth (with a two-year lag) and the annual average salinity were calculated and a series of ecosystem states was simulated for each year at each site for each scenario. This series of ecosystem states was then analysed to identify the likelihood of ecosystem collapse according to the above definition. Refer to Lester and Fairweather (2011) for model development details and relevant caveats.

Scenarios investigated
Six scenarios were investigated to quantify the likelihood of ecological collapse in the Coorong. We used three potential future climate projections for 2030 (i.e. the historical sequence since 1895; the median future climate projection based on three climate change scenarios from 15 global climate models; and a dry future climate projection based on the 10th percentile of the same 45 climate simulations; Chiew *et al.* 2008). Two extraction levels were used (i.e. with and without current infrastructure and extraction levels, with the latter approximating ‘natural’ conditions). All scenarios ran for a period of 114-years. The combinations of each are summarised in Table 2. Refer to Lester *et al.* (2009) for further detail.

Table S18. 2. Definition of the six scenarios investigated

Scenario	Climate	Extraction levels
Current Conditions	historic	+
Without Development	historic	-
Median Future	median	+
Dry Future	dry	+
Median Without Development	median	-
Dry Without Development	dry	-

Note ‘+’ denotes either at current levels and ‘-‘ indicates none. Refer to Lester *et al.* (2009) for additional detail regarding the scenarios modelled.

Likelihood of ecological collapse
In order to calculate the likelihood of collapse for each scenario, the number of years in which degraded ecosystem states were observed for the Coorong under each scenario was counted. Then, the length of each period of degradation and the number of years required to recover from that degradation (i.e. defined as twice the period of degradation, see above) were calculated. These were combined to determine the total proportion of years in which the Coorong was either in a degraded ecological condition, or was recovering from a previous period of degradation. Given that each scenario should be interpreted as 114 years of possible variability due to climatic fluctuations, rather than a realistic

progression through time, using the proportion of years occurring in degraded or recovery states effectively provides an assessment of the stochasticity within the system. Where a second period of degradation occurred prior to complete recovery from an initial period of degradation, the remaining period of recovery was added to the period required to recover from the second period of degradation. Scenarios where the definition of collapse was met were identified. Where ecological collapse was not identified, the likelihood of collapse was calculated as the number of years in which the Coorong was in a period of degradation or recovering from such a period divided by half the length of the model run (i.e. the number of years to reach 50% of degraded or recovery years as per the definition above).

Of the six scenarios investigated, ecological collapse was simulated to occur in four (Figure 4). A dry future climate projection with current extraction levels was likely to result always in ecological collapse. Scenarios with water development but using either a median future climate projection or the historical climate did not ever meet the definition for ecological collapse. However, at current extraction levels, the probability of ecosystem collapse under a future climate that resembles the historical climate was 30%, while the probability of ecosystem collapse under a median future climate change projection was 61%. Under quasi-natural conditions (i.e. without extractions or water resources infrastructure), the likelihood of collapse was 0%, except under a dry future climate projection, when it was 4%.

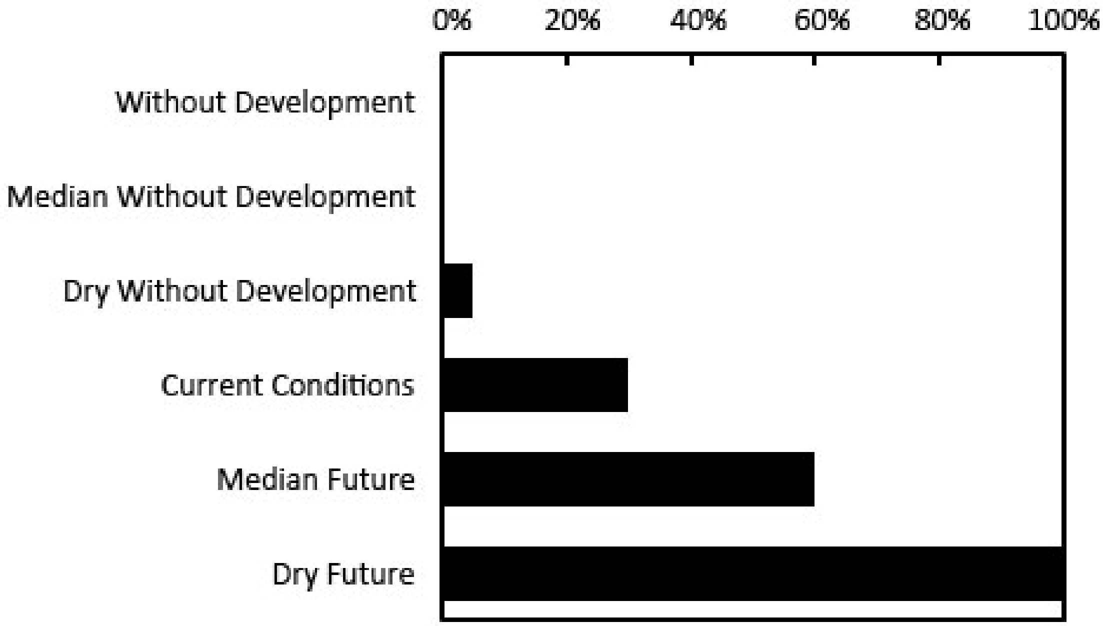

Figure S19. 4. Likelihood of collapse under the six scenarios investigated. Refer to text for definition of each scenario.

While we recognise that completely removing extractions and water-resource infrastructure within the Murray-Darling Basin is impossible, modelling such scenarios provide insight into the amount of degradation that is inherent due to climate change, compared with that caused by extraction levels. Clearly here, extraction levels play the major role in increasing the likelihood of ecological collapse. This means that the upcoming Murray-Darling Basin Plan, and other future changes to water extraction levels and water-resource infrastructure in the Basin, have the potential to dramatically change the likelihood of ecological collapse, so provide a mechanism for addressing threat levels identified here.

One limitation to scenario modelling is that there is rarely an analysis of the likelihood of the different scenarios occurring (Sutherland 2006). This means that it is difficult to identify a single quantitative assessment of the likelihood of ecological collapse, as that assessment depends on which scenario is chosen as the basis of that analysis. However, some estimates of likelihood can be assigned to each of

the scenarios. For example, there is no likelihood that water extractions will cease altogether, so the ‘without development’ scenarios can be discounted from an overall calculation. Thus, three scenarios remain, investigating the likelihood of ecological collapse under current extraction levels under the three climate change projections. Across those three scenarios, the likelihood of ecological collapse ranges from 30% to 60% to 100%. This means that, according to Criterion E, the Coorong is Critically Endangered (plausible range Endangered-Critically Endangered).

Furthermore, the climate simulations included here reflect the median and 10th percentile projections for 2030 climate within the Murray-Darling Basin. Again, for the scenarios using projections for a 2030 climate, the 114 years should be thought of as stochasticity inherent in the Basin-wide climate, rather than a progression from the current climate to a future climate. That is, a possible 2030 climate is simulated for the entire 114 years, with extraction levels and water resources infrastructure held constant throughout. Thus, any assessment of the likelihood of collapse is based on that collapse occurring under conditions likely to occur within the next 18 years, because the climate simulation is for 2030, rather than for a full 50 or 100 year period, as specified by Criterion E. Thus, this assessment is conservative, as the climate in 2062 or 2112 (the required time frames for assessment of criterion E) is highly likely to be more severe than that projected for 2030, and the likelihood of ecological collapse would also be greater than projected for 2030, making it likely that the Coorong is Critically Endangered.

Acknowledgments

We thank Simon Benger for GIS support and Ben Hamilton for research assistance, as well as all the people listed in the Acknowledgments sections of our references cited below for assisting with development of these models.

REFERENCES

Brookes JD, Lamontagne S, Aldridge KT, Benger S, Bissett A, Bucater L, Cheshire AC, Cook PLM, Deegan BM, Dittmann S, Fairweather PG, Fernandes MB, Ford PW, Geddes MC, Gillanders BM, Grigg NJ, Haese RR, Krull E, Langley RA, Lester RE, Loo M, Munro AR, Noell CJ, Nayar S, Paton DC, Revill AT, Rogers DJ, Rolston A, Sharma SK, Short DA, Tanner JE, Webster IT, Wellman NR & Ye Q. 2009. *An Ecosystem Assessment Framework to Guide Management of the Coorong. Final Report of the CLLAMMecology Research Cluster.* CSIRO Water for a Healthy Country Flagship Project, Canberra, 61 pp.

Chiew F, Teng J, Kirono D, Frost A, Bathols J, Vaze J, Viney N, Hennessy K and Cai W. 2008. *Climate change data for hydrologic scenario modelling across the Murray-Darling Basin*. A report to the Australian Government from the CSIRO Murray-Darling Basin Sustainable Yields Project. CSIRO, Canberra, 35 pp.

CSIRO. 2008. Water availability in the Murray. A report to the Australian Government from CSIRO Murray-Darling Basin Sustainable Yield Project. CSIRO, Canberra. 68 pp.

Fairweather PG and RE Lester (2010) Predicting future ecological degradation based on modelled thresholds. *Marine Ecology Progress Series* **413**: 291-304.

Gehrig S and Nicol J. 2010. *Aquatic and littoral vegetation of the Murray River downstream of Lock 1, the Lower Lakes, Murray Estuary and Coorong. A literature review.* South Australian Research and Development Institute (Aquatic Sciences), Adelaide, Australia. SARDI Publication Number F2010/00297-1. SARDI Research Report Series No. 482, 65 pp.

Heneker TM. 2010. *Development of Flow Regimes to Manage Water Quality in the Lower Lakes, South Australia*. Department for Water, Government of South Australia, Adelaide, 98 pp.

Kingsford RT, Walker KF, Lester RE, Young WJ, Fairweather PG, Sammut J and Geddes MC. 2011. A Ramsar wetland in crisis – the Coorong, Lower Lakes and Murray Mouth, *Australian Marine and Freshwater Research* **62:** 255-265.

Lake PS. 2000. Disturbance, patchiness and diversity in streams. *Journal of the North American Benthological Society* **19**: 573-592.

Lester RE and Fairweather PG. 2011. Ecosystem states: Creating a data-derived, ecosystem-scale ecological response model that is explicit in space and time. *Ecological Modelling* **222**: 2690-2703.

Lester RE, Webster IT, Fairweather PG and Langley RA. 2009. *Predicting the future ecological condition of the Coorong. The effect of management actions & climate change scenarios.* CSIRO: Water for a Healthy Country National Research Flagship, 104 pp.

Lester RE, Fairweather PG, Muller KL and Higham JS. 2011a. Describing a "healthy, productive and resilient wetland of international importance": Description of ecological objectives and associated outcomes. In: Lester RE, Fairweather PG and Higham JS *Determining the environmental water requirements for the Coorong, Lower Lakes and Murray Mouth region. Methods and Findings to date.* A report prepared for the South Australian Department of Environment and Natural Resources, Adelaide, Australia. pp. 29-33.

Lester RE, Fairweather PG and Webster IT. 2011b. Describing an environmental water requirement for the Coorong. In: Lester RE, Fairweather PG and Higham JS *Determining the environmental water requirements for the Coorong, Lower Lakes and Murray Mouth region. Methods and Findings to date.* A report prepared for the South Australian Department of Environment and Natural Resources, Adelaide, Australia. pp. 357-372.

Lester RE, Webster IT and Fairweather PG. 2011c. Hydrodynamic impacts of environmental water allocation in the Coorong. In: Lester RE, Fairweather PG and Higham JS *Determining the environmental water requirements for the Coorong, Lower Lakes and Murray Mouth region. Methods and Findings to date.* A report prepared for the South Australian Department of Environment and Natural Resources, Adelaide, Australia. pp. 292-334.

Lester RE, Webster IT, Fairweather PG and Young WJ. 2011d. Linking water-resource models to ecosystem-response models to guide water-resource planning – an example from the Murray-Darling Basin, Australia. *Marine and Freshwater Research* **62**: 279-289.

Lester RE, Langley RA, Fairweather PG and Webster IT. 2011e. Exploring the implications of delivering less water than specified by the EWR. In: Lester RE, Fairweather PG and Higham JS *Determining the environmental water requirements for the Coorong, Lower Lakes and Murray Mouth region. Methods and Findings to date.* A report prepared for the South Australian Department of Environment and Natural Resources, Adelaide, Australia. pp. 373-395.

MDBA. 2010. *Guide to the proposed Basin Plan: Overview*, Murray Darling Basin Authority, Canberra. 223 pp.

Phillips B and Muller K. 2006. *Ecological Character Description: Coorong, Lakes Alexandrina and Albert, Wetland of International Importance.* A report prepared for the South Australian Department for Environment & Heritage, Adelaide, Australia. 336 pp.

Rogers DJ and Paton DC. 2009. *Changes in the distribution and abundance of Ruppia tuberosa in the Coorong*. CSIRO: Water for a Healthy Country National Research Flagship, Canberra. 23 pp.

Sutherland GD. 2006. Predicting the ecological consequences of environmental change. a review of the methods. *Journal of Applied Ecology* **43**: 599-616.

Talbot FH, Russell BC and Anderson GRV. 1978. Coral reef fish communities: unstable, high-diversity systems? *Ecological Monographs* **48**: 425-440.

Webster IT. 2010. The hydrodynamics and salinity dynamics of a coastal lagoon – The Coorong, Australia – Seasonal to multi-decadal time scales. *Estuarine, Coastal and Shelf Science* **90**: 264-274.

Wolanski E. 1987. An evaporation-driven salinity maximum zone in Australian tropical estuaries. *Estuarine, Coastal and Shelf Science* **22**: 415-424.

20 KARST RISING-SPRING (KRS) WETLAND COMMUNITY OF THE SOUTH EAST (SA)

contributed by Ronald Bonifacio and Phil Pisanu, Science Resource Centre, Department of Environment and Natural Resources, South Australia, May 18, 2012

CLASSIFICATION

International: Not classified.
IUCN Habitats Classification Scheme (Version 3.0): 5. Wetlands (inland) / 5.18 Karst and Other Subterranean Inland Aquatic Systems

ECOSYSTEM DESCRIPTION

Characteristic native biota

A number of wetland plant associations occur within the Karst Rising Spring (KRS) Wetland Community in the south east region of South Australia. These include vegetation associations of the spring pools and those of the peripheral peat fens. They consist of reedbeds, sedgelands, *Melaleuca squarrosa* shrublands and Silky Tea-tree wet shrublands (Eardley 1943; Bachmann 2004; Ecological Associates 2008). Of these only the permanently submerged associations of the spring pools can be considered unique to the community based on current information. The associations of the peripheral peat fen generally also occur within other wetland types in the Gambier Basin region.

The spring pools of the KRS Wetland Community support vegetation that is unique to this ecosystem. The vegetation typically occurs as distinct zones, all of which may or may not be present, that occupy different depth bands and are described as follows:

- Pool margins are characterised by a peripheral band of dense reeds, tall sedges and rushes (+/- *Cladium procerum*, *Typha domingensis*) (Ecological Associates 2008) and may feature *Phragmites australis* and *Schoenoplectus validus*. Vegetation is usually dense, with a projected cover of 80 to 100 % and an average height of ~2 m (Ecological Associates 2008).
- At depths immediately below those occupied by peripheral reeds and sedges there may be a band of floating-leaved aquatic vegetation where Water-ribbons (*Triglochin procerum*) and Sea Tassel (*Ruppia polycarpa*) are commonly found (Scholz 1990; Thurgate 1995), as well as Pennywort (*Hydrocotyle* spp) and River Buttercup (*Ranunculus* sp). At some locations (e.g. Piccaninnie Ponds) the area of suitable depth for this vegetation is quite broad and extensive beds occur.
- There is generally an absence of vascular plants from depths approximately below 6 m where the common vegetation is algae including *Enteromorpha intestinalis*, *Batrachospermum boryanum* (a red alga) and the genera *Oscillatoria*, *Anabaena* and *Lyngbya*. Outcropping limestone on the floor of spring pools may feature the mosses *Cratoneuropsis relaxa* and *Fissidens rigidulus* (Thurgate 1995). An exception is at Piccaninnie Pond where the vascular plant community occurs on its steep side at considerable depth, with *Myriophyllum propinquum* (Common Water-milfoil) present to 7 m and *Potamogeton pectinatus* (Fennel Pondweed) to 15 m (Scholz 1990; Thurgate 1995). Pennywort (*Hydrocotyle* spp) and River Buttercup (*Ranunculus* sp) may also be present in this zone and Scholz (1990) mentioned the presence of lichens unique to this EC.
- Vegetation found in the surrounding spring-fed peat fen may contain reedbeds, sedgelands and mid closed shrublands but are not the distinctive plant associations of KRS. However, Silky Tea-tree (*Leptospermum lanigerum*) Wet Shrubland remnant is commonly found associated with the KRS Wetland Community.

The KRS provides resources and habitats to several vertebrate fauna but these are also found in surrounding habitat (see Appendix A). Stygofauna are known to occur in these systems as well as commonly occurring native fishes such as: Common Galaxias (*Galaxias maculatas*); Dwarf Galaxias (*Galaxiella pusilla*); River Blackfish (*Gadopsis marmoratus*); Short-finned Eel (*Anguilla australis*); Southern Pygmy Perch (*Nannoperca australis*); and Variegated Pygmy Perch (*Nannoperca variegata*) (Hammer 2002). The KRS Wetland Community of the South East is the only known habitat for the Glenelg Spiny Freshwater Crayfish (*Euastacus bispinosus*) in South Australia. Some of the terrestrial fauna species associated with this EC are the State-rated (EN) Bright-eyed Brown Butterfly (*Heteronympha cordace wilsoni*) and Ground Parrot (*Pezoporus wallicus wallicus*).

Abiotic environment

The distinguishing feature of this ecological community is the presence of surface expression of groundwater with sufficient head pressure to push water above the seal of the pool resulting in flows at any given time of the year. The water quality in KRS exhibits minimal seasonal variation, temperature is typically low (approx. 6°C - 18°C), turbidity is extremely low and conductivity is low to moderate (approx. 1000 – 3500 $\mu S.cm^{-1}$) (Hammer 2008).

The lower South East of SA has been referred to as a karst province (Marker 1975). The surface soils of the region are underlain by a band of limestone of tertiary marine origin known as the Gambier Limestone (Thurgate 1995). The Gambier Limestone ranges in thickness from several metres in the north to over 300 m at the coast (Harris 1983). The Gambier Limestone contains the tertiary limestone aquifer, commonly referred to as the ‘unconfined’ aquifer. The unconfined aquifer is thought to be recharged locally via the direct infiltration of rainfall (SKM 2009). The Gambier Limestone is underlain by a Tertiary Aquitard of low permeability. Beneath the Tertiary Aquitard is the Dilwyn Formation, which contains a lower Tertiary Confined Sand Aquifer, which is thought to be recharged in parts of the lower South East and western Victoria (SKM 2009).

The Gambier Limestone features extensive karst development (Harris 1983). Karst features form via the dissolution of the limestone matrix by water that has become slightly acidic due to the dissolution of carbon dioxide (Thurgate 1995). Spring pools are components of the KRS Wetland Community but sinkhole lakes are not. At spring pools, groundwater discharges from the karst feature into the surrounding landscape, whereas water does not discharge from sinkhole lakes. Due to spring discharge the native vegetation surrounding spring pools is typically wetland vegetation and hydrological connectivity may exist between adjacent spring pools or the sea. It should be noted that there are a number of spring pools on the coastal fringe of the Limestone Coast for which flow has ceased, either permanently or seasonally, due to anthropogenic influence (Bachmann 2002). These spring pools are regarded as degraded examples of the ecosystem.

A number of KRS Wetland Community sites feature artificial drains, such as the Eight Mile Creek and Deep Creek, that divert groundwater into the greater regional drainage network and ultimately to the sea. Despite their artificial nature, these drains have ecological values that are consistent with their inclusion as part of the KRS ecological community. They are in effect linear extensions of the spring pool habitat described above, featuring many of the same physical and biological components (Hammer *et al.* 2004; Hammer 2008). The water quality within these drains is also excellent and generally similar to that within the spring pools, providing a lotic habitat type that is rare at the regional, state and national level (Hammer *et al.* 2004). They also provide important hydrological connectivity between the spring pools and the sea, which is essential for particular faunal groups, such as diadromous fish species, which require access to both marine and freshwater to complete their life cycles.

Distribution

The KRS Wetland Community occurs in the complex Pleistocene sediments of the Bridgewater Formation and the Tertiary Gambier Limestone Formation of the limestone coast (lower south east) of South Australia from the township of Robe to the Victorian border (Figure 1). Cann et al. (1991, p 164) described the Bridgewater Formation as predominantly consisting of “aeolian bioclastic calcarenites with some seaward horizons of shelly limestone in which the fossil molluscs can be associated with rocky foreshore sedimentation.” The Bridgewater Formation forms a stranded series of elongated beach ridges, subparallel to the present coast. The Gambier Limestone Formation is an extensive shallow-water shelf carbonate of Eocene to Miocene age (James and Bone 1989). The KRS Wetland Community is confined in geographic extent to the Gambier Basin.

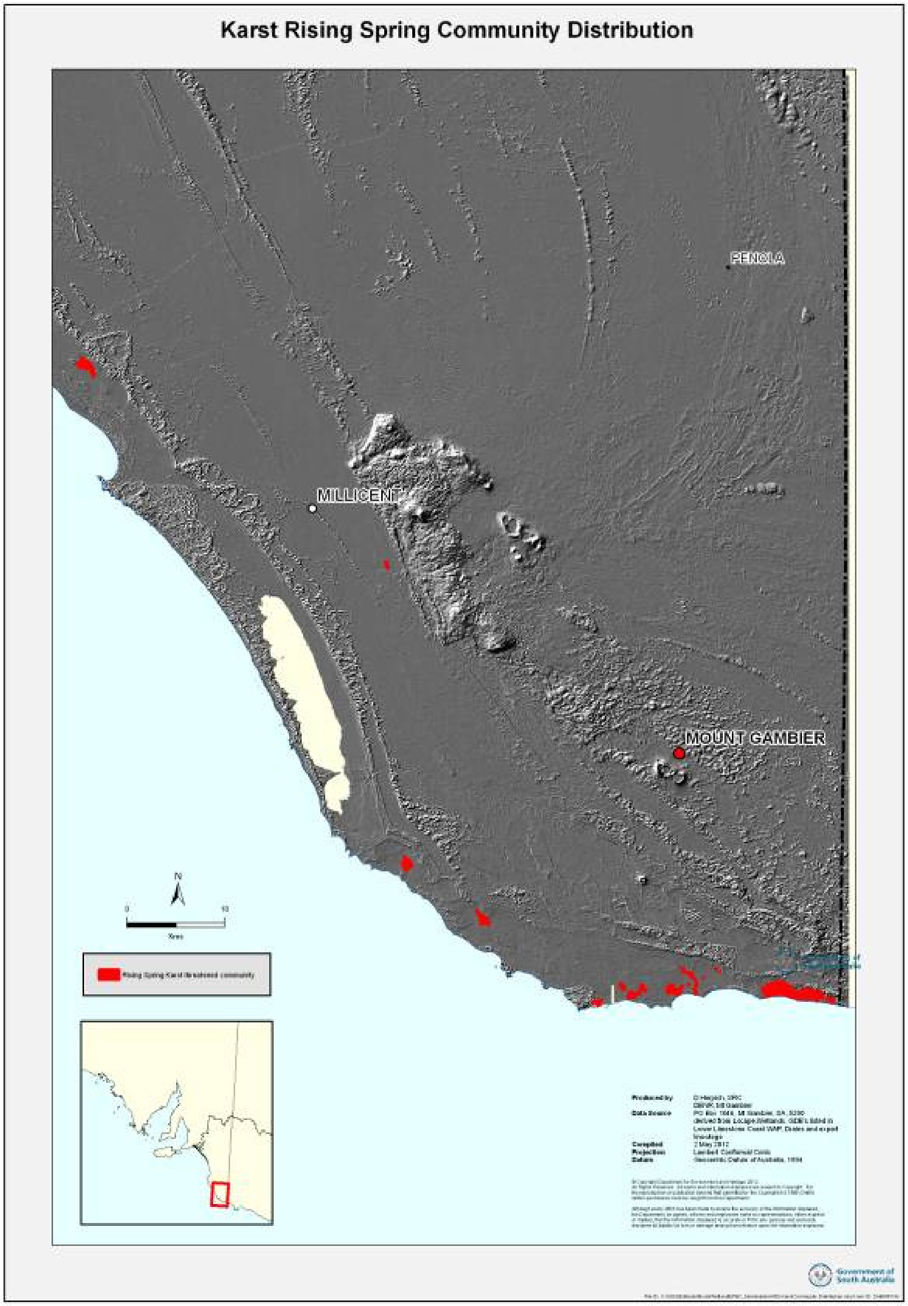

Figure S20. 1. Distribution of Karst Rising Spring Wetland Community in South East of South Australia.

Key processes and interactions

The formation of cavities in the Karst Rising-Spring Wetland Community was predominantly from dissolution of the limestone with the bedrock largely intact (Butcher et al. 2011). For example, "The Piccaninnie Ponds karst wetlands originally formed as dry surface depressions (dolines) where rainwater seeped into the joints of the limestone which were gradually widened by dissolution. Some cracks would have widened faster than others leading to the development of a void and subsidence of the surrounding land (Hallam and Thurgate 1992). When the rate of dissolution is greater than accumulation of insoluble material in the depression the master joint will deepen creating a funnel. Pressurised groundwater was forced up into the dolines via the master joints leading to the creation of the chasms through a combination of continued dissolution and scouring from the upwelling groundwater" (Butcher et al. 2011, p 102). The processes involved in the formation of the Piccaninnie Pond are illustrated in Figure 2. Though the diagrammatic model was specifically drawn to illustrate the processes involve in the formation of Piccaninnie Ponds, the general description (the dissolution of limestone and the subsequent expression of pressurised groundwater) is applicable to all KRS Wetland Communities. The spring pools and the water flow support a diverse flora and fauna.

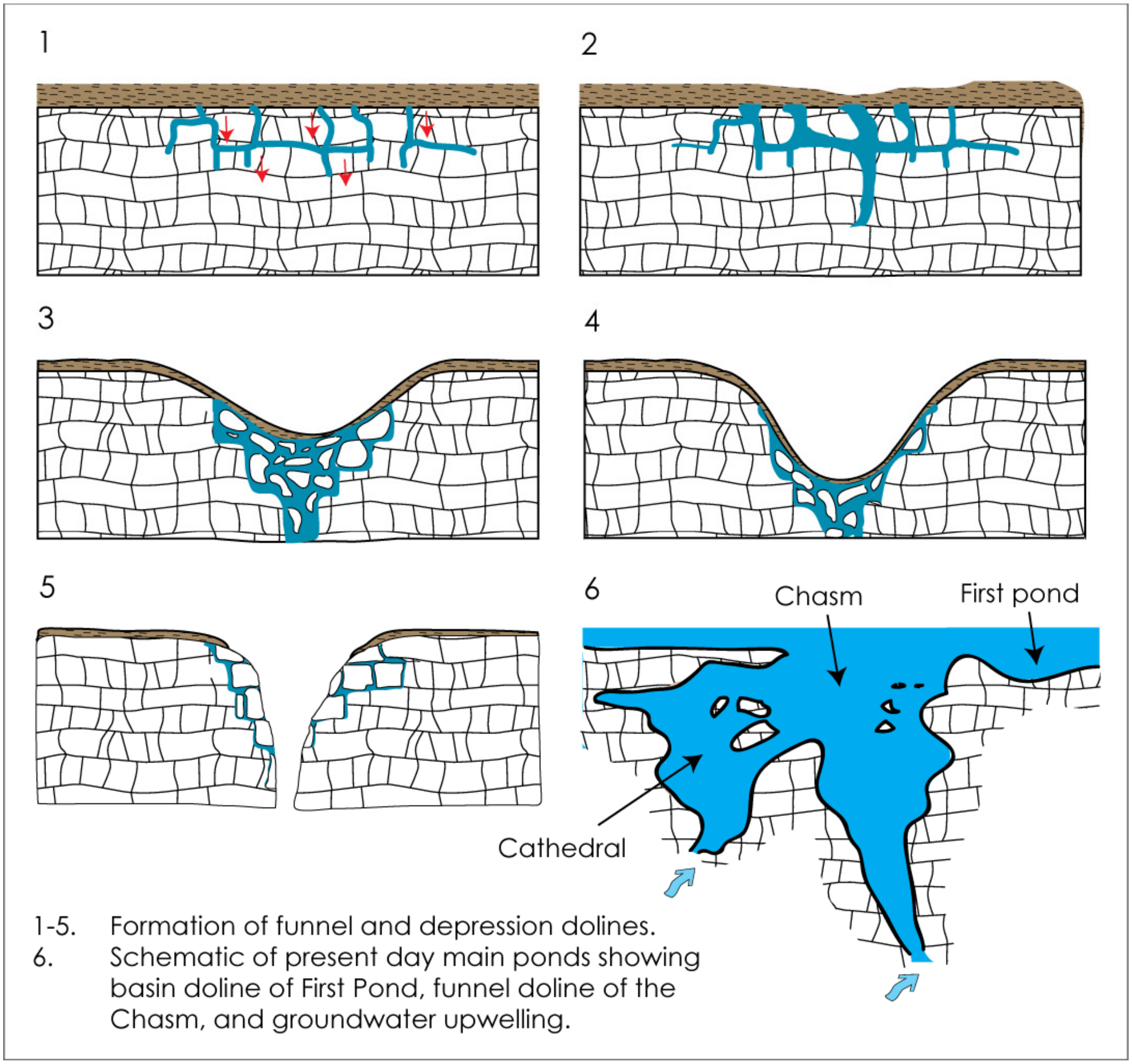

Figure S20. 2. Formation of karst wetlands at Piccaninnie Ponds (after Hallam and Thurgate 1992).

A summary of components, processes and services of the KRS Wetland Community is shown in the conceptual model below (Figure 3). Again, this model specifically used Piccaninnie Pond as an example but is generally applicable to KRS Wetland Community as a whole.

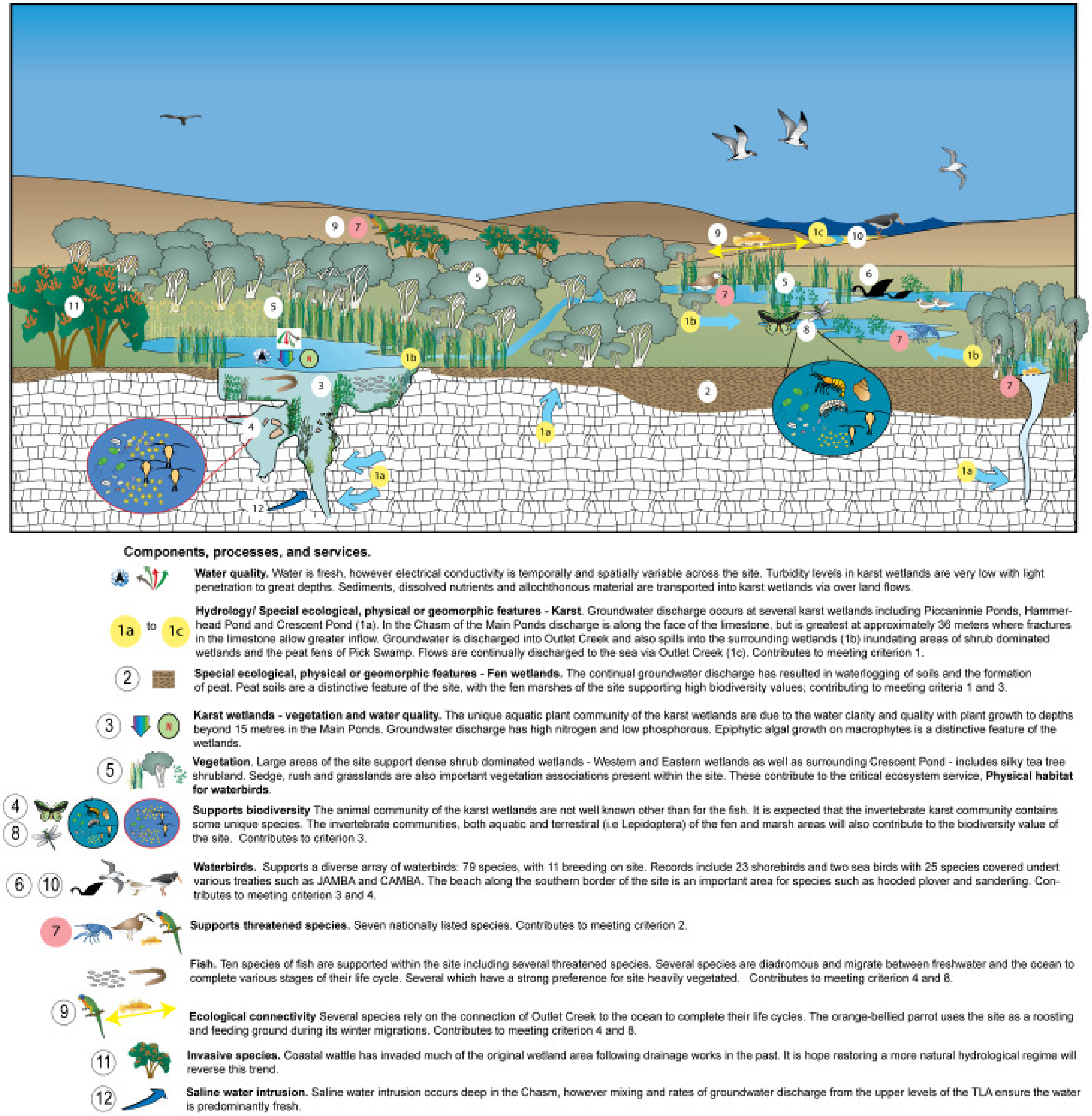

Figure S20. 3. Conceptual model of critical components, processes and services of Karst Rising-Spring Wetland Community. (source Butcher et al. 2011).

Threatening processes

Past threats include: land clearance and drainage (actual). Current threats include: groundwater extraction for irrigated agriculture (actual); reduced water quality (actual); mechanical dragging of

drains (actual); sea water intrusion (actual); climate change (actual – e.g. decrease rainfall; increase temperature); introduced species. Future threats are likely to include: expansion of groundwater extraction for irrigated agriculture (potential); land use change, particularly conversion from grazing to plantation forestry (potential); introduced species (potential); and climate change (actual – e.g. sea level rise).

Land Clearance and Drainage
The South Australian *Native Vegetation Act 1991* essentially brought an end to broad-scale clearance of native vegetation in the state. Thus clearance is a past threat and not likely to occur at KRS site in the future. Prior to World War 2 much of the KRS was intact. Soldier settlement led to the clearance and drainage of large areas of the APCL from the late 1940s (Hallam and Thurgate 1992). For example, the Eight Mile Creek swamp was formerly surrounded Ewens Ponds. It was largely cleared and drained in the 1940s for soldier settlement so that today only small remnants of the original swamp vegetation remain along the margins of the ponds and creek.

Groundwater Extraction for Irrigated Agriculture
A recent investigation (Wood 2011) has confirmed the view of previous authors (e.g. Hallam and Thurgate 1992; Thurgate 1995) that the groundwater expression at KRS sites is derived from the unconfined aquifer of the Gambier Limestone. In the lower South East of SA, groundwater of the unconfined aquifer flows in a south-westerly to southerly direction (Wood 2011.). Due to their location close to the coast, most APCLC sites are 'downstream' of agricultural areas.

Introduced Species
Several introduced species have been recorded in the KRS Wetlands. Escaped trout from an adjacent trout farm have been seen in Ewen Ponds, which can have a deleterious impact to native fishes inhabiting the wetlands. DENR staff and local divers helped remove this threat but this incident highlights the importance of vigilance against the accidental introduction of exotic fish species into the system.

The exotic plant, Water cress (*Nasturtium officinale*), has been found growing around ponds of KRS Wetlands and it can displace native plant species associated with the system. Williams grass (*Festuca arundinacea*) is an invader of wet grassy sedgelands associated with the KRS Wetland Community. Italian Buckthorn (*Rhamnus alaternus*) and Mirror Bush (*Coprosma repens*) may potentially invade and displace native plants in the peripheral peat swamps of the KRS. Animal pest such as foxes (*Vulpes vulpes*), cats (*Felis catus*), rabbits (*Oryctolagus cuniculus*), house mouse (*Mus domesticus*), and black rat (*Rattus rattus*) are also known to occur across the KRS Wetland Community.

Climate Change
Climate change presents both a current and future threat to the KRS. Since 1950 most of the South East of SA has experienced a trend of declining total annual rainfall of 5 – 10 mm per decade (Suppiah *et al.* 2006). Mean annual temperature in the South East is predicted to increase by 0.4 – 1.1°C by 2030 and by 0.9 – 3.5°C by 2070 (Suppiah *et al.* 2006). Mean annual rainfall is predicted to decrease by 1 – 10% by 2030 and by 2 – 30% by 2070 (Suppiah *et al.* 2006). These ranges represent best-case and worst-case scenarios.

There appears to be a relationship between rainfall and groundwater discharge at KRS sites. For example, there has been a trend of declining discharge from Piccaninnie Ponds since the early 1990s that appears to correspond with declining rainfall (Figure 4). A similar relationship has been observed for Ewens Ponds (Wood 2011). Discharge from Cress Creek ceased for the first time in living memory during summer 2008/09 (C. Harding, DWLBC, pers. com., 10/3/2009). Increased temperature and reduced rainfall due to climate change are likely to decrease recharge of the unconfined aquifer and exacerbate the threat of reduced groundwater discharge at KRS sites.

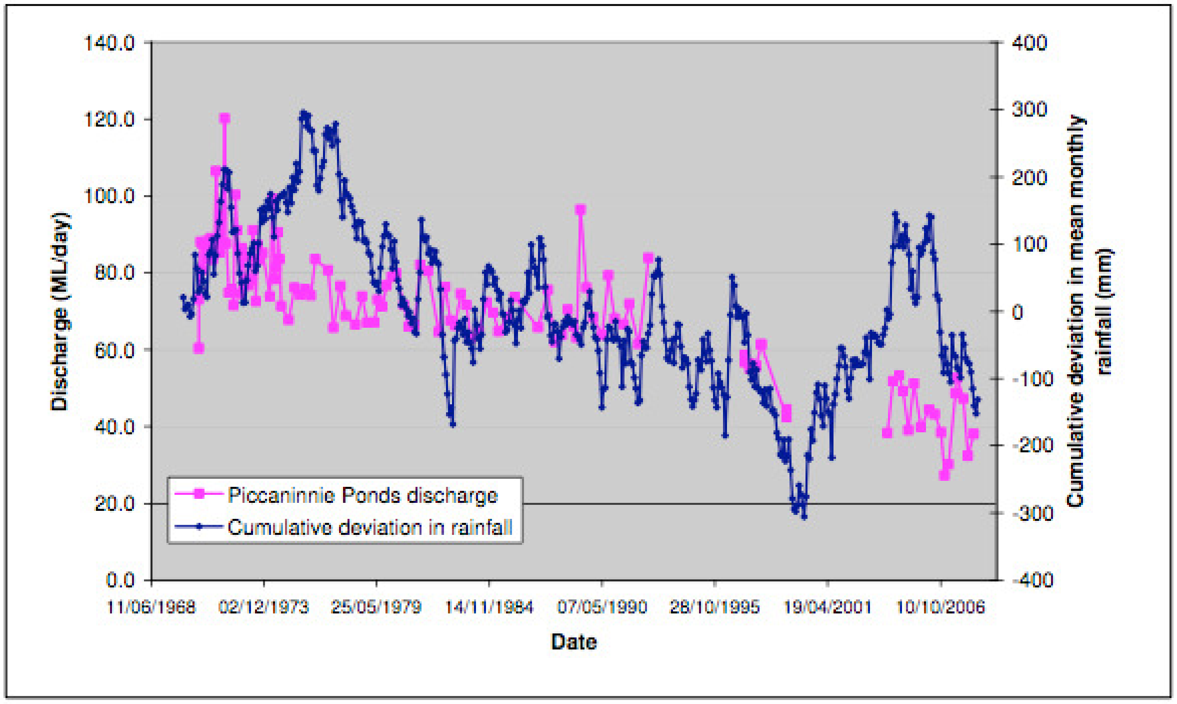

Figure S20. 4. Piccaninnie Ponds discharge and cumulative deviation in mean monthly rainfall measured at Mount Gambier (Wood 2011).

Ecosystem collapse

For assessment of criterion A and B, we assumed the Karst Rising Springs will collapse when their mapped distribution declines to zero. Drying of the springs is the most salient threat to the ecosystems because they are a water-dependant ecosystem. To assess environmental degradation under criterion C, we identified the daily spring discharge rate as a suitable variable, with a collapse threshold assumed to be 30-38 megalitres per day based on limits of acceptable change defined by Butcher et al. (2011).

ASSESSMENT

Summary

Criterion	A	B	C	D	E	Overall
sub-criterion 1	LC	CR	CR	DD	DD	CR
sub-criterion 2	DD	EN	CR	DD		
sub-criterion 3	EN	VU	DD	DD		

Criterion A

The extent of the Karst Rising-Spring Wetland Community of the South East has been mapped using GIS (ArcMap). The process of identification involved a review of the Groundwater Dependent Ecosystems (GDE) listed wetlands within the Lower Limestone Coast Water Allocation Plan, combined with expert knowledge as to the location of Karst Rising Springs. Where a KRS was identified, in general the greater wetland feature / polygon (from DENR Landscape.Wetlands SDE layer) was chosen. A final check involved buffering the Karst sinkhole data which was then overlaid by

intersection with the landscape wetlands features and reviewed for flow (K. Mott, DfW, pers. comm. 05/05/2012). No additional features were identified.

Current decline. Based on the most current mapping, the area of the KRS Wetland Community is estimated to be 7.44 km^2 (744 ha). Two known KRS Wetland Communities (Nene Valley and Blackfellows Caves) have dried up in the last 50 years, and this accounts for a 15% decline in distribution. The status of the community is therefore Least Concern (LC) under A1.

Future decline. There are no data to estimate the future declines in extent of KRS wetlands under this criterion. The status of the community is therefore Data Deficient (DD) under A2.

Historic decline. The comparison of the current distribution of Rising Spring Karst Wetland Community with that of its pre European form was made using data of associated peat fen vegetation (Silky Tea Tree vegetation association) of known and identified KRS Wetlands as surrogate measure (Figure 5). Based on this, the pre European estimate for the area of this community was 19.98 km^2 (1998 ha). With the present area estimated to be 7.44 km^2 (744 ha), this represents an ~63% historical decline in area. The status of the community is therefore Endangered (EN) under A3.

Criterion B

Extent of occurrence. The Karst Rising-Spring Wetland Community has an Extent of Occurrence (EOO) of 1347.07 km^2 (134,707 ha) based on the most recent mapping (Figure 6). There is evidence that the KRS Wetland Community is under continuing threat due to decrease in rainfall and groundwater discharge, which may be caused by over extraction and/or global climate change. For example, two KRS systems dried up within the last 50 years (see above). The threatening process of invasive species is also present in the KRS Wetlands which threatens native flora and fauna associated with this EC. The status of the KRS Wetland Community is therefore Critically Endangered (CR) under B1b.

Area of occurrence. Superimposing a 10 km grid over the mapped polygons of KRS Wetland Community (Figure 6) indicates that they are present within 7 grid cells. Of these, 4 grid cells contain less than 1 km^2 of the community (i.e. <1% of the area of a grid cell). Excluding these small occurrences, the KRS is therefore estimated to occupy three 10 × 10 km grid cells, which makes them Endangered (EN) under criterion B2b.

Number of locations. Based on the mapping of distribution and their Area of Occurrence (AOO), the KRS Wetland Community occurs in three locations. The springs are prone to effects of human acitivities including extraction of groundwater and surface water and declines in water quality due to catchment runoff, such that they may become Critically Endangered within the near future. The status of the community is therefore Vulnerable (VU) under B3.

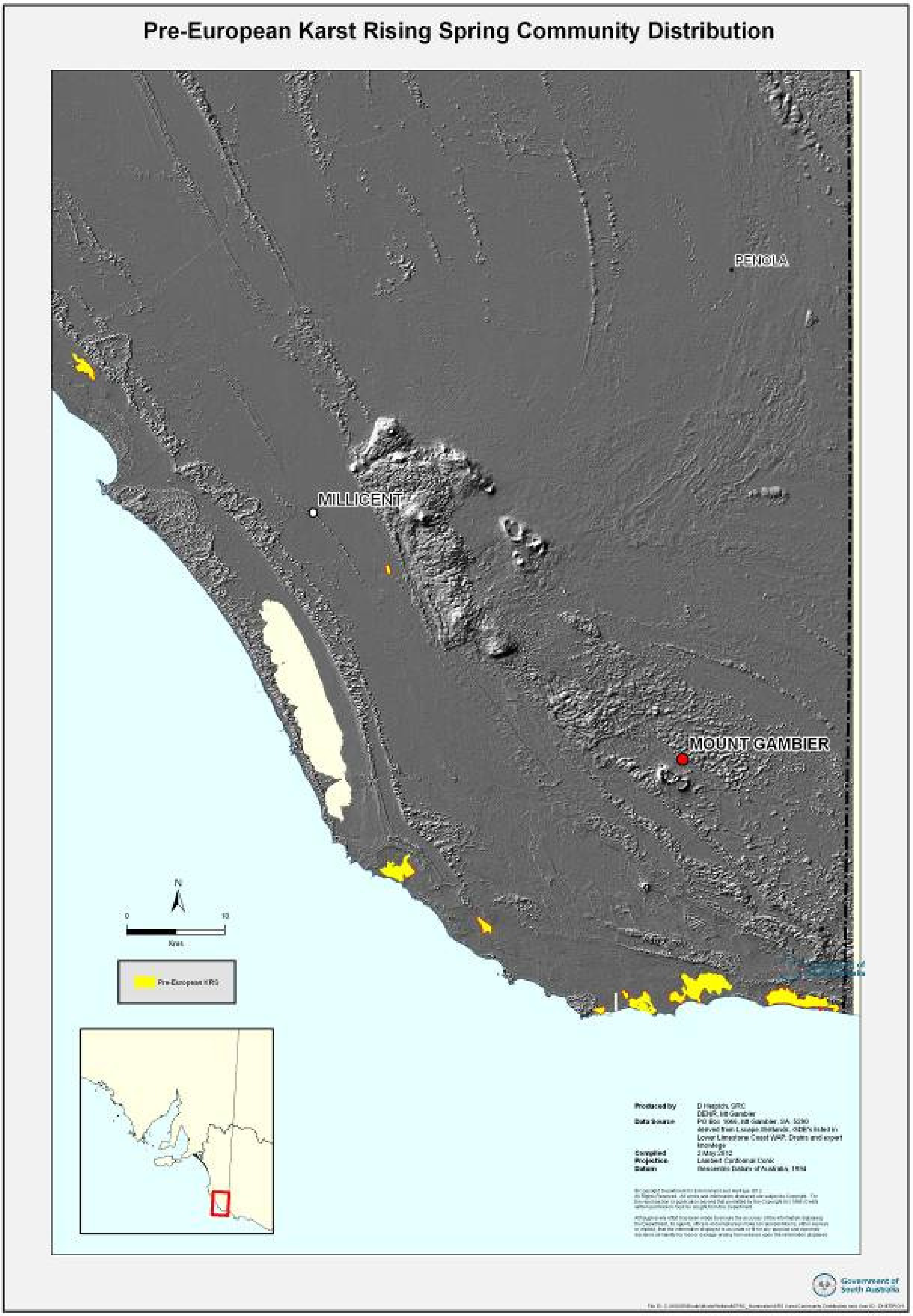

Figure S20. 5. The Pre-European distribution of Karst Rising Spring Wetland Community in South East of South Australia.

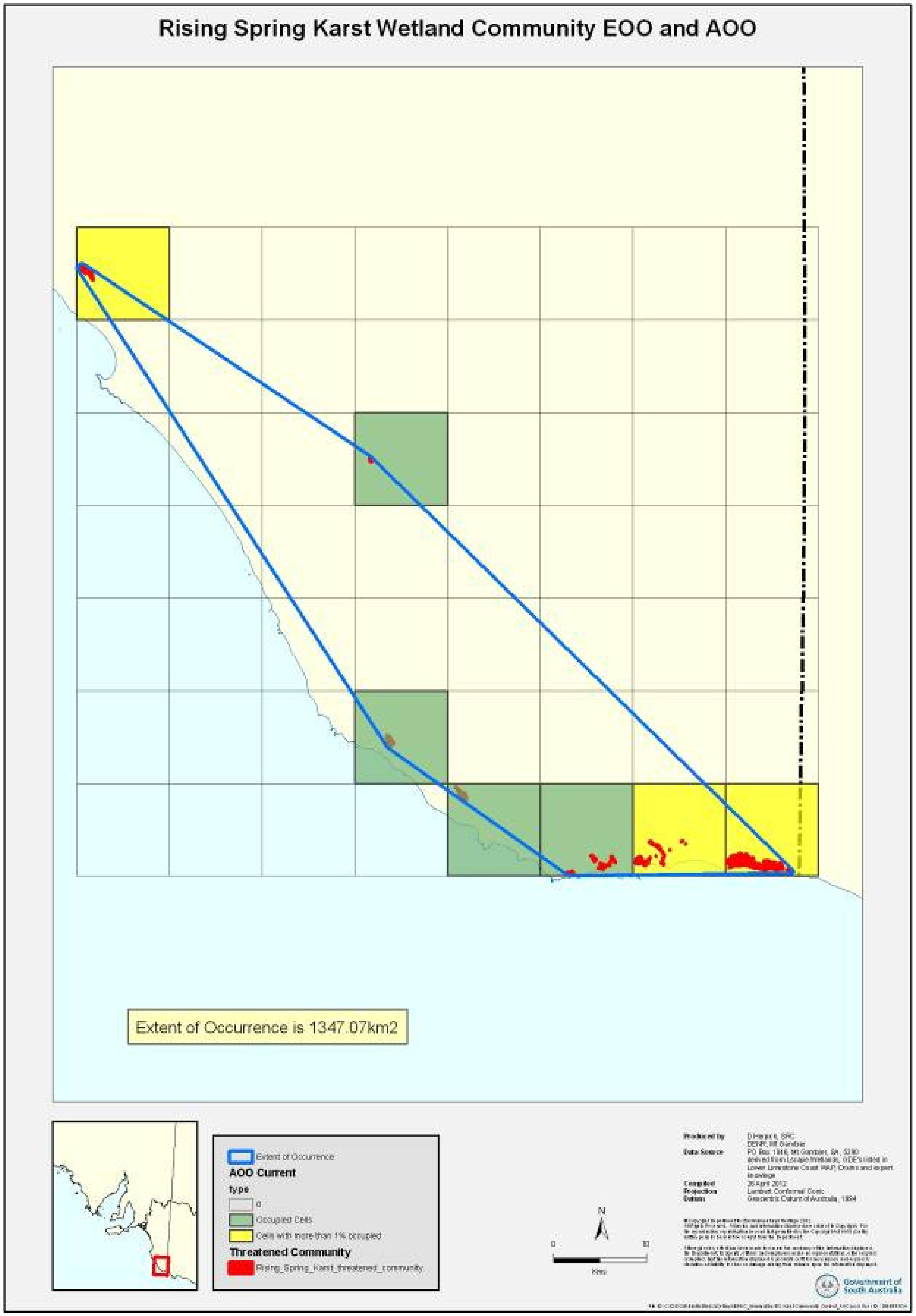

Figure S20. 6. Extent of occurrence and Area of occupancy of Karst Rising Spring wetland communities.

Criterion C

The principal mechanism of environmental degradation is through decline in hydrological processes related to unsustainable extraction of groundwater, draining and global climate change. Suitable hydrological variables for assessing criterion C include ground water discharge volumes from spring pools and flow volume measurement from natural drainage channels. Ground water discharge and stream flow data are available, but only for a few sites.

Current decline: There is insufficient data regarding decline of ground water discharge in all KRS Wetland Community in the south east. However, the decline in discharge has been demonstrated in two KRS sites (Picaninnie Pond and Eight Mile Creek). In particular, the results from Outlet Creek (which drains the Piccaninnie Ponds system) show a decrease in discharge over the past decade from an average daily discharge of between 60 and 80 megalitres per day in the 1970s and 1980s to between 30 and 50 megalitres per day since 2005. The decline in discharge translated to 38% to 50% decline in 35 years. The Ecological Character Description (ECD) of the Piccaninnie Pond Wetland System has an estimated limit of acceptable change in regards to daily discharge rate, which is from 30 to 38 megalitres a day (Butcher et al. 2011). The limit of 30-38 megalitres a day is assumed to be the bounded threshold of collapse for this system (c, green dashed line). Based on a best-fit linear model of the discharge data (red unbroken line), the current average flow (b, green dotted line) was estimated to be 40 ML/yr and the initial average flow (c, green dashed/dotted line) was estimated to be 85.7 ML/yr (Figure 7). A standardised estimate of the relative severity of degradation over the past 42 years is thus 100*(c-b)/(c-a) = 100*(85.7-40)/(85.7-30) = 82% assuming a collapse threshold of 30 megalitres per day or 100*(85.7-40)/(85.7-38) = 96% assuming a collapse threshold of 30 megalitres per day. This estimate is likely to be underestimate the severity of degradation over the past 50 years if decline in discharge commenced prior to 1970.

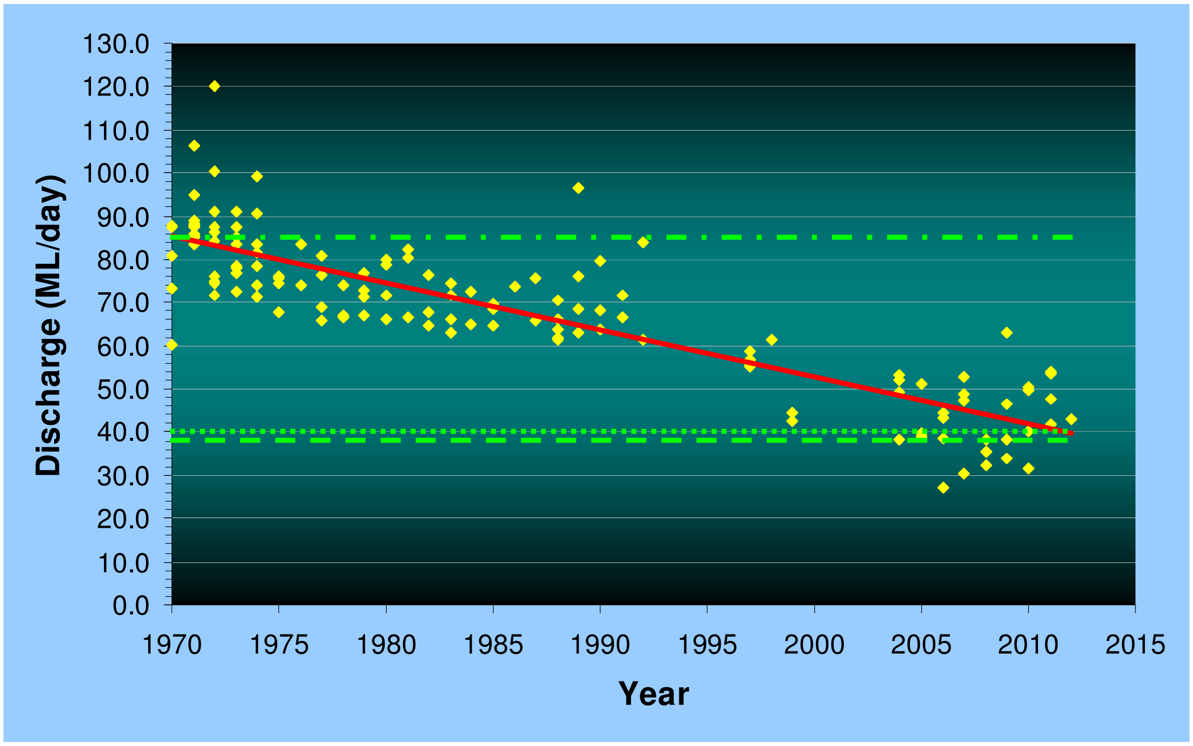

Figure S20. 7. The estimation of relative severity of environmental decline in the Karst Rising-Spring Wetland Community using groundwater discharge data at Piccaninnie Pond (data supplied by Department for Water).

The computation of the extent and relative severity of the decline in discharge was only possible in one site (Piccaninnie Pond) due to a lack of data for other sites. Nevertheless, there is historical evidence that alteration in surface water flow has resulted in significant change in a KRS Wetland Community. For example, there has been extensive modification to surface water drainage in the past 30 years. The area that is now comprised of the Piccaninnie Ponds Conservation area, Pick Swamp and adjoining farm land, was once a continuous wetland complex with large areas of surface water. Originally a natural drainage channel, Freshwater Creek, drained the system eastward into the Glenelg estuary. In 1906 the course of the creek changed as it broke through a depression in the dunes and flowed out to the sea approximately 1.2 kilometres west of the Glenelg River (Scholz 1987). Sometime in the period 1917-1945 the Western wetland became separated from the Main Ponds, and a drain was subsequently cut through the dunes from the Main Ponds (Scholz 1987). Freshwater Creek stopped flowing around 1958, potentially as a result of choking by drifting sand (Scholz 1987). Assuming that the trend of decreasing groundwater discharge at Piccaninnie Pond is applicable to the whole KRS Wetland Community, environmental degradation has occurred with a relative severity of 82-96% over 100% of the extent of the ecosystem and the threat status of the community is Critically Endangered (CR) under criterion C1.

Future decline. A preliminary assessment of the potential vulnerability of coastal wetlands to the impacts of sea level rise (SLR) and salt water intrusion (SWI) arising from climate change in the Lower Limestone Coast Prescribed Wells area has been undertaken (S. Mustafa, DfW, pers. com., 19/4/2012). A comparative analysis for the Karst Rising-Spring Wetland Community can be performed by isolating this ecosystem and conducting a separate analysis on the impact of salt water intrusion.

It has been suggested that KRS Wetland Communities near the coast are among the most vulnerable systems to salt water intrusion (SWI). Two KRS systems, Snuggery and Death Hole, have been suggested as the only sites that may not be impacted by SWI (S. Slatter and S. Mustafa, DfW, pers. comm. 19/04/2012). Therefore of the 21 current KRS sites, 19 sites may be impacted, representing 90.5% impact or 85.5% of the KRS area.

For Sea Level Rise (SLR), the approach adopted both a 2 and 3m AHD scenario. This height was based on:

- a projected sea level rise of 0.5m by 2070 (Short et al. 1995)
- the maximum high tide observation of 1.535m (based on 1981 record for Victor Harbour taken from the National Tidal Facility)
- applying an additional safety buffer of 1m to account for storm surges/waves and erroneously high elevations in the Digital Elevation Model (resulting from vegetation interference with the LiDAR).

The first two dot points make up the 2m scenario, whist the third together with the other points forms the 3m sea level rise scenario. A KRS Wetland Community layer was produced that contained all polygons with elevations equal to or less than both the 2 and 3m AHD scenarios. KRS from this layer that were located within the area boundary were selected to give an indication of the GDEs within the study area that could potentially be impacted by SLR resulting from Climate Change (Figure 8).

For a 2m sea-level rise, 8 out of the 21 KRS sites were projected to be inundated, representing 38% of identified KRS systems. For a 3m sea-level rise, 14 out of the 21 KRS sites were projected to be inundated, representing 66% of identified KRS systems. Note the above modelling scenario excludes preferential conduits for flow i.e. drains.

The analysis showed that of the 21 KRS Wetland Community polygons mapped in the study region, 19 of them have elevations ≤3m AHD, thus they are at risk of salt water intrusion under a climate change

scenario. This is translated to 85.5% of the area of KRS system projected to be impacted by salt water intrusion by 2070, 8 years beyond the 50-year assessment time frame. Given this projected extent of degradation, and assuming that inundation with sea water results in degradation with relative severity of 100%), the status of the community is Critically Endangered (CR) under criterion C2.

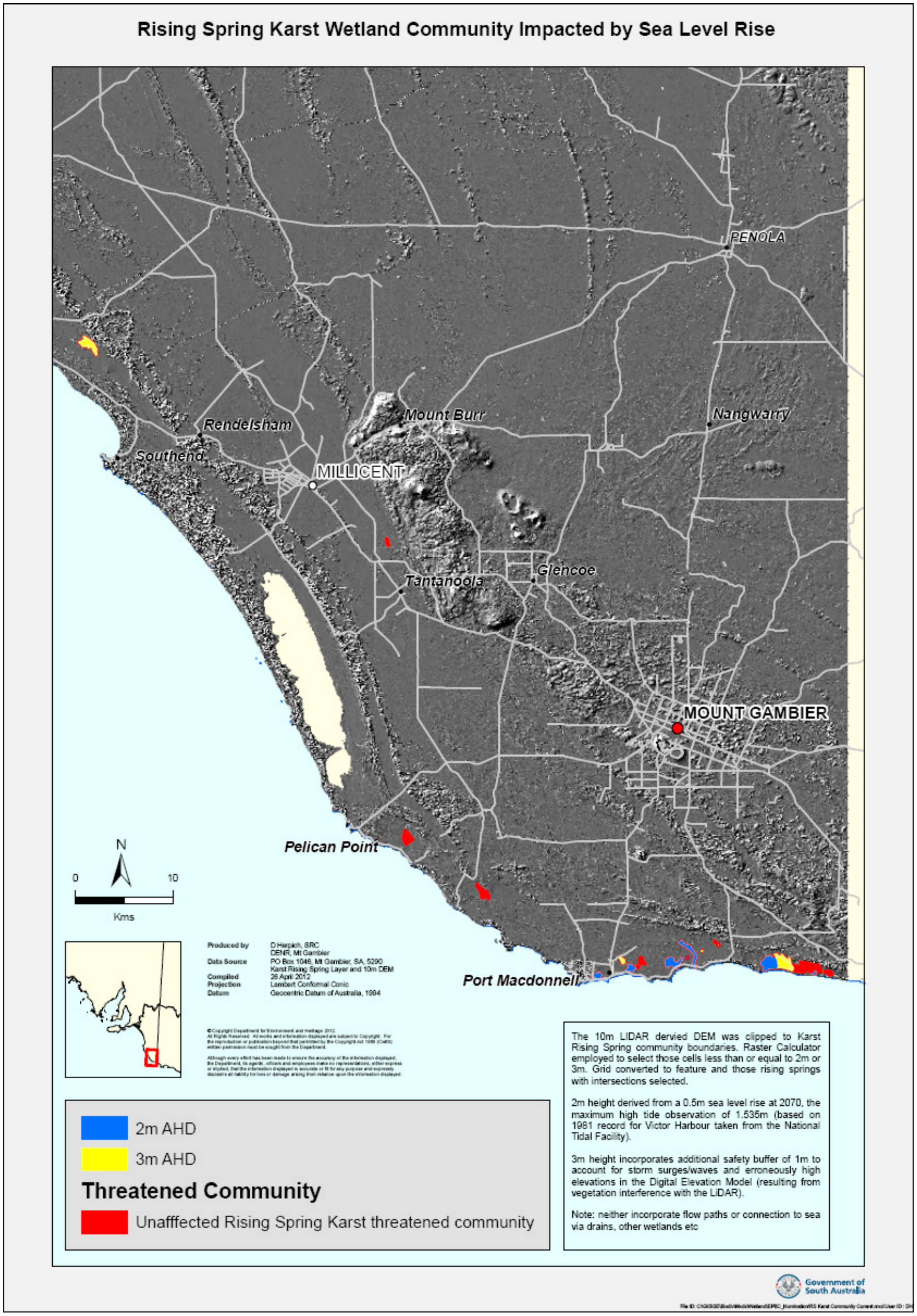

Figure S20. 8. Risk analysis of KRS Wetland Community to salt water intrusion under climate change scenario.

Historic decline. There are no data available to estimate the parameter under this criterion. The status of the community is therefore Data Deficient (DD) under this criterion.

Criterion D

The principal mechanism of disruption in biotic interaction in this ecological community is the change in the biotic component due to change in ground water discharge. The conceptual model of the Karst Rising Spring Wetland Community (Figure 2) shows that reduction in discharge will result in drier peat that is unsuitable substrate to support vegetation associated with KRS. This can potentially allow for invasion of the terrestrial shrub species *Acacia longifolia* var. *sophorae,* seen at Picaninnie Ponds. However, the data are insufficient to quantify the extent and relative severity of disruption in biotic interaction.

Current decline. There is insufficient data to estimate the parameter under this criterion. The status of the community is therefore Data Deficient (DD) under this criterion.

Future decline. There is insufficient data to estimate the parameter under this criterion. The status of the community is therefore Data Deficient (DD) under this criterion.

Historic decline. There is insufficient data to estimate the parameter under this criterion. The status of the community is therefore Data Deficient (DD) under this criterion.

Criterion E

No quantitative analysis has been carried out to assess the risk of EC collapse to Karst Rising-Spring Wetland Community. The status of the EC is therefore Data Deficient (DD) under criterion E.

REFERENCES

Allen, G. R., Midgeley, S. H. and Allen, M. (2002). *Field Guide to the Freshwater Fishes of Australia.* (Western Australian Museum: Perth, Western Australia).

Bachmann, M. R. (2002). *Silky Tea-tree and Cutting Grass Wetland Rehabilitation Project 1999-2002*. (Nature Conservation Society of South Australia: Adelaide).

Bachmann, M. R. (2004). *Protection and management of a karst rising-spring wetland reference area of international significance - Restoration of the Piccaninnie Ponds wetland system (proposed Ramsar site) and the Glenelg River estuary in SA and Victoria*. (Department for Environment and Heritage: Mount Gambier, South Australia).

Bachmann, M. R. and van Weenen, J. (2001). *The distribution and status of the swamp antechinus in South Australia*. (Nature Conservation Society of South Australia: Adelaide, South Australia).

Beck, R. G. (1985). Field observations upon the Dwarf Galaxiid *Galaxiella pusilla* (Mack) (Pisces: Galaxiidae) in the South-east of South Australia, Australia. *South Australian Naturalist* 60(1): 12-22.

Birds South East (2009). *Unpublished Data: Bird Diversity and Abundance at Pick Swamp, May 2007 to February 2009*. (Birds South East: Mt Gambier, South Australia).

Butcher, R., Hale, J. and Cottingham, P. (2008). *Draft ecological character description for Piccaninnie Ponds Karst Wetlands. Prepared for DEH, Mt Gambier*. (Department for Environment and Heritage: Mount Gambier, South Australia).

Butcher, R., Hale, J., and Cottingham, P. (2011). Ecological character description for Piccaninnie Ponds Karst Wetlands. Prepared for the Department of Environment and Natural Resources, Mt Gambier.

Cann, J. H., De Decker, P. and Murray-Wallace, C. V. (1991). Coastal Aboriginal shell middens and their paleoenvironmental significance, Robe Range, South Australia. *Transactions of the Royal Society of South Australia* 115(4): 161-175.

Croft, T. and Carpenter, G. (2001). *Draft Biological Resources for the South East Region, South Australia*. (Department for Environment and Heritage: Adelaide, South Australia).

Drexel, J.F., and Preiss, W.V. (Eds) (1995). The geology of South Australia. Vol 2, The Phanerozoic. Geological Survey of South Australia, Bulletin 54

DSE (2005). *Advisory List of Rare of Threatened Plants in Victoria*. (Victorian Department of Sustainability and Environment: East Melbourne, Victoria).

DSE (2006). *Index of Wetland Condition. Assessment of wetland vegetation. Update - March 2006.* (Department of Sustainability and Environment: Melbourne, Victoria).

DSE (2007). *Advisory List of Threatened Vertebrate Fauna in Victoria - 2007.* (Department of Sustainability and Environment: East Melbourne, Victoria).

Eardley, C. M. (1943). An Ecological Study of the Vegetation of Eight Mile Creek Swamp. A South Australian Coastal Fen Formation. *Transactions of the Royal Society of South Australia* 67(2): 200-223.

Ecological Associates (2008). *Final Report - Floristic Mapping of the Piccaninnie Ponds Conservation Park and Pick Swamp*. (Department for Environment and Heritage: Mt Gambier, South Australia).

Grund, R. (1998). *South Australian Butterflies Checklist.* (R. Grund: Adelaide, South Australia) Last Updated: October 2004. Accessed 12/3/2009.**http://users.sa.chariot.net.au/~rgrund/checklist.htm**.

Hallam, N. D. and Thurgate, M. E. (1992). *The Biology of Piccaninnie and Ewens Ponds and Woolwash, Blacks and Gouldens Holes, South Australia*. (Monash University: Clayton, Victoria).

Hammer, M. (2002). *The South East Fish Inventory: Distribution and Conservation of Freshwater Fishes of South East South Australia*. (Native Fish Australia (SA) Inc: Adelaide, South Australia).

Hammer, M. (2008). *Pilot fish movement study in Lower South East, South Australia. Report to Department for Environment and Heritage, South Australian Government.* (Aquasave Consultants: Adelaide, South Australia).

Hammer, M., Skinner, N. and Playford, T. (2004). *Observations on the 'Mechanical Dragging' of Eight Mile Creek, South-east South Australia. Report to the South Eastern Water Conservation and Drainage Board*. (Aquasave Consultants: Adelaide, South Australia).

Harris, W. K. (1983). Geology. In: *Natural History of the South East.* (Ed. M. J. Tyler, C. R. Twidale, J. K. Ling and J. W. Holmes). (Royal Society of South Australia Inc.: Adelaide, South Australia).

Haywood, B. T. and Natt, V. (2006). First confirmed observation of *Heteronympha cordace wilsoni* Burns (Lepidoptera: Nymphalidae: Satyrinae) in South Australia, *Australian Entomologist* 33: 5-7.

James, N.P. and Bone, Y. (1989). Petrogenesis of Cenozoic, temperate water calacrenites, South Australia: a model for meteoric/shallow burial digenesis of shallow water calcite sediments. *Journal of Sedimentary Petrology* 59:191-203.

Khan, M. T., Khan, T. A. and Wilson, M. E. (2004). Habitat use and movement of river blackfish (*Gadopsis marmoratus* R.) in a highly modified Victorian stream, Australia. *Ecology of Freshwater Fish* 13: 285-293.

Marker, E. M. (1975). *The Lower South East of South Australia: A Karst Province*. (Department of Geography and Environmental Studies, University of Witwatersand: Johannesberg, South Africa).

Morcombe, M. (2003). *Field Guide to Australian Birds.* (Steve Parish Publishing: Archerfield Qld).

Peterson, G. (2005). *Swamp skink (Egernia coventryi) and Salamander Skink (Nannoscincus maccoyi) survey of the south east of South Australia Autumn and Spring 2004*. (Department for Environment and Heritage South Australia: Mount Gambier SA).

Scholz, O. (1987). *The Biology of Piccaninnie Ponds and Ewens Ponds, South Australia, with reference to recent disturbances in the aquatic vegetation at Piccaninnie Ponds.* (Honours) (Botany Department, Monash University: Melbourne, Victoria).

Scholz, O. (1990). Physicochemistry and Vegetation of Piccaninnie Ponds, a Coastal Aquifer-fed Pond in South-eastern South Australia. *Australian Journal of Marine and Freshwater Research* 41: 237-246.

Short, F.T., Burdick, D.M., Kaldy, J.E. (1995) Mesocosm experiments quantify the effects of eutrophication on eelgrass, *Zostera marina* L. *American Society of Limnology and Oceanography* 40: 740-749.

Simpson, K. and Day, N. (1996). *Field Guide to the Birds of Australia.* (Viking Penguin: Ringwood, Victoria).

SKM (2009). *Draft Report - Classification of groundwater-surface water interactions for water dependent ecosystems in the South East, South Australia. Report prepared for the Department of Water, Land and Biodiversity Conservation.* (Sinclair Knight Merz: Hobart, Tasmania).

Suppiah, R., Preston, B., Whetton, P. H., McInnes, K. L., Jones, R. N., Macadam, I., Bathols, J. and Kirono, D. (2006). *Climate change under enhanced greenhouse conditions in South Australia. An updated report on: Assessment of climate change, impacts and risk management strategies relevant to South Australia.* (Climate Impacts and Risk Group, CSIRO Marine and Atmospheric Research: Aspendale, Victoria).

Thurgate, M. E. (1995). *Sinkholes, Caves and Spring Lakes - An Introduction to the Unusual Aquatic Ecosystems of the Lower South East of South Australia. South Australian Underwater Speleological Society Occassional Paper Number 1.* (SAUSS: Adelaide, South Australia).

Wood, C. (2011). *Measurement and evaluation of key groundwater discharge sites in the Lower South East of South Australia,* DFW Technical Report 2011/14, Government of South Australia, through Department for Water, Adelaide.

Appendix A. List of fauna species associated with KRS Wetland Community

Species	Common Name	Listing[1]			Source	Nature of Dependence
		EPBC[2]	SA[3]	Vic[4]		
MAMMALS						
Antechinus minimus maritimus	Swamp Antechinus		E	L, NT	(Bachmann 2004)	In SA occurs almost exclusively in two vegetation types, both of which are often associated with APCLC; Silky Tea-tree Wet Shrubland and *Gahnia* spp. Sedgeland (Bachmann and van Weenen 2001).
BIRDS						
Anas rhynchotis	Australasian Shoveler		R		(Birds South East 2009)	Widely distributed through southern and eastern Australia (Morcombe 2003). Occurs in a broad range of wetland types but prefers permanent waterbodies with dense reeds and shrubs (Morcombe 2003). Flocks of over 100 recorded at least in one KRS site (Pick Swamp) (Birds South East 2009).
Anseranas semipalmata	Magpie Goose		E	L, NT	(Birds South East 2009)	Widely distributed throughout Australia (except arid inland), declining in south eastern Australia (Morcombe 2003). Occurs in a broad range of wetland types, prefers shallowly inundated areas (Morcombe 2003). Flocks of up to 30 recorded at least in one KRS site (Pick Swamp) (Birds South East 2009).
Ardea alba	Great Egret			L	(Birds South East 2009)	Widespread and common throughout Australia (except western deserts) in suitable habitat (Morcombe 2003). Habitat includes wetlands, estuaries and shallow marine waters (Morcombe 2003). Recorded in both open wetland and shrubland areas of at least in one KRS site (Pick Swamp) (Birds South East 2009).
Biziura lobata	Musk Duck		R	V	(Birds South East 2009)	Widely distributed through southern Australia (Morcombe 2003). Occurs in deep permanent lakes and swamps with dense reedbeds and open water (Morcombe 2003). Recorded in relatively high numbers (over 30) at least in one KRS site (Pick Swamp) (Birds South East 2009).
Botaurus poiciloptilus	Australasian Bittern		V	L, E	(Birds South East 2009)	Widely distributed throughout Australia but in decline. Requires dense vegetation (e.g. shrubs, reedbeds, sedges) in freshwater wetlands.
Calyptorhynchus funereus	Yellow-tailed Black-Cockatoo		V		(Birds South East 2009)	Widely distributed through south eastern Australia (Morcombe 2003). Typical habitat is *Eucalypt* forests and woodlands (Morcombe 2003). Occassional visitor to Silky Tea-tree Wet Shrubland at APCLC sites (Birds South East 2009).

Species	Common Name	Listing[1]			Source	Nature of Dependence
		EPBC[2]	SA[3]	Vic[4]		
Chlidonias hybridus javanicus	Whiskered Tern			NT	(Birds South East 2009)	Widely distributed and common through most of Australia (Morcombe 2003). Shallow freshwater wetlands are a preferred habitat (Morcombe 2003). Flocks of over 1000 have been recorded at least in one KRS site (Pick Swamp) (Birds South East 2009).
Dasyornis broadbenti broadbenti	Rufous Bristlebird		R	L, NT	(Bachmann 2002)	Occurs in scattered near-coastal localities from the Murray Mouth to Anglesea (Vic) (Bachmann 2002). Requires dense coastal or wet shrubland vegetation, including Silky Tea-tree Wet Shrubland, particularly for protection of its nest that is constructed on the ground (Bachmann 2002).
Falco peregrinus	Peregrine Falcon		R		(Birds South East 2009)	Widespread in Australia but rare (Morcombe 2003). Habitat generalist (Morcombe 2003). Recorded in low abundance at least in one KRS site (Pick Swamp) (Birds South East 2009).
Gallinago hardwickii	Latham's Snipe		R	NT	(Birds South East 2009)	Widely distributed through Australia (except arid interior) (Morcombe 2003). Regular summer migrant (breeds in Japan). Preferred habitat is low vegetation around wetlands (Morcombe 2003). Occasionally recorded in low abundance in Silky Tea-tree Wet Shrubland at least in one KRS site (Pick Swamp) (Birds South East 2009).
Grus rubicundus	Brolga		V	L, V	(Birds South East 2009)	Widespread throughout eastern and northern Australia (Simpson and Day 1996). Nests in shallow freshwater marshes. Breeding record for at least in one KRS site (Piccaninnie Ponds) (Butcher *et al.* 2008).
Neophema chrysogaster	Orange-bellied Parrot	CE	E	L, CE	(Bachmann 2004)	Known to roost regularly in *Leptospermum lanigerum* shrubland at least in one KRS site (Piccaninnie Ponds)(Bachmann 2004; Butcher *et al.* 2008).
Neophema chrysostoma	Blue-winged Parrot		V		(Birds South East 2009)	Range includes eastern SA, western NSW and most of Victoria and Tasmania (Morcombe 2003). Habitat generalist (Morcombe 2003). Has been recorded in low to moderate numbers (< 100) in both open wetland and shrubland at least in one KRS site (Pick Swamp) (Birds South East 2009).
Oxyura australis	Blue-billed Duck		R	L	(Birds South East 2009)	Widespread but uncommon through south eastern and south western Australia (Morcombe 2003). Breeds in deep, permanent, densely vegetated freshwater wetlands, winters on more open waters (Morcombe 2003). Recorded in numbers up

Species	Common Name	Listing[1]			Source	Nature of Dependence
		EPBC[2]	SA[3]	Vic[4]		
						to 30 in open wetland areas of at least in one KRS site (Pick Swamp) (Birds South East 2009).
Pachycephala olivacea hesperus	Olive Whistler		E		(Bachmann 2002)	Typical of moist, temperate, forested environments, the lower South East of SA is at the western fringe of this species range (Bachmann 2002). Silky Tea-tree Wet Shrubland provides favourable habitat in the region. The Glenelg River sub-species has been recorded only from a small number of locations between Beachport (SA) and Port Fairy (Vic) (Bachmann 2002).
Pezoporus wallicus wallicus	Ground Parrot		E	L, E	(Bachmann 2002)	In SA occurred only in or near Silky Tea-tree Wetland but now presumed extinct (Croft and Carpenter 2001). Persists at Long Swamp in Victoria (Bachmann 2002). Potential for re-establishment in SA if suitable habitat could be reinstated (Bachmann 2004).
Platalea redia	Royal Spoonbill			V	(Birds South East 2009)	Widely distributed throughout Australia (except western deserts) (Morcombe 2003). Prefers shallow wetlands or margins of deeper waterbodies (Morcombe 2003). Recorded in low numbers throughout the year at least in one KRS site (Pick Swamp) (Birds South East 2009).
Plegadis falcinellus	Glossy Ibis		R	NT	(Birds South East 2009)	Widely distributed throughout Australia. Common in coastal north, uncommon and nomadic elsewhere (Morcombe 2003). Occurs in a range of wetland types preferring shallow areas (Morcombe 2003). Flocks of over 30 recorded at least in one KRS site (Pick Swamp) (Birds South East 2009).
Podiceps cristatus	Great Crested Grebe		R		(Birds South East 2009)	Widely distributed throughout Australia except arid interior (Morcombe 2003). Prefers well vegetated margins of open waters such as large lakes and reservoirs (Morcombe 2003). Record of one individual in open wetland area at Pick Swamp (Birds South East 2009) but may utilise open water of spring pools at other sites.
Rallus pectoralis pectoralis	Lewin's Rail		V	L, V	(Bachmann 2002)	Widely distributed through eastern and southern coastal Australia (Simpson and Day 1996). Occurs in a broad range of wetland types that feature dense emergent or fringing vegetation (Bachmann 2002). Known to occur at a number of KRS sites (Bachmann 2002).
Stagonopleura bella	Beautiful Firetail		R		(Birds South East	Distributed through coastal NSW, Victoria and SA and throughout Tasmania, where it is more common (Morcombe 2003). Preferred habitat is dense wetland vegetation

Species	Common Name	Listing[1]			Source	Nature of Dependence
		EPBC[2]	SA[3]	Vic[4]		
					2009)	(Morcombe 2003). Single record of <3 individuals in Silky Tea-tree Wet Shrubland at Pick Swamp (Birds South East 2009).
Stipiturus malachurus polionotum	Southern Emu Wren (southeast SA subspecies)		R		(Bachmann 2004)	Widely distributed throughout near-coastal south-eastern Australia (Simpson and Day 1996). Favoured habitats include *Gahnia* spp. sedgelands of the KRS (Bachmann 2002).
AMPHIBIANS						
Litoria raniformis	Southern Bell Frog	V	V	L, E	(Bachmann 2002)	Widely distributed through south-eastern mainland Australia and northern Tasmania (Cronin 2001). Usually occurs at permanently inundated sites with emergent vegetation. Known to occur at several KRS sites (Bachmann 2002).
REPTILES						
Egernia coventryi	Swamp Skink		E	L, V		Occurs throughout southern Victoria, with the lower South East of SA marking the western limit of its range (Bachmann 2002). In SA has only ever been recorded in Silky Tea-tree Wet Shrubland between Southend and the Victorian border (Bachmann 2002).
Nannoscincus maccoyi	Salamander skink		E		(Bachmann 2002)	Typically occurs in the cool, moist forested uplands of southern Victoria and south-east NSW, populations in South East SA mark the western limit of the species range (Bachmann 2002). These populations occur in KRS Silky Tea-tree Wet Shrubland (Bachmann 2002; Peterson 2005).
Pseudomoia rawlinsoni	Glossy Grass Skink		E	NT	(Bachmann 2002)	Occurs in south-eastern Australia and is at the western margin of its range in the lower South East of SA (Bachmann 2002). Known from a variety of wetland habitats in SA including *Gahnia* spp. sedgelands (Bachmann 2002).
FISH						
Anguilla australis australis	Shortfinned Eel		R			Catadromous. Widely distributed in coastal south eastern Australia. Lower South East of SA is western extremity of range. Occurs in variety of freshwater habitats including lakes, swamps and streams (Allen *et al.* 2002).
Gadopsis marmoratus	River Blackfish		P, E		(Hammer 2002)	Freshwater only. Prefers deep (40 – 60 cm (Khan *et al.* 2004)) permanently inundated habitat with high cover of vegetation or physical substrate (e.g. undercut

Species	Common Name	Listing[1]			Source	Nature of Dependence
		EPBC[2]	SA[3]	Vic[4]		
						banks (Khan *et al.* 2004)) receiving regular flows, such as spring flows (Hammer 2002). This species is locally common in KRS systems (Hammer 2002)
Galaxias brevipinnis	Climbing Galaxias		R		(Hammer 2008)	Amphidromous. Wide but disjunct distribution throughout south eastern Australia. Prefers clear, flowing water in shaded streams with rocky substrate (Allen *et al.* 2002). Known from only one KRS site (Piccaninnie Ponds) where it has been recorded in low abundance (Hammer 2008).
Galaxias truttaceus	Spotted Galaxias		E		(Hammer 2008)	Amphidromous. Range includes most of coastal Victoria and Tasmania, a small area of coastal WA and the lower South East of SA (Allen *et al.* 2002). Usually inhabits the margins of still or flowing rivers, streams and lakes. More common close to the coast (Allen *et al.* 2002). Known from several KRS sites, with a significant population recorded at Piccaninnie Ponds (Hammer 2008).
Galaxiella pusilla	Dwarf Galaxias	V		L, V	(Hammer 2002)	Freshwater only. Permanent and ephemeral habitat. May have the capacity to aestivate when surface water is absent (Beck 1985; Allen *et al.* 2002). Patchily distributed through coastal southern Victoria and south east SA (Allen *et al.* 2002; Hammer 2002). Requires dense, inundated vegetation, including sedgeland and Silky Tea-tree Wet Shrubland (Hammer 2002). Abundant at several APCLC sites, with Pick Swamp a local stronghold for the species (Hammer 2008)
Geotria australis	Pouched Lamprey		V		(Hammer 2008)	Anadromous. Range includes south west WA, South East SA, south west Victoria and Tasmania (Allen *et al.* 2002). Ammocoetes (juveniles) live in mud burrows in the upper reaches of coastal streams. Following metamorphosis adults migrate downstream to the sea. Upstream migration occurs for spawning (Allen *et al.* 2002). Recorded in low numbers at one KRS site (Piccaninnie Ponds) (Hammer 2008). Lampreys are considered rare in the South East region (Hammer 2008).
Mordacia mordax	Shorthead Lamprey		V		(Hammer 2008)	Anadromous. Distributed throughout Victoria, southern NSW, Tasmania and the South East, Mt Lofty Ranges and lower River Murray in SA (Allen *et al.* 2002). Ammocoetes live in burrows in mud or silt in slow-flowing streams. Adult's life spent mostly in estuaries or the sea. Upstream spawning occurs in fast flowing sections of rivers with a mud, sand or silt substrate (Allen *et al.* 2002). A single record from one KRS site (Ewens Ponds) (Hammer 2008). Lampreys are considered

Species	Common Name	Listing[1]			Source	Nature of Dependence
		EPBC[2]	SA[3]	Vic[4]		
						rare in the South East region (Hammer 2008).
Nannoperca obscura	Yarra Pygmy Perch	V	P, E	L, NT	(Hammer 2002)	Freshwater only. Occurs in one APCLC spring pool (Crescent Pond) (Hammer 2002). Geographic range from Frankston, Victoria to Bool Lagoon SA (Allen *et al.* 2002). Prefers flowing water with abundant aquatic vegetation in streams and small lakes.
Nannoperca variegata	Variegated Pygmy Perch	V	P, E	L, E	(Hammer 2002)	Freshwater only. Occurs in several APCLC spring pools and drains. KRS Wetland Community is core habitat for this highly restricted species. Known only from APCLC sites and the Glenelg River system of south-west Victoria. Requires small, swiftly flowing, well vegetated watercourses with cool (14° - 16°C), clear water (Allen *et al.* 2002).
Prototroctes maraena	Australian Grayling	V		L, V	(Hammer 2002)	Amphidromous. Historically reported in two KRS sites (Ewens Ponds, Piccaninnnie Ponds), marking the western extremity of their range, but not recorded since 1983 despite extensive surveys (Hammer 2002; 2008).
Pseudaphritis urvillii	Congolli		R			Catadromous. Nationally a common species widely distributed through the coastal areas of south eastern Australia (Allen *et al.* 2002). A bottom dweller of brackish estuaries but also inhabits slow-flowing freshwater streams (Allen *et al.* 2002). Recorded in low abundance at several KRS sites but also in other habitat types in the South East (Hammer 2002).
INVERTEBRATES						
Hesperilla chrysotricha cyclospila	Chrysotricha Sedge-skipper		V		(Bachmann 2002)	Larvae principally feed on Cutting Grass (*Gahnia trifida*). Range has steadily declined in South Australia. Now extinct on Yorke Peninsula, and has been pushed to the extreme southern areas on Eyre and Fleurieu Peninsulas. It is probably extinct in the Coorong and is present only in a few coastal conservation parks in the Lower South East. On Kangaroo Island it is now mostly found on the western half of the island (Grund 2000).
Hesperilla idothea idothea	Flame Sedge-skipper		LU		(Bachmann 2002)	Subspecies *idothea* is found either in *Gahnia radula* bearing forests, or in open *Gahnia clarkei* wetlands that are surrounded by a shady cover of native trees and large bushes (Grund 2000). Within SA is restricted to the Lower South East Region
Taractrocera	White-		R		(Bachman	Found in areas where its grass host plants remain in a soft green condition

Species	Common Name	Listing[1]			Source	Nature of Dependence
		EPBC[2]	SA[3]	Vic[4]		
papyria papyria	banded Grass-dart				n 2002)	throughout the year. It is fond of swampy areas, particularly in open green grassy verges next to the wetlands (Grund 1998). In South Australia found in the eastern temperate parts of the agricultural belt, and tends to be more common in the cool Lower South East Region.
Heteronympha cordace wilsoni	Bright-eyed Brown		E		(Bachmann 2002; Haywood and Natt 2006)	The only known population in SA is in the Piccaninnie Ponds/Pick Swamp system. Preferred foodplant is the Tall Sedge (*Carex appressa*). The local subspecies of the butterfly also occurs in the adjacent parts of western Victoria, in the *Carex* bogs and swamps associated with the pristine portions of the Glenelg River drainage basin (Grund 1998).
Oriexenica kershawi kanunda	Striped Xenica		V		(Bachmann 2002)	Occurs in the cool, damp shady Silky Tea-tree, *Poa* and *Gahnia clarkei* wetlands adjacent to the coast, which do not dry out over the hot summer months, and where its larval hostplant grass in the understorey remains in a green condition all year round. In SA presently known to occur only within 7 km of the coast, southwards of Millicent in the Lower South East region (Grund 1998).
Oriexenica lathoniella herceus	Silver Xenica		V		(Bachmann 2002)	In SA occurs either in damp shady forest areas or in the cool, damp shady Silky Tea-tree, *Poa* wetlands adjacent or near to the coast, which do not dry out over the hot summer months, and where its larval hostplant grass in the understorey remains in a green condition all year round (Grund 1998).
Tisiphone abeona albifascia	Sword-grass Brown		V		(Bachmann 2002)	In SA only occurs in the Lower South East region, within the remnant *Gahnia clarkei* wetlands (Grund 2000).
Erina hyncinthina form *josephina*	Common Dusky Blue		R		(Bachmann 2002)	The 'hybrid' purple form (*josephina*) is confined to the Lower South East region where the host plant of the blue form does not occur. It is most commonly seen in the coastal heathlands, and is very rarely seen further inland (Grund 1998).

[1]Status codes:
Ex: extinct
CE: critically endangered
E: endangered
V: vulnerable

R: rare
LU: local (uncommon)
P: protected
L: listed
[2]*Environment Protection and Biodiversity Conservation Act 1999*
[3]for mammals, birds, amphibians, reptiles and flora: *National Parks and Wildlife Act 1972*. For fishes: *Fisheries Act 1982* (for ‘protected’ only) and *Draft Action Plan for South Australia Freshwater Fishes 2007* (for all other status codes). For invertebrates: *South Australian Butterflies Checklist* (Grund 1998).
[4]*Flora and Fauna Gaurantee Act 1988* (for ‘listed’ only). For fauna: *Advisory List of Threatened Vertebrate Fauna in Victoria – 2007* (DSE 2007) (for all codes except ‘listed’). For flora: *Advisory List of Rare or Threatened Plants in Victoria* (DSE 2005) (for all codes except ‘listed’)
